# Docking of virtual libraries identifies small-molecule agonists of neurotensin receptors with analgesic activity

**DOI:** 10.1038/s41467-026-74990-1

**Published:** 2026-07-22

**Authors:** Nicolas Panel, Duy Duc Vo, Harald Hübner, Mattia Deluigi, Szymon Pach, Félix Bélair, Dorothee Weikert, Christoph Klenk, Mark Hilge, Niharika Shiva, Isabelle Brochu, Jean-Michel Longpré, Frida Bällgren, Aljona Saleh, Huabin Hu, Jon Kapla, Stefanie Kampen, Israel Cabeza de Vaca, Jan Kihlberg, Nina Wettschureck, Philippe Sarret, Andreas Plückthun, Peter Gmeiner, Jens Carlsson

**Affiliations:** 1 Science for Life Laboratory, Department of Cell and Molecular Biology, Uppsala University, Uppsala, Sweden; 2 Department of Chemistry-BMC, Uppsala University, Uppsala, Sweden; 3 Department of Chemistry and Pharmacy, Medicinal Chemistry, Friedrich-Alexander-Universität Erlangen-Nürnberg, Erlangen, Germany; 4 Department of Biochemistry, University of Zurich, Zürich, Switzerland; 5 Department of Pharmacology-Physiology, Faculty of Medicine and Health Sciences, Institute de Pharmacologie de Sherbrooke, Université de Sherbrooke, Sherbrooke, QC Canada; 6 FAUNeW – Research Center New Bioactive Compounds, Friedrich-Alexander-Universität Erlangen-Nürnberg, Nikolaus-Fiebiger-Str. 10, Erlangen, Germany; 7 Rudolf Buchheim Institute of Pharmacology, Justus Liebig University, Giessen, Germany; 8 Department of Pharmacology, Max Planck Institute for Heart and Lung Research, Bad Nauheim, Germany; 9 Department of Pharmacy, SciLifeLab Drug Discovery and Development, Uppsala University, Uppsala, Sweden; 10 Centre for Molecular Medicine, Medical Faculty, Goethe University, Frankfurt am Main, Germany

**Keywords:** Virtual drug screening, Structure-based drug design, X-ray crystallography, G protein-coupled receptors, Computational chemistry

## Abstract

Peptide-activated G protein-coupled receptors (GPCRs) play crucial roles in numerous diseases, but remain difficult therapeutic targets due to the challenges in developing small-molecule drugs. Here, we explore structure-based strategies to identify small-molecule agonists of neurotensin (NTS) receptors, which hold promise for developing non-opioid analgesics. Chemical libraries of drug-like molecules are first designed based on a receptor-peptide complex, and then 14.5 million compounds are computationally docked to the orthosteric binding site of the NTS_1_ receptor. A set of 39 top-ranked compounds is synthesized, and seven of these are experimentally confirmed to activate the NTS_1_ receptor. Structure-guided optimization yields NTS_1_ ligands with signaling signatures distinct from the endogenous peptide, and these compounds also exhibit high affinity for the NTS_2_ receptor. High-resolution crystal structures of two agonists bound to the NTS_1_ receptor confirm predicted binding modes and reveal key determinants of activation. In vivo, the compounds produce robust antinociception in rodents without inducing hypotension, consistent with a contribution of NTS_2_ receptor activity. To facilitate broader application of our virtual screening approach to peptide-binding GPCRs, we provide access to tailored chemical libraries containing billions of readily synthesizable compounds.

## Abstract (web summary)

In this work, small-molecule ligands of neurotensin receptors were identified using structure-based virtual screening, leading to the discovery of potent agonists with in vivo antinociceptive effects and to insights into the molecular basis of receptor activation.

## Introduction

G protein-coupled receptors (GPCRs) constitute the largest family of eukaryotic membrane proteins and transmit extracellular signals, such as hormones and neurotransmitters, into the cell. The >800 human GPCRs recognize a broad range of ligands, ranging from small molecules to peptides and proteins^1,2^, and have garnered considerable attention as therapeutic targets^3^. Although ~34% of all approved drugs target GPCRs^3^, only a quarter of therapeutically relevant receptors have been exploited, leaving ample opportunities for developing novel treatments^3,4^.

Among the human non-sensory GPCRs, more than 100 are modulated by peptides. These receptors have numerous physiological functions and are often key drug targets in oncology, endocrinology, and neurology^5–7^. Notable examples include opioid receptors, activated by analgesics, and angiotensin receptors, targeted by antihypertensive drugs. Despite the therapeutic relevance of peptide-binding GPCRs, many members within this group remain unexplored as drug targets due to difficulties associated with identifying small molecules or peptides with drug-like properties. Peptide ligands often suffer from poor metabolic stability and limited membrane permeability. In contrast, small molecules generally offer better oral bioavailability and may penetrate the blood-brain barrier more effectively^7,8^. However, the development of potent small-molecule drugs that mimic the functions of considerably larger peptide agonists remains a significant challenge.

G protein-coupled neurotensin receptors (NTSRs) are promising drug targets for pain management^9^, neurodegenerative and neuropsychiatric diseases^10,11^. The two receptor subtypes, NTS_1_R and NTS_2_R, are activated by the endogenous 13–amino-acid peptide agonist neurotensin (NTS) and share high sequence homology (64%)^12^. NTS_1_R is expressed both centrally and peripherally and couples to multiple G proteins, with G_q_ as the primary signaling pathway. In contrast, NTS_2_R is largely restricted to the central nervous system (CNS), and the signaling profile of this subtype remains poorly understood^12,13^. Both NTS_1_R and NTS_2_R are involved in the antinociceptive effects of NTS^9,14,15^, and drugs targeting these receptors could provide a safer alternative to opioid analgesics, which are associated with serious side effects such as addiction, respiratory depression, and tolerance^16–18^. Although NTS and its derivatives are potent agonists, these peptides generally have poor pharmacokinetic properties and a limited ability to cross the blood-brain barrier. Despite extensive academic and industrial efforts, only a few drug-like ligands for NTSRs have been identified over the past three decades^19–24^. Furthermore, no small-molecule NTSR agonist has yet advanced to clinical trials^25^. While no high-resolution structures of NTS_2_R are available, studies of NTS_1_R using crystallography and cryo-EM provided detailed insights into the molecular mechanisms underlying ligand recognition and receptor activation^24,26–33^.

In this work, we explore whether structure-based design can accelerate the discovery of small-molecule ligands for peptide-activated GPCRs and develop an approach for generating tailored chemical libraries for this target class. Millions of compounds are computationally docked into the orthosteric binding site of NTS_1_R^24,26,27^, and top-ranked compounds are synthesized and evaluated experimentally. We identify diverse agonist scaffolds, which are further optimized, and the three most potent agonists are extensively evaluated in signaling assays. Crystal structures of NTS_1_R in complex with two agonists are determined, providing insights into the molecular basis of ligand binding and receptor activation. The discovered ligands also show high affinity and selectivity for the NTS_2_R subtype and exhibit analgesic effects in a rodent pain model. Given the rapidly increasing number of GPCR-peptide complex structures, we believe our approach may be applicable to a large number of drug targets. To further expedite ligand discovery efforts for peptide-binding receptors, we make our tailored chemical libraries publicly accessible.

## **Results**

### Design of chemical libraries tailored for neurotensin receptors

**Fig. 1:**
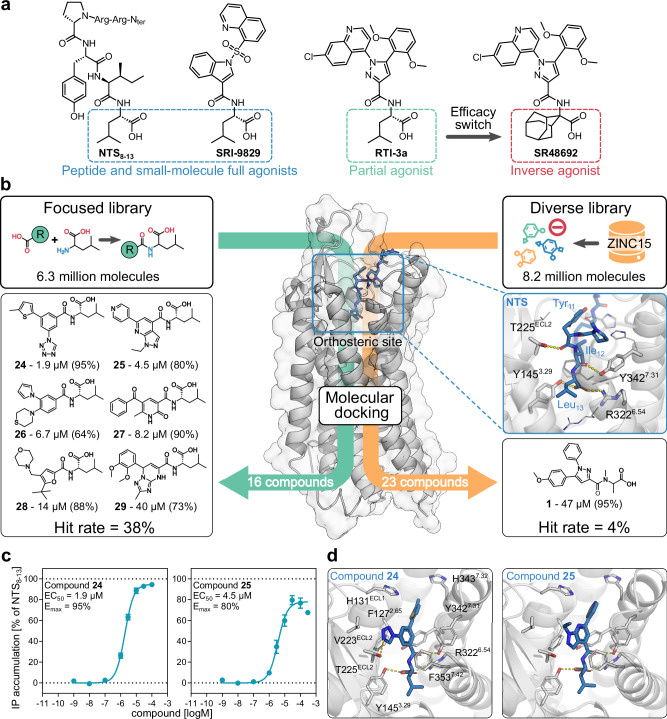
Identification of NTS_1_R agonists by structure-based virtual screening. **a** Chemical structures of full agonists NTS_8–13_ and **SRI-9829**, partial agonist **RTI-3a**, and inverse agonist **SR48692**. The C-terminal chemical groups anchoring the compounds in the NTS_1_R binding site are indicated by the dotted squares. Modification of the C-terminal leucine of **RTI-3a** alters ligand efficacy from partial to inverse agonist. **b** Molecular docking of two virtual libraries to the orthosteric site of hNTS_1_R. The receptor is shown as a gray cartoon with side chains of the binding site and NTS_8–13_ in sticks. NTS_8–13_ is shown as blue sticks, and hydrogen bonds are depicted as yellow dashed lines. Residue numbers correspond to the hNTS_1_R. Chemical structures of screening hits from the focused and diverse library are shown in the left and right panels, respectively. EC_50_ and E_max_ values were determined in inositol-monophosphate (IP_1_ or IP) accumulation assays relative to NTS_1_R activation by NTS_8–13_. **c** Concentration-response curves of compounds **24** and **25** in the IP_1_ accumulation assays relative to NTS_1_R activation by NTS_8–13_. Data indicate mean ± SEM of *n* = 7 (**24**) and *n* = 9 (**25**) independent experiments. **d** Predicted binding modes of the two most potent hits, compounds **24** and **25**. The hNTS_1_R is shown as a gray cartoon with ligands and side chains of the binding site in sticks. Receptor-ligand hydrogen bonds are shown as yellow dashed lines. Residue numbers correspond to the hNTS_1_R. Source data are provided as a Source Data file.

We analyzed crystal structures of rat NTS_1_R (rNTS_1_R) bound to the six C-terminal residues of NTS (NTS_8–13_, Arg_8_–Arg_9_–Pro_10_–Tyr_11_–Ile_12_–Leu_13_) to identify critical interactions for receptor activation^24,34^. The anionic C-terminal carboxylate (Leu_13_) of the potent agonist NTS_8–13_ is the most deeply buried residue in the binding site, and a network of interactions involving a salt bridge with R327^6.54^ and a hydrogen bond with Y146^3.29^ links the agonist to conserved microswitches involved in activation (superscripts represent Ballesteros−Weinstein numbering^35^)^24^. The importance of these interactions is supported by complex structures with the small-molecule agonists **RTI-3a** and **SRI-9829**, which are also anchored by a C-terminal leucine in the binding site (Fig. 1a-b) and mimic the interactions of the last three or four residues of NTS_8–13_, respectively^24^. Furthermore, chemical modifications to the C-terminal leucine side chain of small-molecule agonists can transform an agonist into an inverse agonist, underscoring its role in receptor activation (Fig. 1a)^20,22^. As 75% of the binding site residues interacting with Tyr_11_–Ile_12_–Leu_13_ are conserved between NTS_1_R and NTS_2_R, and **RTI-3a** binds to both subtypes, compounds designed based on the NTS_1_R structure were also expected to be relevant for NTS_2_R.

Two virtual chemical libraries were designed based on the analysis of the receptor-peptide complexes (Fig. 1b). Given the importance of the C-terminal carboxylate group, we first identified commercially available anionic compounds in the ZINC database^36^. The resulting diverse library contained 8.2 million compounds of a size suitable for interacting with the pockets occupied by the C-terminal residues of NTS_8–13_. However, only 0.2% of these compounds contained a C-terminal leucine, a key feature in NTS_1_R activation. To improve coverage of the relevant regions of chemical space, a second virtual library was generated by connecting a C-terminal leucine to commercial building blocks using an amide coupling reaction. This focused library contained 6.3 million compounds accessible via in-house synthesis and included larger molecules to reach a database size comparable to the diverse library. The vast majority of the compounds in the diverse and focused libraries represented novel chemical structures that, to the best of our knowledge, had never been synthesized previously.

### Structure-based docking for NTS_1_R agonists

The molecular docking screens were performed using the crystal structure of a stabilized form of rNTS_1_R (NTSR1–H4_x_) in complex with the small-molecule partial agonist **RTI-3a** (PDB accession code: 6ZA8)^24^. After modifying the structure to match the human NTS_1_R (hNTS_1_R) sequence and optimizing docking parameters^37^, we screened the 14.5 million compounds from the two virtual libraries against the orthosteric binding site using the program DOCK3.7^38^. The top-scoring 10% of each compound library was then clustered based on topological similarity to identify diverse candidate molecules. The 1000 top-ranking clusters from each library were visually inspected, and 23 and 16 compounds were selected from the diverse and focused library, respectively (Supplementary Tables S1-2). Priority was given to compounds forming the same polar interactions as the C-terminal leucine of NTS_8–13_ but featuring diverse chemical groups in the subpockets occupied by the peptide and small-molecule agonists. We also considered contributions to ligand binding that are not accurately captured by the docking scoring function, following standard virtual screening practices. Compounds were deprioritized based on several commonly used criteria, including ligand strain, unsatisfied polar atoms in the ligand or binding site, and unlikely tautomeric or ionization states^37,39^. Finally, the 39 compounds were either ordered from commercial make-on-demand libraries (diverse library) or synthesized in-house (focused library; the detailed synthetic procedures are described in the Supplementary Methods).

### Functional screening of designed compounds

We first evaluated the 39 selected compounds for their G_q_-promoted activity at hNTS_1_R by measuring inositol-monophosphate (IP_1_ or IP) accumulation at three concentrations (10, 30 and 100 μM). The EC_50_ and E_max_ values were determined for the compounds exhibiting a significant and concentration-dependent response (Supplementary Figs. S1-2). Among the 23 compounds from the diverse library, only compound **1** was active and showed weak potency (EC_50_ = 47 µM, E_max_ = 95%; Fig. 1b). Interestingly, six active compounds (**24–29**) from the focused library were identified with EC_50_ values ranging from 1.9 to 40 µM, reflecting a substantially higher hit rate (Fig. 1b and Supplementary Fig. S2). Compounds **24** and **25** showed the highest potencies (EC_50_ = 1.9 and 4.5 μM, respectively) and exhibited high efficacy agonism at hNTS_1_R (E_max_ >80%, Fig. 1c). In contrast to the reference agonists, the leucine moiety in these two compounds is connected via an amide bond to a six-membered aromatic ring, which was predicted to occupy the same pocket as Ile_12_ in the NTS_8***–***13_ complex. In addition, substituents extended into the region occupied by Tyr_11_ and the pocket formed by the extracellular ends of the transmembrane helices 2 and 7 (TM2 and TM7) in the **RTI-3a** complex (Fig. 1d).

### Structure-guided optimization

**Fig. 2:**
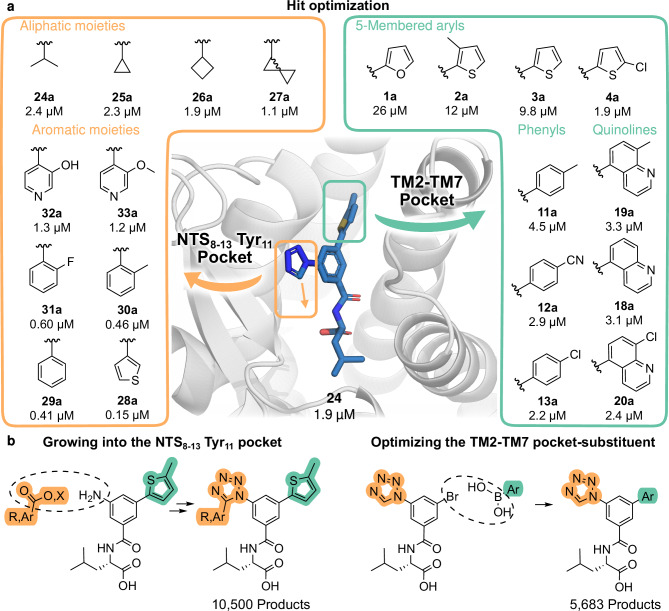
Structure-guided optimization of compound 24. **a** Experimentally evaluated compounds targeting the NTS_8–13_ Tyr_11_ pocket (orange box) and the TM2-TM7 pocket (green box) in hit optimization. NTS_1_R and designed ligands are shown as a gray cartoon and sticks, respectively. Mean EC_50_ values were determined in IP_1_ accumulation assays relative to NTS_1_R activation by NTS_8–13_ from *n *= 2 (**1a**, **3a**, **18a**, **19a**, **24a**, **25a,** **26a**, **27a**, **32a**, **33a**); *n *= 3 (**2a**, **11a**, **12a**, **13a**); *n* = 4 (**4a**, **20a**); *n* = 5 (**31a**); *n* = 6 (**29a**); and *n* = 15 (**28a**, **30a**) independent experiments. **b** Design and synthesis of chemical libraries. Source data are provided as a Source Data file.

Compared to the full agonist **SRI-9829** (EC_50_ = 0.33 µM, E_max_ = 104%) and the partial agonist **RTI-3a** (EC_50_ = 0.25 µM, E_max_ = 59%), compounds **24** and **25** are approximately 7- and 16-fold less potent in inducing IP_1_ generation, respectively. To further optimize these ligands, we carried out additional rounds of virtual library generation, molecular docking, compound synthesis, and IP_1_ accumulation assays. In total, 35 and 60 analogs of compounds **24** and **25**, respectively, were synthesized. Optimization of compound **24** (scaffold **a**: compounds **1a–35a**, Fig. 2 and Supplementary Table S3) resulted in compounds with improved potencies, whereas the analogs of compound **25** (scaffold **b**: compounds **1b–60b**, Supplementary Fig. S3 and Table S4) exhibited comparable or lower activity. The detailed synthetic procedures are described in the Supplementary Methods.

Aiming to optimize compound **24**, we first explored the possibility of replacing the 5-methylthiophen-2-yl group at position 3 (Fig. 2a and Supplementary Table S3). The structure of NTS_1_R bound to **RTI-3a** indicated that larger substituents could be accommodated by the TM2-TM7 pocket. Using in silico Suzuki coupling, we generated a virtual library of 5,683 unique molecules with drug-like properties (Fig. 2b). Based on docking calculations, we identified compounds that maintained the key interactions with Y145^3.29^ and R322^6.54^, and extended into the TM2-TM7 pocket, *e.g*., by forming van der Waals interactions with H343^7.32^ and Y346^7.35^. The selected substituents were based on either five- or six-membered aromatic rings that varied in size, polarity, and shape. A set of 21 compounds was synthesized, and several of these activated hNTS_1_R with potencies comparable to compound **24**. Importantly, the presence and position of the methyl group on the thiophene ring of **24** were important for activity. As no substantial improvement in potency was observed among the first set of analogs, we maintained the 5-methylthiophen-2-yl group and instead focused on optimizing position 5 of the tetrazole moiety in compound **24**. In this case, we identified compounds extending into the pockets occupied by Tyr_11_ of NTS_8–13_ in the NTS_1_R-peptide complex, as the Tyr_11_ side chain is essential for high-affinity binding (Fig. 2b)^40^. From a virtual library of 10,500 compounds based on tetrazole condensation, we selected 12 diverse substituents predicted to interact with residues in the extracellular loops, *e.g*., H131^ECL1^, V223^ECL2^, and T225^ECL2^. Of the seven analogs demonstrating improved potency, **28a** (EC_50_ = 0.15 μM, E_max_ = 101%), **29a** (EC_50_ = 0.41 μM, E_max_ = 101%), and **30a** (EC_50_ = 0.46 μM, E_max_ = 98%) were the most promising (Fig. 2a). The aromatic substituents of these compounds were all predicted to overlap with the position of the Tyr_11_ phenol ring in the NTS_8***–***13_ complex. Additional controls for **28a** and **30a** confirmed the orthosteric engagement of these ligands and that hNTS_1_R mediates their agonistic activity (Supplementary Fig. S4a-c). The identified agonists displayed potencies and efficacies comparable or superior to those of the most potent small-molecule agonists described to date, such as **SRI-9829** (EC_50_ = 0.33 µM, E_max_ = 104%). Compounds **28a** and **30a** both comply with Lipinski’s rule of five^41^ and are, apart from the leucine moiety, structurally dissimilar to the reference agonists **SRI-9829** and **RTI-3a** (Supplementary Table S5).

**Fig. 3:**
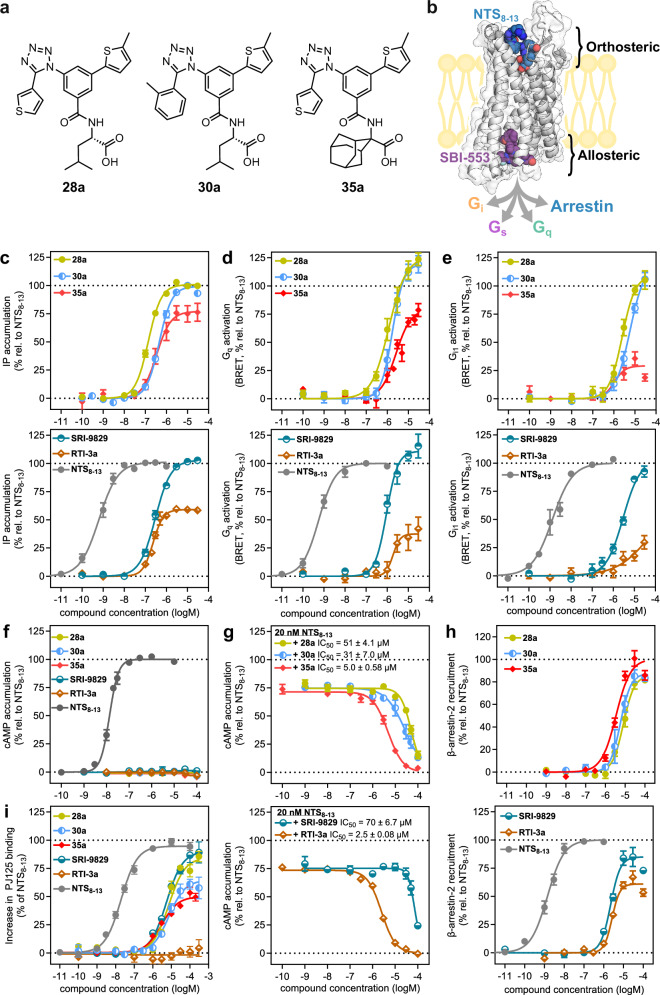
In vitro biological activity of compounds 28a, 30a, and 35a. **a** Chemical structures of compounds **28a**, **30a**, and **35a**. **b** hNTS_1_R signaling and interactions with orthosteric and allosteric ligands (PDB accession code: 8FN0). **c** IP_1_ accumulation assays showed high efficacy for NTS_1_R activation, *n* = 15 (**28a**, **30a**); 8 (**35a**); 13 (**SRI-9829**, NTS_8-13_); 17 (**RTI-3a**); while BRET-based sensing of the activation of (**d**) G_q_ proteins and (**e**) G_i_ proteins reveals reduced potency and, in particular for compound **35a**, reduced efficacy (*n* = 4–8, see Table 1). **f** The small molecules do not induce cAMP accumulation and are thus unable to activate G_s_ proteins, *n* = 5 (**SRI-9829**); 6 (NTS_8-13_); 3 (other compounds). **g** The small molecules inhibit the effect of 20 nM NTS_8–13_, thus acting as functional antagonists for NTS_1_R G_s_-coupling (*n* = 3–5, see Table 1). **h** Among the three identified agonists, compound **35a** is the most potent small-molecule agonist for the recruitment of β-arrestin-2 in the presence of GRK2 (*n* = 3–6, see Table 1). **i** NanoBRET-based binding assay monitoring the agonist-enhanced binding of the fluorescent probe **PJ125** (derived from **SBI-553**), which recognizes an intracellular allosteric pocket of NTS_1_R, *n* = 10 (NTS_8-13_); 6 (**35a**); 5 (other compounds). **c–i** Data show mean ± SEM of *n* = 3–17 independent experiments, each performed in duplicate. Source data are provided as a Source Data file.

In order to assess the role of the leucine moiety in receptor activation, we also replaced its side chain with a bulkier adamantyl group. Previous work has shown that this substitution can result in a compound with inverse agonist activity at NTS_1_R (Fig. 1a)^20,22^. This is consistent with the idea that antagonists and inverse agonists expand the orthosteric binding site, leading to TM helix orientations less compatible with G protein coupling^42^. Interestingly, introducing an adamantyl moiety into compound **28a** to give **35a** yielded an agonist with only slightly reduced efficacy (E_max_ = 81%) and potency (EC_50_ = 0.44 µM) (Fig. 3a).

### Signaling signatures of small-molecule agonists

To assess the pharmacological profiles of the identified ligands, we evaluated the ability of three compounds (**28a**, **30a**, and **35a**; Fig. 3a) and reference ligands **RTI-3a** and **SRI-9829** to activate NTS_1_R (Table 1). NTS_1_R is a relatively promiscuous GPCR that primarily couples to the G_q_ protein. The receptor also activates the G_s_ and G_i/o_ protein families and thereby modulates the activity of adenylyl cyclase. Moreover, NTS_1_R tightly interacts with β-arrestins that mediate receptor desensitization, play an important role in receptor internalization, and scaffold signaling processes (Fig. 3b)^13^.

**Table 1:** Activities of compounds for G protein activation and β-arrestin recruitment by hNTS_1_R in HEK293 cells

	G_q_^a^		G_i_^b^		cAMP^c^	β-arrestin-2^d^	
	EC_50_ [µM]^e^	E_max_ [%]^f^	EC_50_ [µM]^e^	E_max_ [%]^f^	IC_50_ [µM]^g^	EC_50_ [µM]^d^	E_max_ [%]^e^
**28a**	1.1 ± 0.17	131 ± 7 (*7*)	2.6 ± 0.42	104 ± 8 (*4*)	51 ± 4.1 (*5*)	9.7 ± 3.2	89 ± 7 (*4*)
**30a**	1.8 ± 0.28	126 ± 6 (*7*)	6.0 ± 1.0	121 ± 7 (*4*)	31 ± 7.0 (*5*)	6.8 ± 1.2	90 ± 4 (*4*)
**35a**	3.2 ± 0.65	86 ± 5 (*8*)	2.1 ± 0.52	31 ± 5 (*6*)	5.0 ± 0.58 (*5*)	3.1 ± 0.18	93 ± 5 (*4*)
**SRI-9829**	0.98 ± 0.13	112 ± 8 (*4*)	3.0 ± 0.52	107 ± 2 (*4*)	70 ± 6.7 (*3*)	2.2 ± 0.32	86 ± 9 (*3*)
**RTI-3a**	1.7 ± 0.45	47 ± 6 (*6*)	n.c. ^h^	30 ± 4 @30 µM (*4*)	2.5 ± 0.08 (*4*)	2.7 ± 0.22	61 ± 4 (*3*)
**NTS**_**8–13**_	0.00063 ± 0.00009	100 (*6*)	0.0016 ± 0.00048	100 (*8*)	EC_50_: 0.012 ± 0.0012 (6)^j^	0.0016 ± 0.00034	100 (*6*)

^a^ G_q_ activation assessed by BRET between Gα_q_-RlucII and Gβ_1_Gγ_2_-GFP10.

^b^ G_i_ activation assessed by BRET between Gα_i1_-RlucII and Gβ_1_Gγ_2_-GFP10.

^c^ Inhibition of cAMP accumulation by 20 nM NTS_8–13_ measured with the cAMP GloSensor.

^d^ β-arrestin-2 recruitment assessed with the PathHunter assay with hNTS_1_R-PK in the presence of GRK2.

^e^ Mean potency ± SEM.

^f^ Mean efficacy ± SEM relative to the maximum effect of NTS_8–13_ and number (*n*) of individual experiments.

^g^ Inhibitory potency of the ligands and number (*n*) of individual experiments.

^h^ n.c., no full concentration-response curve was observed, and potency was therefore not calculated.

^j^ Potency [µM] derived from assays in agonist mode.

Consistent with our primary screen, the G_q_-biosensor assays confirmed full agonist properties for **28a** and **30a**, with slightly lower potencies. Interestingly, these small-molecule agonists showed superior efficacy (E_max_ = 131% and 126%, respectively) compared with NTS_8–13_ (Fig. 3d and Table 1). The relative efficacy of the partial agonist **35a** was slightly lower (E_max_ = 86%), but superior compared to the reference ligand **RTI-3a** (E_max_ = 47%). Compounds **28a** and **30a** were also full agonists in the G_i1_ activation assay (EC_50_ = 2.6 and 6.0 μM, E_max_ = 104% and 121%), similar to the profile of **SRI-9829** (EC_50_ = 3.0 μM, E_max_ = 107%), while the adamantane derivative **35a** showed only weak partial agonism in this assay (EC_50_ = 2.1 μM, E_max_ = 31%; Fig. 3d-e and Table 1). Interestingly, neither **28a**, **30a**, and **35a** nor **SRI-9829** or **RTI-3a** induced substantial accumulation of cAMP in NTS_1_R-expressing HEK293T cells (Fig. 3f and Table 1). In contrast, compounds **35a** and **RTI-3a** fully inhibited the effect of 20 nM NTS_8–13_ on the intracellular cAMP concentration. Thus, while acting as G_q_ agonists, these ligands showed functional antagonism for the coupling of NTS_1_R to the G_s_ protein, with potencies (IC_50_) of 5.0 µM and 2.5 µM, respectively (Fig. 3g and Table 1). For **28a**, **30a**, and **SRI-9829**, inhibition of the NTS_8–13_ effect was only observed at higher ligand concentrations (IC_50_ > 30 µM).

In the PathHunter β-arrestin-2 recruitment assay^43^, **28a**, **30a**, **35a**, and **SRI-9829** showed very similar efficacies in the presence (E_max_ = 89–93%, Fig. 3h and Table 1) and in the absence (E_max_ = 98–107%, Supplementary Fig. S4d) of overexpressed GRK2, while **RTI-3a** was less effective (E_max_ = 61% and 69%, respectively). Although the overexpression of GRK2 did not have a substantial impact on efficacy, ligand potency increased 1.7- to 3.3-fold for the small-molecule agonists upon co-transfection of this G_βγ_-dependent kinase^44^. For NTS_8–13_, potency did not increase upon GRK2 co-expression (EC_50-GRK2_ = 1.1 nM, EC_50+GRK2_ = 1.6 nM), suggesting that the peptide agonist does not require enhanced kinase expression to induce efficient β-arrestin recruitment. Additional BRET-based experiments measuring the recruitment of β-arrestin-mVenus to a hNTS_1_R-Rluc8 fusion protein confirmed the strong agonist properties of **28a**, **30a**, and also **35a**, which showed the highest potency (EC_50_ = 1.6 µM, E_max_ = 89%) among the identified ligands (Supplementary Fig. S4e).

Overall, the peptide NTS_8–13_ potently activates G_q_, G_i_, and G_s_ proteins and recruits β-arrestin-2 (EC_50_ = 0.63–12 nM), while our small-molecule agonists **28a**,** 30a**, and **35a** display micromolar potencies with a distinct signaling signature. These compounds lead to the activation of G_q_ and G_i_ proteins and promote β-arrestin-2 recruitment, but inhibit signaling via G_s_ proteins.

### Agonist-dependent binding of an allosteric modulator to NTS_1_R

Previous drug discovery efforts identified the allosteric modulator **SBI-553**, which binds to an intracellular site of NTS_1_R (Fig. 3b) and biases the receptor toward β-arrestin recruitment^28,45^. The magnitude and direction of the allosteric modulation can vary depending on the orthosteric agonist, potentially providing an additional avenue for developing therapeutics^46^. To explore the potential to leverage such probe dependence in drug discovery targeting NTS_1_R, we conducted NanoBRET-based binding experiments using an hNTS_1_R-Nanoluc fusion protein and a fluorescent allosteric ligand derived from **SBI-553** (**PJ125**, compound **14** in ref. ^47^, Fig. 3b). Compounds **28a**, **30a**,** 35a**, and **SRI-9829** enhanced the binding of **PJ125** (Fig. 3i and Table 2). While the efficacy of **SRI-9829** and **28a** was close to that of NTS_8–13_ (E_max_ = 82–89%), enhancement of allosteric binding by compounds **30a** and **35a** was slightly weaker (E_max_ = 50–63%). All four small molecules exhibited similar EC_50_ values (2.7–7.4 µM, Table 2), which are in the same range as their potencies determined in the different NTS_1_R G protein activation and β-arrestin recruitment assays (Table 1). Interestingly, the reference ligand **RTI-3a** did not substantially increase **PJ125** binding, suggesting that its relatively weak partial agonism is not sufficient to exhibit positive cooperativity with the allosteric probe.

**Table 2:** Affinities of designed compounds and reference ligands for the orthosteric binding pocket of the human NTS_1_R and NTS_2_R, and effects on the binding of the allosteric fluorescent NTS_1_R ligand **PJ125**

	[^3^H]UR-MK300^a^					PJ125 binding^b^		
	NTS_1_R		NTS_2_R		NTS_2_R selectivity^c^	NTS_1_R-Nluc		
	*K*_i_ (µM) ± SEM	*n*^d^	*K*_i_ (µM) ± SEM	n^d^	*K*_i_(NTS_1_R)/*K*_i_(NTS_2_R)	EC_50_ (µM) ± SEM^e^	E_max_ (%) ± SEM^f^	*n*^g^
**28a**	29 ± 5.7	8	0.680 ± 0.14	5	43	7.2 ± 1.1	82 ±6	5
**30a**	55 ± 11	5	0.690 ± 0.13	10	80	7.4 ± 0.94	63 ± 4	5
**35a**	7.9 ± 1.7	6	0.022 ± 0.0021	5	360	2.7 ± 0.41	50 ± 5	6
**SRI-9829**	50 ± 10	5	0.170 ± 0.037	6	294	5.6 ± 0.88	89 ± 8	5
**RTI-3a**	0.80 ± 0.17	6	2.0 ± 0.38	8	0.4	n.c.^h^	<5	5
**NTS**_**8–13**_	0.00036 ± 0.000052	8	0.00041 ± 0.000076	9	0.88	0.023 ± 0.0044	100	10
**SR142948A**	0.00063 ± 0.000096	6	0.031 ± 7.0	6	0.020	---	---	0

^a^ Binding affinity determined by radioligand displacement using membranes from HEK293T cells expressing the respective receptor.

^b^ Increase in **PJ125** binding to the intracellular allosteric pocket of hNTS_1_R determined by NanoBRET with 0.68 µM **PJ125** and membranes from HEK293T cells expressing hNTS_1_R-Nluc.

^c^ Calculated with the mean *K*_i_.

^d^ Number of individual experiments, each performed in triplicate.

^e^ Increase of fluorescent probe binding relative to the effect of NTS_8–13_.

^f^ Potency derived from three-parameter non-linear regression.

^g^ Number of individual experiments, each performed in duplicate.

^h^ Not calculated (n.c.).

### Complex structures of NTS_1_R with compounds 28a and 30a confirm the predicted agonist binding modes

**Fig. 4:**
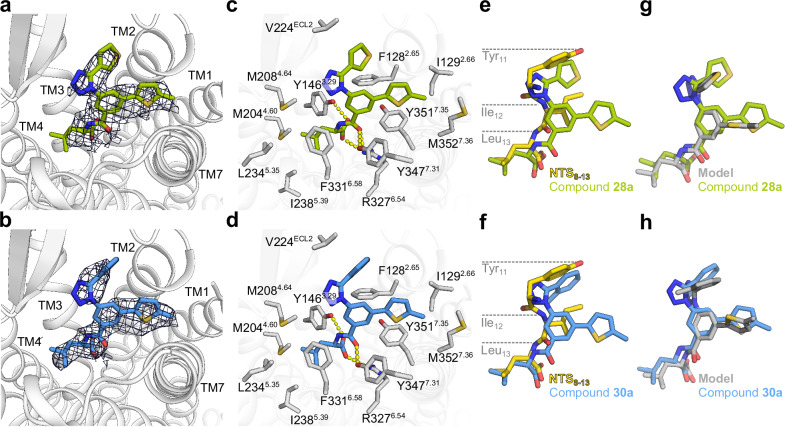
NTSR1-H4_x_ complex structures with small-molecule agonists 28a and 30a. **a**, **b** NTSR1-H4_x_ in complex with **28a** (**a**) and **30a** (**b**). Chicken wires show the electron density of the 2F_o_–F_c_ map contoured at 1σ before inclusion of the agonists. Refined agonists are depicted in green and blue, respectively. **c**, **d** Interactions of NTSR1-H4_x_ with **28a** (**c**) and **30a** (**d**). Hydrogen bonds and salt bridges are shown as yellow dots. **a**–**d** display identical orientations. **e**, **f** Superpositions of conformer A of **28a** (**e**) and **30a** (**f**) onto the three C-terminal residues (NTS_11–13_) of the endogenous agonist (yellow). **g**, **h** Comparison of computational models (gray) and experimentally determined binding modes of conformer A of **28a** (**g**) and **30a** (**h**) (in green and blue, respectively).

To gain atomic insight into the interactions of the promising small-molecule agonists **28a** and **30a** with NTS_1_R, we performed co-crystallization with NTSR1–H4_X_^24^, a construct derived from rNTS_1_R. NTSR1–H4 was stabilized in the presence of NTS_8–13_^48^, and is therefore likely conformationally biased in the extracellular part toward the agonist-bound, active state. This is corroborated by the ~12- and 1700-fold higher affinities of NTSR1–H4_X_ for NTS_8–13_ and **SRI-9829**, respectively, compared to wild-type rNTS_1_R, whereas the affinities for inverse agonists are reduced by 20–40-fold (Supplementary Table S6)^24^. In agreement with these observations, compounds **28a** and **30a** show strong conformational selectivity for NTSR1–H4_X_ (>100-fold) compared to the wild-type receptor, with affinities of 167 and 41 nM, respectively (Supplementary Table S6). In the presence of compound **28a** or **30a**, crystals obtained in lipidic cubic phase diffracted to 3.1 Å resolution. After refinement of the polypeptide chain of NTSR1–H4_X_, the 2F_o_–F_c_ maps confirmed the bound orientation and conformation of compounds **28a** (Fig. 4a) and **30a** (Fig. 4b). For data collection and refinement statistics, see Supplementary Table S7.

The fully refined complexes with compounds **28a** and **30a** revealed the interactions of the agonists with the receptor (Fig. 4c-d). Compounds **28a** and **30a** bind to NTS_1_R in a manner similar to the C-terminal tripeptide Tyr_11_–Ile_12_–Leu_13_ of NTS_8–13_ (Fig. 4e-f). In particular, the position, orientation, and interactions of the leucine moiety for both small-molecule complexes are virtually identical to the corresponding residue in the NTS_8–13_ complex (PDB accession code: 6YVR^24^). In the adjacent site, the phenyl ring of compounds **28a** and **30a** occupies a region filled by the side chain of Ile_12_ in the NTS_8–13_ complex (Fig. 4e-f), forming extensive hydrophobic interactions with F128^2.65^, F331^6.58^, Y347^7.31^, and Y351^7.35^. In the meta position of the phenyl ring, the two small-molecule ligands feature a methylthiophene substituent surrounded by residues from the extracellular ends of TM2 and TM7, an area that is unoccupied in the NTS_8–13_ complex. The methyl group of the thiophene maintains van der Waals interactions with the methyl groups of I129^2.66^ and M352^7.36^, while its sulfur is positioned approximately 3.5 Å above the aromatic ring of Y351^7.35^. The tetrazole ring in the alternative meta position of the phenyl ring of the agonists essentially accounts for the peptide bond between Ile_12_ and Tyr_11_ as well as the CH_2_ group of Tyr_11_ in the NTS_8–13_ complex. In compound **30a**, a toluene in the ortho position of the tetrazole ring occupies a region filled by the aromatic ring of Tyr_11_ (Fig. 4f) and interacts with F128^2.65^ and V224^ECL2^. In contrast, for compound **28a**, the hammerhead-shaped electron density in the 2F_o_–F_c_ map observed before ligand inclusion suggests the presence of two distinct conformations for the tetrazole-thiophene moiety (Supplementary Fig. S5a). Hence, the thiophene substituent may not only adopt an orientation comparable to that of the toluene moiety in compound **30a** (Fig. 4e-f), but also a conformation similar to the one of Pro_10_ in the NTS_8–13_ complex (Supplementary Fig. S5b-c).

Comparison of the computationally predicted binding modes of compounds **28a** and **30a** with the experimentally determined complexes showed that the interactions of the core ligand scaffold—comprising the leucine, phenyl ring, and the amide linking these groups—were nearly identical. The regions occupied by the methylthiophene and aryl tetrazole substituents were also correctly identified. However, the experimental structures also revealed weaknesses in the docking models. The specific interactions formed by the methylthiophene and aryl tetrazole, as well as the shapes of the subpockets accommodating these substituents, differed between the predicted and experimental structures. In addition, the torsional angle between the phenyl and thiophene rings adopted a strained conformation in the models. These discrepancies led to ligand root-mean-square deviations (RMSDs) from the crystal structures of 2.4 and 2.6 Å for compounds **28a** and **30a**, respectively (Fig. 4g-h).

### Molecular basis of full and partial agonism

The **RTI-3a** complex^24^ formed the basis for designing the small-molecule agonists **28a** and **30a**. In contrast to the partial agonist **RTI-3a**, the two compounds **28a** and **30a** are full agonists of both rat and human NTS_1_R for G_q_ activation (Fig. 3c and Supplementary Fig. S6). This raises the question of which molecular determinants are responsible for the differences in the pharmacological properties of these agonists.

**Fig. 5:**
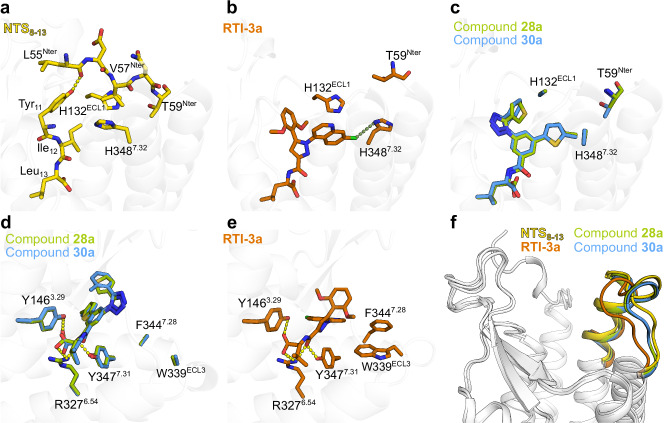
Molecular determinants for binding of NTS_8–13_, 28a, 30a, and RTI-3a. **a**–**c** Orientation and flexibility of H132 and H348 in the NTS_8–13_ (**a**), the **RTI-3a** (**b**), as well as the compound **28a** and **30a** (**c**) complexes. **d**, **e** Orientation and flexibility of W339 and F344 in the compound **28a** and **30a** (**d**), as well as the **RTI-3a** (**e**) complexes. Hydrogen bonds and the Cl–π interaction are shown as yellow and green dots, respectively. **f** Comparison of the backbone of residues 333–350 in the **RTI-3a** (orange), NTS_8–13_ (yellow), **28a** (green), and **30a** (blue) complexes. PDB accession codes for the NTS_8–13_ and **RTI-3a** complexes are 6YVR and 6ZA8, respectively.

In the small-molecule complexes, the methylthiophene substituent of compounds **28a** and **30a**, as well as the chloroquinoline substituent of **RTI-3a**, extend into a pocket partially blocked by the side chains of V57^Nter^ and H348^7.32^ in the NTS_8–13_ complex (Fig. 5a). In particular, in the **RTI-3a** complex, the side chain of H348^7.32^ is rotated by approximately 180˚ and stabilized by a strong Cl-π interaction between its N^δ1^ and the chloro substituent of the quinoline moiety (Fig. 5b)^24,49^. In contrast, in the compound **28a** and **30a** complexes, the orientation of the H348^7.32^ side chain is largely undefined, indicating increased flexibility in this receptor region (Fig. 5c). Furthermore, the methylthiophene substituent in the complexes of compounds **28a** and **30a** is rotated by approximately 60˚ relative to the bulky chloroquinoline moiety in the **RTI-3a** complex. Consequently, the orientation of the H132^ECL1^ side chain is poorly defined in the compound **28a** and **30a** complexes, while it maintains hydrophobic interactions with the chloroquinoline group in the **RTI-3a** complex. Finally, it should be noted that the two small-molecule agonists lack interactions mimicking Arg_8_–Arg_9_–Pro_10_, which are important for NTS binding to NTS_1_R (Supplementary Fig. S7)^40^. These differences in receptor-ligand interactions likely contribute to the lower affinities of compounds **28a** and **30a** compared to **RTI-3a** and NTS_8–13_ (Supplementary Table S6).

Comparisons of the structures also provided insights into the molecular basis of the different ligand efficacy profiles. In the complexes of compounds **28a** and **30a**, not only the orientation of the side chains of H132^ECL1^ and H348^7.32^, but also those of W339^ECL3^ and F344^7.28^ are disordered (Fig. 5d). This contrasts with the **RTI-3a** complex, where the bulky chloroquinoline group allows for hydrophobic interactions with the side chains of W339^ECL3^ and F344^7.28^ (Fig. 5e). While the stabilization of these side chains may be energetically favorable, it results in a 1–3 Å shift of the backbone of residues 333–350 relative to the NTS_8–13_ complex (Fig. 5f). In particular, the chloroquinoline group induces an outward shift at the beginning of TM7 (residues 342–348) compared to the complexes of compounds **28a**, **30a**, and NTS_8–13_. As the backbone of residues 333–350 in the compound **28a** and **30a** complexes aligns much more closely with the NTS_8–13_ complex, this difference may be correlated with the increased efficacy of compounds **28a** and **30a** compared to **RTI-3a** and related to the inclination of TM6 and TM7 responsible for access of the C-terminal helix of Gα.

### Identified ligands show high affinity and selectivity for the NTS_2_R subtype

Competitive binding studies were performed to determine whether compounds **28a**, **30a**, and **35a** act as orthosteric agonists and to characterize their selectivity profiles (Table 2). In agreement with orthosteric binding, the three ligands displaced the labeled NTS_8–13_ derivative [^3^H]UR-MK300^50^ from the hNTS_1_R orthosteric site at high concentrations. The calculated affinities (*K*_i_ = 29 µM for **28a**, *K*_i_ = 55 µM for **30a**, and *K*_i_ = 7.9 µM for **35a**) were comparable to that of the reference full agonist **SRI-9829** (*K*_i_ = 50 µM) but weaker than that of the partial agonist **RTI-3a** (*K*_i_ = 0.80 µM). In contrast, the binding affinities for NTS_8–13_ and the reference antagonist **SR142948A** were up to 150,000-fold higher (0.36 nM and 0.63 nM, respectively). Notably, binding experiments with hNTS_2_R and rNTS_2_R revealed substantially higher affinities than those for hNTS_1_R (hNTS_2_R: *K*_i_ = 22–690 nM; rNTS_2_R K_i_ = 8–22 nM, Supplementary Table S8), thus demonstrating selectivity of compounds **28a**,** 30a**, and **35a** for this subtype. Among the tested small molecules, compound **35a** showed the highest affinity and selectivity for hNTS_2_R (360-fold, *K*_i_ = 22 nM). This further distinguishes the identified scaffold from the reference agonist **RTI-3a** and the antagonist **SR142948A** that exhibit a preference for the hNTS_1_R subtype. The reasons for the relatively low NTS_1_R affinities of the small-molecule agonists (Table 2), as well as for their high affinities for NTS_2_R (Table 2) and the stabilized NTS_1_R variant (Supplementary Table S6), are provided in the discussion. In comparison with the reference agents **SRI-9829**, **RTI-3a**, NTS_8-13_, and the NTS_2_R-selective peptide agonist **NT150**^51,52^, functional assays with digested murine left-ventricular heart tissue revealed that 1 µM of compounds **28a**, **30a**, and **35a** induce a significant formation of IP_1_ in wild-type but not NTS_2_R knockout (KO) mice, confirming that the ligands act as NTS_2_R agonists. NTS_2_R agonism of **35a** and **SRI-9829** was also statistically significant when tested at 100 nM (Supplementary Fig. S8). Finally, the selectivity of compounds **28a**, **30a**, and **35a** was further assessed through binding assays on six additional human GPCRs related to antinociception (µOR, κOR, δOR, 5-HT_1A_, α_2A_, and APJ receptors; Supplementary Table S9). All compounds were strongly selective for the hNTS_2_R subtype, and compound **35a** exhibited more than 600-fold higher affinity for hNTS_2_R than for all the other GPCRs evaluated.

### Analgesic activity of selective high-affinity NTS_2_R agonists

**Fig. 6:**
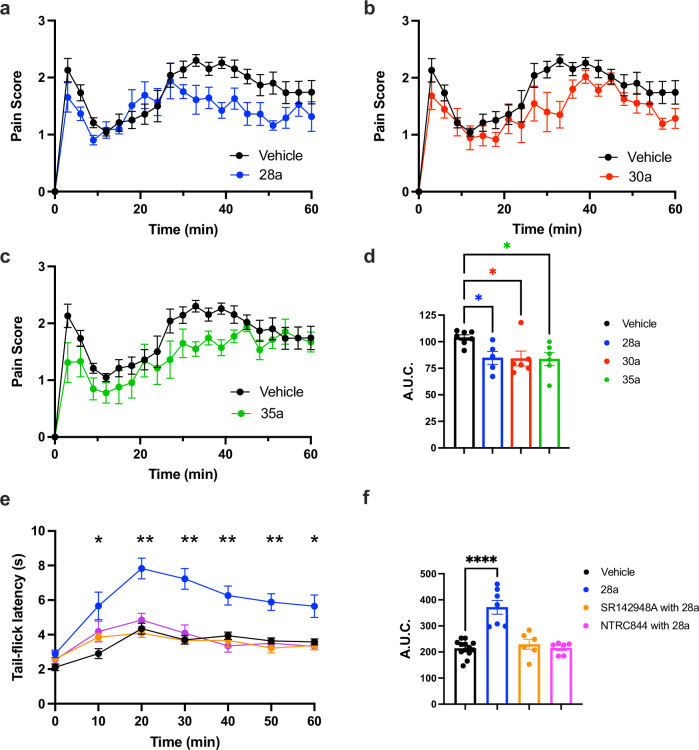
Analgesic effects of compounds 28a, 30a, and 35a. **a**–**c** Time course of formalin-evoked nocifensive behaviors following intrathecal administration of compounds **28a**, **30a**, and **35a** (1200 nmol/kg) in Sprague-Dawley rats. **d** Quantification of cumulative nociceptive response expressed as the area under the curve (AUC) of the formalin test (0–60 min post-injection). *P*-values: **28a** vs vehicle, *p* = 0.0495; **30a** vs vehicle, *p* = 0.0302; **35a** vs vehicle, *p* = 0.0261. **e**, **f** Antinociceptive effects of **28a** in the tail-flick thermal nociception assay, when administered alone or in combination with the non-selective neurotensin receptor antagonist **SR142948A** or the selective NTS_2_R antagonist **NTRC844** (5000 nmol/kg). Data are expressed as mean ± SEM. Pain score: *n* = 7 (vehicle); *n* = 5 (**28a**); *n* = 6 (**30a,** **35a**). Tail-flick latency: *n* = 7 (vehicle, **28a**); *n* = 6 (**SR142948A**, **NTRC844** with **28a**). Time-course data in the tail-flick assay were analyzed by two-way ANOVA followed by Dunnett’s multiple comparisons test. AUC data were analyzed using one-way ANOVA with Dunnett’s correction for multiple comparisons. * *p *< 0.05, ** *p *< 0.01, and **** *p *< 0.0001 versus vehicle. Source data are provided as a Source Data file.

NTS_1_R and NTS_2_R have emerged as promising targets for the development of non-opioid pain-relieving medications, with both subtypes contributing to the antinociceptive effects of the NTS peptide^9,14,15^. However, NTS_1_R activation is also associated with the regulation of cardiovascular function, thermoregulation, and gastrointestinal motility, which may limit its therapeutic utility^53–55^. In contrast, selective modulation of NTS_2_R is predicted to produce analgesia while minimizing adverse effects^40,56–58^. We evaluated the analgesic effectiveness of three high-affinity NTS_2_R ligands (**28a**, **30a**, and **35a**) in rats using the formalin persistent pain model^59^. When administered intrathecally at an equimolar dose (1200 nmol/kg), each compound significantly reduced formalin-induced nocifensive behaviors (licking, biting, shaking, and paw lifting) across the inflammatory phase (Fig. 6a-d). Compound **28a** was further evaluated in the tail-flick assay to assess its efficacy in an acute thermal nociception paradigm. Intrathecal administration of **28a** produced a robust, time-dependent increase in tail-flick latency, indicative of antinociceptive activity (Fig. 6e). To determine receptor subtype involvement, animals were pretreated with either the non-selective neurotensin receptor antagonist **SR142948A**^20,60^ or the selective NTS_2_R antagonist **NTRC844**^58,61^. Both antagonists fully abolished the analgesic effect of compound **28a** in the tail-flick test (Fig. 6e-f). These findings are consistent with the known expression of NTS_2_R on peripheral sensory neurons and within the spinal dorsal horn^62,63^. Finally, unlike NTS_8–13_, which induced a marked hypotensive response, intravenous administration of compounds **28a**, **30a**, and **35a** did not affect blood pressure, supporting a favorable safety profile for these NTS_2_R ligands (Supplementary Fig. S9). Together, these results are consistent with the hypothesis that the compounds elicit analgesia via modulation of NTS_2_R, although the potential involvement of NTS_1_R cannot be entirely excluded and warrants further investigation, *e.g*., using KO animals.

We further characterized the pharmacokinetic profile of compound **28a** in rats. Following intravenous administration of 5 mg/kg, total plasma concentrations declined from 98 µM at 5 min to 0.1 µM at 6 h, and were below the lower limit of quantification (LLOQ) at 24 h. The estimated plasma half-life, terminal volume of distribution, and clearance of compound **28a** were 2.8 h, 1.4 L/kg, and 90 mL/h, respectively (Supplementary Fig. S10). Cerebrospinal fluid (CSF) and brain tissue were collected between 2 and 24 h post-administration (Supplementary Table S10). Concentrations of compound **28a** were below LLOQ in all brain samples (<4 nM) and in the majority of CSF samples (<2 nM). These results indicate that compound **28a** is largely peripherally restricted and does not cross the blood-brain barrier to a significant extent.

### Ultra-large chemical libraries tailored for peptide-binding receptors

**Fig. 7:**
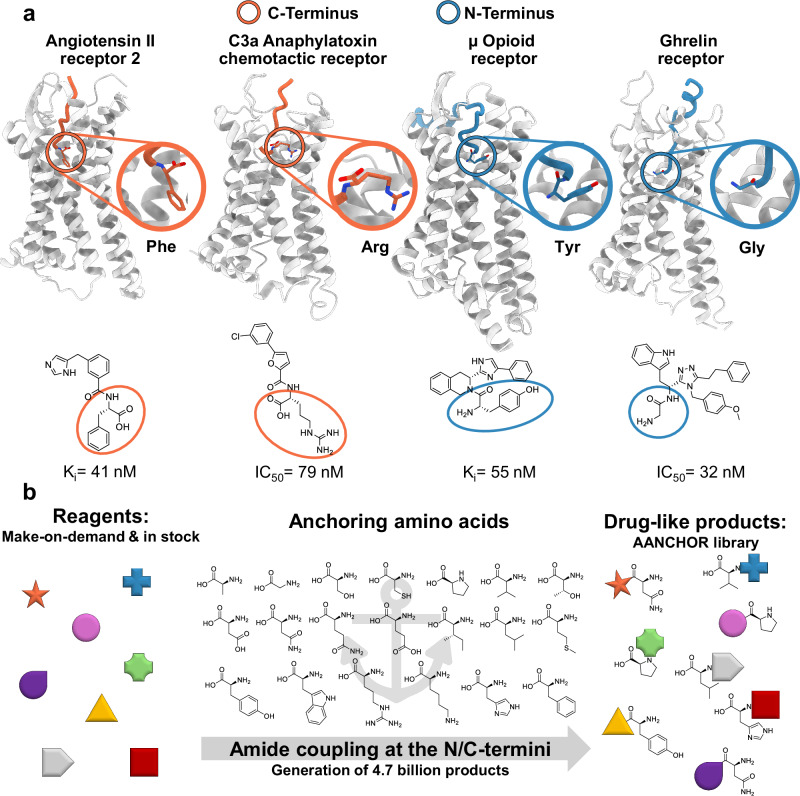
General applicability of the virtual screening strategy. **a** Experimental structures of four GPCRs in complex with peptides (angiotensin II receptor 2^66^, C3a anaphylatoxin chemotactic receptor^65^, μ-opioid receptor^67^, ghrelin receptor^68^ with PDB accession codes 6JOD, 8I95, 8F7Q, and 7W2Z, respectively). Peptide agonists with their C-terminal residue deeply buried in the orthosteric binding site are highlighted in orange, while peptides anchored by their N-terminal residues are shown in blue. Potent ligands based on a single amino acid are shown below each structure (from the ChEMBL database, IDs: 219839, 231405, 361274, and 400814)^64^. In each structure, the amino acid in the small-molecule ligand matches the terminal residue of the endogenous peptide agonist that is anchored in the binding site. The virtual screening approach developed in this study is hence applicable to all of these drug targets. Four additional targets for our approach are shown in Supplementary Fig. S12. **b** Generation of AANCHOR (Amino Acid N/C-termini Hybrids Optimized for Receptors), a database of compounds that can be synthesized from commercially available building blocks using amide coupling. A detailed description of the database is available in Supplementary Fig. S13.

Our virtual screens for NTSR agonists leveraged the fact that the C-terminal amino acid of the peptide agonist is firmly anchored in the orthosteric binding site (Fig. 1). To assess the broader applicability of this approach, we analyzed available bioactivity and structural data for all peptide-binding GPCRs. Ligands of 83 peptide-binding GPCRs were available in the ChEMBL database^64^, and the vast majority of these were peptides (84%), underscoring the scarcity of small-molecule ligands. Among the non-peptide ligands, we identified 144 molecules containing a single terminal amino acid that matched the endogenous agonist peptide of the target, which was the core design principle of our virtual libraries (Supplementary Fig. S11). Notably, among the nine GPCRs binding these ligands, there were established drug targets (*e.g*., angiotensin and opioid receptors), as well as emerging therapeutic targets such as ghrelin and C3a receptors. Structural analyses further revealed that, as for NTS_1_R, the C-terminal residue of the peptides is anchored in the orthosteric binding site of angiotensin and C3a receptors^65,66^. In contrast, the N-terminal residue plays the same role in the μ-opioid and ghrelin receptors (Fig. 7)^67,68^. In all four cases, the small-molecule ligands contained the terminal amino acid that is anchored in the binding site. These observations suggest that our strategy can be applied to other targets.

Recently, the number of GPCR-peptide structures has increased rapidly, and there are several suitable targets for our approach (*e.g*., neurokinin, apelin, motilin, and formyl-peptide receptors) (Supplementary Fig. S12). To enable virtual screens for chemical probes, we share a chemical database containing 4.7 billion compounds based on N- or C-terminal amino acids, which can be readily synthesized from commercially available building blocks (AANCHOR library: Amino Acid N/C-termini Hybrids Optimized for Receptors, 10.5281/zenodo.15094801, Supplementary Fig. S13).

## Discussion

Three main findings emerged from our structure-based virtual screens for small-molecule agonists of the neurotensin G protein-coupled receptors, a family of challenging peptide-binding drug targets. First, non-peptide agonists of NTS_1_R were identified through the computational docking of two virtual chemical libraries. The most potent agonists were discovered from a library specifically designed for the target, an approach we believe is generally applicable to peptide-binding receptors. Second, structure-guided optimization led to the discovery of agonists with signaling signatures distinct from the native peptide. Crystal structures of the receptor-agonist complexes confirmed the computational models and helped rationalize structure-activity relationships. Finally, low-nanomolar NTS_2_R selective compounds produced analgesia in a rodent model, providing starting points for the development of novel drugs to treat pain.

Development of small-molecule agonists for peptide-binding GPCRs is challenging because such compounds must both capture the key pharmacophoric features for receptor activation and maintain drug-like properties. Our study demonstrates that access to the structures of receptor-peptide complexes can accelerate small molecule discovery by guiding the design of libraries covering the chemical space relevant for the target. At the time of our virtual screen, several hundred million compounds were commercially available via make-on-demand libraries^36^, but only a very small fraction of these were anions or contained a C-terminal leucine. This unexpected limitation of the commercial chemical space may have contributed to the fact that only one of the selected compounds (4%) from the diverse (anion-based) library activated NTS_1_R. Our focused library provided orders of magnitude better coverage of the relevant chemical space than the commercial databases and resulted in a nine-fold higher hit rate (38%) than the diverse library. For comparison, a high-throughput screen of several hundred thousand compounds for NTS_1_R agonists resulted in a hit rate of less than 0.1%^69^. These observations agree with recent studies demonstrating that focused libraries can enable efficient ligand discovery for GPCRs recognizing small molecules, *e.g*. serotonin and cannabinoid receptors^70,71^. The increased hit rate does come at the expense of novelty, as part of the endogenous agonist is used as a template for the library. This strategy is therefore most suitable for challenging targets expected to yield low virtual screening hit rates, which includes many peptide-binding GPCRs. We believe our approach to design chemical libraries can be generally applicable to peptide-binding targets, and we share a unique database containing more than four billion compounds. Crystal structures of NTS_1_R bound to compounds **28a** and **30a** confirmed that the overall binding modes of the agonists were correctly predicted, but also illustrated well-known limitations of docking algorithms, including the lack of induced fit and poor treatment of ligand strain energy^72^. In future work, efficiency in both ligand discovery and optimization could be enhanced by machine-learning methods for accelerated library screening and compound selection, molecular dynamics simulations to guide analog design, and computational tools for synthesis planning and prediction of pharmacokinetic properties^73–76^.

Structural comparisons of GPCRs bound to diverse agonists can shed light on the mechanism of receptor activation and enable the design of ligands exhibiting specific efficacy profiles^77,78^. In the case of NTS_1_R, even small changes in the chemical structure of the ligands can have a major impact on efficacy. An intriguing example is the partial agonist **RTI-3a**, which was derived from the inverse agonist **SR48692** by replacing its adamantyl group with a leucine^20,22^. The terminal leucine is a hallmark of nearly all NTS_1_R agonists, but the efficacy profiles of the small molecules containing this moiety range from antagonism to partial and full agonism, with signaling signatures distinct from NTS_8–13_. While **RTI-3a** is only a weak partial agonist, compounds **28a** and **30a** identified in the present study exhibit full agonism. Additionally, and contrary to expectations, compound **35a** carrying an adamantyl group instead of leucine is a strong partial agonist. Collectively, these results show that a fine balance of interactions in the orthosteric site determines agonist efficacy. In particular, the NTS peptide and small-molecule agonists stabilize the extracellular inclination of TM6, TM7, and ECL3 to different extents, which could indirectly influence the G protein binding site. The ligand binding site conformations observed in the crystal structures may hence account for the differential abilities of the agonists to activate G_q_, G_i_, and G_s_. As the Gα subtypes have unique C-terminal sequences, and sometimes differ in their tilt when interacting with the intracellular receptor interface, ligands stabilizing distinct receptor conformations could promote the activation of a subset of signaling pathways. Moreover, the interaction with β-arrestin is generally considered to require smaller openings on the cytoplasmic face, caused by the helical tilt induced by agonists^79^. The recently determined structures of NTS_1_R-agonist complexes bound to G proteins and β-arrestin will pave the way for a deeper understanding of the molecular basis of pathway-specific receptor activation^28–33^.

Our results provide insights into why identifying small-molecule NTS_1_R agonists with high affinity has proven so difficult. While NTS_8–13_ binds with subnanomolar affinity to both NTS_1_R and NTS_2_R, the small-molecule full agonists discovered in this study (**28a** and **30a**) and by others (*e.g*., **SRI-9829**)^24^ exhibit only micromolar affinities for NTS_1_R. Surprisingly, these small-molecule agonists display nanomolar affinities for NTS_2_R, despite the highly conserved orthosteric sites. A plausible explanation for this intriguing disparity in affinities is the possible bias of NTS_1_R and NTS_2_R for inactive or active-like conformational states, respectively. The substantially higher affinities of compounds **28a**, **30a**, and **SRI-9829** for our stabilized receptor NTSR1-H4_X_, compared to wild-type NTS_1_R, can be likely attributed to the conformational bias of NTSR1-H4_X_ toward an active-like state in the extracellular region with high affinity for agonists^24^. This suggests that wild-type NTS_1_R primarily adopts inactive conformations in the apo state, with a high energetic cost required to stabilize the active-like state of the orthosteric site observed by crystallography of NTSR1-H4_X_ with agonists. Conversely, active-like conformations with high agonist affinity could be more easily accessible for NTS_2_R, as supported by its pronounced constitutive activity^80^. GPCR activation frequently involves compaction of the orthosteric site, augmenting receptor-ligand interactions and enhancing agonist affinity^77^. In this context, the Arg_8_–Arg_9_–Pro_10_ residues of NTS_8–13_ are crucial for stabilizing the inward inclinations of TM6, TM7, ECL3, and the N-terminal region, which contract and occlude the NTS_1_R orthosteric site^24^. The small-molecule agonists essentially lack interactions mimicking those of Arg_8_, Arg_9_, and Pro_10_, likely preventing the stabilization of high agonist-affinity states in wild-type NTS_1_R. However, further compound optimization to form interactions in these subpockets would lead to increased molecular weight, illustrating the challenges inherent in the development of small-molecule agonists of peptide-binding GPCRs. Access to structural data and more detailed understanding of the key interactions driving ligand binding will facilitate the design of agonists with drug-like properties.

The extensive research efforts focused on NTS_1_R and NTS_2_R have been driven by their potential as drug targets for treating pain, substance abuse, Parkinson’s disease, and schizophrenia^10,11^. For central nervous system disorders, small molecules are generally considered more suitable therapeutic agents than peptides. Compound **28a**, which demonstrated antinociceptive effects in two pain models and possesses drug-like properties, represents a promising starting point for further optimization of pharmacokinetic properties, with particular focus on improving blood-brain barrier penetration. There are several promising strategies for the development of small-molecule drugs targeting NTSRs. A major challenge in targeting the NTSRs is to avoid the undesired side effects caused by NTS_1_R activation. A first potential strategy is to develop ligands that selectively activate signaling pathways with therapeutic effects, while avoiding those associated with side effects^81^. The β-arrestin-biased allosteric modulator **SBI-810** of NTS_1_R was recently reported to exhibit promising activity in animal models of acute and chronic pain without leading to the side effects caused by G protein activation^82^. Our study shows that it is possible to design small-molecule orthosteric agonists with distinct signaling signatures. The scaffolds discovered in this study may provide a starting point for developing a portfolio of biased NTS_1_R agonists. However, clear connections between specific signaling signatures and the desired pharmacological outcomes in vivo remain to be established. Furthermore, we discovered that binding of an allosteric modulator to NTS_1_R depended on the orthosteric small-molecule agonist used. This intriguing probe-specific behavior may reflect the extent of helix movement elicited by different ligands, which could be leveraged in drug discovery by using combination therapy^46^. A second strategy to avoid NTS_1_R-associated side effects is identifying selective ligands of the NTS_2_R subtype, a promising target for the treatment of acute and chronic pain^9,14,15^. Compounds **28a**, **30a**, and **35a** are small-molecule agonists of this target that show analgesic effects in vivo. Notably, compound **35a** shows very high affinity for NTS_2_R, and because this ligand is selective for this subtype, no hypotensive side effects were observed. These findings underscore the potential of NTS_2_R ligands to provide safer and more effective alternatives to opioids for pain management.

## Methods

### Ethics statement

All experimental procedures were approved by the Animal Care Committee of the Université de Sherbrooke (protocol no. 035 − 18B) and conducted in accordance with the guidelines of the Canadian Council on Animal Care.

### Computational chemistry

#### Generation of the focused and diverse chemical libraries

The diverse chemical library was generated by extracting anionic molecules from the ZINC15 database (purchasability = “Wait OK”, reactivity = “Clean”, 350 Da < Molecular Weight (MW) < 400 Da, clogP <3.5, pH = “Ref-Mid”, Charge = −1)^36^. The total size of the library was 8,226,610 unique compounds. The focused chemical library was generated by connecting building blocks to the amino group of leucine by an amide coupling reaction. Building blocks containing a carboxylate, a methyl ester or an ethyl ester as reactive functional groups were extracted from the ZINC15 library^36^ (purchasability = “Wait OK”, reactivity = “Clean” and MW < 400 Da) by using substructure searches and chemical property filtering with OECHEM 2.1.3 and OMEGA/FILTER 3.1.2.2^83^. The RDKit package^84^ was then used to generate the final compounds by reacting the building blocks with the amino group of the leucine. Finally, all compounds with an MW above 460 Da were removed, resulting in a library of 6,345,793 unique compounds. The focused library was then prepared for docking using the ZINC database protocol^36^. For each molecule, tautomers and protonation states with >20% occupancy (pH range 6.9−7.9) were considered using cxcalc version 18.9.0^85^, and up to 200 conformations were generated using OMEGA^86^.

#### Molecular docking

Molecular docking was performed using the program DOCK3.7^38^. The structure of the stabilized rNTS_1_R bound to the partial agonist **RTI-3a** (PDB accession code: 6ZA8)^24^ was prepared using PyMOL version 2.3.0 (Schrödinger, http://www.pymol.org/pymol). Mutations were introduced into the structure to create a model of the hNTS_1_R, and the residue numbers in this section correspond to the hNTS_1_R. Protonation states of ionizable residues Asp, Glu, Lys, Arg, and His were automatically assigned to their most probable state at pH 7 by the program reduce^87^. Binding site residues His131^ECL1^ and His343^7.32^ were protonated at the ε and δ positions, respectively. The dipole moments of the Tyr145^3.29^, Thr225^ECL2^, and Tyr342^7.31^ side chain hydroxyl groups were increased to favor polar interactions with these residues^37^. The grids needed to calculate the docking score (electrostatic, van der Waals and desolvation energies) were prepared using DOCK3.7. In the calculation of electrostatic potentials using QNIFFT^88,89^, a set of spheres was added on the protein surface to define the boundary between solute and solvent. A second set of spheres was used to prepare the grids that determine ligand desolvation penalties. Control calculations to optimize docking performance were primarily performed using a set of 14 diverse NTS_1_R agonists from the ChEMBL database^64^ and 951 property-matched decoys^90^. In addition, four sets of property-unmatched decoys were generated to assess the balance between electrostatic interactions and desolvation energies. These decoys had the net charge shifted by –2, –1, +1, and +2 relative to the NTS_1_R agonists (365, 703, 923, and 818 decoys, respectively). We then evaluated different radii of the spheres (0.2–2.0 Å, a total of 121 combinations)^37^. Based on ligand enrichment (quantified using the adjusted LogAUC^90^) and redocking of **RTI-3a**, we selected values of 1.6 and 1.2 Å for the electrostatic and ligand desolvation spheres, respectively. The heavy atoms of **RTI-3a** were used to define the binding site by generating 41 matching spheres onto which pre-calculated conformations of the docked compound were superimposed. In the screening of the chemical libraries, DOCK3.7 typically sampled thousands of compound orientations within the receptor binding site and, for each orientation, scored hundreds of pre-calculated conformations. The lowest-energy pose of each compound was subsequently refined by 500 steps of rigid-body minimization, and the resulting docking score was used to rank the library^38^. The diversity among the top-ranked compounds was increased by clustering the top 10% top-scoring molecules using ECFP4-based fingerprints implemented in RDKit^84^ and a Tanimoto coefficient of 0.6 and 0.5 for the focused and diverse chemical library, respectively. The best scoring member of the cluster was chosen as a representative, and 1,000 compounds from each library were visually inspected to select compounds for synthesis and experimental evaluation. Compounds selected from the docking calculations were either purchased from commercial make-on-demand libraries (Supplementary Table S11) or synthesized in-house (detailed synthetic procedures are described in the Supplementary Methods).

#### Generation of chemical libraries for hit optimization

In a first iteration of optimization of compound **25**, in-house synthesis was combined with selections from the Enamine REAL Space make-on-demand database^91^. Substructure searches in the Enamine REAL Space database were performed with OpenEye’s OEToolkits^83^. In parallel, virtual libraries of analogs substituted at either positions 1 or 6 on the pyrazolo[3,4-b]pyridyl scaffold were generated from commercially available building blocks from the ZINC15 database (subset = “building block”, purchasability level = “50”, reactivity = “clean”)^36^. Building blocks containing unique acetyl, halogen or boronic acid reactive groups compatible with condensation or Suzuki coupling reactions at position 6 and alkyl/aryl halides and hydrazine containing building blocks compatible with an alkylation reaction at position 1 were identified with OpenEye’s OEToolkits^83^. The RDKit package^84^ was used to generate the final compounds by reacting the building blocks with the core scaffold, leading to a virtual library of 4,445 and 5,633 unique analogs substituted at positions 1 and 6, respectively. In a second iteration of optimization, substructure searches for 1,6-disubstituted pyrazolo[3,4-b]pyridine-4-carboxylic acid and acid halides were performed in the Enamine REAL Space database and Enamine building block library. The substructure search included a methylene linker at position 1 or analogs thereof (carbonyl and sulfonyl linkers). For position 6 of the pyrazolo[3,4-b]pyridine, cyclopropyl, 2,5-dimethylfuran- or -thiophene-3-yl, phenyl, and azines were included in the searches.

In the case of compound **24**, a virtual chemical library of analogs substituting the 5-methylthiophen-2-yl at position 3 with other five- and six-membered ring moieties was first generated. Aryl halide (24,892 molecules) and boronic acid (4,388 molecules) building blocks were retrieved from Enamine. The RDKit package^84^ was then used to generate a virtual library through in silico Suzuki coupling, which contained 5683 unique molecules with an MW less than 500 Da. In a second step, sets of building blocks needed to explore substitutions on the tetrazole moiety of compound **24** were obtained from Enamine, and these libraries contained 39,000 carboxylic acids and 800 acyl halides. If multiple reaction sites were possible (e.g., for dicarboxylic acids), all combinations of products were generated. In silico tetrazole condensation returned a total of 69,976 compound **24** derivatives. In addition, the amine groups in the acyl halide and carboxylic acids were deprotected from the fluorenylmethyloxycarbonyl (Fmoc), tert-butyloxycarbonyl (Boc), benzyloxycarbonyl (Cbz), and benzyl (Bn)-groups in the final products, returning approximately 9,000 additional compounds. After removing duplicates, the library contained 10,500 unique compound **24** derivatives with an MW less than 500 Da.

#### Analysis of ligands and experimental structures of peptide-binding GPCRs

A set of 91 human peptide-binding GPCRs was retrieved using the GPCRdb web services (https://gpcrdb.org/services/receptorlist/)^6^. Bioactivity data for these GPCRs were obtained from the ChEMBL database (version 34)^64^. Only bioactivity data from published literature (document type = “Publication”) were considered, and ligands with reported *K*_i_, *K*_d_, IC_50_, or EC_50_ values better than 10 µM for the GPCRs were extracted. Molecular structure standardization, including the removal of salts and minor components, tautomerization, and charge neutralization, was then performed using the canSARchem registration workflow^92^. The resulting data set contained 41,266 unique ligands with activity at 83 of the peptide-binding GPCRs. After filtering for compounds containing an amidated amino acid moiety, 6708 ligands remained. Out of these ligands, non-peptide small molecule ligands containing N- or C-terminal moieties were then identified using SMARTS-based filtering in KNIME 4.6.3 with RDKit extension^84,93^. A set of 297 unique non-peptide compounds (i.e., not containing two consecutive alpha-amino acid moieties connected by an amide bond) with activity at 37 different peptide-binding GPCRs were identified. These compounds and receptors were then further analyzed by comparing with the endogenous peptide ligands and all available experimental structures of these GPCRs, which were obtained using the GPCRdb web services (https://gpcrdb.org/services/ligands/peptides/). In this case, we included ligands complexed with receptors from all species. The analysis revealed 144 unique ligand-receptor pairs, corresponding to 135 unique compounds, with nine ligands sharing activities at two receptors.

#### Generation of the AANCHOR library: amino acid N/C-termini hybrids optimized for receptors

Chemical libraries tailored for peptide-binding GPCRs were generated by amide coupling reactions between: (1) The backbone carboxylic acid and amino group of 20 proteinogenic amino acids, their N-formylated, and C-amidated derivatives and (2) acyl halides, carboxylic acids/carboxylates, esters, and compounds containing a primary or secondary amino group (or analogs of thereof: hydrazines, hydroxylamines). The acylation and amine building blocks (2) were obtained from the Chemspace in-stock library (686,000 compounds) and Enamine REAL Space (39 billion compounds)^91^. The acylation and amine building blocks were stratified according to their theoretical reactivity to prevent potential side reactions (i.e. self-coupling, mixtures of regioisomers, or oligomerization). Each building block was allowed to contain only one of the most reactive groups and zero of the more reactive groups, e.g. the carboxylic acids were allowed to have maximally one carboxylic acid and no acyl halide, primary or secondary amine. The RDKit package^84^ was then used to generate the final compounds by reacting amino acids with the prefiltered building blocks (amino acids + acyl halides, carboxylic acids, esters, amines; C-amidated amino acids + acyl halides, carboxylic acids, esters; N-formylated amino acids + amines). Finally, all product molecules with a heavy atom count greater than 28 were removed (approximately MW < 500 Da), resulting in a library of over 4.7 billion unique drug-like compounds.

### In vitro **biological assays**

#### Cell culture

HEK293T cells (gift from the Chair of Physiology, FAU Erlangen-Nürnberg) and HEK293 cells stably expressing β-arrestin2 tagged to the PathHunter enzyme acceptor, a fragment of β-galactosidase (β-arrestin HEK293, DiscoverX), were maintained in a humidified atmosphere at 37 °C and 5% CO_2_ in DMEM-F12 (Gibco Life Sciences) supplemented with 10% fetal bovine serum (FBS), 100 µg mL^−1^ penicillin, 100 µg mL^−1^ streptomycin and 2 mM L-glutamine. For the β-arrestin HEK293 cells, additional 150 µg mL^−1^ hygromycin was added. Cells were grown on 10 cm dishes, subcultured every 3–4 days, and regularly confirmed to be free of mycoplasma contamination using the MycoalertPlus detection kit (Lonza).

#### IP_1_ accumulation assays for hNTS_1_R and NK1 receptor

Determination of the G_q_ signaling by NTS_1_R was measured applying the IP-One HTRF® assay (Revvity-Cisbio, Codolet, France) according to the manufacturer’s protocol and as described previously^94^. In brief, HEK-293T cells were grown to a confluence of approx. 70% and transiently transfected with 0.5 µg of the plasmid encoding the human NTS_1_R (cDNA Resource Center, Bloomsberg, PA) and 2.5 µg of a mock plasmid applying the Mirus TransIT-293 transfection reagent (Peqlab, Erlangen, Germany). For control experiments with the human neurokinin 1 (NK1) receptor, 0.5 µg of a NK1 receptor encoding plasmid was used instead. After one day, cells were detached from the culture dish, seeded into black 384-well plates (10,000 cells/well, Greiner Bio-One, Frickenhausen, Germany) and maintained for 24 h at 37 °C. Cells were incubated with the compounds (final concentration range 0.01 nM to 100 μM) in duplicate for 90 min at 37 °C. For inhibition assays with SR142948A, cells were preincubated with 1.0 µM of the test compounds for 30 min, before the serial dilutions of SR142948A were added, and the incubation was continued for 60 min. The accumulation of second messenger was stopped by adding detection reagents (IP1-d2 conjugate and Anti-IP1cryptate-TB conjugate) and monitoring time-resolved FRET with a Clariostar plate reader equipped with the respective filter sets (BMG Labtec, Ortenberg, Germany). FRET-ratios were calculated as the ratio of emission intensity of the acceptor (665/10 nm) and the donor intensity (620/10 nm). Raw FRET-ratios were normalized to buffer conditions (0%) and the maximum effect of NTS_8–13_ (100%). For screening purposes, ligand effects were analyzed at three different concentrations (10, 30, and 100 µM), while subsequently determined full concentration-response curves were analyzed using the four-parameter equation implemented in GraphPad Prism 10.2 for Windows (GraphPad Software, La Jolla, USA) to derive the maximum efficacy (*E*_max_) and ligand potency (EC_50_). For each compound, *n* = 2–17 independent experiments were performed with each concentration in duplicate.

#### IP_1_ accumulation assays for rNTS_1_R

Ligand-induced IP_1_ accumulation was measured essentially as described previously^24^. HEK293T cells were transiently transfected with a pcDNA5 expression vector encoding rNTS_1_R and were seeded at 20,000 cells per well in poly-L-lysine-coated 384-well plates (Greiner). 48 h after transfection, cells were washed with assay buffer (10 mM HEPES pH 7.4, 1 mM CaCl_2_, 0.5 mM MgCl_2_, 4.2 mM KCl, 146 mM NaCl, 5.5 mM glucose, 0.2% (w/v) BSA) and incubated for 2 h at 37 °C with a concentration range of ligands diluted in assay buffer supplemented with 50 mM LiCl. IP_1_ accumulation was determined using the HTRF IP-One kit (Cisbio) according to the manufacturer’s protocol. Fluorescence intensities were measured on a SPARK fluorescence plate reader (Tecan). To generate concentration-response curves, data were analyzed by a three-parameter logistic equation in GraphPad Prism software (version 6.07).

#### IP_1_ accumulation assays with digested murine left-ventricular heart tissue

IP_1_ production was determined using the IP-One Gq-HTRF kit (Revvity Germany GmbH, 62IPAPEB) according to the manufacturer’s instructions. Left ventricles were digested using dispase (1.2 units/mL), collagenase II (2 mg/mL), and elastase (0.02 mg/mL) in serum-free DMEM, then washed and resuspended in 1× stimulation buffer (8000 cells in 7 μL). 7 μL cells/well were seeded into a white opaque 384-well plate (PerkinElmer, 6007680), then 7 µL/well of ligand solution dissolved in stimulation buffer were added to produce the following final concentrations: Phenylephrine (50 µM, Sigma-Aldrich), NTS_8-13_ (10 nM, Abcam), **NT150**^51^ (100 nM), **28a** (100 nM and 1 µM), **30a** (100 nM and 1 µM), **35a** (100 nM and 1 µM), **SRI-9829** (100 nM and 1 µM), and **RTl-3a** (100 nM and 1 µM). Cells were incubated in the presence/absence of ligands for 30 min at 37 °C, 5% CO_2_. After this, samples were processed in accordance with the manufacturer’s instructions. The HTRF signals were determined under Terbium cryptate mode with FlexStation 3 (Molecular Devices) at 620 nm (donor) and 665 nm (acceptor), and the results were calculated based on the extrapolation from the standard curve equation using SoftMax Pro 7 software. Differences in protein input between wildtype and NTS_2_R-KO were adjusted based on BCA protein measurements. Statistical analyses were performed using GraphPad Prism 10 (version 10.1.2). NTS_2_R-KO mice were generated as described in ref. ^52^

#### G_q_/G_i1_ protein activation

The NTS_1_R-mediated activation of G_q_ and G_i1_ proteins was determined by BRET based on the separation of RlucII-Gα_q_ or RlucII-Gα_i1_ from Gβ/Gγ_2_-GFP10 as described previously^95,96^. In brief, HEK293T cells were detached from their culture dish, diluted to a density of 2.5 ∙ 10^5^ cells mL^−1^ and transfected in suspension using PEI (25 kDa, linear) at a 3:1 reagent to DNA ratio. Per 3 ∙ 10^5^ cells, 100 ng of the NTS_1_R plasmid together with the BRET biosensor at a receptor:Gα:Gβ:Gγ ratio of 2:1:2:5, and the total DNA amount was completed to 1 µg with single-stranded DNA (ssDNA, Sigma Aldrich) for the activation of G_q_. Similarly, transfections for the activation of G_i_ were conducted with 200 ng of the receptor plasmid together with the BRET biosensor at a receptor:Gα:Gβ:Gγ ratio of 4:1:2:8. Directly after transfection, cells were transferred to white 96-well plates (Greiner BioOne, 20,000 cells/well) and cultured for 48 h at 37 °C, 5% CO_2_. On the day of the assay, the medium was replaced by PBS followed by an equilibration period of 45 min at 37 °C. Ligand stock solutions (in DMSO) were diluted in PBS and incubated for 30 min at 37 °C. Five minutes before the measurement, coelenterazine 400a (Iris Biotech) was added at a final concentration of 5 µM. Bioluminescence was recorded using a Clariostar plate reader (BMG Labtech, Ortenberg, Germany) equipped with the respective filter set (410–80 nm (donor) and 515–30 nm (acceptor)), and BRET was determined as the ratio of the light emitted by the acceptor divided by the signal emitted from the donor. Responses were normalized to the maximum effect of NTS_8–13_ (100%) and basal conditions (0%). Concentration-response curves were analyzed using the algorithms for four-parameter non-linear regression implemented in GraphPad Prism 10.2 for Windows. For each compound, *n* = 3–8 independent experiments were performed, with each concentration in duplicate.

#### cAMP accumulation

To determine NTS_1_R-mediated G_s_ activation, accumulation of cAMP was measured with the GloSensor cAMP assay (Promega, Madison, USA) according to the manufacturer’s protocol. HEK293T cells were transfected with 1 µg NTS_1_R (in pcDNA3.1) and 2 µg of the 22F-GloSensor plasmid (Promega), a fusion of firefly luciferase to a cAMP binding domain, using TransIT293. 24 h after transfection, cells were detached with culture medium, and 10,000 cells/well were distributed to a white 384-well plate (Greiner BioOne). Cells were cultured for 24 h at 37 °C in 5% CO_2_, before the medium was replaced with 20 µL of assay buffer (3% GloSensor cAMP Reagent in HBSS). Cells were equilibrated for 60 min in the dark at room temperature, before basal luminescence was measured with a Clariostar plate reader (BMG Labtech). Ligand stock solutions diluted with HBSS were added (5 µL) and cells were incubated with the ligands for 30 min in the dark at room temperature. For inhibition assays, cells were incubated for 15 min with the test compounds at various concentrations, before 20 nM NTS_8–13_ were added, and cells were incubated for further 15 min. Luminescence was recorded with a Clariostar plate reader (BMG Labtech) and the obtained signals were normalized to basal conditions (0%) and the maximum response of NTS_8–13_ (100%). Concentration-response curves were analyzed with the algorithms for four-parameter non-linear regression in GraphPad Prism 10.2 to derive the relative efficacy (E_max_) and potency (EC_50_). For each compound, n = 3-6 independent experiments were performed, with each concentration in duplicate.

#### β-arrestin-2 recruitment (PathHunter)

β-arrestin-2 recruitment was investigated employing the PathHunter assay as described previously^43^ and according to the manufacter’s protocol (DiscoverX, Eurofins). β-arrestin HEK293 (expressing β-arrestin fused to the large fragment of β-galactosidase) were transiently transfected with 0.5 µg of the plasmid encoding the NTS_1_R-PK1 construct (a fusion to a small fragment of β-galactosidase), together with or without 0.5 µg GRK2 plasmid, and complemented to a total amount of 3 µg per plate with a mock pcDNA3.1 plasmid using TransIT293. 24 h after transfection, cells were detached using Versene (Invitrogen), resuspended in cell plating reagent 7 (fluorescent β-gal substrate CP7, DiscoverX) and seeded in white 384-well plates (Greiner BioOne) at a density of 5000 cells/well. After cultivation at 37 °C, 5% CO_2_ for 24 h, cells were stimulated with the test compounds for 90 min at 37 °C. 10 µL of the detection mix was added, and chemiluminescence was measured with a CLARIOstar microplate reader after incubation for 60 min in the dark at room temperature. Data were normalized to the basal luminescence and maximum response of NTS_8–13_ and analyzed by four-parameter nonlinear regression using the algorithms of GraphPad Prism 10.2 for Windows. For each compound, *n* = 4–12 independent experiments were performed, with each concentration in duplicate.

#### β-arrestin-2 recruitment (BRET)

The recruitment of β-arrestin-2 was determined by BRET between NTS_1_R-Rluc8 and β-arrestin-2-mVenus as described previously^47^. The transfection was carried out in suspension as described for the activation of G_q_ with the following DNA amounts per 3 ∙ 10^5^ HEK293T cells: 70 ng NTS_1_R-Rluc8, 200 ng β-arrestin-2-mVenus, 35 ng GRK2, and 90 ng ssDNA. 20,000 cells per well were directly distributed to white 96-well plates (Greiner BioOne) and grown for 48 h at 37 °C, 5% CO_2_. On the day of the assay, the growth medium was replaced with PBS, and cells were equilibrated for 45 min at 37 °C before ligand dilutions were added. Cells were incubated for 30 min at 37 °C and coelenterazine-h (Biomol, 5 µM final concentration) was added 20 min before the measurement. BRET was determined as described for the activation of Gα_q_, but with the respective 475/30 nm and 535/30 nm emission filters. Responses were normalized to the maximum effect of NTS_8–13_ (100%) and basal conditions (0%) and concentration-response curves were analyzed using the algorithms for four parameter non-linear regression in GraphPad Prism 10.2 to derive the compounds’ relative efficacy (E_max_) and potency (EC_50_). For each compound, *n* = 4–7 independent experiments were performed, with each concentration in duplicate.

#### Radioligand binding assays for hNTS_1_R, hNTS_2_R, µOR, κOR, δOR, 5-HT_1A_, and α_2A_ receptors

Binding affinities of the compounds for the hNTS_1_R and hNTS_2_R were determined with membrane preparations from HEK293T cells transiently transfected with the respective receptor plasmids (cDNA Research Center, Bloomsberg, PA) in analogy to a previously described protocol^97^. Hence, membranes expressing the respective receptors (NTS_1_R: *B*_max_ 7600 ± 750 fmol/mg protein, protein concentration 1–4 µg/well; NTS_2_R: *B*_max_ 850 ± 120 fmol/mg protein, protein concentration 6–10 μg/well) were incubated together with the radioligand [^3^H]UR-MK300^50^ (specific activity 2.41 TBq/mmol, *K*_D_ 0.66 nM) at a final concentration of 0.50–0.70 nM with varying concentrations of the test compounds for 60 min at 37 °C. Non-specific binding was determined in the presence of 10 µM NTS_8-13_. For the determination of binding affinities to related GPCRs, homogenates from HEK cells were used which were transiently transfected with the plasmids for the human opioid receptor subtypes µOR, κOR and δOR, the serotonin receptor 5-HT_1A_ and the adrenergic α_2A_ (all from cDNA Center). Appropriate membranes (for µOR: B_max_ = 6200 fmol/mg protein, protein concentration of 2 µg/well; for κOR: B_max_ = 4500 fmol/mg protein, protein concentration of 2 µg/well; for δOR: B_max_ = 1500 fmol/mg protein, protein concentration of 6 µg/well; for 5-HT_1A_: B_max_ = 3400 ± 1100 fmol/mg protein, protein concentration of 3-5 µg/well; for α_2A_: B_max_ = 3000 ± 1600 fmol/mg protein, protein concentration of 3–10 µg/well) were incubated with the radioligand [^3^H]diprenorphine (from PerkinElmer, Rodgau Germany, specific activity 30 Ci/mmol; K_*D*_ 0.15 nM for µOR, K_*D*_ 0.070 nM for κOR, K_*D*_ 0.15 nM for δOR) at a final concentration of 0.2 nM, with [^3^H]WAY100635 (from Novandi, Södertälje, Sweden; specific activity 83 Ci/mmol; K_*D*_ 0.070 nM for 5-HT_1A_) at 0.2 nM or with [^3^H]RX821002 (Novandi; specific activity 51 Ci/mmol; K_*D*_ 0.63 ± 0.03 nM for α_2A_) at 0.5 nM, respectively. Non-specific binding to µOR, κOR, δOR was measured in the presence of 10 µM naloxone or 10 µM of unlabeled ligand for 5-HT_1A_ and α_2A_. Experiments were performed as described above using different buffer systems (buffer A (50 mM Tris, pH 7.4) for µOR, κOR, δOR, and α_2A_; buffer B (50 mM Tris, 0.1 M EDTA, 5 mM MgCl_2_, 100 µg/mL bacitracin, pH 7.4) for 5-HT_1A_). Reactions were terminated by rapid filtration through GF/B filters, dried filters were sealed with scintillation wax, and bound radioactivity was detected with a MicroBeta2 counter (Perkin Elmer, Rodgau, Germany). Competition binding curves were analyzed by three-parameter nonlinear regression using the algorithms implemented in GraphPad Prism10.2 for Windows to calculate an IC_50_ value and the derived affinity constant *K*_*i*_ according to the equation of Cheng and Prusoff^98^. Per compound *n* = 5–10 independent experiments were performed with each concentration in triplicate.

#### Radioligand binding assay for rNTS_2_R

HEK293 cells stably expressing rNTS_2_R were cultured in DMEM. Culture media were supplemented with 10% FBS, 100 U/mL penicillin, 100 μg/mL streptomycin, 20 mM HEPES, and cells were incubated at 37 ^◦^C in a humidified chamber at 5% CO_2_. All media and additives were from Wisent (St-Bruno, QC). Compounds were first resuspended in 100% DMSO at 100 mM and subsequently diluted to a 10-mM working solution to limit precipitation. Competitive radioligand binding experiments were performed by incubating 50 μg of freshly prepared cell membranes with 105 pM [^125^I]-Tyr^3^-NT (2200 Ci/mmol; Revvity, Waltham, MA, USA) in binding buffer (50 mM Tris-HCl, pH 7.5, 0.2 % BSA, 2% DMSO). The compounds were tested in dose-response displacement curves at increasing concentrations ranging from 10^−11^ to 10^−5^ M in the final reaction wells. After 1 h of incubation at room temperature, the binding reaction mixture was transferred to polyethyleneimine-coated 96-well-filter plates (Millipore, Billerica, MA). The reaction was terminated by filtration, and plates were washed three times with 170 μl of ice-cold binding buffer. Glass fiber filters were then counted in a gamma-counter (1470 Wizard2, PerkinElmer). Non-specific binding was measured in the presence of 10^−5^ M unlabeled NTS_8–13_ and represented <5% of total binding. IC_50_ values were determined from the competition curves as the unlabeled ligand concentration inhibiting half of the ^125^I-Tyr3-NTS-specific binding.

Binding data of the dose-response experiments were plotted using GraphPad Prism 9.3 using the One-site—Fit log (IC_50_) and normalized to the NTS_8–13_ curve ranging from 10^−11^ to 10^−5^ M and represent the mean ± SEM of the number of experiments indicated in the figure and performed in duplicate. The IC_50_ calculated from the competitive radioligand binding assays was then transformed into *K*_i_ values using the Cheng–Prusoff equation^98^. The NTS_2_R K_d_ was determined as 0.7 nM.

#### Radioligand binding assay for the APJ receptor

HEK293 cells expressing the YFP epitope-tagged human apelin (APJ) receptor were washed once with PBS and subjected to one freeze–thaw cycle and cultured in DMEM. Culture media were supplemented with 10% FBS, 100 U/mL penicillin, 100 μg/mL streptomycin, 20 mM HEPES, and 0.4 mg/mL G418, and cells were incubated at 37 ^◦^C in a humidified chamber at 5% CO_2_. All media and additives were from Wisent (St-Bruno, QC). Compounds were resuspended in 100% DMSO at 100 mM. Competitive radioligand binding experiments were performed by incubating 15 μg of cell membranes with 0.2 nM radiolabeled [^125^I][Nle^75^,Tyr^77^]Pyr-apelin-13^35^ (820 Ci/mmol) with either Apelin-13 in increasing concentrations (10^–11^ to 10^–5^ M) or a single concentration of a small molecule (10^–4^ M). Reactions were incubated for 1 h at room temperature in a final volume of 200 μL in binding buffer (50 mM Tris-HCl, pH 7.5, 0.2 % BSA, 2% DMSO). After 1 h of incubation at room temperature, the binding reaction mixture was transferred to polyethyleneimine-coated 96-well-filter plates (Millipore, Billerica, MA). The reaction was terminated by filtration, and plates were washed three times with 170 μl of ice-cold binding buffer. Glass fiber filters were then counted in a gamma-counter (1470 Wizard2, PerkinElmer). Non-specific binding was measured in the presence of 10^−5^ M unlabeled Ape-13 and represented <5% of total binding. IC_50_ values were determined from inhibition curves as the unlabeled ligand concentration inhibiting 50% of [^125^I]-apelin-13 specific binding. Binding data of the single-dose displacement experiments were normalized to the Ape-13 curve ranging from 10^−11^ to 10^−5^ M and expressed in percent of displacement of radiolabeled ligand. The Ape-13 curve was plotted using GraphPad Prism 10.4 (La Jolla, CA, USA) using the One-site—Fit log (IC_50_) and each compound’s percent of ^[125I]^ Pyr-Apelin-13 displacement represents the mean ± SEM of two different experiments and performed in duplicate.

#### HTRF ligand-binding assays for NTSR1-H4x

Ligand binding experiments on cell membranes were essentially performed as described previously^24^. All receptor variants were cloned into a mammalian expression vector containing an N-terminal SNAP-tag (Cisbio). 5 × 10^6^ HEK293T cells in 10 cm Petri dishes were transiently transfected with receptor constructs using TransIT-293 (Mirus Bio) and 2 µg plasmid DNA. 48 h after transfection, cells were labeled with 50 nM SNAP-Lumi4-Tb (Cisbio) in assay buffer (20 mM HEPES pH 7.5, 100 mM NaCl, 3 mM MgCl_2_ and 0.2% (w/v) BSA) for 1.5 h at 37 °C. Thereafter, cells were washed four times with assay buffer and crude cell membrane extracts were prepared as described previously^99^. 0.1–1 µg membranes per well were then incubated for 4 h at room temperature in assay buffer containing fluorescently labeled peptide HL488-NTS_8–‍13_ (NTS_8–‍13_ labeled with HiLyte Fluor 488 at the N-terminus) (Eurogentec). For competition binding, 0.3–5 nM of HL488-NTS_8–‍13_ tracer peptide and the indicated concentration ranges of unlabeled competitor ligands were used. Fluorescence intensities were measured on a SPARK fluorescence plate reader (Tecan) with an excitation wavelength of 340 nm and emission wavelengths of 620 nm and 520 nm for Tb^3+^ and the fluorophore HiLyte Fluor 488, respectively. The ratio of FRET-donor and -acceptor fluorescence intensities (F520 nm/F620 nm) was calculated. Total binding was obtained in the absence of competitor, and nonspecific binding was determined in the presence of 100 μM unlabeled NTS_8–‍13_. Data were normalized to the specific binding for each individual experiment and were analyzed by global fitting to a one-site heterologous competition equation with the GraphPad Prism software (version 6.07). To obtain K_i_ values, data were corrected for fluorescent ligand occupancy of each mutant with the Cheng-Prusoff equation^98^.

#### Allosteric ligand binding

Additional binding assays were performed with the fluorescent ligand **PJ125**, a TAMRA-labeled derivative of the allosteric modulator SBI-553, as described previously (compound 14 in ref. ^47^). In brief, membranes from HEK293T expressing an NTS_1_R-Nluc fusion protein (20 µL, final protein concentration 2 µg/well) were incubated in a 384-well plate (Greiner BioOne) together with the fluorescent probe (PJ125, 5 µL, final concentration 0.68 µM) and 5 µL of the test ligands in varying concentration in nanoBRET buffer (50 mM Na_2_HPO_4_, 50 mM KH_2_PO_4_, pH 7.4, 1 mg/mL saponin, 5% FBS) for 90 min at 37 °C. After addition of 5 µL of a furimazine solution (Promega, final dilution 1:5000) and incubation for 5 min at ambient temperature, BRET measurements were carried out with a Clariostar microplate reader as described for the G_q/i_ activation assays but using 475–30 nm and 620–10 nm filters, respectively. Data were normalized to basal conditions (0%) and the maximum effect of NTS_8–13_ (100%) and analyzed using the three-parameter algorithm for non-linear regression implemented in GraphPad Prism 10.2 for Windows to derive the ligands’ maximum efficacy (E_max_) and potency (EC_50_) for the enhancement of allosteric ligand binding at NTS_1_R. For each ligand, 5-10 independent experiments were performed, with each concentration in duplicate.

### Structural biology experiments

#### Generation of the NTSR1-H4_X_ construct co-crystallized with compounds 28a and 30a

The amino acid sequence of the NTSR1-H4_X_ construct that was co-crystallized with compounds **28a** and **30a** is reported in Supplementary Fig. S14. This construct was made by modifying the sequence of wild-type rNTS_1_R (UniProt ID: P20789) as follows: introduction of 26 amino acid substitutions as in the previously reported mutant rNTSR1-H4 (Supplementary Table S12) (note that the mutation F342 → A had not been reported in the original reference^48^), deletion of N-terminal residues M1–A49 and ICL3 residues E273–T290, and fusion of L371^7.55^ at the C-terminal end of TM7 to the N terminus of the crystallization chaperone DARPin D12 (see below) via the linker sequence AEDLVEDWE, as depicted in Supplementary Fig. S14 and as previously described^24^, thereby creating a GPCR-DARPin shared helix and deleting rNTS_1_R residues V372–Y424. Compared to the originally reported DARPin D12^100^, the DARPin D12 used here and in previous studies involving GPCR-DARPin D12 fusions^24,101^ was modified as follows: deletion of residues S1 and D2 and introduction of six amino acid substitutions, i.e., L3 → K, G4 → A, K5 → R, A13 → K, L157 → A, and N158 → A (Supplementary Fig. S14). The purified NTSR1-H4_X_ construct used for co-crystallization comprises a cleaved HRV 3 C protease cleavage site at its N-terminal end (sequence: GP) and a cleaved HRV 3 C protease cleavage site at its C-terminal end (sequence: LEVLFQ).

#### Expression of NTSR1-H4_X_ in *Escherichia coli*

For expression in *E. coli*, the gene encoding the NTSR1-H4_X_ construct described above was cloned into a previously reported pBR322-derived vector^24,102^. Briefly, the resulting expression construct consisted of an N-terminal maltose-binding protein (MBP), followed by a His_6_-tag, a HRV 3 C protease cleavage site (sequence: LEVLFQGP), the receptor construct NTSR1-H4_X_ described above, a second HRV 3 C protease cleavage site (sequence: LEVLFQGP), followed by thioredoxin A (TrxA) and a C-terminal His_10_-tag. The HRV 3 C protease cleaves the peptide bond between Q and G of the above-mentioned cleavage site.

Expression was carried out in *E. coli* BL21 cells having a deletion of the *fhuA2* gene to confer phage T1 resistance (New England Biolabs), similarly as previously described^24^. A starter culture of cells harboring the pBR322-derived expression plasmid (described above) was grown overnight at 37 °C in 2×YT medium containing 1% (w/v) glucose and 100 µg/mL ampicillin. Expression cultures (1 L) consisting of 2×YT, 0.2% (w/v) glucose and 100 µg/mL ampicillin were inoculated with the starter culture to an OD_600 nm_ of ~0.05 and grown at 37 °C to an OD_600 nm_ of ~0.5, followed by induction with 1 mM isopropyl-β-D-thiogalactopyranoside (IPTG) and cultivation for ~18 h at 28 °C. Cells were harvested by centrifugation at 5000 × *g* for 20 min at 4 °C. The cell pellet was resuspended with Resuspension Buffer (100 mM HEPES pH 8.0 at 4 °C, 30% (v/v) glycerol, 400 mM NaCl) at 4 °C, frozen in liquid nitrogen, and stored at –80 °C.

#### Purification of NTSR1-H4_X_ for crystallization in the lipidic cubic phase

Purification of NTSR1-H4_X_ for crystallization in the lipidic cubic phase was carried out similarly as previously described^24^. 100 mL of frozen resuspended cells (see above, corresponding to ~33 g of pellet) were thawed and all the following steps were carried out at 4 °C. 250 mg of lysozyme (Sigma-Aldrich), 625 μL of 1 M MgCl_2_, and 625 μL of 10 mg/mL DNase I (Roche) were added and the mixture stirred for 1 h. Subsequently, receptors were solubilized by incubation with 2% (w/v) n-dodecyl-β-D-maltopyranoside (DDM, Anatrace) and 0.2% (w/v) cholesteryl hemisuccinate (CHS, Sigma-Aldrich) for 2 h while stirring, followed by sonication for 30 min (15 min sonication, 5 min pause, 15 min sonication) using a Sonifier 250 (Branson) at a duty cycle of 30% and output 5. The lysate containing detergent-solubilized receptors was adjusted to 20 mM imidazole final concentration, pH 8.0 at RT, and centrifuged at 20,000 × *g* for 30 min. The supernatant was adjusted to 0.05 mg/mL DNase I (Roche) and rolled overnight with 25 mL of TALON Superflow resin (GE Healthcare) equilibrated with Talon Wash Buffer I (25 mM HEPES pH 8.0 at 4 °C, 10% (v/v) glycerol, 600 mM NaCl, 0.1% (w/v) DDM, 5 mM MgCl_2_, 2 mM 2-mercaptoethanol (2-ME), 20 mM imidazole pH 8.0 at RT). Subsequently, the resin was washed with 11 column volumes (CV) of Talon Wash Buffer I followed by 5 CV of Talon Wash Buffer II (25 mM HEPES pH 8.0 at 4 °C, 10% (v/v) glycerol, 150 mM NaCl, 0.1% (w/v) DDM, 2 mM 2-mercaptoethanol, 20 mM imidazole pH 8.0 at RT). Protein elution was carried out with 3.8 CV of Talon Elution Buffer (25 mM HEPES pH 7.0 at 4 °C, 10% (v/v) glycerol, 150 mM NaCl, 0.1% (w/v) DDM, 2 mM 2-ME, 250 mM imidazole pH 8.0 at RT). The eluted protein was concentrated in four Vivaspin 20 concentrators (100 kDa molecular weight cut-off, Sartorius) to total 5 mL and subsequently desalted using two PD-10 G-25 columns (GE Healthcare) equilibrated with G-25 Buffer (10 mM HEPES pH 7.0 at 4 °C, 10% (v/v) glycerol, 50 mM NaCl, 0.025% (w/v) DDM, 2 mM dithiothreitol (DTT)) according to the manufacturer’s instructions. The protein sample was incubated overnight with HRV 3 C protease (produced in-house) to cleave off the fusion proteins MBP and TrxA from the receptor construct described above. The protein mixture was slowly loaded onto 2.5 mL of SP Sepharose cation-exchange chromatography resin (GE Healthcare) equilibrated with G-25 Buffer. To increase the receptor yield, the flow-through of the SP-column was loaded onto another 2.5 mL of SP Sepharose cation-exchange chromatography resin. Both SP-columns were washed with 5 CV of SP Wash Buffer I (10 mM HEPES pH 7.0 at 4 °C, 10% (v/v) glycerol, 35 mM NaCl, 0.025% (w/v) DDM, 2 mM DTT, pH adjusted to 7.7 at 4 °C using 0.1 M NaOH) and with 5 CV of SP Wash Buffer II (10 mM HEPES pH 7.0 at 4 °C, 10% (v/v) glycerol, 0.025% (w/v) DDM, 0.005% (w/v) CHS, 2 mM DTT) to remove the fusion proteins MBP and TrxA. Protein elution was carried out from both SP-columns in six fractions of 1 mL, 1 mL, 2 mL, 2 mL, 1 mL, and 1 mL, respectively, using SP Elution Buffer (10 mM HEPES pH 7.5 at RT, 10% (v/v) glycerol, 500 mM NaCl, 0.015% (w/v) DDM, 0.003% (w/v) CHS, 2 mM DTT). Fractions containing ~0.5–2.1 mg/mL of NTSR1-H4_X_ receptor construct (and having an A_260 nm_/A_280 nm_ ratio ≈ 0.55–0.7) were combined and adjusted with SP Elution Buffer to ~0.7 mg/mL. Protein concentrations were determined by absorbance at 280 nm using a Nanodrop 2000 spectrophotometer (Thermo Fisher Scientific). Purified apo NTSR1-H4_X_ was incubated overnight with 150 µM of either compound **28a** or **30a** (prior to complexation with the apo receptor, the proper ligand amount was desiccated in a tube starting from a stock dissolved in DMSO). Subsequently, 2.4 mL of the receptor-ligand sample was concentrated with a Vivaspin 2 concentrator (100 kDa molecular weight cut-off, Sartorius) to 26 μL for reconstitution into the lipidic cubic phase (LCP).

#### Crystallization of NTSR1-H4_X_ in the lipidic cubic phase

Concentrated ligand-bound NTSR1-H4_X_ (see above) was reconstituted in the lipidic cubic phase (LCP) by mixing the concentrated receptor sample with molten monoolein (Sigma-Aldrich) supplemented with 10% (w/w) cholesterol (Sigma-Aldrich) at a protein-to-lipid ratio of 20:32 (v/v), using the two-syringe method (100 µL syringes, Hamilton). Crystallization trials were carried out at 20 °C in 96-well glass sandwich plates (SWISSCI) with a 120 µm spacer. A Crystal Gryphon LCP crystallization robot (Art Robbins Instruments) was used to dispense 40 nL boli and to cover them with 800 nL of precipitant solution. The plates were immediately sealed with a cover glass and incubated at 20 °C in a Rock Imager 1000 (Formulatrix). Optimized crystals of NTSR1-H4_X_ in the presence of compound **28a** grew in 100 mM Na acetate pH 4.5 (Hampton Research), 350 mM K citrate tribasic (Hampton Research), 30% (v/v) PEG400 (Sigma-Aldrich), and 50 μM **28a**. Optimized crystals of NTSR1-H4_X_ in the presence of compound **30a** grew in 100 mM Na cacodylate pH 5.6 (Hampton Research), 350 mM K citrate tribasic (Hampton Research), 30% (v/v) PEG400 (Sigma-Aldrich), and 50 μM **30a**. Optimized crystals are shown in Supplementary Fig. S15. Crystals were harvested by picking the entire LCP bolus at room temperature with MicroMeshes (MiTeGen) and flash frozen in liquid nitrogen without adding further cryoprotectant.

#### Data collection, structure determination, and structural analysis

X-ray data were recorded from NTSR1–H4_x_ crystals, obtained in lipidic cubic phase (LCP) under various conditions in the presence of either compound **28a** or **30a**. The measurements were performed at beamline P13, operated by EMBL Hamburg at the PETRA III storage ring. All data were analyzed with the autoPROC toolbox^103^ that makes use of the data processing package XDS^104^ and the scaling program AIMLESS^105^. The best LCP crystals diffracted to 3.1 Å resolution and suffered from substantial anisotropy, which we corrected with the program STARANISO^106^. The structures were determined by molecular replacement using the program PHASER^107^ with the coordinates of NTS_1_R–H4_x_ of an earlier published NTS_8–13_ complex (PDB ID: 6YVR)^24^. Refinement of the polypeptide chains of NTSR1–H4_x_ with REFMAC5^108^ and model building in Coot^109^ unequivocally revealed the binding of compounds **28a** and **30a**, respectively. As the ratio of unique reflections to parameters to be refined was below 1, we used ISOLDE^110^ within ChimeraX^111^, which enabled flexible fitting in conjunction with molecular dynamics. Both complex structures were finalized with one round of REFMAC5^108^ refinement and were validated with the help of the MolProbity^112^ module within the Phenix program package^113^. For detailed data collection and refinement statistics see Supplementary Table S7.

### In vivo **efficacy study**

#### Animal housing and acclimatization

Adult male Sprague-Dawley rats (250−400 g, 8–10 weeks; Charles River Laboratories, St-Constant, Québec, Canada) were housed in groups of four in transparent, closed cages containing aspen bedding, under control environment conditions (12 h light/dark cycle, 22 °C and 50% humidity) with ad libitum access to food and water. Prior to behavioral testing, rats were acclimatized for three days to handling and to the testing devices in a quiet, dedicated housing room. All behavioral experiments were performed in the same quiet room by both female and male experimenters to minimize stress and reduce potential experimenter-related bias.

#### Formalin pain test

The analgesic efficacy of each compound was evaluated using the formalin-induced tonic pain model^59^. Animals were randomly assigned to treatment groups, and all experiments were conducted by two experimenters blinded to treatment conditions. Compounds were resuspended in vehicle composed of 16% DMSO, 42% water and 42% lipid-based buffer (0.09 M sodium carbonate, 0.25% N,N-dimethylacetamide, 0.25% Kolliphor EL). Each small molecule was administered via intrathecal (i.t.) injection at a dose of 1200 nmol/kg. Five minutes later, rats received a 50 μl intraplantar injection of diluted 2% formaldehyde (equivalent to 5% formalin; Bioshop, Burlington, Canada) into the plantar surface of the right hind paw. Immediately following formalin injection, rats were placed individually into transparent Plexiglas chambers (30 × 30 × 30 cm) positioned over a mirror angled at 45°, allowing unobstructed visualization of the paws. Spontaneous nocifensive behaviors were recorded continuously for 60 min. Intraplantar injection of formalin produced the characteristic biphasic nocifensive response, consisting of an acute phase (0−9 min post-injection), reflecting direct activation of peripheral nociceptors and a prolonged inflammatory phase (21−60 min post-injection), associated with central sensitization.

Nociceptive behaviors were quantified using a weighted pain score^59^. Behaviors were scored in 3-min blocks during 60 min by measuring the time spent in each of four behavioral categories: 0, injected paw comparable to the contralateral paw; 1, reduced weight bearing on the injected paw; 2, elevation of the injected paw without surface contact; 3, licking, biting, or shaking of the injected paw. Higher scores corresponded to greater pain intensity. A weighted pain score, ranging from 0 to 3, was calculated for each time interval using the following formula (1 × T1 + 2 × T2 + 3 × T3)/180, where T1, T2, and T3 represent the time (in seconds) spent in behavioral categories 1, 2, and 3, respectively, during each 180-s interval. Data were analyzed and plotted using GraphPad Prism v10.3. Areas under the curve (AUCs) were calculated for each rat. Group comparisons for bar graph representation were performed using one-way ANOVA followed by Dunnett’s multiple comparisons test. All error bars represent mean ± SEM.

#### Tail-flick test

Acute thermal nociception was assessed using the tail-flick test, a well-established assay for evaluating pain sensitivity and analgesic efficacy. The distal portion of the tail was immersed in a temperature-controlled water bath at 52 °C, and a cut-off latency of 10 s was imposed to prevent tissue damage. Tail-flick latency, defined as the time elapsed between tail immersion and rapid tail withdrawal, was used as the index of nociceptive sensitivity. Prior to testing, animals were individually acclimatized to handling and to the behavioral apparatus for 5 min per day over three consecutive days. On the test day, baseline tail-flick latencies were recorded before drug injection. Under light anesthesia, compound 28a was administered intrathecally at a dose of 1200 nmol/kg, either alone or in combination with a well-characterized non-selective NTSR antagonist (**SR142948A**^20,60^) or an NTS_2_R-selective antagonist (**NTRC844**^58,61^). **SR142948A** (5000 nmol/kg) was prepared in a vehicle containing 6% DMSO, 47% water and 47% lipid-based buffer (0.09 M sodium carbonate, 0.25% N,N- dimethylacetamide, 0.25% Kolliphor EL). **NTRC844** (5000 nmol/kg) was dissolved in 45% DMSO, 45% water, and 10% Kolliphor EL. Antagonists were administered intrathecally 10 min prior to compound **28a**.

Thermal nociception was assessed every 10 min for up to 60 min following i.t. administration of compound or vehicle. Data were analyzed and plotted using GraphPad Prism 10.6 and are expressed as mean ± SEM (*n* = 6–7 animals per group). Time-course data were analyzed using two-way ANOVA followed by Dunnett’s multiple comparisons test. Areas under the curve, (AUCs; 0-60 min), were calculated for each animal and analyzed using one-way ANOVA test followed by Dunnett’s multiple comparisons test.

#### Blood pressure test

Rats were anesthetized with ketamine/xylazine (87 mg/kg: 13 mg/kg, i.p.) and placed in supine position on a heating pad. Mean arterial blood pressure (ΔMABP) was measured through a catheter (PE 50 filled with heparinized saline) inserted in the right carotid artery and connected to a Micro-Med transducer (model TDX-300, USA) linked to a blood pressure Micro-Med analyzer (model BPA-100c). Another catheter (PE 10 filled with heparinized saline) was inserted into the left jugular vein for bolus injection of 0.9% vehicle or each compound to be tested. Blood pressure was recorded each second for up to 300 s following intravenous injection. Blood pressure data were plotted using GraphPad Prism 10.3.

### In vivo **pharmacokinetics study**

#### Plasma, CSF and brain tissue sample collection

Twelve male Sprague Dawley rats (Charles River), with mean body weight of 250 g, received a single intravenous (i.v.) dose of compound **28a** at 5 mg/kg. For animal IDs 1-6, plasma, brain, and cerebrospinal fluid (CSF) were collected at a single time point after dosing, i.e., either at 2 h (animals 1–3) or at 6 h (animals 4-6). For animal IDs 7-12, serial plasma samples were collected at 5, 15, and 30 min, and 1, 2, 4, 6, and 24 h post-dose, followed by terminal collection of brain and CSF. Terminal brain and CSF samples were therefore obtained at 2 h (animals 1–3), 6 h (animals 4–6), and 24 h (animals 7–12) after dosing. Plasma, brain, and CSF samples were collected into Eppendorf® Safe-Lock microcentrifuge tubes (0.5 mL and 1.5 mL, polypropylene, colorless, low-binding surface; Eppendorf), and stored frozen until further processing. For administration, compound **28a** was formulated in a vehicle of DMSO, lipidic buffer, and water (1:4:5, v/v/v). The lipidic buffer contained 0.09 M sodium carbonate, 0.25% N,N-dimethylacetamide, and 0.25% Kolliphor EL.

Chemicals and materials. The experiments were performed using a 10 mM dimethyl sulfoxide (DMSO) stock solution of compound 28a. The internal standards carbutamide (BCS, CAS no. 339-43-5) and warfarin (cat no A2250) were purchased from Sigma-Aldrich. Acetonitrile (AcN, LiChrosolv., LC-grade) and methanol (MeOH; LiChrosolv®, mass spectrometry grade) were purchased from Merck. Formic acid (FA; HiPerSolv Chromanorm®) was purchased from VWR. MilliQ water (mQ-H2O; Milli-Q® SQ 200 P Ultrapure Water Dispensing Module (ZSQ200UPT0, SQPAK™ Quanta polishing cartridge), 18.2 MΩ·cm at 25 °C, Millipore) was used for preparation of mobile phase A and isotonic phosphate buffer. The isotonic phosphate buffer (67 mM, pH 7.4) was prepared in house by dissolving Na_2_HPO_4_·2H_2_O (Sigma-Aldrich, Cas No: 10028-24-7), KH_2_PO_4_ (Sigma-Aldrich, Cas No. 7778-77-0), and NaCl (Merck, Cas No. 7647-14-5) in MilliQ water. Kolliphor was purchased from Sigma-Aldrich (Cas No. 61791-12-6).

Sample preparation for bioanalysis. Brain tissue representing the whole brain was weighed in Precellys® Hard Tissue Homogenizing CK28 tubes (2 mL, polypropylene co‑polymer) containing inert 2.8 mm zirconium oxide beads. For each mg of tissue, 3 × 1.03 µL of 0.1 % formic acid in AcN:MeOH (1:1, v/v) was added (1:3, w:v) to aid homogenization. The tissue was homogenized for 30 s at 5000 rpm with a Minilys, centrifuged at 7000 rpm for 15 min, and the supernatant was collected. To prevent detector saturation, the dosing solution was diluted 300‑fold in plasma (1:299, v:v). High‑concentration plasma samples (5‑ and 15‑min samples from animals 7–12 and the 240‑min sample from animal 7) were diluted 10‑fold (5:45, v:v) before preparation. Twenty µL of undiluted and diluted plasma, brain homogenate supernatant, undiluted CSF, and diluted dosing solution were precipitated with 180 µL acetonitrile containing 100 nM carbutamide and 100 nM warfarin as internal standards. After centrifugation for 20 min at 2465 × *g* at 4 °C, the supernatant was transferred to a Corning® 96‑well storage microplate (round‑bottom, clear polypropylene, non‑sterile; product no. 3365) and injected onto the column.

Plasma standards, quality control (QC), and blank samples were prepared from pooled human plasma from two non‑smoking donors (citric acid anticoagulant, Uppsala Academic Hospital). Blank, standards, and QCs for brain homogenate were prepared from one compound‑naïve rat brain. Due to limited compound‑naïve CSF, CSF samples were quantified using standards and QCs prepared in isotonic phosphate buffer (67 mM, pH 7.4). Calibration standards and QCs were generated by serial dilution of a 10 mM stock solution and intermediate solutions in their respective matrices. The dosing solution was quantified using a single‑point calibration standard prepared from the 10 mM stock in DMSO.

Standard curves were linear across 100–10,000 nM (undiluted plasma), 1000–10,000 nM (diluted plasma), 1–50 nM (brain homogenate), and 2–50 nM (CSF). Each curve included at least five concentration levels meeting the acceptance criteria (precision and accuracy ≤15%, except ± 20% at the lower limit of quantification, LLOQ). LLOQs were 100 nM (undiluted plasma), 1000 nM (diluted plasma), 1 nM (brain homogenate), and 2 nM (CSF). At the LLOQ and higher, analyte peak areas were more than five times the blank response, fulfilling the ≥ 5:1 signal‑to‑noise requirement. Concentrations measured in diluted samples were corrected by multiplying by the dilution factor (e.g., four for brain samples) to obtain undiluted concentrations.

#### Liquid chromatography and mass spectrometry (UPLC-MSMS) conditions

An Acquity UPLC system connected to a triple quadrupole mass spectrometer (XevoTM TQ-S micro, Waters Corp.) was used to inject aliquots of the processed samples onto an ACQUITY UPLC BEH C8 column (1.7 µm particle size, 2.1 × 50 mm; Waters), kept at 60 °C. For chromatographic separation a total running time of 2 min and the following gradient (time/% B): 0/1; 0.6/1; 1.4/99; 1.8/99; 1.9/1; 2/99, were applied. Mobile phases consisted of A) 0.1% formic acid in MilliQ water, and B) 0.1% formic acid in acetonitrile. The flow rate was set to 0.5 mL/min. The injection volume for in vivo samples was 5 µL, and for the dosing solution 2 µL. Electrospray (ESI+) conditions of the mass spectrometer operating in multiple reaction monitoring (MRM) mode were set to: capillary voltage, 3 kV; desolvation gas temperature, 500 °C, and desolvation gas flow, 1000 L/h. MRM transitions are specified in Supplementary Table S13.

#### Pharmacokinetic parameter estimation

Pharmacokinetic parameters were estimated by non-compartmental analysis (NCA). Total plasma concentration-time data were used to calculate the area under the concentration-time curve (AUC) from 0 to 6 h (AUC_0-6h_) by the linear trapezoidal method. AUC was extrapolated to infinity (AUC_0-inf_) by adding AUC_rest_, *i.e*., the ratio of the last measured concentration to the terminal elimination rate constant. The terminal elimination rate constant was estimated by log-linear regression of the final three sampling points (2–6 h), while the distribution-phase rate constant was estimated from the first three sampling points (5–30 min). The initial concentration (C_0_) was obtained by back-extrapolation using the distribution-phase slope. The terminal half-life (*t*_1/2_) was calculated based on the terminal elimination rate constant. Clearance (CL) was calculated based on dose and AUC_0-inf_, and the central volume of distribution (V_c_) was estimated using dose and C_0_, and the apparent volume of distributions (V_z_ and V_ss_) were estimated based on AUC, dose, and the terminal elimination rate constant. Three plasma samples were identified as outliers using the ROUT method (GraphPad Prism version 10.4.2 for Windows, GraphPad Software, San Diego, California USA, www.graphpad.com) and were excluded from the analysis. Estimations normalized to body weight was based on the average body weight of 250 g.

### Reporting summary

Further information on research design is available in the Nature Portfolio Reporting Summary linked to this article.

## Supplementary information

Supplementary Information

Reporting Summary

Transparent Peer Review file

## Source data

Source Data

## Data Availability

The ZINC15 library is available at https://zinc15.docking.org. The PDB accession code for the crystal structure used for molecular docking calculations is 6Z8A. All compounds tested are listed in the Supplementary Information and Source Data File. Chemical identities, purities (LC/MS), yields and spectroscopic analysis (^1^H,^13^C NMR) for selected active compounds are provided in the Supplementary Information. The crystallographic data generated in this study have been deposited in the PDB under accession codes 9QC1 (compound **28a**) and 9QD4 (compound **30a**). The AANCHOR database is available on Zenodo (10.5281/zenodo.15094801)^114^. Source data are provided with this paper.
