# Supplementary material for: Docking of virtual libraries identifies small-molecule agonists of neurotensin receptors with analgesic activity
Source: Nat Commun. 2026 Jul 22;17:6970. doi: 10.1038/s41467-026-74990-1 (PMC13392031; doi:10.1038/s41467-026-74990-1)
Supplement: Supplementary file 1 — Supplementary Information [file 41467_2026_74990_MOESM1_ESM.pdf]

Supplementary Information

Docking of virtual libraries identifies small-molecule agonists of neurotensin receptors with analgesic activity

Nicolas Panel[1,†], Duy Duc Vo[1,2,†,*], Harald Hübner[3,†], Mattia Deluigi[4], Szymon Pach[1], Félix Bélair[5], Dorothee Weikert[3,6], Christoph Klenk[4,7], Mark Hilge[4], Niharika Shiva[8], Isabelle Brochu[5], Jean-Michel Longpré[5], Frida Bällgren[9], Aljona Saleh[9], Huabin Hu[1], Jon Kapla[1], Stefanie Kampen[1], Israel Cabeza de Vaca[1], Jan Kihlberg[2], Nina Wettschureck[8,10], Philippe Sarret[5,*], Andreas Plückthun[4,*], Peter Gmeiner[3,6,*], Jens Carlsson[1,*]

[1]Science for Life Laboratory, Department of Cell and Molecular Biology, Uppsala University, SE-751 24 Uppsala, Sweden.

[2]Department of Chemistry-BMC, Uppsala University, SE-751 23, Uppsala, Sweden.

[3]Department of Chemistry and Pharmacy, Medicinal Chemistry, Friedrich-Alexander-Universität Erlangen-Nürnberg, Nikolaus-Fiebiger-Straße 10, 91058 Erlangen, Germany.

[4]Department of Biochemistry, University of Zurich, Winterthurerstrasse 190, CH-8057 Zürich, Switzerland.

[5]Department of Pharmacology-Physiology, Faculty of Medicine and Health Sciences, Institute de Pharmacologie de Sherbrooke, Université de Sherbrooke, Sherbrooke, Quebec, Canada.

[6]FAUNeW – Research Center New Bioactive Compounds, Friedrich-Alexander-Universität Erlangen-Nürnberg, Nikolaus-Fiebiger-Str. 10, 91058 Erlangen, Germany.

[7]Rudolf Buchheim Institute of Pharmacology, Justus Liebig University, Giessen, Germany.

[8]Department of Pharmacology, Max Planck Institute for Heart and Lung Research, Bad Nauheim, Germany.

[9]Department of Pharmacy, SciLifeLab Drug Discovery and Development, Uppsala University, SE-751 23 Uppsala, Sweden.

[10]Centre for Molecular Medicine, Medical Faculty, Goethe University, Frankfurt am Main, Germany.

[†]Contributed equally to this work

[*]To whom correspondence should be addressed: jens.carlsson@icm.uu.se, peter.gmeiner@fau.de, plueckthun@bioc.uzh.ch, duc.duy.vo@kemi.uu.se, philippe.sarret@usherbrooke.ca

Table of contents

Supplementary figures

Figure S1. Functional screening of designed compounds at three concentrations S3
Figure S2. Concentration-response curves of virtual screening hits S4
Figure S3. Structure-activity relationships for compound **25** S5
Figure S4. Extended *in vitro* biological activity of compounds **28a**, **30a**, and **35a** S6
Figure S5. Double conformation of tetrazole-thiophene moiety in the compound **28a** complex S7
Figure S6. IP_1 response of cells expressing $rNTS_1R$ to **28a** and **30a** S8
Figure S7. Molecular determinants for high-affinity binding of $NTS_{8–13}$ to NTS_1R compared to **28a** and **30a** S9
Figure S8. Agonist-induced IP_1 formation in digested murine left-ventricular heart tissue harvested from wild-type mice or NTS_2R-deficient mice S10
Figure S9. Effects of compounds **28a**, **30a**, and **35a** on blood pressure S11
Figure S10. Mean total plasma concentration-time profile of compound **28a** following a 5 mg/kg intravenous dose in rats S12
Figure S11. Ligands for peptide-binding GPCRs mimicking the C- or N-terminal residue of the endogenous peptide S13
Figure S12. Novel GPCR targets for virtual screening approach S14
Figure S13. Detailed description of the AANCHOR database generation S15
Figure S14. NTSR1-$H4_X$ crystallization construct and fusion of DARPin D12 to rNTSR1-H4 S16
Figure S15. Crystals of NTSR1-$H4_X$ in the presence of compounds **28a** and **30a** grown in LCP S17

Supplementary tables

Table S1. Docking ranks and functional activities of compounds from the diverse library for $hNTS_1R$ S18
Table S2. Docking ranks and functional activities of compounds from the focused library for $hNTS_1R$ S20
Table S3. Functional activities of compound **24** analogs for $hNTS_1R$ S22
Table S4. Functional activities of compound **25** analogs for $hNTS_1R$ S27
Table S5. Physicochemical properties and similarity to reference agonists of compounds **28a**, **30a**, and **35a** S35
Table S6. Affinities of compounds for thermostabilized variant NTSR1-H4 and wild-type $rNTS_1R$ S36
Table S7. Final data collection statistics S37
Table S8. Affinities of compounds **28a**, **30a**, and **35a** for $rNTS_2R$ S38
Table S9. Affinities of compounds **28a**, **30a**, and **35a** for human GPCRs S39
Table S10. Concentration of compound **28a** in brain tissue and CSF samples from pharmacokinetic study in rats S40
Table S11. Vendor and catalog identifiers for commercially purchased compounds S41
Table S12. Mutations of rNTSR1-H4 compared to wild-type $rNTS_1R$ S42
Table S13. The LC-MS/MS multiple reaction monitoring (MRM) transitions used for quantification of *in vivo* samples S43

Supplementary methods: Chemistry

General synthetic procedures S44
Synthesis of compounds selected from virtual screening **24-40** S45
Optimization of compound **24** (Scaffold a) S46
Optimization of compound **25** (Scaffold b) S50
Analytical data of synthesized compounds S53

Supplementary references S218

Supplementary figures

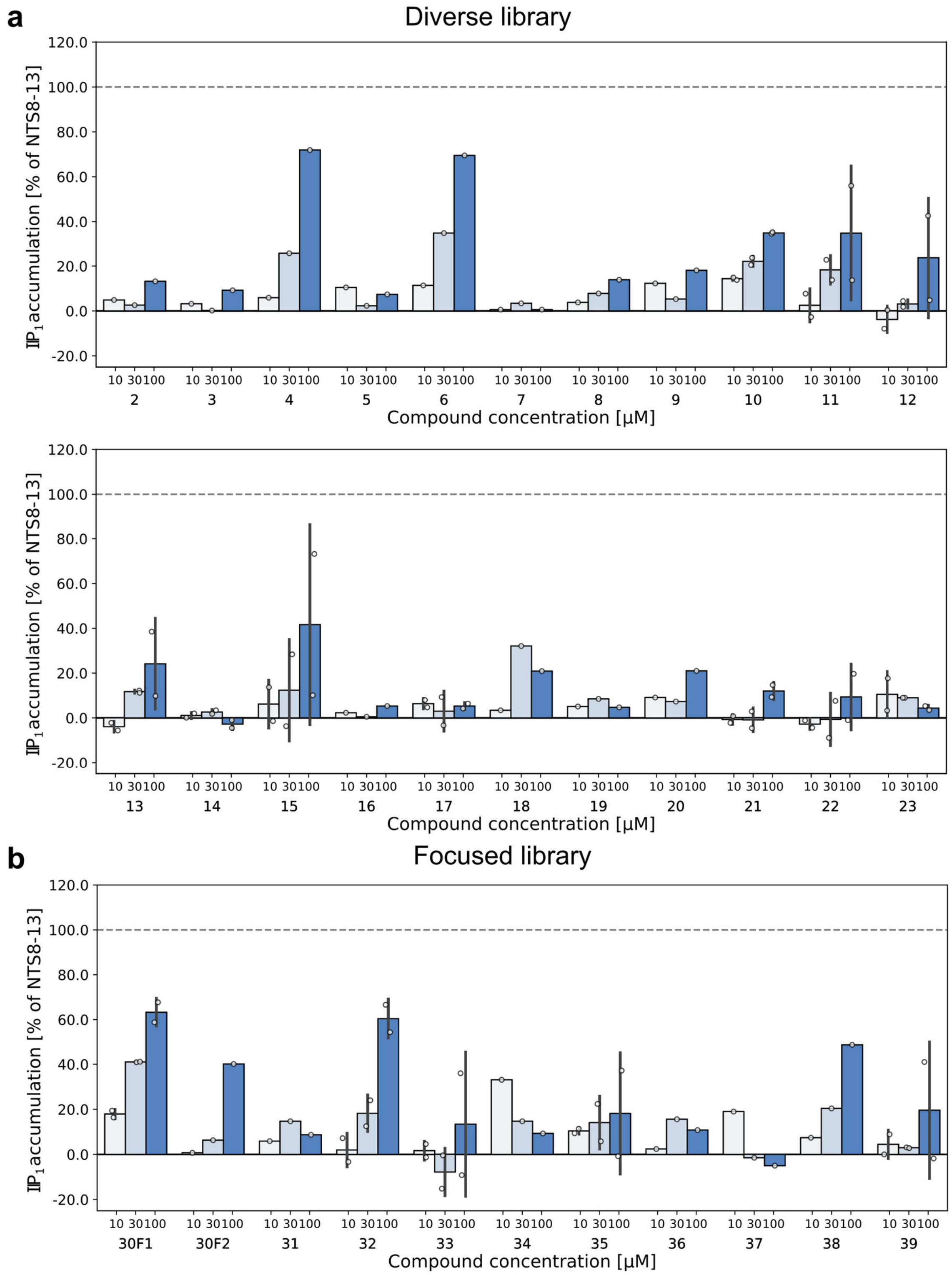

Supplementary Figure S1. Functional screening of designed compounds at three concentrations. G_q-promoted activity at $hNTS_1R$ was evaluated by IP_1 accumulation at 10, 30 and 100 µM for compounds from the (a) diverse and (b) focused libraries. Error bars correspond to the standard deviation calculated from two independent experiments (n=2). The activity of compounds with no error bar was measured in one experiment (n=1). Source data are provided as a Source Data file.

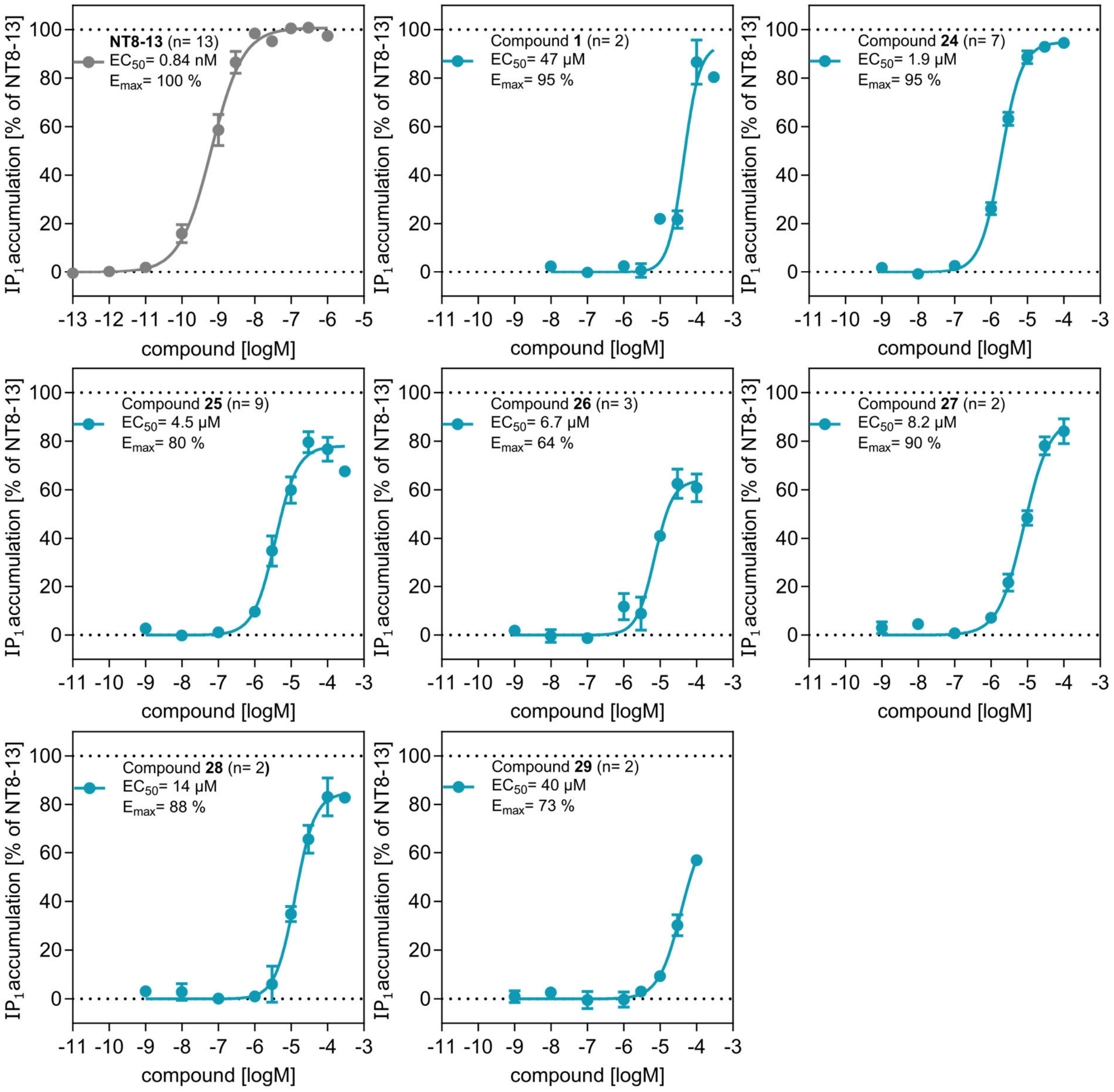

Supplementary Figure S2. Concentration-response curves of virtual screening hits. $hNTS_1R$ activation was measured in an IP_1 accumulation assay. Data indicate mean ± SEM of n independent experiments. Source data are provided as a Source Data file.

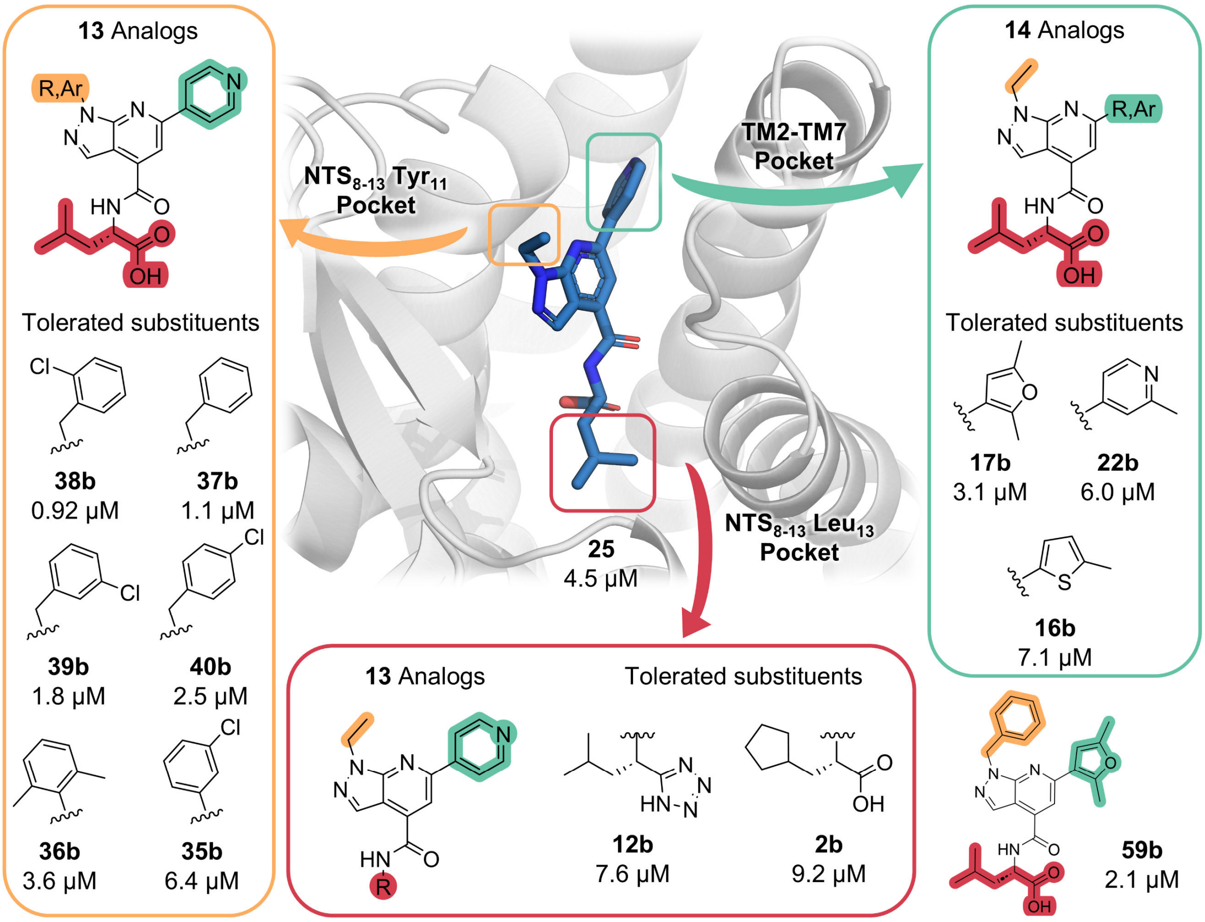

Supplementary Figure S3. Structure-activity relationships for compound 25. To improve potency, we first explored substitutions of the leucine by other amino acids and tetrazole derivatives (compounds **1b**-**13b**). Of the 13 synthesized analogs, only two compounds displayed potencies comparable to compound **25** (EC_{50} = 4.5 μM) in the IP_1 accumulation assay (red box: compounds **12b** and **2b** with EC_{50} values of 7.6 and 9.2 μM, respectively). Next, two virtual libraries of 4445 and 5633 analogs substituted at either position 1 or position 6, respectively, were generated from commercially available building blocks. An additional diverse set of 28 analogs with substitutions at both positions 1 and 6 was identified by substructure search in the Enamine REAL Space database.[1] In the second round of optimization, a subset of 39 compounds (13 and 14 molecules substituted at positions 1 and 6, respectively, and 12 molecules with substitutions at both positions) was selected for experimental evaluation. From the 6-(pyridine-4-yl) substitution library, three compounds showed activity in the range of compound **25** (green box: EC_{50} = 3.1-7.1 μM). From the 1-ethyl substitution library, six compounds showed similar or better activity than compound **25** (orange box: EC_{50} = 1.0-3.5 μM). The 1-benzyl substitution appeared to be optimal with a five-fold increase in potency (**38b**, EC_{50} = 0.92 μM). Finally, we combined the most favorable substitution patterns at positions 1 and 6. The substrates for seven combinations of 1 and 6 modified analogs were purchasable. The most potent compound in this series was compound **59b** with an EC_{50} value of 2.1 μM, which has 1-benzyl and 6-(2,5-dimethylfurane-3-yl) substituents. Source data are provided as a Source Data file.

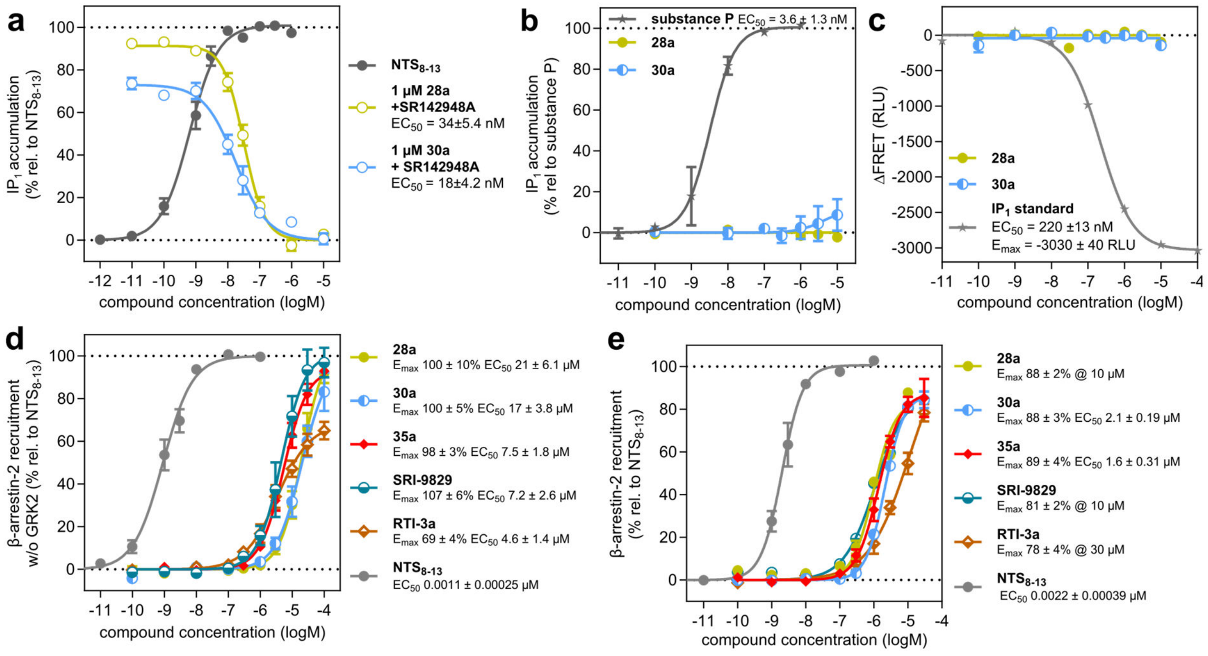

Supplementary Figure S4. Extended *in vitro* biological activity of compounds 28a, 30a, and 35a. **(a)** The NTS_1R antagonist **SR142948A** inhibits the IP_1 accumulation elicited by 1 µM of **28a** (n = 5) or **30a** (n = 3), confirming orthosteric engagement of the compounds. **(b)** In cells transfected with the neurokinin 1 receptor [n = 3 (**30a**, substance P); n = 4 (**28a**)] or **(c)** a mock plasmid [n = 4 (IP_1 standard, **30a**); n = 7 (**28a**)], **28a** and **30a** do not induce IP_1 accumulation, confirming that $hNTS_1R$ mediates their agonistic activity. **(d)** β-arrestin-2 recruitment measured with the PathHunter enzyme fragment complementation assay in the absence of GRK2 shows similar efficacies, but reduced potencies compared to cells overexpressing GRK2 [n = 5 (**SRI-9829**); 6 (**RTI-3a**), 12 (NTS_{8-13}); 7 (all others)]. **(e)** The agonist properties of the small molecules are confirmed in a second readout for β-arrestin-2 recruitment based on BRET between NTS_1R-Rluc8 and β-arrestin-2 mVenus in HEK293 cells that are also co-transfected with GRK2 [n = 4 (**RTI-3a**); 5 (**SRI-9829**, **35a**); 6 (**28a**, **30a**); 7 (NTS_{8-13})]. **(a-e)** Data show mean ± SEM of n independent experiments, each performed in duplicate. Source data are provided as a Source Data file.

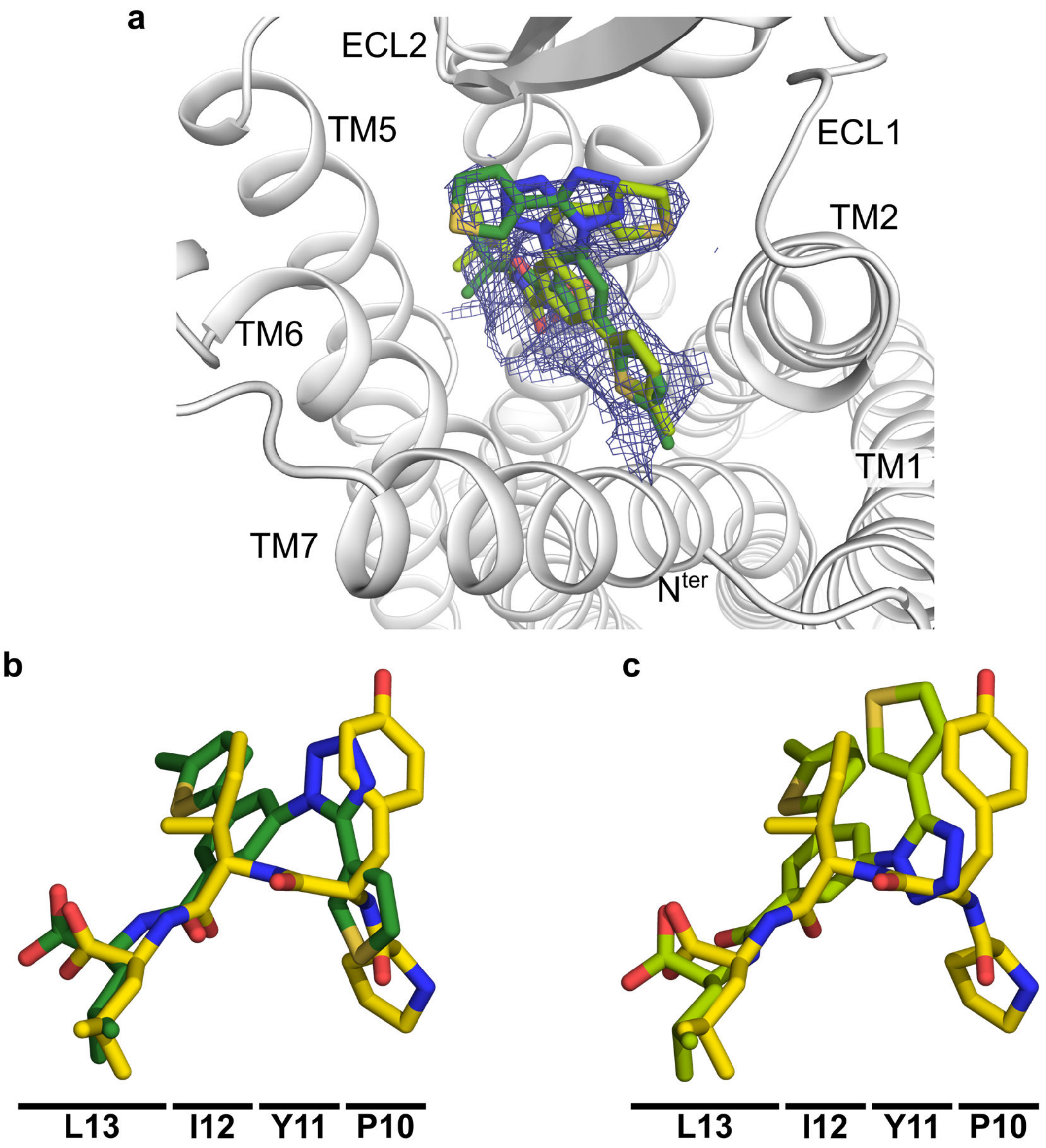

Supplementary Figure S5. Double conformation of tetrazole-thiophene moiety in the compound 28a complex. (a) Hammerhead-shaped electron density in the $2F_o$–F_c map before inclusion of the ligand in the NTSR1-H4$_x$-**28a** complex. Blue chicken wire shows the electron density contoured at 0.8σ with the final model of the double conformation in light and dark green. **(b-c)** Alternative conformations of compound **28a** superimposed onto $NTS_{10–13}$ (yellow).

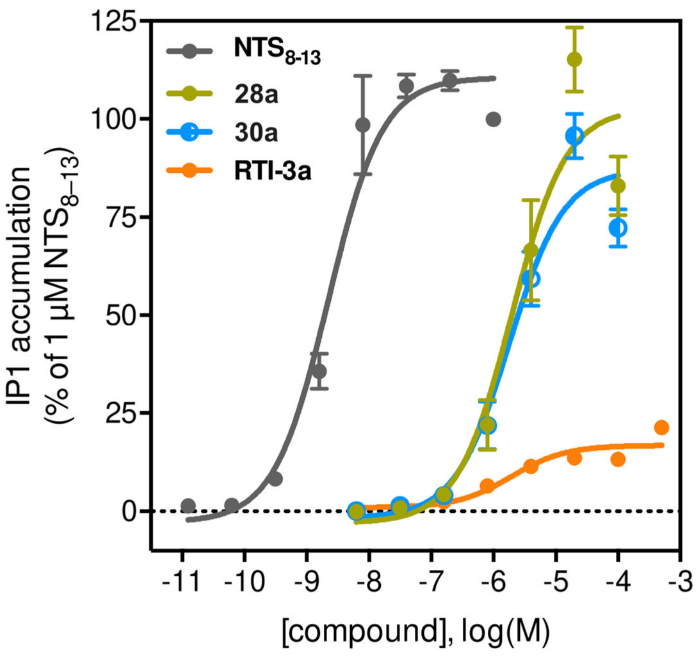

Supplementary Figure S6. IP$_1$ response of cells expressing rNTS$_1$R to compounds 28a and 30a. IP1 accumulation measured in HEK293T cells transiently expressing rNTS$_1$R. Data represent mean values ± SEM of n = 4 independent experiments, each performed in duplicate. Source data are provided as a Source Data file.

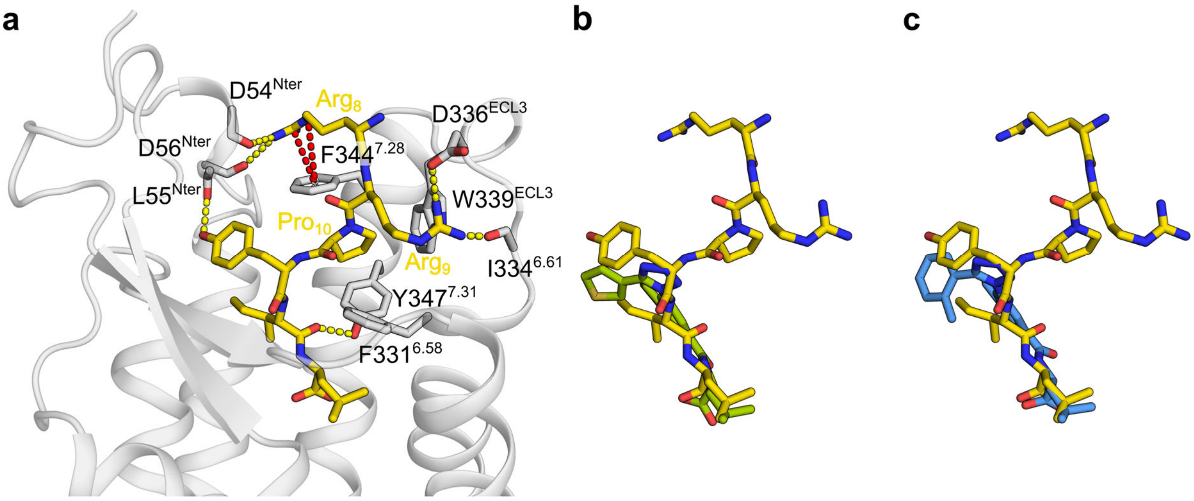

Supplementary Figure S7. Molecular determinants for high-affinity binding of NTS$_{8–13}$ to NTS$_1$R compared to 28a and 30a. (a) Interactions between NTS$_{8–13}$ and NTSR1-H4$_x$. Hydrogen bonds and salt-bridges are shown as yellow dots while cation-π interactions are shown as red dots. **(b–c)** Superpositions of conformer A of compounds **28a** (green) **(b)** and **30a** (blue) **(c)** onto the six C-terminal residues (NTS$_{8–13}$) of the endogenous agonist (yellow). Panels **a**–**c** display identical orientations. The binding affinity of NTS$_{8–13}$ is substantially enhanced by specific interactions of Arg$_8$, Arg$_9$, and Pro$_{10}$. In particular, Arg$_8$ forms a strong cation-π interaction with the phenyl ring of F344$^{7.28}$, while its guanidinium headgroup exhibits hydrogen bonds with the backbone carbonyl oxygens of D54^{Nter} and D56^{Nter}, engaging residues preceding TM1. Arg$_9$ maintains a stacking interaction with its C$^{\beta}$, C$^{\gamma}$, and C$^{\delta}$ atoms and the indole group of W339^{ECL3}, whereas its guanidinium headgroup forms a salt-bridge with D336^{ECL3} and a hydrogen bond with the backbone carbonyl oxygen of I334$^{6.61}$. Finally, Pro$_{10}$ engages in hydrophobic interactions with F331$^{6.58}$, W339^{ECL3}, F344$^{7.28}$, and Y347$^{7.31}$. Together, these interactions of Arg$_8$, Arg$_9$, and Pro$_{10}$ increase the binding affinity of NTS$_{8–13}$ by four to five orders of magnitude compared to compounds **28a** and **30a**.

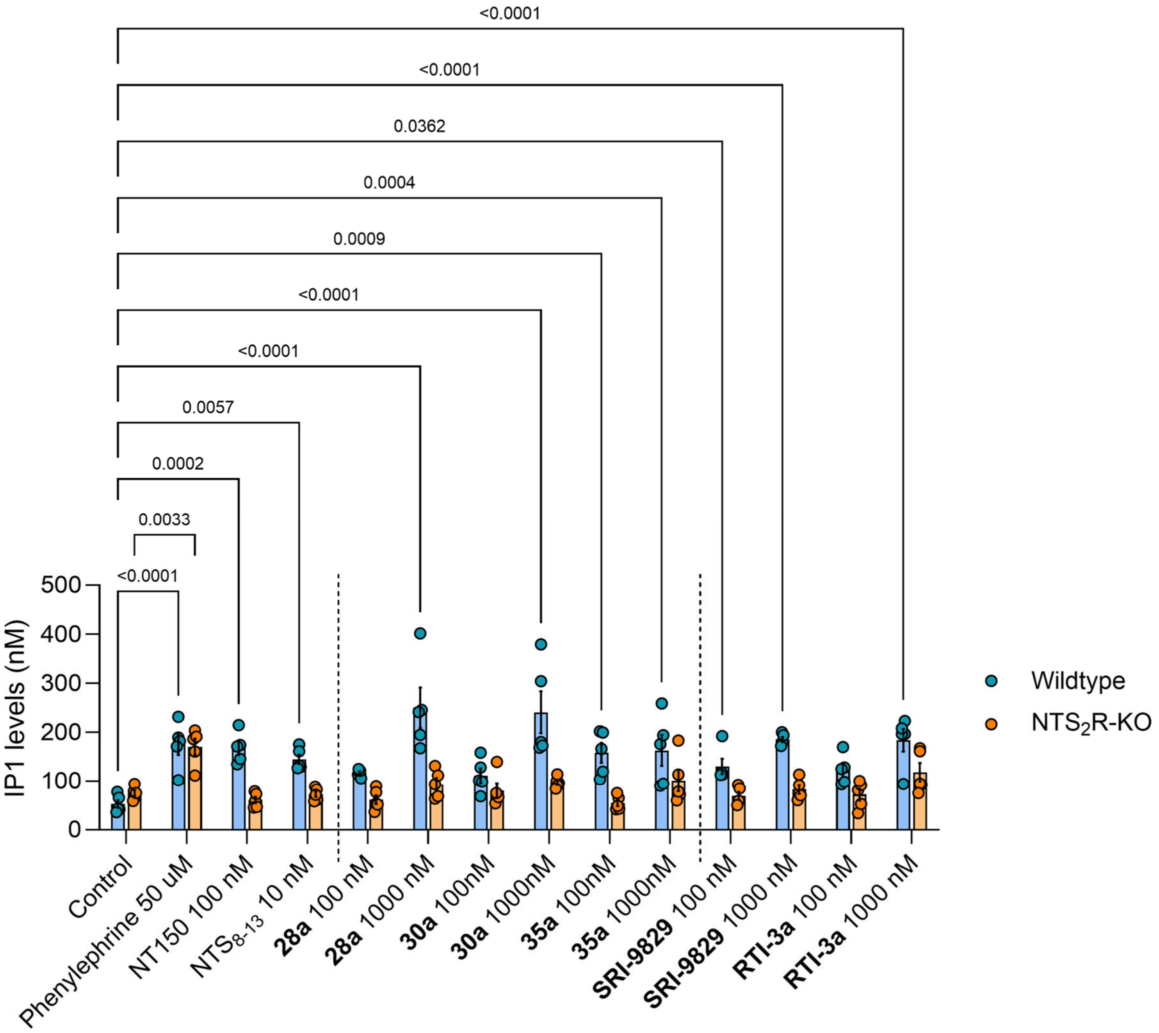

Supplementary Figure S8. Agonist-induced IP_1 formation in digested murine left-ventricular heart tissue harvested from wild-type mice or NTS_2R-deficient mice. Data are shown as means ± SEM and individual data points from independent experiments (n = 5). Comparisons between treatment groups were performed using two-way ANOVA with Sidak’s multiple comparisons test; only P values for significant differences are indicated. Source data are provided as a Source Data file.

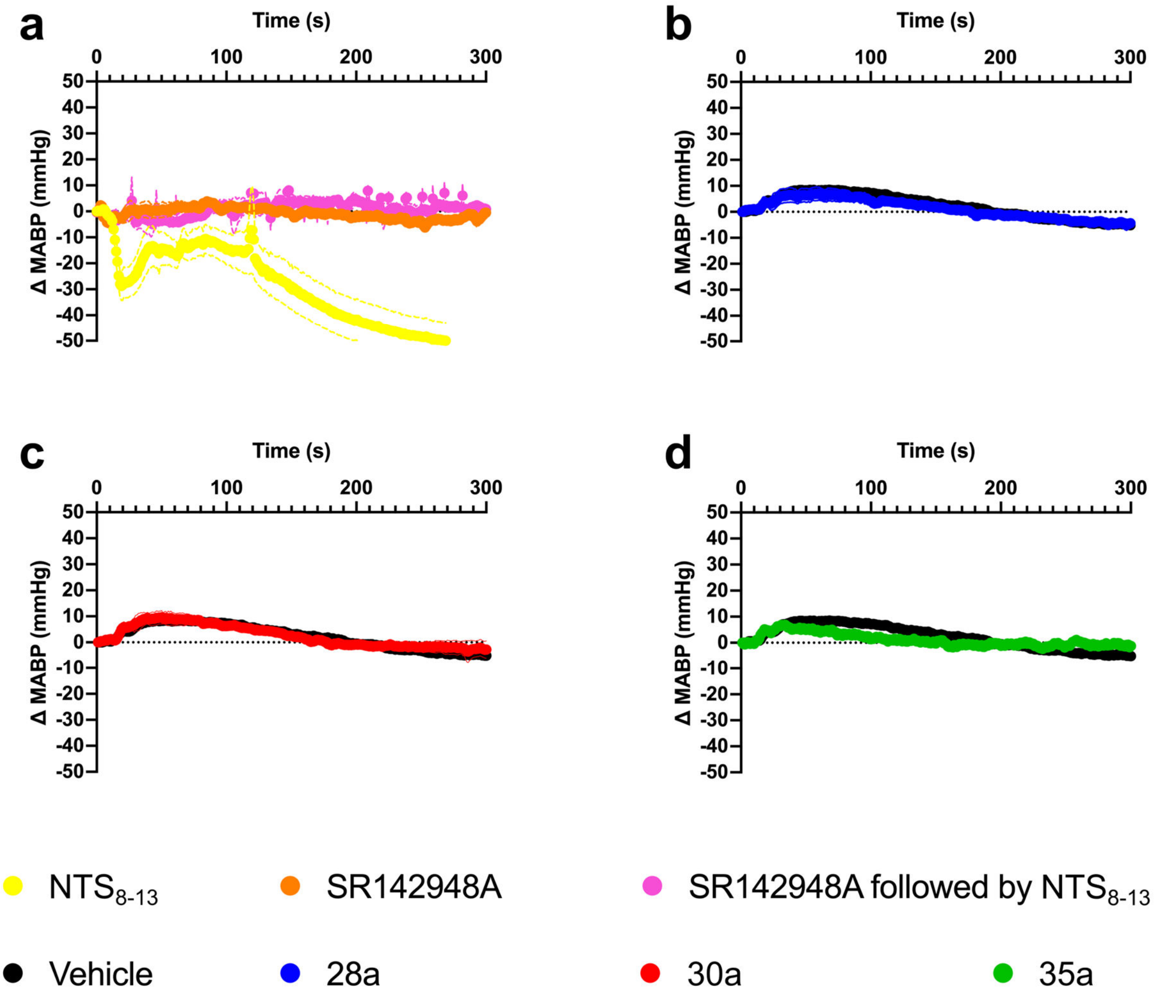

Supplementary Figure S9. Effects of compounds 28a, 30a, and 35a on blood pressure. Delta mean arterial blood pressure (ΔMABP) recorded over 300 s after intravenous (i.v.) injection of vehicle, NTS_{8-13} at 122 nmol/kg or compounds (**28a**, **30a** or **35a**) at 1000 nmol/kg. **(a)** Pretreatment with **SR142948A** (1000 nmol/kg) reduced the ability of NTS_{8-13} to induce hypotension. **(b, c,** and **d)** i.v. injection of **28a**, **30a** or **35a** did not alter ΔMABP, n = 5 (**28a**, **30a**, **35a**); n = 6 (**SR142948A** followed by NTS_{8-13}); n = 7 (NTS_{8-13}, SR142948A, vehicle) independent experiments. Source data are provided as a Source Data file.

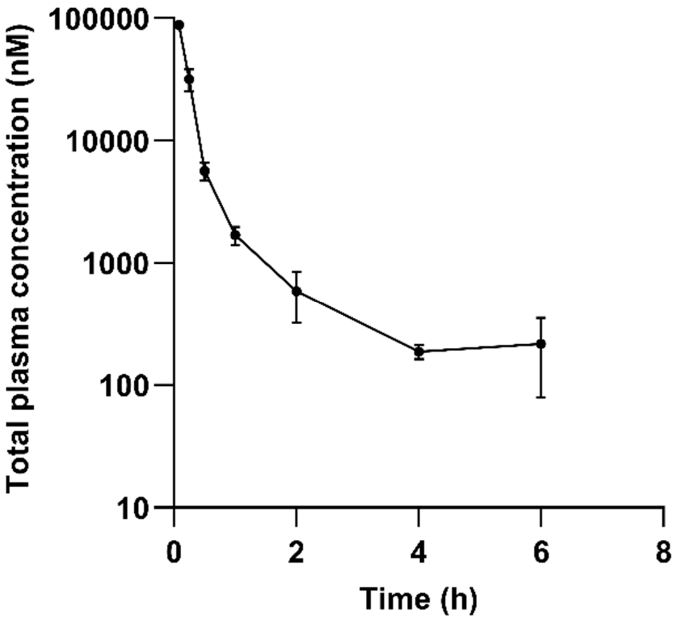

Supplementary Figure S10. **Mean total plasma concentration-time profile of compound 28a following a 5 mg/kg intravenous dose in rats.** Data were obtained from 12 animals and error bars represent the standard deviation, n = 4 (6h); n = 5 (0.5h, 4h); n = 6 (0.08h, 0.25h, 1h); n = 9 (2h) independent experiments. Source data are provided as a Source Data file.

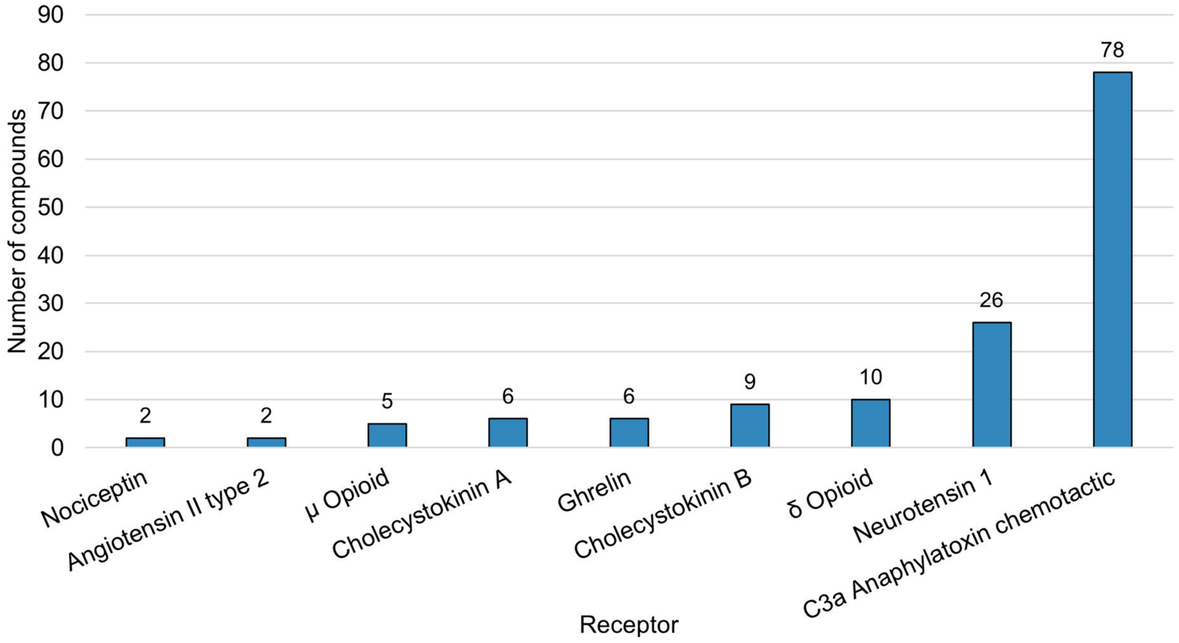

Supplementary Figure S11. Ligands for peptide-binding GPCRs mimicking the C- or N terminal residue of the endogenous peptide. Analysis of 6708 ligands of peptide-binding GPCRs from the ChEMBL database identified 144 non-peptide compounds containing the C- or N-terminal amino acid of the endogenous peptide agonist. These compounds were ligands of nine different GPCRs.

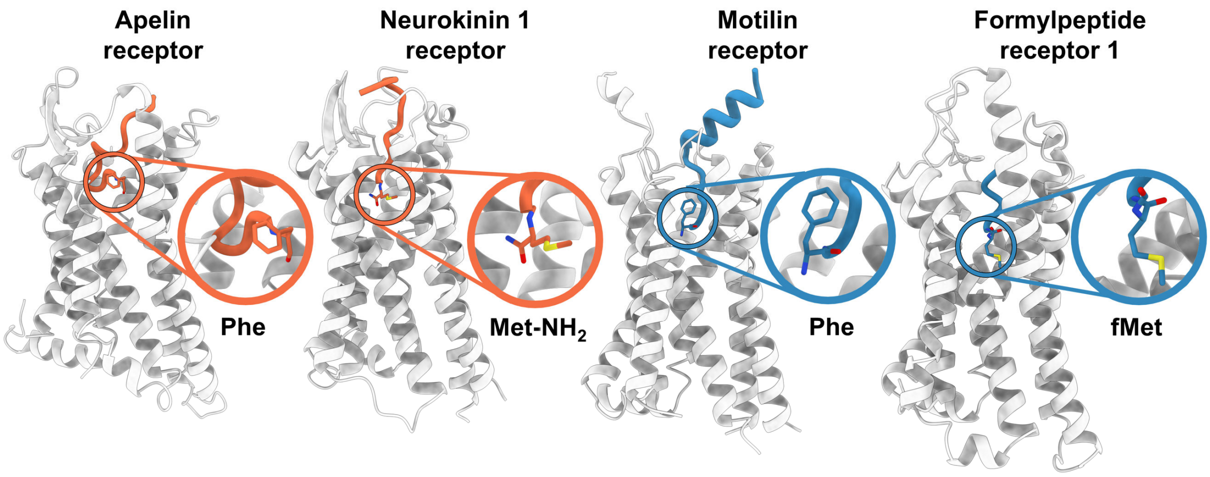

Supplementary Figure S12. Novel GPCR targets for virtual screening approach. Experimental structures of four class A GPCRs in complex with peptides (APLNR[2], NK1R[3], MLNR[4], and FPR1[5] with PDB accession codes 8XZG, 7RMG, 8IBV, and 7T6T, respectively). In each case, the C- or N-terminus is deeply buried in the orthosteric site, providing a suitable anchor for a small molecule ligand. The receptors are shown as cartoons and the peptides anchored with the C- or N-terminal residue are colored in orange and blue, respectively. In these cases, no small-molecule ligands containing the N- or C-terminal residue of the peptide agonist were available in the ChEMBL database[6]. These peptide-binding GPCRs are promising targets for the virtual screening strategy.

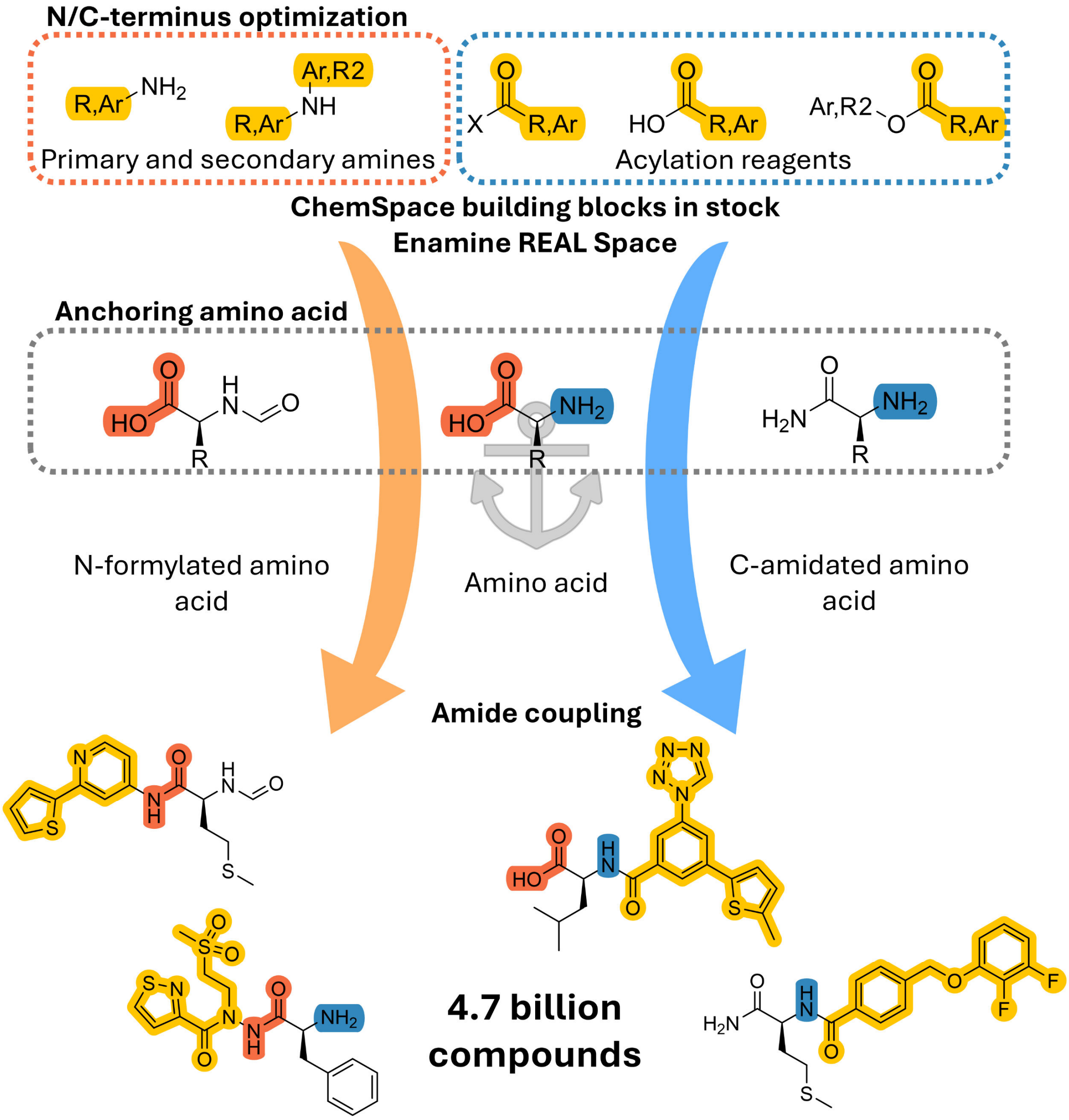

Supplementary Figure S13. Detailed description of the AANCHOR database generation. The in-stock building blocks obtained from ChemSpace and make-on-demand reagents obtained from Enamine REAL Space[1] were coupled via C- and N-termini through amidation. The free carboxy-group of N-formylated building blocks was coupled to primary and secondary amines only. The free alpha-amino group of amino acid amides was coupled with the acylation reagents (acyl halides, carboxylic acids, and esters) only. Free amino acids were coupled either with the free amino or carboxyl-groups. In total, more than 4.7 billion compounds with a heavy atom count lower than 29 were generated.

NTSR1-H4X:

GPGSGPNSDLDVNTDIYSKVLVTAIYLALFVVGTVGNGVTLFTLARKKSLQSLQSRVDYYLGSLALSDL
LILLFALPVDLYNFIWVHHPWAFGDAGCKGYYFLREACTYATALNVVSLSVELYLAICHPFKAKTLMSR
SRTKKFISAIWLASALLAIPMLFTMGLQNLSGDGTHPGGLVCTPIVDTATLRVVIQLNTFMSFLFPMLVA
SILNTVAARRLTVMVHQAAFNMTIEPGRVQALRRGVLVLRAVVIAFVVCWLPYHVRRLMFVYISDEQW
TTALFDFYHYFYMLSNALVYVSAAINPILYNLAEDLVEDWEKARKLLEAARKGQDDEVRILLA
NGADVNTADETGFTPLHLAAWEGHLGIVEVLLKNGADVNANDERGHTPLHLAAYTG
HLEIVEVLLKNGAGVNATDVIGTAPLHLAAMWGHLEIVEVLLKNGADVNAQDKFGKT
PFDLAIDNGNEDIAEVLQKAATRELEVLFQ

b

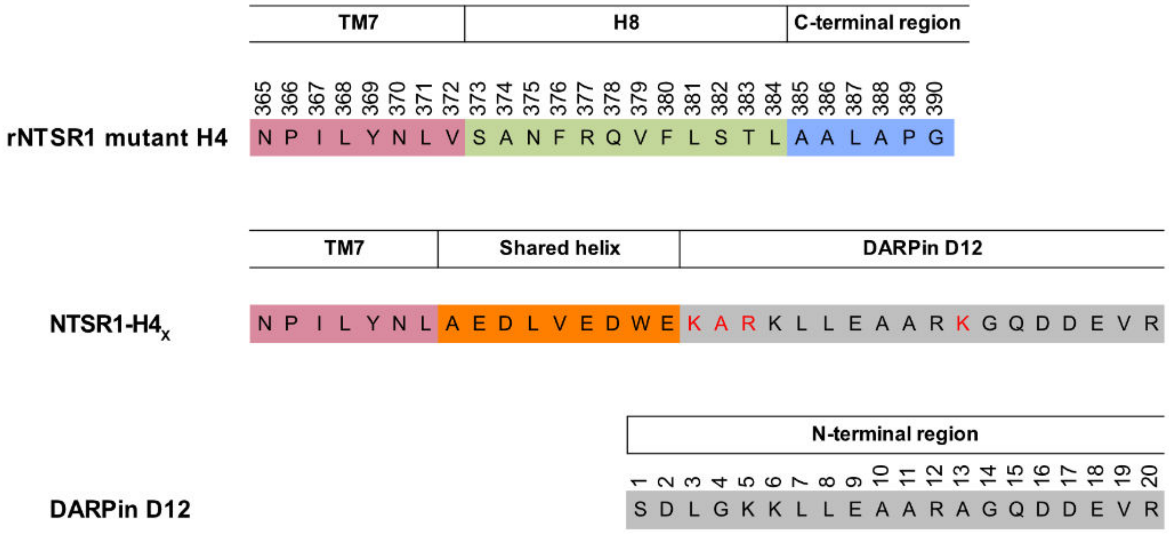

Supplementary Figure S14. NTSR1-H4X crystallization construct and fusion of DARPin D12 to rNTSR1-H4. (a) Amino acid sequence of NTSR1-H4X. The GPCR region is colored in raspberry, DARPin D12 in gray, the shared helix in orange, the cleaved HRV 3C protease cleavage sites in magenta, whereas the short linkers are not colored. The six mutations in DARPin D12 are highlighted in red. **(b)** Fusion site. Top row: C-terminal end of TM7 of rNTSR1-H4 (raspberry), helix 8 (H8, light green), and part of the C-terminal region (light blue). Bottom row: N-terminal region of DARPin D12 (gray). Middle row (aligned with top and bottom sequence): crystallized fusion construct. It is a fusion of the C-terminal end of TM7 (raspberry) via a shared helix (orange) to DARPin D12 (gray). The first two N-terminal residues of DARPin D12 were deleted, and four mutations were introduced in its N-terminal region (highlighted in red).

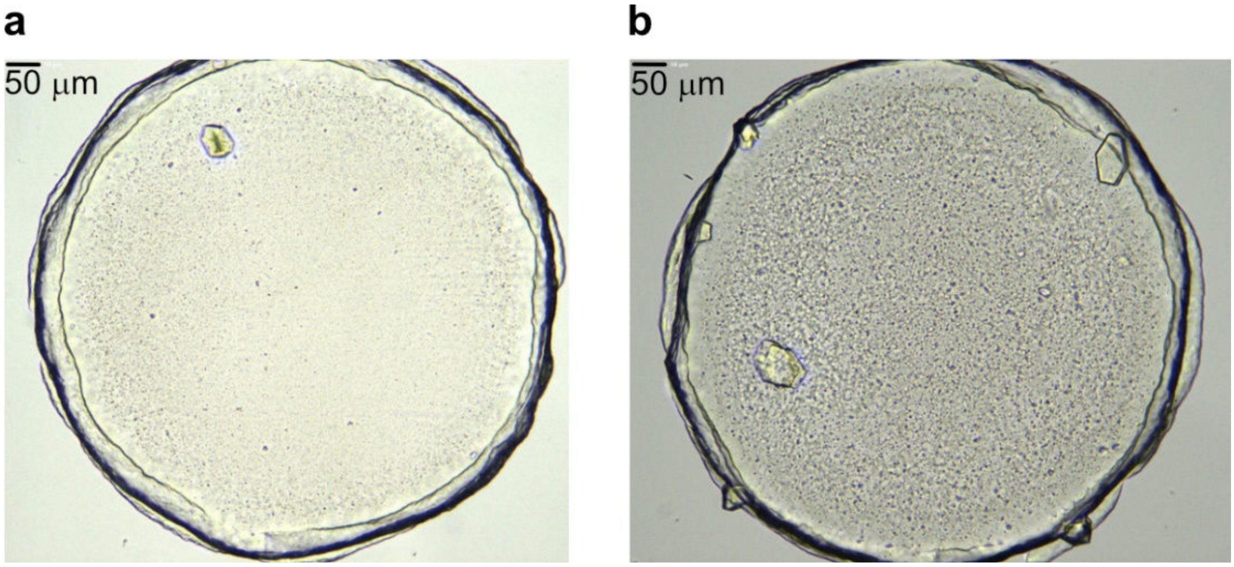

Supplementary Figure S15. Crystals of NTSR1-H4$_X$ in the presence of compounds (a) 28a and (b) 30a grown in LCP.

Supplementary tables

Supplementary Table S1. Docking ranks and functional activities of compounds from the diverse library for $hNTS_1R$. The EC_{50} and E_{max} values were determined for the compounds exhibiting a significant and concentration-dependent response (Figures S1-2).[a] Source data are provided as a Source Data file.

ID	2D structure	Docking Rank	EC_{50} [µM ± SD][b]	E_{max} [% ± SD][c]	n[d]
**1**	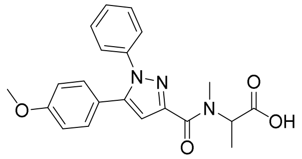	1186	47 ± 5.7	95 ± 3	2
**2**	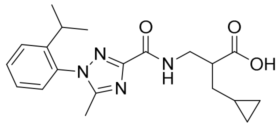	31	n.d[e]	n.d	
**3**	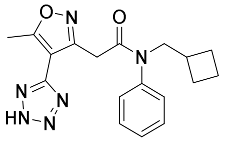	435	n.d	n.d	
**4**	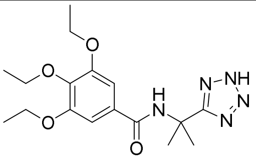	282	n.d	n.d	
**5**	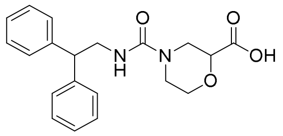	240	n.d	n.d	
**6**	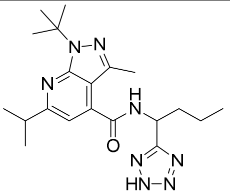	1752	n.d	n.d	
**7**	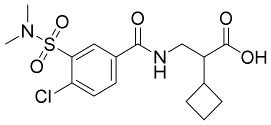	1995	n.d	n.d	
**8**	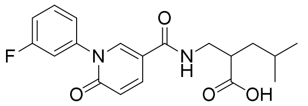	148	n.d	n.d	
**9**	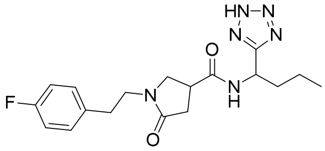	1651	n.d	n.d	
**10**	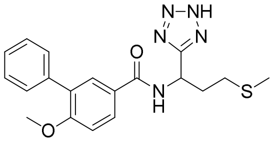	344	n.d	n.d	

**11**	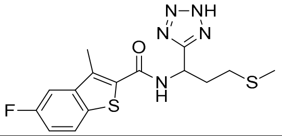	784	n.d	n.d	
**12**	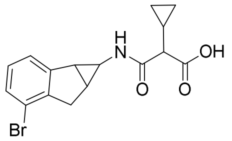	435	n.d	n.d	
**13**	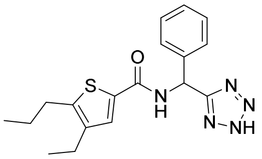	336	n.d	n.d	
**14**	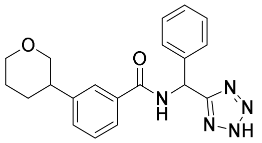	1624	n.d	n.d	
**15**	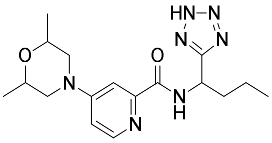	498	n.d	n.d	
**16**	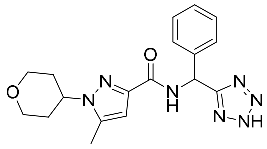	1321	n.d	n.d	
**17**	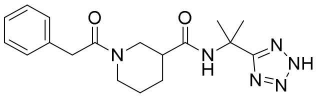	1724	n.d	n.d	
**18**	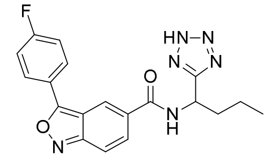	816	n.d	n.d	
**19**	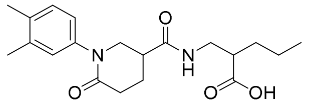	1037	n.d	n.d	
**20**	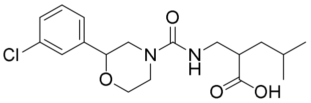	411	n.d	n.d	
**21**	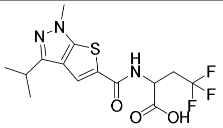	2128	n.d	n.d	
**22**	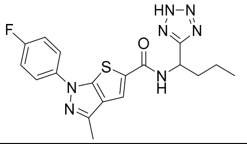	207	n.d	n.d	
**23**	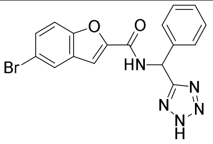	1591	n.d	n.d	

[a] Receptor activation was determined by applying an IP_1 accumulation assay measuring $G\alpha_q$ mediated second messenger accumulation. [b] Potency for $hNTS_1R$ activation in µM ± SD. [c] Maximum efficacy in % ± SD relative to the full effect of NTS8-13. [d] Number of individual experiments, each performed in duplicate. [e] Not determined.

Supplementary Table S2. Docking ranks and functional activities of compounds from the focused library for $hNTS_1R$. The EC_{50} and E_{max} values were determined for the compounds exhibiting a significant and concentration-dependent response (Figure S1-2).[a] Source data are provided as a Source Data file.

ID	2D structure	Docking Rank	EC_{50} [µM ± SD][b]	E_{max} [% ± SD][c]	n[d]
**24**	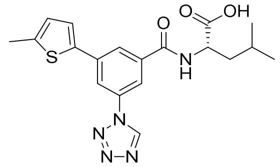	304	1.9 ± 0.47	95 ± 5	7
**25**	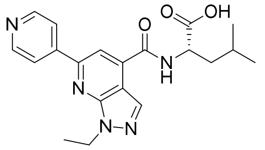	755	4.5 ± 0.90[e]	80 ± 4[f]	9
**26**	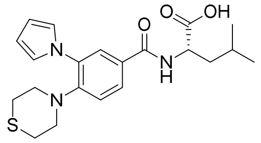	4037	6.7 ± 2.6	64 ± 11	2
**27**	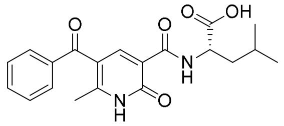	45080	8.2 ± 1.7	90 ± 8	3
**28**	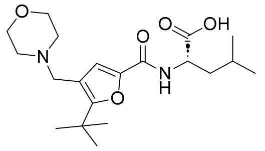	1953	14 ± 1.4	88 ± 11	2
**29**	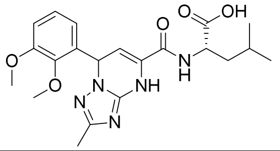	26640	40 ± 12	73 ± 4	2
**30**	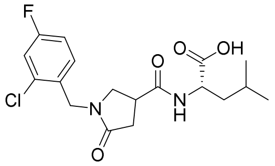	127790	n.d[g]	n.d	
**31**	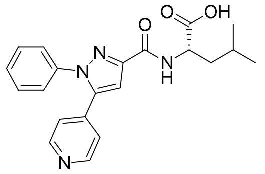	49747	n.d	n.d	
**32**	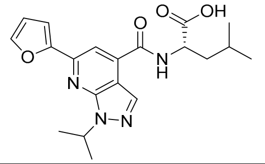	97771	n.d	n.d	
**33**	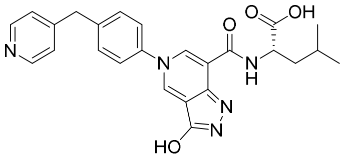	3406	n.d	n.d	

**34**	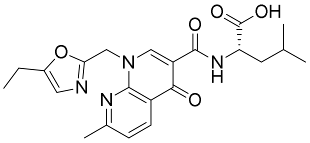	47409	n.d	n.d	
**35**	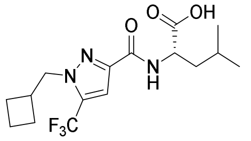	28933	n.d	n.d	
**36**	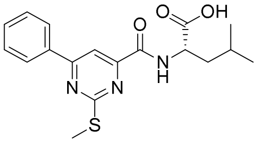	2862	n.d	n.d	
**37**	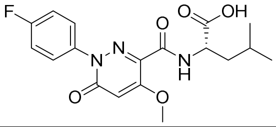	16586	n.d	n.d	
**38**	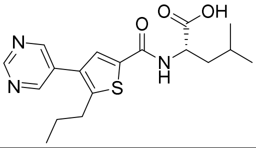	2921	n.d	n.d	
**39**	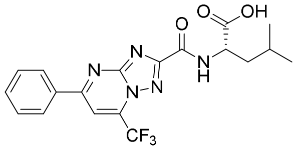	48740	n.d	n.d	

[a] Receptor activation was determined by applying an IP_1 accumulation assay measuring $G\alpha_q$ mediated second messenger accumulation. [b] Potency for $hNTS_1R$ activation in µM ± SD. [c] Maximum efficacy in % ± SD relative to the full effect of NTS8-13. [d] Number of individual experiments, each performed in duplicate. [e] Potency for $hNTS_1R$ activation in µM ± S.E.M. [f] Maximum efficacy in % ± S.E.M. [g] Not determined.

Supplementary Table S3. Functional activities of compound **24** analogs for $hNTS_1R$. The EC_{50} and E_{max} values were determined for the compounds showing an agonist effect of at least 70 % at 10 µM and an increasing response at 30 µM and 100 µM. The EC_{50} and E_{max} values of compounds with a profile of constant efficacy at 10 µM, 30 µM, and 100 µM, indicating a partial agonist activity, were also determined.[a] Source data are provided as a Source Data file.

ID	2D structures	EC_{50} [µM ± SD][b]	E_{max} [% ± SD][c]	n[d]
**TM2-TM7 pocket optimization**				
**1a**	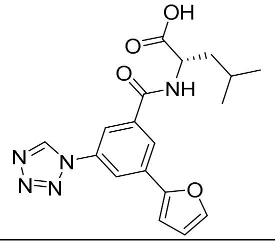	26 ± 0.71	90 ± 1	2
**2a**	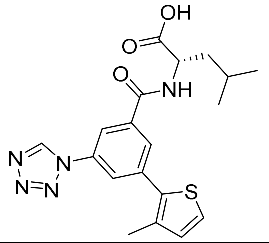	12 ± 4.5	88 ± 6	3
**3a**	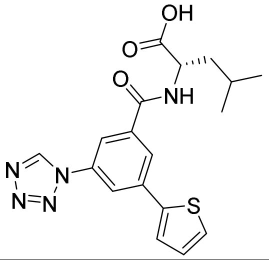	9.8 ± 3.2	94 ± 9	2
**4a**	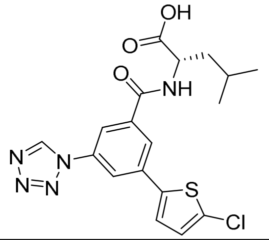	1.9 ± 0.45	97 ± 2	4
**5a**	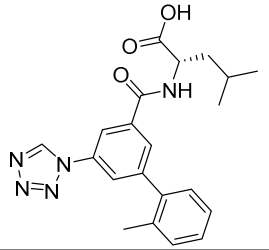	14 ± 0.7	54 ± 2	2
**6a**	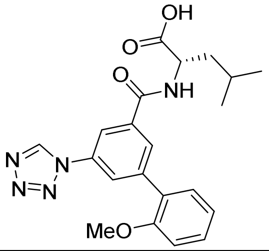	15 ± 1.4	64 ± 6	2

**7a**	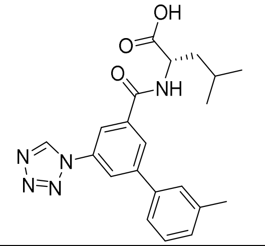	18 ± 7.8	73 ± 7	2
**8a**	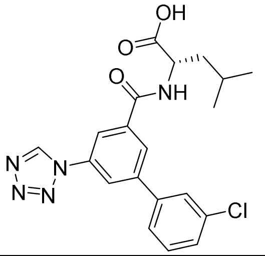	19 ± 7.8	84 ± 8	2
**9a**	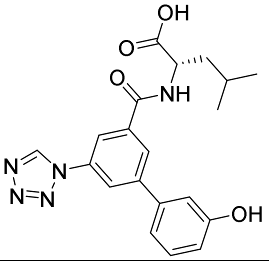	11 ± 0.0	83 ± 2	2
**10a**	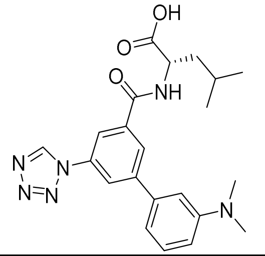	52 ± 10	55 ± 5	5
**11a**	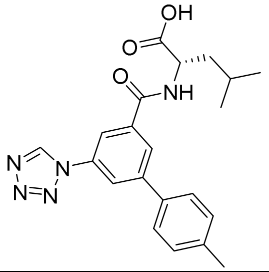	4.5 ± 1.2	86 ± 5	3
**12a**	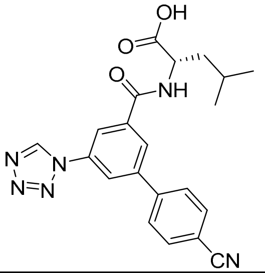	2.9 ± 0.12	97 ± 4	3
**13a**	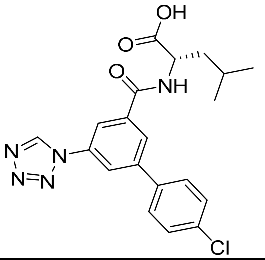	2.2 ± 0.20	97 ± 2	3

**14a**	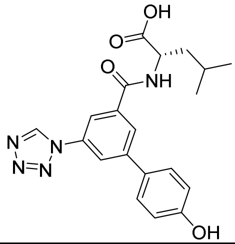	6.0 ± 0.78	91 ± 5	2
**15a**	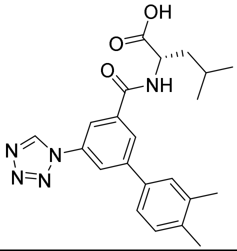	13 ± 6.1	86 ± 11	2
**16a**	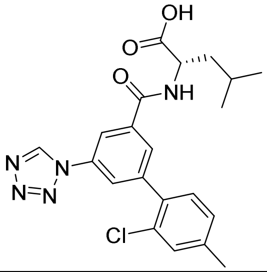	5.5 ± 0.99	84 ± 8	2
**17a**	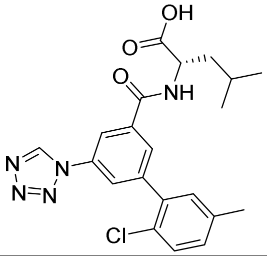	17 ± 2.8	78 ± 6	2
**18a**	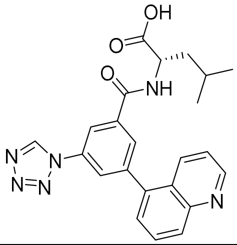	3.1 ± 0.85	75 ± 3	2
**19a**	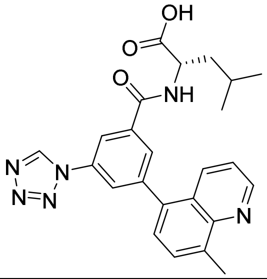	3.3 ± 0.49	64 ± 1	2
**20a**	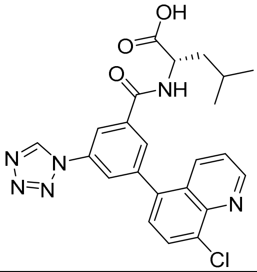	2.4 ± 0.23	80 ± 1	4

**21a**	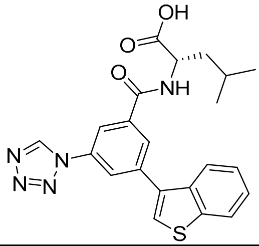	8.9 ± 3.0	60 ± 4	2
**22a**	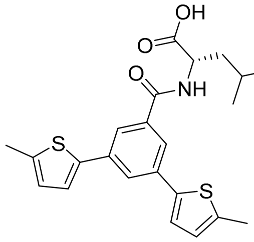	3.1 ± 1.4	86 ± 2	2
**23a**	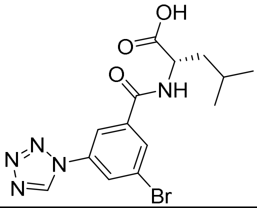	74 ± 16	104 ± 9	4
**NTS$_{8\text{-}13}$ Tyr$_{11}$ pocket optimization**				
**24a**	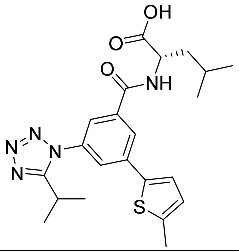	2.4 ± 0.92	94 ± 3	2
**25a**	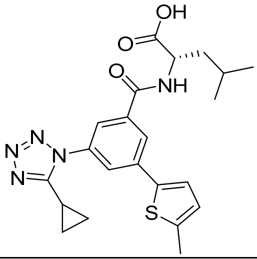	2.3 ± 0.71	91 ± 4	2
**26a**	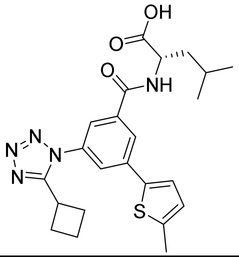	1.9 ± 0.28	91 ± 4	2
**27a**	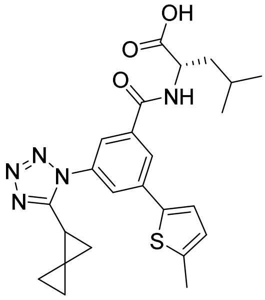	1.1 ± 0.21	90 ± 1	2

**28a**	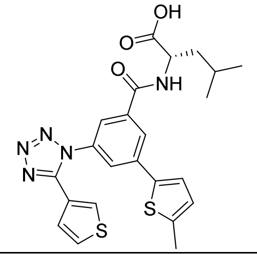	0.15 ± 0.01[e]	101 ± 1[f]	15
**29a**	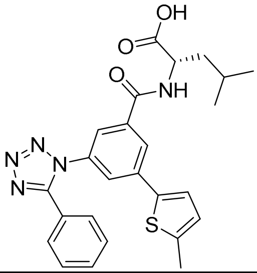	0.41 ± 0.10[e]	101 ± 4[f]	6
**30a**	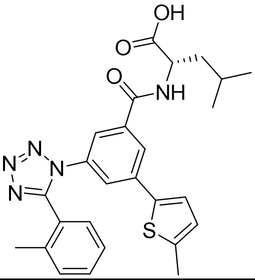	0.46 ± 0.04[e]	98 ± 1[f]	15
**31a**	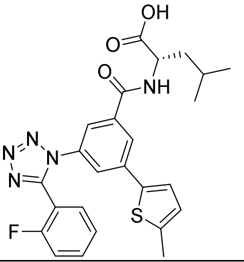	0.60 ± 0.14[e]	91 ± 5[f]	5
**32a**	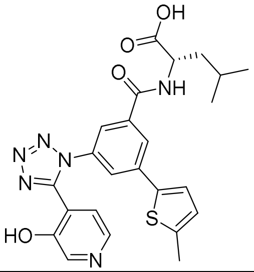	1.3 ± 0.50	92 ± 7	2
**33a**	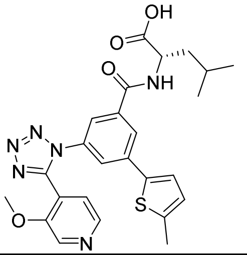	1.2 ± 0.54	95 ± 2	2

**34a**	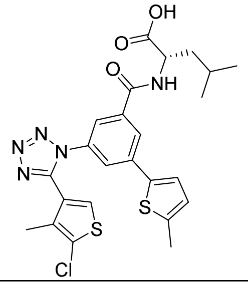	3.5 ± 0.42	89 ± 4	2
**Leucine substitution**				
**35a**	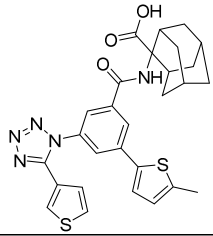	0.44 ± 0.09[e]	81 ± 6[f]	8

[a] Receptor activation was determined by applying an IP_1 accumulation assay measuring $G\alpha_q$ mediated second messenger accumulation. [b] Potency for $hNTS_1R$ activation in µM ± SD. [c] Maximum efficacy in % ± SD relative to the full effect of NTS8-13. [d] Number of individual experiments, each performed in duplicate. [e] Potency for $hNTS_1R$ activation in µM ± S.E.M. [f] Maximum efficacy in % ± S.E.M.

Supplementary Table S4. Functional activities of compound **25** analogs for $hNTS_1R$. The EC_{50} and E_{max} values were determined for the compounds showing an agonist effect of at least 70 % at 10 µM and an increasing response at 30 µM and 100 µM. The EC_{50} and E_{max} values of compounds with a profile of constant efficacy at 10 µM, 30 µM, and 100 µM, indicating a partial agonist activity, were also determined.[a] Source data are provided as a Source Data file.

ID	2D Structure	EC_{50} [µM ± SD][b]	E_{max} [% ± SD][c]	n[d]
**Leucine substitution**				
**1b**	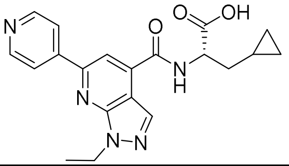	n.d[e]	n.d	
**2b**	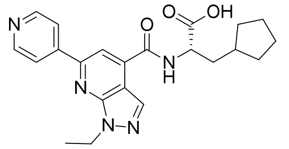	9.2 ± 6.7	98 ± 7	5
**3b**	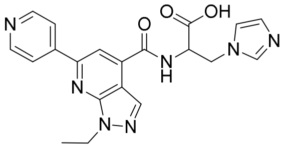	n.d	n.d	
**4b**	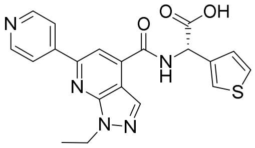	n.d	n.d	
**5b**	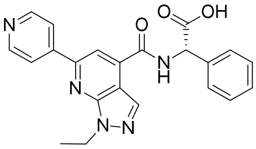	n.d	n.d	

**6b**	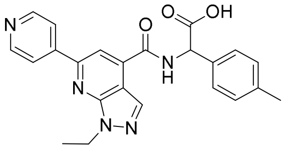	n.d	n.d	
**7b**	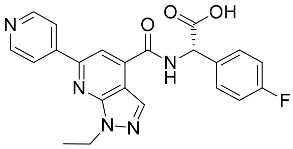	n.d	n.d	
**8b**	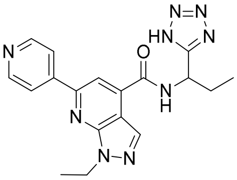	n.d	n.d	
**9b**	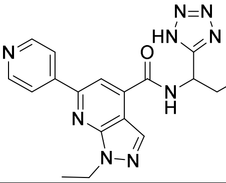	n.d	n.d	
**10b**	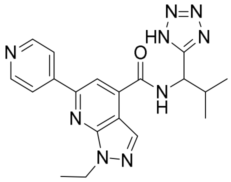	n.d	n.d	
**11b**	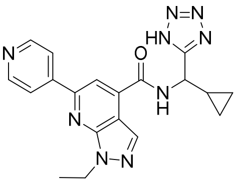	n.d	n.d	
**12b**	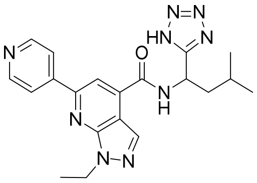	7.6 ± 1.5	81 ± 2	3
**13b**	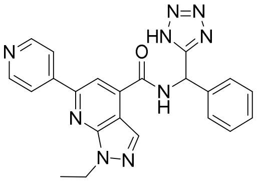	n.d	n.d	
**Pyridine substitution**				
**14b**	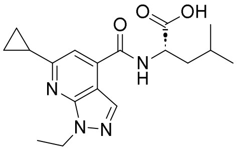	n.d	n.d	
**15b**	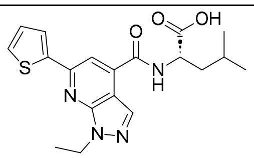	n.d	n.d	

**16b**	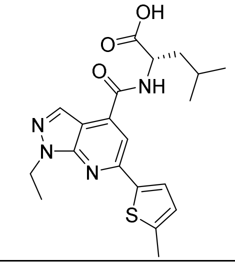	7.1 ± 2.2	79 ± 5	3
**17b**	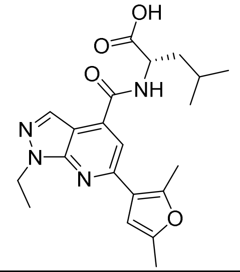	3.1 ± 1.1	83 ± 6	3
**18b**	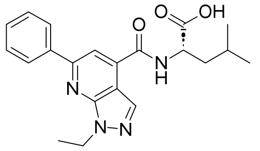	n.d	n.d	
**19b**	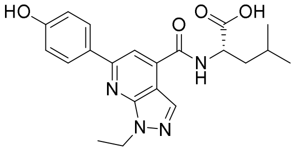	n.d	n.d	
**20b**	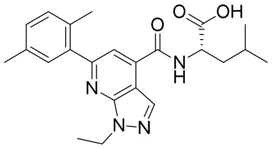	n.d	n.d	
**21b**	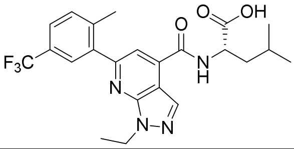	n.d	n.d	
**22b**	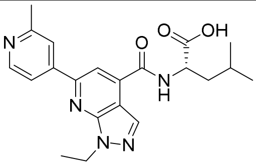	6.0 ± 0.64	92 ± 9	2
**23b**	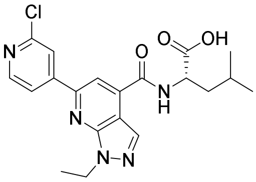	-	23 ± 14 @100 µM	5
**24b**	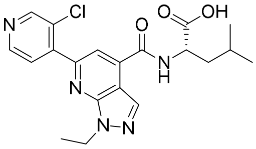	n.d	n.d	
**25b**	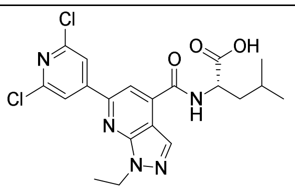	n.d	n.d	

**26b**	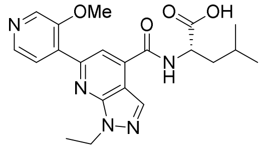	n.d	n.d	
**27b**	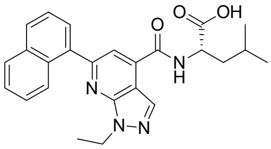	9.0 ± 4.1	57 ± 6	4
**Ethyl substitution**				
**28b**	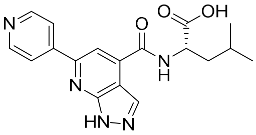	n.d	n.d	
**29b**	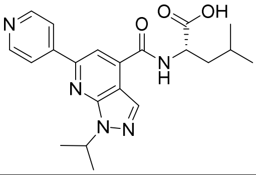	19 ± 5.3	80 ± 13	3
**30b**	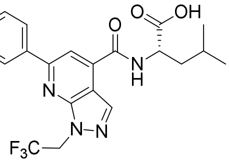	n.d	n.d	
**31b**	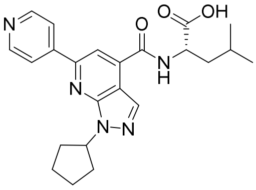	n.d	n.d	
**32b**	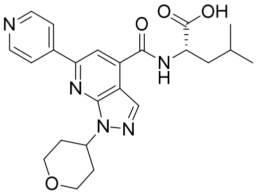	n.d	n.d	
**33b**	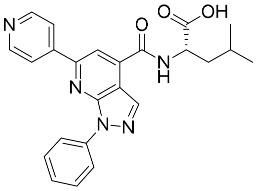	n.d	n.d	
**34b**	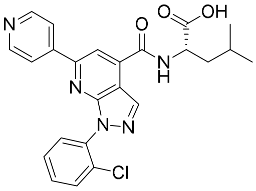	n.d	n.d	
**35b**	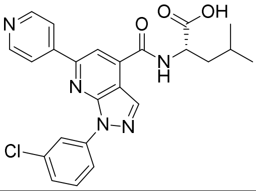	6.4 ± 2.2	38 ± 17	3

**36b**	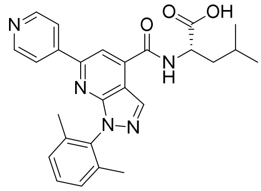	3.6 ± 0.49	91 ± 6	2
**37b**	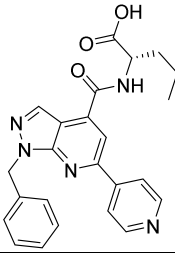	1.1 ± 0.17	90 ± 3	4
**38b**	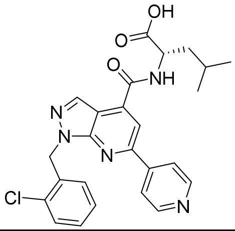	0.92 ± 0.51	92 ± 9	6
**39b**	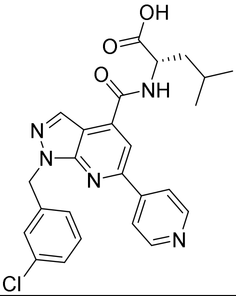	1.8 ± 0.82	90 ± 4	6
**40b**	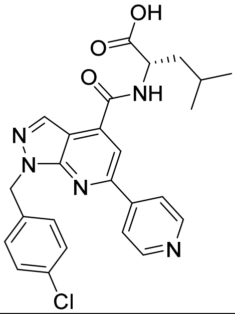	2.5 ± 0.42	77 ± 0	2
**41b**	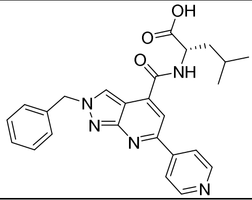	52 ± 39	52 ± 8	4
**Ethyl and pyridine substitution**				
**42b**	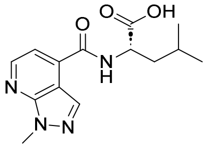	n.d	n.d	
**43b**	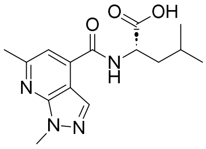	n.d	n.d	

**44b**	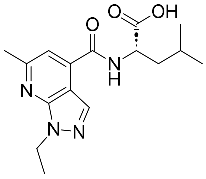	n.d	n.d	
**45b**	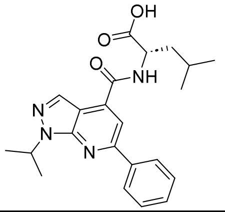	n.d	n.d	
**46b**	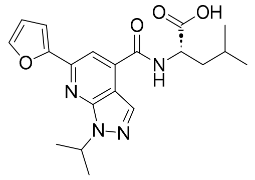	n.d	n.d	
**47b**	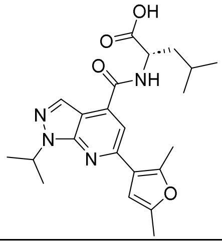	2.8 ± 0.35	82 ± 1	2
**48b**	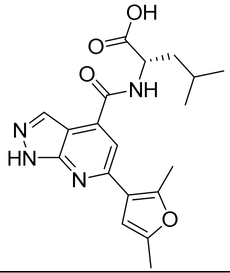	25 ± 9.9	71 ± 2	2
**49b**	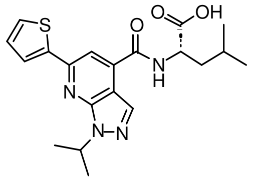	n.d	n.d	
**50b**	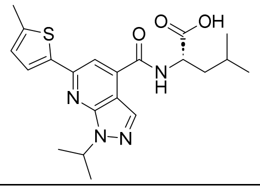	n.d	n.d	
**51b**	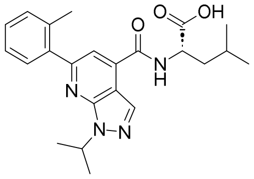	n.d	n.d	
**52b**	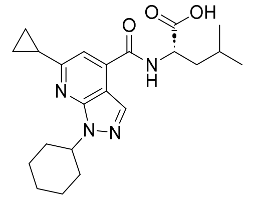	n.d	n.d	

**53b**	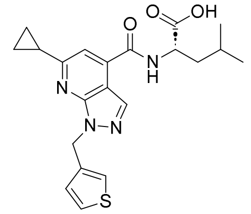	3.2 ± 0.90	93 ± 4	3
**54b**	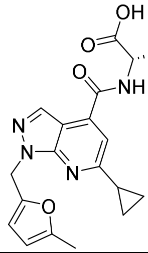	2.9 ± 1.1	99 ± 1	2
**55b**	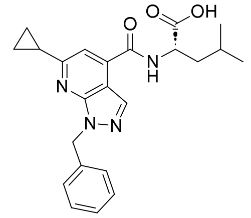	2.6 ± 1.0	97 ± 4	3
**56b**	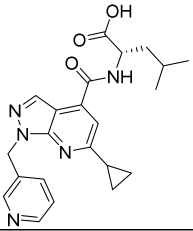	8.1 ± 3.6	99 ± 7	3
**57b**	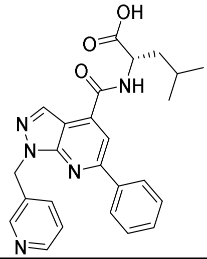	7.1 ± 0.99	78 ± 4	2
**58b**	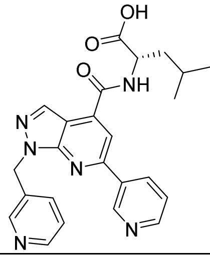	11 ± 3.6	57 ± 9	3
**59b**	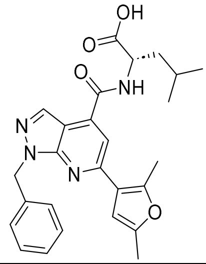	2.1 ± 0.21	81 ± 3	2

**60b**	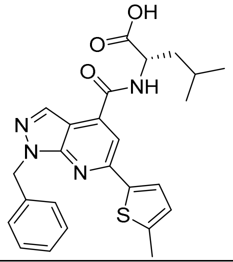	6.0 ± 0.38	73 ± 3	3

[a] Receptor activation was determined by applying an IP_1 accumulation assay measuring $G\alpha_q$ mediated second messenger accumulation. [b] Potency for $hNTS_1R$ activation in µM ± SD. [c] Maximum efficacy in % ± SD relative to the full effect of NTS8-13. [d] Number of individual experiments, each performed in duplicate. [e] Not determined.

Supplementary Table S5. Physicochemical properties and similarity to reference agonists of compounds **28a**, **30a**, and **35a**.

	Physicochemical properties[a]				Similarity[b]
	**MW (Da)**	**HBA**	**HBD**	**cLogP**	**(T_C)**
**Lipinski's rule**	< 500	≤ 10	≤5	≤5	-
**28a**	482	8	2	4.7	0.29
**30a**	490	8	2	4.9	0.31
**35a**	546	8	2	5.4	0.16

[a] MW, molecular weight; HBA, hydrogen bond acceptors; HBD, hydrogen bond donors; LogP, calculated logarithm of the octanol/water partition coefficient. Properties were calculated using RDKit.[7] [b] Maximal Tanimoto similarity (T_c, ECFP4-based fingerprints) of the compound to reference agonists **SRI-9829** and **RTI-3a**. In virtual screening applications, compound pairs with $T_c < 0.35$ are typically considered dissimilar.[8]

Supplementary Table S6. Affinities of selected compounds for the thermostabilized variant NTSR1-H4 and wild-type $rNTS_1R$.[a] Source data are provided as a Source Data file.

	NTSR1-H4		$rNTS_1R$	
	K_i (µM) ± SEM	n	K_i (µM) ± SEM	n
**28a**	0.167 ± 0.030	6	37 ± 5.2	5
**30a**	0.041 ± 0.008	6	43 ± 6.3	5
**SRI-9829**	0.0020 ± 0.0004	5	3.4 ± 0.6	10
**RTI-3a**	0.501 ± 0.050	4	0.153 ± 0.029	5
**NTS_{8-13}**	0.00020 ± 0.00004	4	0.0024 ± 0.0003	11

[a]Binding affinities were determined by competition of HiLyte Fluor 488-labeled NTS_{8-13} in HTRF-binding experiments with membranes from transiently transfected HEK293T cells expressing SNAP-tagged NTSR1-H4 or SNAP-tagged $rNTS_1R$. IC_{50} values were obtained from a non-parametric curve fit and corrected with the Cheng-Prusoff equation to obtain K_i values.[9] Data reflect means of 4-11 independent experiments ± SEM measured in duplicate.

Supplementary Table S7. Final data collection statistics.[a]

**Ligand**	**28a**	**30a**
**PDB accession code**	**9QC1**	**9QD4**
**Data collection**		
Space group	$C222_1$	$C222_1$
Cell dimensions		
a,b,c (Å)	75.66<br>215.89<br>95.62	75.93<br>215.39<br>95.85
α,β,γ (˚)	90<br>90<br>90	90<br>90<br>90
Resolution (Å)	107.94–3.12<br>(3.47–3.12)	71.61–3.10<br>(3.39–3.10)
R_{merge}	0.22 (4.70)	1.00 (9.86)
R_{pim}	0.05 (0.97)	0.25 (2.44)
$I/\sigma(I)$	10.8 (1.4)	8.5 (1.4)
$CC_{1/2}$	0.99 (0.2)	0.97 (0.71)
Completeness (%)	87.8 (43.6)	91.0 (39.9)
Redundancy	25.6 (22.3)	15.8 (16.1)
**Refinement**		
Resolution (Å)	71.4–3.12	37.94–3.12
No. reflections	9367	11354
No. atoms		
Protein	3160	3304
Ligand	33	35
Oligomeric state	Monomer	Monomer
R_{work}/R_{free}	0.294/0.308	0.294/0.307
Ramachandran plot	0/2.44/97.56	0/1.75/98.25
MolProbity Overall score	0.92	0.99
MolProbity Clash score	1.17	2.16
Rotamer outliers [%]	0.38	0.33
r.m.s. deviations		
Bond lengths (Å)	0.01	0.01
Bond angles (˚)	1.15	1.24

[a] Output by autoPROC[10] that makes use of the data processing package XDS[11], the scaling program AIMLESS[12], and the program STARANISO[13] for anisotropy correction. Numbers in brackets denote values determined for the highest resolution shells. Refinement of the complexes, performed in ISOLDE[14] and REFMAC5[15], was analyzed by the MolProbity[16] module within the program package Phenix.[17]

Supplementary Table S8. Affinities of compounds **28a**, **30a**, and **35a** for $rNTS_2R$. Source data are provided as a Source Data file.

Compound	K_i ± SEM (nM)[a]
**$NTS_{8\text{-}13}$**	0.59 ± 0.22
**28a**	22 ± 15
**30a**	8.8 ± 6.6
**35a**	8.1 ± 5.8

[a] The binding results were normalized to $NTS_{8\text{-}13}$ binding (From 100 % radioligand-receptor association to 0 % association of the ^{125}I-[Tyr3]-NTS on NTS_2R). Data are expressed ± SEM of n = 3 separate experiments in duplicate for $NTS_{8\text{-}13}$ and n = 3 separate experiments in duplicate for **28a**, **30a**, and **35a**.

Supplementary Table S9. Affinities of compounds **28a**, **30a**, and **35a** for human GPCRs: Opioid (μ, κ, and δ), serotonin ($5\text{-}HT_{1A}$), adrenergic (α_{2A}), and apelin (APJ) receptors.[a] Source data are provided as a Source Data file.

receptor	**28a**		**30a**		**35a**	
	K_i (μM) ± SEM[b]	n[c]	K_i (μM) ± SEM[b]	n[c]	K_i (μM) ± SEM[b]	n[c]
μ	59 ± 18	4	46 ± 19	4	14 ± 3.0	4
κ	50 ± 21	4	16 ± 5.5	4	21 ± 9.2	4
δ	67 ± 12	4	24 ± 4.4	4	22 ± 7.6	4
$5\text{-}HT_{1A}$	64 ± 12	4	49 ± 17	4	15 ± 2.3	5
α_{2A}	48 ± 18	4	91 ± 4.8	4	18 ± 5.4	3
APJ	>100	2	>100	2	>100	2

[a] Binding affinities determined by radioligand displacement with membranes from HEK293T cells expressing the respective human receptor. [b] Mean K_i values and standard deviation. [c] Number of individual experiments, each performed in triplicate for the μ, κ, δ, $5\text{-}HT_{1A}$ and α_{2A} receptors, and in duplicate for the APJ receptor.

Supplementary Table S10. Concentration of compound **28a** in brain tissue and CSF samples from pharmacokinetic study in rats.

Animal	Time (h)	Concentration (nM)	
		Brain tissue[a]	CSF
**1**	2	< 4	6[b]
**2**	2	< 4	25[b]
**3**	2	< 4	2
**4**	6	< 4	< 2[c]
**5**	6	< 4	< 2[c]
**6**	6	< 4	2[b]
**7**	24	< 4	-[d]
**8**	24	< 4	< 2[c]
**9**	24	< 4	< 2[c]
**10**	24	< 4	< 2[c]
**11**	24	< 4	< 2[c]
**12**	24	< 4	6[b]

[a] Concentration below the lower limit of quantification (LLOQ; 4 nM) for all tested samples. [b] Sample showed clear evidence of blood contamination based on visual inspection. It should be noted that four of the five CSF samples in which low concentrations of compound **28a** were detected (2-25 nM) also showed evidence of blood contamination, likely leading to artificially elevated values. [c] Concentration below the lower limit of quantification (LLOQ; 2 nM). [d] No sample was obtained.

Supplementary Table S11. Vendor and catalog identifiers for commercially available compounds.

Compound	Vendor	Vendor ID
**1**	Enamine	Z4464967438
**2**	Enamine	Z4467182693
**3**	Enamine	Z4467101527
**4**	Enamine	Z1268335947
**5**	Enamine	Z2353285861
**6**	Enamine	Z4467139101
**7**	Enamine	Z4467144925
**8**	Enamine	Z4467198837
**9**	Enamine	Z4467130062
**10**	Enamine	Z4467101591
**11**	Enamine	Z4467101607
**12**	Enamine	Z4467177527
**13**	Enamine	Z4467133482
**14**	Enamine	Z4467152713
**15**	Enamine	Z4467136202
**16**	Enamine	Z4467150309
**17**	Enamine	Z4467148082
**18**	Enamine	Z4467138487
**19**	Enamine	Z4467137998
**20**	Enamine	Z4467126385
**21**	Enamine	Z4467147014
**22**	Enamine	Z4467121967
**23**	Enamine	Z4467151055
**15b**	Enamine	Z1455256721
**18b**	Enamine	Z1455256081
**42b**	Enamine	Z3800940066
**43b**	Enamine	Z1455260878
**44b**	Enamine	Z4067868795
**45b**	Enamine	Z1455261481
**46b**	Enamine	Z1455262691
**47b**	Enamine	Z1455263295
**49b**	Enamine	Z1455263293
**50b**	Enamine	Z4114376246
**50b**	Enamine	Z5408836859
**51b**	Enamine	Z5408836878
**52b**	Enamine	Z5408836905
**53b**	Enamine	Z1455263182
**55b**	Enamine	Z1455259716

Supplementary Table S12. Mutations of rNTSR1-H4 compared to wild-type rNTS$_1$R.[a]

Sequential	B-W	wild-type rNTS$_1$R	rNTSR1-H4
83	1.51	S	G
86	1.54	A	L
101	2.38	T	R
103	2.40	H	D
105	2.42	H	Y
119	2.56	L	F
121	2.58	M	L
124	2.61	E	D
143	3.26	R	K
150	3.33	D	E
161	3.44	A	V
167	3.50	R	L
213	4.69	R	L
234	5.35	V	L
235	5.36	K	R
240	5.41	V	L
253	5.54	I	A
260	5.61	I	A
262	5.63	N	R
263	5.64	K	R
305	6.32	H	R
332	6.59	C	V
342	7.26	F	A
354	7.38	T	S
358	7.42	F	V
362	7.46	S	A

[a] The mutation F342→A had not been reported in the original reference[18]. rNTSR1-H4 has also been termed HTGH4 elsewhere.[19,20] B-W: Ballesteros-Weinstein numbering system.[21]

Supplementary Table S13. The LC-MS/MS multiple reaction monitoring (MRM) transitions used for quantification of *in vivo* samples.

Compound name	Parent (m/z)	Daughter (m/z)	Cone (V)	CE (V)	Notes
BCS-IS[a]	272.0957	92.0693	34	28	IS for brain homogenate, CSF, plasma (undiluted)
Warfarin	309.1596	163.005	28	16	IS for plasma (diluted), and dosing solution, due to insufficient signal of BCS
**28a**	482.1319	294.6184	45	23	
**28a**	482.1319	322.9209	45	19	Used for quantification

[a] BCS, carbutamide; IS, internal standard.

Supplementary methods: Chemistry

General synthetic procedures. All reagents were purchased from Fluorochem, Sigma-Aldrich, Enamine and Chemtronica. DCM, methanol, DMF, and acetonitrile (99.9%) were purchased from VWR International AB, whereas THF was purchased from Sigma-Aldrich. Reagents and solvents were used as such without further purification. All reactions involving air or moisture-sensitive reagents or intermediates were performed under a nitrogen atmosphere. Mainly LC-MS was used for monitoring reactions using an Agilent 1100 series HPLC having a C18 Atlantis T3 column (3.0 × 50 mm, 5 μm). Acetonitrile–water (flow rate 0.75 mL/min with gradient of 5-95% of acetonitrile over 6 min) was used as mobile phase and a Waters micromass Z_Q (model code: MM1) mass spectrometer with electrospray ionization mode was used for detection of molecular ions. TLC silica gel 60 F_{254} plates from Merck were also sometimes used for monitoring reactions and particularly during purification of compounds. Visualization of the developed TLC was done using UV light (254 nm) and staining with ninhydrin or anisaldehyde stain. After workup, organic phases were dried over Na_2SO_4/$MgSO_4$ and filtered before being concentrated under reduced pressure. Silica gel (Matrex, 60 Å, 35–70 μm, Grace Amicon) was used for purification of intermediate compounds with flash column chromatography. ^{1}H and ^{13}C NMR spectra were recorded at 298 K on an Agilent Technologies 400 MR spectrometer at 400 MHz or 100 MHz, or on Bruker Avance Neo spectrometers at 500 MHz or 125 MHz. Chemical shifts are reported in parts per million (ppm, δ) referenced to the residual ^{1}H resonance of the solvent ($(CD_3)_2CO$, δ 2.05; $CDCl_3$, δ 7.26; CD_3OD, δ 3.31; DMSO-d_6, δ 2.50). Splitting patterns are designated as follows: s (singlet), d (doublet), t (triplet) and m (multiplet), br (broad). Coupling constants (J values) are listed in hertz (Hz). Preparative reversed-phase HPLC was performed on a Kromasil C8 column (250 × 21.2 mm, 5 μm) on a Gilson HPLC equipped with a Gilson 322 pump, UV/Visible-156 detector and 202 collector using acetonitrile-water gradients as eluents with a flow rate of 15 mL/min and detection at 210 or 254 nm. Unless otherwise stated, all the tested compounds were purified by HPLC. The purity of all tested compounds is ≥95% as determined by high resolution ^{1}H NMR spectroscopy (500/600 MHz) and LCMS.

Synthesis of compounds selected from virtual screening 24-40 (17 compounds)

General procedure A: One pot, 2 steps of amide coupling and ester hydrolysis using L-leucine ethyl ester

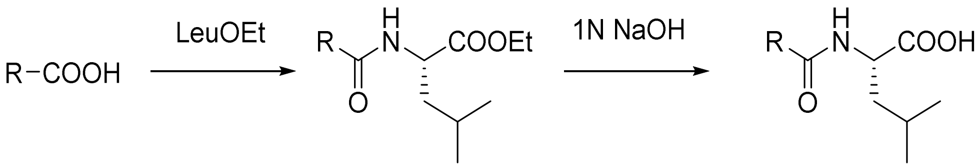

A mixture of the carboxylic acid (≤ 0.05 mmol), L-leucine ethyl ester hydrochloride (1 equiv), 2-(1H-benzotriazole-1-yl)-1,1,3,3-tetramethylaminium tetrafluoroborate (TBTU) (1 equiv), Et_3N (2 equiv) in DMSO (0.7 mL) (DMF was used instead for **24**) was stirred at rt for 1 h. Then 1 N NaOH solution was added (6 equiv) and the mixture was stirred at rt for 15 min and then acidified with TFA. After filtering, the mixture was directly purified by HPLC using a gradient of 20-90% of acetonitrile in H_2O (H_2O + 0.1% TFA) for 30 min to afford the desired product. Yield: 20-52%.

Optimization of compound 24 (Scaffold a)

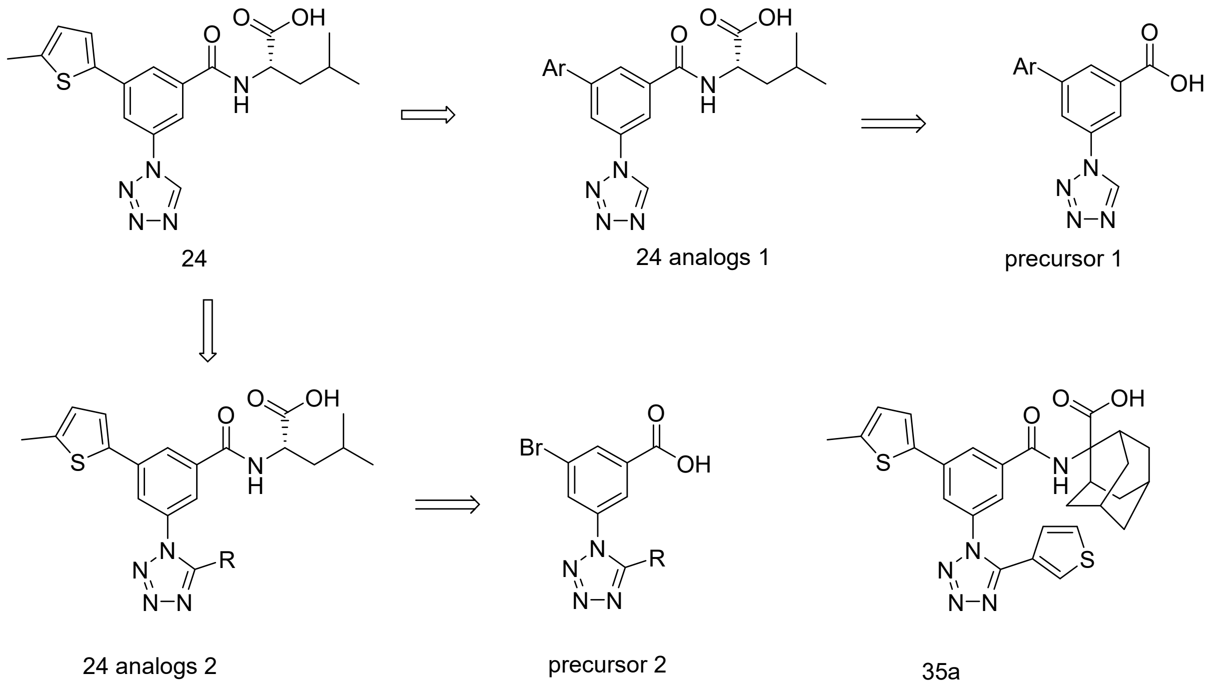

Scheme 1. General structures of the two series of analogs of compound **24**, and the corresponding carboxylic acids required for their synthesis. The structure of an adamantyl analog of compound **24** has also been included.

Compound **24** analogs 1 were synthesized starting from 3-amino-5-bromobenzoic acid (**1**) (scheme 2). Reaction of **1** with sodium azide and ethyl orthoformate in AcOH furnished a tetrazole bromobenzoic acid intermediate, which was employed directly in a Suzuki coupling with a series of arylboronic acids using $PdCl_2dppf$ as catalyst to afford the desired benzoic acids (**Precursors 1**). The benzoic acids (**Precursors 1**) were then used without purification in the subsequent coupling with L-leucine ethyl ester using TBTU as coupling reagent, followed by ester hydrolysis to afford the final compounds (compound **24 analogs 1**).

Compound **24** analogs 2 were synthesized by reaction of methyl 3-amino-5-bromobenzoate (**2**) (scheme 2) with a series of acyl chlorides to furnish the corresponding amides. Transformation of the amides to iminium chlorides using $SOCl_2$, followed by condensation with sodium azide to provide a tetrazole and saponification of the methyl ester afforded the target benzoic acids (**Precursors 2**). Amide coupling of **Precursors 2** with L-leucine ethyl ester furnished an aryl bromide intermediate, which was employed in a Suzuki coupling with (5-methylthiophen-2-yl)boronic acid using $PdCl_2dppf$ as catalyst, followed by ester hydrolysis to afford the final products (compound **24 analogs 1**).

Compound **35a**, an analog of **24** analogs 2, was synthesized from precursor 2 by coupling to a-amino adamantyl tertbutyl ester, Suzuki coupling with (5-methylthiophen-2-yl)boronic acid and deprotection of the tert-butyl ester by 4N HCl in dioxane.

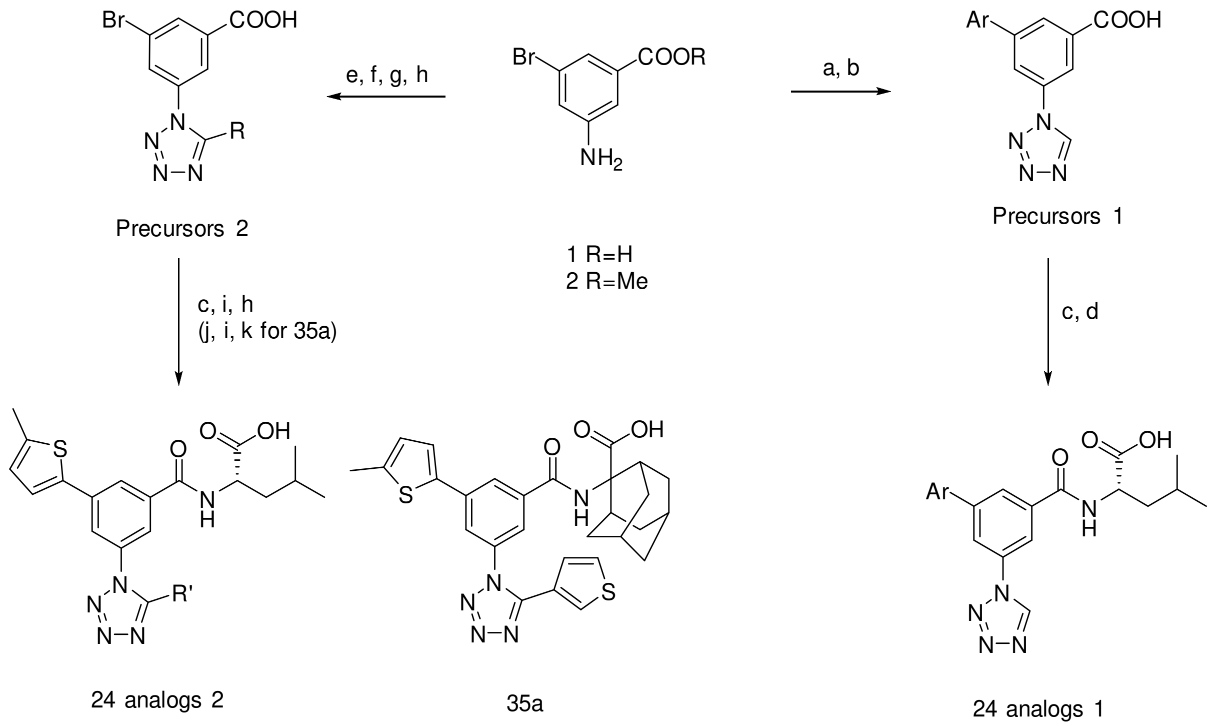

Scheme 2. Synthesis of two series of analogs of compound **24** and the analog **35a**. Compound **24** analogs **1** were prepared starting from **1**, while compound **24 analogs 2** and **35a** were prepared from **2**. Reagents and conditions: a) NaN_3, $CH(OEt)_3$, AcOH, 110 °C, 5 h. b) $PdCl_2dppf$, $ArB(OH)_2$, K_2CO_3, 1:1 dioxane:H_2O, 100 °C, 2 h. c) L-LeuOEt.HCl, TBTU, Et_3N, DMF, rt, 30 min (DMSO instead of DMF for analogs 2). d) 1N NaOH, 7:3 DMF:H_2O, rt, 30 min. e) RCOCl, Et_3N, DCM, rt, 1 h. f) $SOCl_2$, 80 °C, 3 h. g) NaN_3, CH_3CN, 90 °C, overnight. h) 1N NaOH, 7:3 DMSO:H_2O, 70 °C, 30 min. i) $PdCl_2dppf$, (5-methylthiophen-2-yl)boronic acid, K_2CO_3, 7:3 DMSO:H_2O, 100 °C, 2 h. j) tert-butyl 2-aminoadamantane-2-carboxylate, HATU, 1:1 DMF:DCM, Et_3N, DMF, rt, 30 min .k) 4N HCl/DOX, rt, ovn.

Synthesis of compounds 1a-23a (compound 24 analogs 1, 23 compounds) from 3-(1H-tetrazol-1-yl)-5-substituted benzoic acids

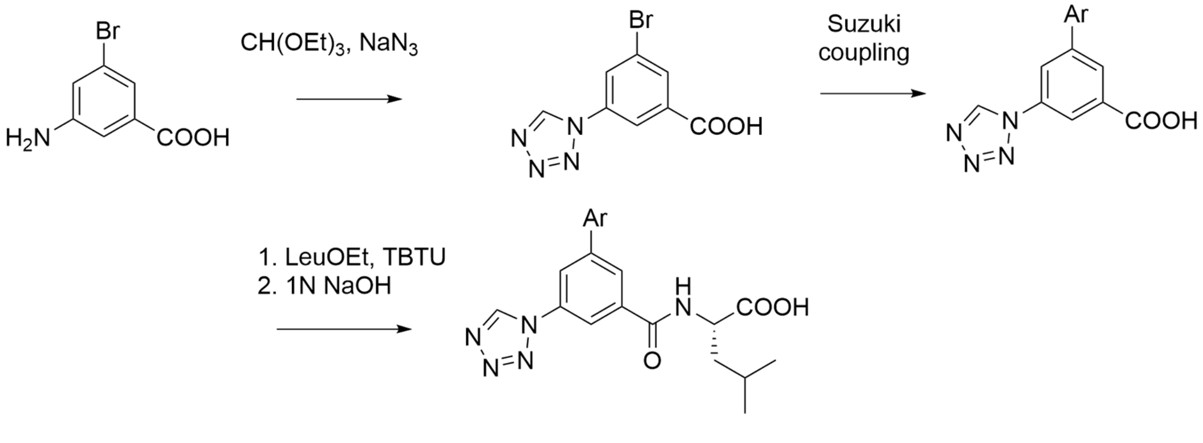

Step 1. 3-bromo-5-(1H-tetrazol-1-yl)benzoic acid

A mixture of 3-amino-5-bromobenzoic acid (9 mmol), NaN_3 (4 equiv), triethyl orthoformate (4 equiv) in AcOH (9 mL) was heated at 110 °C for 5 hrs. After cooling down and dilution with H_2O (200 mL), the precipitate was filtered and dried to afford the desired product as light brown solid. Yield: 85%.

^{1}H NMR (500 MHz, DMSO-d$_6$) δ 13.86 (s, 1H), 10.24 (d, *J* = 1.6 Hz, 1H), 8.48 (t, *J* = 2.2 Hz, 1H), 8.43 (d, *J* = 1.9 Hz, 1H), 8.19 (d, *J* = 1.9 Hz, 1H).
^{13}C NMR (126 MHz, DMSO- d$_6$) δ 164.9, 142.7, 135.2, 134.6, 132.5, 127.7, 122.7, 120.6.
LCMS (ESI+): calculated for $C_8H_6BrN_4O_2$ $(M+H)^+$: 269.0; found 269.3.

Step 2. General procedure for Suzuki coupling

A mixture of 3-bromo-5-(1H-tetrazol-1-yl)benzoic acid (0.1 mmol), $ArB(OH)_2$ (1.5 equiv), $Pd(OAc)_2$ (2% mol), Sphos (4% mol), and K_2CO_3 (2 equiv) in iPrOH:H_2O (1:1, v/v, 1 mL) was heated at 80 °C for 2 hrs. After cooling down, the mixture was acidified with 1N HCl and extracted with ethyl acetate. The organic phase was dried over Na_2SO_4, filtered and concentrated under reduced pressure to give a crude of the desired 3-(1H-tetrazol-1-yl)-5-substituted benzoic acid, which was used as such in the next step. Yield: 78-100%.

Step 3. One pot amide coupling and ester hydrolysis, using **procedure A** with DMF as solvent. Yield: 10-40%.

Synthesis of compounds 24a-34a (compound 24 analogs 2, 11 compounds) from 3-(5-substituted-1H-tetrazol-1-yl)-5-(5-methylthiophen-2-yl)benzoic acids

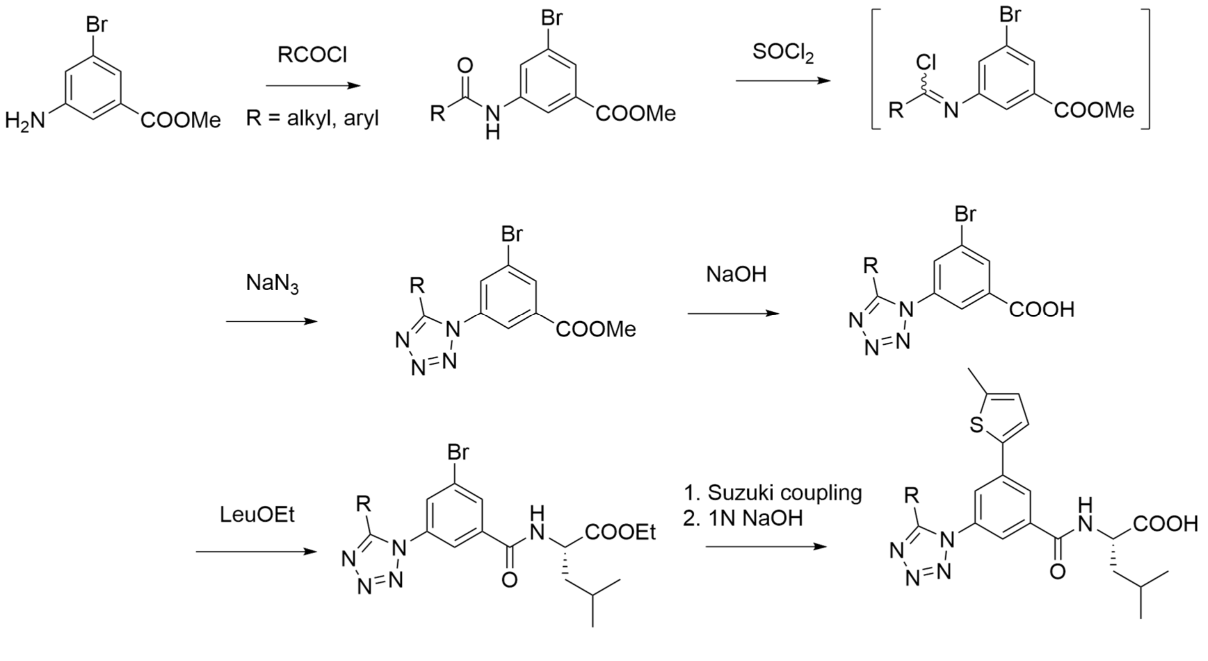

Step 1. Synthesis of 3-(5-substituted-1H-tetrazol-1-yl)-5-(5-methylthiophen-2-yl)benzoic acids.

Et_3N (1 equiv) and acyl chloride reagent (1 equiv) were added to a solution of methyl 3-amino-5-bromobenzoate in DCM (4 mL/mmol) at 0 °C. The reaction was stirred at rt for 30 min. Solvent was removed to dryness and $SOCl_2$ (18 equiv) was added. The

mixture was stirred at 80 °C for 2 hrs to form a chloro-imine intermediate. After concentrating to dryness, NaN_3 and ACN (4 mL/mmol) were added and the mixture was stirred overnight at 90 °C to form the tetrazole. After cooling down, 1N NaOH (10 equiv) was added and the reaction was heated at 70 °C for 30 min to hydrolyse the methyl ester. After acidifying by 1N HCl, the product, 3-(5-substituted-1H-tetrazol-1-yl)-5-(5-methylthiophen-2-yl)benzoic acid, was extracted by ethyl acetate and used as such for the next step or purified by HPLC. Yield: 10-50% (overall).

Step 2. One pot synthesis of **24a-34a** through amide coupling, suzuki coupling and ester hydrolysis.

A mixture of benzoic acid (≤ 0.05 mmol), L-Leucine ethyl ester hydrochloride (1 equiv), 2-(1H-benzotriazole-1-yl)-1,1,3,3-tetramethylaminium tetrafluoroborate (TBTU) (1 equiv), and Et_3N (2 equiv) in DMSO (0.7 mL) was stirred at rt for 1 h. After that, $PdCl_2dppf$ (5% mol), $ArB(OH)_2$ (1.5 equiv), K_2CO_3 (2 equiv), and H_2O (0.3 mL) were added to the reaction mixture. It was degassed and heated at 100 °C for 2 hrs for the Suzuki coupling reaction. After cooling down, aqueous 1 N NaOH solution was added (6 equiv), the mixture was stirred at rt for 15 min and acidified with TFA. After filtering, the mixture was directly purified by HPLC using a gradient of 20-100% of acetonitrile in H_2O (H_2O + 0.1% TFA) for 30 min to afford the desired product. Yield: 10-60%.

Synthesis of compound 35a

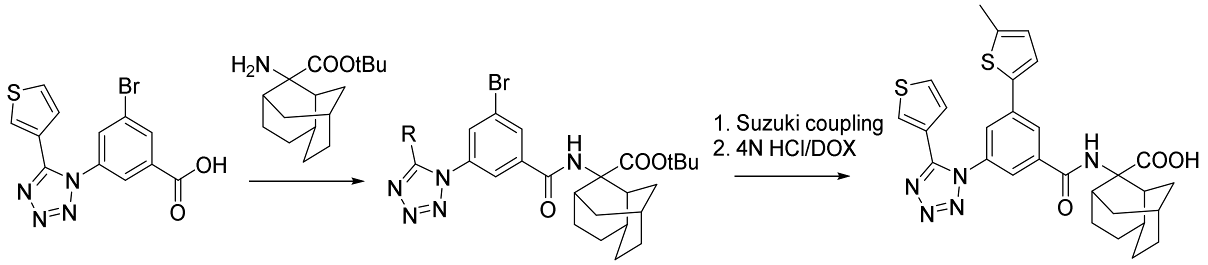

Compound 35a was prepared in a similar manner as above by amide coupling and Suzuki coupling steps. After that, the crude was purified by HPLC using a gradient of 20-100% of acetonitrile in H_2O (H_2O + 0.1% TFA) for 30 min to afford the tert-butyl ester intermediate. The later was deprotected with 4N HCl in dioxane to afford the desired product.

Optimization of compound 25 (Scaffold b)

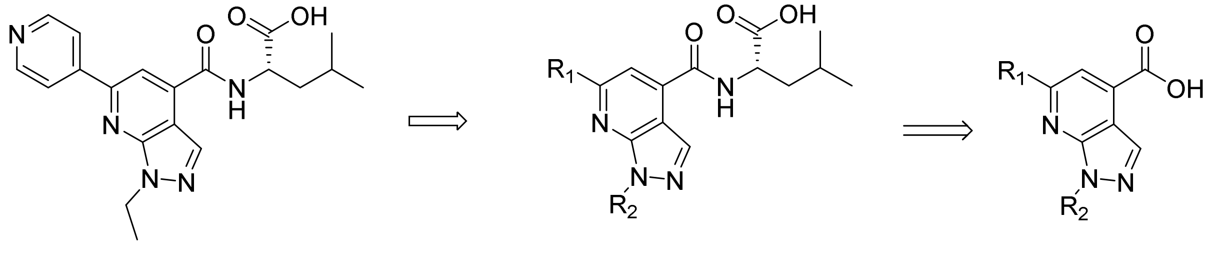

25   25 analogs 3

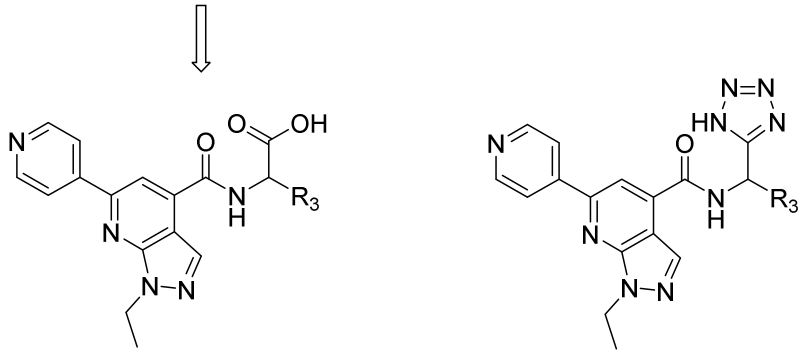

25 analogs 1   25 analogs 2

Scheme 3. General structures of the three types of analogs of **25** originating from variation of the structures of i) the heterocyclic carboxylic acid moiety, ii) the amino acid residue, and iii) by replacement of the carboxyl group by a tetrazole bioisostere.

Compound 25 analogs 1: Application of general procedure A to different amino acid esters (**1b-7b**, 7 compounds). Yield: 16-51%.

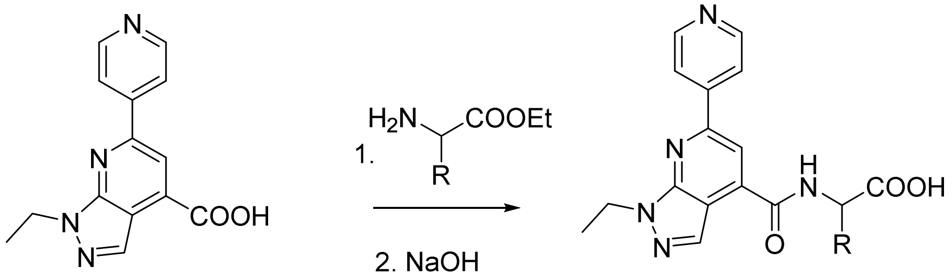

Compound 25 analogs 2 (**8b-13b**, 6 compounds)**:** Using **general procedure B**: Amide coupling to tetrazole analogs of amino acids

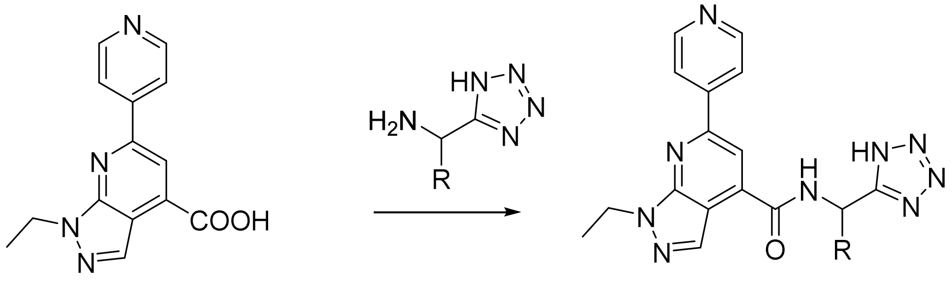

A mixture of the acid (≤ 0.05 mmol, 1 equiv), tetrazole analog of the amino acids (1 equiv), 2-(1H-benzotriazole-1-yl)-1,1,3,3-tetramethylaminium tetrafluoroborate (TBTU) (1 equiv), and Et_3N (2 equiv) in DMSO (0.7 mL) was stirred at rt for 1 h and then acidified with TFA. After filtering, the mixture was directly purified by HPLC using a gradient of 20-90% of acetonitrile in H_2O (H_2O + 0.1% TFA) for 30 min to afford the desired product. Yield: 20-43%.

Compound 25 analogs 3 (**14b-60b**, 57 compounds)**:** Analogs of compound **25** based on leucine modified either at the pyridyl or the ethyl substituent of the 1H-pyrazolo[3,4-b]pyridine-4-carboxylic acid moiety, or at both positions using either of synthetic routes A or B.

Route A

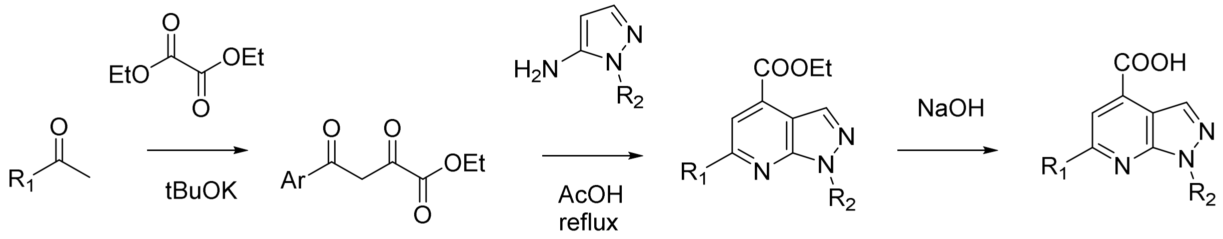

Route B

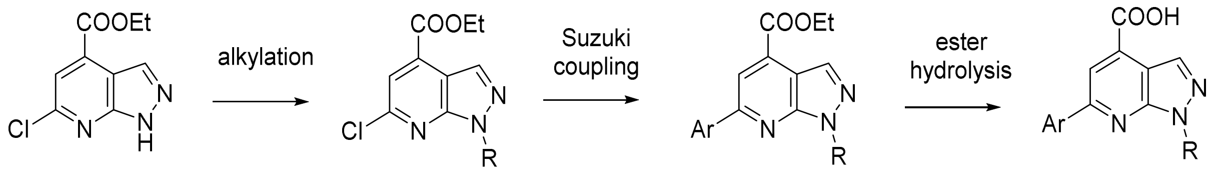

Route A

This is a modified version of the procedures reported in references [22,23]

Step 1: Synthesis of acyl pyruvate

The methylketone substrate (0.2 mmol, 1 equiv) was added to a suspension of tBuOK (1.2 equiv) in toluene (5 mL/mmol) at 0 °C . The mixture was stirred at 0 °C for 15 min. Then diethyl oxalate (1.5 equiv) was added via a syringe, and the resulting mixture was stirred overnight at rt. LCMS showed conversion into the major desired product. The crude was dried and used as such in the next step.

Step 2: Condensation of acyl pyruvate and aminopyrazole

The mixture of acyl pyruvate (1 equiv), aminopyrazole derivative (1 equiv) in AcOH (5 mL/mmol) was stirred at rt for 15 min and then refluxed for 4 hrs. LCMS showed conversion into the major desired product. The solvent was removed and the crude was used as such in the next step.

Step 3: Ester hydrolysis

NaOH (10 equiv) was added to the ester (1 equiv) in a 1:1:1 mixture of THF:MeOH:H_2O (5 mL/mmol). The resulting mixture was stirred at 70 °C for 30 min, after which LCMS showed full conversion of the ester to an acid. After acidifying with 2N HCl (pH 3-4) and removal of the organic solvent, the precipitated product was collected by filtration and dried in vacuo to give the desired product. Yield: 50-77% over 3 steps. The product was used as such for the next step without purification.

Route B

tBuOK (15 mg, 2 equiv) was added to a mixture of ethyl 6-chloro-1H-pyrazolo[3,4-b]pyridine-4-carboxylate (CAS 1426918-16-2, EN300-7431150, 15 mg, 0.066 mmol, 1 equiv) in DMSO (0.3 mL). The mixture was stirred for 30 min before adding the desired alkyl bromide (1.5 equiv). The reaction was stirred overnight at rt. After that, $PdCl_2dppf$ (4.8 mg, 0.1 equiv), $ArB(OH)_2$ (2 equiv), K_2CO_3 (2 equiv) and a mixture of dioxane:H_2O (0.6 mL, 1:1, v/v) was added and the reaction was heated at 100 °C for 2 hrs. LCMS showed full conversion of Suzuki coupling and ester hydrolysis. After acidifying with AcOH, the product was purified by HPLC using 20-50% CH_3CN in H_2O (H_2O + 0.1% formic acid) to afford the desired product. Yield: 10-30% over 3 steps.

After preparation of the desired the 1H-pyrazolo[3,4-b]pyridine-4-carboxylic acid all compounds in compound **25 analogs 3** series were prepared according to **general procedure A.** Yield: 16-51%.

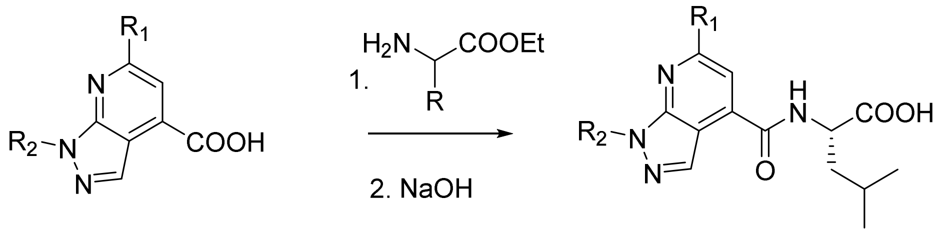

Analytical data of synthesized compounds

Compounds from the virtual screening (compounds 24-39)

Compound 24

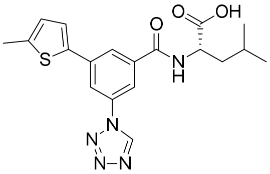

^{1}H NMR (500 MHz, CD_3OD) δ 9.88 (s, 1H), 8.24 – 8.18 (m, 3H), 7.44 (d, *J* = 3.6 Hz, 1H), 6.84 (d, *J* = 3.5 Hz, 1H), 4.71 (dd, *J* = 10.0, 4.2 Hz, 1H), 2.54 (s, 3H), 1.90 – 1.82 (m, 1H), 1.84 – 1.73 (m, 2H), 1.01 (dd, *J* = 10.3, 5.5 Hz, 6H).
^{13}C NMR (126 MHz, CD_3OD) δ 168.3, 143.2, 143.1, 140.1, 138.6, 138.1, 136.3, 128.0, 126.6, 126.0, 121.0, 119.2, 41.3, 26.3, 23.5, 21.7, 15.3.
LCMS (ESI+): calculated for $C_{19}H_{22}N_5O_3S$ $(M+H)^+$: 400.1; found 400.4.

Compound 25

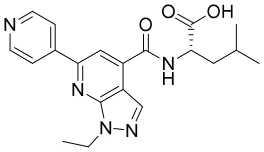

^{1}H NMR (600 MHz, CD_3OD) δ 8.93 – 8.89 (m, 2H), 8.74 – 8.69 (m, 2H), 8.41 (s, 1H), 8.36 (s, 1H), 4.83 – 4.78 (m, 1H), 4.70 (q, *J* = 7.3 Hz, 2H), 1.91 – 1.80 (m, 3H), 1.57 (t, *J* = 7.3 Hz, 3H), 1.04 (d, *J* = 5.3 Hz, 6H).
^{13}C NMR (150 MHz, CD_3OD) δ 175.8, 167.3, 153.7, 152.1, 151.9, 145.9, 138.9, 133.5, 125.1, 115.1, 114.2, 52.8, 43.4, 41.4, 26.3, 23.4, 21.8, 15.2.
LCMS (ESI+): calculated for $C_{20}H_{24}N_5O_3$ $(M+H)^+$: 382.2; found 382.2.

Compound 26

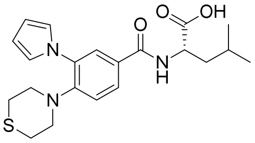

^{1}H NMR (600 MHz, CD_3OD) δ 7.79 (dd, *J* = 8.5, 2.2 Hz, 1H), 7.72 (d, *J* = 2.2 Hz, 1H), 7.15 (d, *J* = 8.5 Hz, 1H), 7.06 (t, *J* = 2.2 Hz, 2H), 6.28 (t, *J* = 2.2 Hz, 2H), 4.65 (dd, *J* = 10.3, 4.3 Hz, 1H), 3.06 – 3.02 (m, 4H), 2.63 – 2.58 (m, 4H), 1.84 – 1.69 (m, 3H), 0.98 (dd, *J* = 14.2, 6.1 Hz, 6H).
^{13}C NMR (150 MHz, CD_3OD) δ 176.3, 169.3, 151.9, 134.8, 129.2, 128.1, 127.3, 122.3, 120.8, 110.5, 53.8, 52.7, 41.4, 28.5, 26.3, 23.4, 21.8.
LCMS (ESI+): calculated for $C_{21}H_{28}N_3O_3S$ $(M+H)^+$: 402.2; found 402.2.

Compound 27

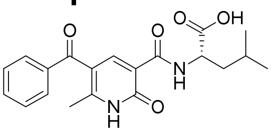

^{1}H NMR (600 MHz, CD_3OD) δ 8.46 (s, 1H), 7.75 – 7.70 (m, 2H), 7.67 – 7.61 (m, 1H), 7.56 – 7.50 (m, 2H), 4.63 – 4.57 (m, 1H), 2.54 (s, 3H), 1.74 (dddd, *J* = 14.7, 12.8, 9.4, 6.5 Hz, 3H), 1.01 – 0.93 (m, 5H).

^{13}C NMR (150 MHz, CD_3OD) δ 195.2, 175.7, 165.3, 163.9, 157.1, 146.7, 139.3, 134.2, 130.7, 129.9, 118.2, 117.0, 52.4, 42.3, 26.3, 23.3, 22.1, 18.7.
LCMS (ESI+): calculated for $C_{20}H_{23}N_2O_5$ $(M+H)^+$: 371.2; found 371.2.

Compound 28

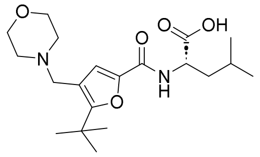

^1H NMR (600 MHz, CD_3OD) δ 7.35 (s, 1H), 4.75 (dd, *J* = 10.6, 4.2 Hz, 1H), 4.54 (s, 2H), 4.13 – 3.47 (m, 8H), 1.99 – 1.91 (m, 1H), 1.90 – 1.80 (m, 2H), 1.60 (s, 9H), 1.09 (dd, *J* = 16.9, 6.2 Hz, 6H).
^{13}C NMR (150 MHz, CD_3OD) δ 175.7, 166.3, 160.2, 146.7, 117.9, 110.4, 65.0, 53.5, 53.2, 51.9, 41.3, 36.1, 30.0, 26.2, 23.4, 21.7.
LCMS (ESI+): calculated for $C_{20}H_{33}N_2O_5$ $(M+H)^+$: 381.2; found 381.2.

Compound 29

Mixture of diastereomers

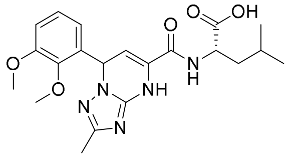

^1H NMR (600 MHz, CD_3OD) δ 7.05 (td, *J* = 7.9, 2.1 Hz, 1H), 7.01 (dd, *J* = 8.3, 1.7 Hz, 1H), 6.68 (dd, *J* = 7.6, 1.7 Hz, 1H), 6.35 (dd, *J* = 4.0, 1.0 Hz, 1H), 5.78 (dd, *J* = 6.3, 3.9 Hz, 1H), 4.54 (ddd, *J* = 10.3, 4.2, 1.6 Hz, 1H), 3.85 (s, 3H), 3.80 (d, *J* = 10.9 Hz, 3H), 2.21 (s, 3H), 1.75 – 1.62 (m, 2H), 1.01 – 0.87 (m, 6H).
^{13}C NMR (150 MHz, CD_3OD) δ 175.8, 163.9, 158.4, 158.3, 154.5, 150.4, 148.3, 148.3, 134.2, 134.2, 130.1, 125.4, 121.0, 114.4, 104.0, 61.2, 57.7, 56.4, 52.5, 41.1, 26.2, 23.3, 21.7, 13.3.
LCMS (ESI+): calculated for $C_{21}H_{28}N_5O_5$ $(M+H)^+$: 430.2; found 430.2.

Compound 30

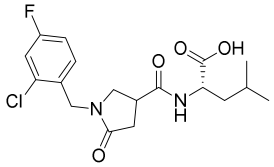

Diastereomer 1

^1H NMR (600 MHz, CD_3OD) δ 7.38 (dd, *J* = 8.6, 6.0 Hz, 1H), 7.25 (dd, *J* = 8.6, 2.6 Hz, 1H), 7.09 (td, *J* = 8.4, 2.6 Hz, 1H), 4.56 (q, *J* = 15.5 Hz, 2H), 4.40 (dd, *J* = 9.9, 5.1 Hz, 1H), 3.58 (dd, *J* = 10.1, 8.8 Hz, 1H), 3.50 (dd, *J* = 10.0, 5.4 Hz, 1H), 3.27 (tdd, *J* = 8.5, 7.5, 5.4 Hz, 1H), 2.70 – 2.65 (m, 2H), 1.75 – 1.57 (m, 3H), 0.97 (d, *J* = 6.5 Hz, 3H), 0.93 (d, *J* = 6.4 Hz, 3H).
^{13}C NMR (151 MHz, CD_3OD) δ 175.9, 175.8, 175.3, 164.3, 162.7, 135.4, 135.3, 132.3, 132.2, 130.9, 118.0, 117.8, 115.6, 115.5, 52.2, 51.1, 44.5, 41.4, 37.5, 35.2, 26.1, 23.3, 21.7.
LCMS (ESI+): calculated for $C_{18}H_{23}ClFN_2O_4$ $(M+H)^+$: 385.1; found 385.2.

Diastereomer 2

^{1}H NMR (600 MHz, CD_3OD) δ 7.40 (dd, *J* = 8.6, 6.0 Hz, 1H), 7.26 (dd, *J* = 8.6, 2.6 Hz, 1H), 7.09 (td, *J* = 8.4, 2.6 Hz, 1H), 4.61 (d, *J* = 15.5 Hz, 1H), 4.51 (d, *J* = 15.4 Hz, 1H), 4.42 – 4.36 (m, 1H), 3.56 (dd, *J* = 10.0, 8.7 Hz, 1H), 3.45 (dd, *J* = 9.9, 5.2 Hz, 1H), 3.31 – 3.23 (m, 1H), 2.71 (d, *J* = 7.9 Hz, 2H), 1.67 – 1.56 (m, 3H), 0.96 – 0.91 (m, 3H), 0.89 (d, *J* = 5.8 Hz, 3H).

^{13}C NMR (151 MHz, CD_3OD) δ 175.8, 175.7, 175.3, 164.3, 162.7, 135.4, 135.4, 132.4, 132.3, 130.9, 118.0, 117.8, 115.6, 115.5, 52.2, 51.0, 44.4, 41.5, 41.3, 37.5, 35.3, 26.1, 23.3, 21.7.

LCMS (ESI+): calculated for $C_{18}H_{23}ClFN_2O_4$ $(M+H)^+$: 385.1; found 385.2.

Compound 31

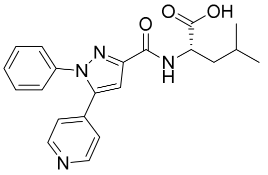

^{1}H NMR (600 MHz, CD_3OD) δ 8.61 – 8.54 (m, 2H), 7.89 (ddd, *J* = 8.1, 2.2, 1.6 Hz, 1H), 7.57 (ddd, *J* = 8.1, 5.2, 0.9 Hz, 1H), 7.50 – 7.43 (m, 3H), 7.43 – 7.37 (m, 2H), 7.21 (s, 1H), 4.72 (dd, *J* = 9.6, 4.6 Hz, 1H), 1.87 – 1.72 (m, 3H), 0.99 (dd, *J* = 6.2, 3.4 Hz, 6H).

^{13}C NMR (151 MHz, CD_3OD) δ 175.8, 163.7, 148.2, 148.1, 147.9, 142.0, 140.5, 140.3, 130.6, 130.3, 128.7, 127.1, 126.0, 109.9, 52.0, 41.7, 26.2, 23.4, 21.9.

LCMS (ESI+): calculated for $C_{21}H_{23}N_4O_3$ $(M+H)^+$: 379.2; found 379.2.

Compound 32

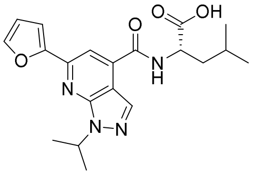

^{1}H NMR (600 MHz, CD_3OD) δ 8.29 (s, 1H), 7.99 (s, 1H), 7.75 (dd, *J* = 1.9, 0.8 Hz, 1H), 7.31 (dd, *J* = 3.4, 0.8 Hz, 1H), 6.67 (dd, *J* = 3.4, 1.7 Hz, 1H), 5.38 (hept, *J* = 6.7 Hz, 1H), 4.76 (dd, *J* = 10.4, 4.2 Hz, 1H), 1.90 – 1.80 (m, 2H), 1.83 – 1.76 (m, 1H), 1.59 (d, *J* = 6.7 Hz, 7H), 1.03 (d, *J* = 5.9 Hz, 6H).

^{13}C NMR (151 MHz, CD_3OD) δ 175.8, 168.1, 154.7, 151.5, 149.8, 145.8, 138.1, 133.2, 113.5, 112.9, 111.9, 111.6, 52.7, 50.0, 41.3, 26.4, 23.4, 22.3, 21.8.

LCMS (ESI+): calculated for $C_{20}H_{25}N_4O_4$ $(M+H)^+$: 385.2; found 385.2.

Compound 33

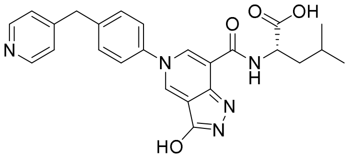

^{1}H NMR (600 MHz, CD_3OD) δ 8.79 (d, *J* = 1.6 Hz, 1H), 8.74 – 8.70 (m, 2H), 8.36 (d, *J* = 1.6 Hz, 1H), 7.92 – 7.87 (m, 2H), 7.70 – 7.65 (m, 2H), 7.61 – 7.56 (m, 2H), 4.74 – 4.69 (m, 1H), 4.42 (s, 2H), 1.81 (tdt, *J* = 15.6, 9.0, 4.2 Hz, 3H), 1.00 (dd, *J* = 13.1, 6.0 Hz, 6H).

^{13}C NMR (151 MHz, CD_3OD) δ 175.4, 165.5, 164.5, 162.1, 143.9, 143.8, 143.5, 142.1, 140.5, 137.9, 132.4, 128.3, 125.6, 116.1, 115.7, 52.8, 42.2, 41.7, 26.3, 23.3, 22.1.
LCMS (ESI+): calculated for $C_{25}H_{26}N_5O_4$ $(M+H)^+$: 460.2; found 460.2.

Compound 34

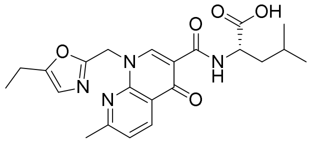

^1H NMR (600 MHz, CD_3OD) δ 9.07 (s, 1H), 8.61 (d, *J* = 8.2 Hz, 1H), 7.44 (d, *J* = 8.2 Hz, 1H), 6.71 (t, *J* = 1.2 Hz, 1H), 5.91 – 5.83 (m, 2H), 4.69 (dd, *J* = 8.3, 5.8 Hz, 1H), 2.67 (qd, *J* = 7.6, 1.2 Hz, 2H), 2.63 (s, 3H), 1.88 – 1.73 (m, 3H), 1.21 (t, *J* = 7.6 Hz, 3H), 1.00 (dd, *J* = 16.1, 6.2 Hz, 6H).
^{13}C NMR (151 MHz, CD_3OD) δ 178.7, 175.8, 166.1, 165.6, 159.6, 157.2, 150.2, 149.9, 137.2, 123.0, 122.5, 121.2, 113.6, 52.4, 52.3, 42.4, 42.2, 26.3, 25.0, 23.4, 23.3, 22.2, 22.1, 19.7, 12.1.
LCMS (ESI+): calculated for $C_{22}H_{27}N_4O_5$ $(M+H)^+$: 427.2; found 427.2.

Compound 35

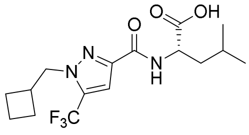

^1H NMR (600 MHz, CD_3OD) δ 7.14 (s, 1H), 4.70 – 4.64 (m, 1H), 4.32 (d, *J* = 7.4 Hz, 2H), 2.96 (hept, *J* = 7.8 Hz, 1H), 2.07 (tdt, *J* = 10.6, 5.8, 3.0 Hz, 2H), 2.00 – 1.84 (m, 3H), 1.89 (s, 1H), 1.84 – 1.76 (m, 1H), 1.78 – 1.73 (m, 1H), 1.76 – 1.69 (m, 1H), 0.98 (dd, *J* = 10.9, 5.9 Hz, 6H).
^{13}C NMR (151 MHz, CD_3OD) δ 175.7, 162.9, 146.3, 134.8, 134.5, 134.3, 134.0, 123.8, 122.0, 120.3, 118.5, 108.8, 57.6, 52.0, 41.7, 36.8, 26.7, 26.2, 23.4, 21.9, 18.9.
LCMS (ESI+): calculated for $C_{16}H_{13}F_3N_3O_3$ $(M+H)^+$: 362.2; found 362.2.

Compound 36

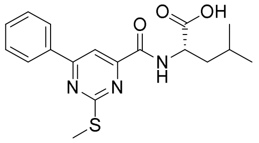

^1H NMR (600 MHz, CD_3OD) δ 8.21 – 8.16 (m, 2H), 8.12 (s, 1H), 7.58 – 7.49 (m, 3H), 4.71 (dd, *J* = 9.3, 5.0 Hz, 1H), 2.68 (s, 3H), 1.90 – 1.72 (m, 3H), 1.00 (dd, *J* = 6.4, 4.3 Hz, 7H).
^{13}C NMR (151 MHz, CD_3OD) δ 175.3, 174.1, 167.5, 164.8, 158.6, 137.1, 132.9, 130.1, 128.5, 109.9, 52.4, 41.9, 30.7, 26.3, 23.3, 22.1, 14.4.
LCMS (ESI+): calculated for $C_{18}H_{22}N_3O_3S$ $(M+H)^+$: 360.2; found 360.2.

Compound 37

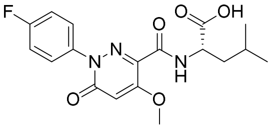

^{1}H NMR (601 MHz, CD_3OD) δ 7.60 (ddd, *J* = 9.2, 4.8, 2.3 Hz, 2H), 7.23 (td, *J* = 8.8, 2.5 Hz, 2H), 6.47 (d, *J* = 1.4 Hz, 1H), 4.66 – 4.58 (m, 1H), 3.95 (s, 3H), 1.81 – 1.70 (m, 2H), 1.73 – 1.65 (m, 1H), 1.00 – 0.94 (m, 6H).
^{13}C NMR (151 MHz, CD_3OD) δ 175.3, 174.0, 164.4, 163.8, 163.7, 163.5, 162.8, 161.0, 160.9, 138.6, 138.6, 138.4, 129.1, 129.1, 116.6, 116.5, 105.4, 57.3, 52.5, 52.4, 41.7, 41.5, 26.1, 26.1, 23.3, 23.2, 21.9, 21.9.
LCMS (ESI+): calculated for $C_{18}H_{21}FN_3O_5$ $(M+H)^+$: 378.2; found 378.2.

Compound 38

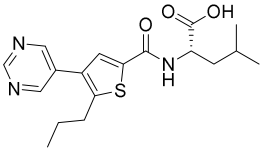

^{1}H NMR (600 MHz, CD_3OD) δ 9.15 (s, 1H), 8.88 (s, 2H), 7.81 (s, 1H), 4.63 (dd, *J* = 10.5, 4.2 Hz, 1H), 2.93 – 2.87 (m, 2H), 1.83 – 1.72 (m, 2H), 1.75 (s, 1H), 1.74 – 1.67 (m, 2H), 1.02 – 0.94 (m, 8H).
^{13}C NMR (151 MHz, CD_3OD) δ 176.0, 164.0, 157.9, 157.5, 150.5, 137.2, 133.0, 132.0, 131.0, 52.5, 41.4, 31.6, 26.2, 26.1, 23.4, 21.7, 14.0.
LCMS (ESI+): calculated for $C_{18}H_{24}N_3O_3S$ $(M+H)^+$: 362.2; found 362.2.

Compound 39

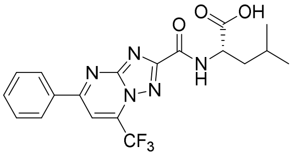

^{1}H NMR (600 MHz, CD_3OD) δ 8.39 (dd, *J* = 8.3, 1.4 Hz, 3H), 7.69 – 7.60 (m, 3H), 4.76 (dd, *J* = 9.5, 4.1 Hz, 1H), 1.88 (dd, *J* = 9.7, 8.1 Hz, 1H), 1.86 – 1.79 (m, 2H), 1.02 (dd, *J* = 6.1, 3.3 Hz, 6H).
LCMS (ESI+): calculated for $C_{19}H_{19}F_3N_5O_3$ $(M+H)^+$: 422.1; found 422.2.

Optimization of compound 24 (Scaffold a)

Compound 1a

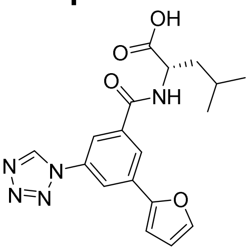

^{1}H NMR (500 MHz, CD_3OD) δ 9.90 (s, 1H), 8.38 – 8.34 (m, 2H), 8.24 (t, *J* = 1.9 Hz, 1H), 7.69 (d, *J* = 1.9 Hz, 1H), 7.09 (d, *J* = 3.4 Hz, 1H), 6.62 (dd, *J* = 3.5, 1.8 Hz, 1H), 4.72 (dd, *J* = 10.4, 4.1 Hz, 1H), 1.87 (td, *J* = 11.4, 3.5 Hz, 1H), 1.80 (ddt, *J* = 15.1, 11.0, 5.1 Hz, 2H), 1.02 (dd, *J* = 10.3, 5.7 Hz, 6H).

^{13}C NMR (125 MHz, CD_3OD) δ 176.0, 168.3, 152.8, 145.0, 143.2, 138.1, 136.3, 134.6, 124.4, 119.6, 119.5, 113.3, 109.2, 41.3, 26.4, 26.3, 23.4, 21.7.
LCMS (ESI+): calculated for $C_{18}H_{20}N_5O_4$ $(M+H)^+$: 370.2; found 370.5.

Compound 2a

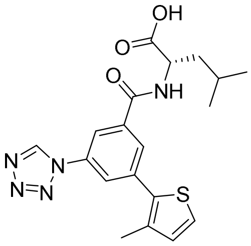

^1H NMR (500 MHz, CD_3OD) δ 9.89 (s, 1H), 8.33 (t, *J* = 1.8 Hz, 1H), 8.14 (dt, *J* = 9.4, 1.7 Hz, 2H), 7.43 (d, *J* = 5.1 Hz, 1H), 7.02 (d, *J* = 5.1 Hz, 1H), 4.71 (dd, *J* = 10.3, 4.1 Hz, 1H), 2.40 (s, 3H), 1.89 – 1.72 (m, 3H), 1.01 (dd, *J* = 8.6, 5.8 Hz, 6H).
^{13}C NMR (125 MHz, CD_3OD) δ 176.0, 168.3, 143.2, 138.9, 137.8, 136.5, 135.9, 135.9, 132.6, 129.9, 126.2, 124.9, 119.7, 41.2, 26.3, 23.4, 21.7, 15.0.
LCMS (ESI+): calculated for $C_{19}H_{22}N_5O_3S$ $(M+H)^+$: 400.1; found 400.0.

Compound 3a

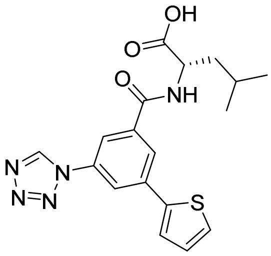

^1H NMR (500 MHz, CD_3OD) δ 9.92 (s, 1H), 8.32 (dt, *J* = 12.2, 1.7 Hz, 2H), 8.28 (t, *J* = 1.7 Hz, 1H), 7.69 (d, *J* = 3.6 Hz, 1H), 7.56 (d, *J* = 5.0 Hz, 1H), 7.19 (dd, *J* = 5.1, 3.6 Hz, 1H), 4.72 (d, *J* = 8.8 Hz, 1H), 1.90 – 1.83 (m, 1H), 1.80 (h, *J* = 4.6 Hz, 2H), 1.02 (dd, *J* = 9.3, 5.5 Hz, 6H).
^{13}C NMR (125 MHz, CD_3OD) δ 168.2, 143.2, 142.5, 138.4, 138.2, 136.4, 129.6, 128.1, 126.7, 126.6, 121.6, 119.7, 41.3, 26.3, 23.5, 21.8.
LCMS (ESI+): calculated for $C_{18}H_{20}N_5O_3S$ $(M+H)^+$: 386.1; found 386.2.

Compound 4a

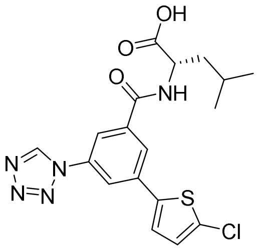

^1H NMR (500 MHz, CD_3OD) δ 9.91 (s, 1H), 8.32 – 8.25 (m, 2H), 8.22 (s, 1H), 7.52 (d, *J* = 4.0 Hz, 1H), 7.09 (d, *J* = 3.9 Hz, 1H), 4.71 (d, *J* = 8.9 Hz, 1H), 1.85 (q, *J* = 10.0 Hz, 1H), 1.81 – 1.76 (m, 2H), 1.01 (dd, *J* = 10.5, 5.2 Hz, 6H).
^{13}C NMR (125 MHz, CD_3OD) δ 168.0, 143.2, 141.3, 138.3, 137.3, 136.5, 132.1, 129.2, 126.3, 126.2, 121.3, 120.2, 41.3, 26.3, 23.5, 21.8.
LCMS (ESI+): calculated for $C_{18}H_{19}ClN_5O_3S$ $(M+H)^+$: 420.1; found 419.9.

Compound 5a

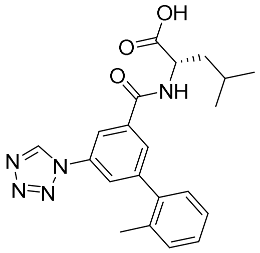

^{1}H NMR (500 MHz, CD_3OD) δ 9.87 (s, 1H), 8.39 (t, *J* = 2.0 Hz, 1H), 8.06 – 7.99 (m, 2H), 7.36 – 7.26 (m, 4H), 4.71 (dd, *J* = 10.3, 4.0 Hz, 1H), 2.32 (s, 3H), 1.88 – 1.71 (m, 3H), 1.00 (t, *J* = 6.5 Hz, 6H).
^{13}C NMR (125 MHz, CD_3OD) δ 176.0, 168.4, 146.0, 143.2, 140.7, 137.4, 136.5, 135.6, 131.7, 130.7, 130.5, 129.6, 127.3, 125.7, 119.8, 41.2, 26.3, 23.4, 21.7, 20.5.
LCMS (ESI+): calculated for $C_{21}H_{24}N_5O_3$ $(M+H)^+$: 394.2; found 394.1.

Compound 6a

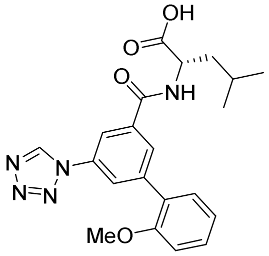

^{1}H NMR (500 MHz, CD_3OD) δ 9.85 (s, 1H), 8.30 (t, *J* = 1.8 Hz, 1H), 8.19 (dt, *J* = 16.7, 1.7 Hz, 2H), 7.48 – 7.38 (m, 2H), 7.14 (d, *J* = 8.3 Hz, 1H), 7.08 (t, *J* = 7.5 Hz, 1H), 4.71 (dd, *J* = 10.4, 4.1 Hz, 1H), 3.85 (s, 3H), 1.89 – 1.72 (m, 3H), 1.01 (dd, *J* = 7.6, 5.9 Hz, 6H).
^{13}C NMR (125 MHz, CD_3OD) δ 176.0, 168.6, 157.9, 143.2, 142.7, 137.1, 135.3, 131.7, 131.3, 130.8, 129.1, 126.1, 122.2, 119.6, 112.7, 56.1, 41.3, 26.3, 23.4, 21.8.
LCMS (ESI+): calculated for $C_{21}H_{24}N_5O_4$ $(M+H)^+$: 410.2; found 410.1.

Compound 7a

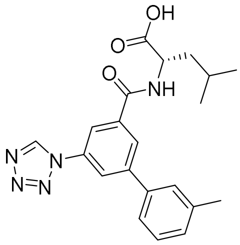

^{1}H NMR (500 MHz, CD_3OD) δ 9.91 (s, 1H), 8.33 (d, *J* = 2.3 Hz, 1H), 8.29 (s, 2H), 7.62 (s, 1H), 7.57 (d, *J* = 7.7 Hz, 1H), 7.40 (t, *J* = 7.7 Hz, 1H), 7.27 (d, *J* = 7.5 Hz, 1H), 4.73 (dd, *J* = 10.4, 4.2 Hz, 1H), 2.45 (s, 3H), 1.91 – 1.82 (m, 1H), 1.79 (dddd, *J* = 16.1, 12.2, 7.7, 4.6 Hz, 2H), 1.01 (dd, *J* = 8.8, 5.7 Hz, 6H).
^{13}C NMR (125 MHz, CD_3OD) δ 176.1, 168.5, 145.2, 143.2, 140.2, 139.8, 137.8, 136.2, 130.5, 130.2, 128.9, 128.0, 125.4, 123.3, 119.9, 41.3, 26.3, 23.4, 21.7, 21.5.
LCMS (ESI+): calculated for $C_{21}H_{24}N_5O_3$ $(M+H)^+$: 394.2; found 394.4.

Compound 8a

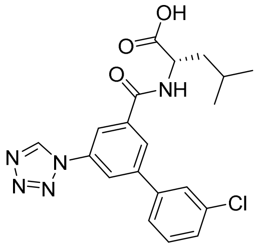

^{1}H NMR (500 MHz, CD_3OD) δ 9.94 (s, 1H), 8.40 (s, 1H), 8.36 – 8.30 (m, 2H), 7.88 (t, *J* = 1.9 Hz, 1H), 7.75 (d, *J* = 7.6 Hz, 1H), 7.52 (t, *J* = 7.8 Hz, 1H), 7.49 – 7.44 (m, 1H), 4.77 – 4.70 (m, 1H), 1.91 – 1.74 (m, 3H), 1.01 (dd, *J* = 9.4, 5.5 Hz, 6H).
^{13}C NMR (125 MHz, CD_3OD) δ 168.3, 143.4, 143.3, 141.9, 138.1, 136.4, 136.2, 131.8, 129.7, 128.4, 128.1, 126.8, 123.4, 120.7, 41.3, 26.3, 23.4, 21.7.
LCMS (ESI+): calculated for $C_{20}H_{21}ClN_5O_3$ $(M+H)^+$: 414.1; found 414.3.

Compound 9a

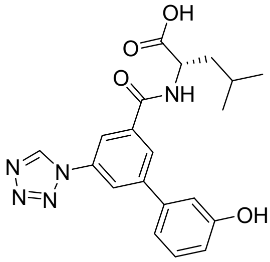

^{1}H NMR (500 MHz, CD_3OD) δ 9.91 (s, 1H), 8.33 (s, 1H), 8.27 (d, *J* = 1.7 Hz, 2H), 7.33 (t, *J* = 7.8 Hz, 1H), 7.25 (d, *J* = 7.7 Hz, 1H), 7.19 (d, *J* = 2.2 Hz, 1H), 6.87 (dd, *J* = 8.1, 2.3 Hz, 1H), 4.76 – 4.70 (m, 1H), 1.90 – 1.82 (m, 1H), 1.80 (ddt, *J* = 14.9, 9.7, 4.5 Hz, 2H), 1.01 (dd, *J* = 8.4, 5.7 Hz, 6H).
^{13}C NMR (125 MHz, CD_3OD) δ 168.5, 159.4, 145.1, 143.2, 141.2, 137.9, 136.1, 131.3, 128.1, 123.3, 120.0, 119.4, 116.7, 115.0, 41.3, 26.3, 23.5, 21.7.
LCMS (ESI+): calculated for $C_{20}H_{22}N_5O_4$ $(M+H)^+$: 396.2; found 396.0.

Compound 10a

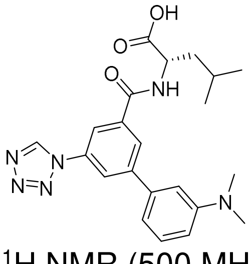

^{1}H NMR (500 MHz, CD_3OD) δ 9.98 (d, *J* = 3.0 Hz, 1H), 8.46 (t, *J* = 2.0 Hz, 2H), 8.39 (q, *J* = 1.6 Hz, 1H), 8.16 (dt, *J* = 4.0, 1.9 Hz, 1H), 8.05 (dt, *J* = 7.4, 1.4 Hz, 1H), 7.83 – 7.73 (m, 2H), 4.75 (ddd, *J* = 10.1, 8.1, 4.3 Hz, 1H), 3.43 (s, 6H), 1.92 – 1.72 (m, 3H), 1.05 – 0.90 (m, 7H).
^{13}C NMR (125 MHz, CD_3OD) δ 176.0, 174.7, 168.2, 144.8, 143.3, 142.8, 142.7, 142.6, 142.6, 138.3, 138.1, 136.5, 136.4, 132.7, 130.5, 128.5, 123.9, 123.8, 121.9, 121.8, 121.1, 120.8, 120.7, 41.3, 41.1, 26.3, 26.2, 23.4, 23.3, 21.8, 21.7.
LCMS (ESI+): calculated for $C_{22}H_{27}N_6O_3$ $(M+H)^+$: 423.2; found 423.6.

Compound 11a

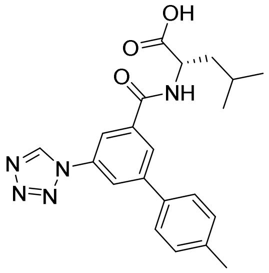

^1H NMR (500 MHz, CD_3OD) δ 9.90 (s, 1H), 8.29 (d, *J* = 13.7 Hz, 3H), 7.68 (d, *J* = 7.8 Hz, 2H), 7.33 (d, *J* = 7.8 Hz, 2H), 4.73 (dd, *J* = 10.3, 4.1 Hz, 1H), 3.31 (s, 3H), 2.40 (s, 3H), 1.87 (td, *J* = 11.4, 3.5 Hz, 1H), 1.80 (ddd, *J* = 15.7, 9.4, 4.8 Hz, 2H), 1.01 (dd, *J* = 9.0, 5.7 Hz, 6H).
^{13}C NMR (125 MHz, CD_3OD) δ 168.6, 145.0, 143.2, 140.0, 137.9, 136.9, 136.2, 130.9, 128.1, 127.8, 123.0, 119.7, 41.3, 26.3, 23.4, 21.7, 21.2.
LCMS (ESI+): calculated for $C_{21}H_{24}N_5O_3$ $(M+H)^+$: 394.2; found 394.4.

Compound 12a

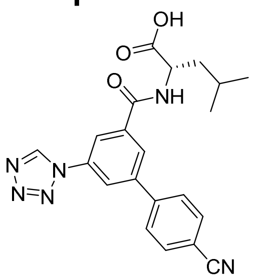

^1H NMR (500 MHz, CD_3OD) δ 9.94 (s, 1H), 8.47 – 8.39 (m, 2H), 8.38 (d, *J* = 1.7 Hz, 1H), 8.02 (d, *J* = 8.2 Hz, 2H), 7.91 (d, *J* = 8.2 Hz, 2H), 4.76 – 4.70 (m, 1H), 1.90 – 1.75 (m, 3H), 1.01 (dd, *J* = 8.0, 5.6 Hz, 6H).
^{13}C NMR (125 MHz, CD_3OD) δ 168.1, 144.4, 143.3, 142.9, 138.3, 136.5, 134.1, 129.4, 128.3, 123.7, 121.3, 119.4, 113.4, 41.4, 26.3, 23.5, 21.8.
LCMS (ESI+): calculated for $C_{21}H_{21}N_6O_3$ $(M+H)^+$: 405.2; found 405.1.

Compound 13a

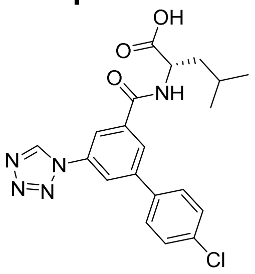

^1H NMR (500 MHz, CD_3OD) δ 9.92 (s, 1H), 8.37 (d, *J* = 1.6 Hz, 1H), 8.35 – 8.28 (m, 2H), 7.80 (d, *J* = 8.3 Hz, 2H), 7.53 (d, *J* = 8.2 Hz, 2H), 4.76 – 4.70 (m, 1H), 1.86 (dd, *J* = 15.3, 6.4 Hz, 1H), 1.79 (dp, *J* = 9.3, 5.3 Hz, 2H), 1.01 (dd, *J* = 9.2, 5.5 Hz, 6H).
^{13}C NMR (125 MHz, CD_3OD) δ 168.3, 143.7, 143.3, 138.5, 138.1, 136.3, 136.0, 130.4, 129.9, 128.0, 123.3, 120.4, 41.3, 26.3, 23.5, 21.7.
LCMS (ESI+): calculated for $C_{20}H_{21}ClN_5O_3$ $(M+H)^+$: 414.1; found 414.3.

Compound 14a

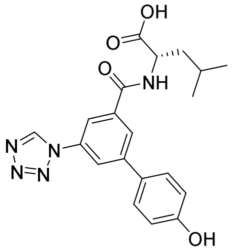

^{1}H NMR (500 MHz, CD_3OD) δ 9.89 (s, 1H), 8.27 – 8.21 (m, 3H), 7.65 (d, *J* = 8.3 Hz, 2H), 6.92 (d, *J* = 8.2 Hz, 2H), 4.72 (dd, *J* = 10.1, 4.2 Hz, 1H), 1.91 – 1.82 (m, 1H), 1.80 (ddt, *J* = 15.0, 10.9, 5.1 Hz, 2H), 1.01 (dd, *J* = 8.8, 5.7 Hz, 6H).
^{13}C NMR (125 MHz, CD_3OD) δ 176.1, 168.7, 159.6, 145.0, 143.2, 137.8, 136.2, 130.9, 129.5, 127.3, 122.6, 119.0, 117.0, 41.3, 26.3, 23.5, 21.7.
LCMS (ESI+): calculated for $C_{20}H_{22}N_5O_4$ $(M+H)^+$: 396.2; found 396.1.

Compound 15a

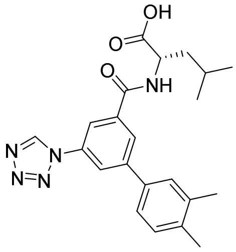

^{1}H NMR (500 MHz, CD_3OD) δ 9.90 (s, 1H), 8.32 – 8.24 (m, 3H), 7.57 (d, *J* = 2.0 Hz, 1H), 7.50 (dd, *J* = 7.9, 2.0 Hz, 1H), 7.27 (d, *J* = 7.8 Hz, 1H), 4.73 (dd, *J* = 10.2, 4.0 Hz, 1H), 2.37 (s, 3H), 2.33 (s, 3H), 1.91 – 1.74 (m, 3H), 1.01 (dd, *J* = 8.9, 5.7 Hz, 6H).
^{13}C NMR (125 MHz, CD_3OD) δ 168.6, 145.1, 143.2, 138.6, 138.6, 137.8, 137.3, 136.2, 131.4, 129.3, 127.8, 125.6, 123.0, 119.6, 41.3, 26.3, 23.5, 21.8, 19.9, 19.6.
LCMS (ESI+): calculated for $C_{22}H_{26}N_5O_3$ $(M+H)^+$: 408.2; found 408.3.

Compound 16a

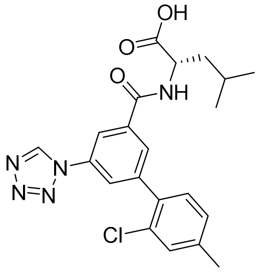

^{1}H NMR (500 MHz, CD_3OD) δ 9.87 (s, 1H), 8.40 (t, *J* = 1.9 Hz, 1H), 8.14 (d, *J* = 1.8 Hz, 1H), 8.10 (d, *J* = 1.6 Hz, 1H), 7.44 – 7.39 (m, 2H), 7.28 (d, *J* = 7.9 Hz, 1H), 4.74 – 4.68 (m, 1H), 2.41 (s, 3H), 1.88 – 1.72 (m, 3H), 1.00 (t, *J* = 6.6 Hz, 6H).
^{13}C NMR (125 MHz, CD_3OD) δ 166.8, 141.8, 141.8, 140.5, 136.0, 135.0, 134.1, 131.6, 130.9, 130.2, 129.4, 128.0, 124.7, 118.9, 39.9, 24.9, 22.0, 20.3, 19.5.
LCMS (ESI+): calculated for $C_{21}H_{23}ClN_5O_3$ $(M+H)^+$: 428.2; found 428.3.

Compound 17a

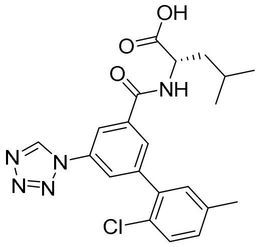

^{1}H NMR (500 MHz, CD_3OD) δ 9.88 (s, 1H), 8.42 (t, J = 1.9 Hz, 1H), 8.15 (t, J = 1.9 Hz, 1H), 8.11 (d, J = 1.6 Hz, 1H), 7.44 (d, J = 8.2 Hz, 1H), 7.37 (d, J = 2.3 Hz, 1H), 7.26 (dd, J = 8.3, 2.2 Hz, 1H), 4.71 (dd, J = 10.2, 4.0 Hz, 1H), 2.41 (s, 3H), 1.89 – 1.80 (m, 1H), 1.78 (s, 2H), 1.00 (t, J = 6.5 Hz, 6H).
^{13}C NMR (125 MHz, CD_3OD) δ 168.2, 143.4, 143.2, 139.1, 138.9, 137.4, 135.5, 133.0, 131.8, 131.0, 130.8, 130.2, 126.0, 120.4, 41.3, 26.3, 23.4, 21.7, 20.8.
LCMS (ESI+): calculated for $C_{21}H_{23}ClN_5O_3$ $(M+H)^+$: 428.2; found 428.4.

Compound 18a

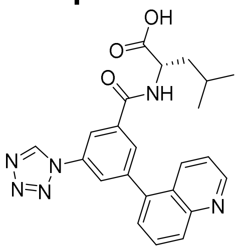

^{1}H NMR (500 MHz, CD_3OD) δ 9.91 (s, 1H), 8.95 – 8.91 (m, 1H), 8.53 (t, J = 1.8 Hz, 1H), 8.39 (d, J = 8.5 Hz, 1H), 8.23 (t, J = 1.8 Hz, 1H), 8.19 – 8.14 (m, 2H), 7.92 (dd, J = 8.5, 7.1 Hz, 1H), 7.75 (d, J = 7.0 Hz, 1H), 7.59 (dd, J = 8.6, 4.2 Hz, 1H), 4.72 (dd, J = 10.5, 3.9 Hz, 1H), 1.88 – 1.79 (m, 1H), 1.78 (ddt, J = 12.8, 8.4, 4.6 Hz, 2H), 1.00 (dd, J = 6.0, 3.7 Hz, 6H).
^{13}C NMR (125 MHz, CD_3OD) δ 176.1, 168.2, 151.5, 149.0, 143.2, 143.0, 139.5, 137.8, 136.0, 136.0, 131.1, 130.7, 130.0, 129.4, 127.8, 126.4, 123.3, 120.8, 41.3, 26.3, 23.4, 21.7.
LCMS (ESI+): calculated for $C_{23}H_{23}N_6O_3$ $(M+H)^+$: 431.2; found 431.5.

Compound 19a

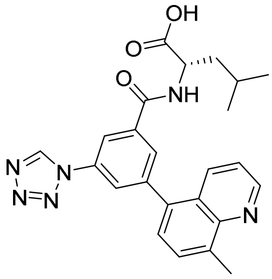

^{1}H NMR (600 MHz, CD_3OD) δ 9.90 (s, 1H), 9.08 (dd, J = 4.8, 1.6 Hz, 1H), 8.75 (dd, J = 8.6, 1.7 Hz, 1H), 8.54 (t, J = 1.8 Hz, 1H), 8.24 (t, J = 1.8 Hz, 1H), 8.17 (t, J = 1.5 Hz, 1H), 7.95 (dd, J = 7.4, 1.2 Hz, 1H), 7.86 – 7.79 (m, 2H), 4.72 (dd, J = 10.2, 4.2 Hz, 1H), 2.91 (s, 3H), 1.87 – 1.73 (m, 3H), 1.00 (t, J = 6.3 Hz, 6H).
^{13}C NMR (150 MHz, CD_3OD) δ 175.9, 168.1, 148.4, 143.9, 143.2, 142.2, 141.2, 138.1, 138.0, 136.1, 135.4, 133.5, 131.3, 130.5, 128.4, 126.6, 123.1, 121.0, 52.9, 41.3, 26.3, 23.4, 21.7, 18.0.
LCMS (ESI+): calculated for $C_{24}H_{25}N_6O_3$ $(M+H)^+$: 445.2; found 445.4.

Compound 20a

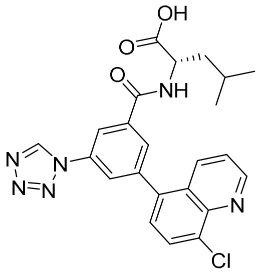

^{1}H NMR (600 MHz, CD_3OD) δ 9.90 (s, 1H), 9.03 (dd, *J* = 4.2, 1.6 Hz, 1H), 8.54 (t, *J* = 1.8 Hz, 1H), 8.43 (dd, *J* = 8.6, 1.6 Hz, 1H), 8.23 (t, *J* = 1.8 Hz, 1H), 8.16 (t, *J* = 1.6 Hz, 1H), 8.06 (d, *J* = 7.7 Hz, 1H), 7.73 – 7.65 (m, 2H), 4.72 (dd, *J* = 10.3, 4.2 Hz, 1H), 1.88 – 1.77 (m, 2H), 1.79 – 1.73 (m, 1H), 0.99 (t, *J* = 5.8 Hz, 6H).
^{13}C NMR (150 MHz, CD_3OD) δ 175.9, 168.1, 152.0, 145.0, 143.2, 142.2, 138.8, 137.9, 136.8, 136.1, 134.4, 131.1, 130.8, 129.3, 129.2, 126.5, 124.0, 121.0, 52.9, 41.3, 26.3, 23.4, 21.7.
LCMS (ESI+): calculated for $C_{23}H_{22}ClN_6O_3$ $(M+H)^+$: 465.1; found 465.0.

Compound 21a

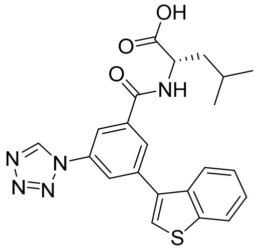

^{1}H NMR (500 MHz, CD_3OD) δ 9.91 (s, 1H), 8.41 (t, *J* = 1.8 Hz, 1H), 8.29 (dt, *J* = 5.0, 1.6 Hz, 2H), 7.99 (dt, *J* = 7.4, 1.7 Hz, 2H), 7.85 (s, 1H), 7.45 (pd, *J* = 7.1, 1.4 Hz, 2H), 4.73 (dd, *J* = 10.5, 4.1 Hz, 1H), 1.86 (td, *J* = 10.9, 3.2 Hz, 1H), 1.84 – 1.73 (m, 2H), 1.01 (t, *J* = 5.7 Hz, 6H).
^{13}C NMR (125 MHz, CD_3OD) δ 176.1, 168.4, 143.2, 142.2, 139.8, 138.3, 138.0, 136.5, 136.1, 129.7, 127.2, 126.0, 126.0, 124.8, 124.1, 123.3, 120.2, 41.3, 26.3, 23.4, 21.8.
LCMS (ESI+): calculated for $C_{22}H_{22}N_5O_3S$ $(M+H)^+$: 436.1; found 436.1.

Compound 22a

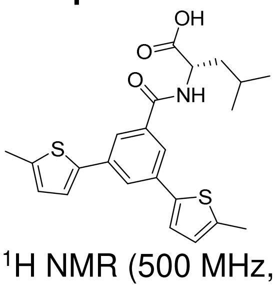

^{1}H NMR (500 MHz, CD_3OD) δ 7.92 (d, *J* = 1.7 Hz, 2H), 7.84 (t, *J* = 1.8 Hz, 1H), 7.31 (d, *J* = 3.5 Hz, 2H), 6.80 (d, *J* = 3.6 Hz, 2H), 4.70 (dd, *J* = 10.5, 4.1 Hz, 1H), 2.52 (s, 6H), 1.91 – 1.72 (m, 3H), 1.01 (dd, *J* = 9.9, 5.9 Hz, 6H).
^{13}C NMR (125 MHz, CD_3OD) δ 169.9, 141.7, 141.7, 137.1, 136.8, 127.7, 125.6, 125.3, 123.6, 41.2, 26.4, 23.5, 21.8, 15.3.
LCMS (ESI+): calculated for $C_{23}H_{26}NO_3S_2$ $(M+H)^+$: 428.1; found 428.2.

Compound 23a

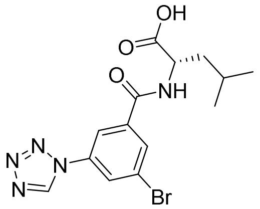

^{1}H NMR (500 MHz, CD_3OD) δ 9.86 (s, 1H), 8.38 (d, *J* = 1.8 Hz, 1H), 8.33 (d, *J* = 2.0 Hz, 1H), 8.22 (d, *J* = 1.6 Hz, 1H), 4.71 – 4.64 (m, 1H), 1.87 – 1.81 (m, 1H), 1.77 (dt, *J* = 9.9, 6.0 Hz, 2H), 1.00 (dd, *J* = 12.1, 5.2 Hz, 6H).
^{13}C NMR (125 MHz, CD_3OD) δ 167.0, 143.2, 139.0, 136.7, 132.6, 127.9, 124.5, 120.3, 41.3, 26.3, 23.4, 21.7.
LCMS (ESI+): calculated for $C_{14}H_{17}BrN_5O_3$ $(M+H)^+$: 382.0; found 382.1.

Compound 24a

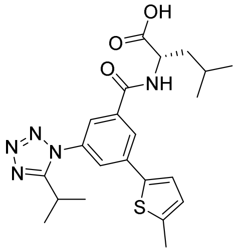

^{1}H NMR (500 MHz, CD_3OD) δ 8.33 (t, *J* = 1.6 Hz, 1H), 7.96 (t, *J* = 1.8 Hz, 1H), 7.87 (t, *J* = 1.8 Hz, 1H), 7.44 (d, *J* = 3.6 Hz, 1H), 6.85 (dd, *J* = 3.8, 1.2 Hz, 1H), 4.69 (dd, *J* = 10.4, 4.1 Hz, 1H), 2.54 (d, *J* = 1.2 Hz, 3H), 1.88 – 1.79 (m, 1H), 1.78 (tq, *J* = 8.4, 2.8 Hz, 2H), 1.38 (dd, *J* = 6.9, 2.6 Hz, 6H), 1.00 (dd, *J* = 8.6, 5.8 Hz, 6H).
^{13}C NMR (125 MHz, CD_3OD) δ 176.1, 168.1, 161.8, 143.3, 139.8, 138.6, 138.0, 136.1, 128.1, 127.0, 126.8, 125.7, 123.6, 53.0, 41.2, 26.3, 25.4, 23.4, 21.7, 21.3, 21.3, 15.3.
LCMS (ESI+): calculated for $C_{22}H_{28}N_5O_3S$ $(M+H)^+$: 442.2; found 442.4.

Compound 25a

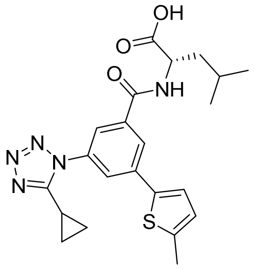

^{1}H NMR (500 MHz, CD_3OD) δ 8.97 (d, *J* = 7.8 Hz, 1H), 8.30 (d, *J* = 1.7 Hz, 1H), 8.04 (d, *J* = 1.9 Hz, 1H), 8.00 (d, *J* = 1.8 Hz, 1H), 7.44 (d, *J* = 3.6 Hz, 1H), 6.85 (dd, *J* = 3.6, 1.3 Hz, 1H), 4.74 – 4.66 (m, 1H), 2.54 (s, 3H), 2.14 (tt, *J* = 8.1, 5.3 Hz, 1H), 1.89 – 1.81 (m, 1H), 1.78 (tt, *J* = 11.5, 5.1 Hz, 2H), 1.32 – 1.22 (m, 4H), 1.01 (dd, *J* = 9.5, 5.7 Hz, 6H).
^{13}C NMR (125 MHz, CD_3OD) δ 168.4, 168.3, 159.1, 143.2, 140.0, 138.5, 138.0, 138.0, 136.1, 128.1, 126.7, 126.6, 125.1, 123.0, 53.0, 41.2, 41.2, 26.3, 23.4, 21.7, 15.3, 10.2, 10.2, 5.7.
LCMS (ESI+): calculated for $C_{22}H_{26}N_5O_3S$ $(M+H)^+$: 440.2; found 440.4.

Compound 26a

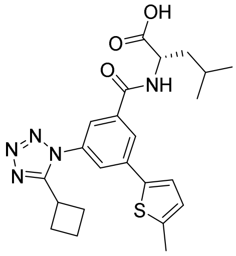

^{1}H NMR (500 MHz, CD_3OD) δ 8.29 (s, 1H), 7.87 (d, J = 1.5 Hz, 1H), 7.82 (s, 1H), 7.43 (d, J = 3.6 Hz, 1H), 6.85 (dd, J = 3.6, 1.3 Hz, 1H), 4.71 (s, 1H), 3.86 (p, J = 8.5 Hz, 1H), 2.58 – 2.48 (m, 5H), 2.51 – 2.41 (m, 1H), 2.44 – 2.37 (m, 1H), 2.16 (dq, J = 11.0, 8.8 Hz, 1H), 2.03 (dddd, J = 14.9, 11.4, 7.9, 4.5 Hz, 1H), 1.86 (s, 1H), 1.77 (s, 2H), 1.00 (dd, J = 11.1, 4.9 Hz, 6H).
^{13}C NMR (125 MHz, CD_3OD) δ 168.3, 159.8, 143.2, 139.9, 138.4, 138.0, 136.0, 128.1, 126.7, 126.6, 125.0, 123.0, 41.1, 30.1, 28.7, 28.7, 26.5, 23.5, 21.7, 19.5, 15.3.
LCMS (ESI+): calculated for $C_{23}H_{28}N_5O_3S$ $(M+H)^+$: 454.2; found 454.4.

Compound 27a

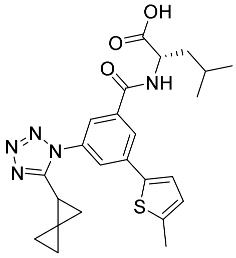

^{1}H NMR (500 MHz, CD_3OD) δ 8.29 (q, J = 1.5 Hz, 1H), 7.91 (t, J = 1.8 Hz, 1H), 7.84 (q, J = 2.0 Hz, 1H), 7.42 (d, J = 3.6 Hz, 1H), 6.85 (dd, J = 3.6, 1.2 Hz, 1H), 4.74 – 4.66 (m, 1H), 2.56 – 2.50 (m, 4H), 1.88 – 1.80 (m, 1H), 1.83 – 1.72 (m, 4H), 1.14 – 1.06 (m, 1H), 1.01 (dd, J = 9.8, 5.5 Hz, 8H), 0.94 – 0.85 (m, 1H).
^{13}C NMR (125 MHz, CD_3OD) δ 175.9, 168.3, 168.2, 158.1, 143.2, 139.9, 138.5, 138.0, 137.9, 137.9, 137.9, 136.0, 128.1, 126.7, 126.5, 126.5, 124.9, 122.8, 122.8, 41.2, 41.2, 41.2, 26.3, 23.4, 21.7, 21.7, 20.9, 16.4, 16.4, 15.3, 12.9, 7.4, 5.6, 5.5.
LCMS (ESI+): calculated for $C_{24}H_{28}N_5O_3S$ $(M+H)^+$: 466.2; found 466.4.

Compound 28a

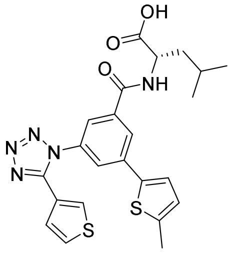

^{1}H NMR (500 MHz, CD_3OD) δ 8.33 (t, J = 1.7 Hz, 1H), 7.89 (dt, J = 5.4, 1.9 Hz, 2H), 7.81 (dd, J = 3.0, 1.3 Hz, 1H), 7.60 (dd, J = 5.2, 2.9 Hz, 1H), 7.37 – 7.28 (m, 2H), 6.81 (dd, J = 3.6, 1.3 Hz, 1H), 4.70 – 4.64 (m, 1H), 2.51 (d, J = 1.1 Hz, 3H), 1.86 – 1.69 (m, 3H), 0.99 (dd, J = 10.6, 5.6 Hz, 6H).
^{13}C NMR (125 MHz, CD_3OD) δ 168.0, 151.9, 143.3, 139.8, 138.5, 138.0, 136.6, 130.6, 129.0, 128.1, 128.0, 127.2, 126.7, 126.0, 124.7, 123.9, 41.1, 26.3, 23.4, 21.7, 15.3.
LCMS (ESI+): calculated for $C_{23}H_{24}N_5O_3S_2$ $(M+H)^+$: 482.1; found 482.3.

Compound 29a

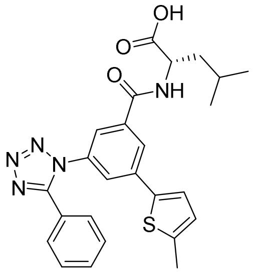

^{1}H NMR (500 MHz, CD_3OD) δ 8.26 (d, *J* = 1.7 Hz, 1H), 7.85 (t, *J* = 1.8 Hz, 1H), 7.78 (t, *J* = 1.9 Hz, 1H), 7.64 – 7.59 (m, 2H), 7.60 – 7.53 (m, 1H), 7.49 (dd, *J* = 8.4, 7.0 Hz, 2H), 7.26 (d, *J* = 3.7 Hz, 1H), 6.78 (dd, *J* = 3.6, 1.4 Hz, 1H), 4.68 – 4.62 (m, 1H), 2.49 (d, *J* = 1.1 Hz, 3H), 1.79 (dt, *J* = 14.8, 7.4 Hz, 1H), 1.77 – 1.67 (m, 2H), 0.97 (dd, *J* = 14.6, 5.6 Hz, 6H).
^{13}C NMR (125 MHz, CD_3OD) δ 168.0, 155.5, 143.2, 139.8, 138.3, 137.9, 136.6, 132.8, 130.3, 128.0, 126.7, 126.5, 125.6, 124.7, 123.5, 41.1, 26.3, 23.4, 21.7, 15.3.
LCMS (ESI+): calculated for $C_{25}H_{26}N_5O_3S$ $(M+H)^+$: 476.2; found 476.4.

Compound 30a

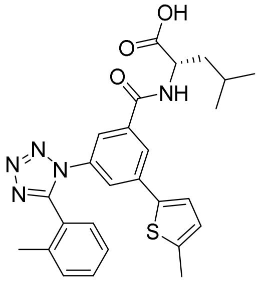

^{1}H NMR (500 MHz, CD_3OD) δ 8.16 (t, *J* = 1.6 Hz, 1H), 7.82 (t, *J* = 1.8 Hz, 1H), 7.60 (t, *J* = 1.8 Hz, 1H), 7.50 (td, *J* = 7.5, 1.5 Hz, 1H), 7.43 – 7.37 (m, 2H), 7.37 – 7.31 (m, 1H), 7.16 (d, *J* = 3.6 Hz, 1H), 6.77 (dd, *J* = 3.6, 1.3 Hz, 1H), 4.63 (dd, *J* = 10.5, 4.0 Hz, 1H), 2.49 (d, *J* = 1.1 Hz, 3H), 2.15 (s, 3H), 1.85 – 1.67 (m, 3H), 1.03 – 0.94 (m, 6H).
^{13}C NMR (125 MHz, CD_3OD) δ 176.0, 168.1, 155.3, 143.1, 139.8, 139.1, 138.1, 137.9, 136.2, 132.8, 132.2, 131.5, 128.0, 127.6, 126.4, 126.2, 124.7, 123.9, 122.1, 52.9, 41.2, 26.3, 23.5, 21.7, 19.7, 15.3.
LCMS (ESI+): calculated for $C_{26}H_{28}N_5O_3S$ $(M+H)^+$: 490.2; found 490.4.

Compound 31a

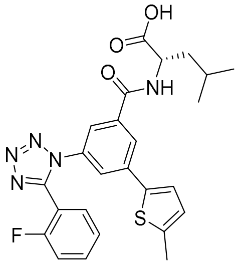

^{1}H NMR (500 MHz, CD_3OD) δ 8.22 (d, *J* = 1.7 Hz, 1H), 7.83 (t, *J* = 1.7 Hz, 1H), 7.81 – 7.59 (m, 3H), 7.43 (td, *J* = 7.6, 0.9 Hz, 1H), 7.29 – 7.21 (m, 2H), 6.78 (dd, *J* = 3.6, 1.3 Hz, 1H), 4.64 (dd, *J* = 10.7, 3.8 Hz, 1H), 2.49 (d, *J* = 1.1 Hz, 3H), 1.87 – 1.74 (m, 1H), 1.74 (q, *J* = 4.3 Hz, 1H), 1.74 – 1.67 (m, 1H), 0.98 (dd, *J* = 17.4, 5.8 Hz, 6H).

^{13}C NMR (125 MHz, CD_3OD) δ 175.9, 168.0, 161.9, 159.9, 151.7, 143.1, 139.8, 138.2, 137.8, 136.5, 135.6, 135.5, 133.0, 132.9, 130.3, 128.0, 126.5, 126.5, 126.4, 124.0, 122.2, 117.7, 117.5, 113.4, 113.3, 41.1, 26.3, 23.5, 21.7, 15.3.
LCMS (ESI+): calculated for $C_{25}H_{25}FN_5O_3S$ $(M+H)^+$: 494.2; found 494.4.

Compound 32a

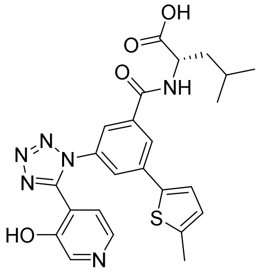

^1H NMR (500 MHz, CD_3OD) δ 8.23 (d, *J* = 1.5 Hz, 1H), 7.87 (d, *J* = 1.7 Hz, 1H), 7.74 (t, *J* = 1.8 Hz, 1H), 7.25 (d, *J* = 3.6 Hz, 1H), 6.79 (dd, *J* = 3.6, 1.2 Hz, 1H), 4.64 (dd, *J* = 10.6, 4.0 Hz, 1H), 2.49 (d, *J* = 1.1 Hz, 3H), 1.87 – 1.76 (m, 1H), 1.78 – 1.65 (m, 2H), 0.98 (dd, *J* = 18.0, 6.1 Hz, 6H).
^{13}C NMR (125 MHz, CD_3OD) δ 175.9, 168.0, 143.1, 140.9, 139.9, 138.1, 137.6, 137.0, 128.0, 126.3, 123.8, 122.0, 52.8, 41.1, 26.3, 23.4, 21.6, 15.3.
LCMS (ESI+): calculated for $C_{24}H_{25}N_6O_3S$ $(M+H)^+$: 493.2; found 493.4.

Compound 33a

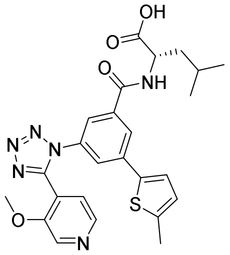

^1H NMR (500 MHz, CD_3OD) δ 8.49 (s, 2H), 8.24 (t, *J* = 1.6 Hz, 1H), 7.81 (t, *J* = 1.8 Hz, 1H), 7.78 (s, 1H), 7.70 (t, *J* = 1.9 Hz, 1H), 7.26 (d, *J* = 3.6 Hz, 1H), 6.80 (dd, *J* = 3.6, 1.3 Hz, 1H), 4.65 (dd, *J* = 10.5, 3.9 Hz, 1H), 3.57 (s, 3H), 2.50 (d, *J* = 1.1 Hz, 3H), 1.86 – 1.76 (m, 1H), 1.79 – 1.67 (m, 2H), 0.98 (dd, *J* = 16.9, 6.0 Hz, 6H).
^{13}C NMR (125 MHz, CD_3OD) δ 175.9, 168.0, 151.8, 143.5, 143.2, 139.8, 138.1, 137.8, 137.0, 135.7, 128.1, 126.4, 126.3, 123.6, 122.7, 121.9, 56.9, 52.8, 41.1, 26.3, 23.4, 21.7, 15.3.
LCMS (ESI+): calculated for $C_{25}H_{27}N_6O_4S$ $(M+H)^+$: 507.2; found 507.4.

Compound 34a

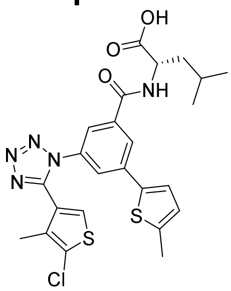

^1H NMR (500 MHz, CD_3OD) δ 8.25 (t, *J* = 1.6 Hz, 1H), 7.84 (t, *J* = 1.7 Hz, 1H), 7.78 (t, *J* = 1.8 Hz, 1H), 7.50 (s, 1H), 7.30 (d, *J* = 3.6 Hz, 1H), 6.81 (dd, *J* = 3.6, 1.3 Hz,

1H), 4.66 (dd, *J* = 11.0, 3.4 Hz, 1H), 2.51 (d, *J* = 1.1 Hz, 3H), 2.15 (s, 3H), 1.86 – 1.79 (m, 1H), 1.77 – 1.70 (m, 2H), 0.99 (dd, *J* = 15.0, 5.7 Hz, 6H).
^{13}C NMR (125 MHz, CD_3OD) δ 168.1, 151.4, 143.3, 139.8, 138.4, 137.9, 136.3, 135.2, 128.7, 128.6, 128.1, 126.6, 126.6, 124.8, 124.6, 122.9, 41.1, 26.3, 23.5, 21.7, 15.3, 13.0.
LCMS (ESI+): calculated for $C_{24}H_{25}ClN_5O_3S_2$ $(M+H)^+$: 530.1; found 530.3.

Compound 35a

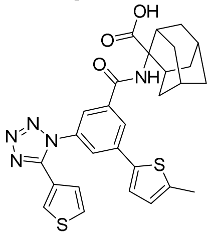

^{1}H NMR (500 MHz, DMSO-6) δ 12.27 (s, 1H), 8.41 (s, 1H), 8.15 (d, *J* = 1.7 Hz, 1H), 8.05 (d, *J* = 1.9 Hz, 1H), 7.91 (dt, *J* = 3.0, 1.6 Hz, 2H), 7.73 (dd, *J* = 5.2, 2.9 Hz, 1H), 7.47 (d, *J* = 3.6 Hz, 1H), 7.25 (dd, *J* = 5.2, 1.3 Hz, 1H), 6.88 (dd, *J* = 3.5, 1.3 Hz, 1H), 2.61 (t, *J* = 3.2 Hz, 2H), 2.49 (s, 3H), 2.13 – 2.01 (m, 4H), 1.84 – 1.76 (m, 2H), 1.69 (d, *J* = 9.0 Hz, 4H), 1.55 (d, *J* = 12.7 Hz, 2H).
^{13}C NMR (125 MHz, DMSO-d6) δ 173.6, 164.4, 150.3, 141.3, 138.2, 137.0, 135.7, 134.8, 129.9, 128.6, 127.3, 126.9, 126.0, 125.9, 124.7, 123.5, 123.3, 63.5, 37.5, 33.5, 32.8, 31.4, 26.5, 26.3, 15.2.
LCMS (ESI+): calculated for $C_{28}H_{28}N_5O_3S_2$ $(M+H)^+$: 546.2; found 546.4.

Optimization of compound 25 (Scaffold b)

Compound 1b

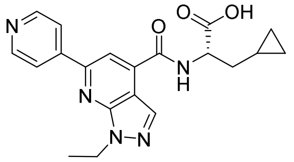

^{1}H NMR (500 MHz, DMSO-d_6) δ 12.78 (s, 1H), 9.25 (d, *J* = 7.8 Hz, 1H), 8.82 (s, 2H), 8.43 (d, *J* = 2.2 Hz, 1H), 8.38 (s, 1H), 8.27 (t, *J* = 5.8 Hz, 2H), 4.62 (dq, *J* = 16.7, 7.5 Hz, 3H), 1.86 (dt, *J* = 15.1, 8.0 Hz, 1H), 1.71 (dt, *J* = 13.2, 6.1 Hz, 1H), 1.50 (td, *J* = 7.2, 2.1 Hz, 3H), 0.91 (dt, *J* = 13.6, 7.2 Hz, 1H), 0.51 – 0.39 (m, *J* = 7.7 Hz, 2H), 0.28 – 0.21 (m, 1H), 0.12 (dt, *J* = 10.0, 4.4 Hz, 1H).
^{13}C NMR (125 MHz, DMSO-d_6) δ 173.3, 164.5, 153.0, 150.6, 150.4, 145.1, 136.6, 132.4, 121.4, 112.8, 112.3, 53.5, 41.8, 35.7, 14.8, 8.3, 4.8, 4.0.
LCMS (ESI+): calculated for $C_{20}H_{22}N_5O_3$ $(M+H)^+$: 380.2; found 380.3.

Compound 2b

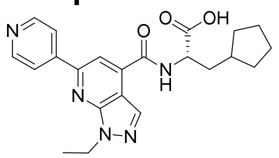

^{1}H NMR (500 MHz, DMSO-d_6) δ 12.83 (s, 1H), 9.23 (d, *J* = 7.9 Hz, 1H), 8.86 (d, *J* = 5.3 Hz, 2H), 8.44 (s, 1H), 8.40 (s, 1H), 8.34 (d, *J* = 5.2 Hz, 2H), 4.63 (q, *J* = 7.2 Hz,

2H), 4.55 (ddd, J = 9.6, 7.7, 5.1 Hz, 1H), 2.02 – 1.82 (m, 2H), 1.79 (ttd, J = 10.9, 8.8, 4.1 Hz, 2H), 1.67 – 1.56 (m, 2H), 1.50 (t, J = 7.2 Hz, 5H), 1.24 – 1.11 (m, 2H).
^{13}C NMR (125 MHz, DMSO-d$_6$) δ 174.1, 164.9, 153.1, 150.8, 150.2, 146.4, 136.9, 132.9, 122.2, 113.3, 112.8, 52.7, 42.2, 37.4, 37.1, 32.8, 32.1, 25.2, 25.0, 15.3.
LCMS (ESI+): calculated for $C_{22}H_{26}N_5O_3$ (M+H)$^+$: 408.2; found 408.3.

Compound 3b

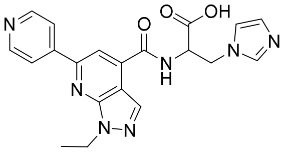

^{1}H NMR (500 MHz, CD_3OD) δ 9.03 (s, 1H), 8.80 – 8.75 (m, 2H), 8.39 (d, J = 5.5 Hz, 2H), 8.33 (s, 1H), 8.24 (s, 1H), 7.70 (s, 1H), 7.54 (s, 1H), 5.23 (t, J = 6.6 Hz, 1H), 4.99 (dd, J = 14.1, 4.7 Hz, 1H), 4.78 – 4.70 (m, 1H), 4.70 (s, 1H), 4.68 (d, J = 7.3 Hz, 1H), 1.56 (t, J = 7.2 Hz, 3H).
^{13}C NMR (125 MHz, CD_3OD) δ 171.4, 167.4, 154.2, 152.0, 149.8, 149.3, 137.6, 137.5, 133.2, 124.2, 123.6, 121.3, 114.2, 113.8, 54.6, 51.0, 43.4, 15.2.
LCMS (ESI+): calculated for $C_{20}H_{20}N_7O_3$ (M+H)$^+$: 406.2; found 406.2.

Compound 4b

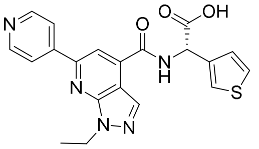

^{1}H NMR (500 MHz, CD_3OD) δ 8.82 (d, J = 5.6 Hz, 2H), 8.54 (d, J = 5.9 Hz, 2H), 8.44 (s, 1H), 8.35 (s, 1H), 7.59 – 7.56 (m, 1H), 7.48 (dd, J = 5.1, 3.0 Hz, 1H), 7.29 (dd, J = 5.1, 1.4 Hz, 1H), 5.94 (s, 1H), 4.71 (d, J = 7.3 Hz, 2H), 1.58 (t, J = 7.2 Hz, 3H).
^{13}C NMR (125 MHz, CD_3OD) δ 173.3, 167.1, 153.3, 152.0, 151.3, 148.4, 148.2, 138.5, 137.4, 133.5, 128.2, 127.5, 125.1, 124.3, 114.8, 114.3, 54.4, 43.4, 15.2.
LCMS (ESI+): calculated for $C_{20}H_{18}N_5O_3S$ (M+H)$^+$: 408.1; found 408.2.

Compound 5b

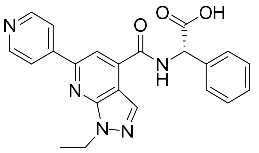

^{1}H NMR (500 MHz, DMSO-d$_6$) δ 13.13 (s, 1H), 9.70 (d, J = 7.3 Hz, 1H), 8.80 (s, 2H), 8.43 (d, J = 1.9 Hz, 2H), 8.26 (d, J = 5.1 Hz, 2H), 7.56 (d, J = 7.3 Hz, 2H), 7.47 – 7.35 (m, 3H), 5.75 – 5.70 (m, 1H), 4.63 (q, J = 7.2 Hz, 2H), 1.50 (t, J = 7.2 Hz, 3H).
^{13}C NMR (125 MHz, DMSO-d$_6$) δ 171.6, 164.4, 164.3, 153.0, 150.4, 150.4, 145.2, 136.6, 136.2, 132.3, 128.7, 128.2, 121.5, 112.8, 112.7, 57.1, 57.0, 41.8, 14.8.
LCMS (ESI+): calculated for $C_{22}H_{20}N_5O_3$ (M+H)$^+$: 402.2; found 402.3.

Compound 6b

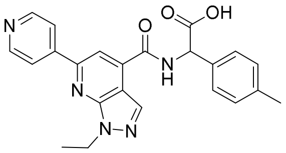

^{1}H NMR (500 MHz, DMSO-d6) δ 13.06 (s, 1H), 9.63 (d, *J* = 7.2 Hz, 1H), 8.79 (d, *J* = 5.0 Hz, 2H), 8.41 (d, *J* = 4.3 Hz, 2H), 8.30 – 8.21 (m, 2H), 7.43 (d, *J* = 7.8 Hz, 2H), 7.24 (d, *J* = 7.7 Hz, 2H), 5.66 (d, *J* = 7.1 Hz, 1H), 4.62 (q, *J* = 7.2 Hz, 2H), 2.32 (s, 3H), 1.50 (t, *J* = 7.2 Hz, 3H).
^{13}C NMR (125 MHz, DMSO-d6) δ 171.8, 164.3, 153.0, 150.5, 150.4, 145.1, 137.5, 136.2, 133.6, 132.3, 129.2, 129.0, 128.1, 127.5, 121.5, 112.8, 112.7, 56.8, 41.8, 20.8, 14.8.
LCMS (ESI+): calculated for $C_{23}H_{22}N_5O_3$ $(M+H)^+$: 416.2; found 416.2.

Compound 7b

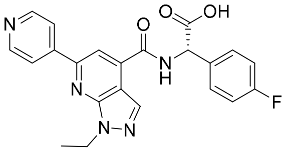

^{1}H NMR (500 MHz, DMSO-d6) δ 13.19 (s, 1H), 9.70 (d, *J* = 7.2 Hz, 1H), 8.80 (s, 2H), 8.42 (d, *J* = 4.5 Hz, 2H), 8.25 (d, *J* = 4.9 Hz, 2H), 7.61 (dd, *J* = 8.4, 5.4 Hz, 2H), 7.27 (t, *J* = 8.7 Hz, 2H), 5.74 (d, *J* = 7.0 Hz, 1H), 4.63 (q, *J* = 7.3 Hz, 2H), 1.50 (t, *J* = 7.2 Hz, 3H).
^{13}C NMR (125 MHz, DMSO-d6) δ 171.5, 164.3, 164.2, 162.9, 161.0, 153.0, 150.5, 150.4, 145.1, 136.1, 132.9, 132.9, 132.3, 130.4, 130.3, 121.5, 115.5, 115.4, 112.8, 112.7, 56.3, 56.2, 41.8, 14.8.
LCMS (ESI+): calculated for $C_{22}H_{19}FN_5O_3$ $(M+H)^+$: 420.2; found 420.2.

Compound 8b

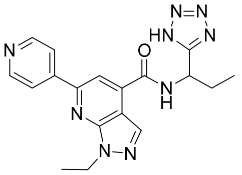

^{1}H NMR (500 MHz, DMSO-d6) δ 9.59 (d, *J* = 7.8 Hz, 1H), 8.82 (d, *J* = 4.6 Hz, 2H), 8.42 (d, *J* = 14.7 Hz, 2H), 8.27 – 8.22 (m, 2H), 5.44 (td, *J* = 8.5, 6.0 Hz, 1H), 4.63 (q, *J* = 7.2 Hz, 2H), 2.19 (ddd, *J* = 13.7, 7.7, 6.2 Hz, 1H), 2.07 (ddd, *J* = 13.6, 8.7, 7.1 Hz, 1H), 1.49 (t, *J* = 7.2 Hz, 3H), 1.02 (t, *J* = 7.3 Hz, 3H).
^{13}C NMR (150 MHz, DMSO-d6) δ 164.6, 152.9, 150.5, 150.4, 145.1, 136.2, 132.3, 121.4, 112.7, 112.5, 46.2, 41.8, 26.1, 14.8, 10.5.
LCMS (ESI+): calculated for $C_{18}H_{20}N_9O$ $(M+H)^+$: 378.2; found 378.3.

Compound 9b

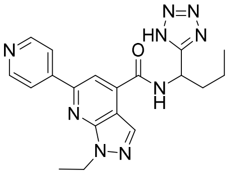

^{1}H NMR (500 MHz, DMSO-d6) δ 9.60 (d, *J* = 7.8 Hz, 1H), 8.83 (s, 2H), 8.42 (d, *J* = 16.2 Hz, 2H), 8.25 (d, *J* = 5.0 Hz, 2H), 5.53 (td, *J* = 8.5, 6.1 Hz, 1H), 4.63 (q, *J* = 7.2

Hz, 2H), 2.18 – 2.01 (m, 2H), 1.50 (t, J = 7.3 Hz, 3H), 1.54 – 1.37 (m, 2H), 0.96 (t, J = 7.3 Hz, 3H).
^{13}C NMR (150 MHz, DMSO-d$_6$) δ 164.5, 152.9, 150.5, 150.4, 145.0, 136.2, 132.3, 121.3, 112.7, 112.5, 44.4, 41.7, 34.8, 18.7, 14.8, 13.4.
LCMS (ESI+): calculated for $C_{19}H_{22}N_9O$ $(M+H)^+$: 392.2; found 392.2.

Compound 10b

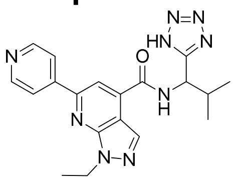

^{1}H NMR (500 MHz, DMSO-d$_6$) δ 9.58 (d, J = 8.1 Hz, 1H), 8.84 – 8.80 (m, 2H), 8.39 (d, J = 5.1 Hz, 2H), 8.26 (d, J = 4.8 Hz, 2H), 5.33 (t, J = 8.0 Hz, 1H), 4.63 (q, J = 7.2 Hz, 2H), 1.49 (t, J = 7.2 Hz, 3H), 1.08 (d, J = 6.7 Hz, 3H), 0.91 (d, J = 6.6 Hz, 3H).
^{13}C NMR (150 MHz, DMSO-d$_6$) δ 164.7, 152.9, 150.5, 150.3, 145.1, 136.2, 132.2, 121.4, 112.7, 112.6, 50.4, 41.7, 31.1, 19.2, 18.9, 14.8.
LCMS (ESI+): calculated for $C_{19}H_{22}N_9O$ $(M+H)^+$: 392.2; found 392.3.

Compound 11b

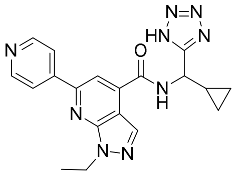

^{1}H NMR (500 MHz, DMSO-d$_6$) δ 9.86 (d, J = 7.4 Hz, 1H), 8.82 (d, J = 5.2 Hz, 2H), 8.42 (d, J = 4.0 Hz, 2H), 8.26 (d, J = 5.0 Hz, 2H), 4.89 (t, J = 8.4 Hz, 1H), 4.63 (q, J = 7.3 Hz, 2H), 1.58 (tt, J = 9.1, 4.9 Hz, 1H), 1.49 (t, J = 7.2 Hz, 3H), 0.69 (q, J = 7.7 Hz, 2H), 0.57 (q, J = 4.0 Hz, 2H).
^{13}C NMR (150 MHz, DMSO-d$_6$) δ 164.2, 157.4, 152.9, 150.5, 150.4, 145.0, 136.1, 132.3, 121.3, 112.7, 112.5, 49.2, 41.7, 14.7, 14.6, 3.9, 3.8.
LCMS (ESI+): calculated for $C_{19}H_{20}N_9O$ $(M+H)^+$: 390.2; found 390.3.

Compound 12b

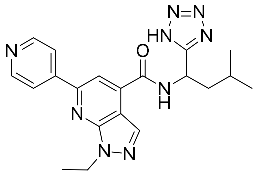

^{1}H NMR (500 MHz, DMSO-d$_6$) δ 9.62 (d, J = 7.9 Hz, 1H), 8.83 (s, 2H), 8.43 (s, 1H), 8.39 (s, 1H), 8.25 (s, 2H), 5.60 (q, J = 7.8 Hz, 1H), 4.63 (q, J = 7.2 Hz, 2H), 2.07 – 1.92 (m, 2H), 1.70 (dt, J = 13.7, 6.8 Hz, 1H), 1.49 (t, J = 7.2 Hz, 3H), 0.97 (d, J = 6.5 Hz, 6H).
^{13}C NMR (125 MHz, DMSO-d$_6$) δ 164.5, 158.1, 153.0, 150.6, 150.5, 145.1, 136.2, 132.4, 121.5, 112.8, 112.5, 43.0, 41.8, 24.4, 22.7, 21.7, 14.9.
LCMS (ESI+): calculated for $C_{20}H_{24}N_9O$ $(M+H)^+$: 406.2; found 406.3.

Compound 13b

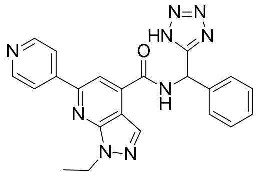

^1H NMR (500 MHz, DMSO-d_6) δ 10.18 (d, *J* = 7.6 Hz, 1H), 8.81 (s, 2H), 8.47 (s, 1H), 8.43 (s, 1H), 8.25 (d, *J* = 5.6 Hz, 2H), 7.51 (d, *J* = 7.5 Hz, 2H), 7.44 (t, *J* = 7.5 Hz, 2H), 7.38 (t, *J* = 7.2 Hz, 1H), 7.32 (dd, *J* = 13.4, 5.6 Hz, 1H), 6.82 (d, *J* = 7.5 Hz, 1H), 4.63 (q, *J* = 7.2 Hz, 2H), 1.49 (t, *J* = 7.2 Hz, 3H).
^{13}C NMR (125 MHz, DMSO-d_6) δ 164.3, 153.0, 150.6, 150.4, 145.1, 137.5, 135.8, 132.3, 128.7, 128.3, 128.0, 121.4, 112.8, 112.8, 48.7, 41.8, 14.8.
LCMS (ESI+): calculated for $C_{22}H_{20}N_9O$ $(M+H)^+$: 426.2; found 426.2.

Compounds 14b-15b: commercial vendor

Compound 16b

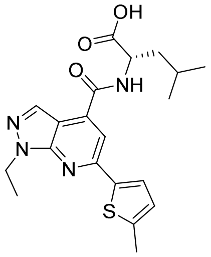

^1H NMR (500 MHz, CD_3OD) δ 8.26 (s, 1H), 7.97 (s, 1H), 7.71 (d, *J* = 3.6 Hz, 1H), 6.86 (dd, *J* = 3.7, 1.3 Hz, 1H), 4.80 – 4.73 (m, 1H), 4.57 (q, *J* = 7.2 Hz, 2H), 2.55 (d, *J* = 1.1 Hz, 3H), 1.90 – 1.75 (m, 3H), 1.53 (t, *J* = 7.2 Hz, 3H), 1.03 (d, *J* = 5.3 Hz, 6H).
^{13}C NMR (125 MHz, CD_3OD) δ 168.1, 154.0, 151.7, 145.6, 143.2, 137.8, 133.4, 128.4, 127.8, 112.3, 112.0, 43.0, 41.3, 26.4, 23.4, 21.8, 15.6, 15.2.
LCMS (ESI+): calculated for $C_{20}H_{24}N_4O_3S$ $(M+H)^+$: 401.2; found 401.1.

Compound 17b

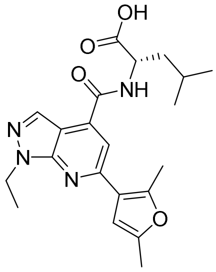

^1H NMR (500 MHz, CD_3OD) δ 8.24 (s, 1H), 7.69 (s, 1H), 6.57 (d, *J* = 1.3 Hz, 1H), 4.76 (dd, *J* = 9.9, 4.5 Hz, 1H), 4.57 (q, *J* = 7.2 Hz, 2H), 2.72 (s, 3H), 2.31 (s, 3H), 1.90 – 1.75 (m, 3H), 1.51 (t, *J* = 7.2 Hz, 3H), 1.03 (d, *J* = 6.1 Hz, 6H).
^{13}C NMR (125 MHz, CD_3OD) δ 175.9, 168.3, 155.0, 152.6, 151.8, 151.4, 137.6, 133.1, 121.7, 114.2, 111.5, 107.0, 52.7, 43.1, 41.3, 26.4, 23.4, 21.8, 15.2, 14.6, 13.3.
LCMS (ESI+): calculated for $C_{21}H_{26}N_4O_4$ $(M+H)^+$: 399.2; found 399.0.

Compound 18b: commercial vendor

Compound 19b

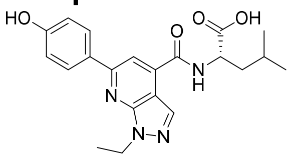

^{1}H NMR (600 MHz, CD_3OD) δ 8.28 (d, *J* = 1.9 Hz, 1H), 8.15 – 8.10 (m, 2H), 8.02 (d, *J* = 2.0 Hz, 1H), 6.96 – 6.91 (m, 2H), 4.78 (t, *J* = 6.7 Hz, 1H), 4.63 (qd, *J* = 7.3, 1.8 Hz, 2H), 1.90 – 1.84 (m, 1H), 1.81 (tq, *J* = 12.0, 6.2 Hz, 2H), 1.54 (td, *J* = 7.2, 1.9 Hz, 3H), 1.03 (d, *J* = 5.1 Hz, 6H).
^{13}C NMR (151 MHz, CD_3OD) δ 175.9, 168.4, 160.7, 158.6, 152.1, 137.9, 133.1, 131.3, 130.2, 116.7, 113.0, 112.2, 52.8, 43.0, 41.4, 26.4, 23.4, 21.8, 15.3.
LCMS (ESI+): calculated for $C_{21}H_{25}N_4O_4$ $(M+H)^+$: 397.2; found 397.5.

Compound 20b

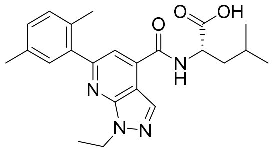

^{1}H NMR (500 MHz, CD_3OD) δ 8.37 (s, 1H), 7.68 (s, 1H), 7.36 (d, *J* = 1.9 Hz, 1H), 7.26 – 7.16 (m, 2H), 4.80 – 4.73 (m, 1H), 4.61 (q, *J* = 7.3 Hz, 2H), 2.38 (d, *J* = 12.1 Hz, 6H), 1.88 – 1.75 (m, 3H), 1.51 (t, *J* = 7.2 Hz, 3H), 1.06 – 0.97 (m, 6H).
^{13}C NMR (125 MHz, CD_3OD) δ 168.1, 161.6, 151.4, 140.8, 137.7, 136.7, 134.4, 133.2, 132.0, 131.5, 130.6, 117.1, 112.5, 43.3, 41.3, 26.4, 23.4, 21.8, 21.0, 20.2, 15.4.
LCMS (ESI+): calculated for $C_{23}H_{29}N_4O_3$ $(M+H)^+$: 409.2; found 409.3.

Compound 21b

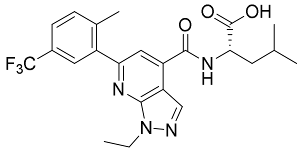

^{1}H NMR (500 MHz, CD_3OD) δ 8.40 (s, 1H), 7.84 (d, *J* = 2.1 Hz, 1H), 7.73 (s, 1H), 7.68 (dd, *J* = 8.0, 2.0 Hz, 1H), 7.57 (d, *J* = 8.0 Hz, 1H), 4.77 (dd, *J* = 9.2, 4.4 Hz, 1H), 4.62 (q, *J* = 7.2 Hz, 2H), 2.50 (s, 3H), 1.87 – 1.78 (m, 2H), 1.79 (t, *J* = 6.1 Hz, 1H), 1.52 (t, *J* = 7.2 Hz, 3H), 1.01 (q, *J* = 3.1 Hz, 6H).
^{13}C NMR (120 MHz, CD_3OD) δ 167.8, 159.4, 151.5, 142.4, 141.8, 138.1, 133.3, 132.8, 129.9, 129.6, 129.4, 129.1, 127.7, 127.7, 127.6, 127.6, 126.8, 126.5, 126.4, 126.4, 126.4, 124.7, 122.5, 116.8, 113.0, 43.3, 41.3, 26.4, 23.4, 21.8, 20.8, 15.3.
LCMS (ESI+): calculated for $C_{23}H_{29}F_3N_4O_3$ $(M+H)^+$: 463.2; found 463.2.

Compound 22b

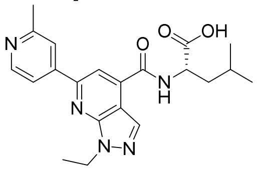

^{1}H NMR (500 MHz, CD_3OD) δ 8.63 (s, 1H), 8.41 (s, 1H), 8.27 (d, *J* = 6.3 Hz, 2H), 8.20 (d, *J* = 5.3 Hz, 1H), 4.79 (d, *J* = 9.7 Hz, 1H), 4.70 (q, *J* = 7.2 Hz, 2H), 2.72 (d, *J* = 2.6 Hz, 3H), 1.91 – 1.79 (m, 3H), 1.57 (t, *J* = 7.2 Hz, 3H), 1.04 (d, *J* = 5.5 Hz, 6H).

^{13}C NMR (125 MHz, CD_3OD) δ 167.7, 159.6, 154.5, 152.0, 149.0, 138.6, 133.4, 123.4, 123.4, 120.9, 120.9, 114.4, 113.8, 43.3, 41.4, 26.3, 23.4, 22.9, 21.8, 15.2.
LCMS (ESI+): calculated for $C_{21}H_{26}N_5O_3$ $(M+H)^+$: 396.2; found 396.3.

Compound 23b

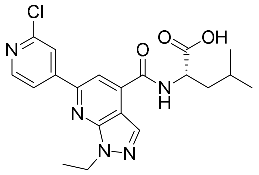

^1H NMR (600 MHz, CD_3OD) δ 9.44 (s, 1H), 8.72 (d, J = 8.0 Hz, 1H), 8.38 – 8.35 (m, 1H), 8.17 (d, J = 1.8 Hz, 1H), 7.66 (t, J = 6.3 Hz, 1H), 4.78 (dd, J = 10.1, 4.6 Hz, 1H), 4.67 (dd, J = 8.0, 6.3 Hz, 2H), 4.25 (q, J = 7.2 Hz, 2H), 1.89 – 1.75 (m, 3H), 1.56 (td, J = 7.4, 1.9 Hz, 3H), 1.31 (ddd, J = 8.1, 6.8, 1.9 Hz, 3H), 1.03 (t, J = 5.2 Hz, 6H).
^{13}C NMR (150 MHz, CD_3OD) δ 174.1, 167.8, 155.0, 152.1, 150.6, 149.0, 138.3, 137.2, 136.4, 133.4, 125.7, 113.6, 113.3, 62.6, 53.0, 43.2, 41.2, 26.3, 23.3, 21.9, 15.2, 14.5.
LCMS (ESI+): calculated for $C_{20}H_{23}ClN_5O_3$ $(M+H)^+$: 416.1; found 416.2.

Compound 24b

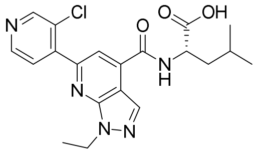

^1H NMR (500 MHz, CD_3OD) δ 8.76 (s, 1H), 8.64 (d, J = 4.9 Hz, 1H), 8.42 (s, 1H), 7.90 (s, 1H), 7.77 (d, J = 5.0 Hz, 1H), 4.80 – 4.73 (m, 1H), 4.64 (q, J = 7.2 Hz, 2H), 1.88 – 1.80 (m, 1H), 1.80 (s, 1H), 1.82 – 1.75 (m, 1H), 1.53 (t, J = 7.2 Hz, 3H), 1.01 (q, J = 2.8 Hz, 6H).
^{13}C NMR (126 MHz, CD_3OD) δ 175.8, 167.5, 154.9, 151.5, 151.1, 148.9, 147.7, 138.0, 133.4, 131.5, 127.1, 116.6, 113.7, 52.7, 43.4, 41.3, 26.4, 23.4, 21.8, 15.3.
LCMS (ESI+): calculated for $C_{20}H_{23}ClN_5O_3$ $(M+H)^+$: 416.1; found 416.2.

Compound 25b

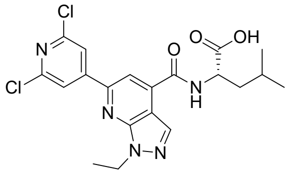

^1H NMR (500 MHz, CD_3OD) δ 8.43 (s, 1H), 8.29 (d, J = 8.5 Hz, 3H), 4.79 (dd, J = 10.6, 3.7 Hz, 1H), 4.69 (q, J = 7.3 Hz, 2H), 1.91 – 1.77 (m, 3H), 1.57 (t, J = 7.3 Hz, 3H), 1.07 – 0.99 (m, 6H).
^{13}C NMR (126 MHz, CD_3OD) δ 176.0, 167.3, 153.2, 152.4, 152.4, 151.8, 138.5, 133.6, 122.2, 114.9, 113.8, 52.8, 43.3, 41.4, 26.3, 23.4, 21.8, 15.2.
LCMS (ESI+): calculated for $C_{20}H_{22}Cl_2N_5O_3$ $(M+H)^+$: 450.2; found 450.2.

Compound 26b

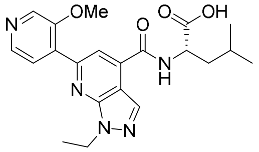

^{1}H NMR (500 MHz, CD_3OD) δ 8.54 (s, 1H), 8.38 (s, 1H), 8.37 (s, 1H), 8.19 (s, 1H), 8.01 (d, *J* = 4.9 Hz, 1H), 4.77 (dd, *J* = 10.5, 3.9 Hz, 1H), 4.66 (q, *J* = 7.3 Hz, 2H), 4.07 (s, 3H), 1.89 – 1.77 (m, 3H), 1.54 (t, *J* = 7.2 Hz, 3H), 1.03 (dt, *J* = 7.3, 3.7 Hz, 6H).
^{13}C NMR (126 MHz, CD_3OD) δ 175.7, 168.1, 155.2, 154.0, 151.7, 142.8, 137.6, 137.6, 135.2, 133.1, 126.3, 117.8, 113.4, 57.3, 52.7, 43.3, 41.3, 26.4, 23.4, 21.8, 15.3.
LCMS (ESI+): calculated for $C_{21}H_{29}N_5O_4$ $(M+H)^+$: 412.2; found 412.6.

Compound 27b

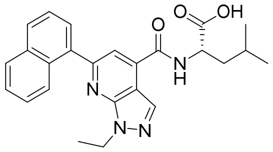

^{1}H NMR (600 MHz, CD_3OD) δ 8.43 (d, *J* = 1.8 Hz, 1H), 8.10 (d, *J* = 8.5 Hz, 1H), 8.03 (d, *J* = 8.3 Hz, 1H), 7.99 (d, *J* = 8.2 Hz, 1H), 7.85 (d, *J* = 1.8 Hz, 1H), 7.75 (d, *J* = 7.1 Hz, 1H), 7.67 – 7.61 (m, 1H), 7.55 (t, *J* = 7.5 Hz, 1H), 7.51 (t, *J* = 7.7 Hz, 1H), 4.77 (dd, *J* = 9.4, 4.1 Hz, 1H), 4.64 (tt, *J* = 8.4, 4.2 Hz, 2H), 1.86 – 1.75 (m, 3H), 1.54 (td, *J* = 7.2, 1.8 Hz, 3H), 1.03 – 0.96 (m, 6H).
^{13}C NMR (151 MHz, CD_3OD) δ 175.8, 168.1, 160.5, 151.7, 139.1, 138.0, 135.5, 133.3, 132.5, 130.6, 129.5, 129.2, 127.8, 127.2, 126.5, 126.3, 117.9, 113.0, 52.7, 43.4, 41.3, 26.4, 23.4, 21.8, 15.3.
LCMS (ESI+): calculated for $C_{25}H_{27}N_4O_3$ $(M+H)^+$: 431.2; found 431.2.

Compound 28b

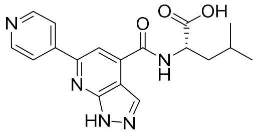

^{1}H NMR (600 MHz, CD_3OD) δ 8.82 (d, *J* = 5.1 Hz, 2H), 8.35 (d, *J* = 2.2 Hz, 1H), 8.21 (d, *J* = 4.8 Hz, 2H), 7.89 (d, *J* = 1.8 Hz, 1H), 7.05 (d, *J* = 2.3 Hz, 1H), 4.73 (s, 1H), 1.93 – 1.74 (m, 3H), 1.01 (t, *J* = 6.4 Hz, 6H).
^{13}C NMR (150 MHz, CD_3OD) δ 150.9, 147.2, 125.1, 106.6, 100.142.0, 26.3, 23.4, 22.0.
LCMS (ESI+): calculated for $C_{18}H_{20}N_5O_3$ $(M+H)^+$: 354.2; found 354.2.

Compound 29b

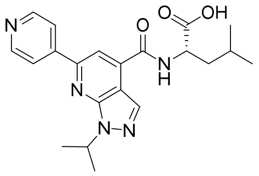

^{1}H NMR (600 MHz, CD_3OD) δ 8.91 (s, 2H), 8.53 (d, *J* = 2.1 Hz, 1H), 8.47 (s, 2H), 8.37 (d, *J* = 2.2 Hz, 1H), 5.64 – 5.55 (m, 1H), 4.95 (d, *J* = 9.3 Hz, 1H), 1.99 (td, *J* = 15.0, 7.9 Hz, 3H), 1.78 (d, *J* = 6.6 Hz, 6H), 1.18 (d, *J* = 4.9 Hz, 6H).

^{13}C NMR (150 MHz, CD_3OD) δ 175.8, 167.8, 154.4, 151.7, 150.7, 148.2, 138.5, 133.2, 123.4, 114.4, 113.5, 50.4, 41.4, 26.4, 23.4, 22.3, 21.8.
LCMS (ESI+): calculated for $C_{21}H_{26}N_5O_3$ $(M+H)^+$: 396.2; found 396.6.

Compound 30b

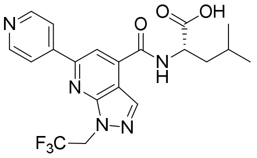

^1H NMR (600 MHz, CD_3OD) δ 8.76 (s, 2H), 8.52 (d, *J* = 1.9 Hz, 1H), 8.33 (t, *J* = 4.3 Hz, 3H), 5.42 – 5.34 (m, 2H), 4.82 – 4.77 (m, 1H), 1.85 (dt, *J* = 15.9, 8.4 Hz, 3H), 1.06 – 0.99 (m, 6H).
^{13}C NMR (150 MHz, CD_3OD) δ 175.8, 167.3, 155.6, 153.4, 150.9, 147.8, 139.1, 135.8, 126.0, 123.4, 114.5, 52.9, 41.4, 26.3, 23.4, 21.8.
LCMS (ESI+): calculated for $C_{20}H_{21}F_3N_5O_3$ $(M+H)^+$: 436.2; found 436.5.

Compound 31b

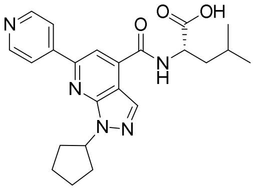

^1H NMR (500 MHz, CD_3OD) δ 8.85 (s, 2H), 8.43 (s, 2H), 8.39 (s, 1H), 8.25 (s, 1H), 5.62 (p, *J* = 7.4 Hz, 1H), 4.80 (d, *J* = 9.4 Hz, 1H), 2.31 – 2.14 (m, 4H), 2.10 – 1.99 (m, 2H), 1.91 – 1.84 (m, 1H), 1.84 (d, *J* = 5.0 Hz, 2H), 1.83 (d, *J* = 4.3 Hz, 1H), 1.81 (s, 1H), 1.03 (d, *J* = 5.1 Hz, 6H).
^{13}C NMR (125 MHz, CD_3OD) δ 167.8, 154.0, 152.2, 150.1, 149.0, 138.5, 133.2, 132.0, 131.5, 130.6, 117.1, 114.6, 113.6, 59.3, 41.4, 33.3, 33.3, 26.4, 25.8, 23.4, 21.8.
LCMS (ESI+): calculated for $C_{23}H_{28}N_5O_3$ $(M+H)^+$: 422.2; found 422.3.

Compound 32b

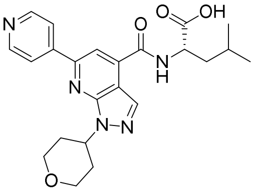

^1H NMR (600 MHz, CD_3OD) δ 8.80 (s, 2H), 8.44 – 8.38 (m, 3H), 8.27 (d, *J* = 1.6 Hz, 1H), 5.31 (td, *J* = 11.4, 5.6 Hz, 1H), 4.82 – 4.77 (m, 1H), 4.15 (dd, *J* = 11.8, 4.4 Hz, 2H), 3.73 (t, *J* = 11.9 Hz, 2H), 2.44 (qd, *J* = 12.3, 4.5 Hz, 2H), 2.06 – 2.00 (m, 2H), 1.85 (dt, *J* = 14.9, 8.2 Hz, 3H), 1.03 (d, *J* = 5.0 Hz, 6H).
^{13}C NMR (150 MHz, CD_3OD) δ 175.8, 167.6, 153.9, 151.9, 149.7, 149.5, 138.7, 133.6, 123.9, 114.7, 113.9, 68.2, 55.0, 52.8, 41.4, 33.4, 26.4, 23.4, 21.8.
LCMS (ESI+): calculated for $C_{23}H_{28}N_5O_3$ $(M+H)^+$: 438.2; found 438.2.

Compound 33b

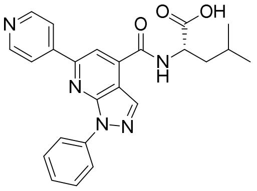

^{1}H NMR (500 MHz, CD_3OD) δ 8.76 (s, 2H), 8.63 (s, 1H), 8.38 – 8.31 (m, 5H), 7.60 (t, *J* = 8.0 Hz, 2H), 7.40 (t, *J* = 7.4 Hz, 1H), 4.85 – 4.78 (m, 1H), 1.94 – 1.80 (m, 3H), 1.10 – 1.01 (m, 6H).
^{13}C NMR (126 MHz, CD_3OD) δ 175.9, 167.4, 155.2, 152.2, 150.6, 148.5, 140.5, 138.9, 135.5, 130.3, 127.7, 123.5, 122.6, 116.1, 114.4, 52.8, 41.4, 26.4, 23.5, 21.8.
LCMS (ESI+): calculated for $C_{24}H_{24}N_5O_3$ $(M+H)^+$: 430.2; found 430.2.

Compound 34b

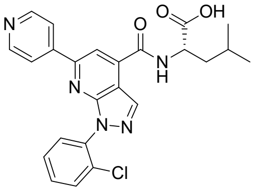

^{1}H NMR (600 MHz, CD_3OD) δ 8.88 (s, 2H), 8.73 (d, *J* = 1.7 Hz, 1H), 8.62 (s, 2H), 8.49 (d, *J* = 1.9 Hz, 1H), 7.73 (d, *J* = 7.9 Hz, 1H), 7.69 (d, *J* = 7.6 Hz, 1H), 7.65 – 7.56 (m, 2H), 1.92 – 1.83 (m, 3H), 1.06 (t, *J* = 5.2 Hz, 6H).
^{13}C NMR (150 MHz, CD_3OD) δ 175.8, 167.1, 153.3, 146.0, 139.6, 136.8, 136.1, 133.4, 132.2, 131.7, 131.2, 129.1, 125.2, 115.6, 115.1, 52.9, 41.4, 26.4, 23.4, 21.8.
LCMS (ESI+): calculated for $C_{24}H_{23}ClN_5O_3$ $(M+H)^+$: 464.1; found 464.1.

Compound 35b

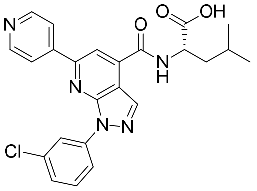

^{1}H NMR (500 MHz, CD_3OD) δ 8.64 (s, 1H), 8.46 (t, *J* = 2.1 Hz, 1H), 8.43 – 8.32 (m, 4H), 7.58 (d, *J* = 8.1 Hz, 1H), 7.38 (dd, *J* = 8.0, 2.1 Hz, 1H), 4.82 (dd, *J* = 10.6, 3.9 Hz, 1H), 1.93 – 1.81 (m, 3H), 1.05 (t, *J* = 5.8 Hz, 6H).
^{13}C NMR (126 MHz, CD_3OD) δ 175.9, 167.1, 155.1, 152.3, 150.0, 149.0, 141.7, 139.1, 136.2, 135.8, 131.7, 128.6, 127.3, 121.9, 120.1, 116.6, 114.8, 52.8, 41.4, 26.4, 23.5, 21.8.
LCMS (ESI+): calculated for $C_{24}H_{23}ClN_5O_3$ $(M+H)^+$: 464.1; found 464.2.

Compound 36b

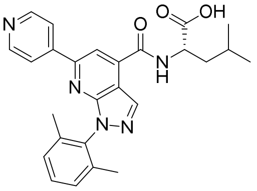

^{1}H NMR (600 MHz, CD_3OD) δ 8.80 – 8.58 (m, 3H), 8.34 (d, *J* = 1.8 Hz, 1H), 8.17 (s, 2H), 7.43 – 7.38 (m, 1H), 7.30 (d, *J* = 7.6 Hz, 2H), 4.83 (s, 1H), 1.96 (d, *J* = 4.3 Hz, 6H), 1.93 – 1.83 (m, 3H), 1.06 (t, *J* = 4.6 Hz, 6H).

^{13}C NMR (150 MHz, CD_3OD) δ 175.8, 167.6, 155.8, 153.1, 150.6, 148.1, 139.1, 138.5, 137.3, 135.4, 130.9, 129.5, 123.4, 114.2, 52.9, 41.4, 26.4, 23.4, 21.8, 17.8.
LCMS (ESI+): calculated for $C_{26}H_{28}N_5O_3$ $(M+H)^+$: 458.2; found 458.6.

Compound 37b

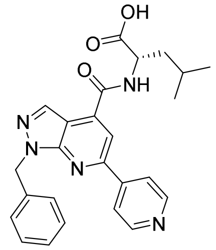

^1H NMR (500 MHz, CD_3OD) δ 8.80 (s, 2H), 8.48 (s, 1H), 8.40 (s, 1H), 7.39 – 7.34 (m, 2H), 7.34 – 7.27 (m, 2H), 7.29 – 7.22 (m, 1H), 5.86 (s, 2H), 4.85 – 4.77 (m, 1H), 1.90 – 1.79 (m, 3H), 1.03 (q, J = 2.6 Hz, 6H).
^{13}C NMR (125 MHz, CD_3OD) δ 175.8, 167.3, 152.6, 152.4, 146.1, 139.2, 138.2, 134.1, 129.7, 129.5, 129.0, 128.9, 115.2, 114.4, 52.8, 52.1, 41.4, 26.3, 23.4, 21.8.
LCMS (ESI+): calculated for $C_{25}H_{26}N_5O_3$ $(M+H)^+$: 444.2; found 444.1.

Compound 38b

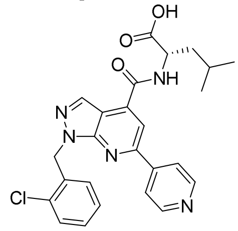

^1H NMR (500 MHz, CD_3OD) δ 8.99 (s, 2H), 8.89 (d, J = 6.1 Hz, 2H), 8.55 (s, 1H), 8.48 (s, 1H), 7.45 (dd, J = 8.1, 1.3 Hz, 1H), 7.30 (td, J = 7.7, 1.8 Hz, 1H), 7.22 (td, J = 7.6, 1.3 Hz, 1H), 7.08 (dd, J = 7.8, 1.7 Hz, 1H), 6.00 (d, J = 3.2 Hz, 2H), 4.88 – 4.73 (m, 1H), 1.91 – 1.80 (m, 3H), 1.04 (dt, J = 6.0, 3.0 Hz, 6H).
^{13}C NMR (125 MHz, CD_3OD) δ 175.8, 167.1, 155.8, 152.7, 151.6, 144.1, 139.4, 135.5, 134.6, 134.2, 130.9, 130.7, 130.6, 128.3, 125.8, 115.6, 114.9, 52.8, 41.4, 26.3, 23.4, 21.8.
LCMS (ESI+): calculated for $C_{25}H_{25}ClN_5O_3$ $(M+H)^+$: 478.2; found 478.4.

Compound 39b

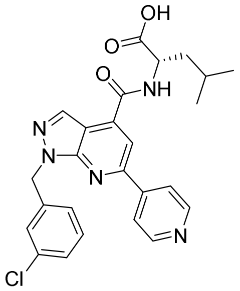

^1H NMR (500 MHz, CD_3OD) δ 8.94 (s, 2H), 8.73 (s, 2H), 8.50 (s, 1H), 8.40 (s, 1H), 7.39 (d, J = 1.8 Hz, 1H), 7.34 – 7.25 (m, 3H), 5.86 (s, 2H), 4.84 – 4.77 (m, 1H), 1.88 – 1.81 (m, 3H), 1.03 (q, J = 2.8 Hz, 6H).
^{13}C NMR (125 MHz, CD_3OD) δ 175.8, 167.3, 153.2, 152.5, 146.9, 140.5, 139.2, 135.5, 134.4, 131.3, 129.1, 129.0, 127.4, 115.2, 114.5, 52.8, 51.4, 41.4, 26.3, 23.4, 21.8.

LCMS (ESI+): calculated for $C_{25}H_{25}ClN_5O_3$ (M+H)$^+$: 478.2; found 478.5.

Compound 40b

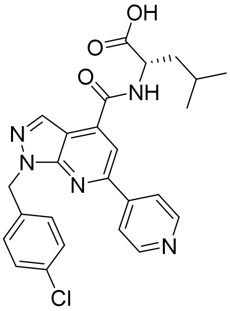

^{1}H NMR (500 MHz, CD_3OD) δ 8.91 (s, 2H), 8.69 (s, 2H), 8.50 (d, *J* = 7.4 Hz, 1H), 8.40 (d, *J* = 2.4 Hz, 1H), 7.51 – 7.25 (m, 4H), 5.86 (d, *J* = 6.1 Hz, 2H), 4.83 – 4.77 (m, 1H), 1.90 – 1.79 (m, 3H), 1.03 (q, *J* = 2.4 Hz, 6H).
^{13}C NMR (125 MHz, CD_3OD) δ 175.8, 167.3, 153.0, 152.4, 146.7, 140.5, 139.2, 137.0, 134.8, 134.3, 131.3, 130.6, 129.8, 129.1, 129.0, 127.4, 115.2, 114.5, 52.8, 51.3, 41.4, 26.3, 23.4, 21.8.
LCMS (ESI+): calculated for $C_{25}H_{25}ClN_5O_3$ (M+H)$^+$: 478.2; found 478.0.

Compound 41b

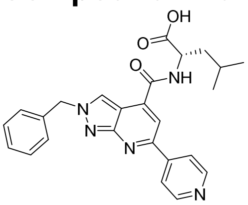

^{1}H NMR (500 MHz, CD_3OD) δ 8.71 (s, 1H), 8.32 (s, 1H), 7.48 – 7.42 (m, 2H), 7.42 – 7.35 (m, 2H), 7.38 – 7.31 (m, 1H), 5.77 (s, 2H), 4.81 – 4.74 (m, 1H), 1.89 – 1.76 (m, 3H), 1.02 (dd, *J* = 5.9, 4.3 Hz, 6H).
^{13}C NMR (125 MHz, CD_3OD) δ 175.9, 167.3, 160.5, 154.7, 146.3, 139.2, 136.7, 130.0, 129.7, 129.5, 127.1, 114.9, 113.3, 59.3, 52.7, 41.3, 26.3, 23.4, 21.8.
LCMS (ESI+): calculated for $C_{25}H_{26}N_5O_3$ (M+H)$^+$: 444.2; found 444.0.

Compounds 42b-47b: commercial vendor

Compound 48b

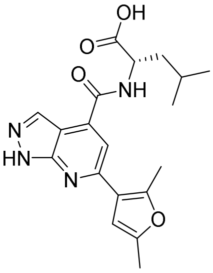

^{1}H NMR (500 MHz, CD_3OD) δ 8.28 (d, *J* = 2.4 Hz, 1H), 7.56 (s, 1H), 6.92 (d, *J* = 2.4 Hz, 1H), 6.74 (d, *J* = 1.3 Hz, 1H), 4.71 (dd, *J* = 9.9, 4.3 Hz, 1H), 2.51 (s, 3H), 2.35 (s, 3H), 1.94 – 1.83 (m, 1H), 1.85 – 1.78 (m, 1H), 1.81 – 1.72 (m, 1H), 1.01 (t, *J* = 6.2 Hz, 6H).
^{13}C NMR (125 MHz, MeOD) δ 175.6, 165.3, 154.3, 152.0, 149.6, 149.2, 146.6, 143.8, 114.3, 108.6, 105.3, 99.2, 52.4, 41.9, 26.4, 26.2, 23.4, 22.0, 14.2, 13.2.
LCMS (ESI+): calculated for $C_{19}H_{23}N_5O_4$ (M+H)$^+$: 371.2; found 371.3.

Compounds 49b-53b: commercial vendor

Compound 54b

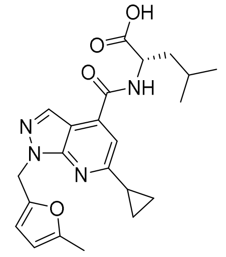

^{1}H NMR (500 MHz, CD_3OD) δ 8.21 (s, 1H), 7.46 (s, 1H), 6.20 (d, *J* = 3.1 Hz, 1H), 5.90 (dd, *J* = 3.1, 1.3 Hz, 1H), 5.54 (s, 2H), 4.73 (dd, *J* = 10.6, 3.8 Hz, 1H), 2.30 (tt, *J* = 8.1, 4.7 Hz, 1H), 2.18 (d, *J* = 1.0 Hz, 3H), 1.87 – 1.72 (m, 3H), 1.26 – 1.15 (m, 2H), 1.13 (dt, *J* = 8.2, 3.1 Hz, 2H), 1.01 (dd, *J* = 5.7, 1.3 Hz, 6H).
^{13}C NMR (125 MHz, CD_3OD) δ 175.8, 168.3, 166.0, 153.4, 152.3, 149.5, 137.1, 133.6, 115.2, 112.1, 110.6, 107.3, 52.6, 44.4, 41.3, 26.3, 23.4, 21.7, 18.5, 13.4, 11.8, 11.7.
LCMS (ESI+): calculated for $C_{22}H_{27}N_4O_4$ $(M+H)^+$: 411.2; found 411.1.

Compounds 55b: commercial vendor

Compound 56b

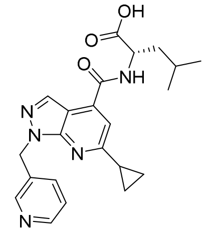

^{1}H NMR (500 MHz, CD_3OD) δ 8.77 (s, 2H), 8.32 (d, *J* = 8.1 Hz, 1H), 8.30 (s, 1H), 7.89 (s, 1H), 7.52 (s, 1H), 5.86 (s, 2H), 4.77 – 4.70 (m, 1H), 2.31 (tt, *J* = 8.0, 4.9 Hz, 1H), 1.88 – 1.80 (m, 1H), 1.80 (dd, *J* = 4.4, 2.1 Hz, 1H), 1.80 – 1.73 (m, 1H), 1.22 – 1.10 (m, 4H), 1.01 (dd, *J* = 6.1, 3.3 Hz, 6H).
^{13}C NMR (125 MHz, CD_3OD) δ 175.8, 168.0, 166.6, 152.7, 144.5, 144.4, 144.4, 137.4, 134.8, 115.6, 112.5, 52.6, 41.2, 26.3, 23.4, 21.7, 18.5, 12.0, 11.9.
LCMS (ESI+): calculated for $C_{22}H_{26}N_5O_3$ $(M+H)^+$: 408.2; found 408.5.

Compound 57b

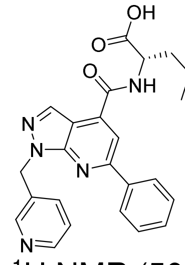

^{1}H NMR (500 MHz, CD_3OD) δ 8.41 (s, 1H), 8.35 (d, *J* = 7.8 Hz, 1H), 8.28 – 8.22 (m, 2H), 8.18 (s, 1H), 7.54 (dd, *J* = 8.2, 6.1 Hz, 2H), 7.54 – 7.45 (m, 1H), 6.01 (s, 2H), 4.78 (dd, *J* = 9.9, 4.1 Hz, 1H), 1.91 – 1.83 (m, 1H), 1.85 – 1.76 (m, 2H), 1.02 (d, *J* = 5.5 Hz, 6H).
^{13}C NMR (125 MHz, CD_3OD) δ 175.9, 167.8, 159.1, 152.8, 139.6, 138.4, 134.9, 131.2, 130.0, 128.7, 114.1, 113.3, 52.7, 41.3, 26.4, 23.4, 21.8.

LCMS (ESI+): calculated for $C_{25}H_{26}N_5O_3$ (M+H)$^+$: 444.2; found 444.5.

Compound 58b

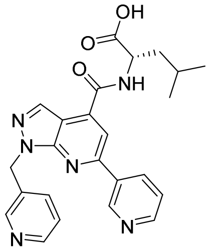

^{1}H NMR (500 MHz, CD_3OD) δ 9.64 (s, 1H), 9.18 (dt, *J* = 8.2, 1.7 Hz, 1H), 8.94 (s, 1H), 8.88 (s, 1H), 8.77 – 8.72 (m, 1H), 8.52 – 8.45 (m, 2H), 8.35 (s, 1H), 8.04 (dd, *J* = 8.2, 5.3 Hz, 1H), 7.92 (dd, *J* = 8.1, 5.5 Hz, 1H), 6.08 (s, 2H), 4.84 – 4.76 (m, 1H), 1.91 – 1.82 (m, 2H), 1.85 – 1.78 (m, 1H), 1.03 (d, *J* = 6.0 Hz, 6H).
^{13}C NMR (125 MHz, CD_3OD) δ 175.8, 167.1, 153.3, 152.6, 146.2, 145.3, 145.0, 144.2, 144.1, 142.2, 139.2, 138.1, 135.3, 128.0, 127.6, 114.7, 114.1, 52.8, 41.3, 26.3, 23.4, 21.8.
LCMS (ESI+): calculated for $C_{24}H_{25}N_6O_3$ (M+H)$^+$: 445.2; found 445.1.

Compound 59b

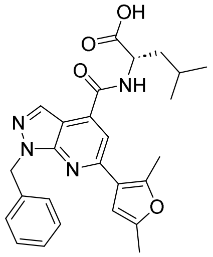

^{1}H NMR (500 MHz, CD_3OD) δ 8.29 (s, 1H), 7.73 (s, 1H), 7.33 – 7.25 (m, 4H), 7.24 (ddd, *J* = 10.1, 5.0, 2.5 Hz, 1H), 6.59 (d, *J* = 1.3 Hz, 1H), 5.72 (s, 2H), 4.79 – 4.73 (m, 1H), 2.67 (s, 3H), 2.31 (s, 3H), 1.89 – 1.76 (m, 3H), 1.02 (d, *J* = 5.8 Hz, 6H).
^{13}C NMR (125 MHz, CD_3OD) δ 175.9, 168.2, 155.4, 152.8, 152.3, 151.5, 138.6, 137.8, 133.7, 129.6, 128.8, 128.7, 121.7, 114.5, 111.6, 107.0, 51.8, 41.3, 26.4, 23.4, 21.8, 14.7, 13.3.
LCMS (ESI+): calculated for $C_{26}H_{29}N_4O_4$ (M+H)$^+$: 461.2; found 461.4.

Compound 60b

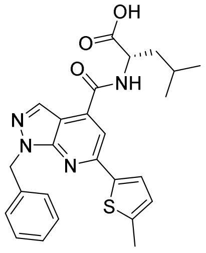

^{1}H NMR (500 MHz, CD_3OD) δ 8.26 (s, 1H), 7.97 (s, 1H), 7.70 (d, *J* = 3.7 Hz, 1H), 7.37 (d, *J* = 7.4 Hz, 2H), 7.29 (t, *J* = 7.4 Hz, 2H), 7.24 (t, *J* = 7.3 Hz, 1H), 6.85 (d, *J* = 3.7 Hz, 1H), 5.68 (s, 2H), 4.79 – 4.73 (m, 1H), 3.31 (s, 5H), 2.54 (s, 3H), 1.89 – 1.76 (m, 3H), 1.02 (d, *J* = 5.3 Hz, 6H).
^{13}C NMR (125 MHz, CD_3OD) δ 175.9, 168.0, 154.2, 152.1, 145.7, 143.2, 138.4, 137.9, 133.9, 129.6, 129.2, 128.8, 128.5, 127.9, 112.4, 112.1, 52.7, 51.7, 41.3, 26.3, 23.4, 21.8, 15.6.
LCMS (ESI+): calculated for $C_{25}H_{27}N_4O_3S$ (M+H)$^+$: 463.2; found 463.1.

Diverse library LCMS spectra

Compound 1

MaxPeak: 95.38%
Ret_Time: 1.348 min

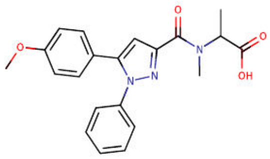

Mol Wt 379.41

Exact Mass 379.17

#	Time	Area%
1	1.311	4.62
2	1.348	95.38

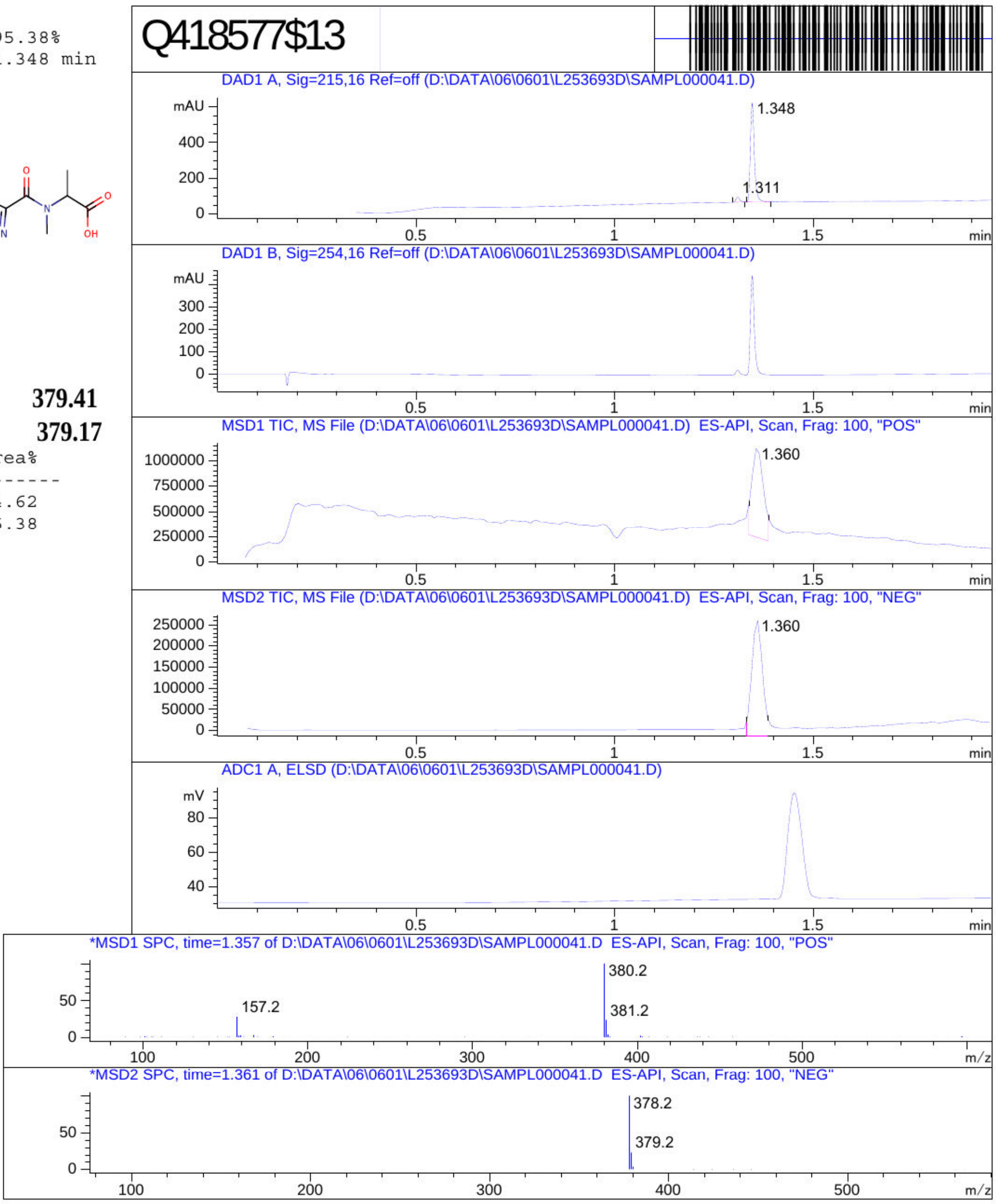

RT 1.360

RT 1.360

Inj.Date 5/31/2020 LT -5- Acq. Method C:\CHEM32\-> ->

Compound 2

MaxPeak: 94.52%
Ret_Time: 1.249 min

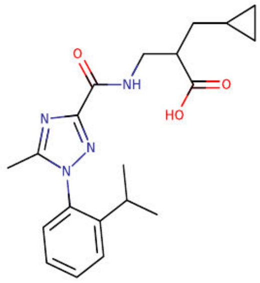

Mol Wt 370.44
Exact Mass 370.23

#	Time	Area%
1	1.249	94.52
2	1.263	5.48

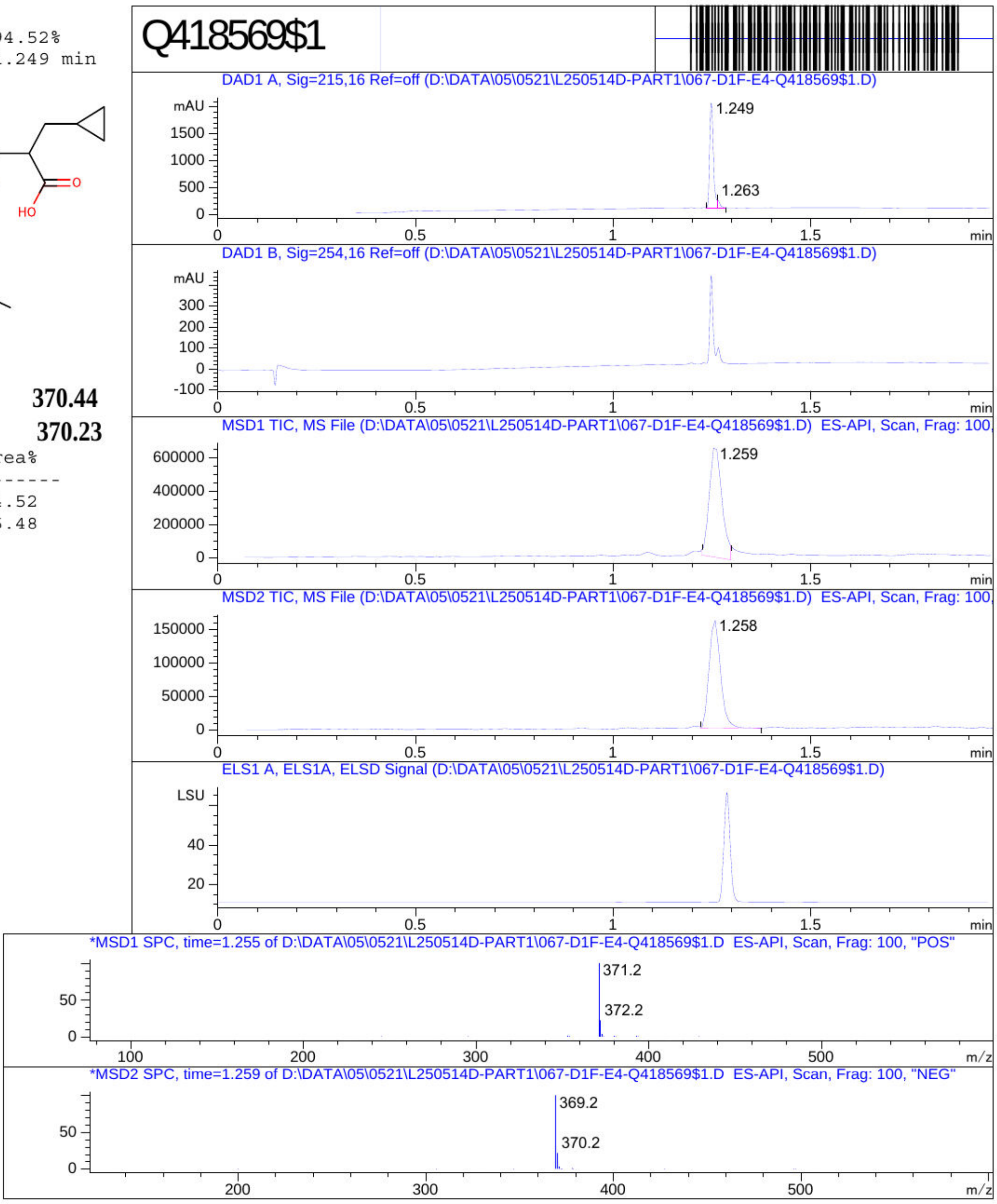

RT 1.259

RT 1.258

Inj.Date 5/21/2020 LT -15- Acq. Method C:\Chem32\-> ->

Compound 3

MaxPeak: 95.88%
Ret_Time: 1.356 min

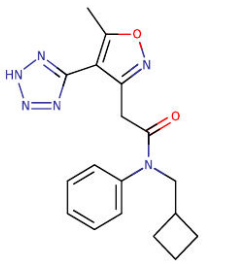

Mol Wt 352.39
Exact Mass 352.18

#	Time	Area%
1	0.834	4.12
2	1.356	95.88

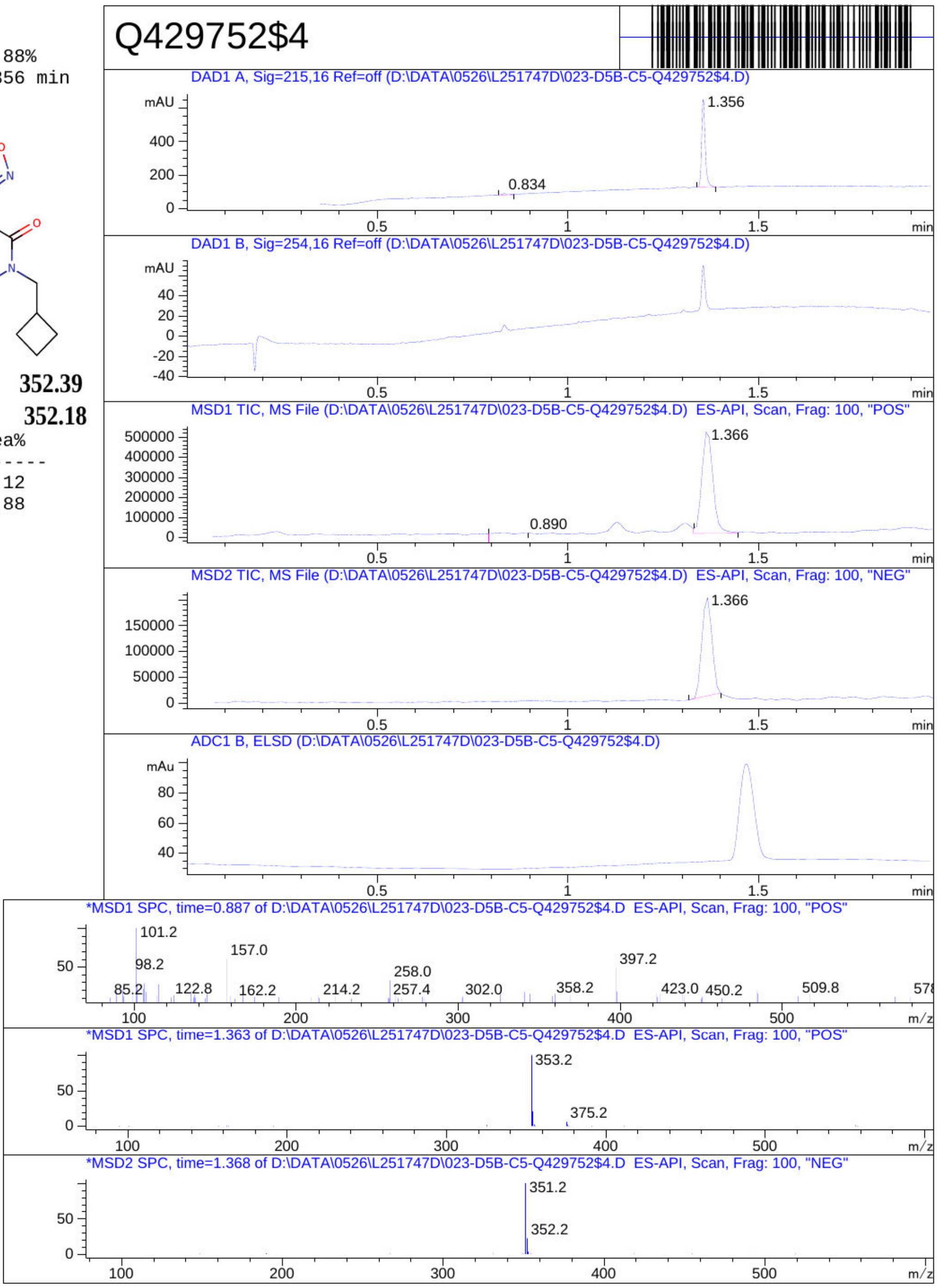

RT 0.890

RT 1.366

RT 1.366

Inj.Date 5/26/2020 OA -10- Acq. Method C:\Chem32\-> ->

Compound 4

MaxPeak: 97.62%
Ret_Time: 1.156 min

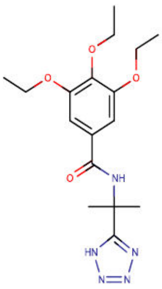

Mol Wt 363.41

Exact Mass 363.21

#	Time	Area%
1	1.104	2.38
2	1.156	97.62

Q418559$1

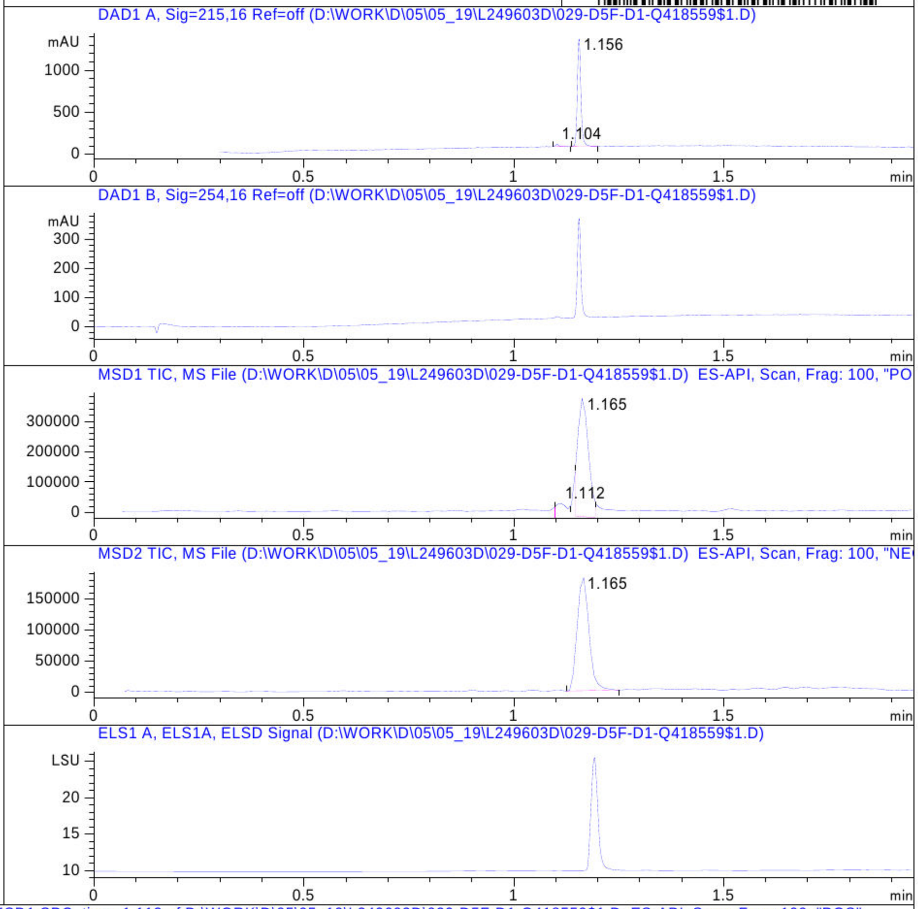

RT 1.112

RT 1.165

RT 1.165

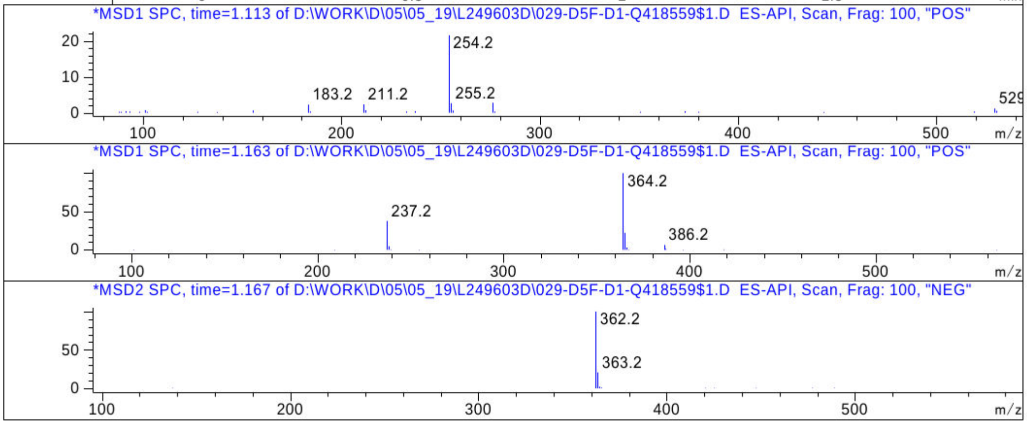

Inj.Date 5/19/2020 K -16- Acq. Method C:\Chem32\-> ->

Compound 5

MaxPeak: 100.00%
Ret_Time: 1.003 min

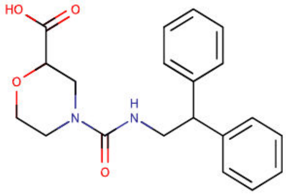

Mol Wt 354.4
Exact Mass 354.18

#	Time	Area%
1	1.003	100.00

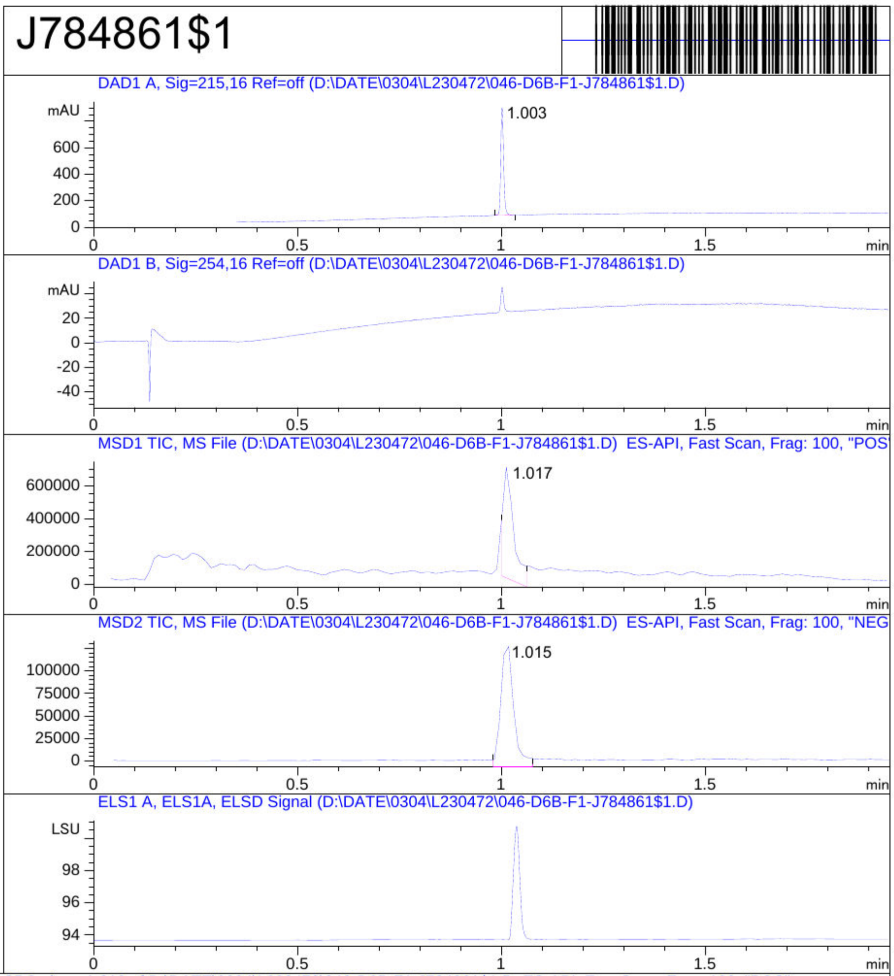

RT 1.017

RT 1.015

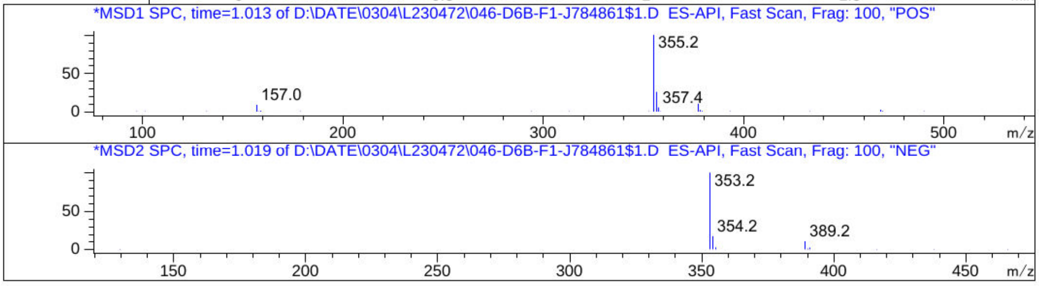

Inj.Date 3/4/2020 E Acq. Method C:\Users\ -> ->

Compound 6

MaxPeak: 100.00%
Ret_Time: 1.523 min

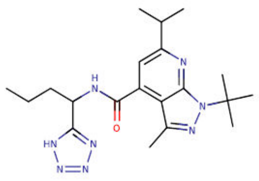

Mol Wt 398.5
Exact Mass 398.29

#	Time	Area%
1	1.523	100.00

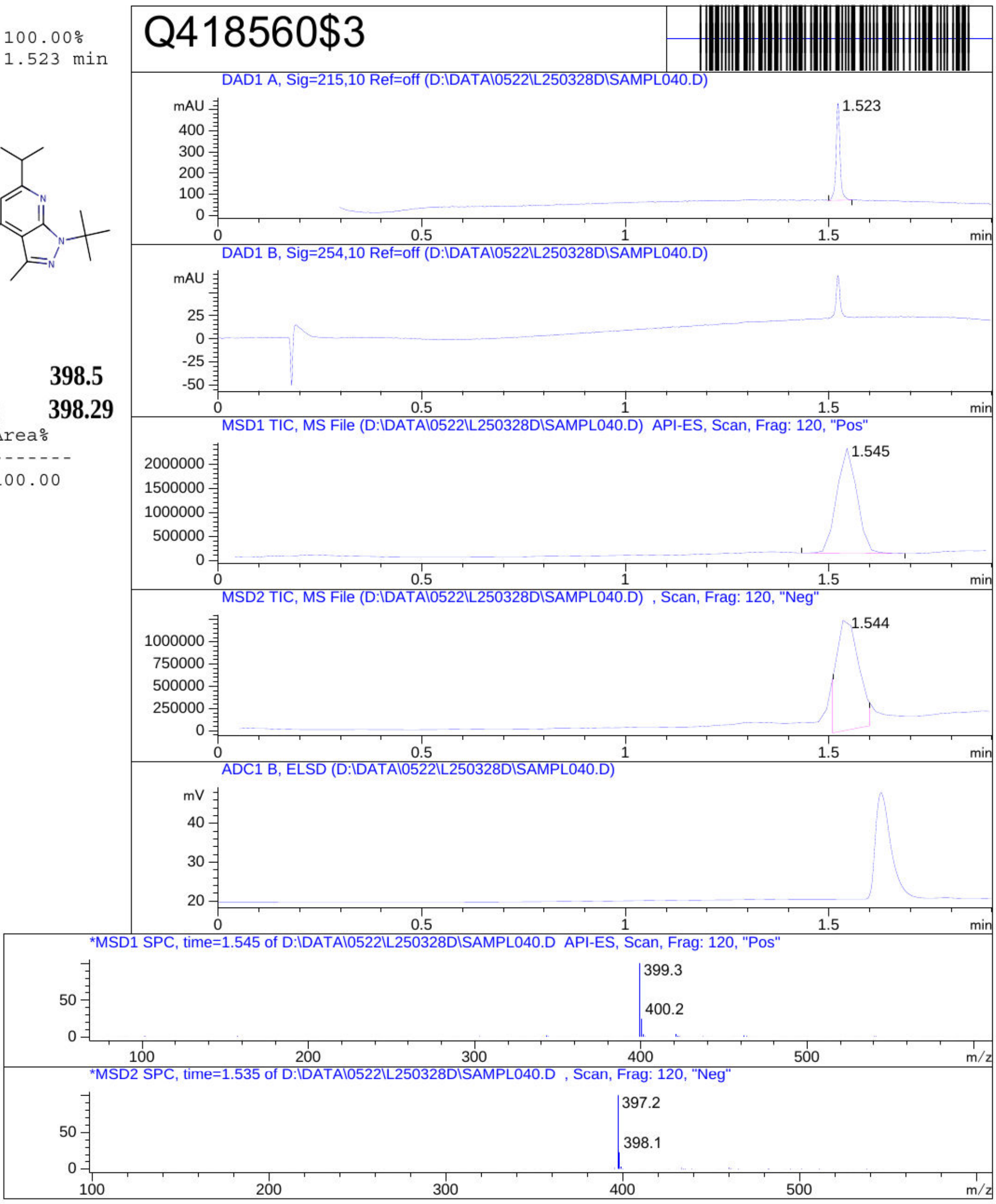

RT 1.545

RT 1.544

Inj.Date 5/22/2020 M -SL- Acq. Method C:\HPCHEM\-> ->

Compound 7

MaxPeak: 100.00%
Ret_Time: 1.252 min

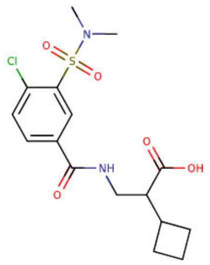

Mol Wt 388.87
Exact Mass 388.1

#	Time	Area%
1	1.252	100.00

RT 1.274

RT 1.276

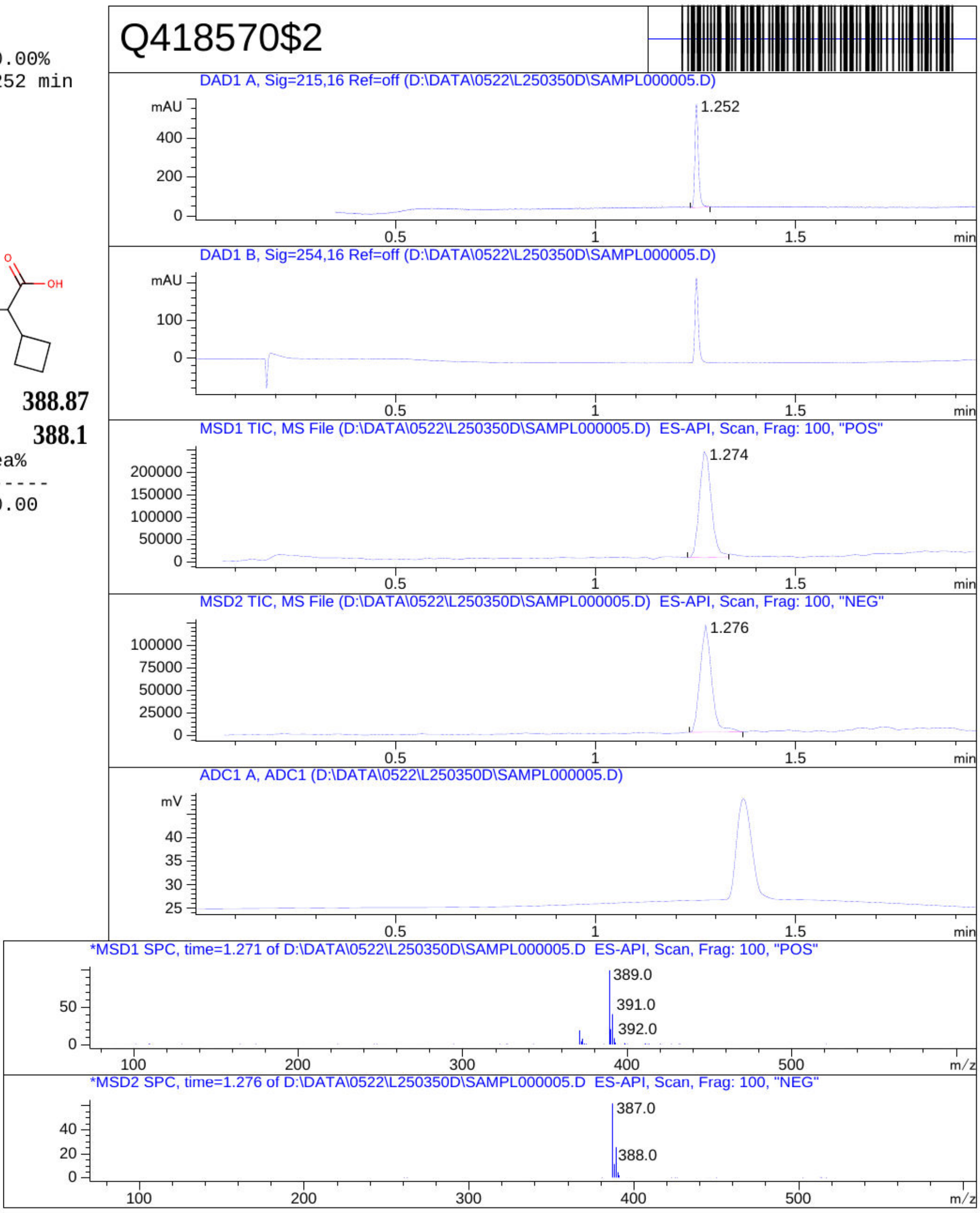

Compound 8

MaxPeak: 100.00%
Ret_Time: 1.197 min

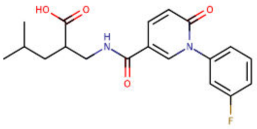

Mol Wt 360.38
Exact Mass 360.17

#	Time	Area%
1	1.197	100.00

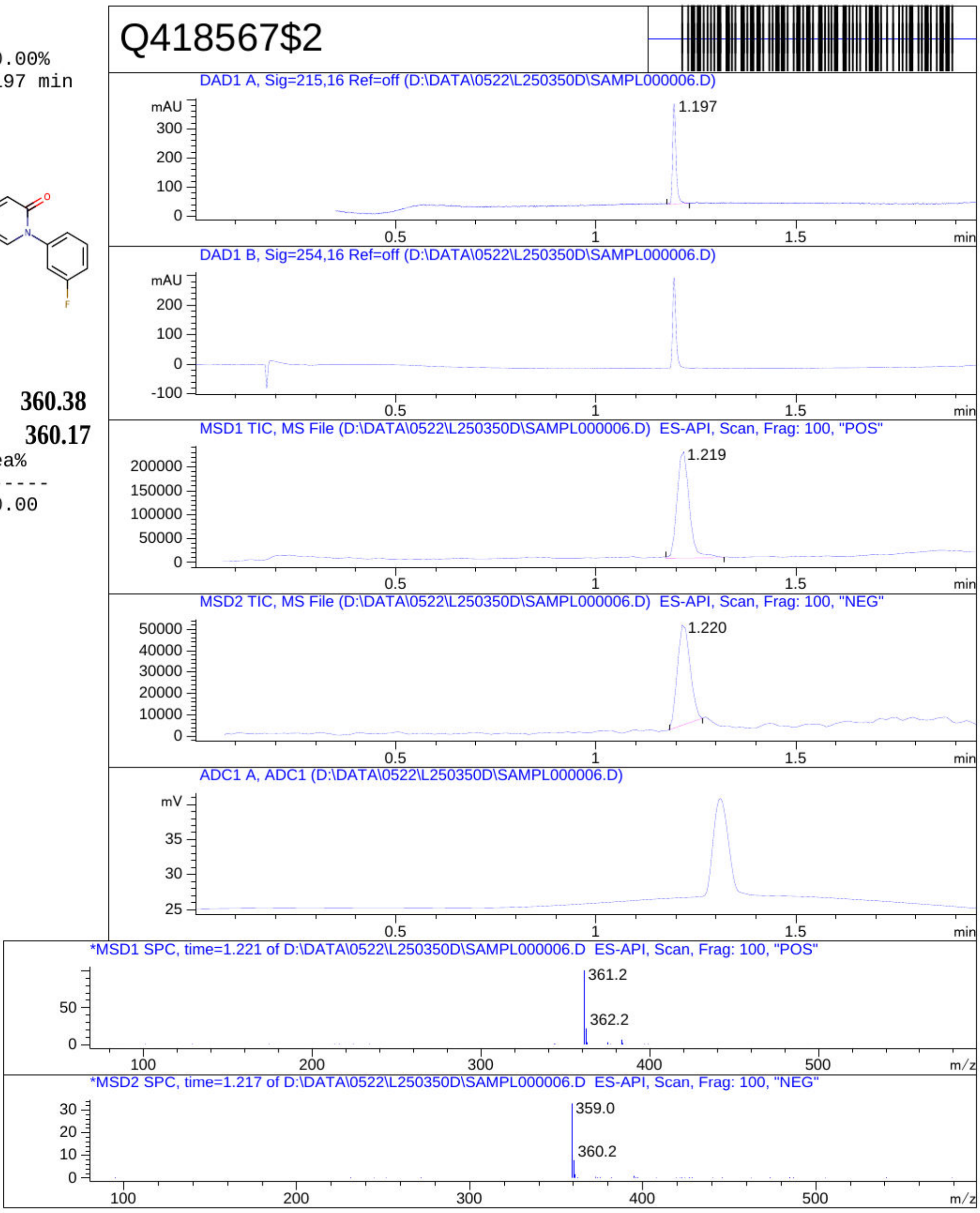

RT 1.219

RT 1.220

Inj.Date 5/21/2020 OA - 4 - Acq. Method C:\CHEM32\-> ->

Compound 9

MaxPeak: 100.00%
Ret_Time: 1.122 min

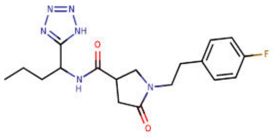

Mol Wt 374.41
Exact Mass 374.21

#	Time	Area%
1	1.122	100.00

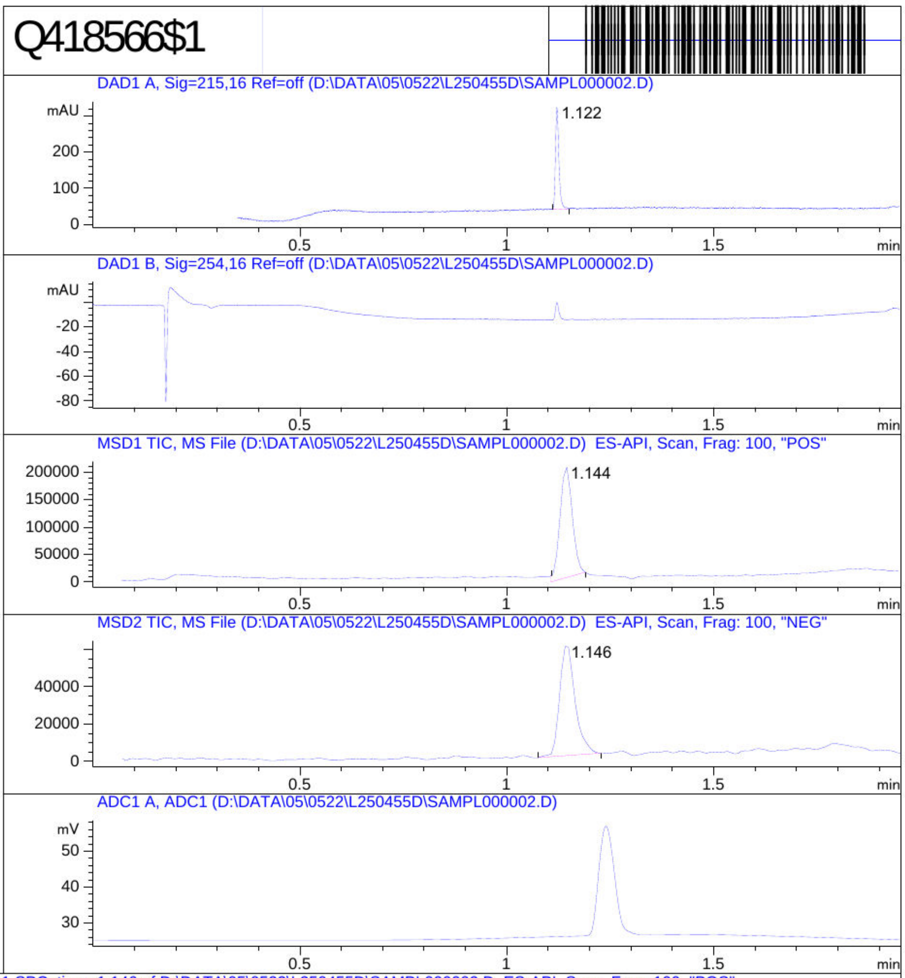

RT 1.144

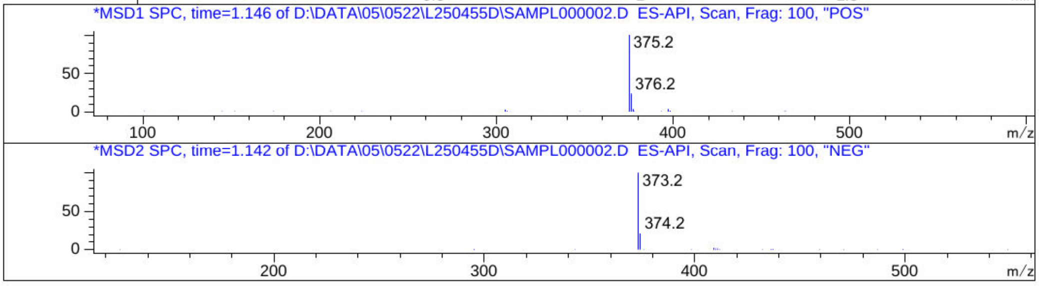

RT 1.146

Compound 10

```
MaxPeak:  90.47%
Ret_Time: 1.114 min
```

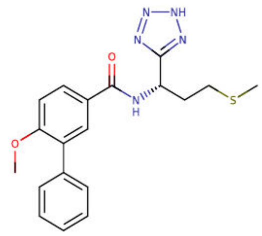

Mol Wt 383.47

Exact Mass 383.16

#	Time	Area%
1	1.102	9.53
2	1.114	90.47

RT 1.123

RT 1.124

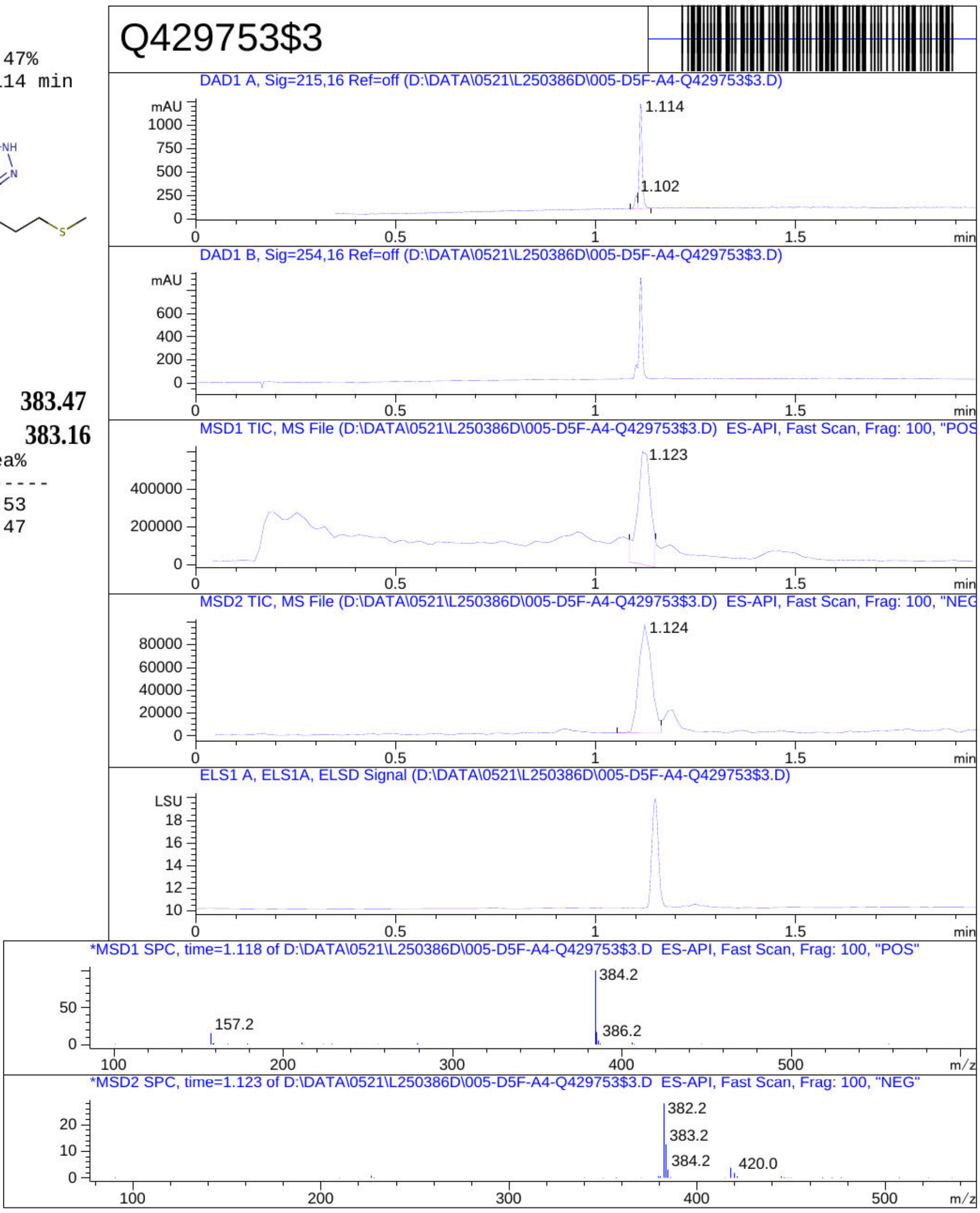

Inj.Date 5/21/2020 OA Acq. Method C:\Users\ -> ->

Compound 11

MaxPeak: 100.00%
Ret_Time: 1.309 min

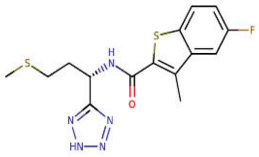

Mol Wt 365.45

Exact Mass 365.09

#	Time	Area%
1	1.309	100.00

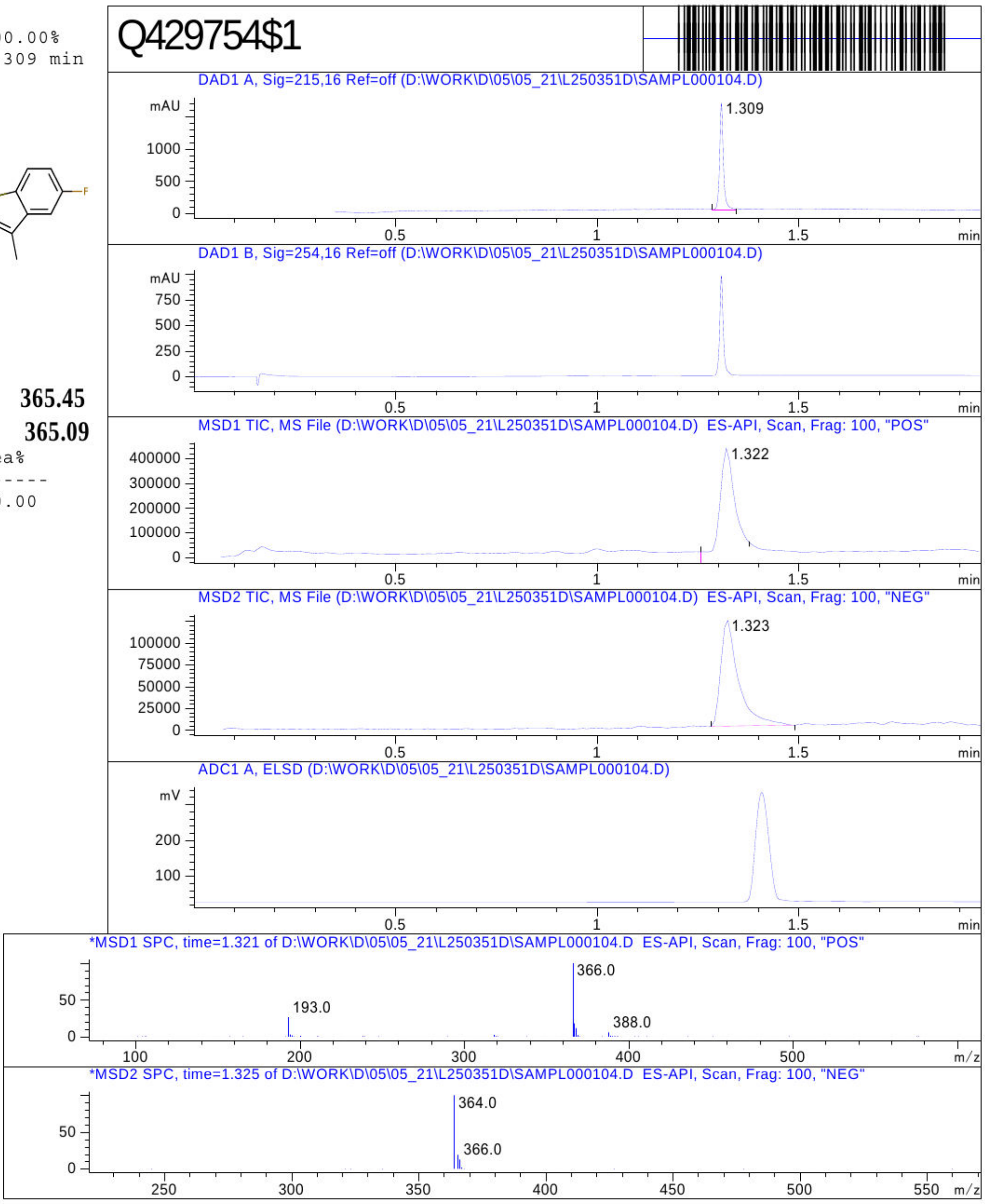

RT 1.322

RT 1.323

Inj.Date 5/21/2020 K -6- Acq. Method C:\CHEM32\-> ->

Compound 12

MaxPeak: 100.00%
Ret_Time: 1.313 min

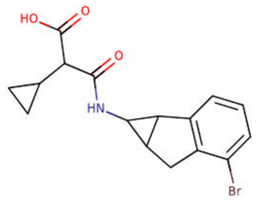

Mol Wt 350.21
Exact Mass 349.05

#	Time	Area%
1	1.313	100.00

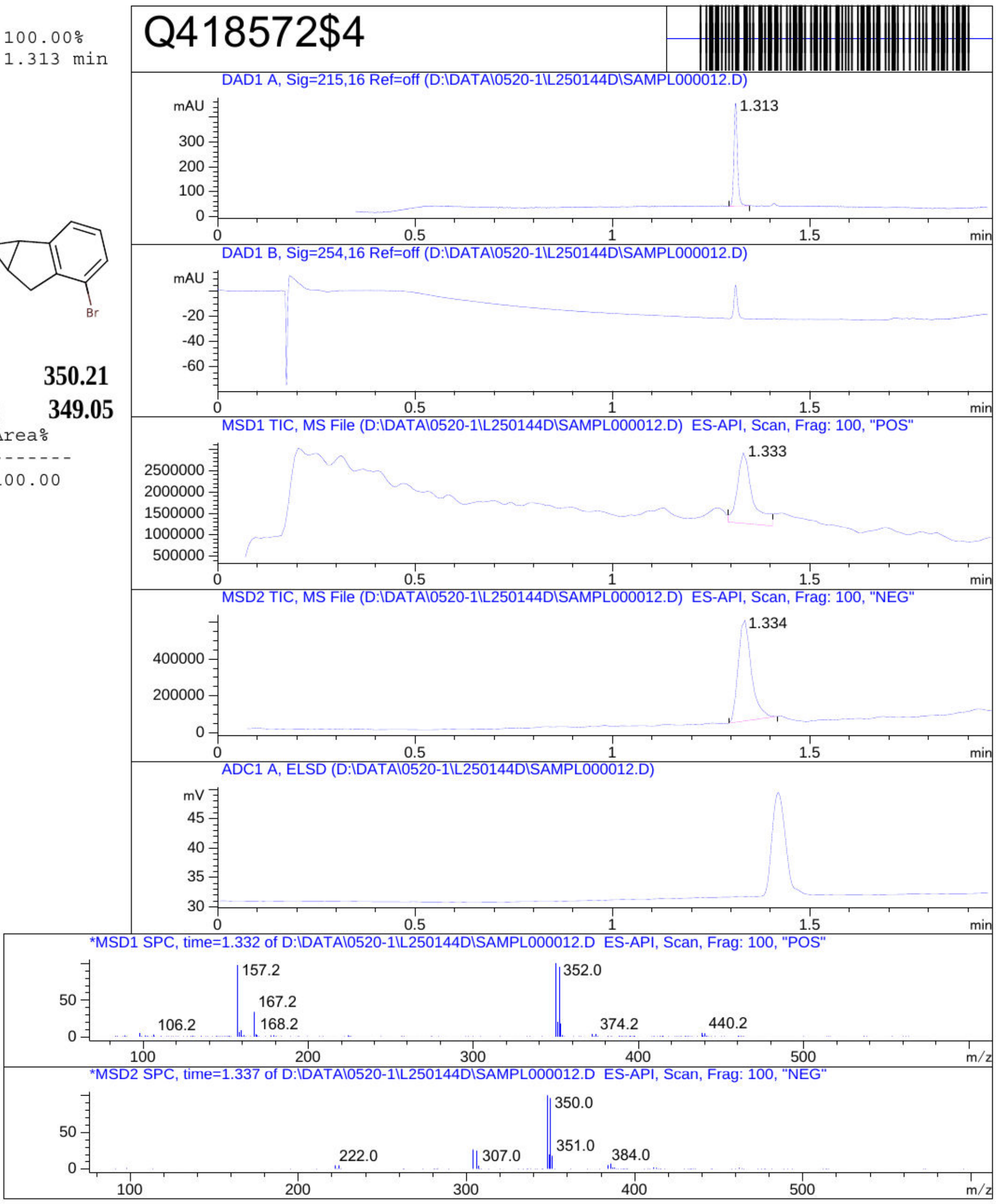

RT 1.333

RT 1.334

Inj.Date 5/20/2020 M -3- Acq. Method C:\CHEM32\-> ->

Compound 13

MaxPeak: 95.20%
Ret_Time: 1.476 min

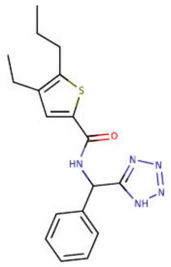

Mol Wt 355.46

Exact Mass 355.17

#	Time	Area%
1	1.434	4.80
2	1.476	95.20

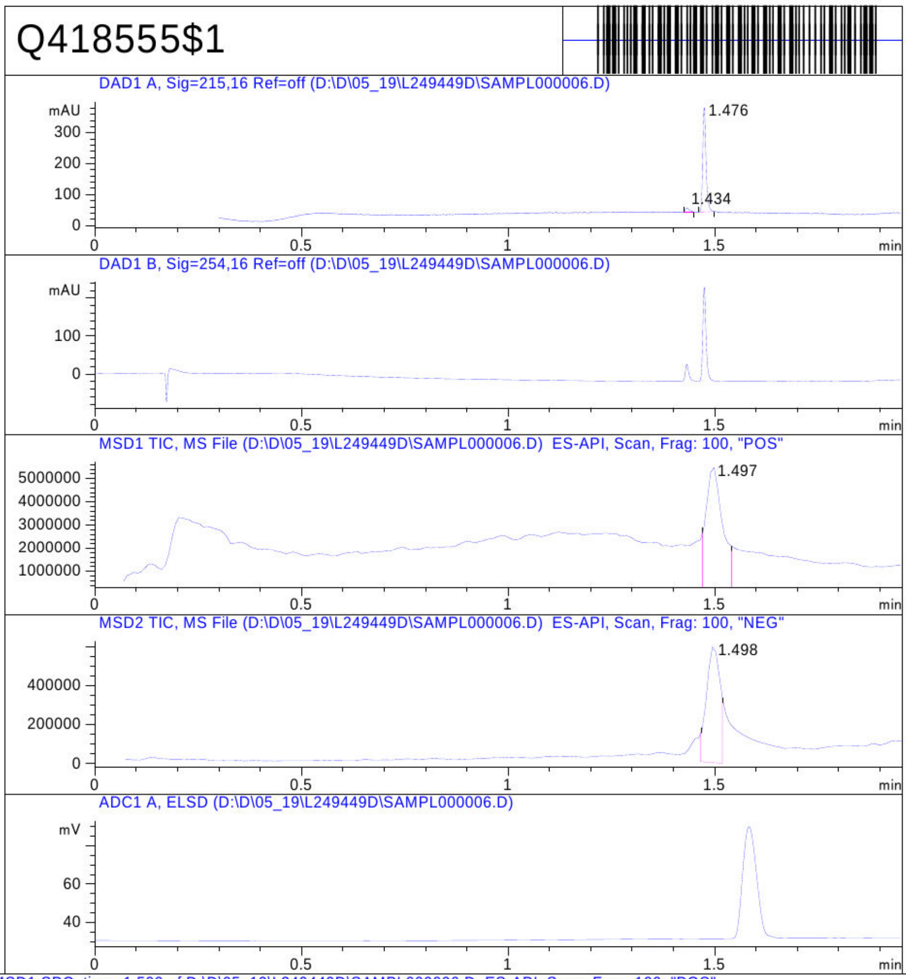

RT 1.497

RT 1.498

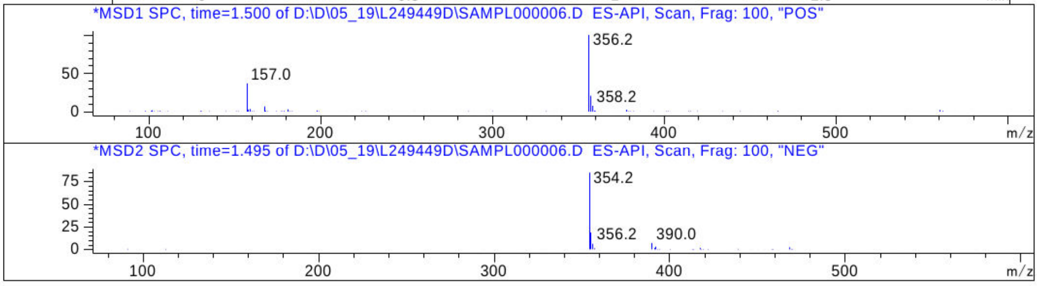

Inj.Date 5/19/2020 N -3- Acq. Method C:\CHEM32\-> ->

Compound 14

MaxPeak: 98.04%
Ret_Time: 1.201 min

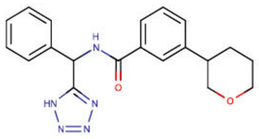

Mol Wt 363.41

Exact Mass 363.19

#	Time	Area%
1	1.118	1.96
2	1.201	98.04

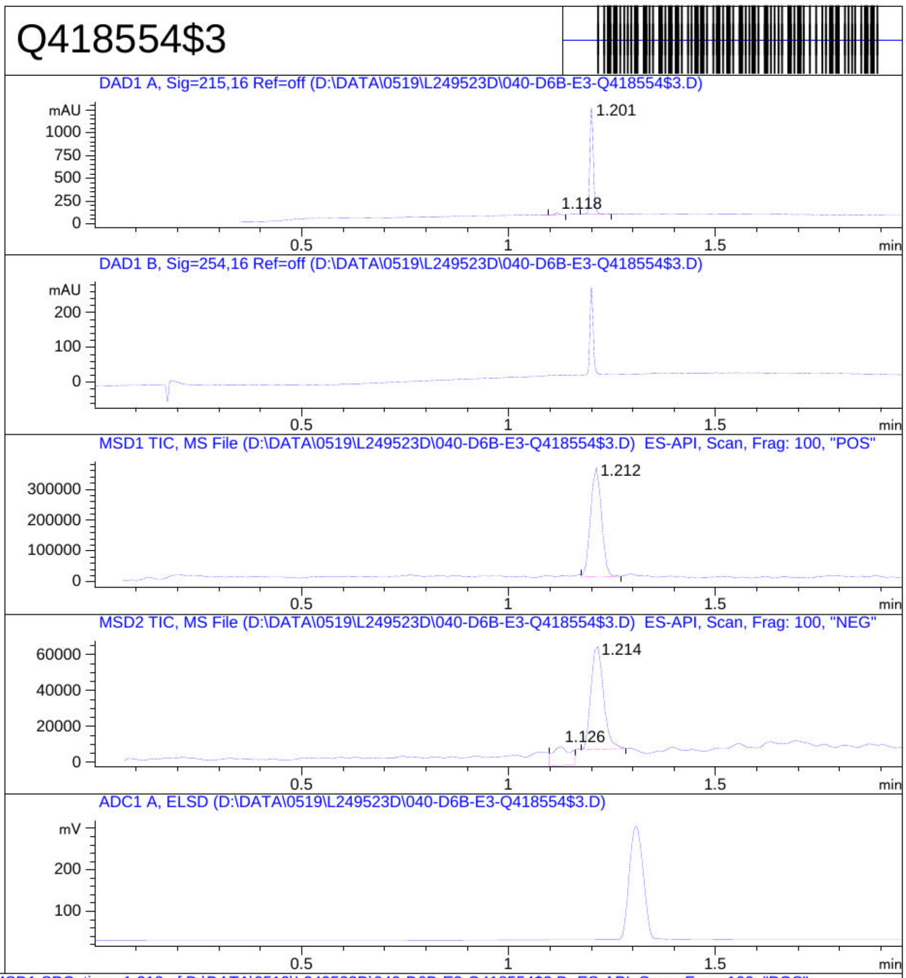

RT 1.212

RT 1.126

RT 1.214

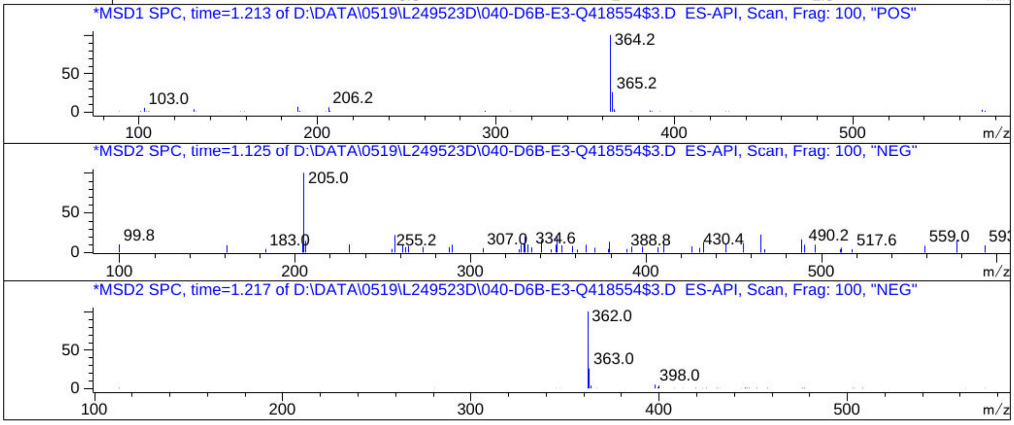

Inj.Date 5/18/2020 OA -7- Acq. Method C:\Chem32\-> ->

Compound 15

MaxPeak: 100.00%
Ret_Time: 0.926 min

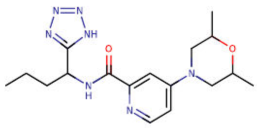

Mol Wt 359.43
Exact Mass 359.23

#	Time	Area%
1	0.926	100.00

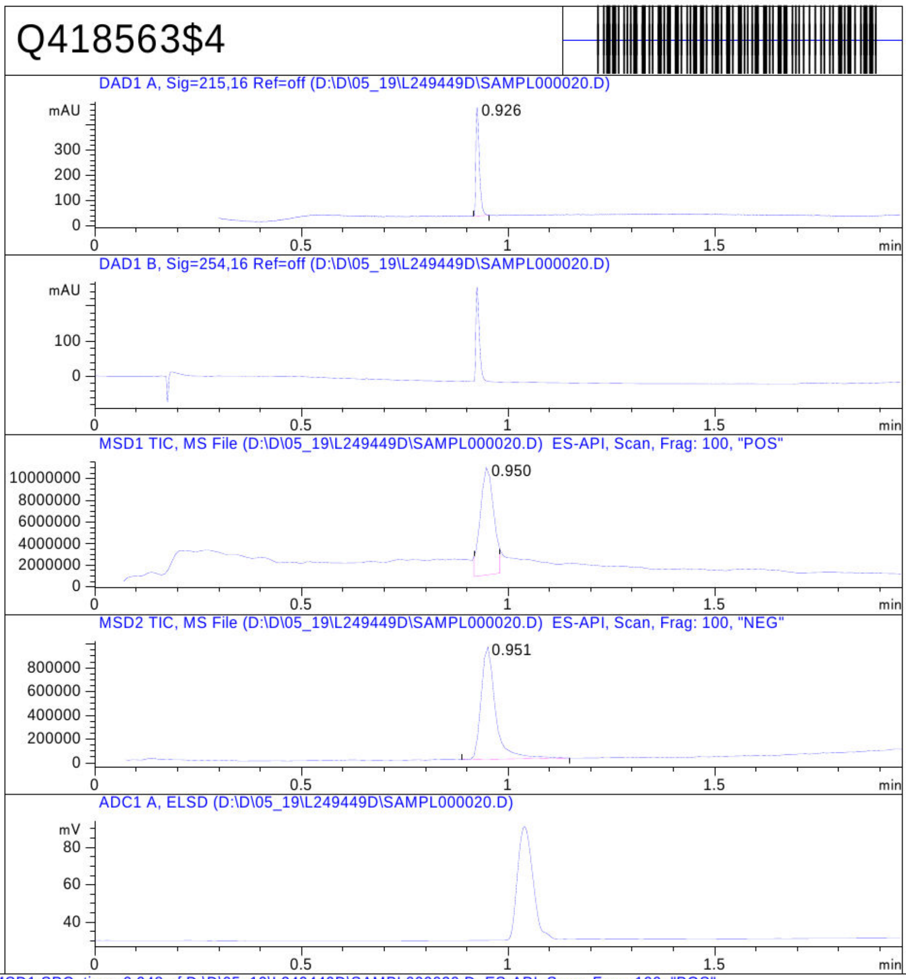

RT 0.950

RT 0.951

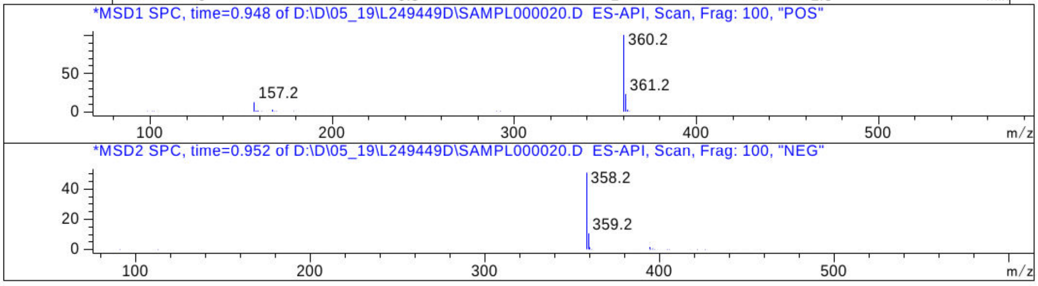

Compound 16

MaxPeak: 100.00%
Ret_Time: 1.094 min

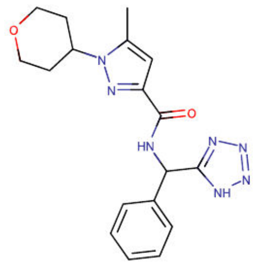

Mol Wt 367.4

Exact Mass 367.19

#	Time	Area%
1	1.094	100.00

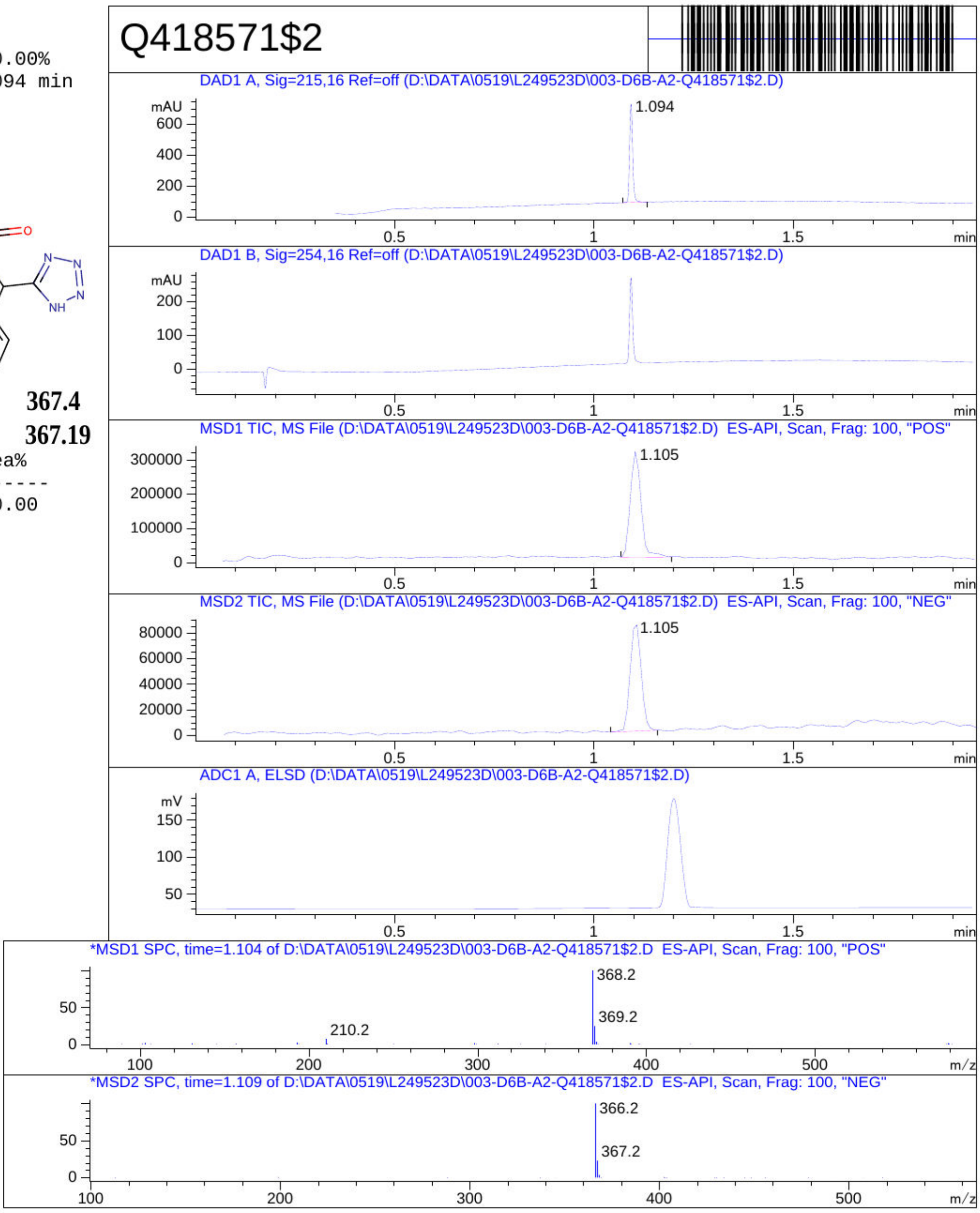

RT 1.105

RT 1.105

Compound 17

MaxPeak: 100.00%
Ret_Time: 0.970 min

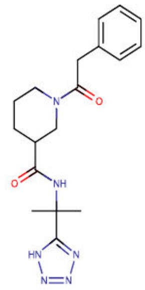

Mol Wt 356.42

Exact Mass 356.22

#	Time	Area%
1	0.970	100.00

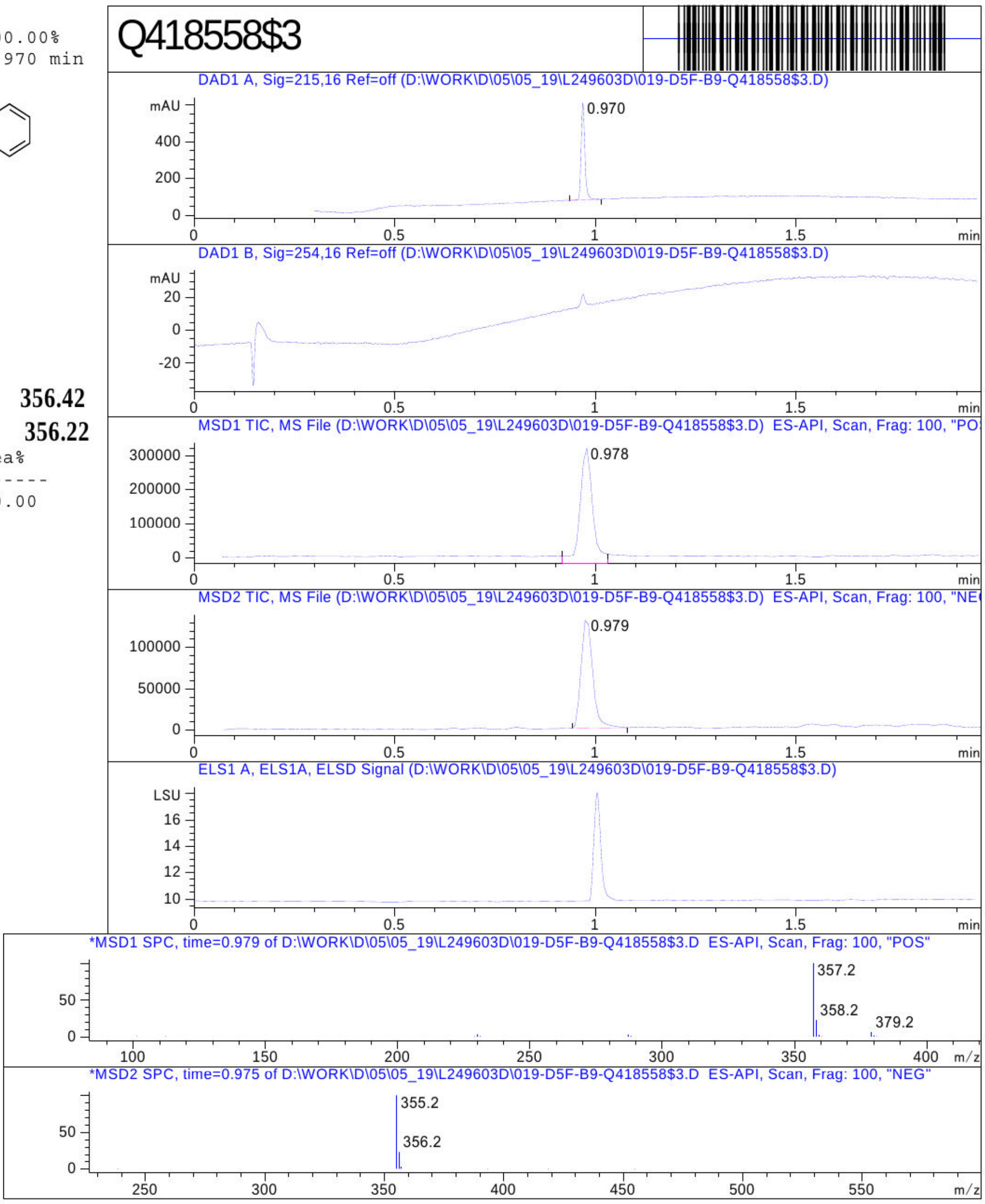

RT 0.978

RT 0.979

Inj.Date 5/19/2020 K -16- Acq. Method C:\Chem32\-> ->

Compound 18

```
MaxPeak:  92.16%
Ret_Time: 1.130 min
```

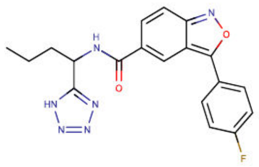

Mol Wt 380.38

Exact Mass 380.15

#	Time	Area%
1	1.101	7.84
2	1.130	92.16

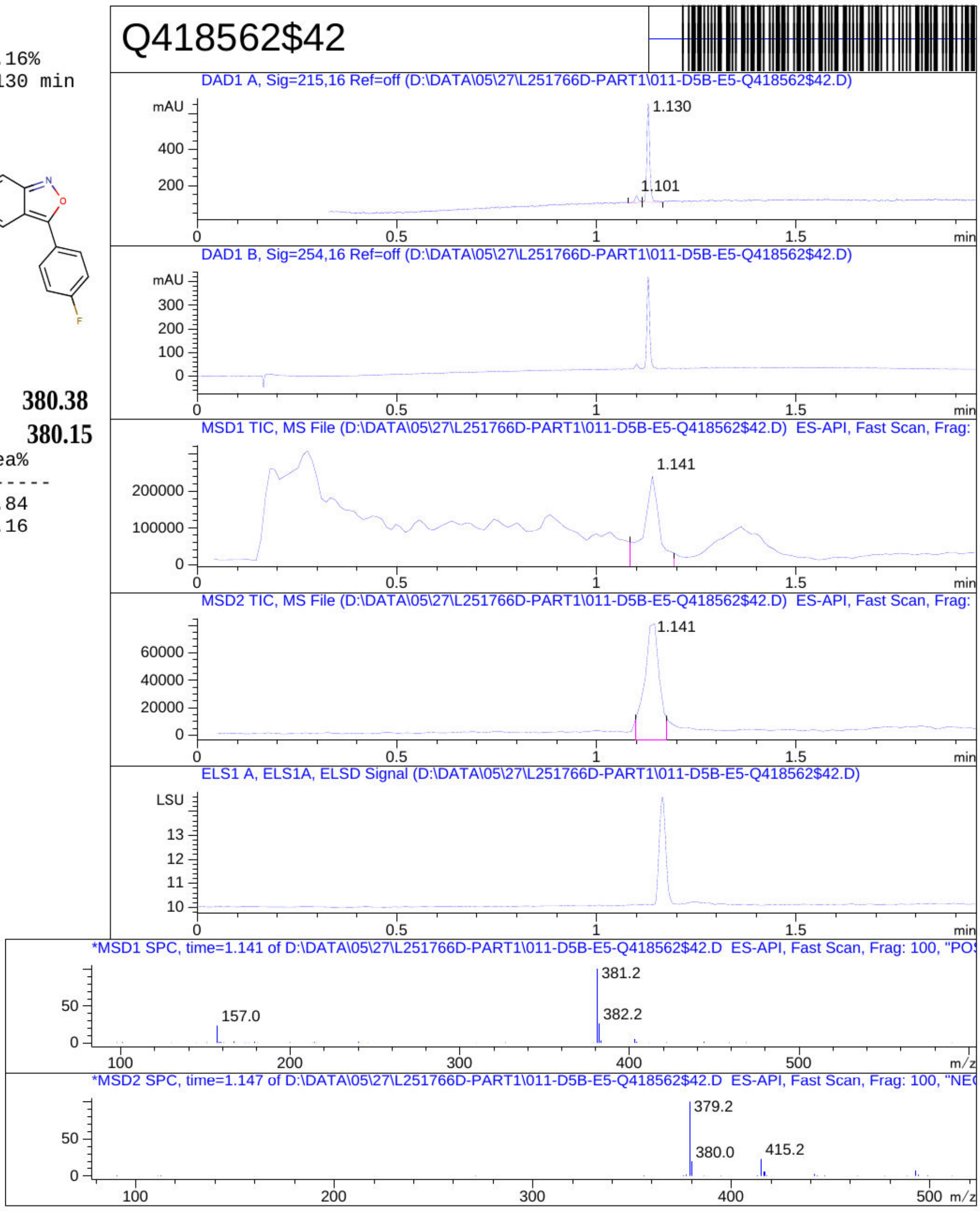

RT 1.141

RT 1.141

Inj.Date 5/26/2020 LB Acq. Method C:\Users\ -> ->

Compound 19

MaxPeak: 100.00%
Ret_Time: 1.839 min

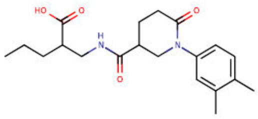

Mol Wt 360.45

Exact Mass 360.24

#	Time	Area%
1	1.839	100.00

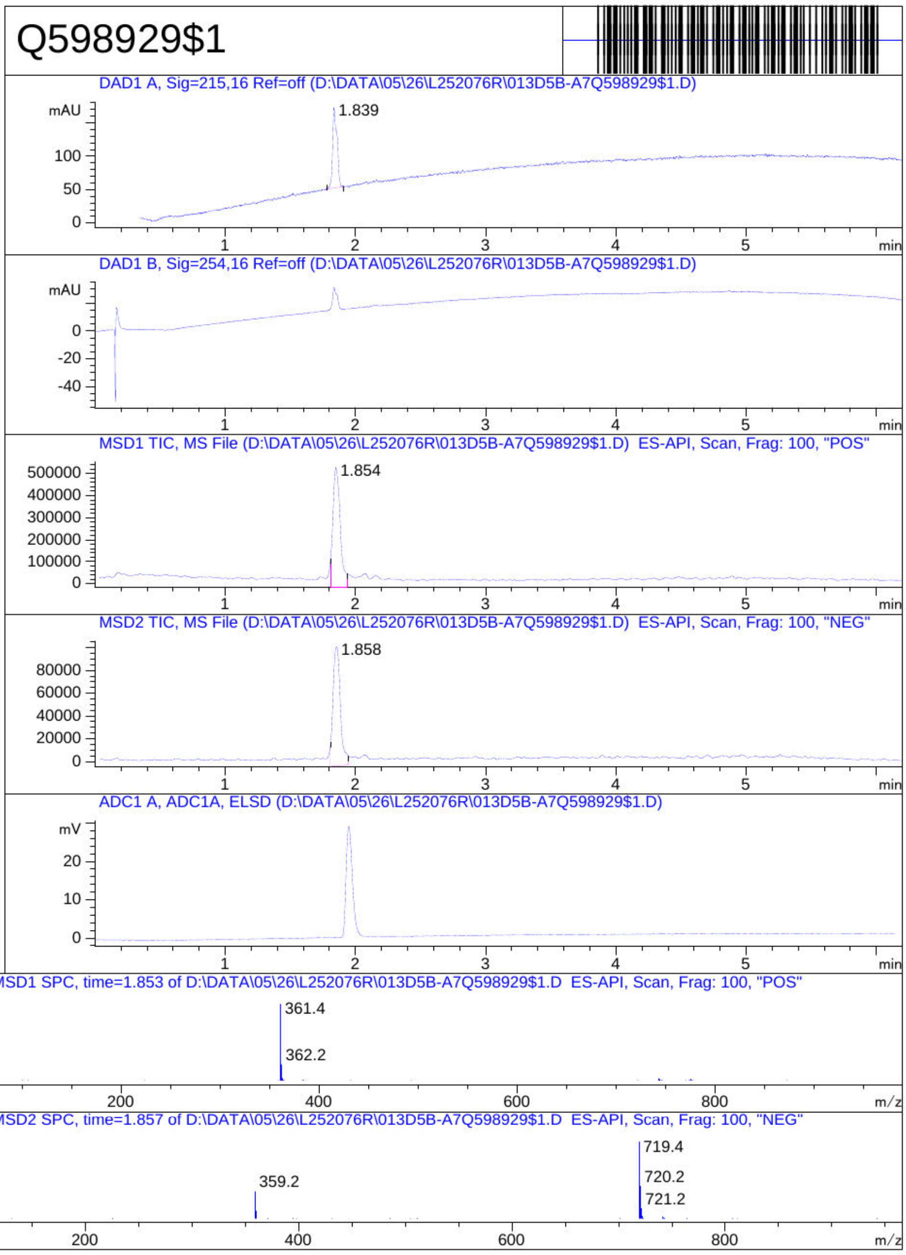

Inj.Date 5/26/2020 LB -11- Acq. Method C:\Chem32\-> ->

Compound 20

MaxPeak: 100.00%
Ret_Time: 1.161 min

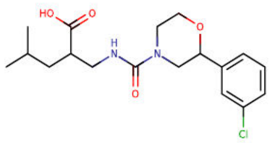

Mol Wt 368.86
Exact Mass 368.18

#	Time	Area%
1	1.161	100.00

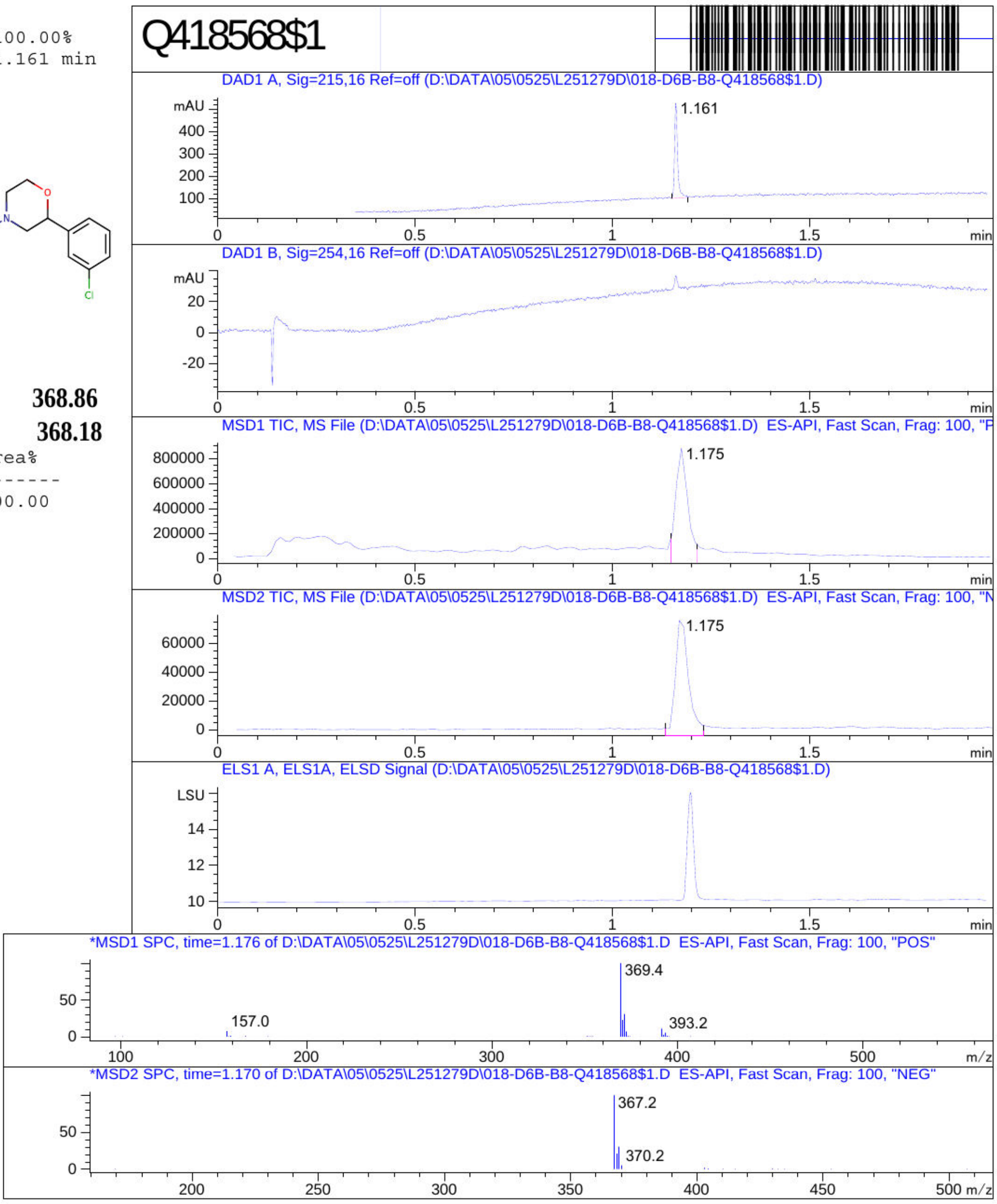

RT 1.175

RT 1.175

Inj.Date 5/22/2020 LT Acq. Method C:\Users\ -> ->

Compound 21

MaxPeak: 100.00%
Ret_Time: 1.146 min

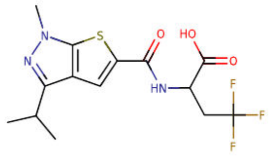

Mol Wt 363.36
Exact Mass 363.1

#	Time	Area%
1	1.146	100.00

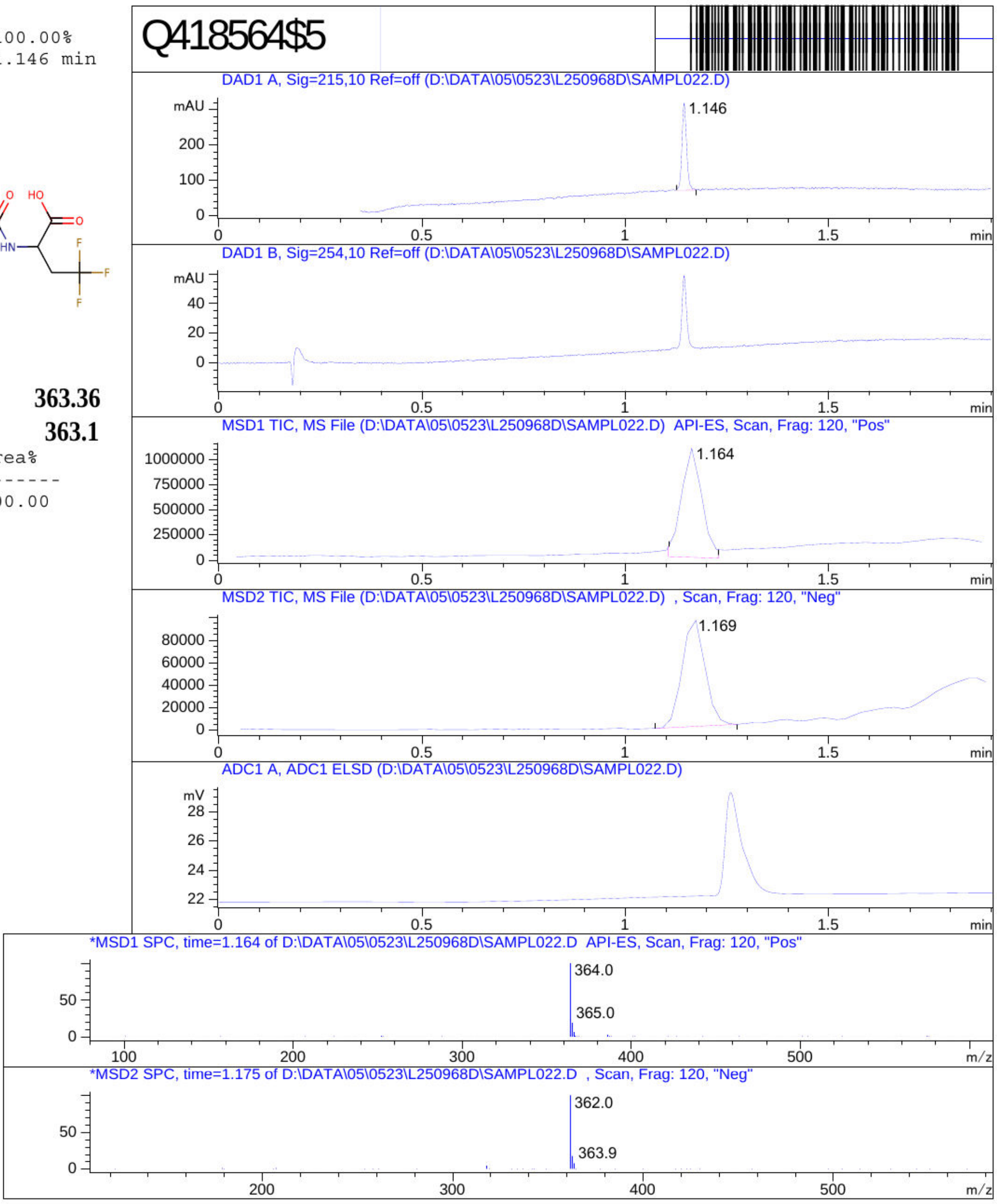

RT 1.164

RT 1.169

Inj.Date 5/23/2020 LT -VL- Acq. Method C:\HPCHEM\-> ->

Compound 22

MaxPeak: 100.00%
Ret_Time: 1.316 min

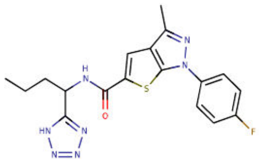

Mol Wt 399.44
Exact Mass 399.14

#	Time	Area%
1	1.316	100.00

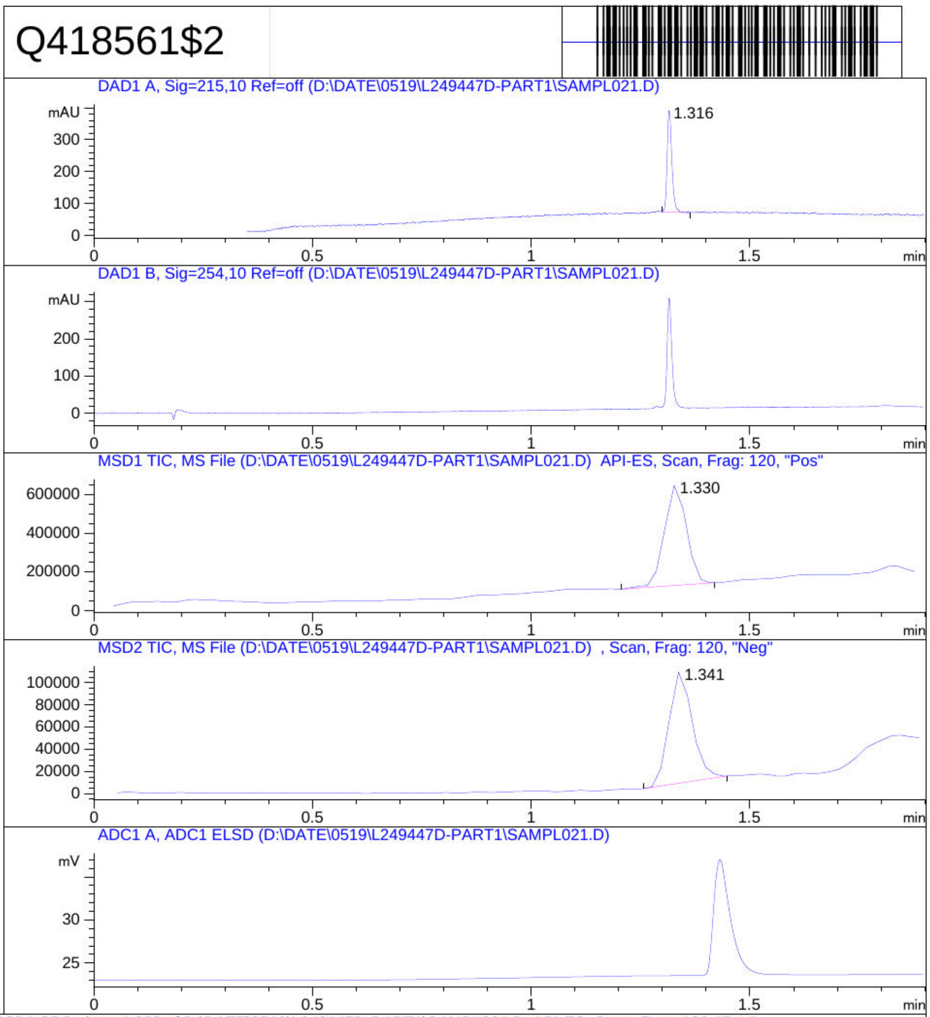

RT 1.330

RT 1.341

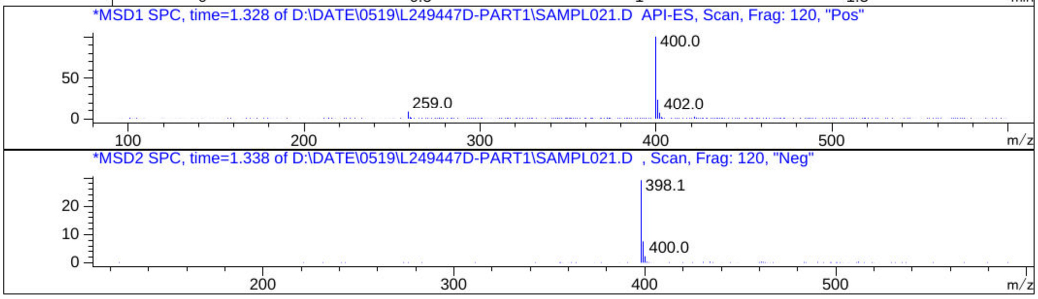

Inj.Date 5/19/2020 O -VL- Acq. Method C:\HPCHEM\-> ->

Compound 23

MaxPeak: 100.00%
Ret_Time: 1.367 min

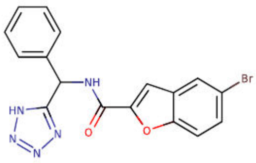

Mol Wt 398.21
Exact Mass 397.02

#	Time	Area%
1	1.367	100.00

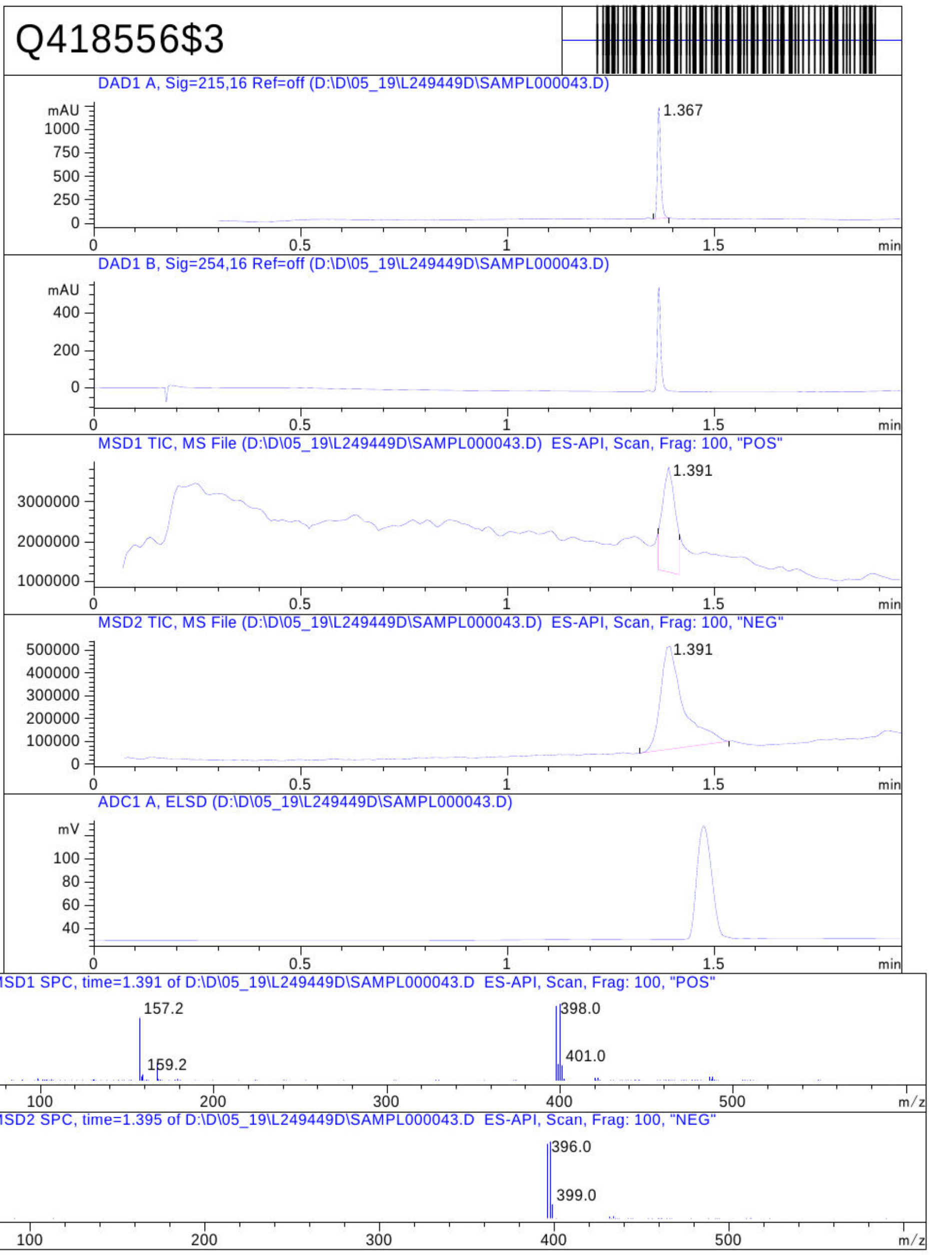

RT 1.391

RT 1.391

Inj.Date 5/19/2020 N -3- Acq. Method C:\CHEM32\-> ->

Focused library NMR and LCMS spectra

^{1}H NMR of **Compound 24** (500 MHz, CD_3OD)

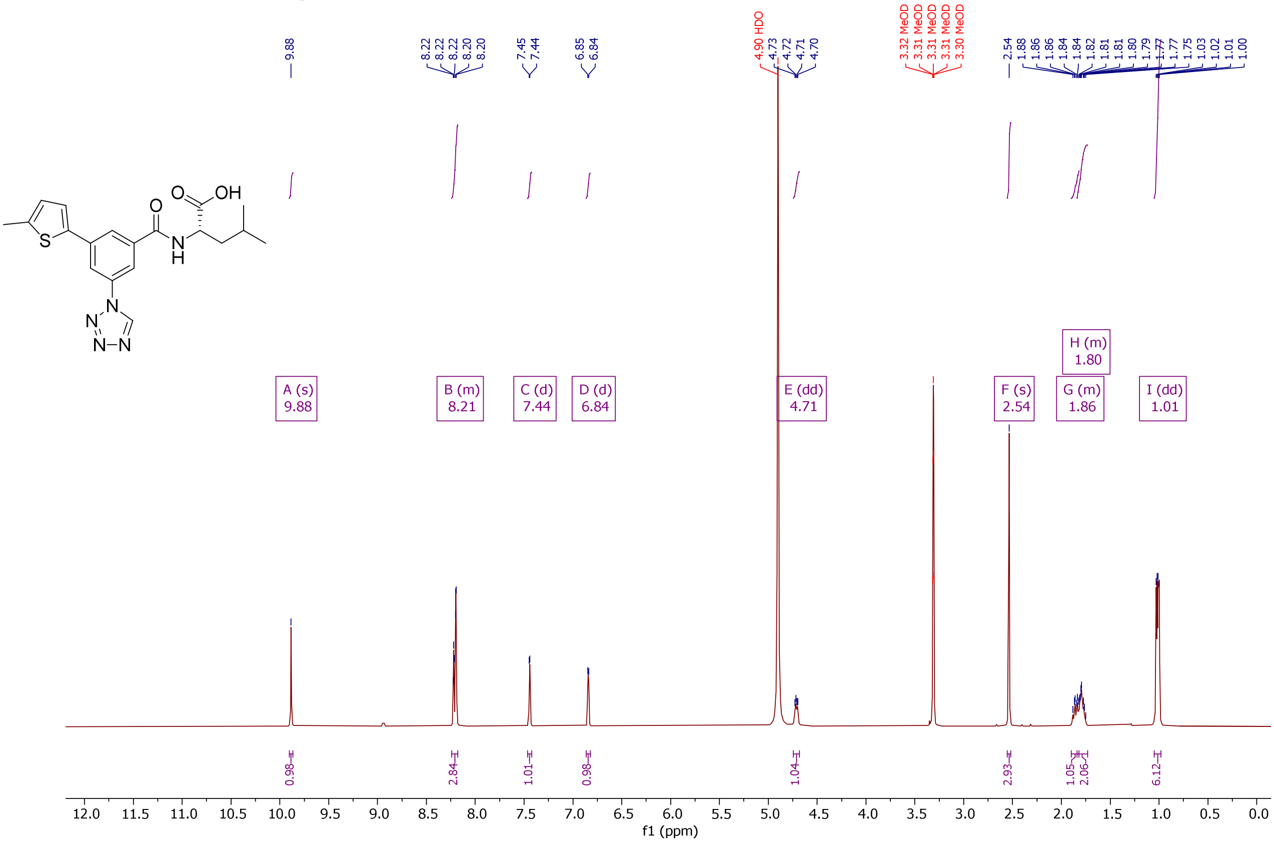

^{13}C NMR of **Compound 24** (125 MHz, CD_3OD)

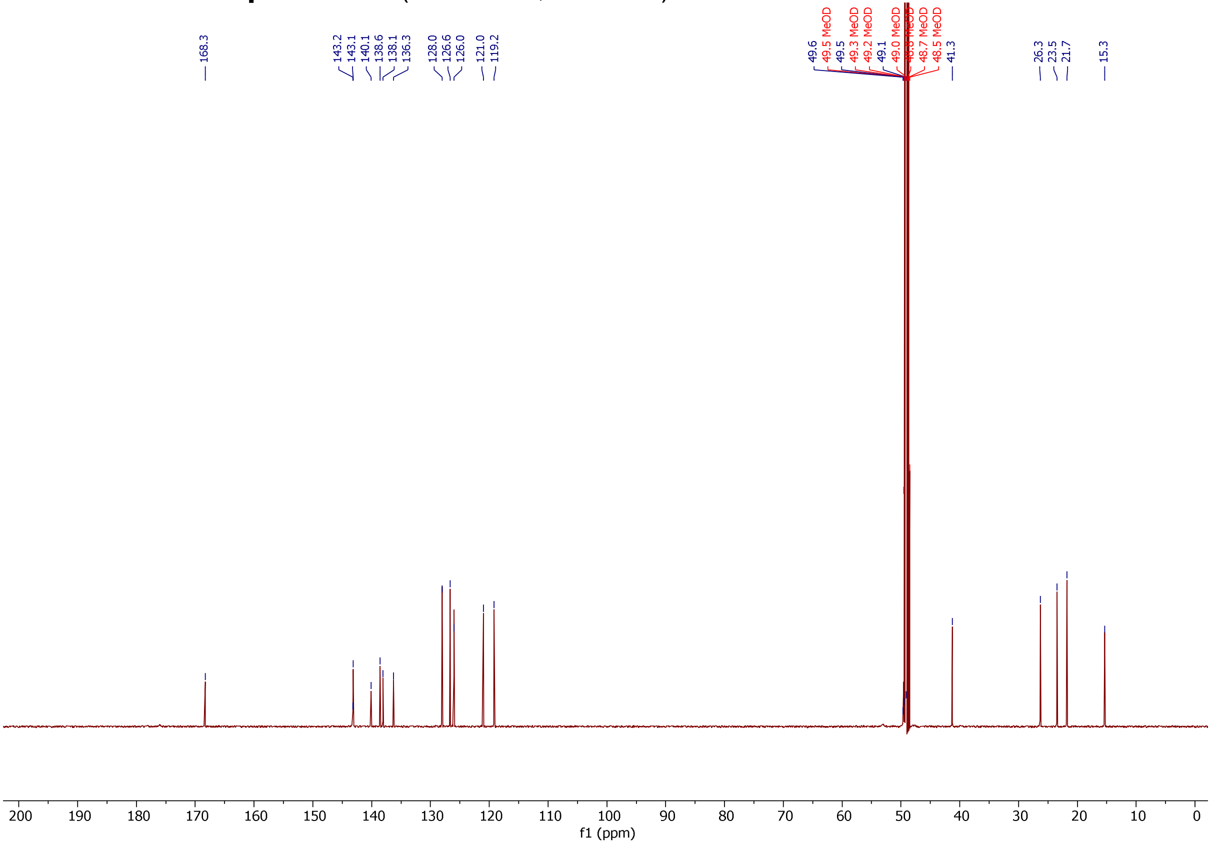

^{1}H NMR of **Compound 25** (600 MHz, CD_3OD)

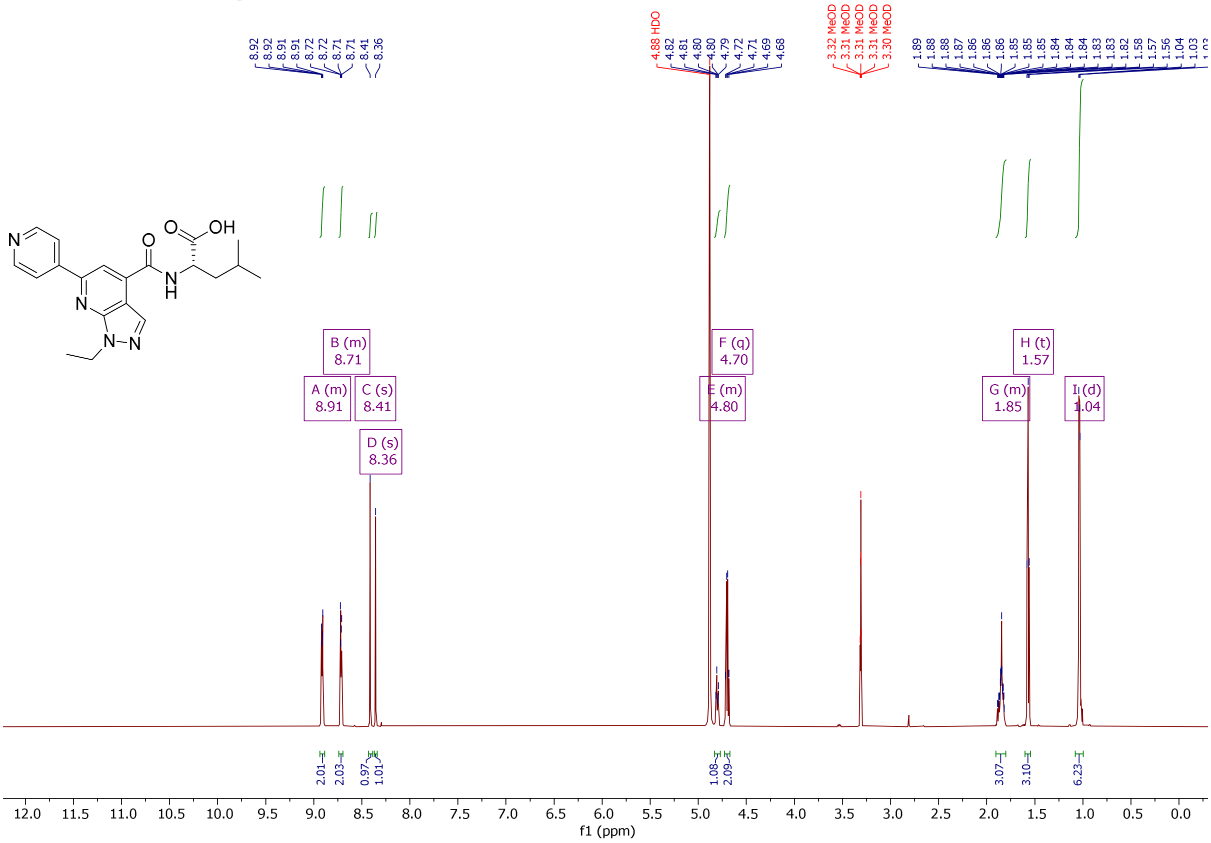

^{13}C NMR of **Compound 25** (150 MHz, CD_3OD)

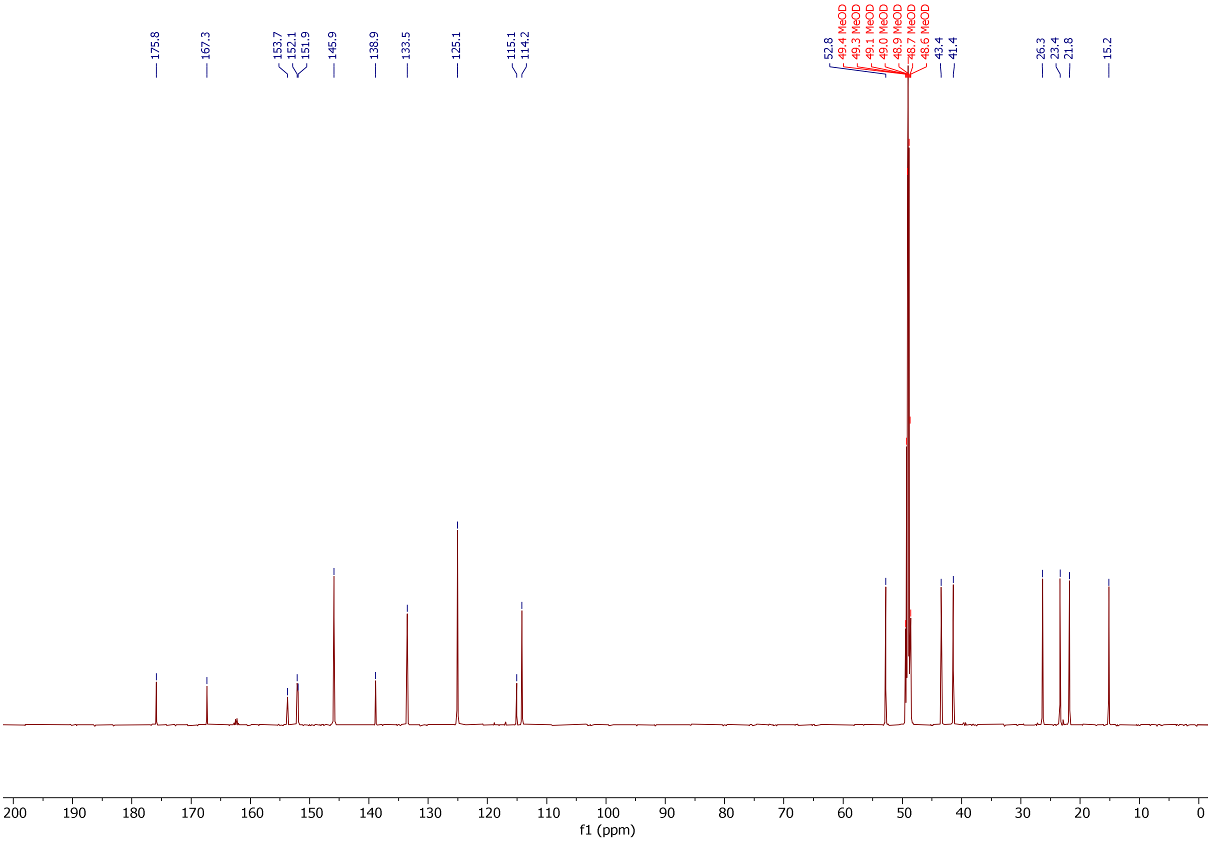

^{1}H NMR of **Compound 26** (600 MHz, CD_3OD)

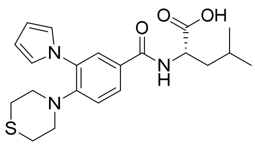

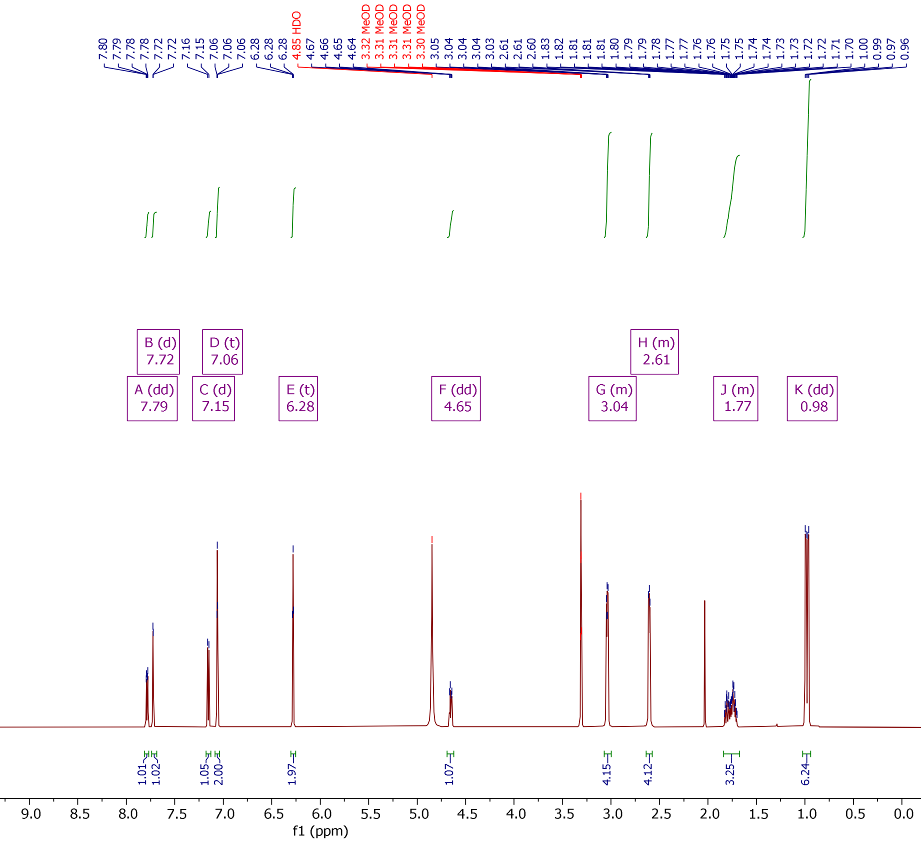

^{13}C NMR of **Compound 26** (150 MHz, CD_3OD)

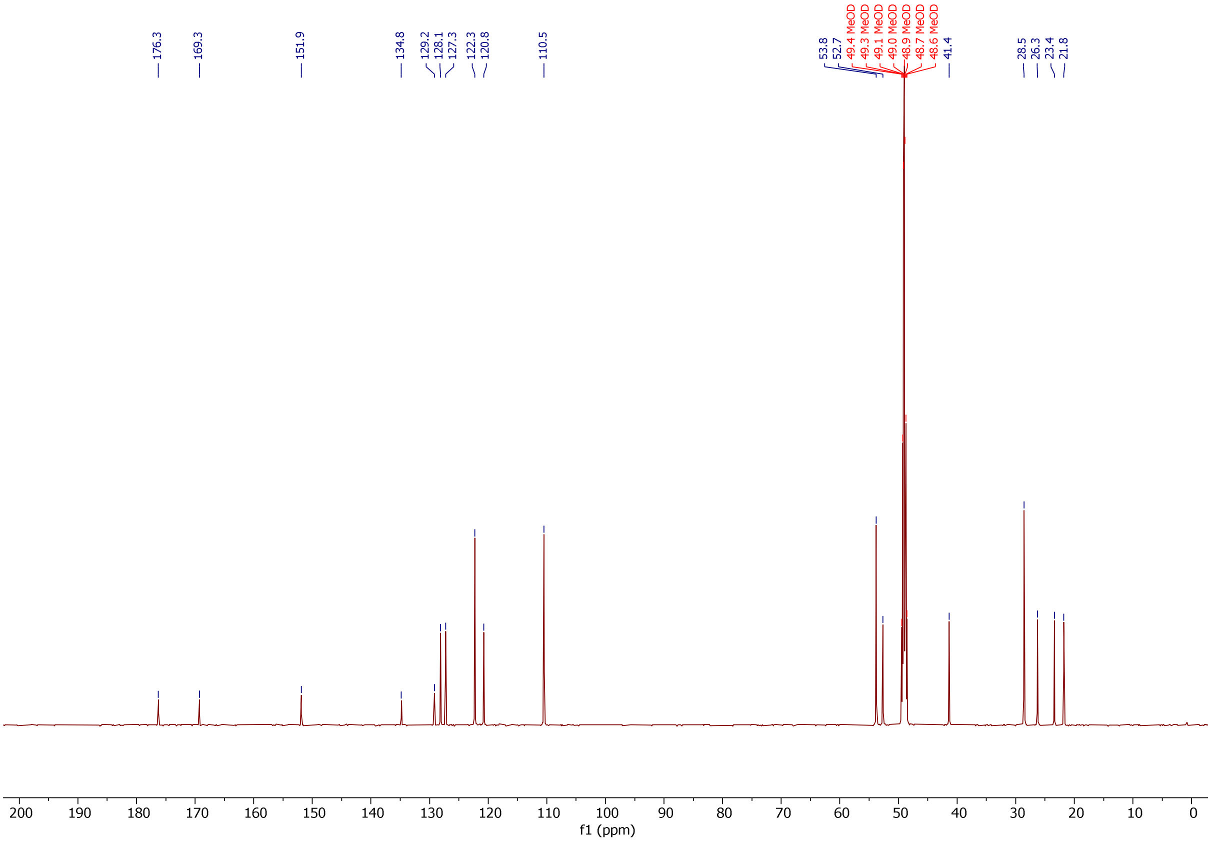

^{1}H NMR of **Compound 27** (600 MHz, CD_3OD)

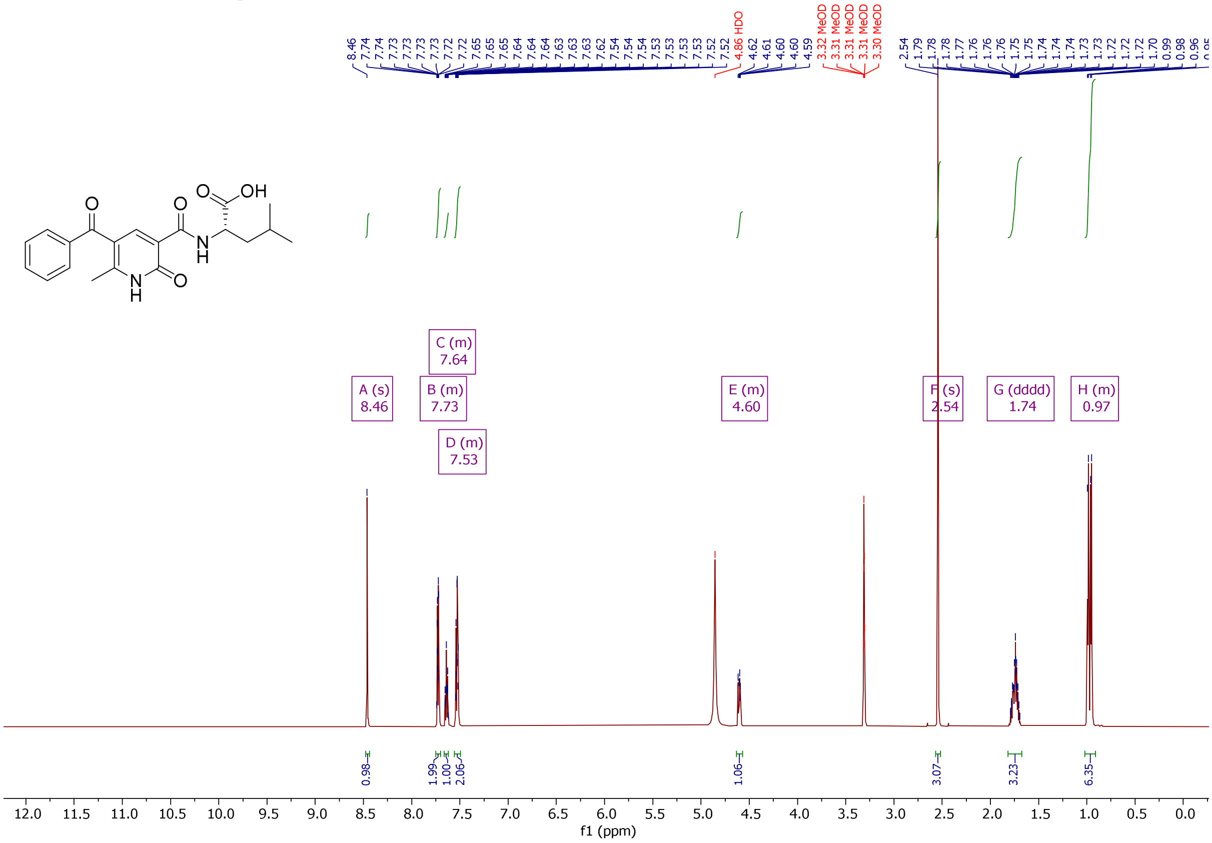

^{13}C NMR of **Compound 27** (150 MHz, CD_3OD)

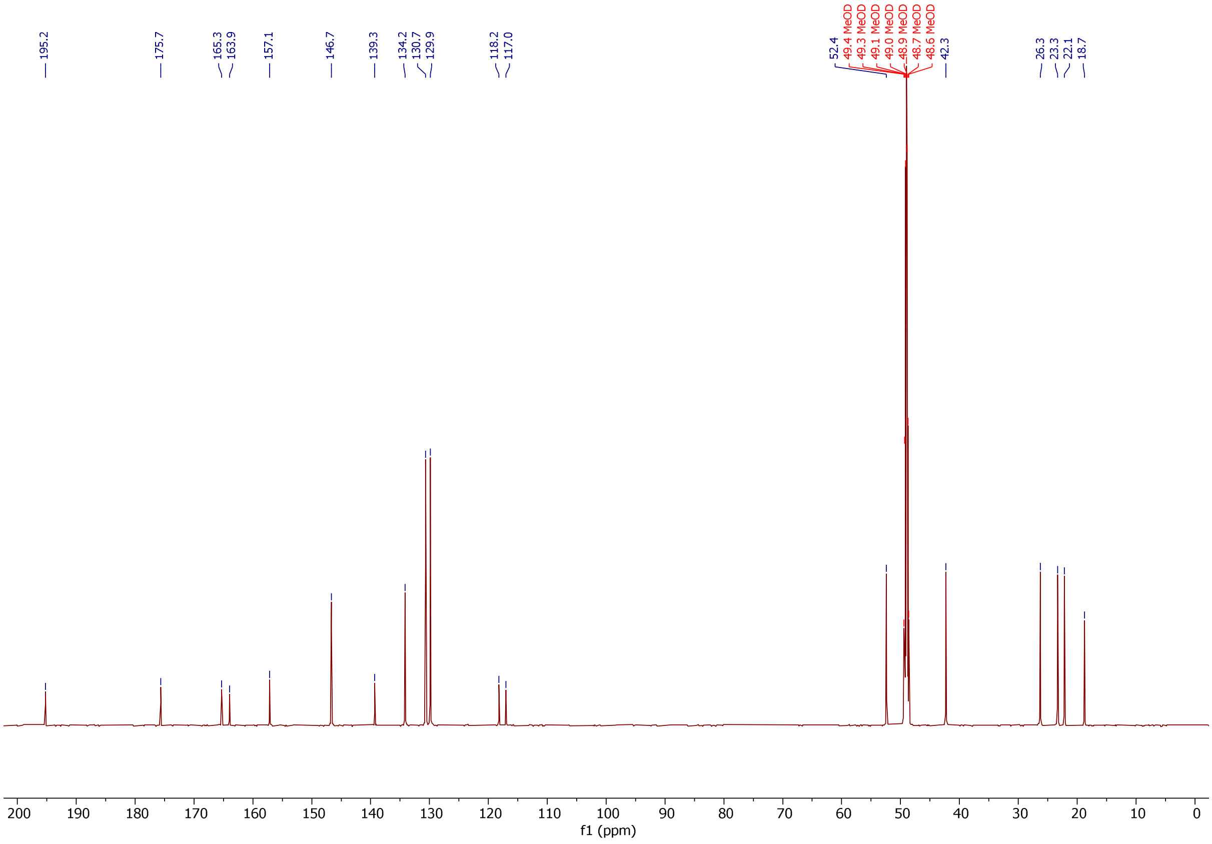

^{1}H NMR of **Compound 28** (600 MHz, CD_3OD)

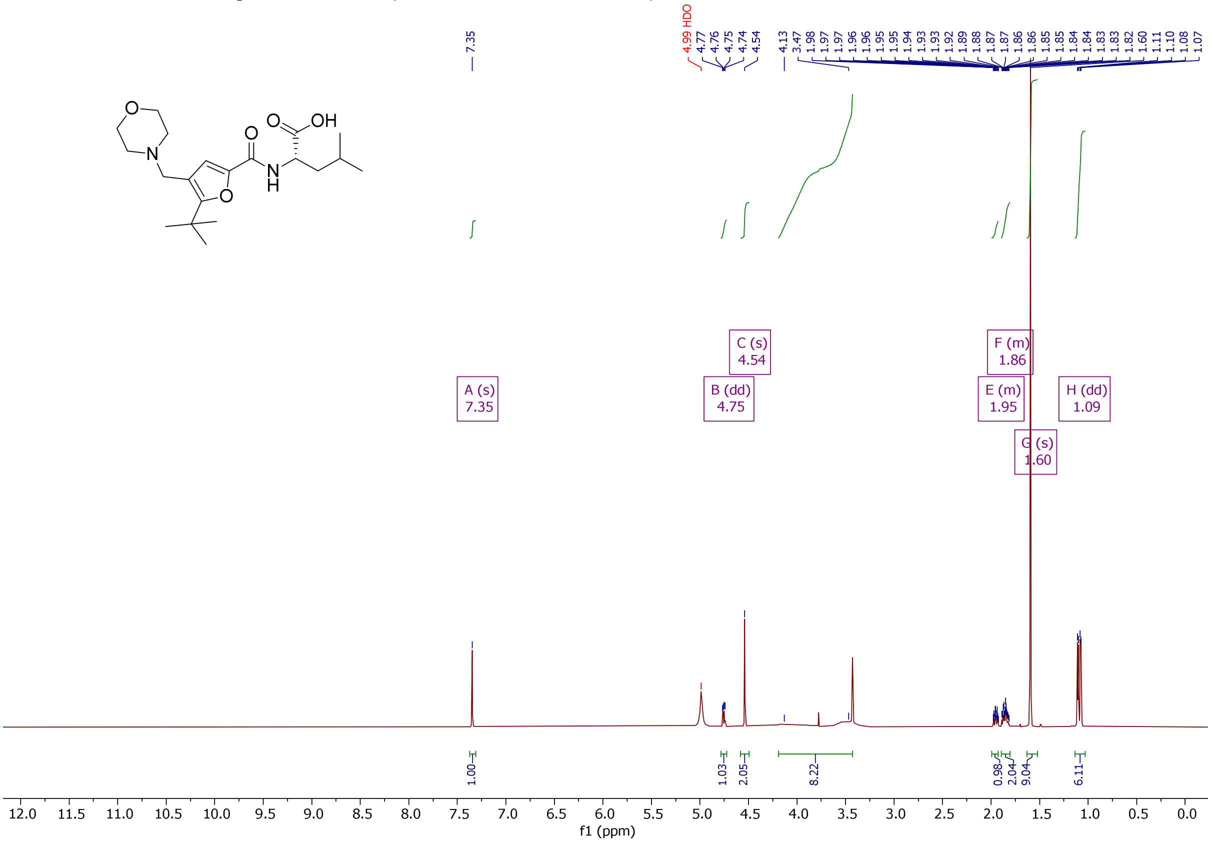

^{13}C NMR of **Compound 28** (150 MHz, CD_3OD)

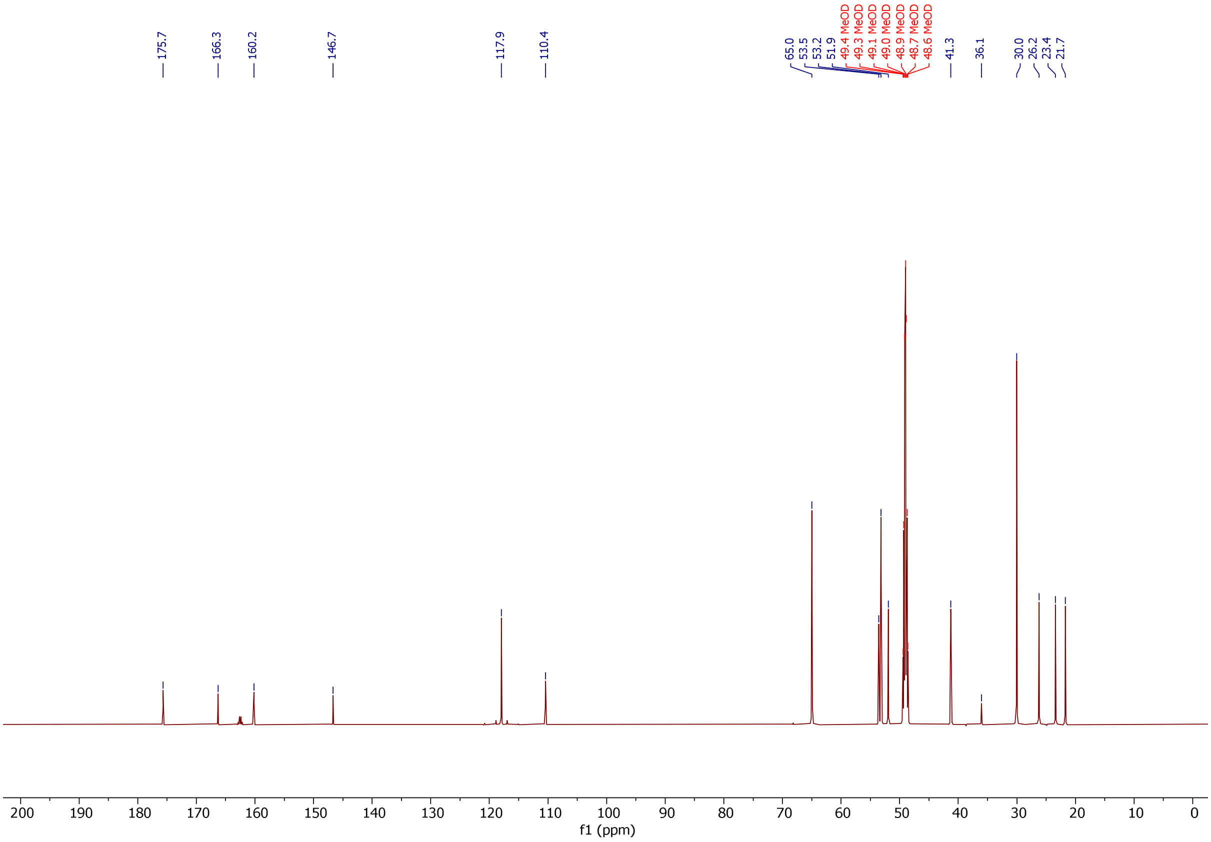

^{1}H NMR of **Compound 29** (600 MHz, CD_3OD)

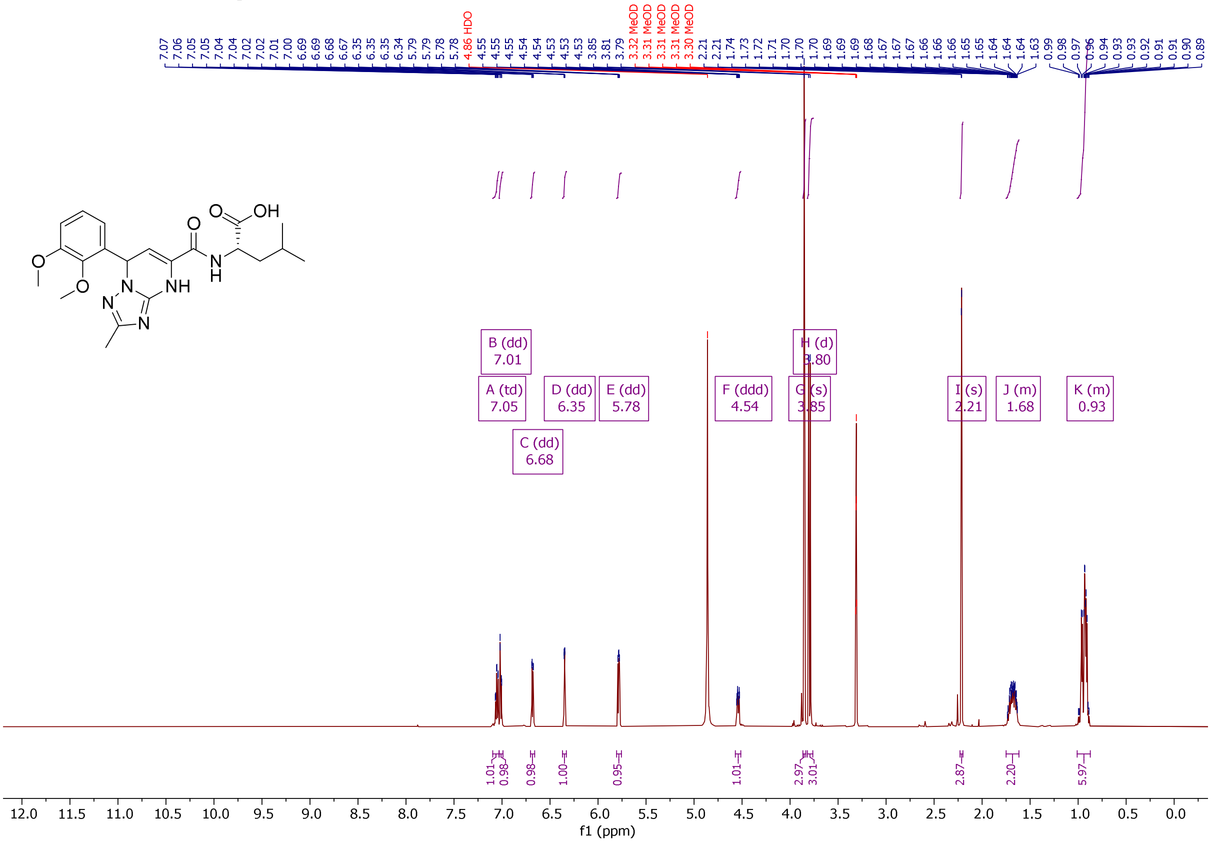

^{13}C NMR of **Compound 29** (150 MHz, CD_3OD)

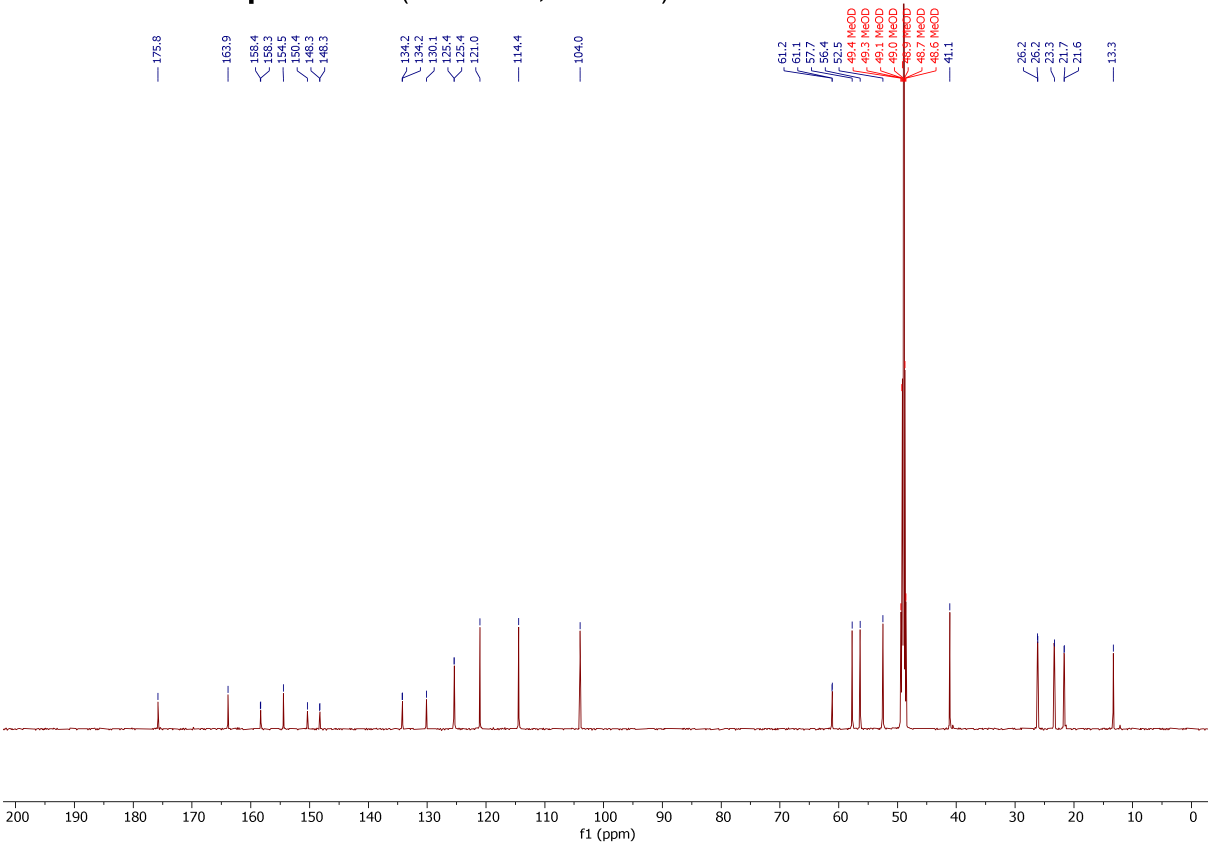

^{1}H NMR of **Compound 30F1** (600 MHz, CD_3OD)

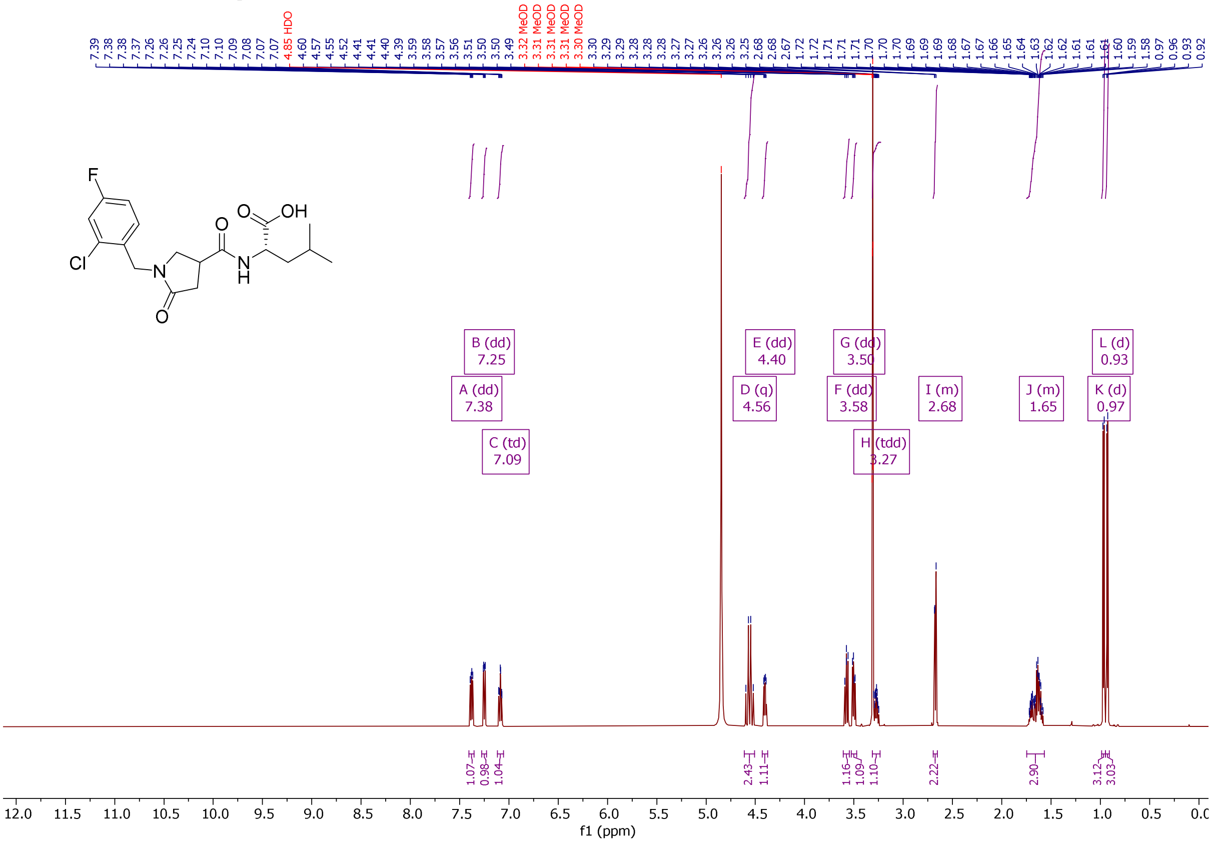

^{13}C NMR of **Compound 30F1** (150 MHz, CD_3OD)

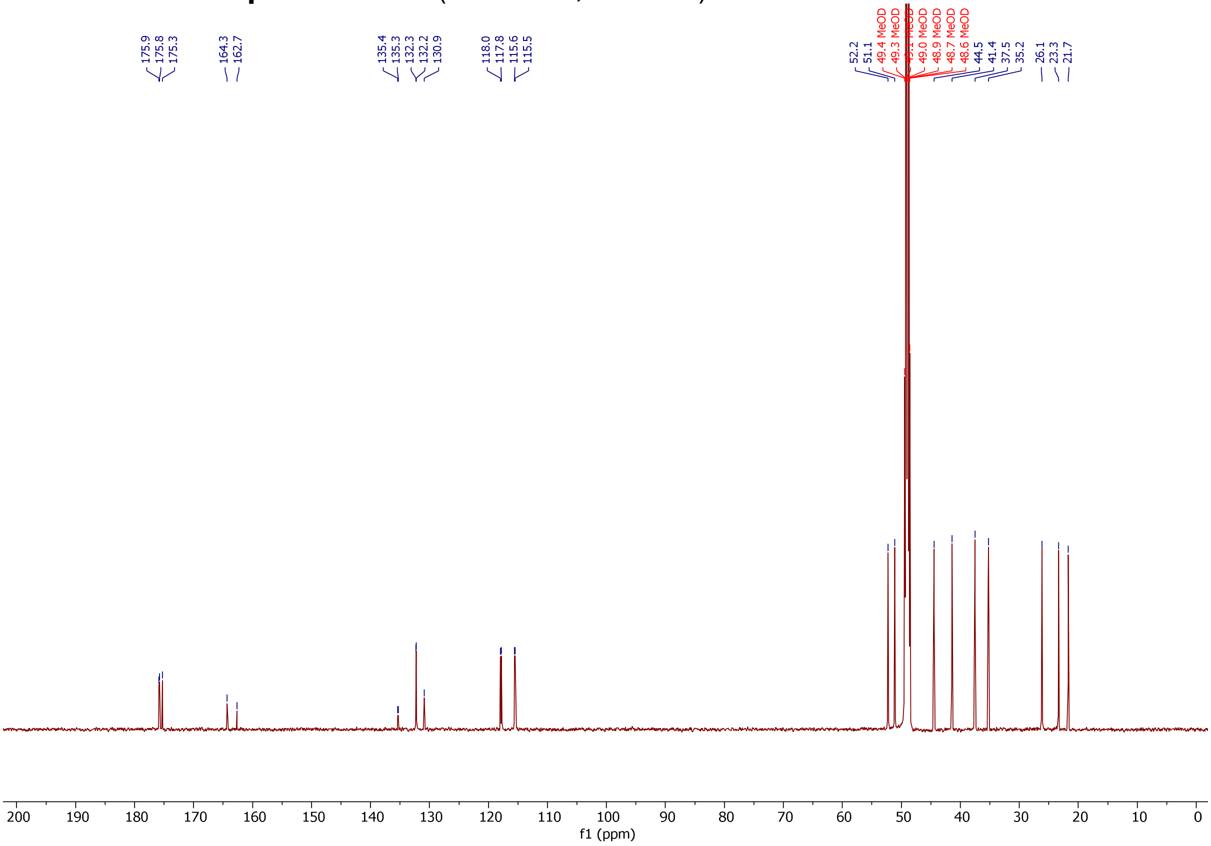

^{1}H NMR of **Compound 30F2** (600 MHz, CD_3OD)

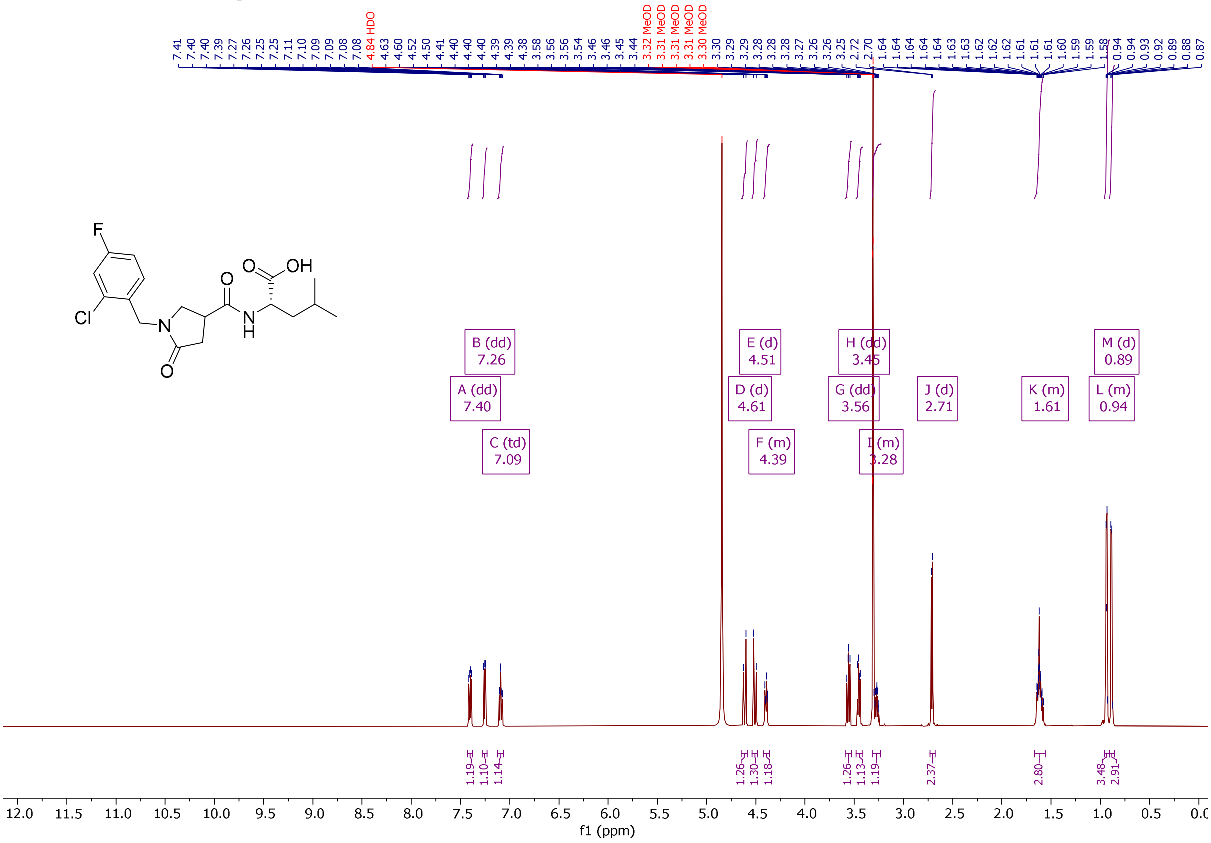

^{13}C NMR of **Compound 30F2** (150 MHz, CD_3OD)

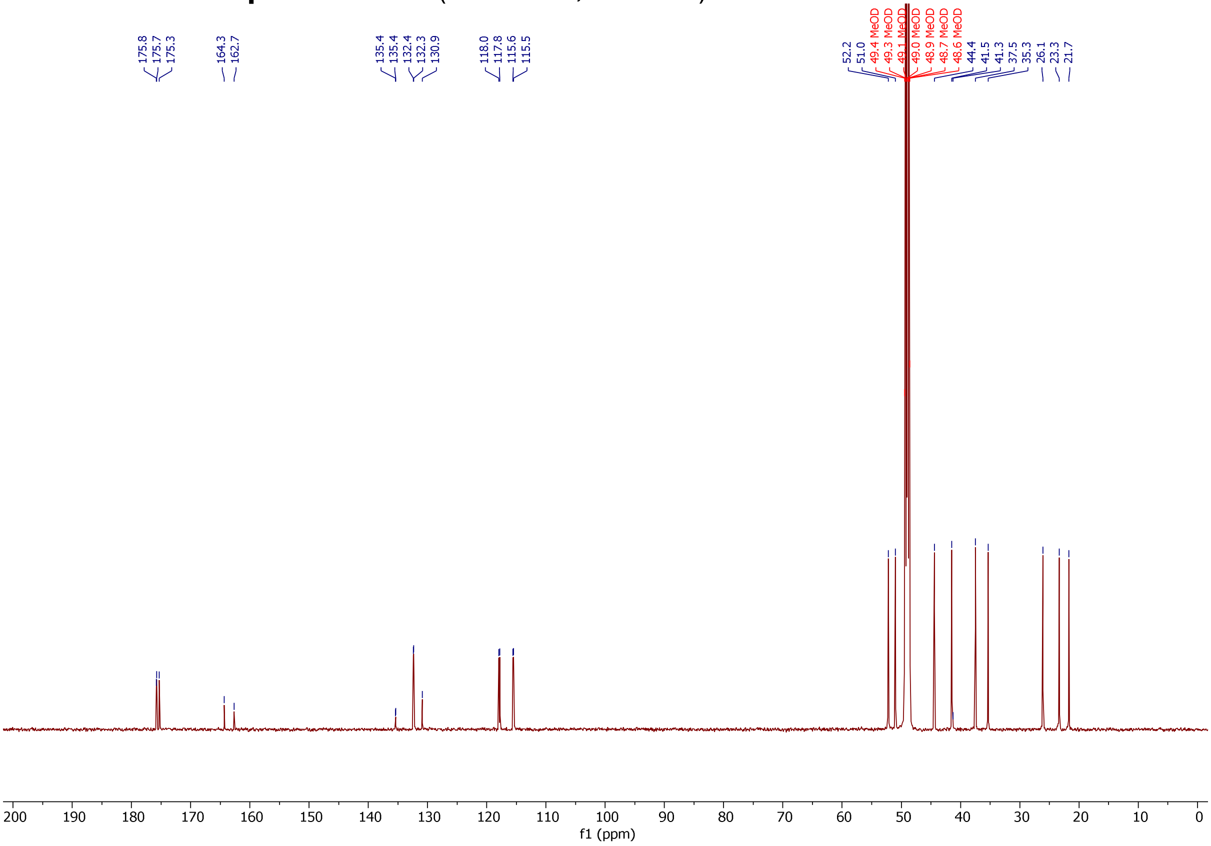

^{1}H NMR of **Compound 31** (600 MHz, CD_3OD)

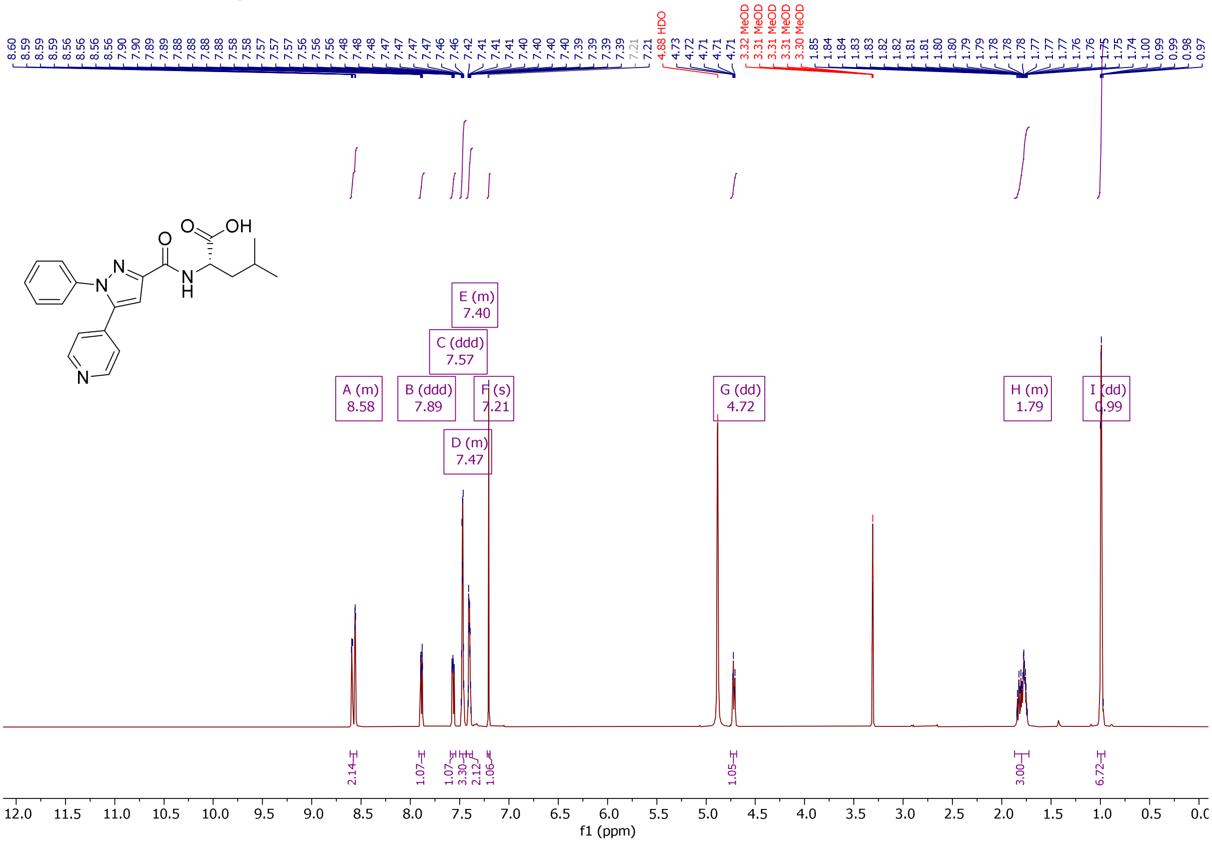

^{13}C NMR of **Compound 31** (150 MHz, CD_3OD)

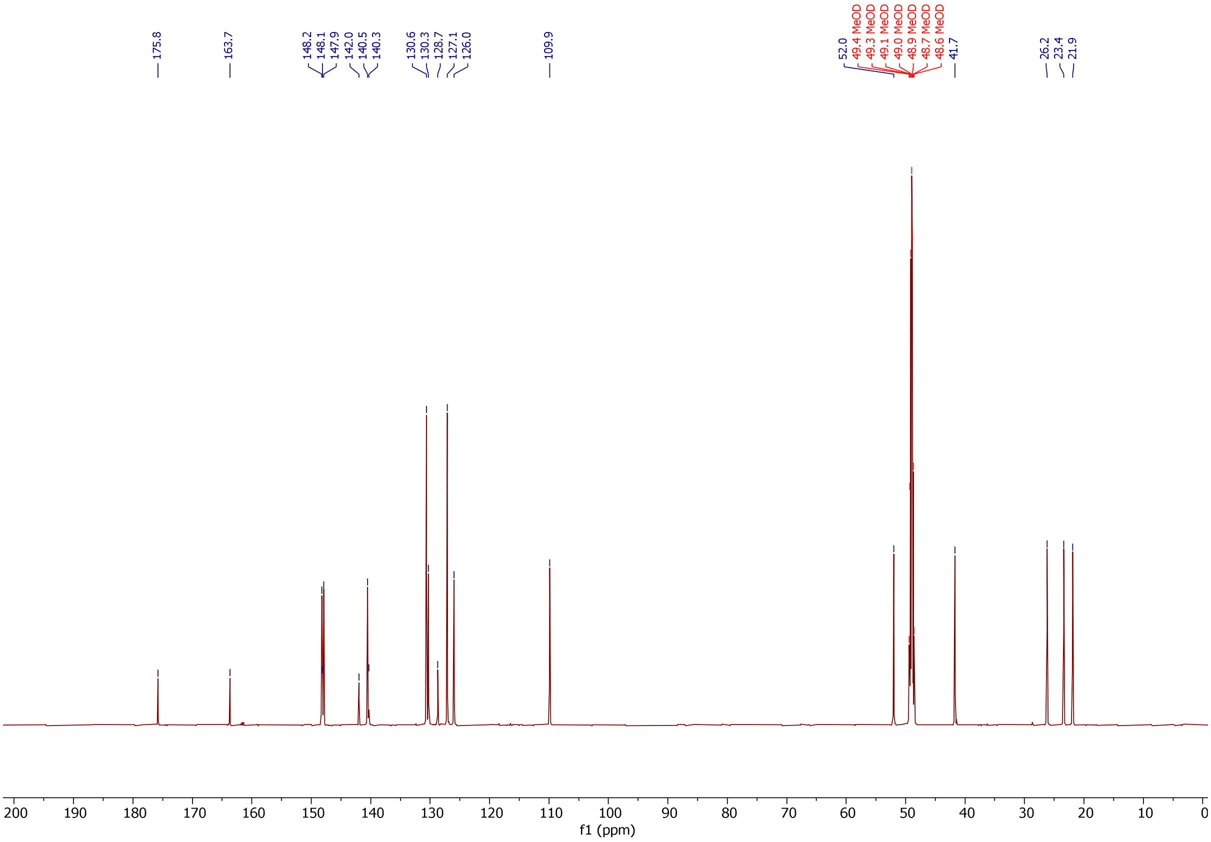

^{1}H NMR of **Compound 32** (600 MHz, CD_3OD)

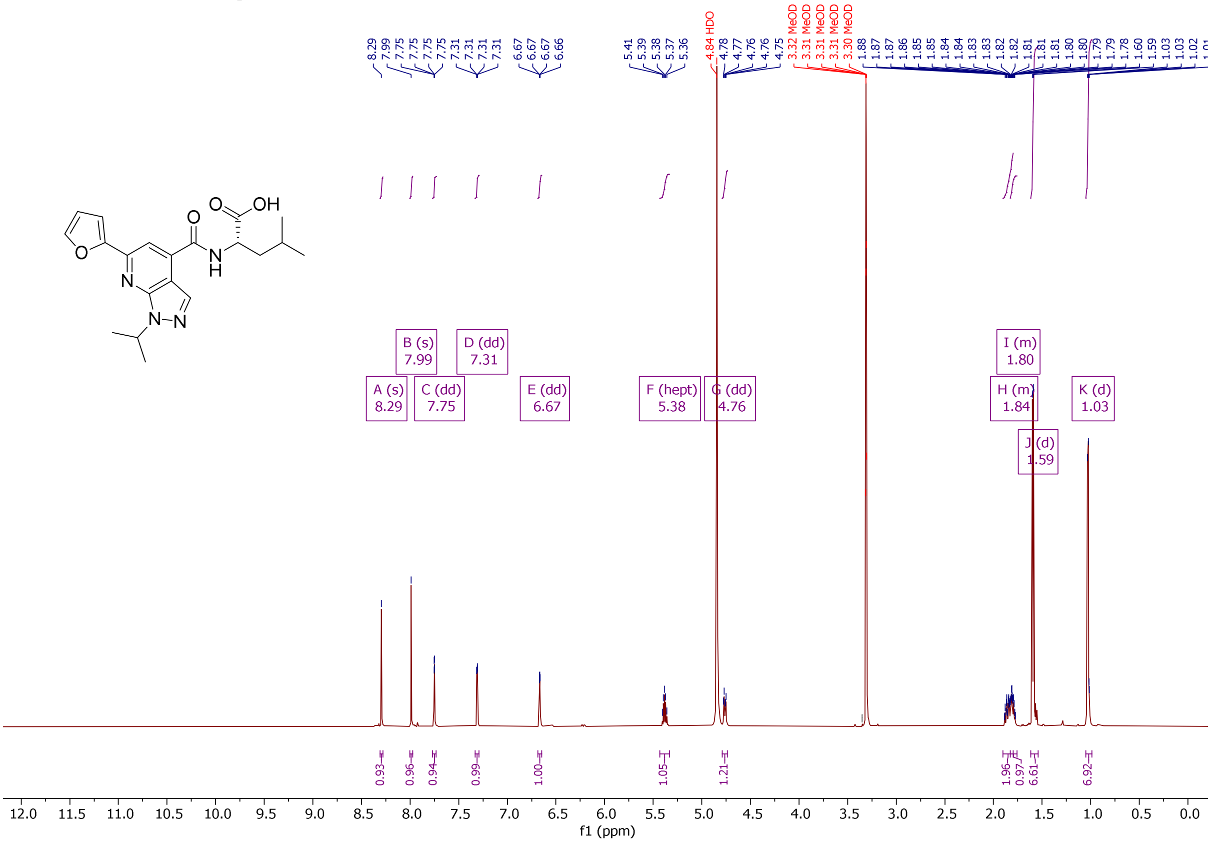

^{13}C NMR of **Compound 32** (150 MHz, CD_3OD)

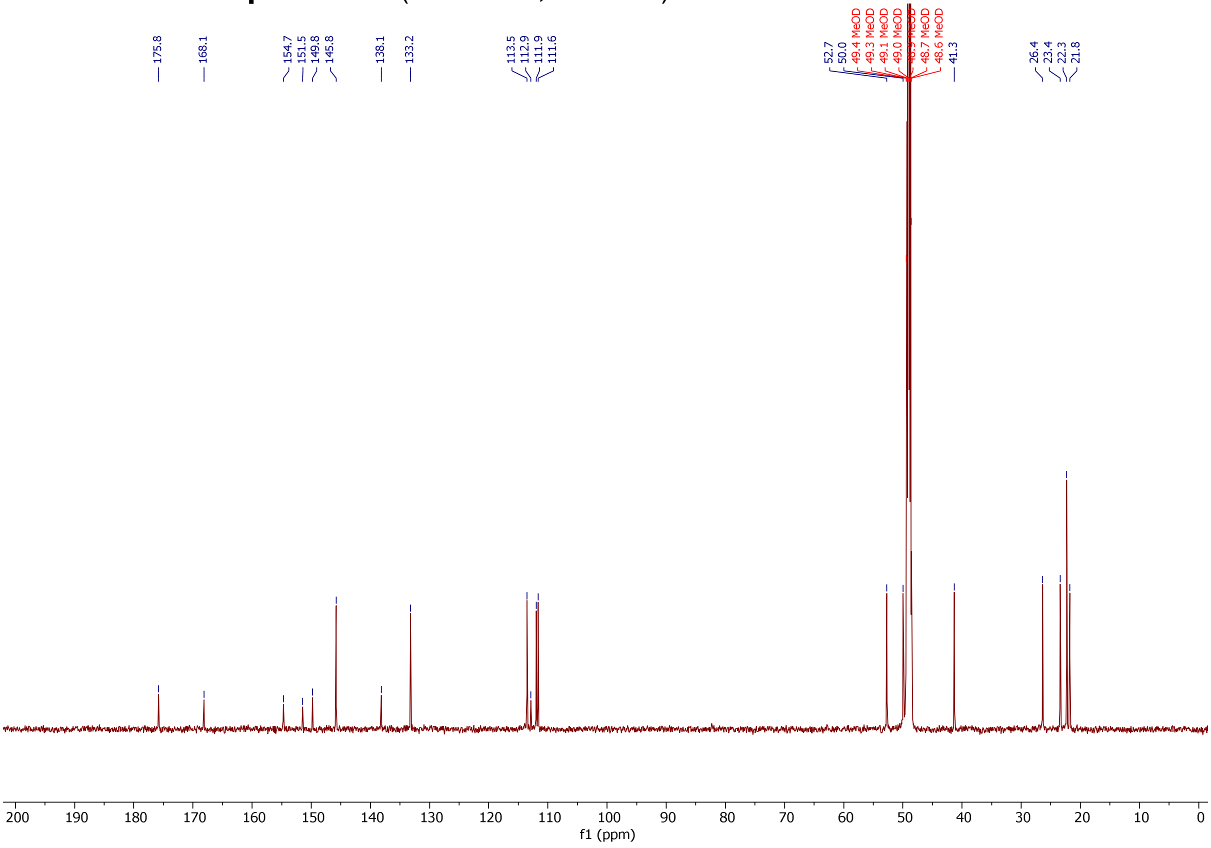

^{1}H NMR of **Compound 33** (600 MHz, CD_3OD)

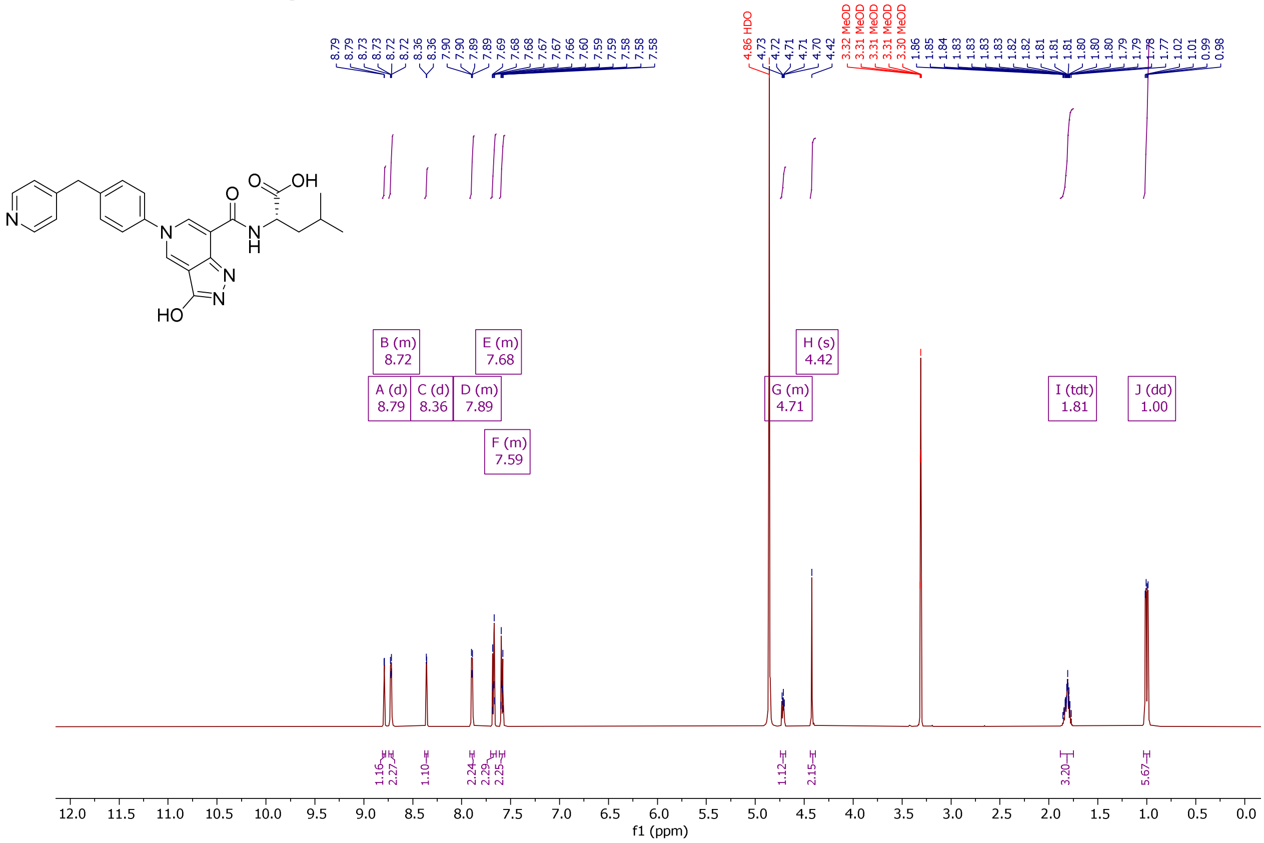

^{13}C NMR of **Compound 33** (150 MHz, CD_3OD)

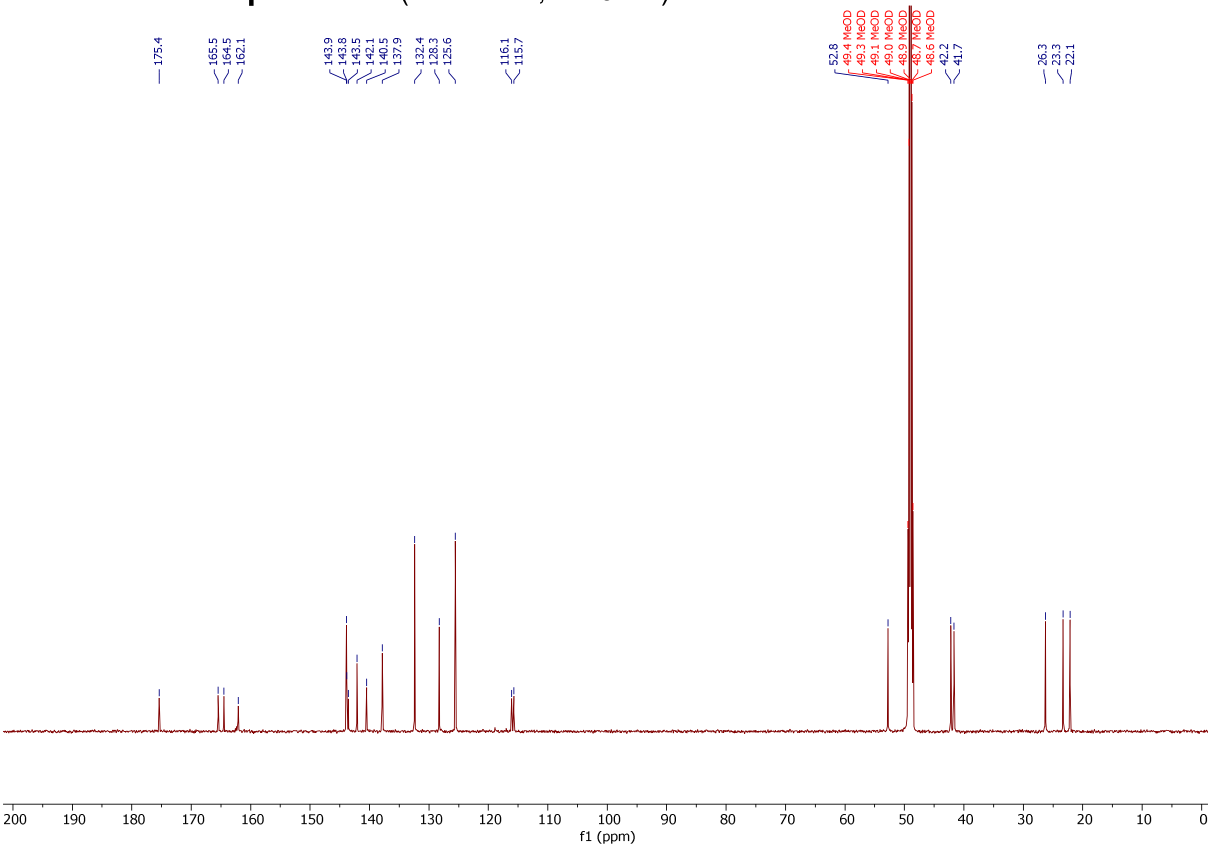

^{1}H NMR of **Compound 34** (600 MHz, CD_3OD)

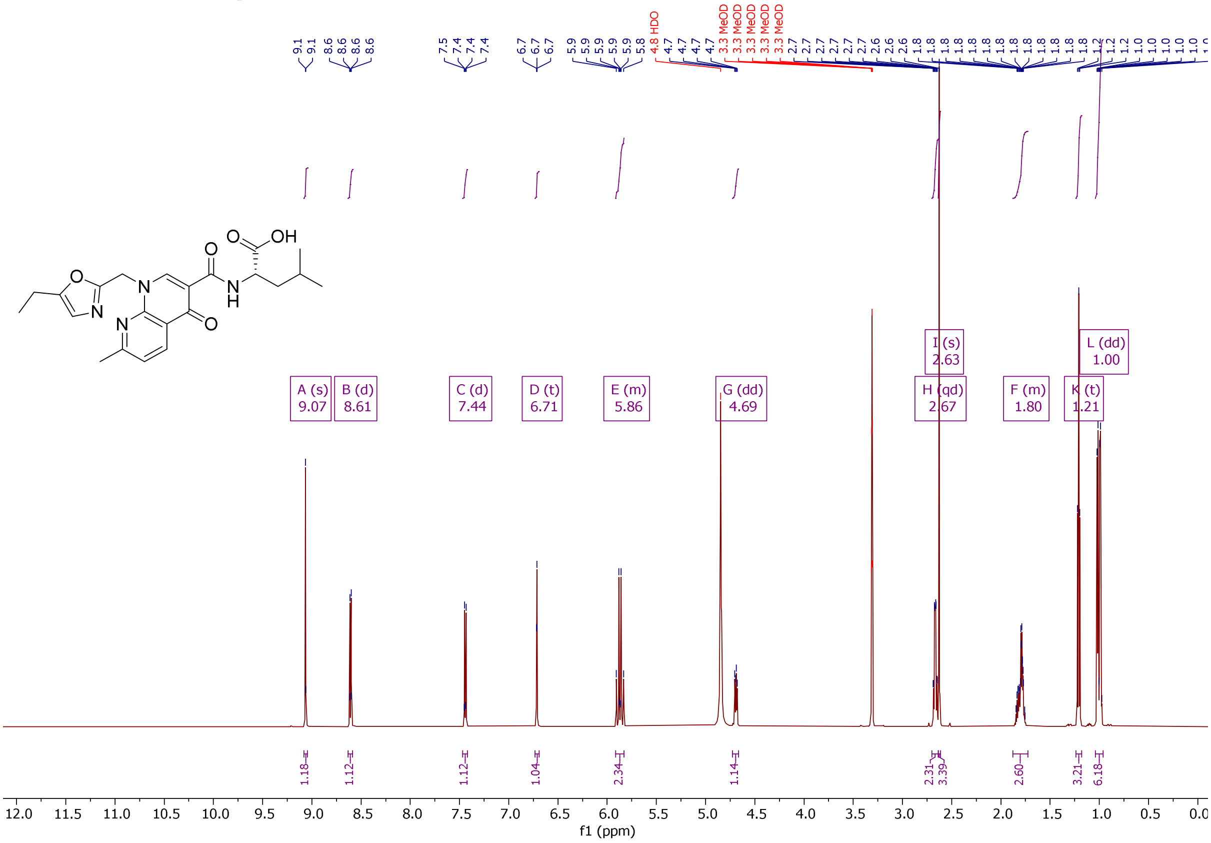

^{13}C NMR of **Compound 34** (150 MHz, CD_3OD)

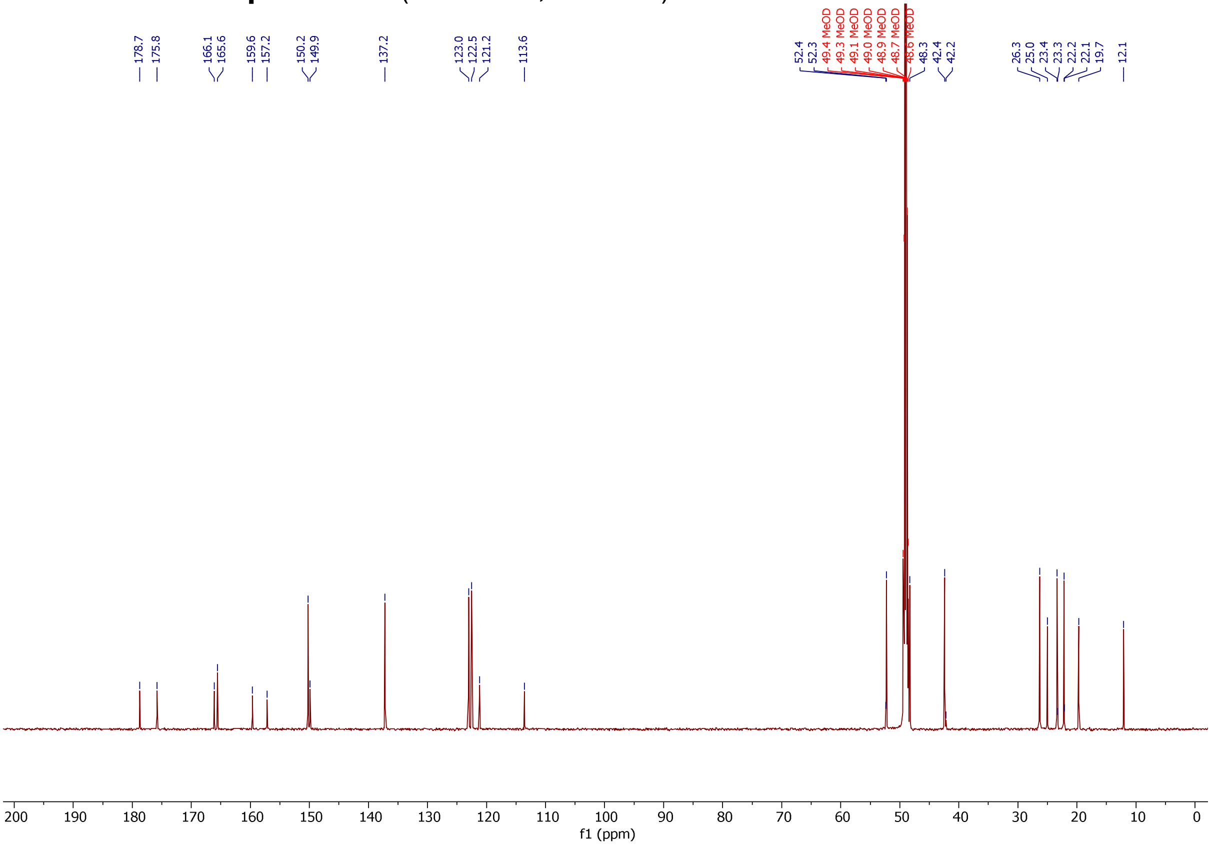

^{1}H NMR of **Compound 35** (600 MHz, CD_3OD)

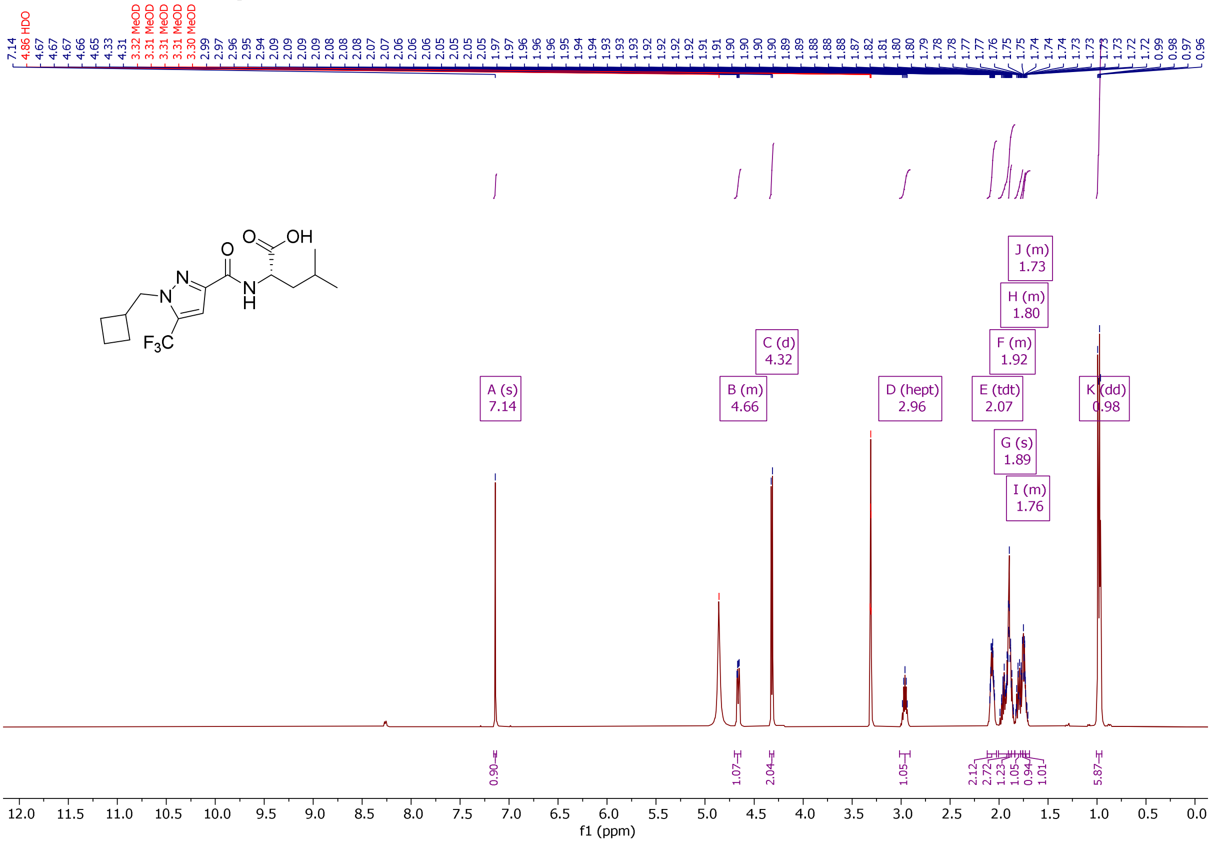

^{13}C NMR of **Compound 35** (150 MHz, CD_3OD)

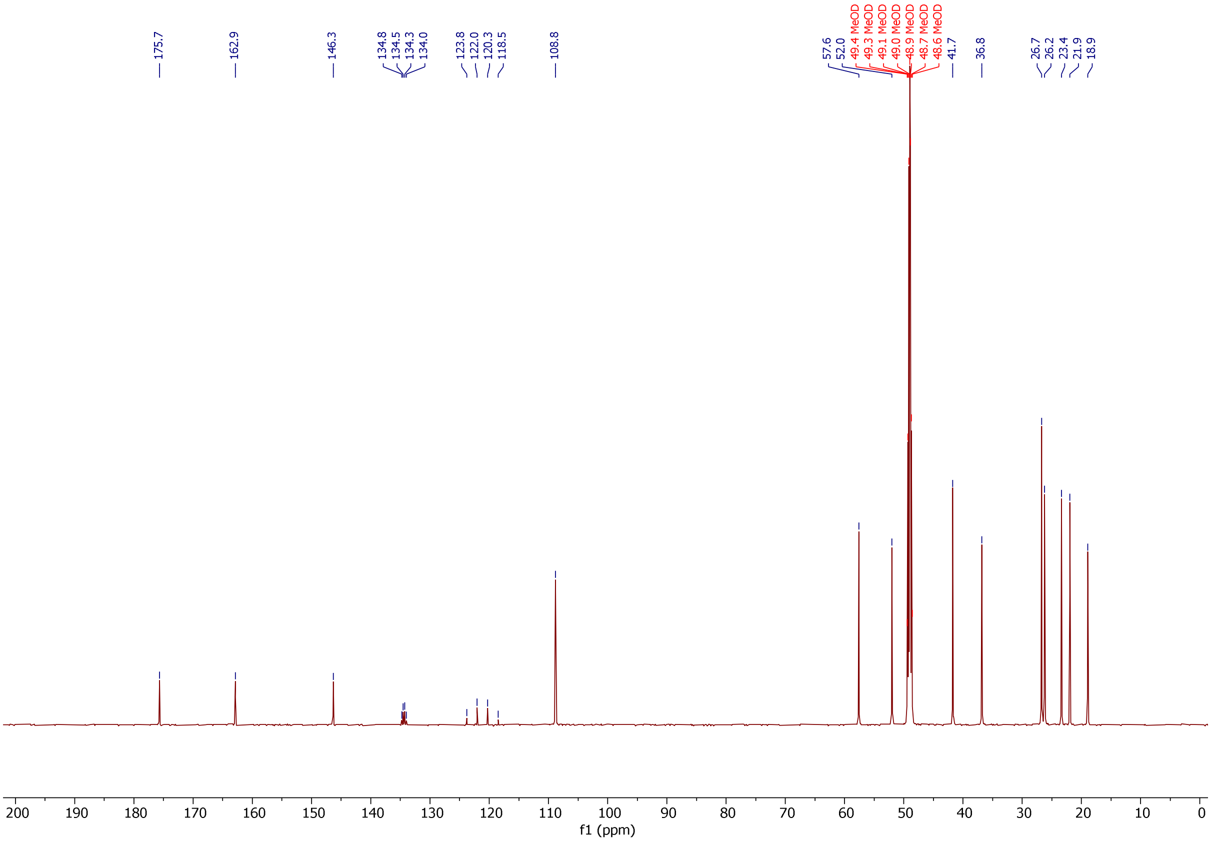

^{1}H NMR of **Compound 36** (600 MHz, CD_3OD)

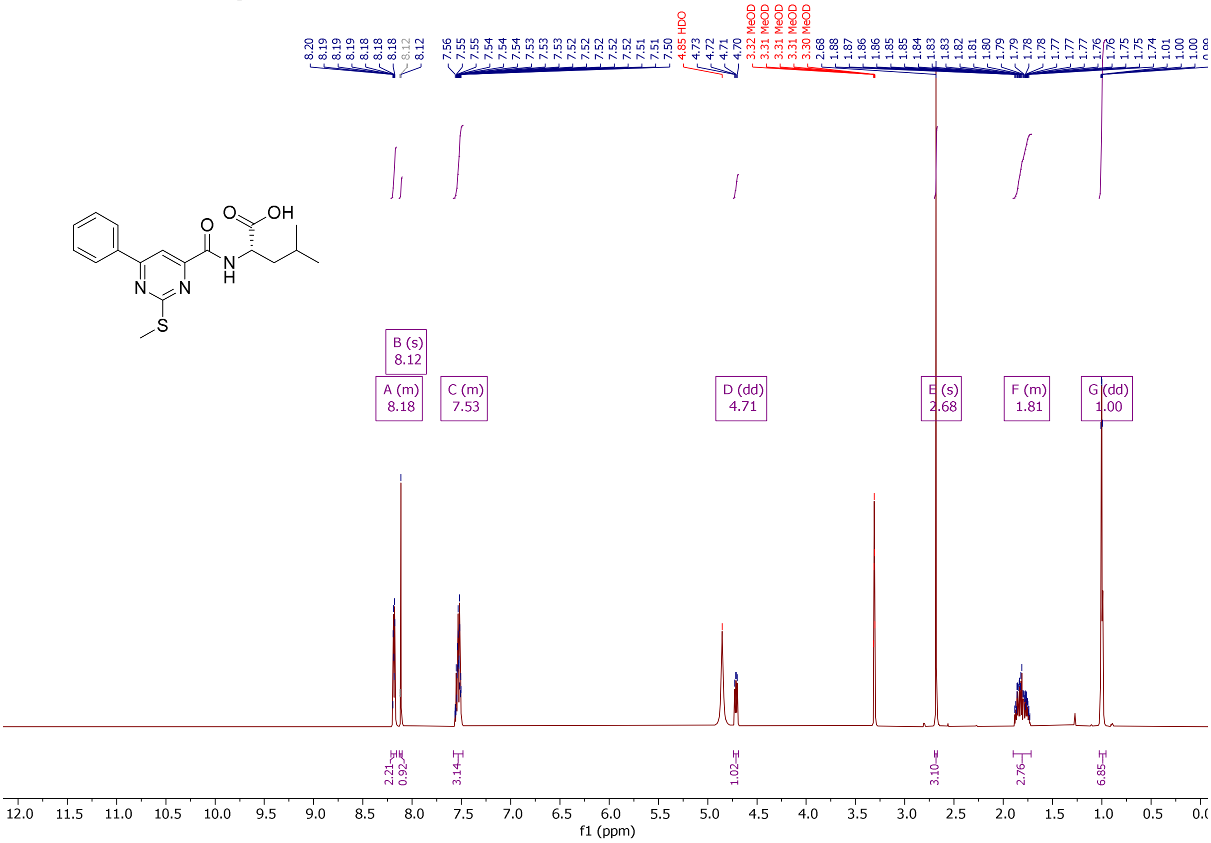

^{13}C NMR of **Compound 36** (150 MHz, CD_3OD)

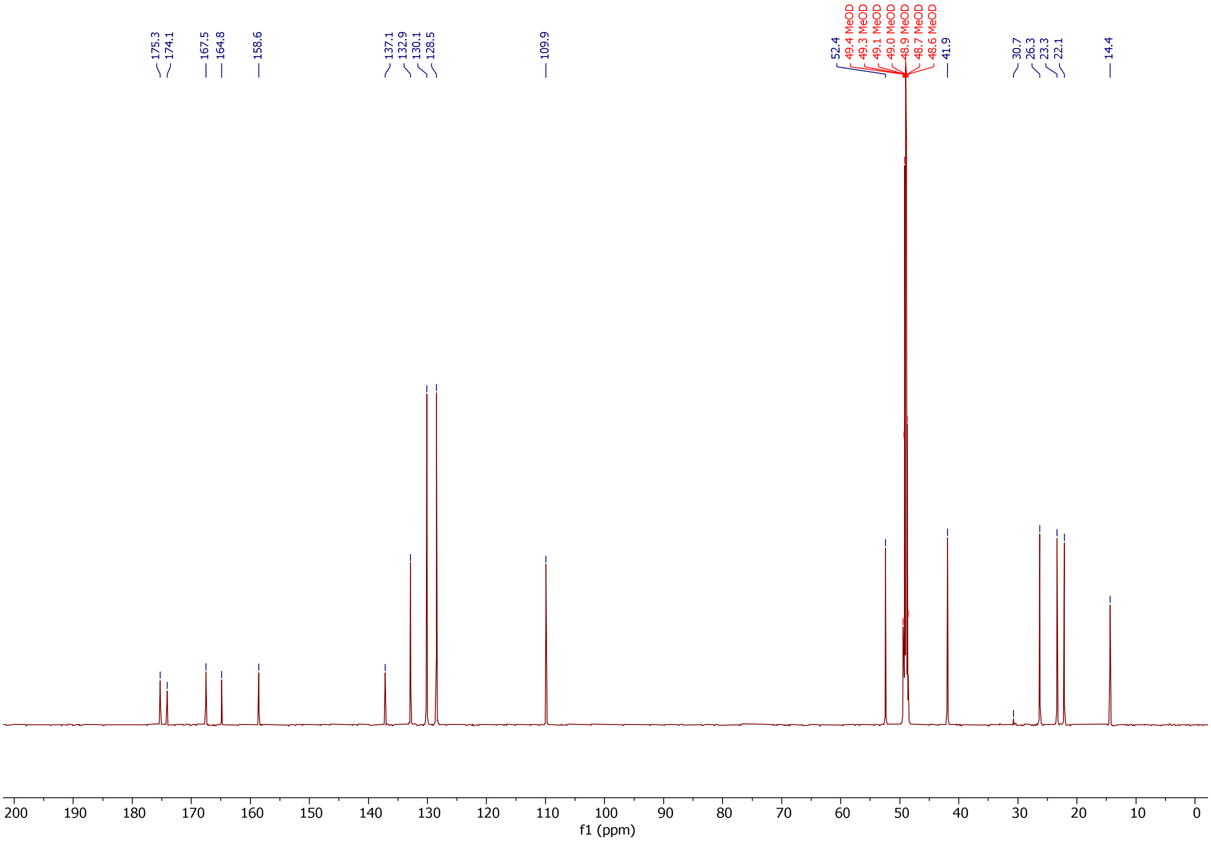

^{1}H NMR of **Compound 37** (600 MHz, CD_3OD)

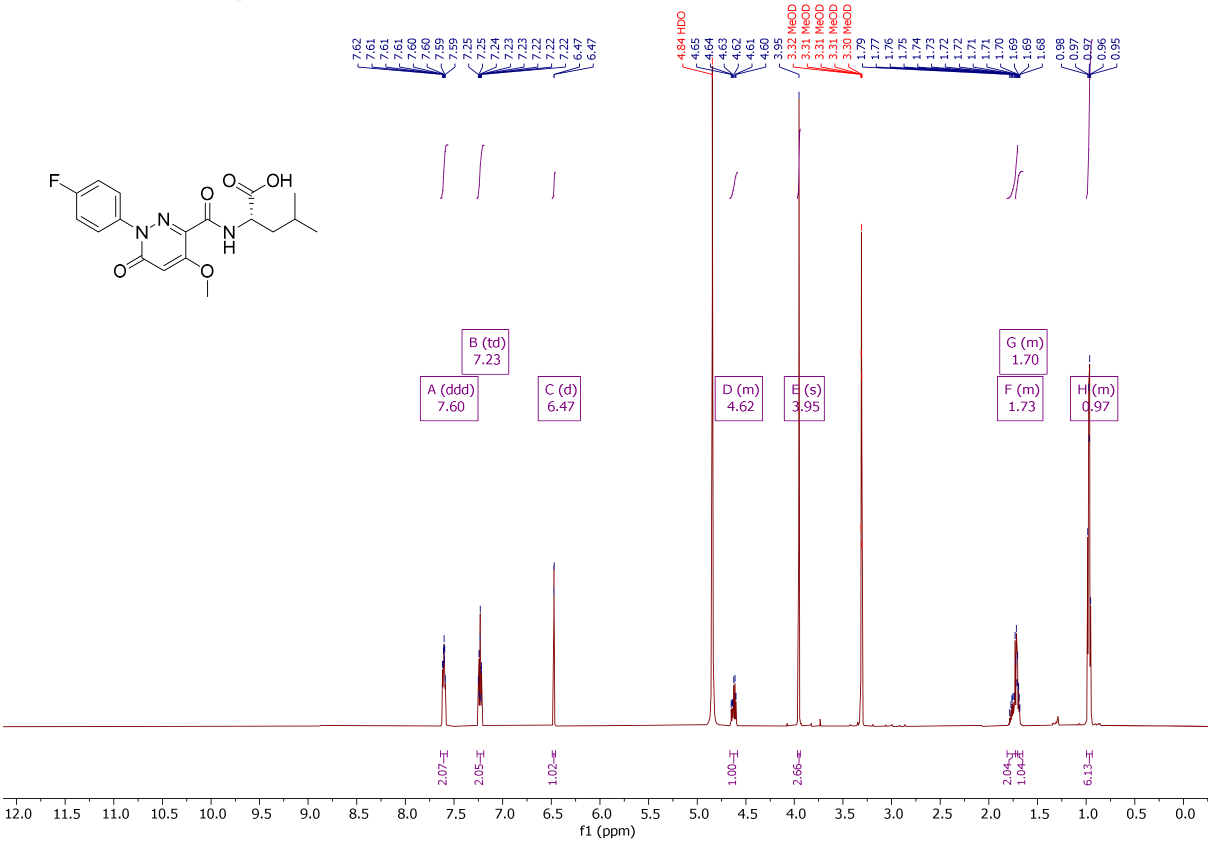

^{13}C NMR of **Compound 37** (150 MHz, CD_3OD)

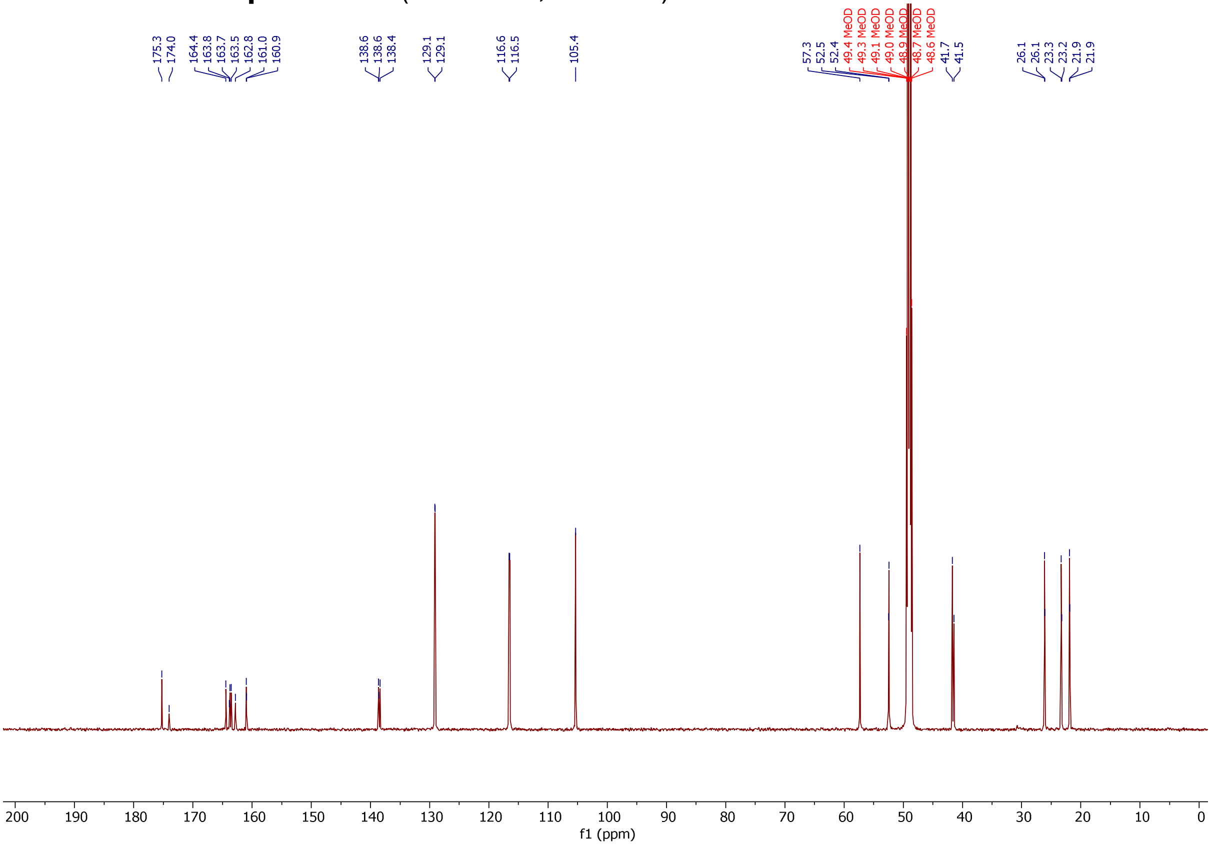

^{1}H NMR of **Compound 38** (600 MHz, CD_3OD)

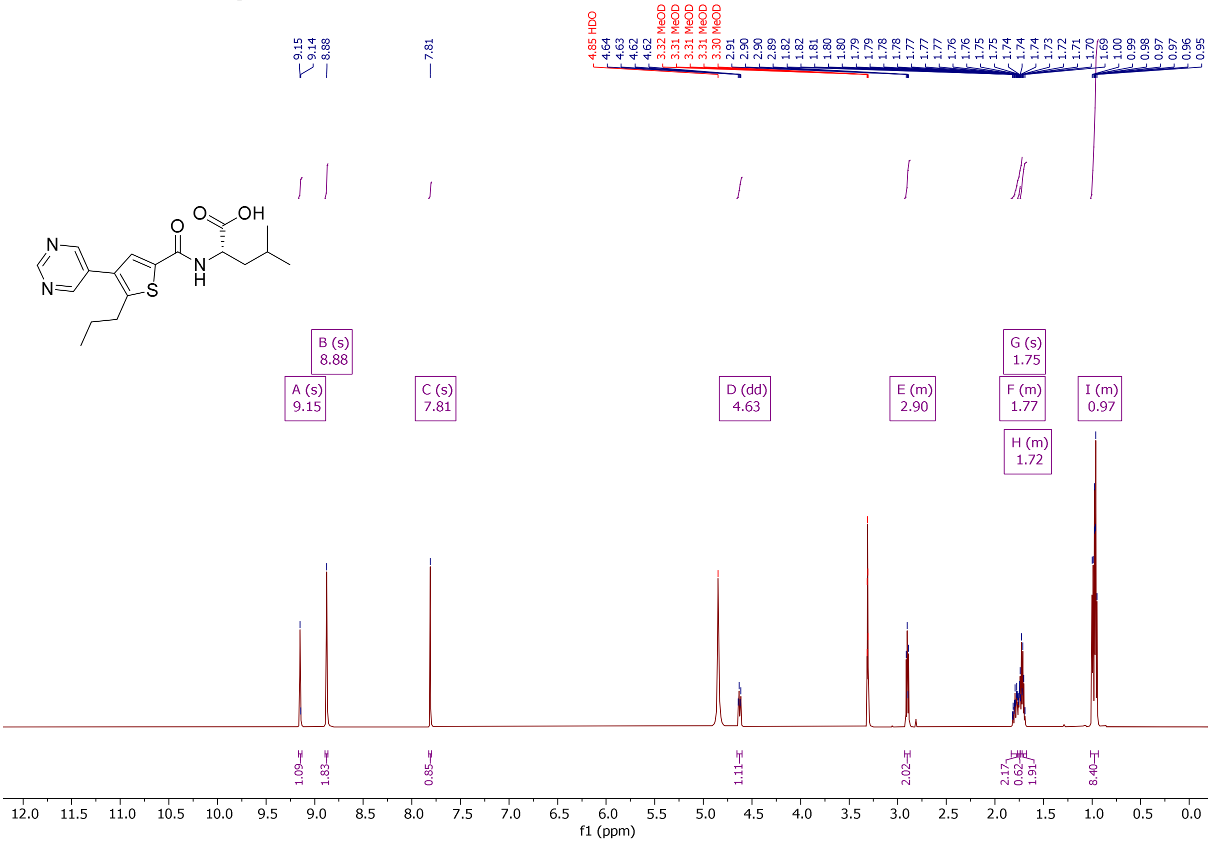

^{13}C NMR of **Compound 38** (150 MHz, CD_3OD)

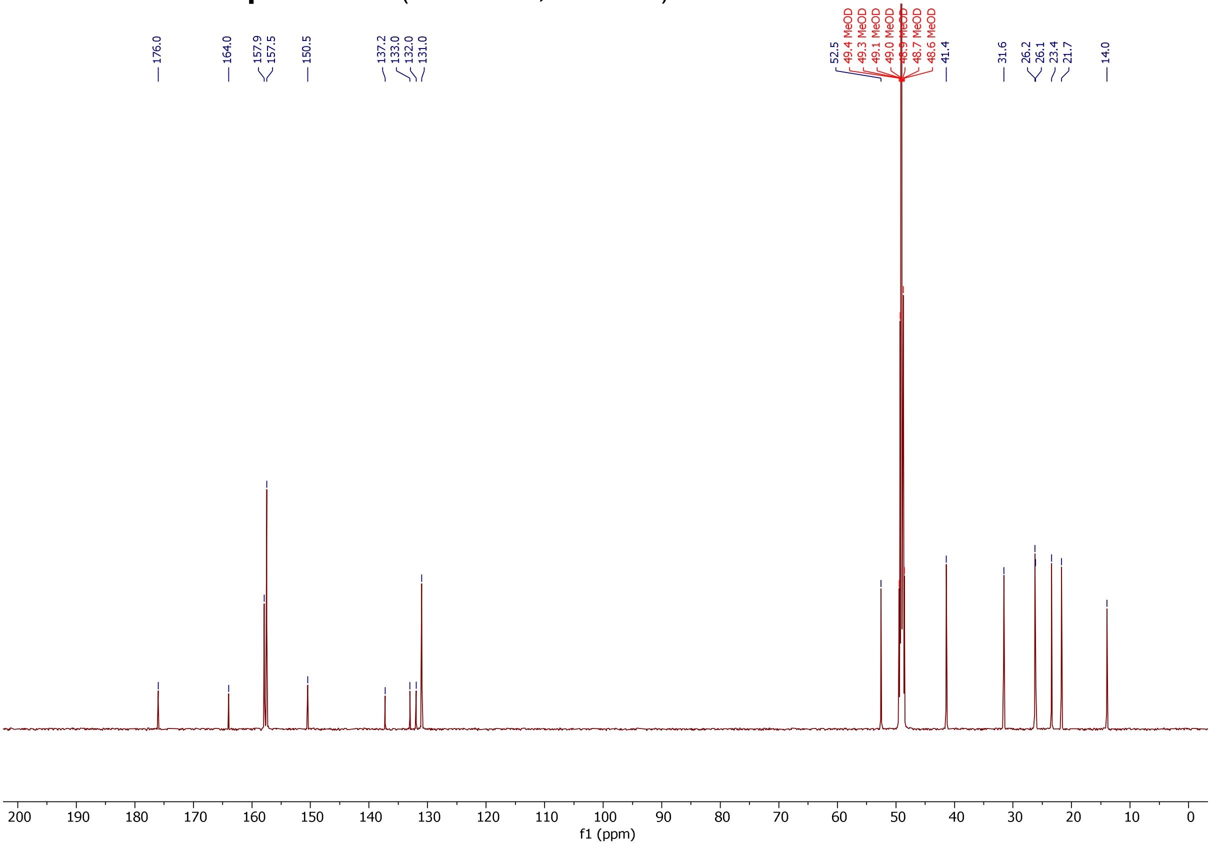

^{1}H NMR of **Compound 39** (600 MHz, CD_3OD)

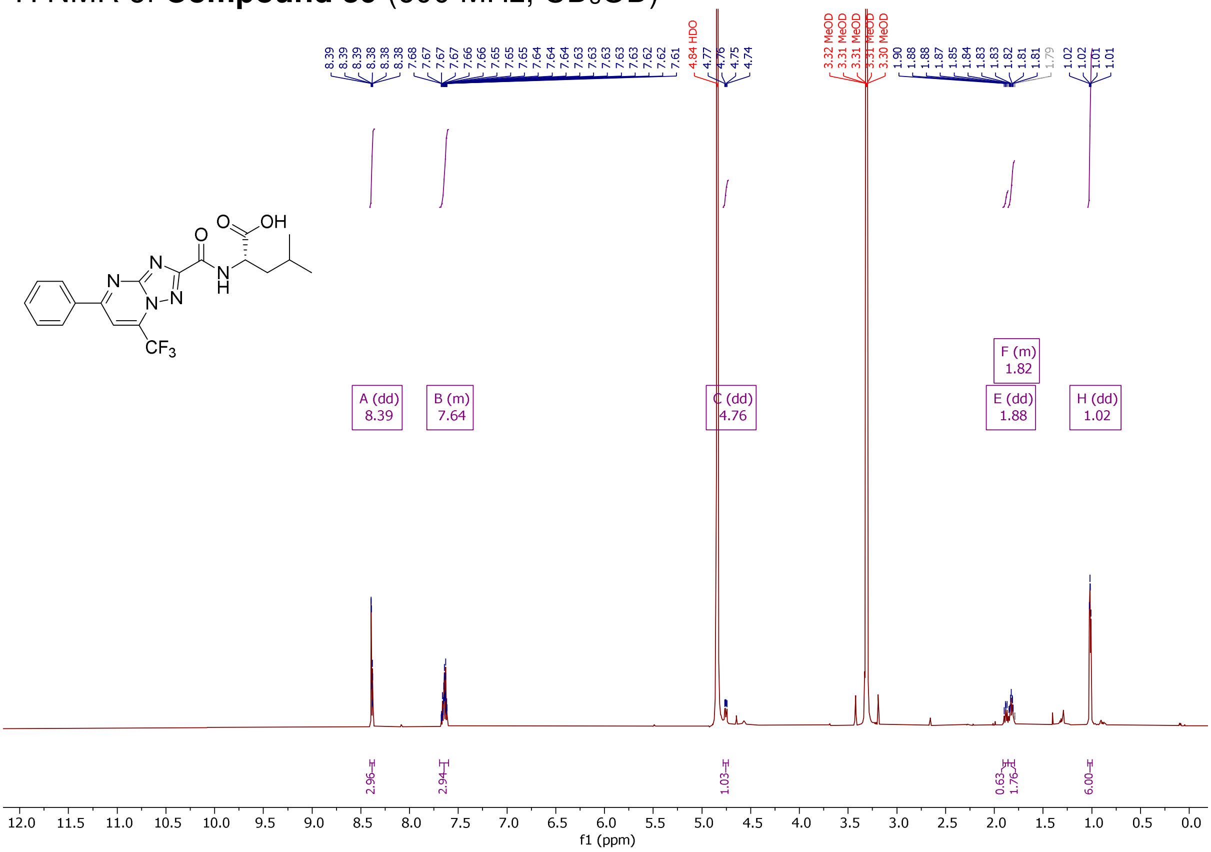

Compound 24 optimization (scaffold a) NMR spectra

^{1}H NMR of **Compound 1a** (500 MHz, CD_3OD)

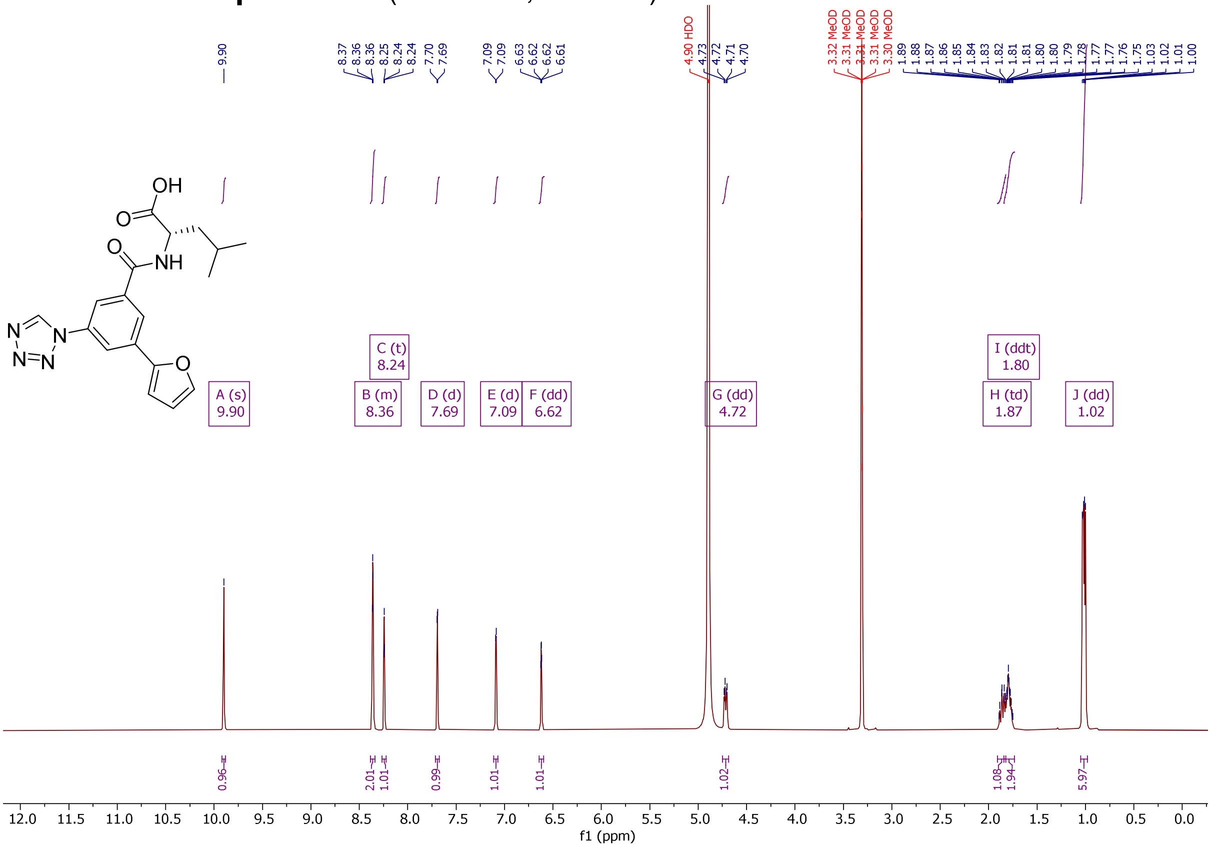

^{13}C NMR of **Compound 1a** (125 MHz, CD_3OD)

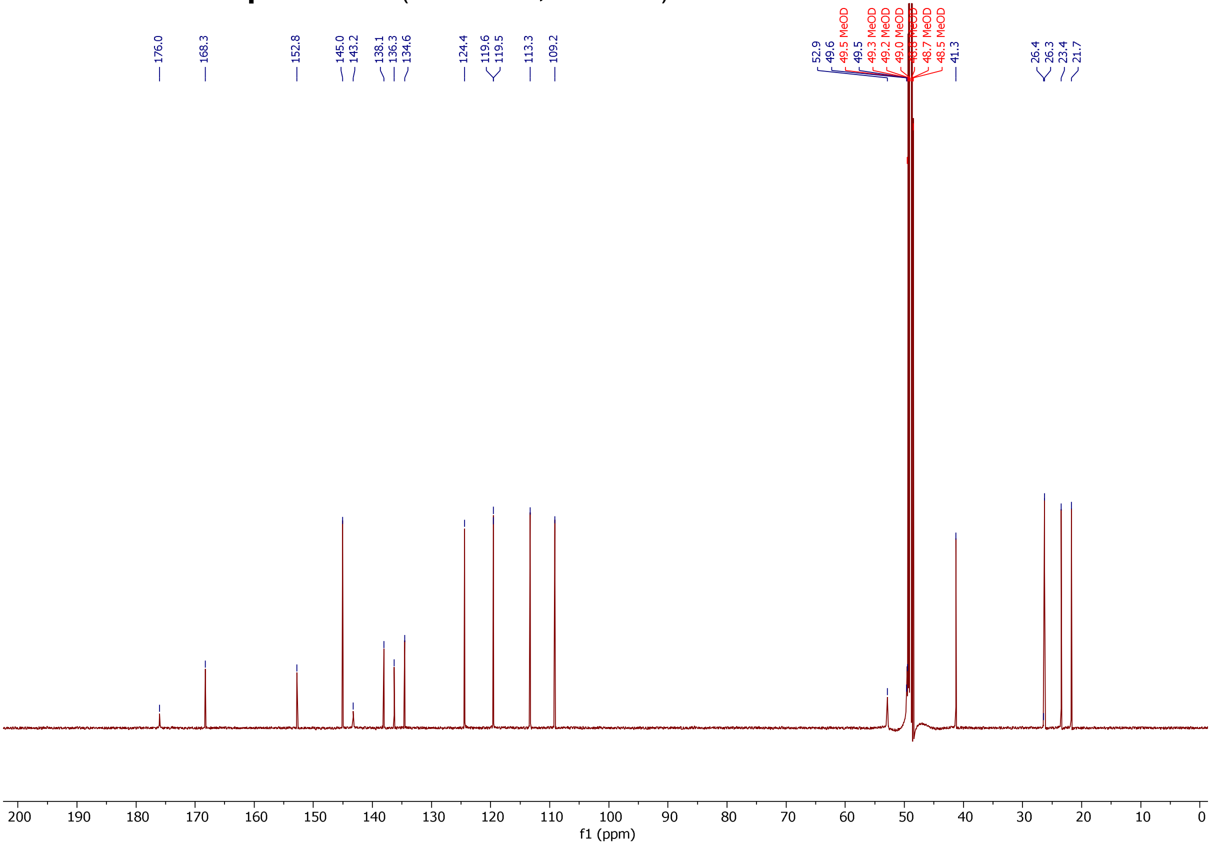

^{1}H NMR of **Compound 2a** (500 MHz, CD_3OD)

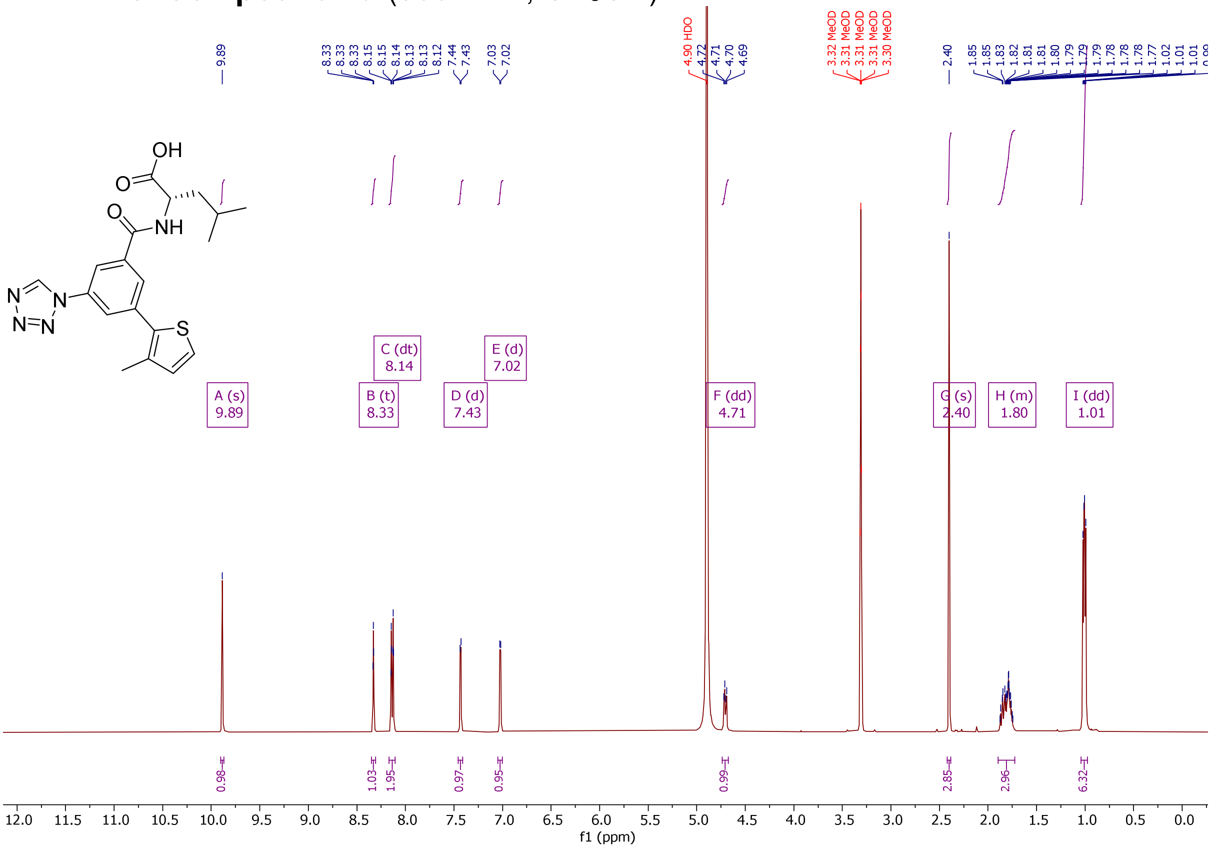

^{13}C NMR of **Compound 2a** (125 MHz, CD_3OD)

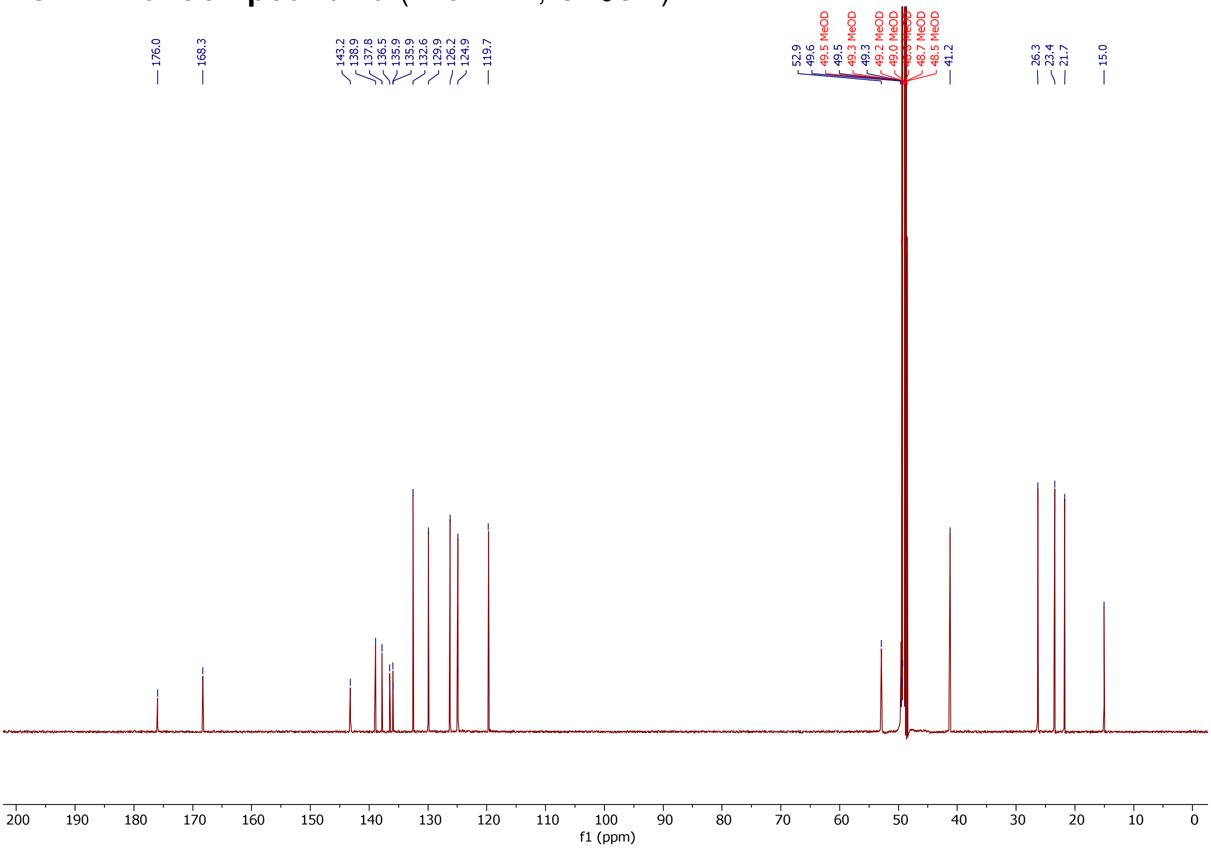

^{1}H NMR of **Compound 3a** (500 MHz, CD_3OD)

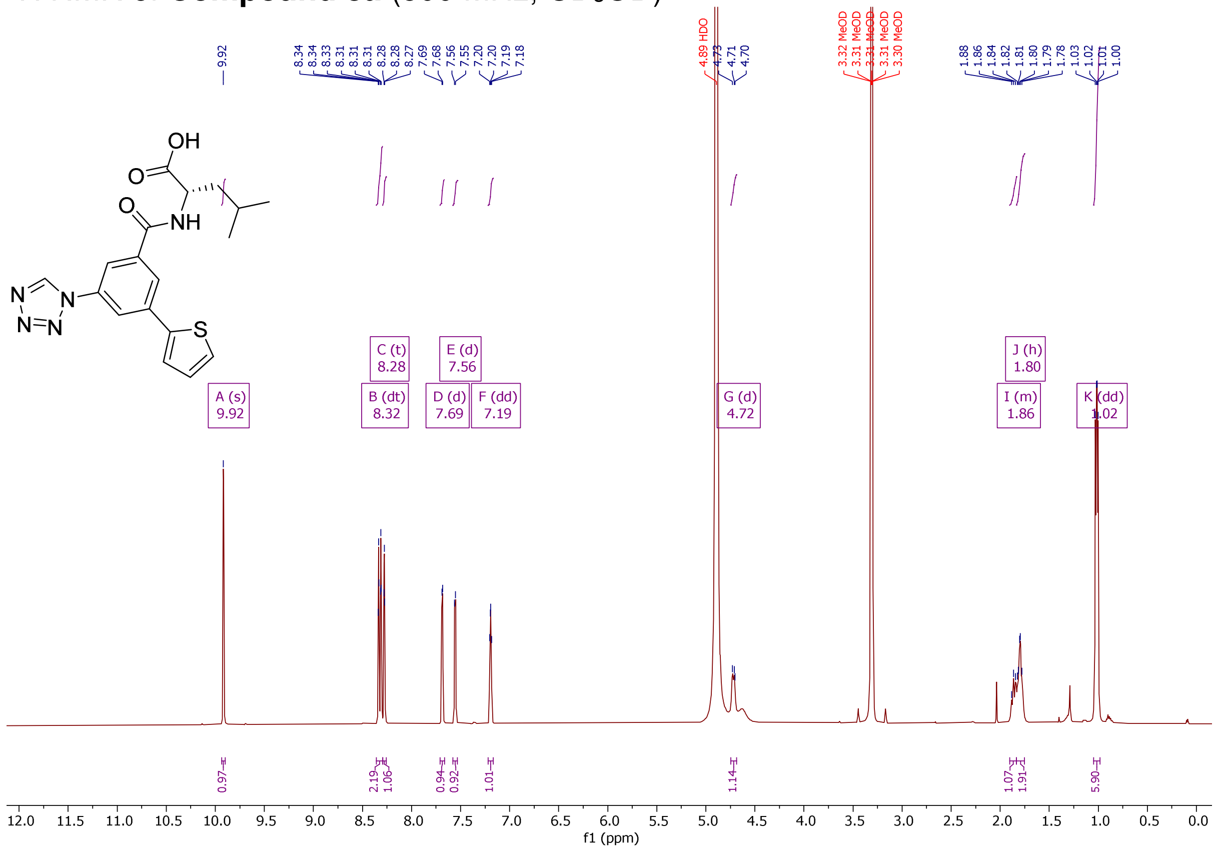

^{13}C NMR of **Compound 3a** (125 MHz, CD_3OD)

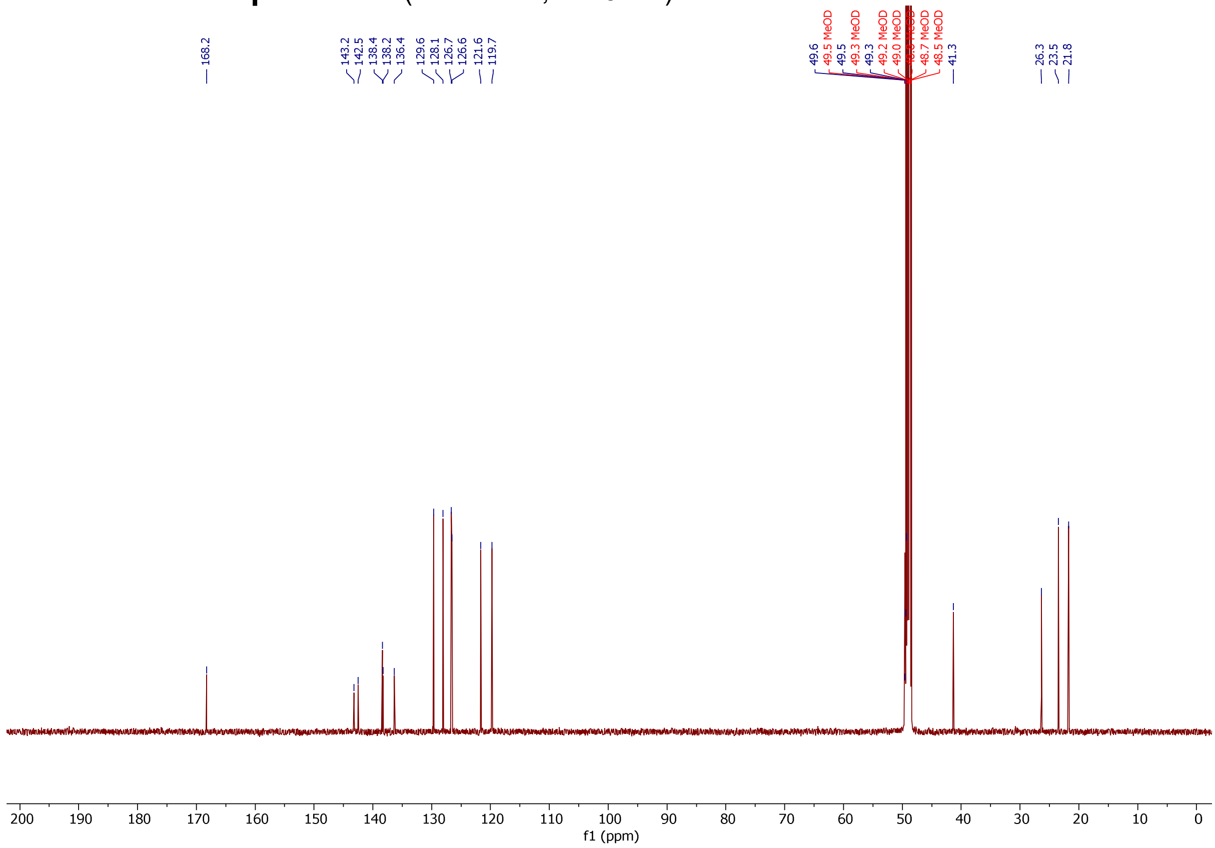

^{1}H NMR of **Compound 4a** (500 MHz, CD_3OD)

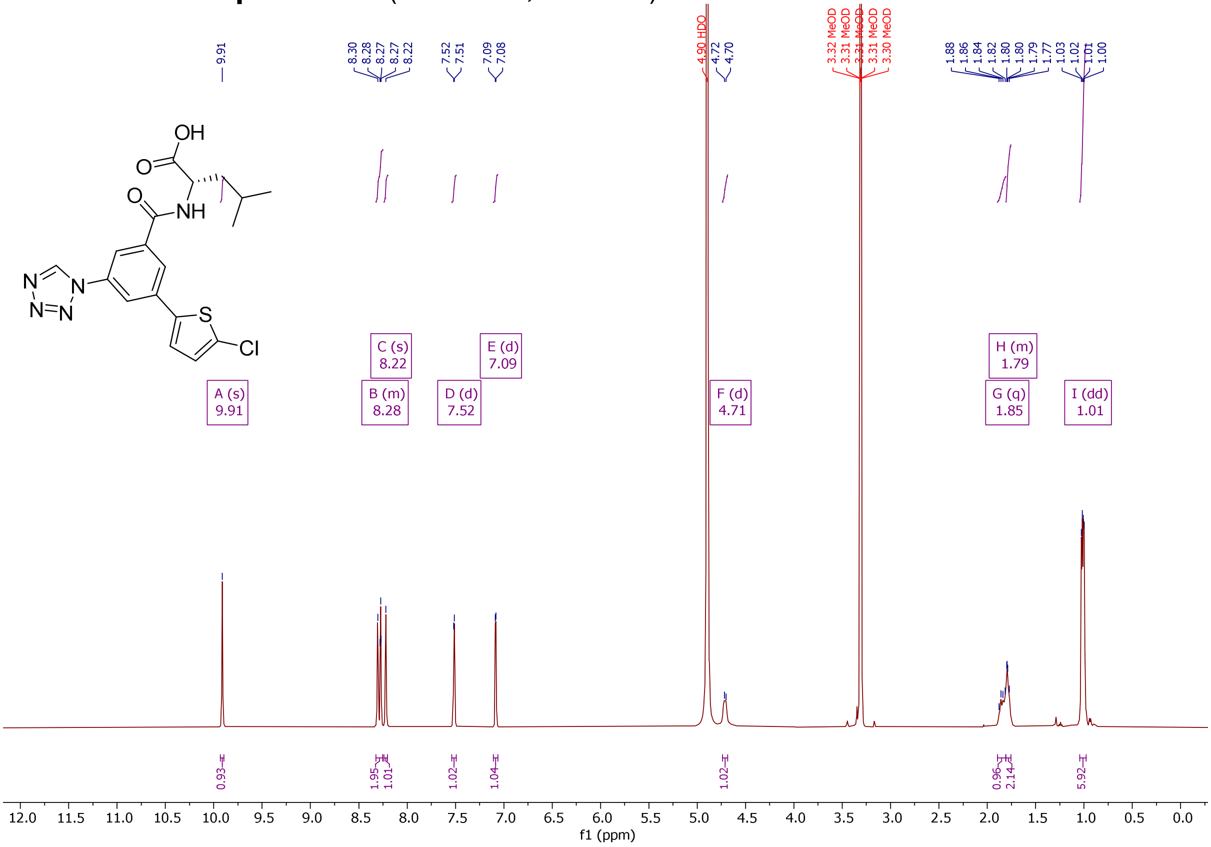

^{13}C NMR of **Compound 4a** (125 MHz, CD_3OD)

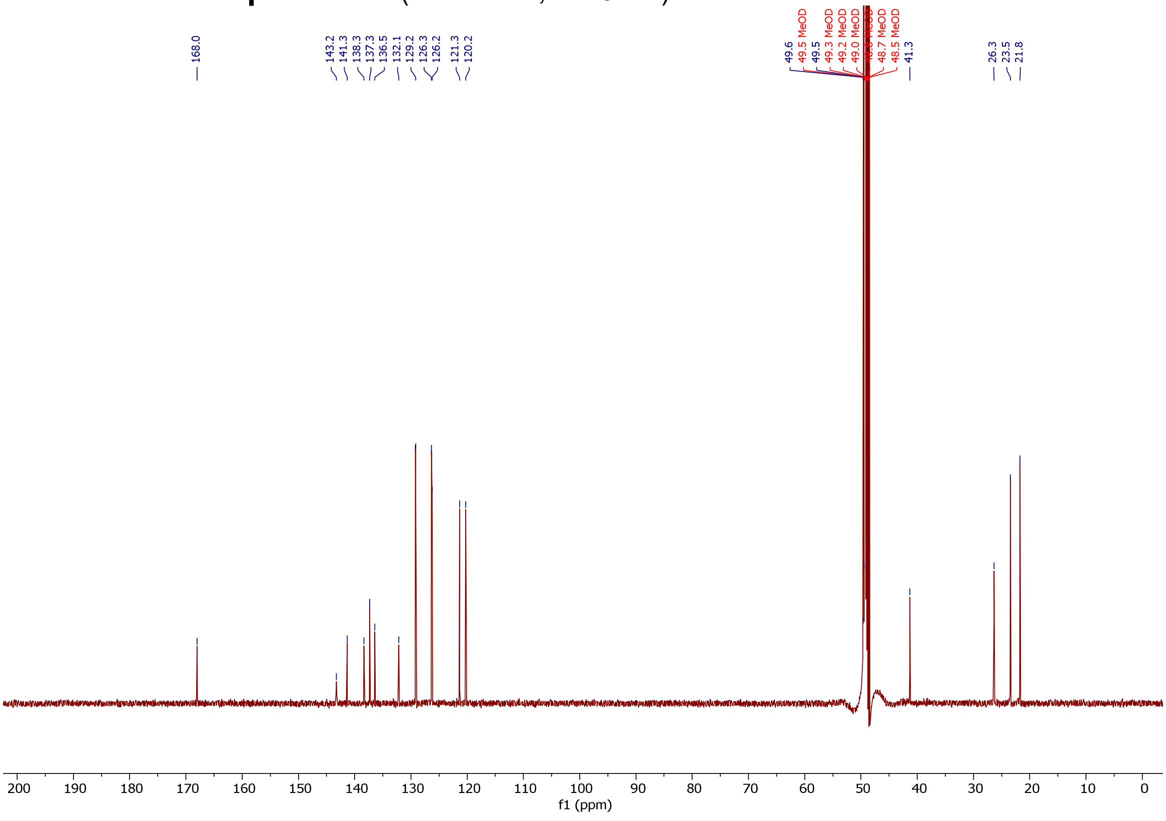

^{1}H NMR of **Compound 5a** (500 MHz, CD_3OD)

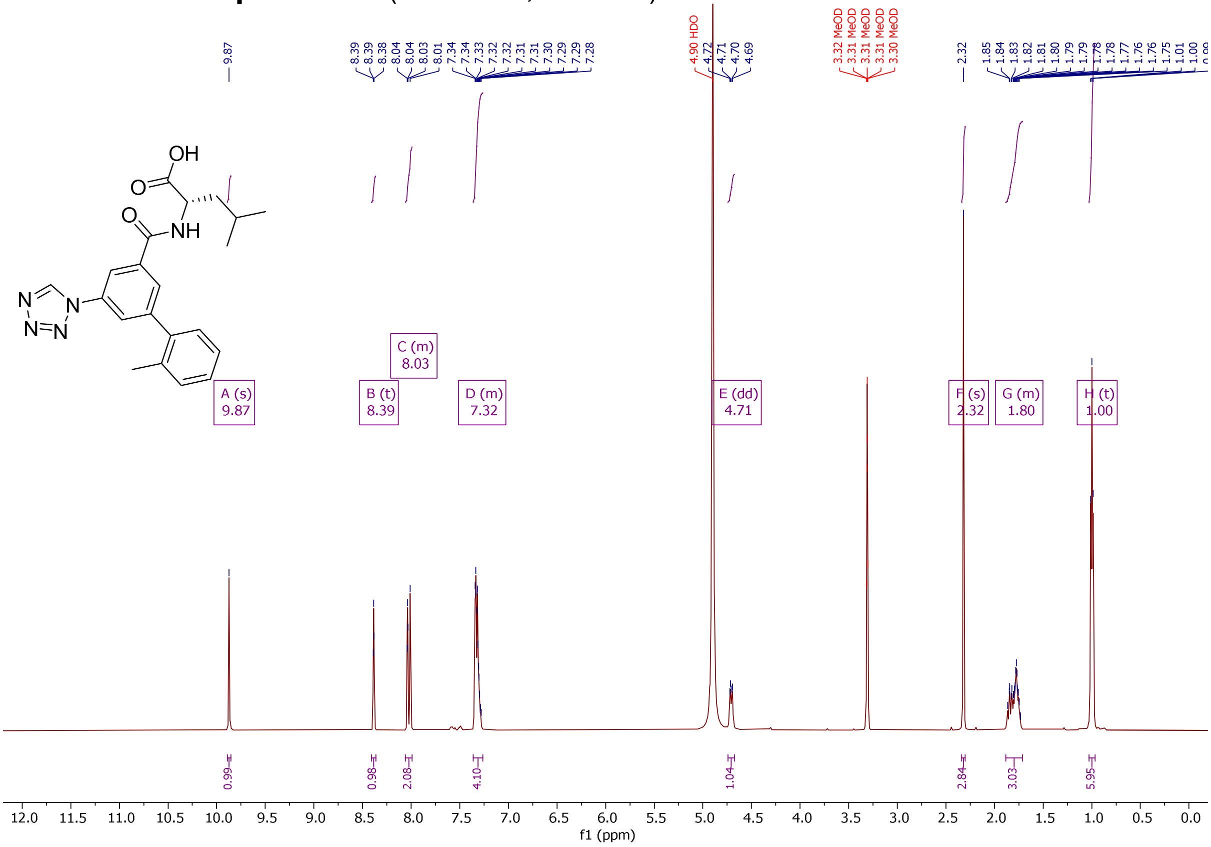

^{13}C NMR of **Compound 5a** (125 MHz, CD_3OD)

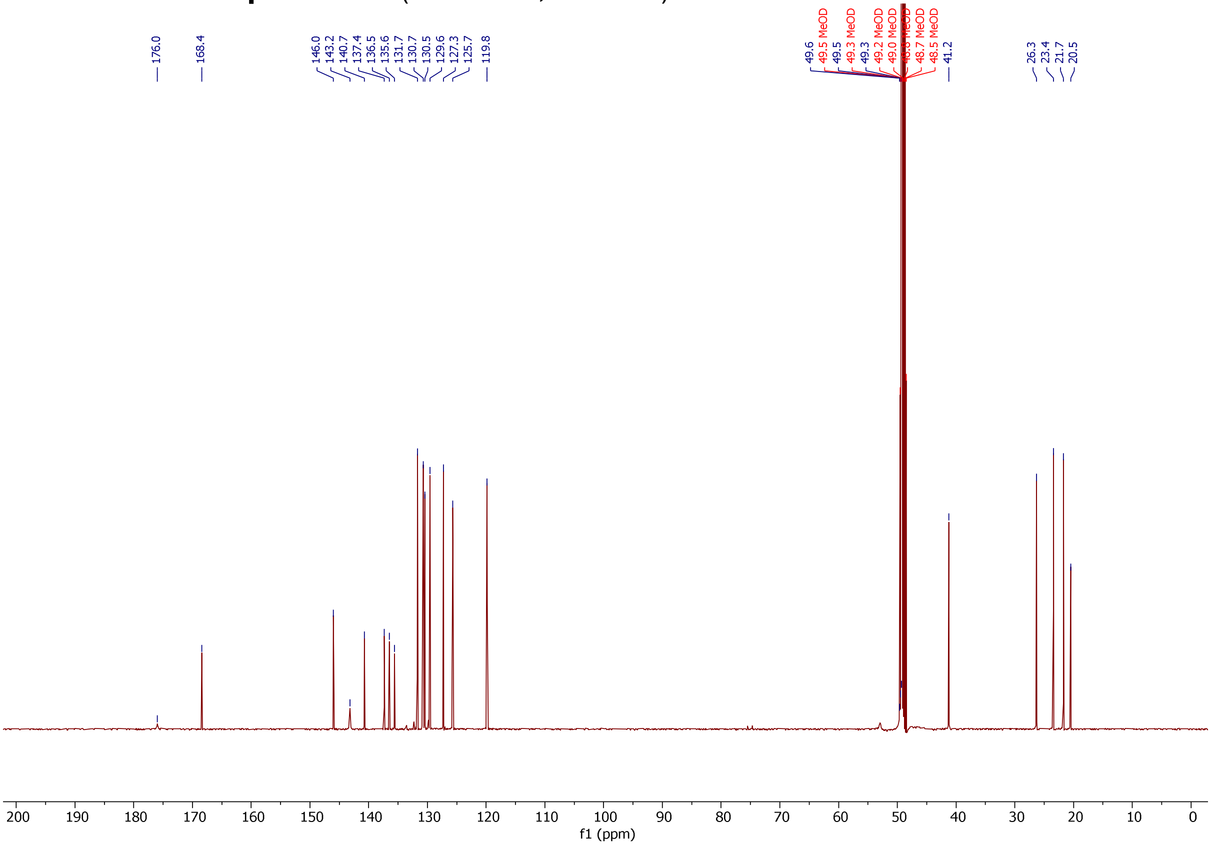

^{1}H NMR of **Compound 6a** (500 MHz, CD_3OD)

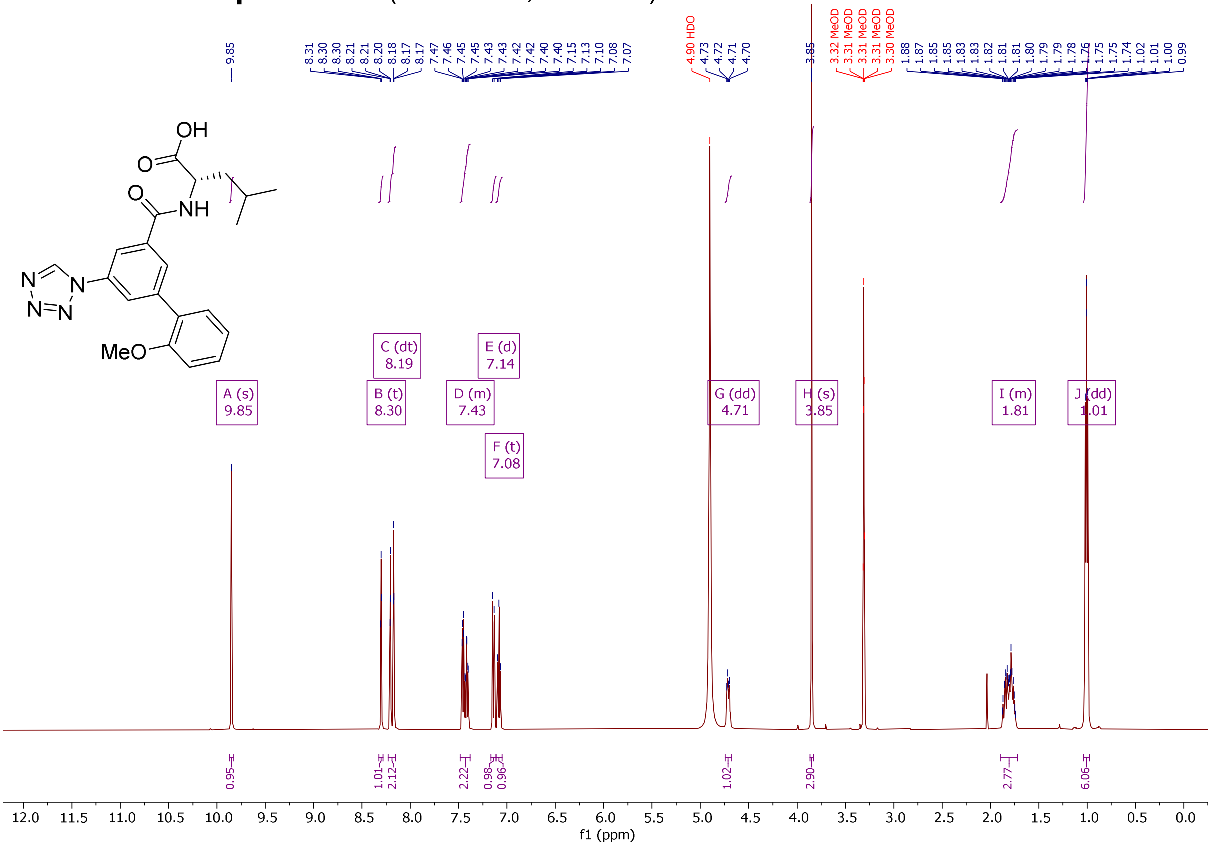

^{13}C NMR of **Compound 6a** (125 MHz, CD_3OD)

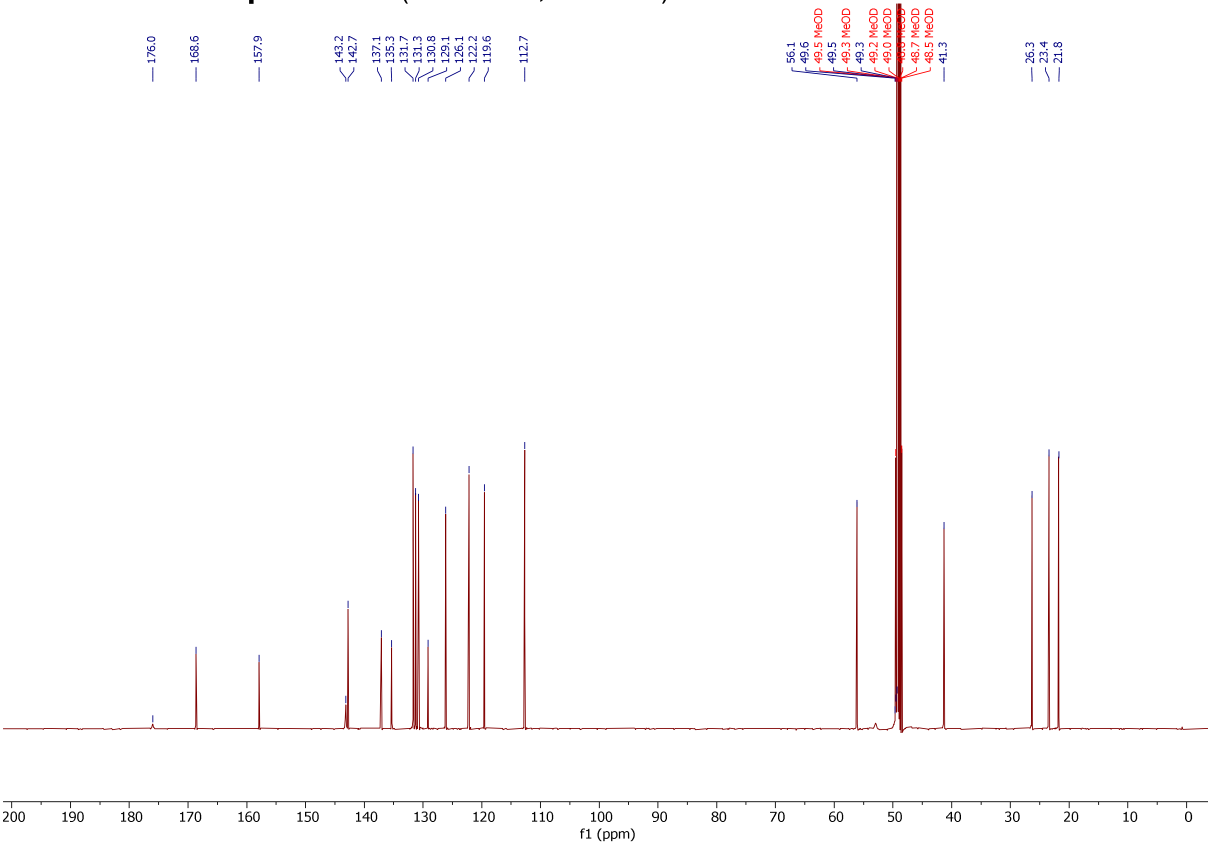

^{1}H NMR of **Compound 7a** (500 MHz, CD_3OD)

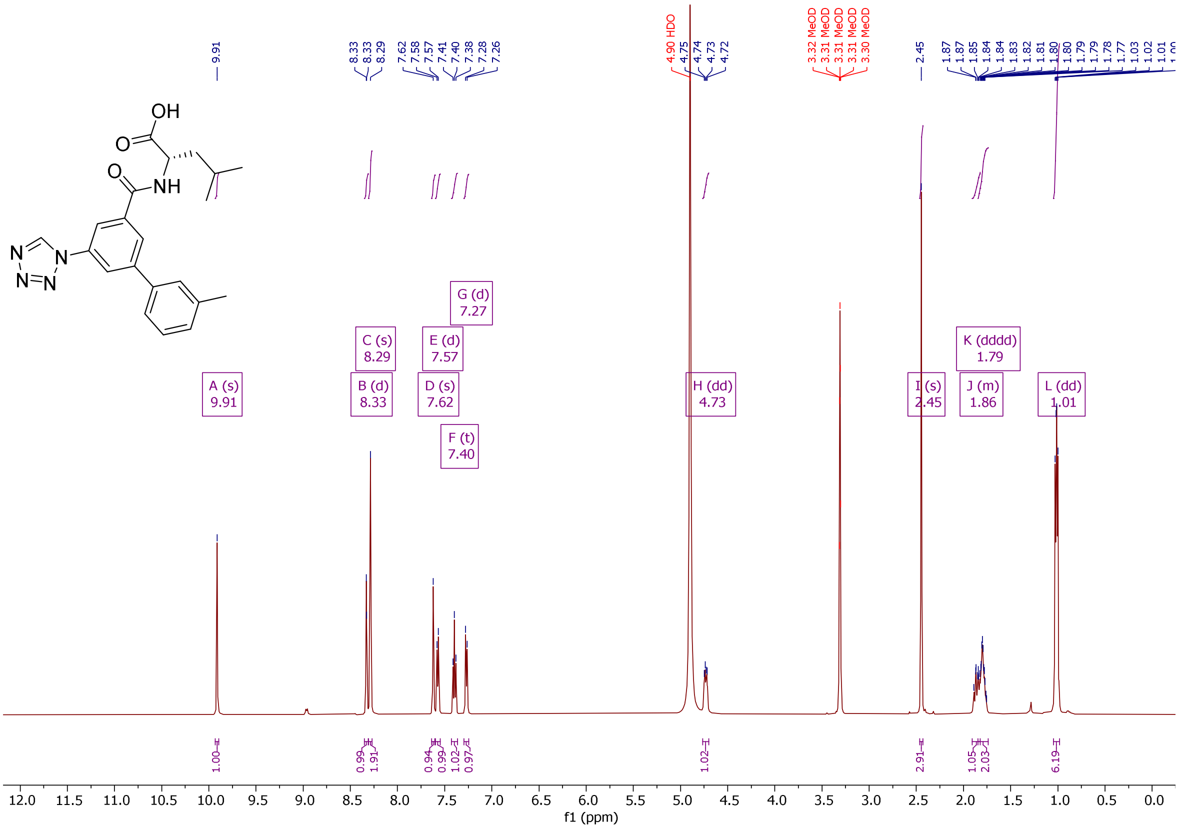

^{13}C NMR of **Compound 7a** (125 MHz, CD_3OD)

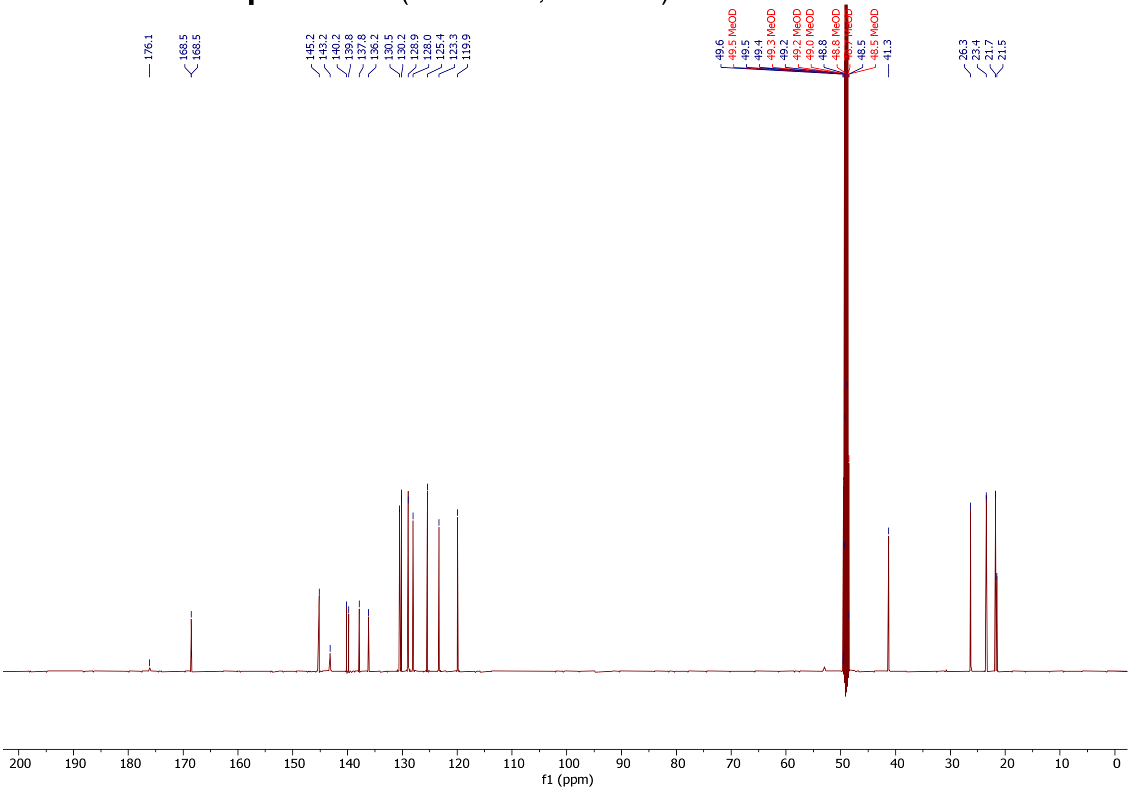

^{1}H NMR of **Compound 8a** (500 MHz, CD_3OD)

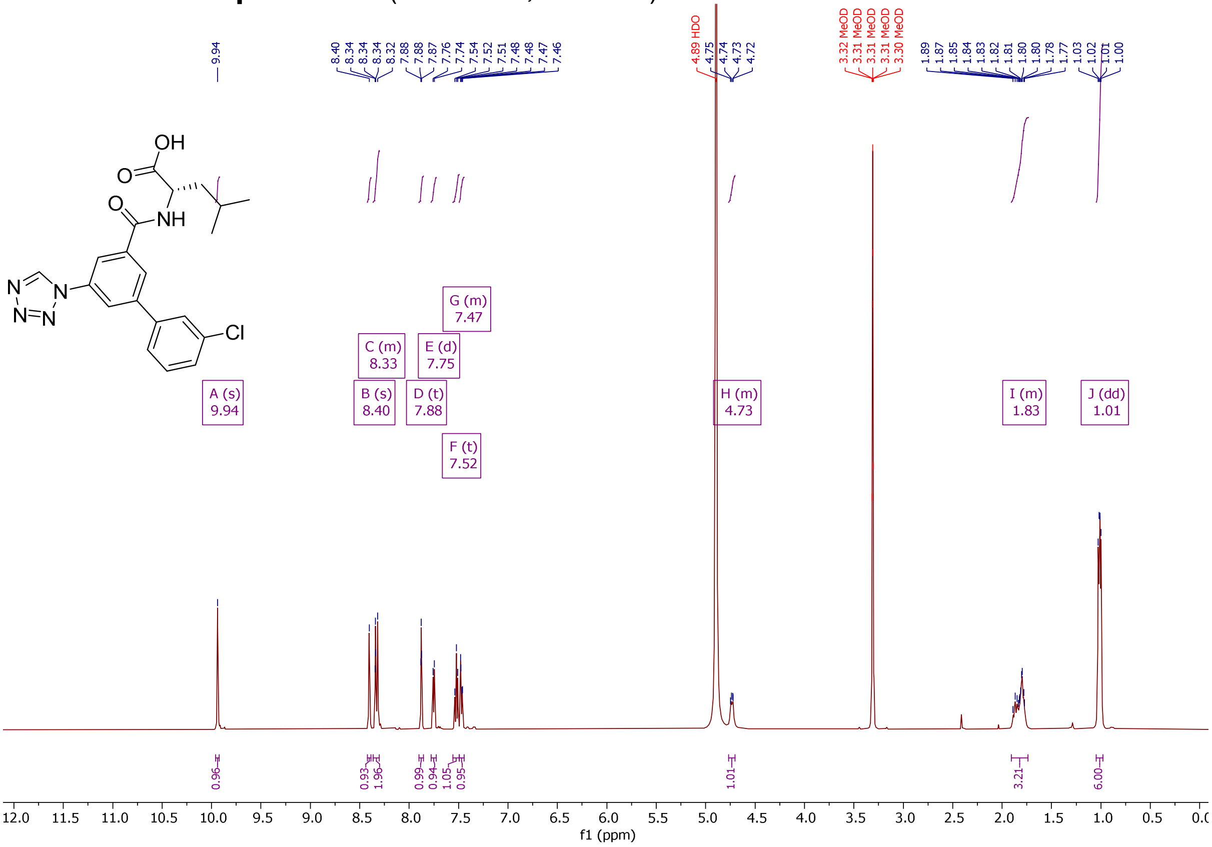

^{13}C NMR of **Compound 8a** (125 MHz, CD_3OD)

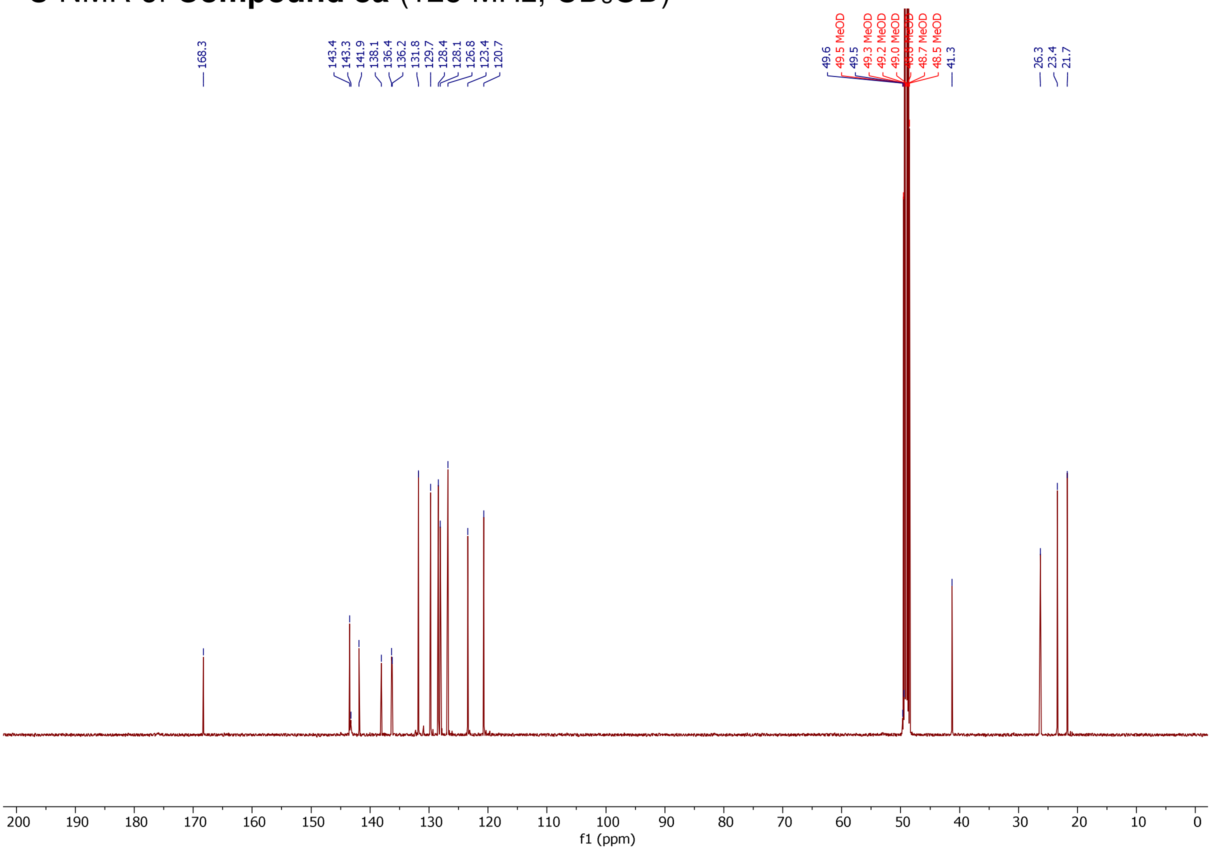

^{1}H NMR of **Compound 9a** (500 MHz, CD_3OD)

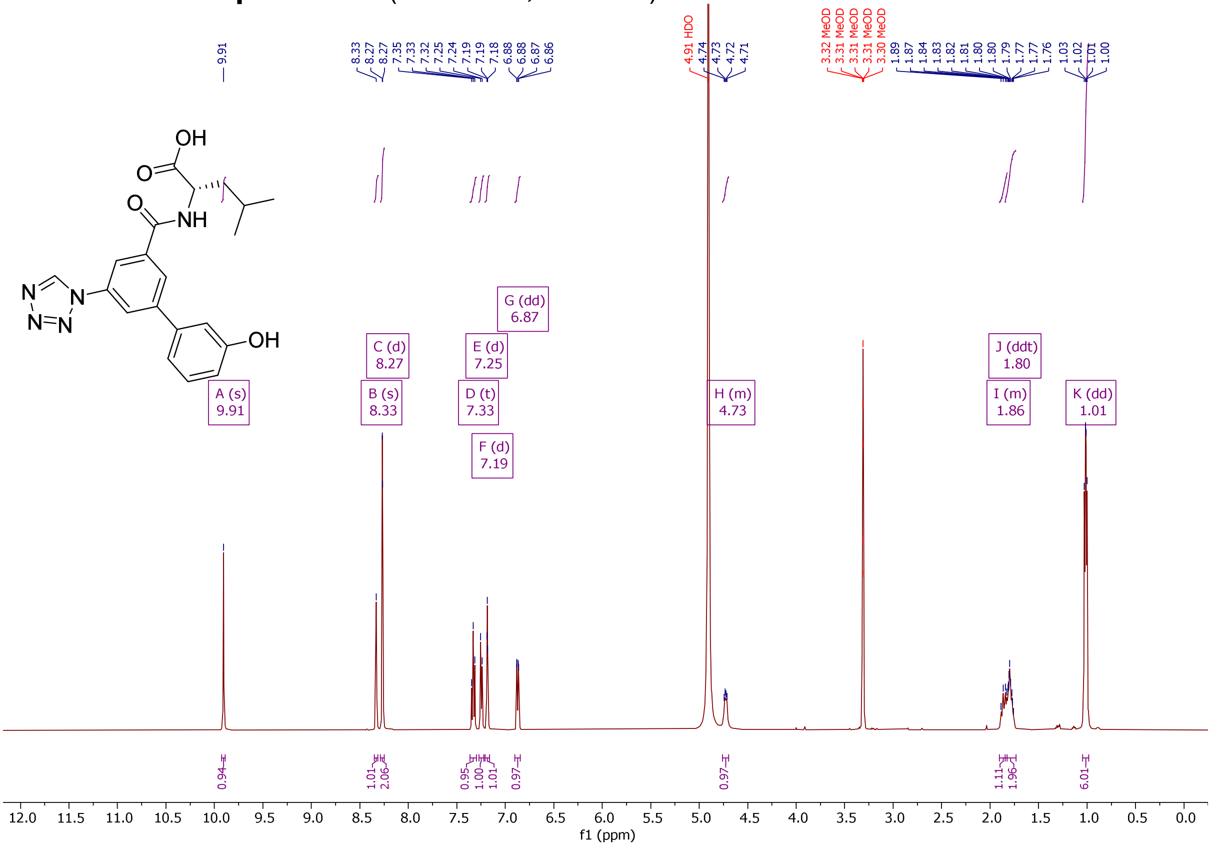

^{13}C NMR of **Compound 9a** (125 MHz, CD_3OD)

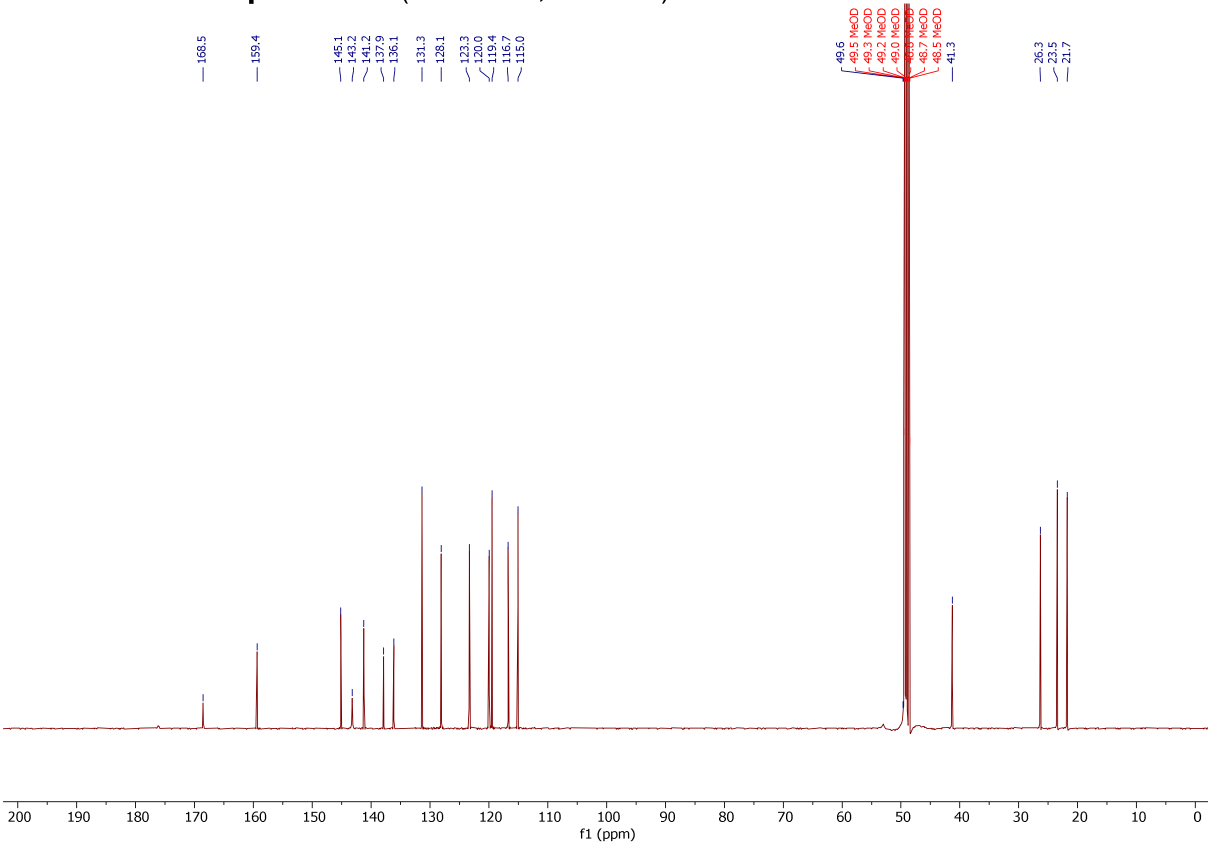

^{1}H NMR of **Compound 10a** (500 MHz, CD_3OD)

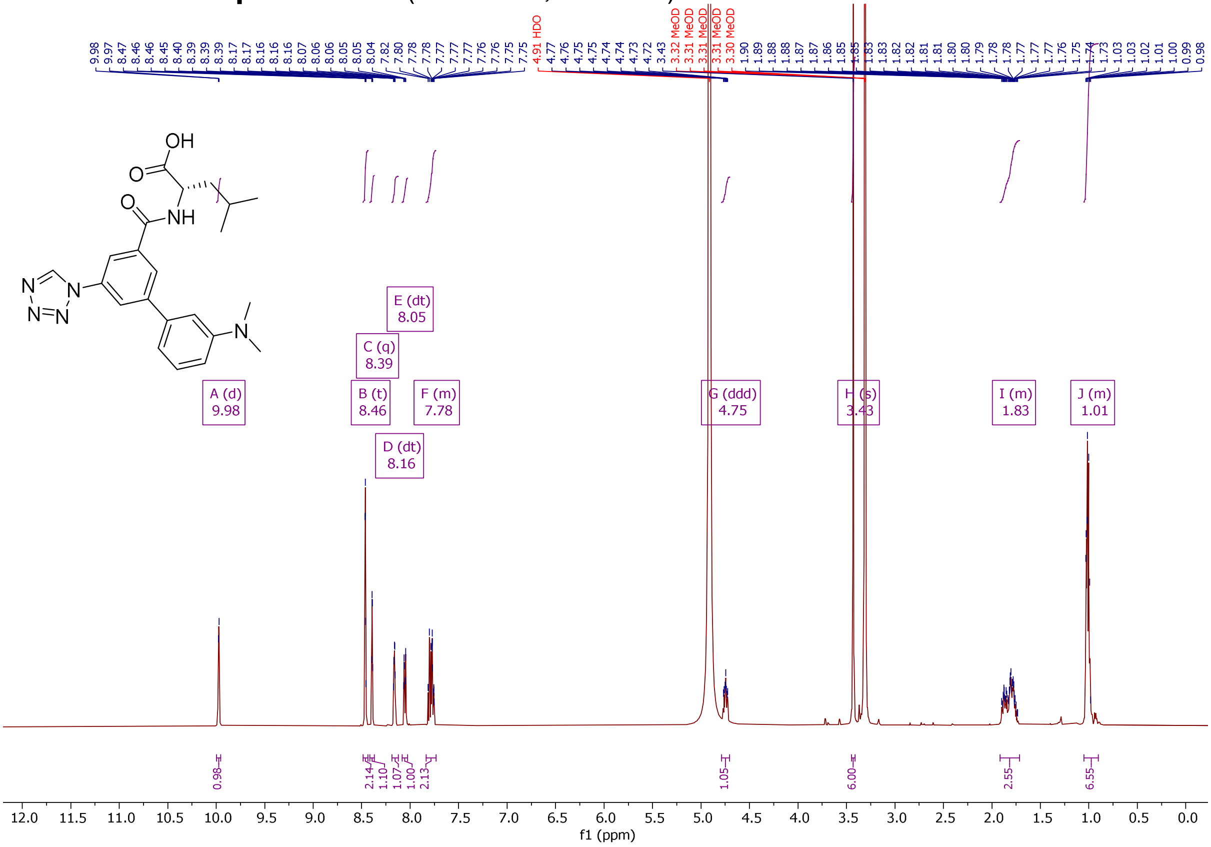

^{13}C NMR of **Compound 10a** (125 MHz, CD_3OD)

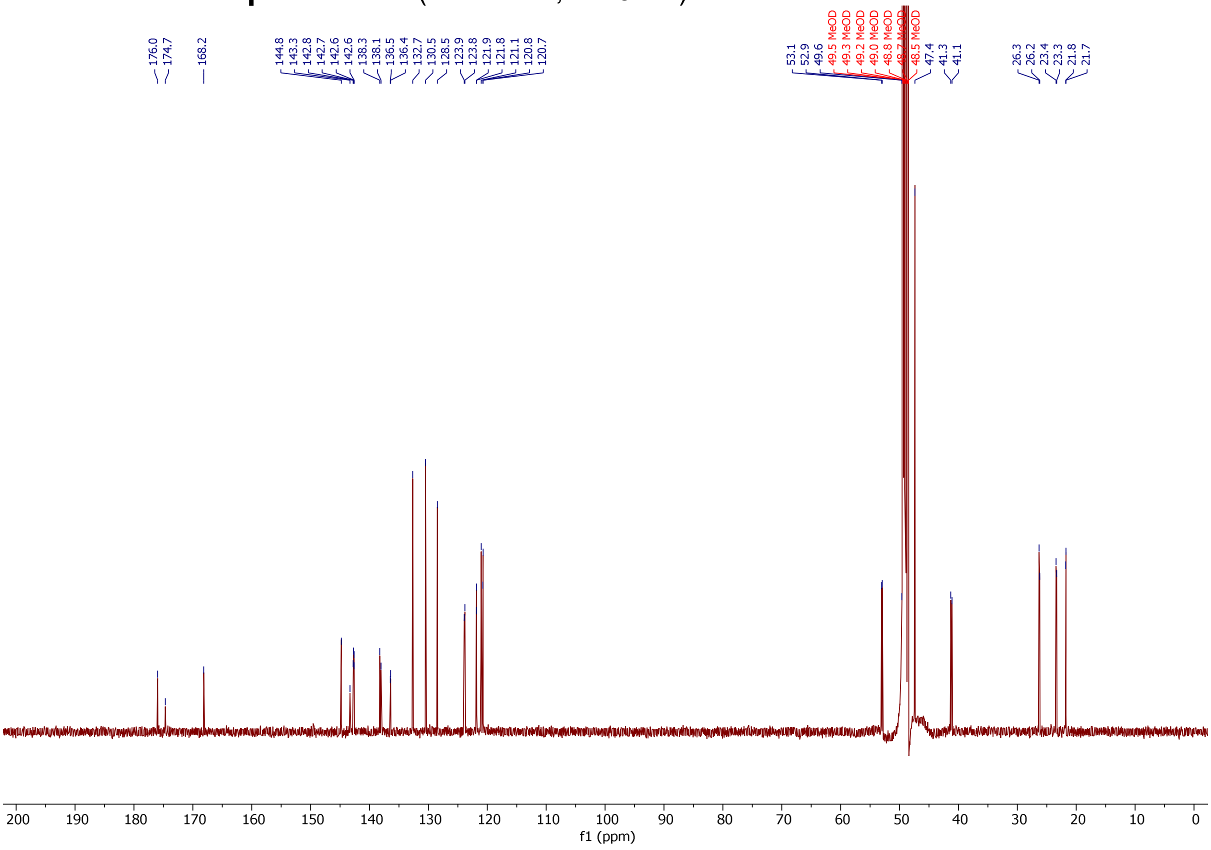

^{1}H NMR of **Compound 11a** (500 MHz, CD_3OD)

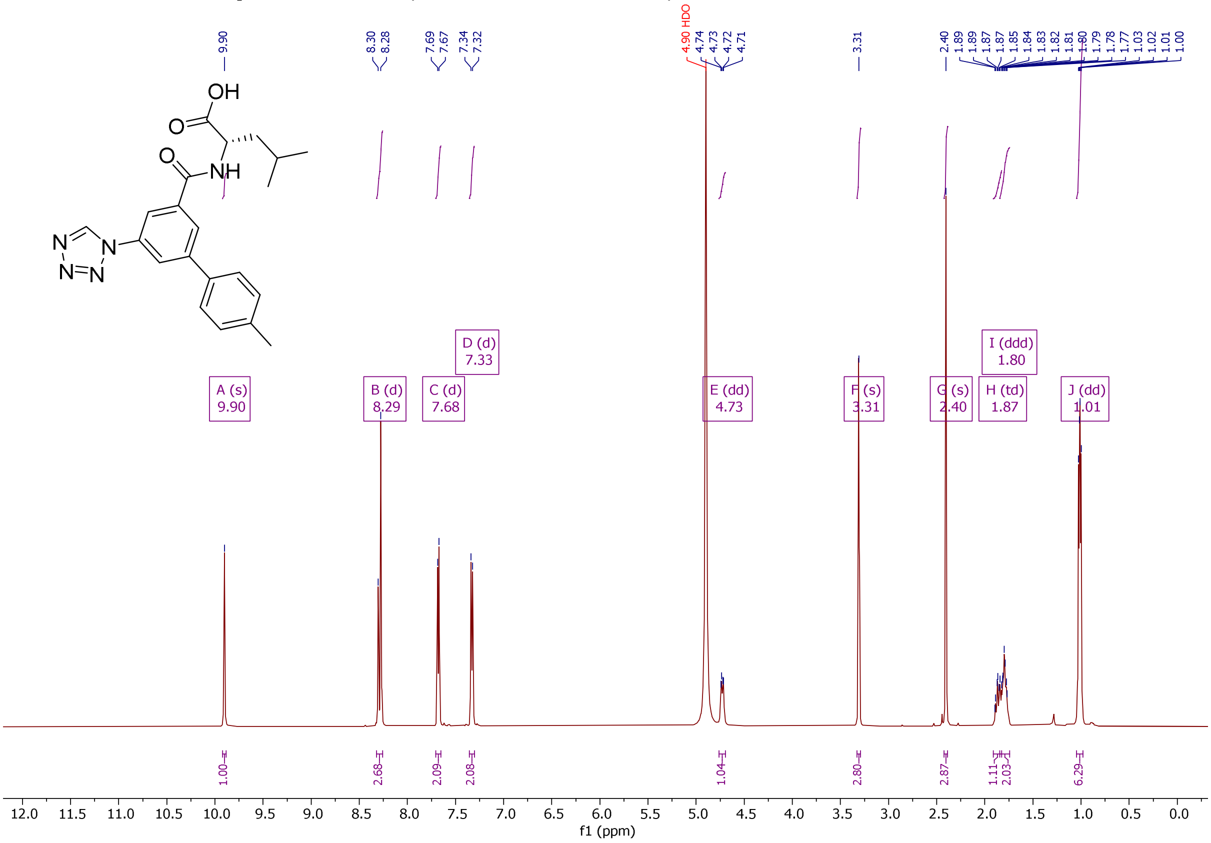

^{13}C NMR of **Compound 11a** (125 MHz, CD_3OD)

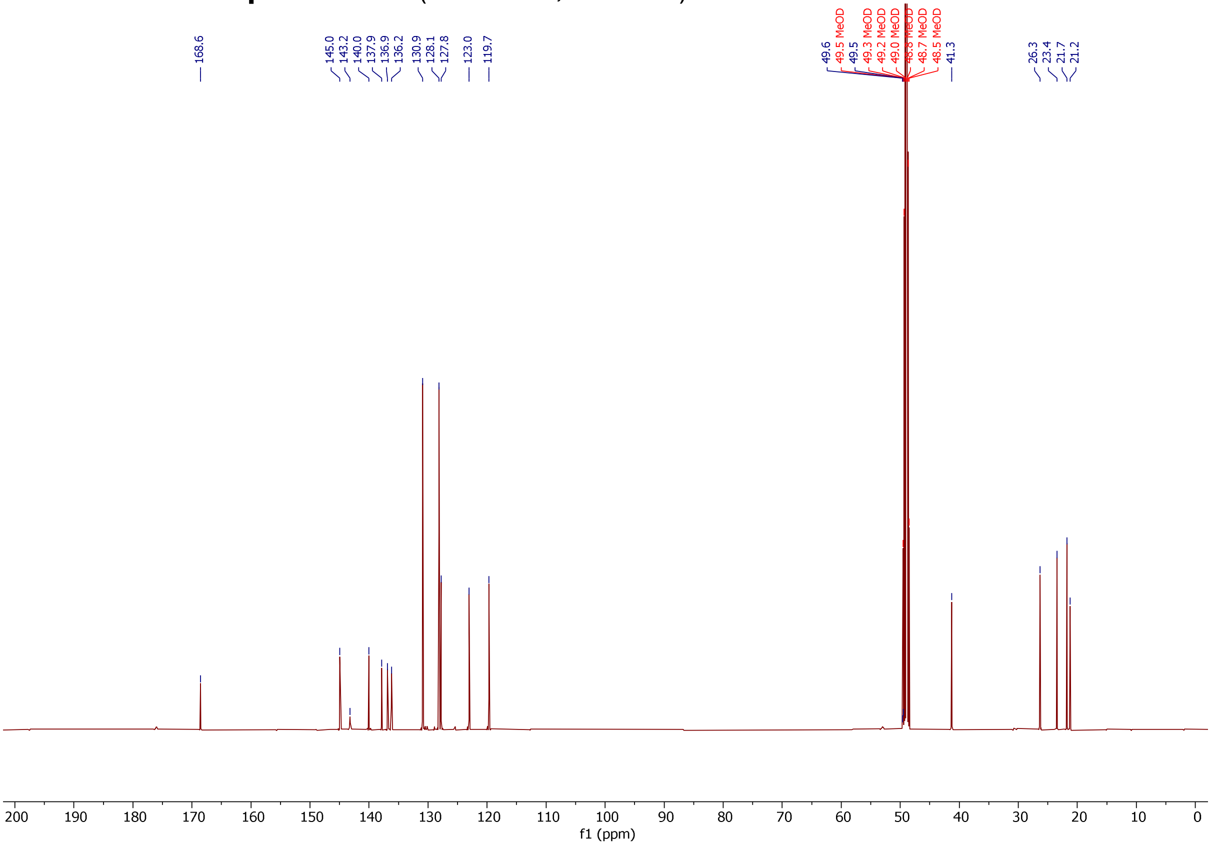

^{1}H NMR of **Compound 12a** (500 MHz, CD_3OD)

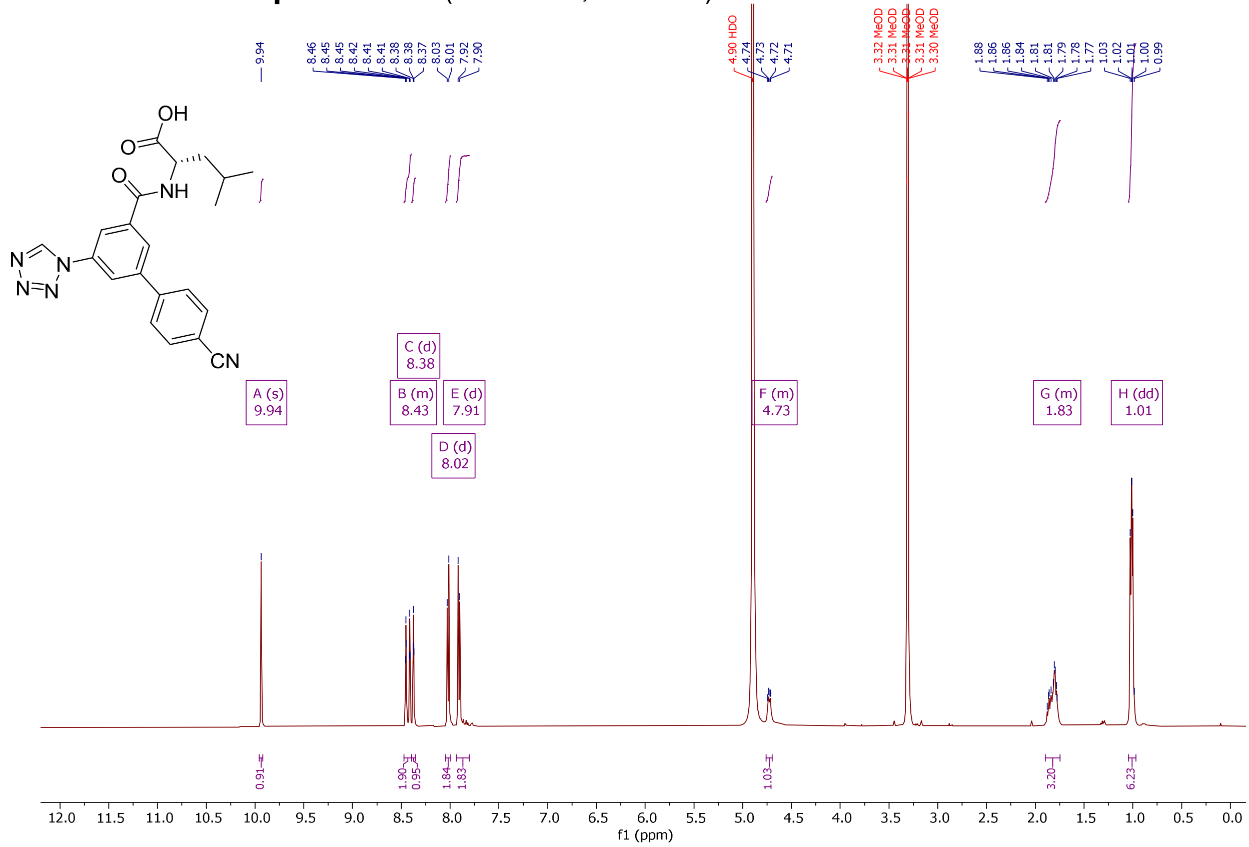

^{13}C NMR of **Compound 12a** (125 MHz, CD_3OD)

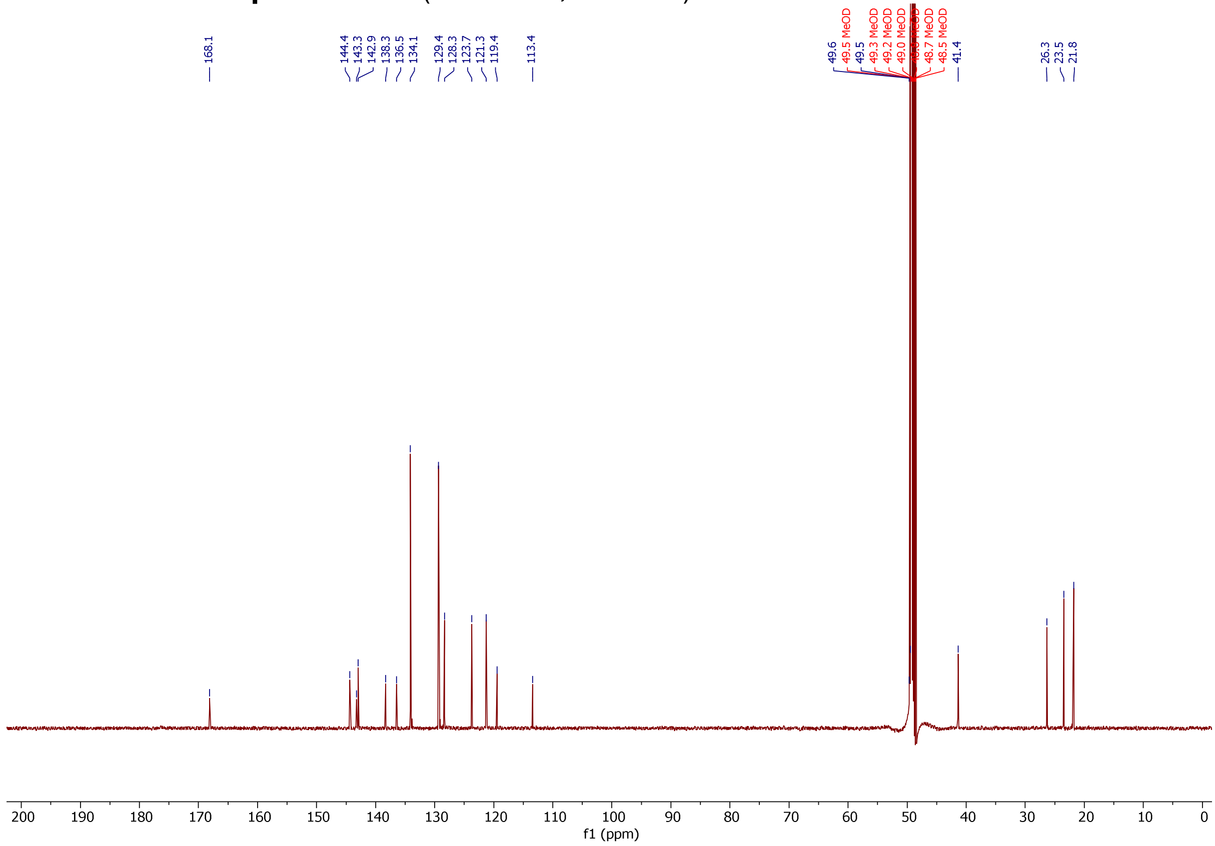

^{1}H NMR of **Compound 13a** (500 MHz, CD_3OD)

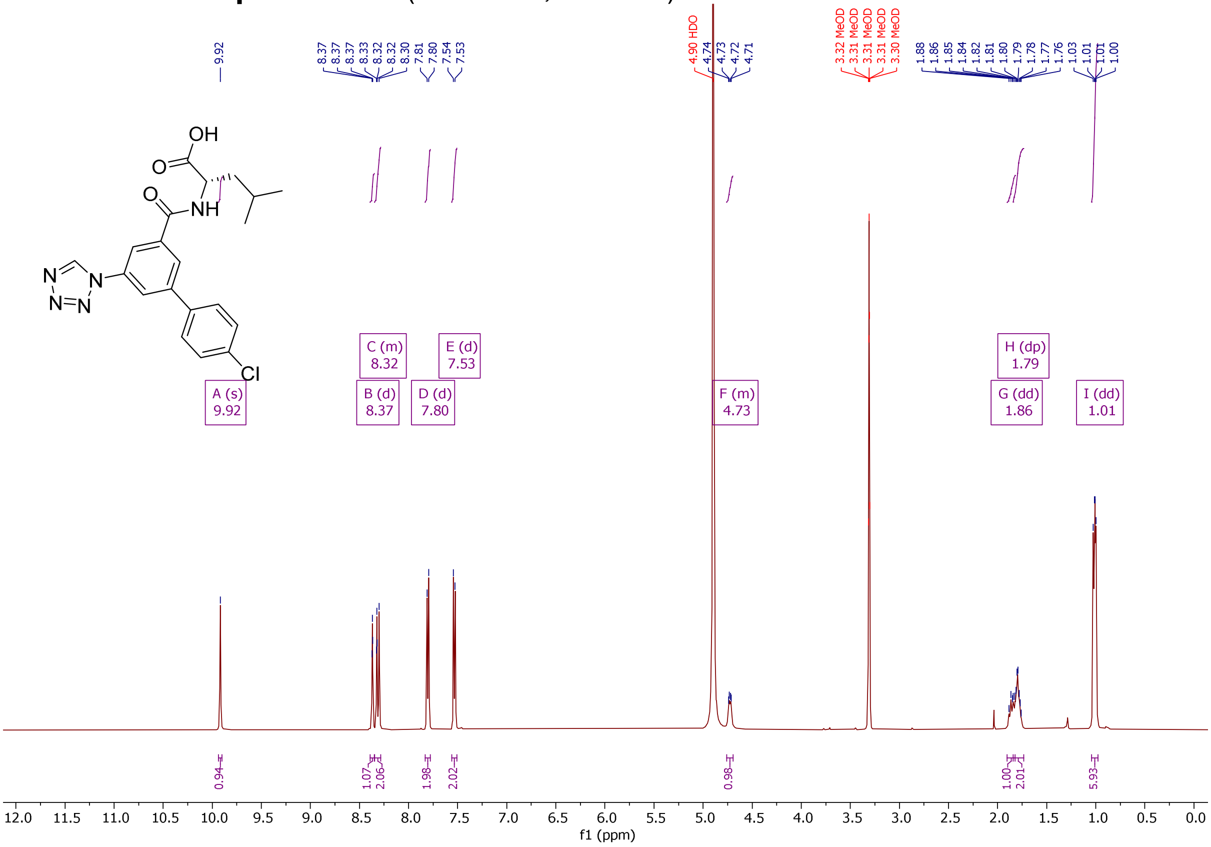

^{13}C NMR of **Compound 13a** (125 MHz, CD_3OD)

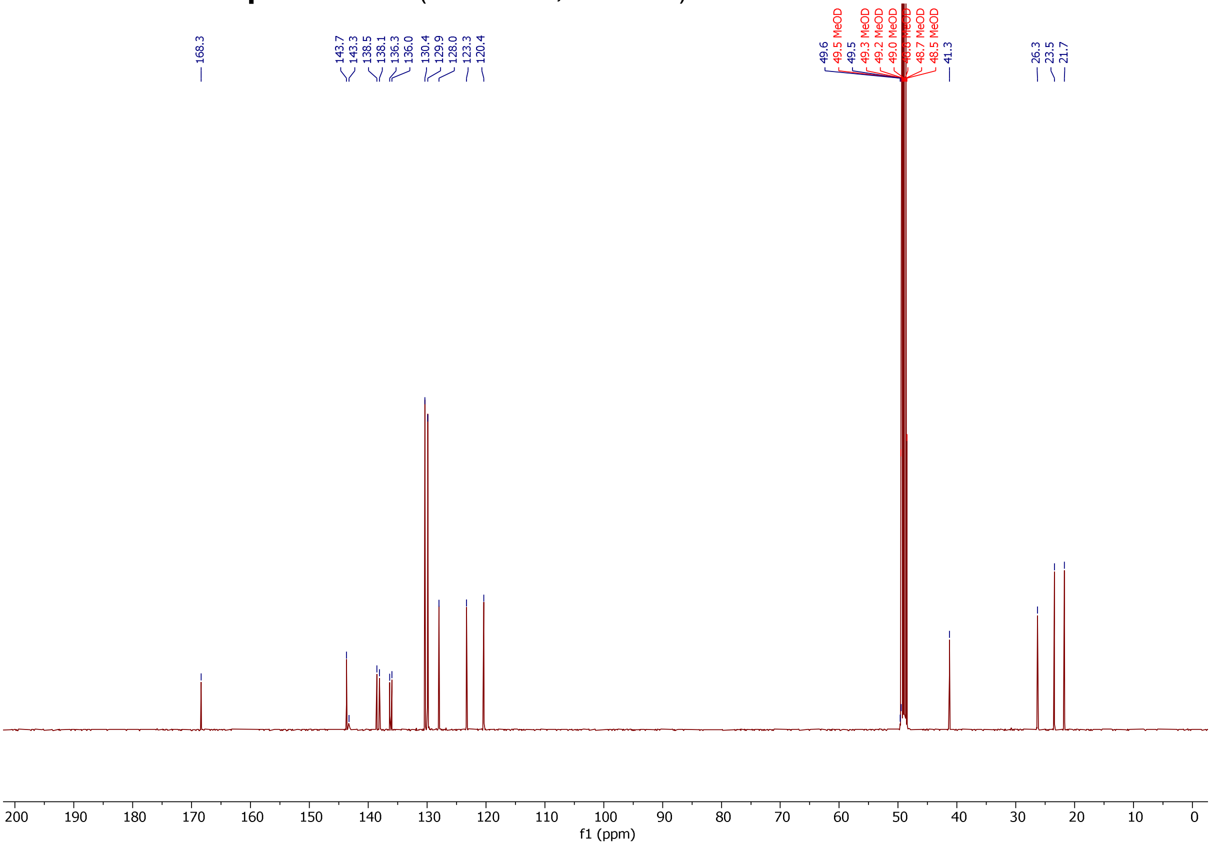

^{1}H NMR of **Compound 14a** (500 MHz, CD_3OD)

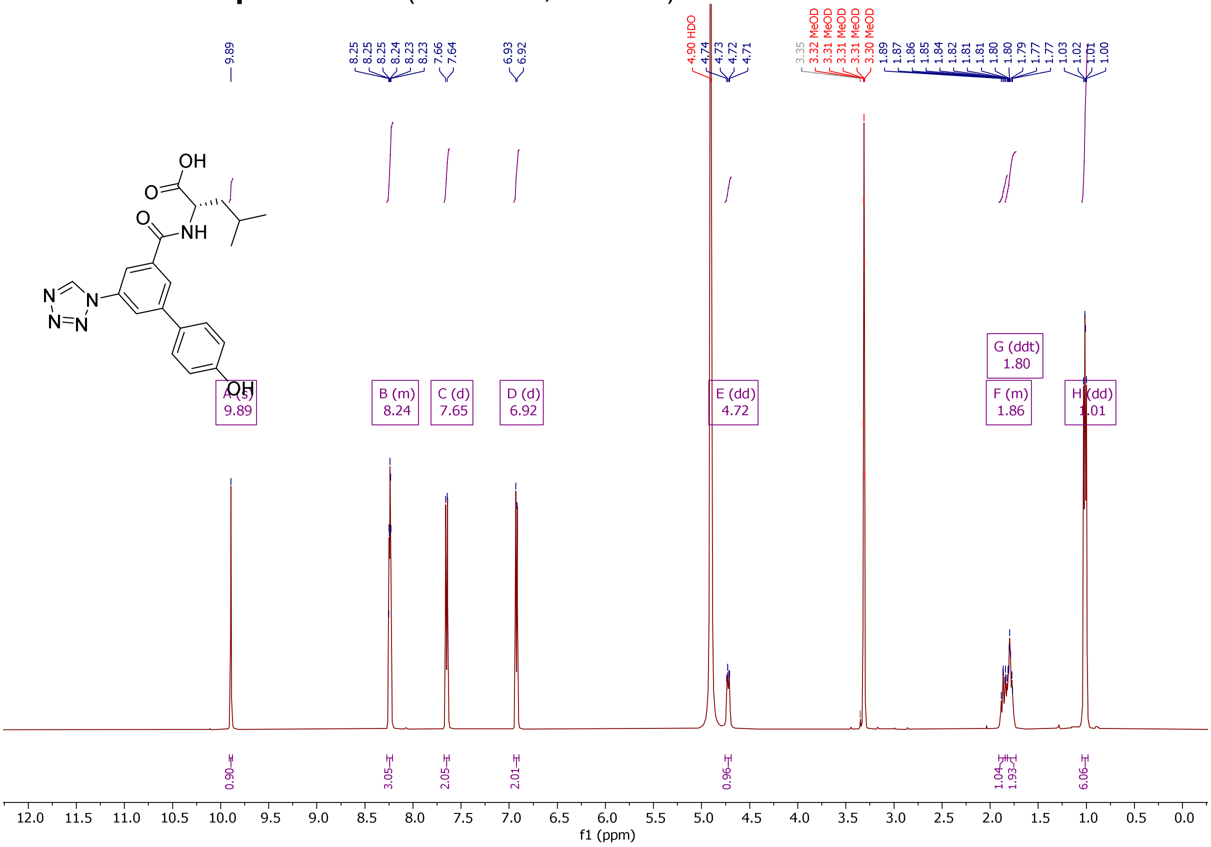

^{13}C NMR of **Compound 14a** (125 MHz, CD_3OD)

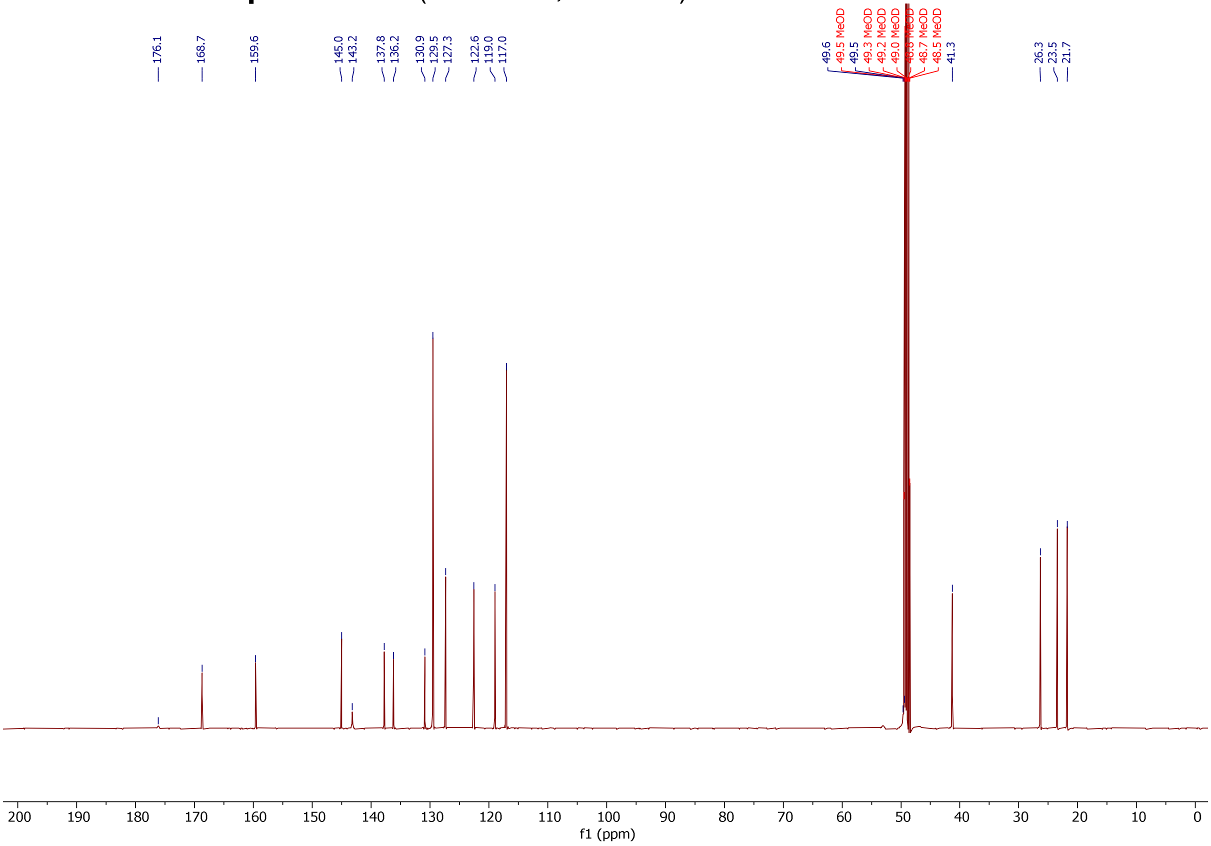

^{1}H NMR of **Compound 15a** (500 MHz, CD_3OD)

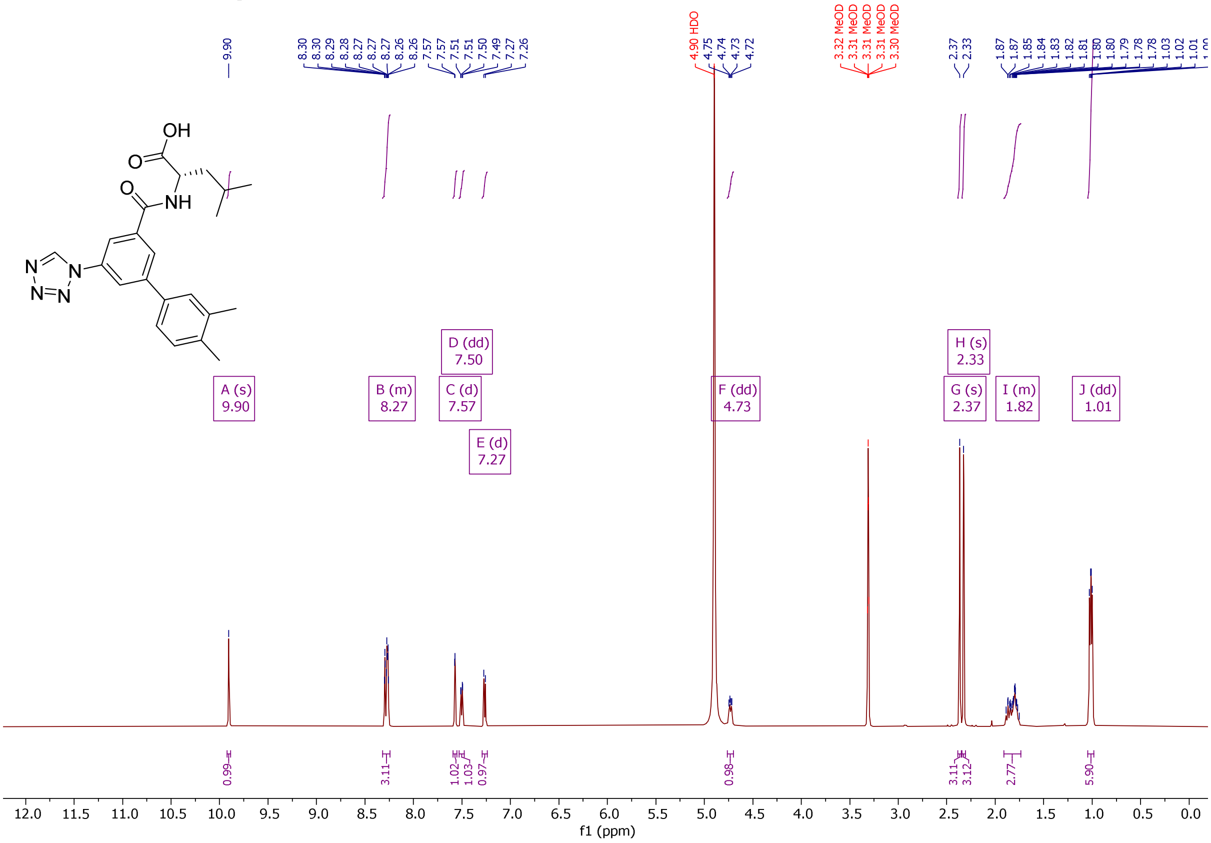

^{13}C NMR of **Compound 15a** (125 MHz, CD_3OD)

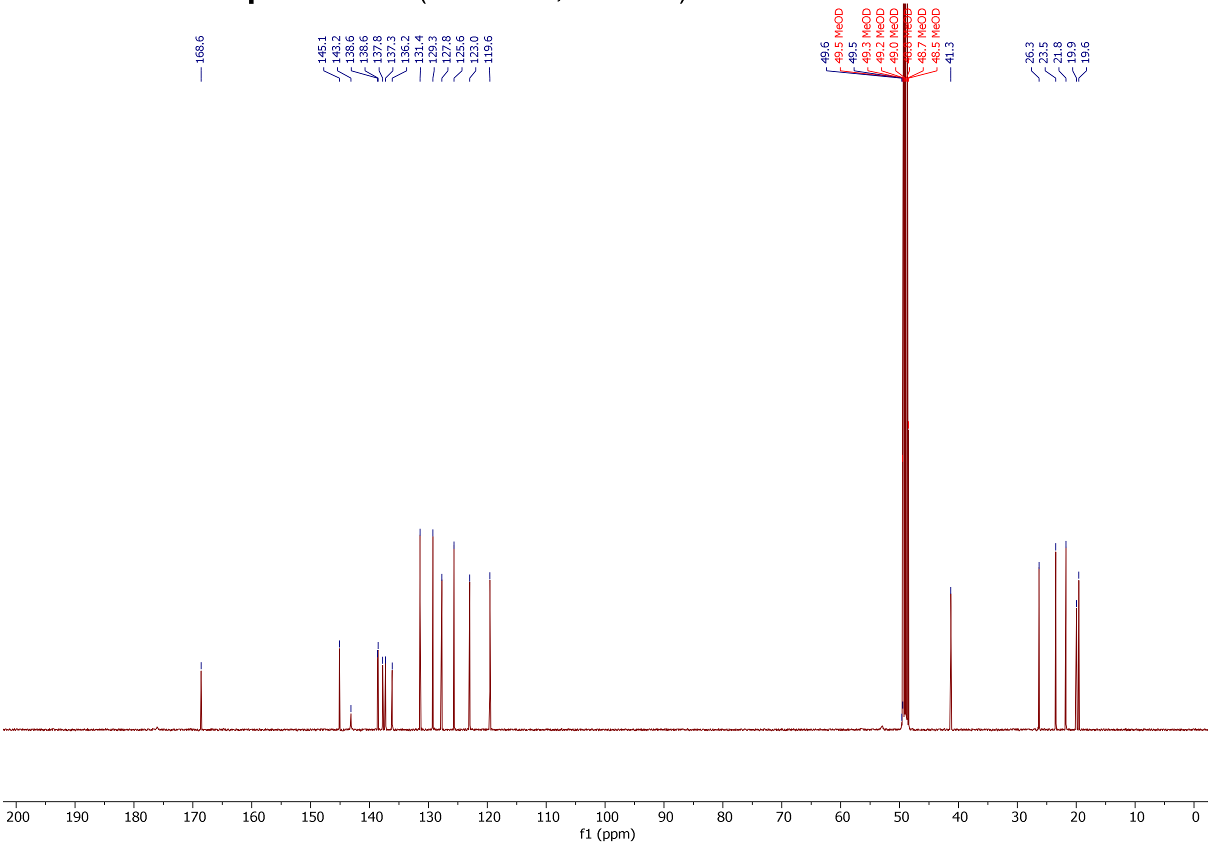

^{1}H NMR of **Compound 16a** (500 MHz, CD_3OD)

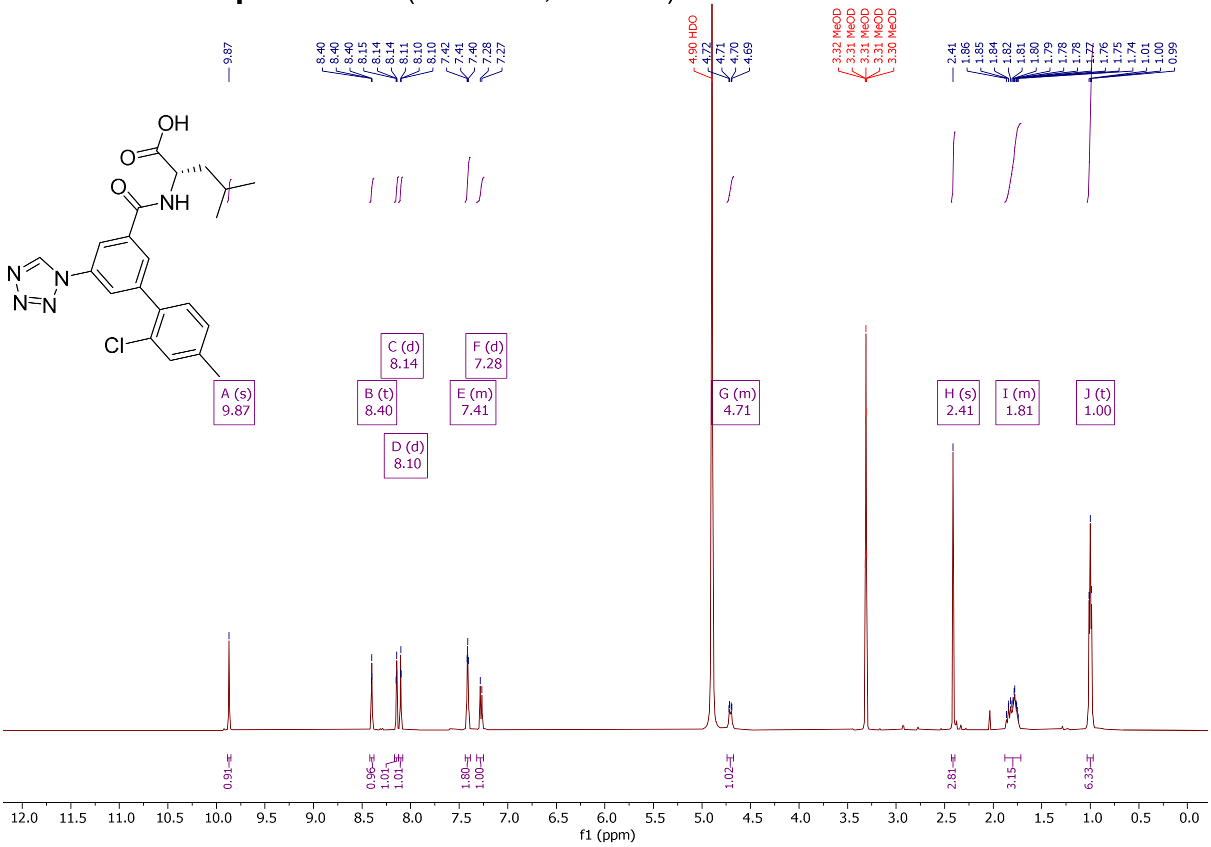

^{13}C NMR of **Compound 16a** (125 MHz, CD_3OD)

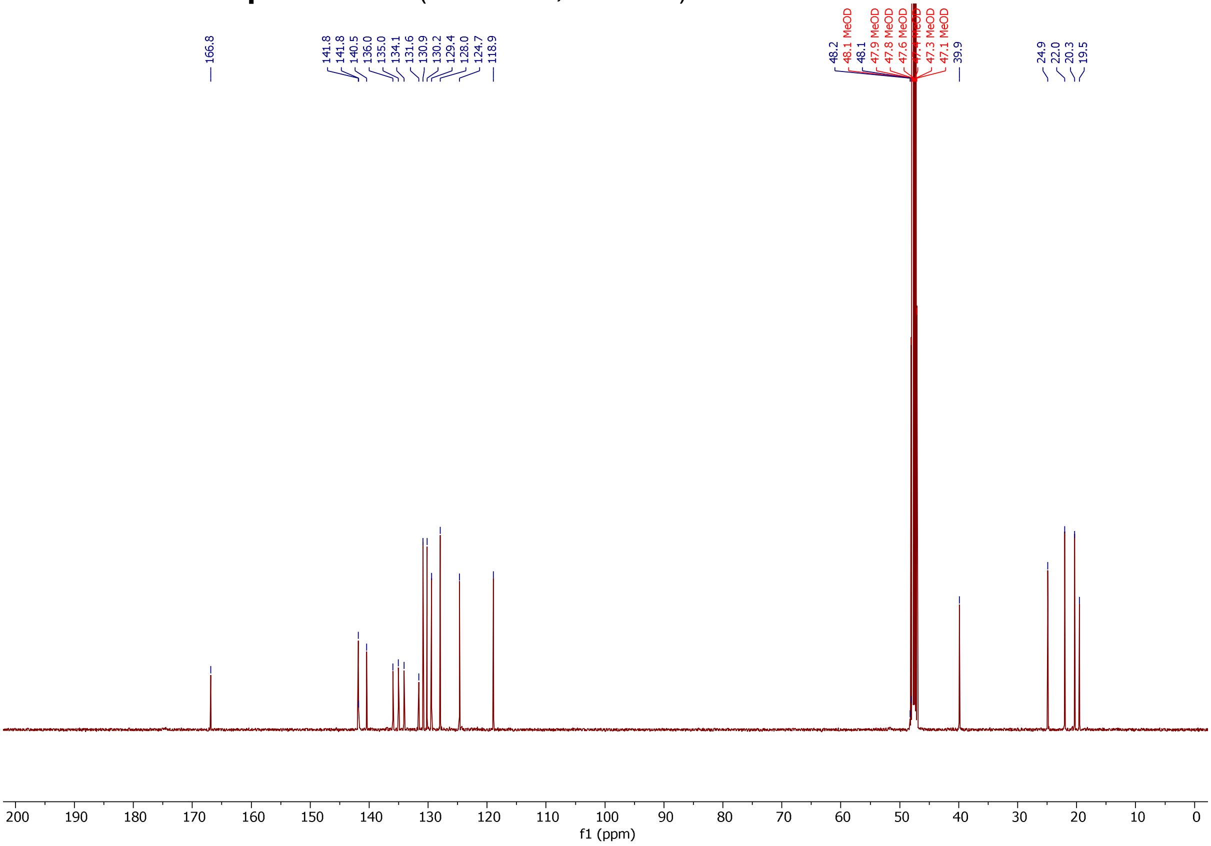

^{1}H NMR of **Compound 17a** (500 MHz, CD_3OD)

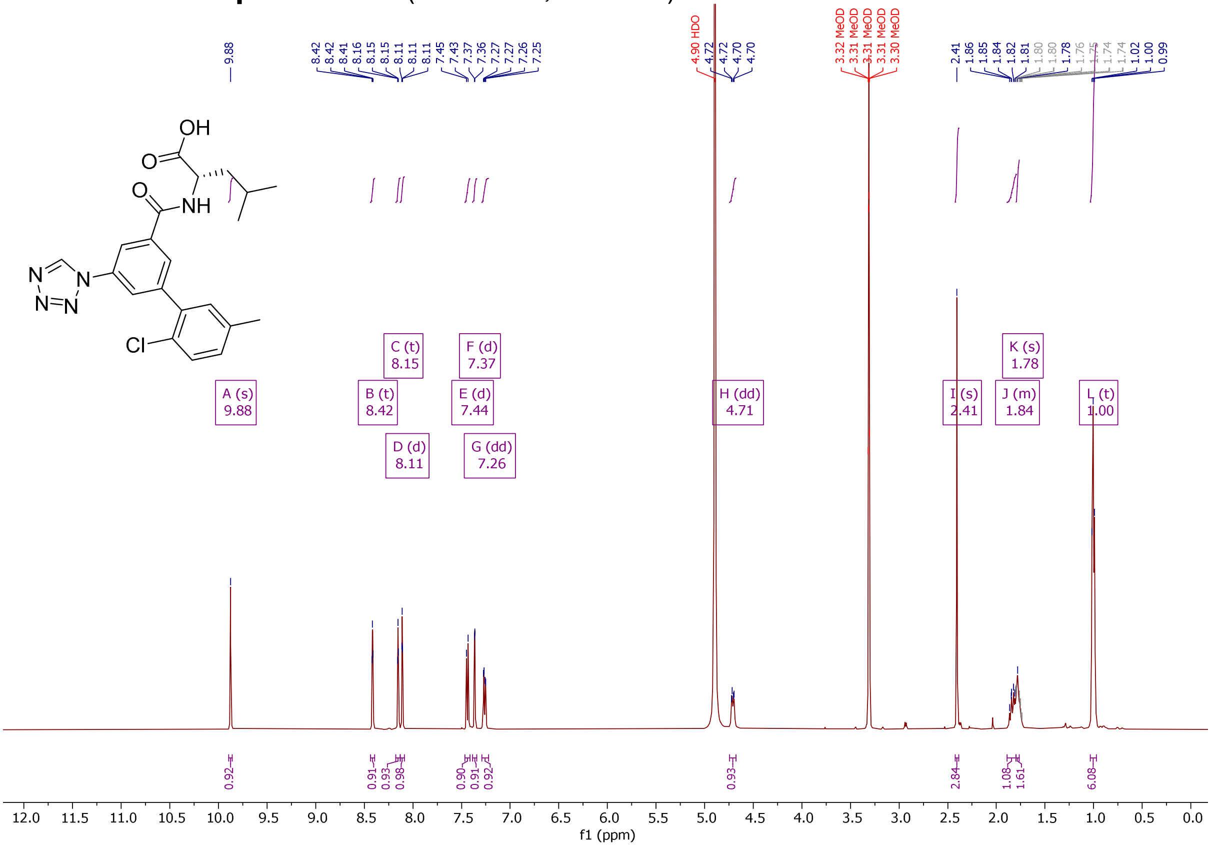

^{13}C NMR of **Compound 17a** (125 MHz, CD_3OD)

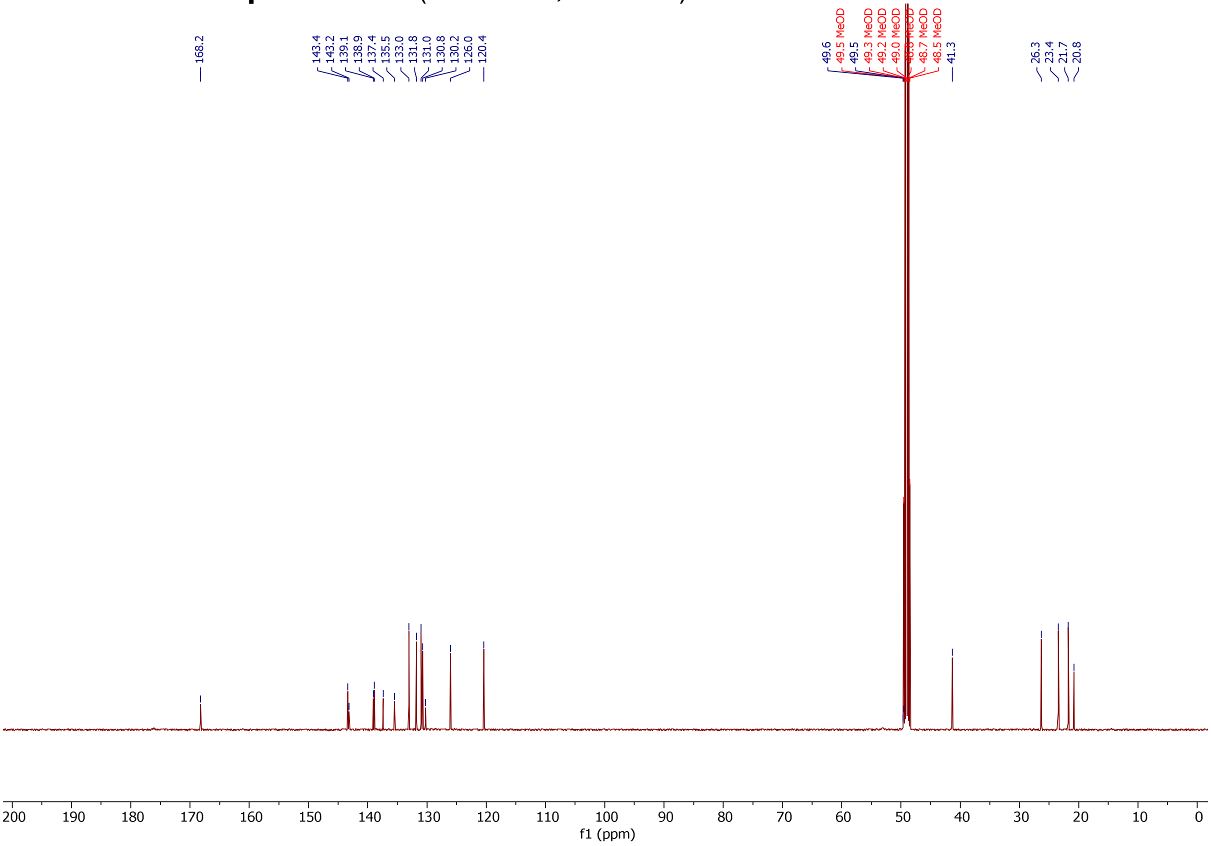

^{1}H NMR of **Compound 18a** (500 MHz, CD3OD)

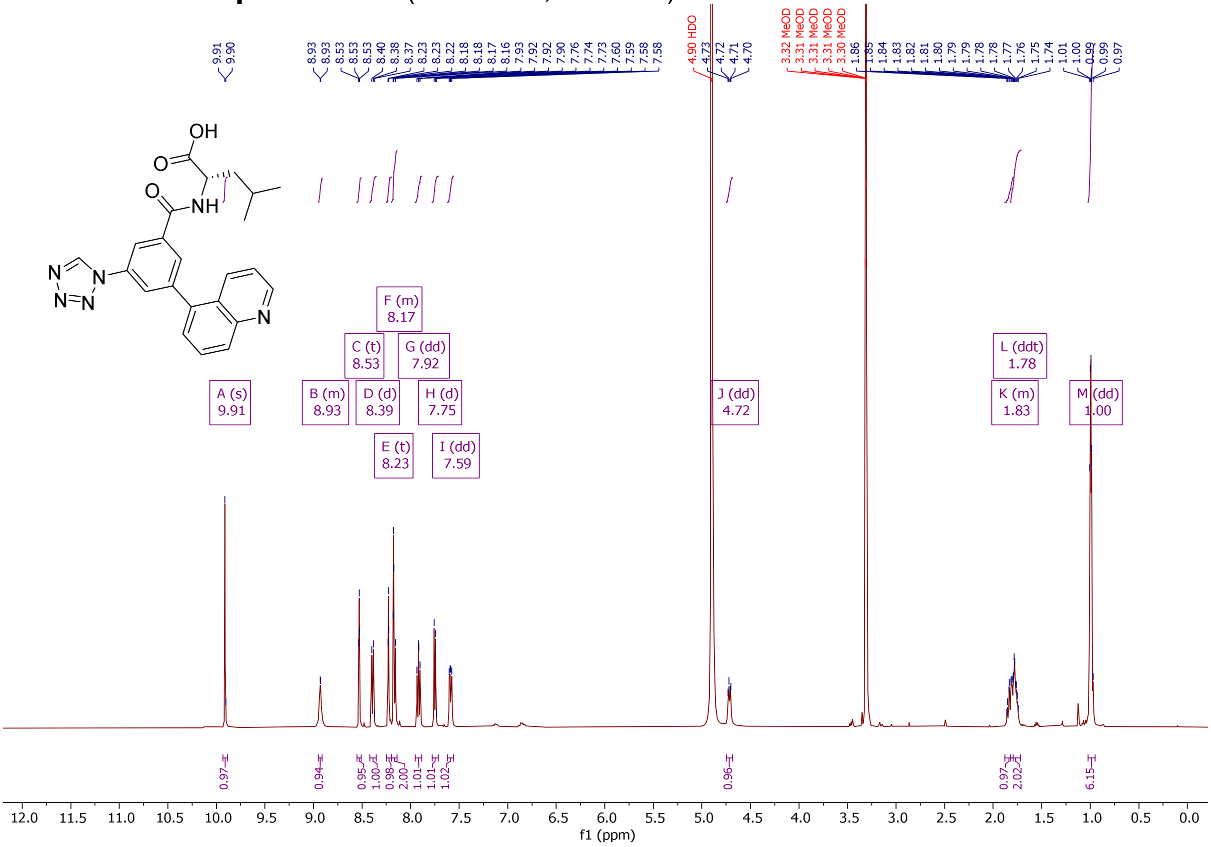

^{13}C NMR of **Compound 18a** (125 MHz, CD3OD)

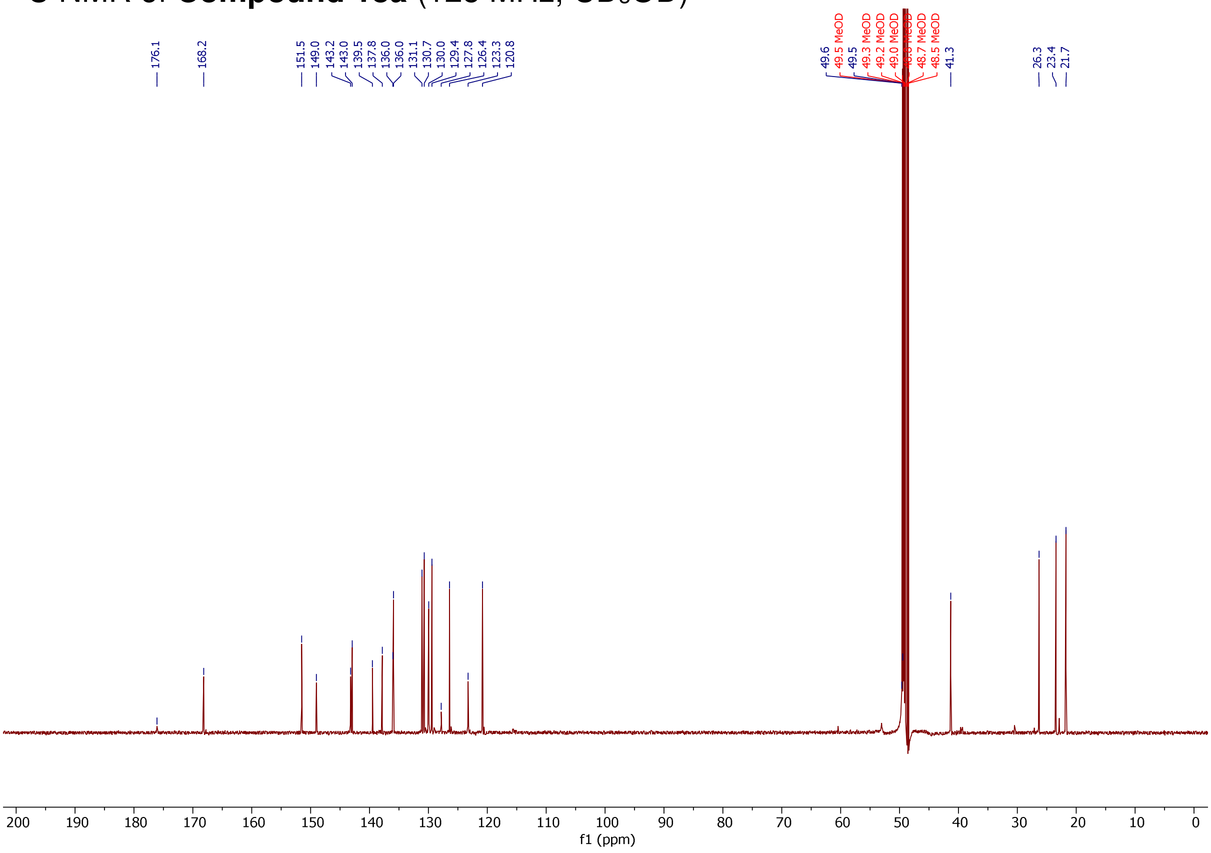

^{1}H NMR of **Compound 19a** (600 MHz, CD_3OD)

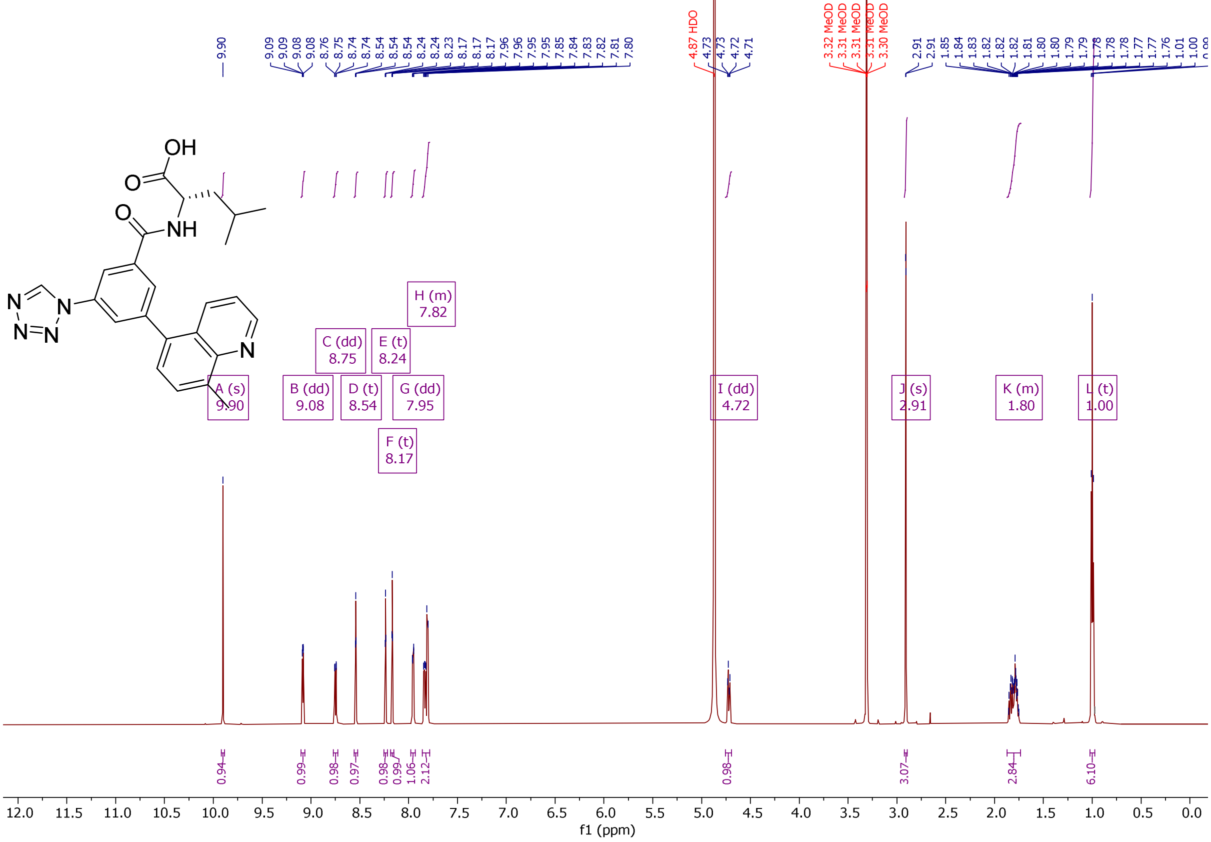

^{13}C NMR of **Compound 19a** (150 MHz, CD_3OD)

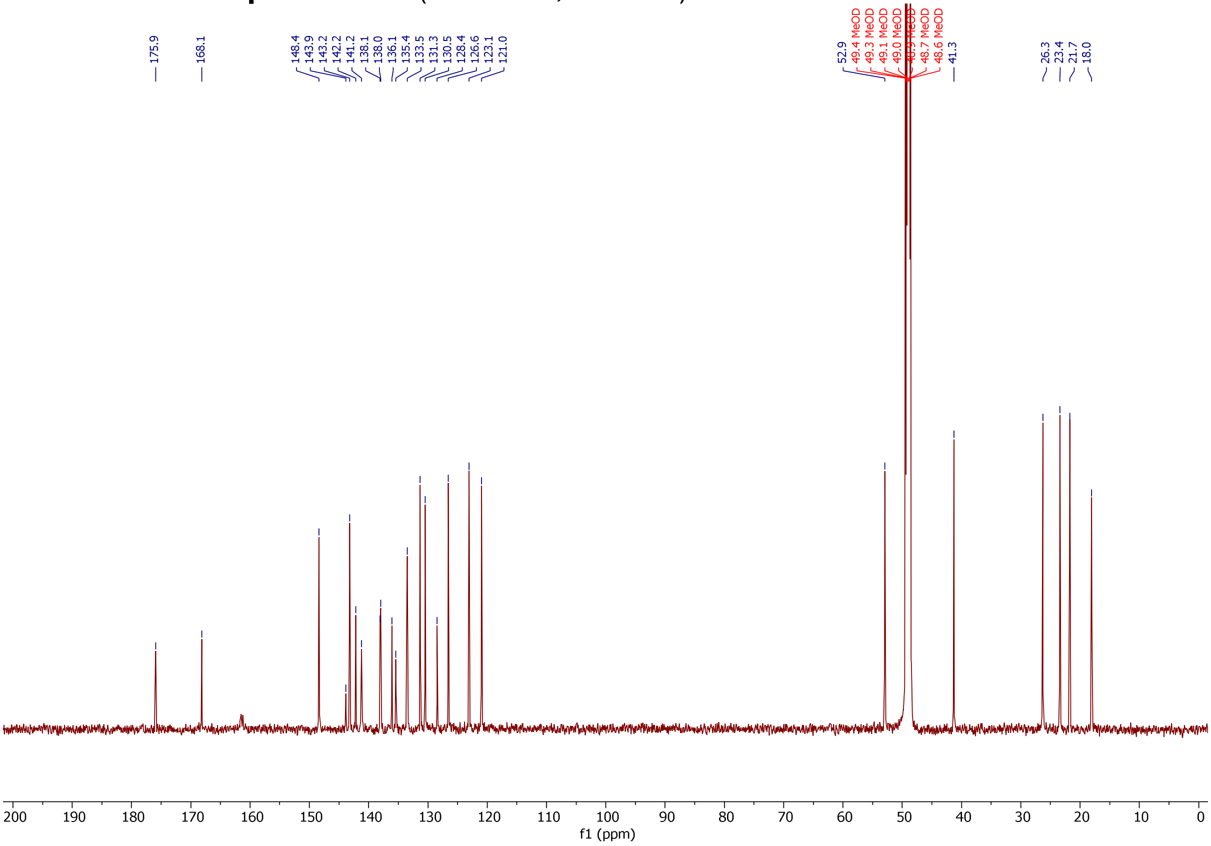

^{1}H NMR of **Compound 20a** (600 MHz, CD_3OD)

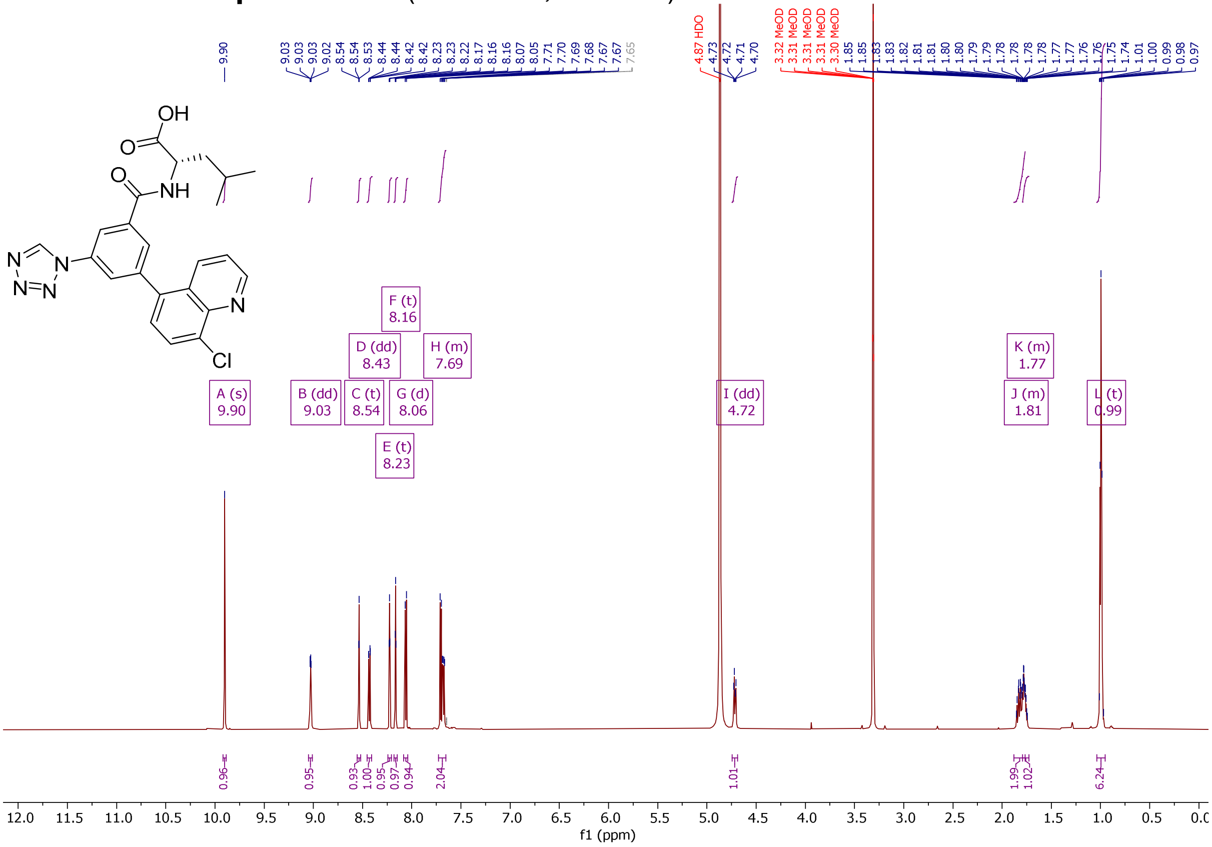

^{13}C NMR of **Compound 20a** (150 MHz, CD_3OD)

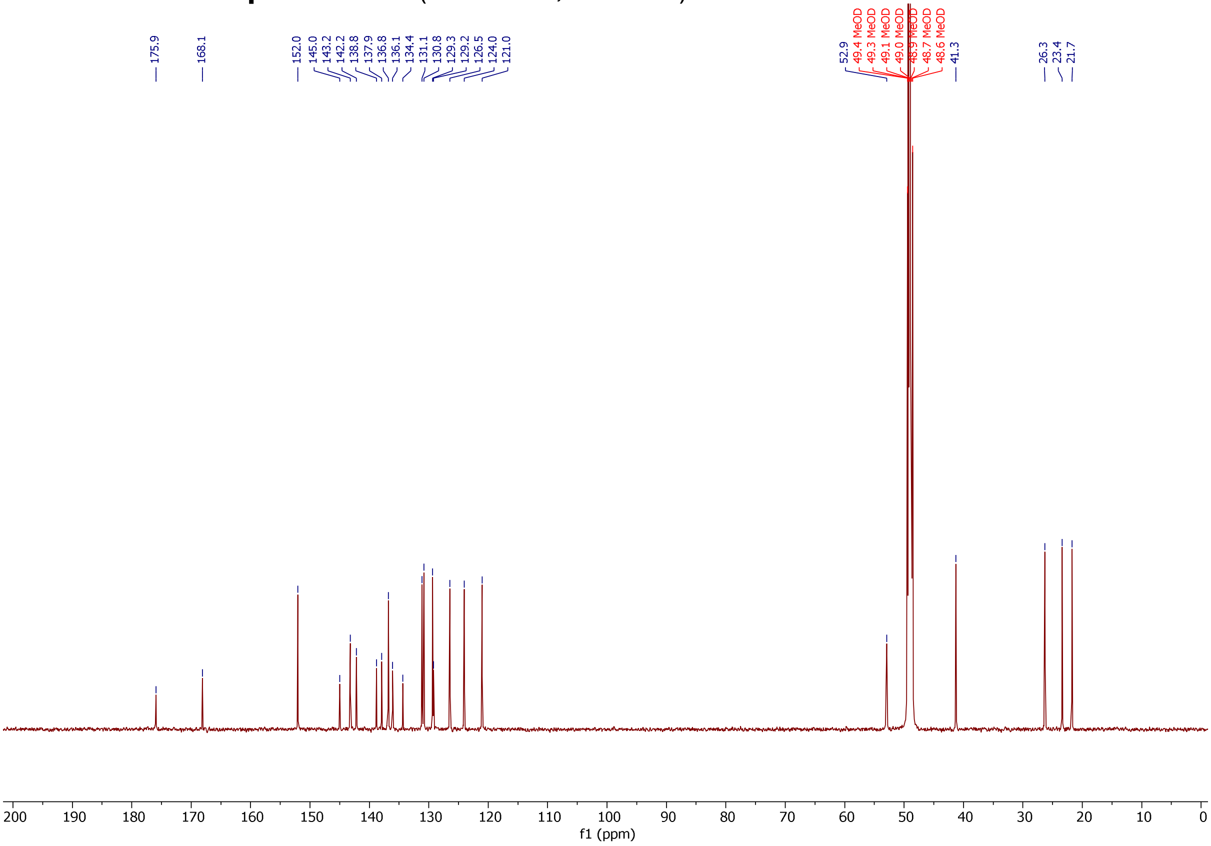

^{1}H NMR of **Compound 21a** (500 MHz, CD_3OD)

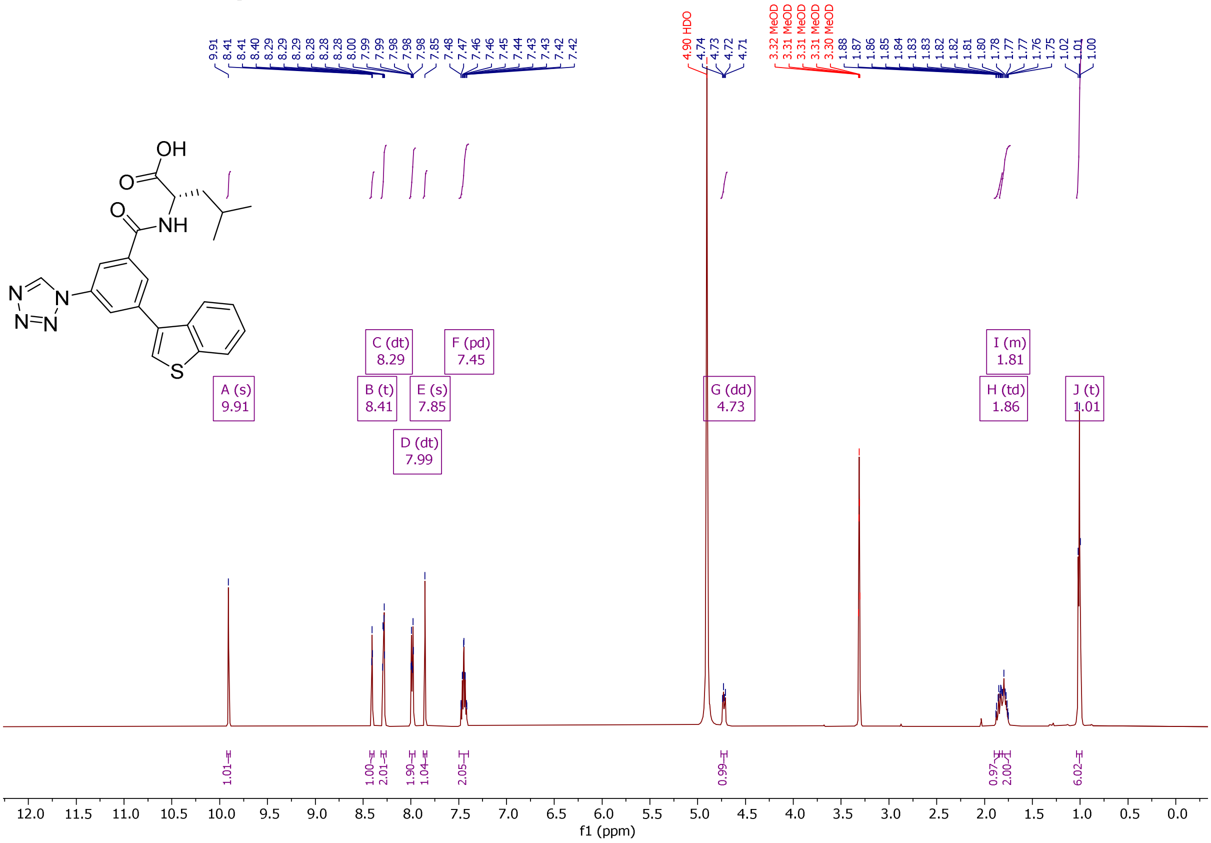

^{13}C NMR of **Compound 21a** (125 MHz, CD_3OD)

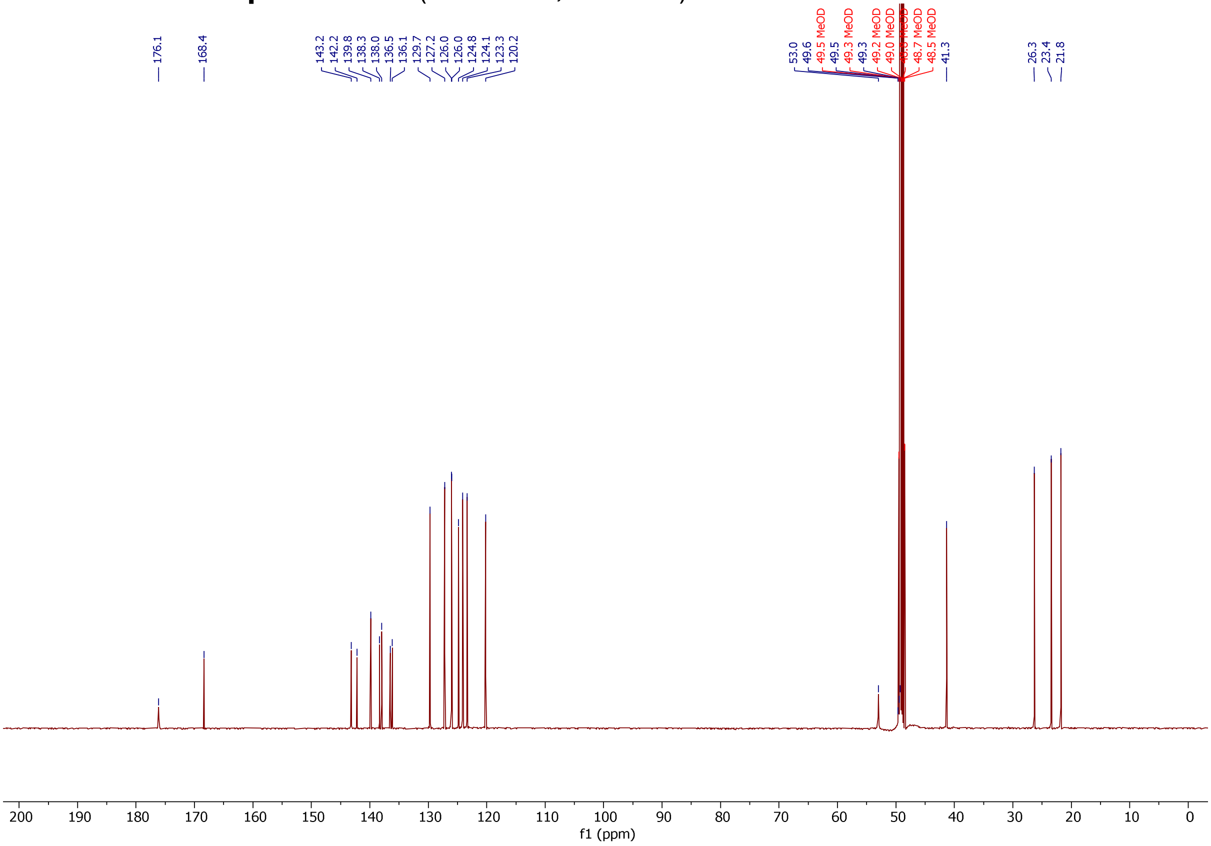

^{1}H NMR of **Compound 22a** (500 MHz, CD_3OD)

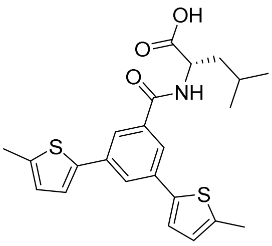

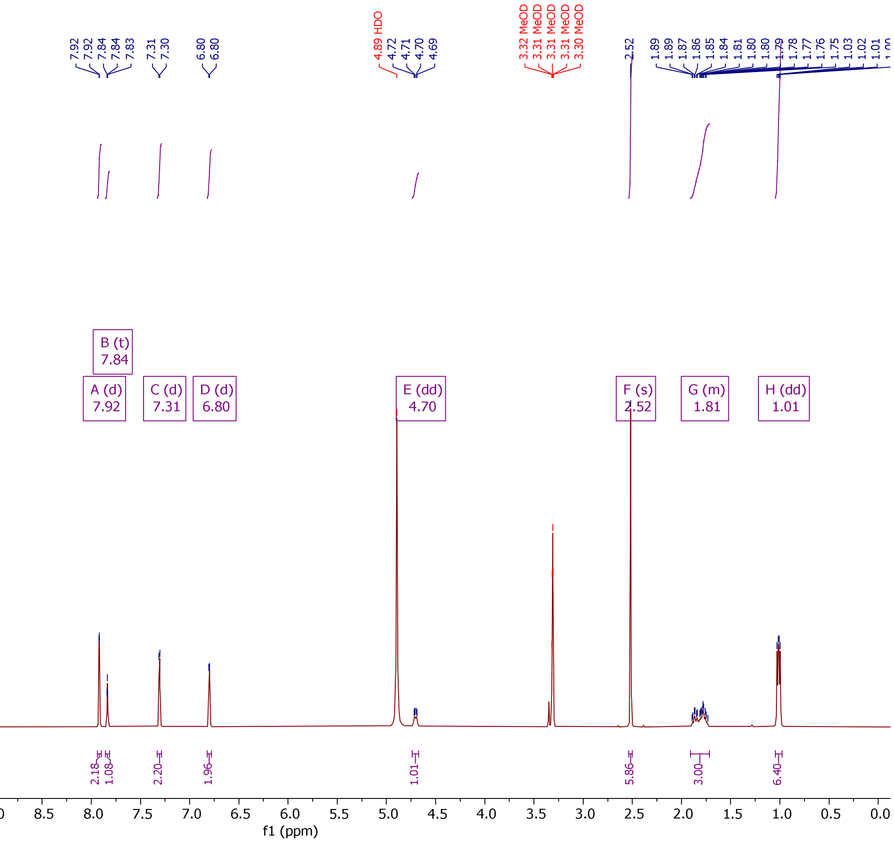

^{13}C NMR of **Compound 22a** (125 MHz, CD_3OD)

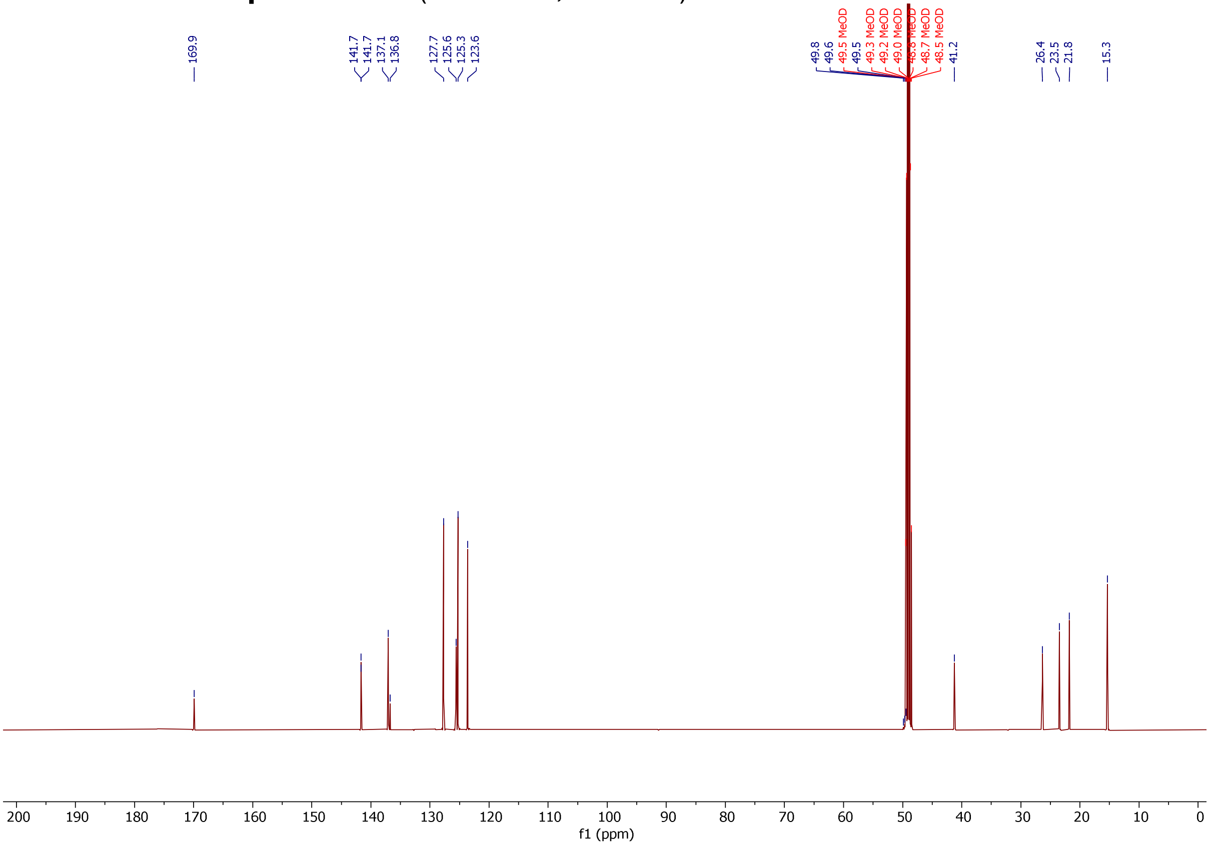

^{1}H NMR of **Compound 23a** (500 MHz, CD_3OD)

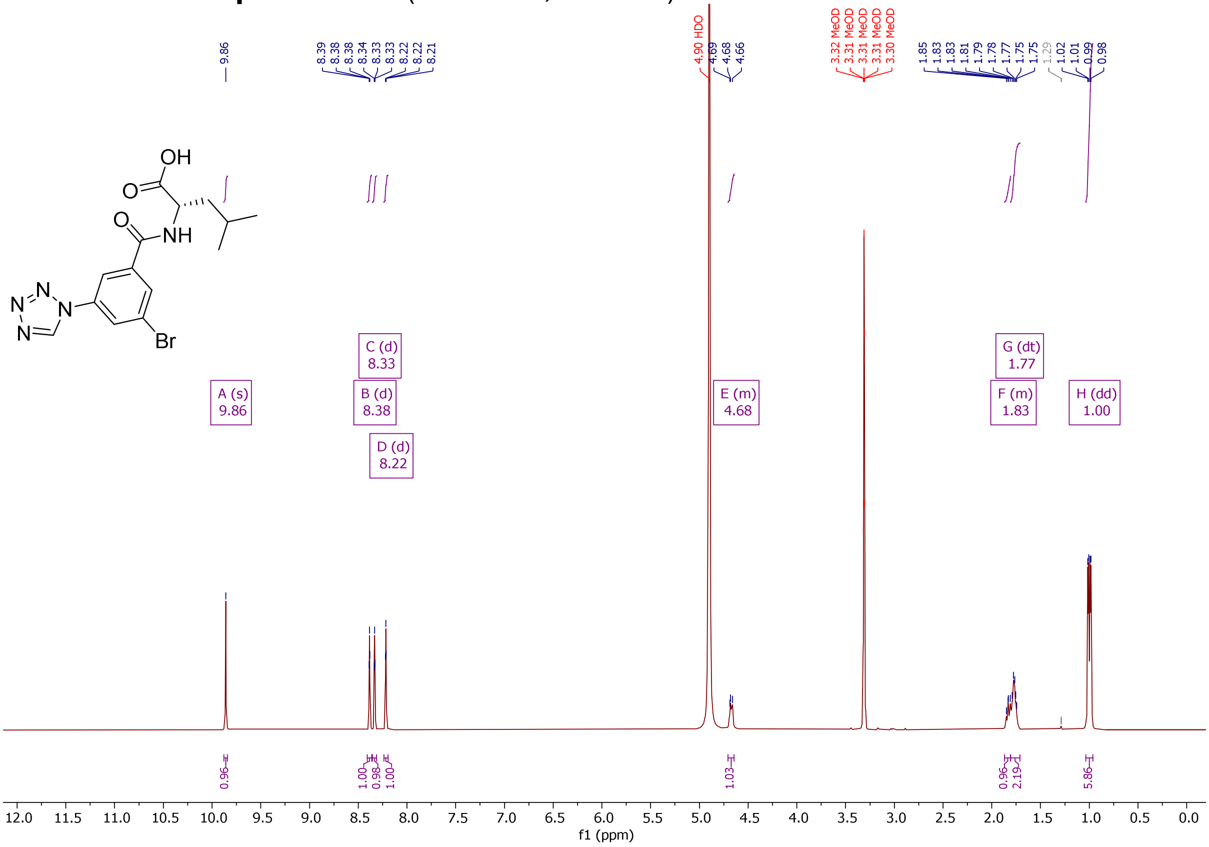

^{13}C NMR of **Compound 23a** (125 MHz, CD_3OD)

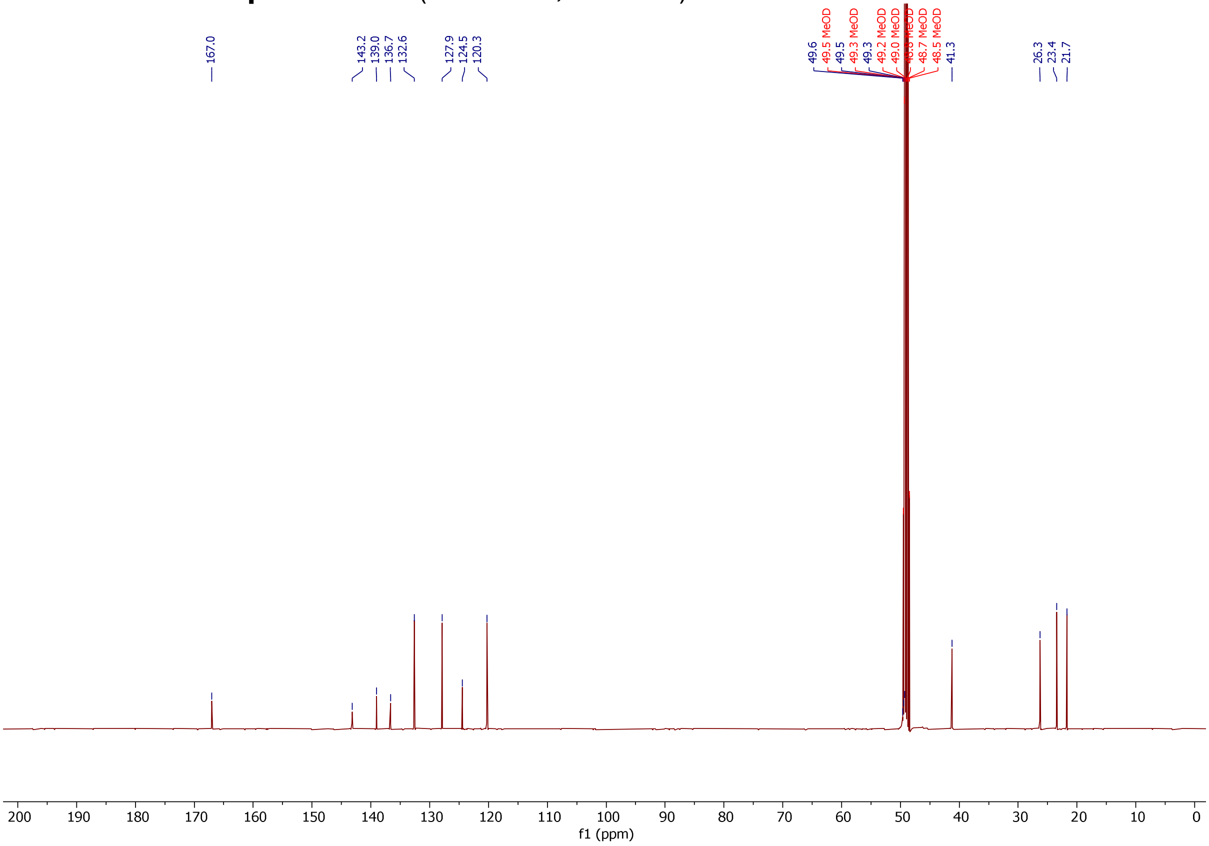

^{1}H NMR of **Compound 24a** (500 MHz, CD_3OD)

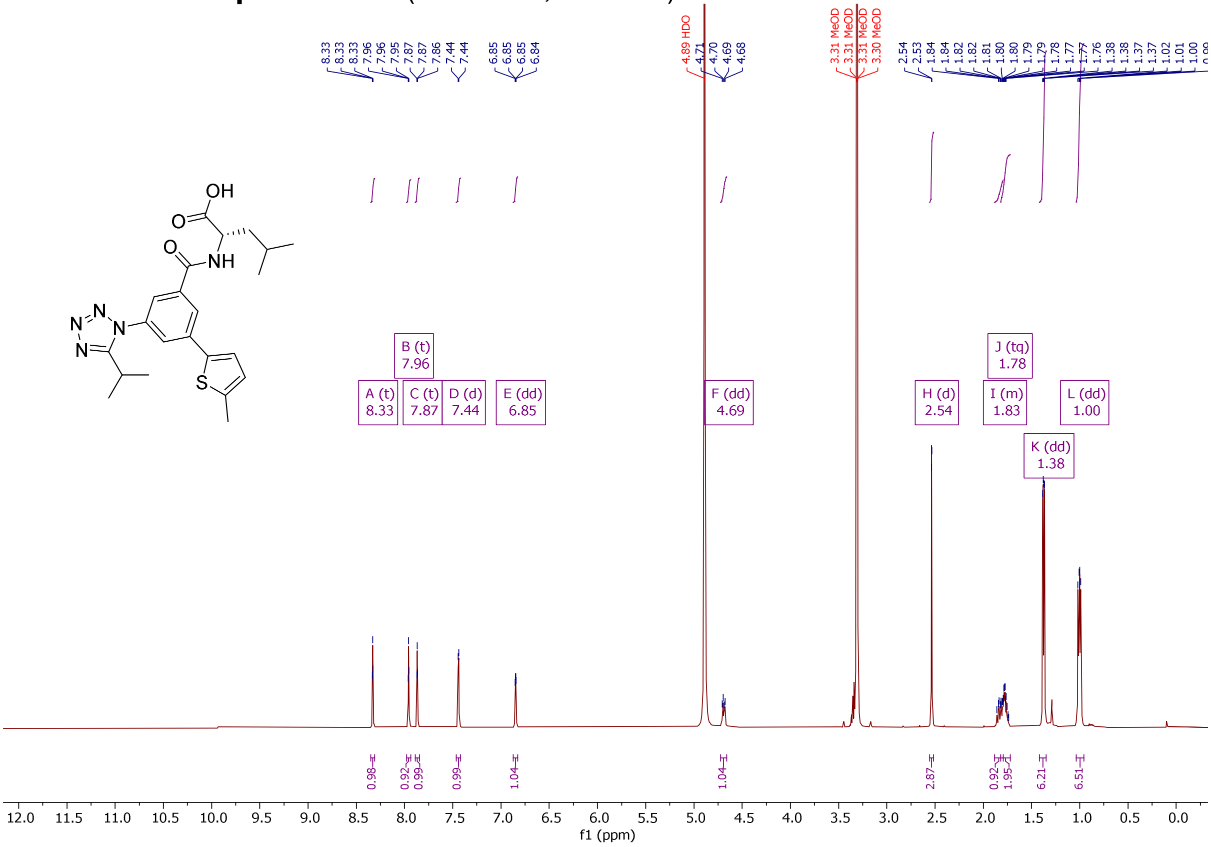

^{13}C NMR of **Compound 24a** (125 MHz, CD_3OD)

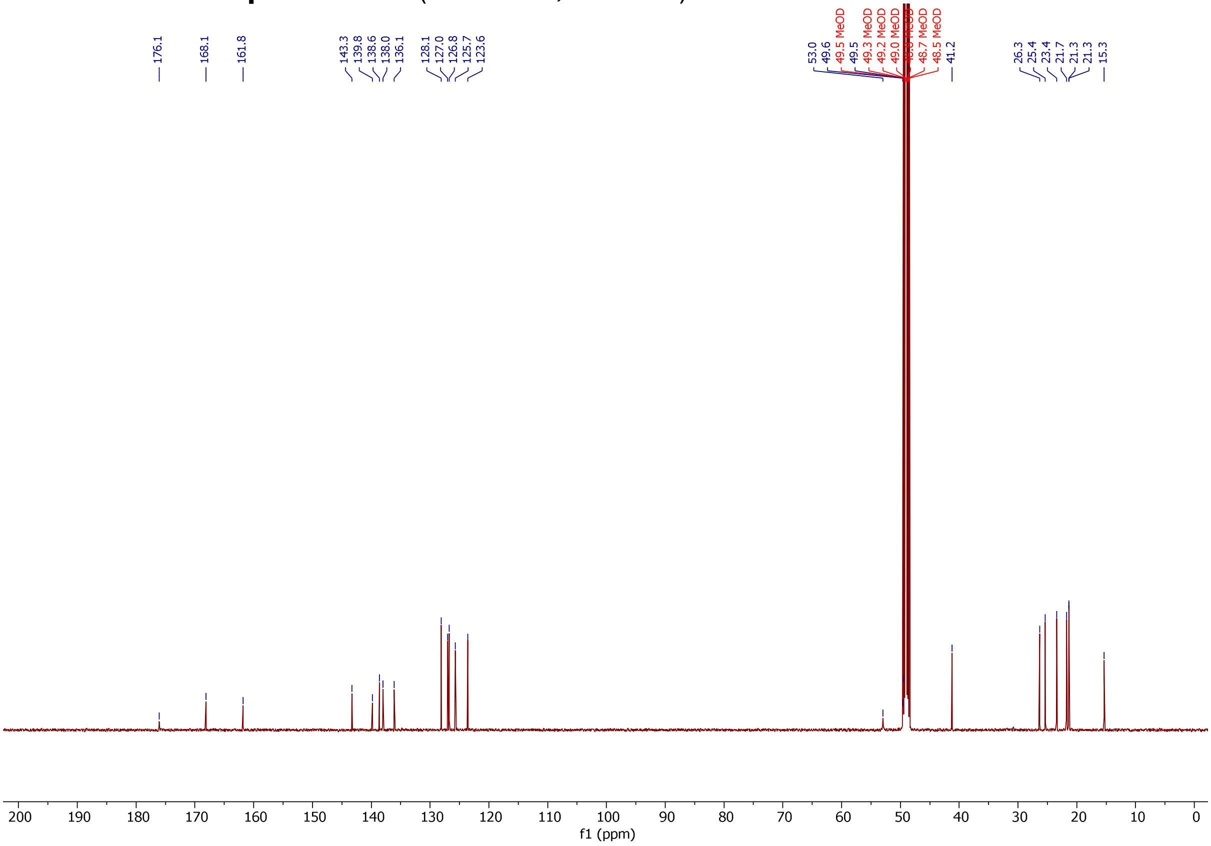

^{1}H NMR of **Compound 25a** (500 MHz, CD_3OD)

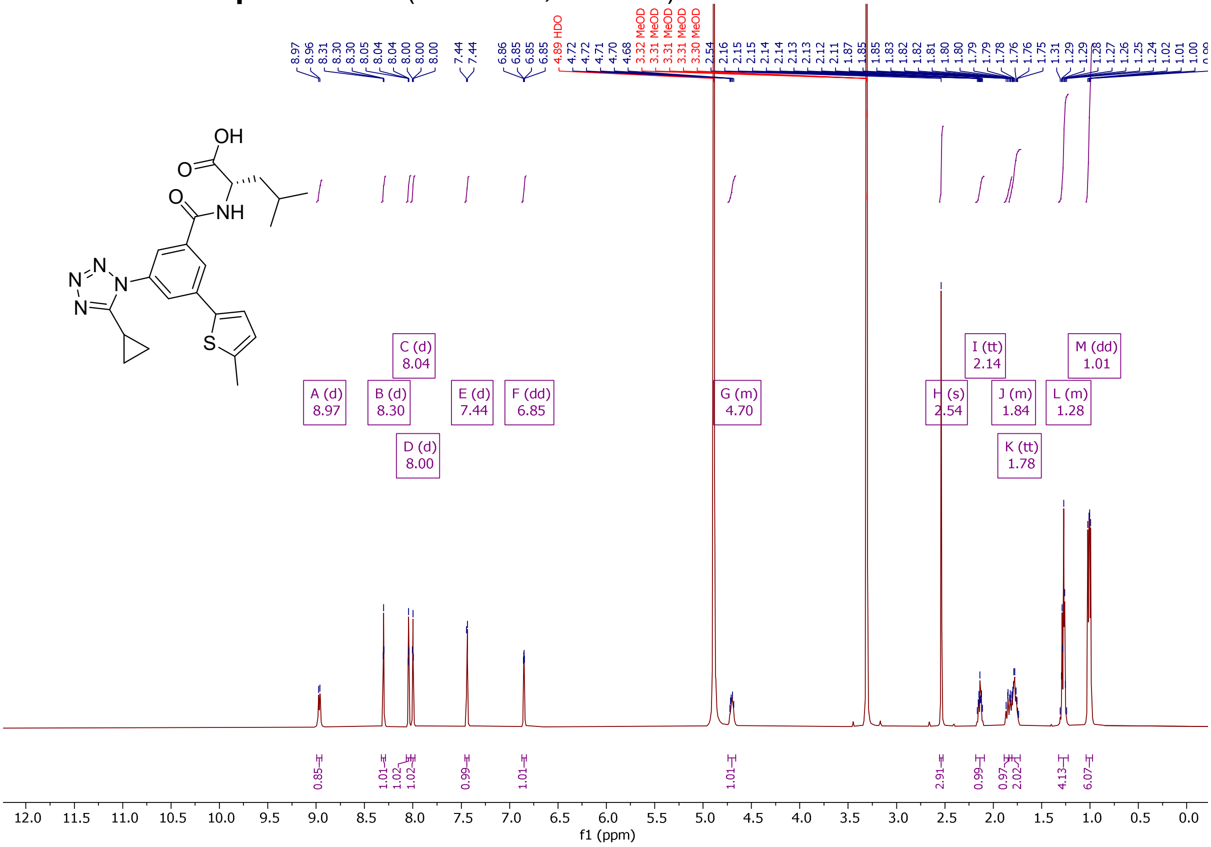

^{13}C NMR of **Compound 25a** (125 MHz, CD_3OD)

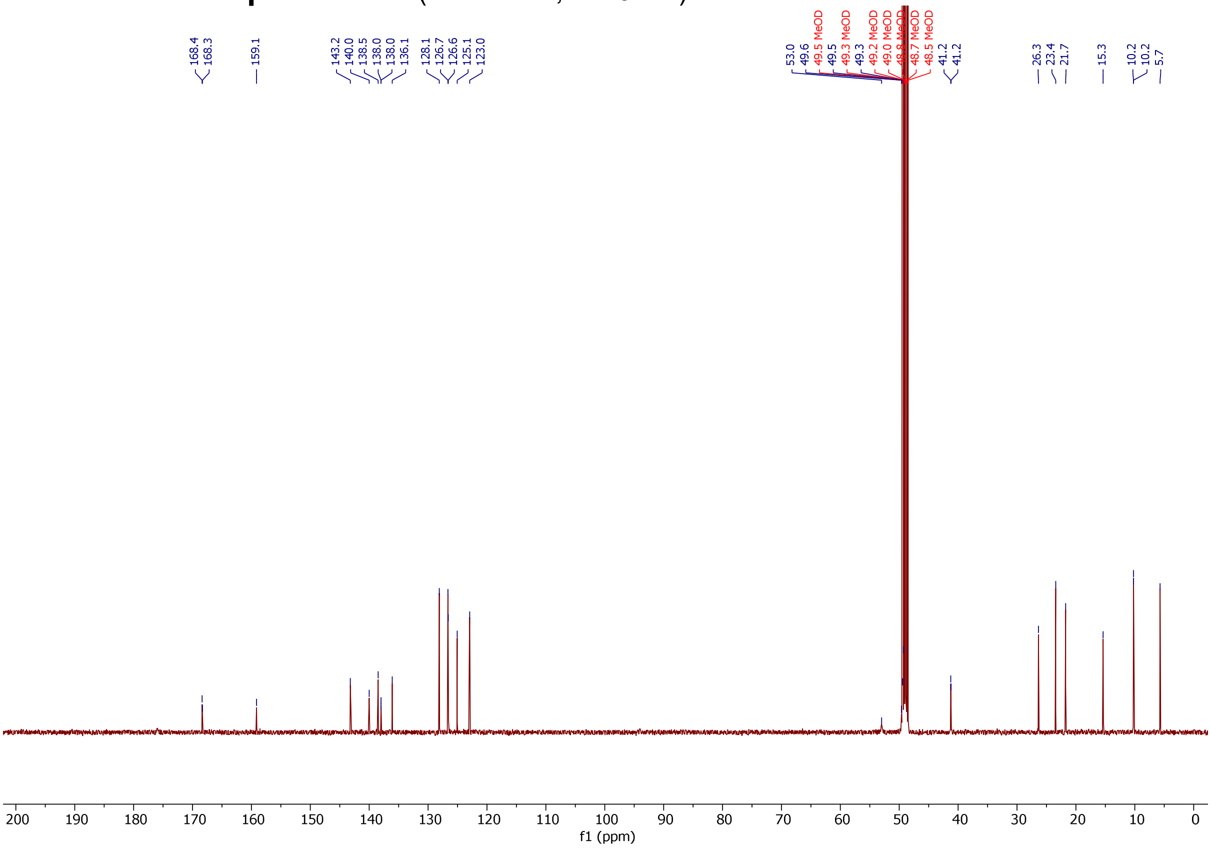

^{1}H NMR of **Compound 26a** (500 MHz, CD_3OD)

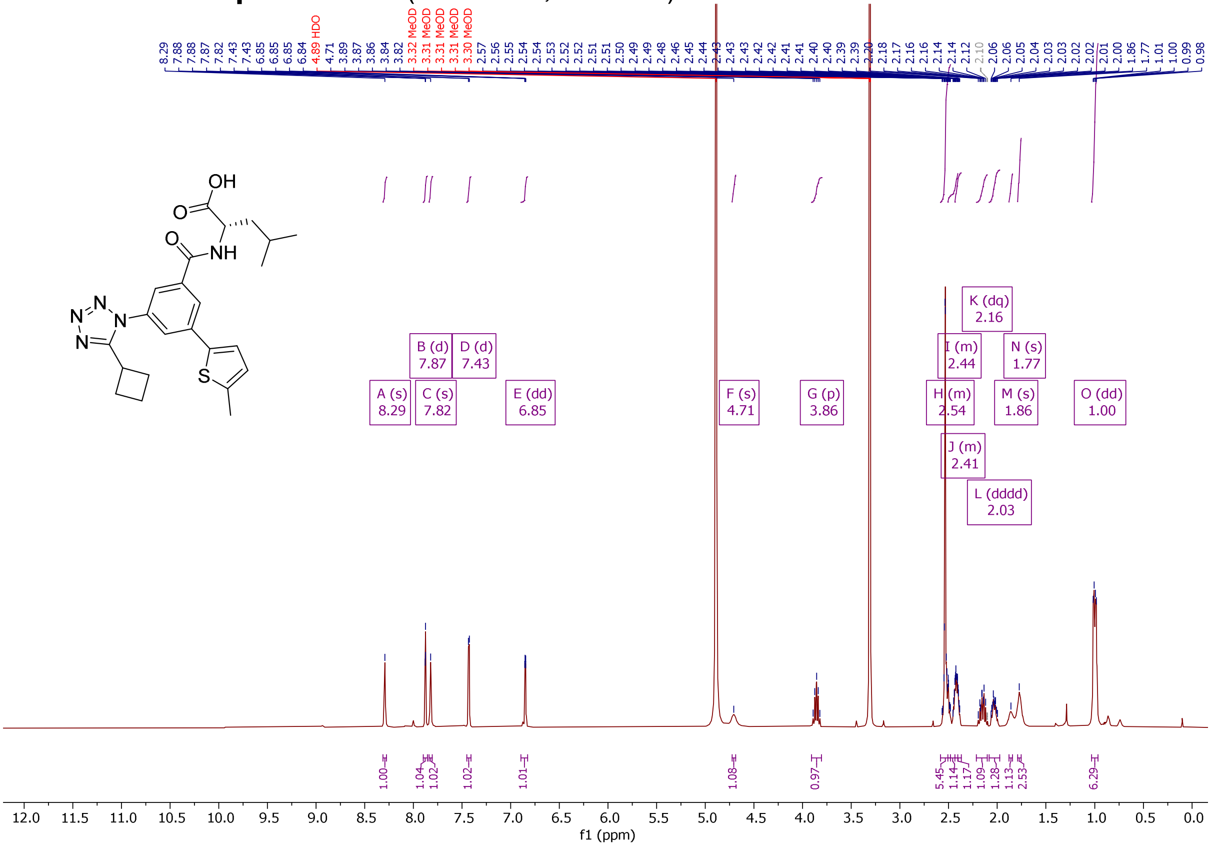

^{13}C NMR of **Compound 26a** (125 MHz, CD_3OD)

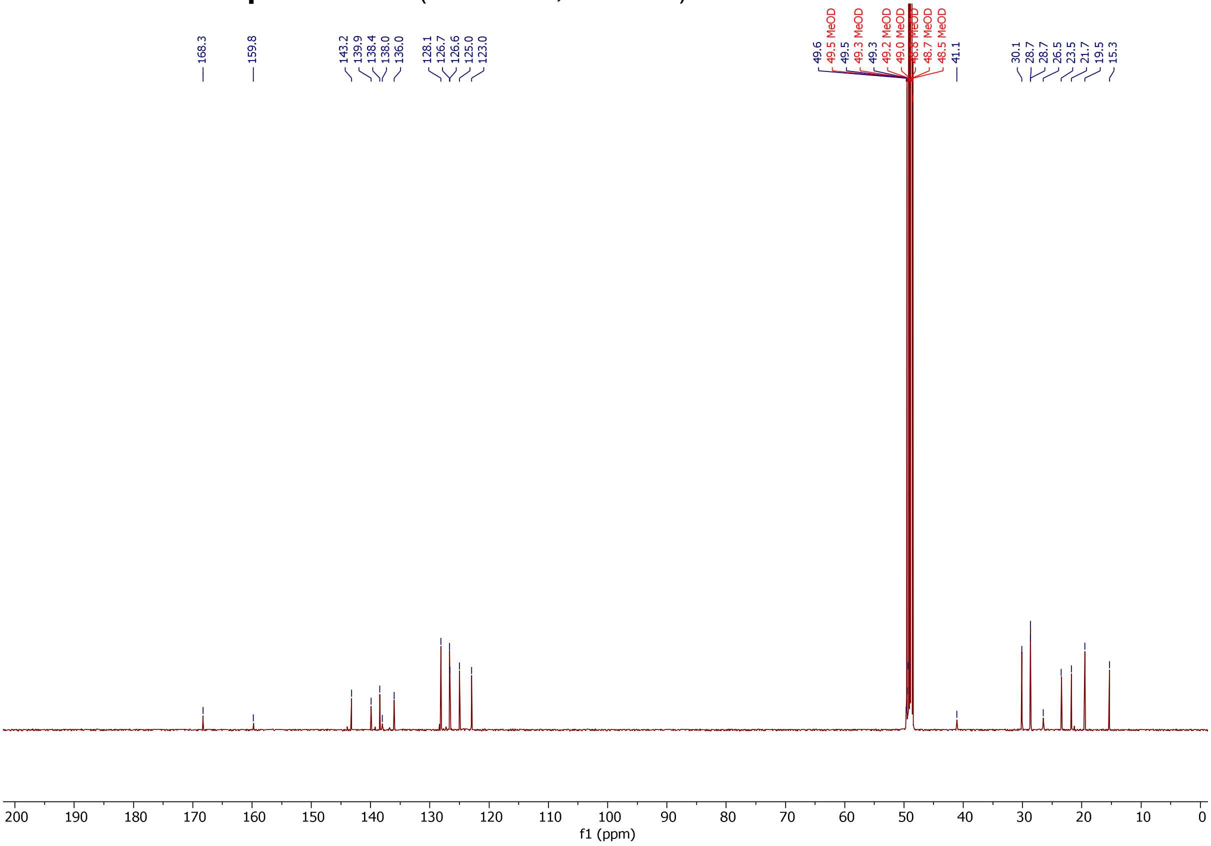

^{1}H NMR of **Compound 27a** (500 MHz, CD_3OD)

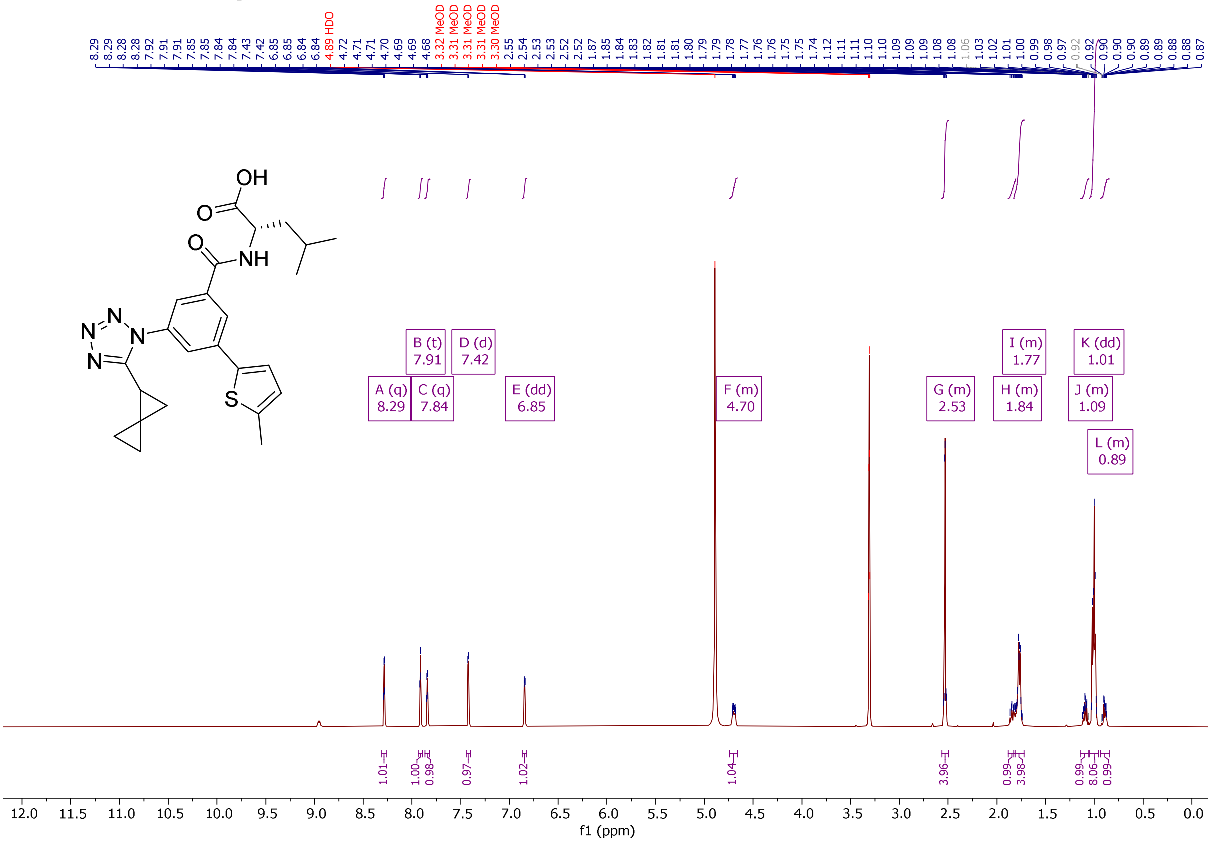

^{13}C NMR of **Compound 27a** (125 MHz, CD_3OD)

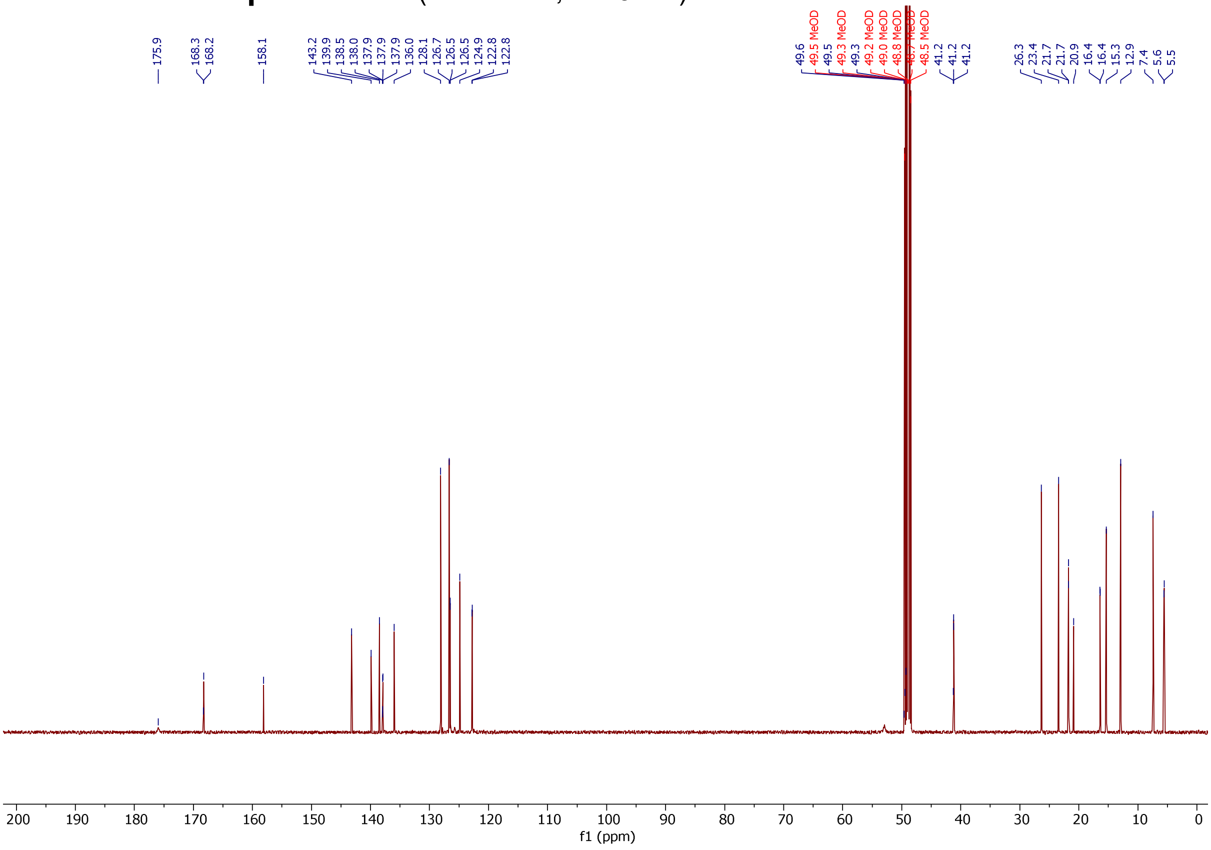

^{1}H NMR of **Compound 28a** (500 MHz, CD_3OD)

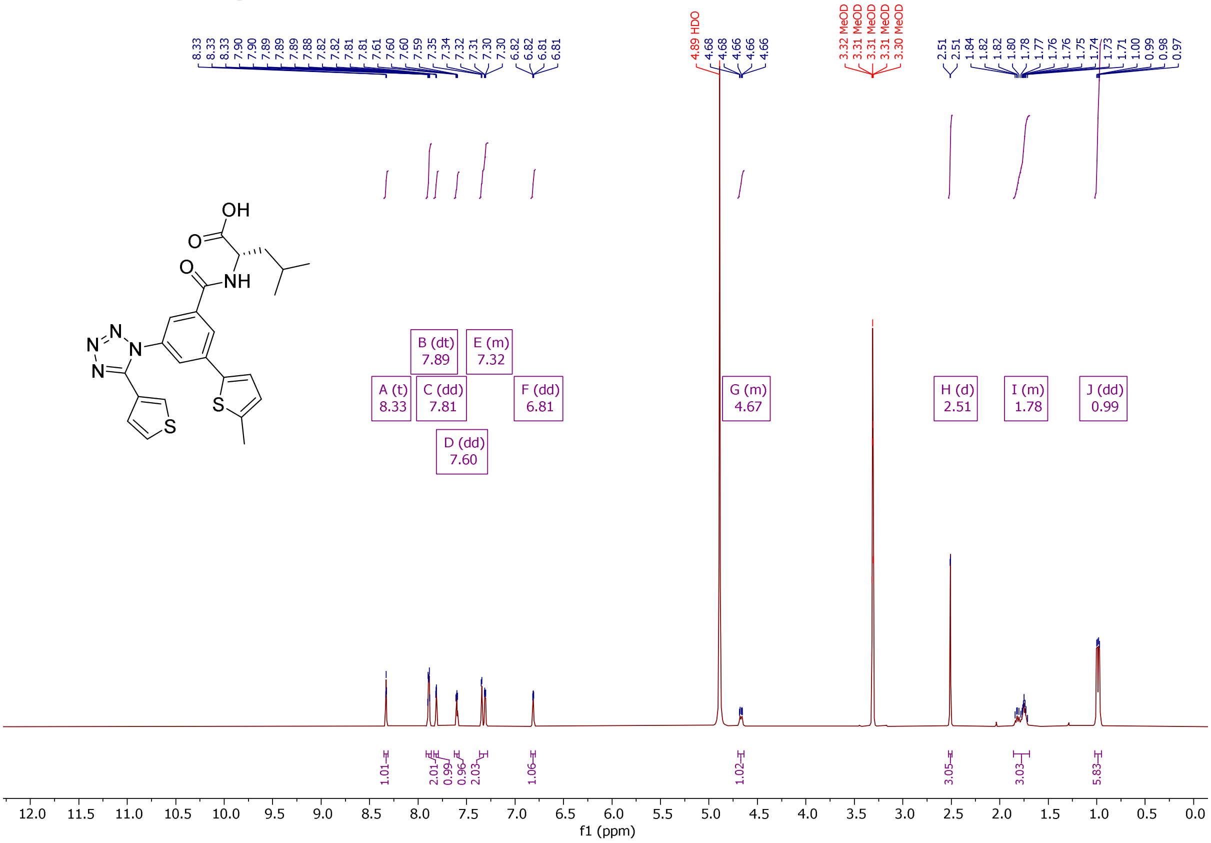

^{13}C NMR of **Compound 28a** (125 MHz, CD_3OD)

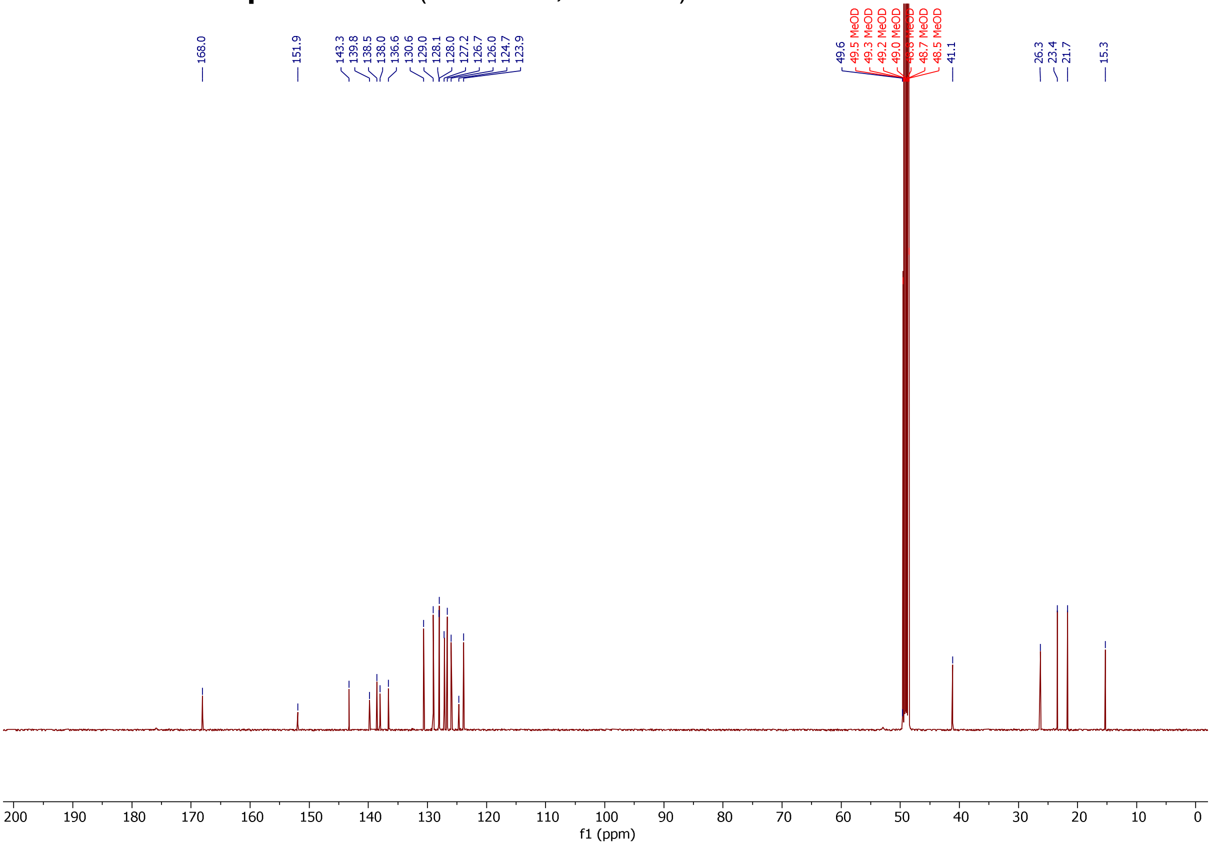

^{1}H NMR of **Compound 29a** (500 MHz, CD_3OD)

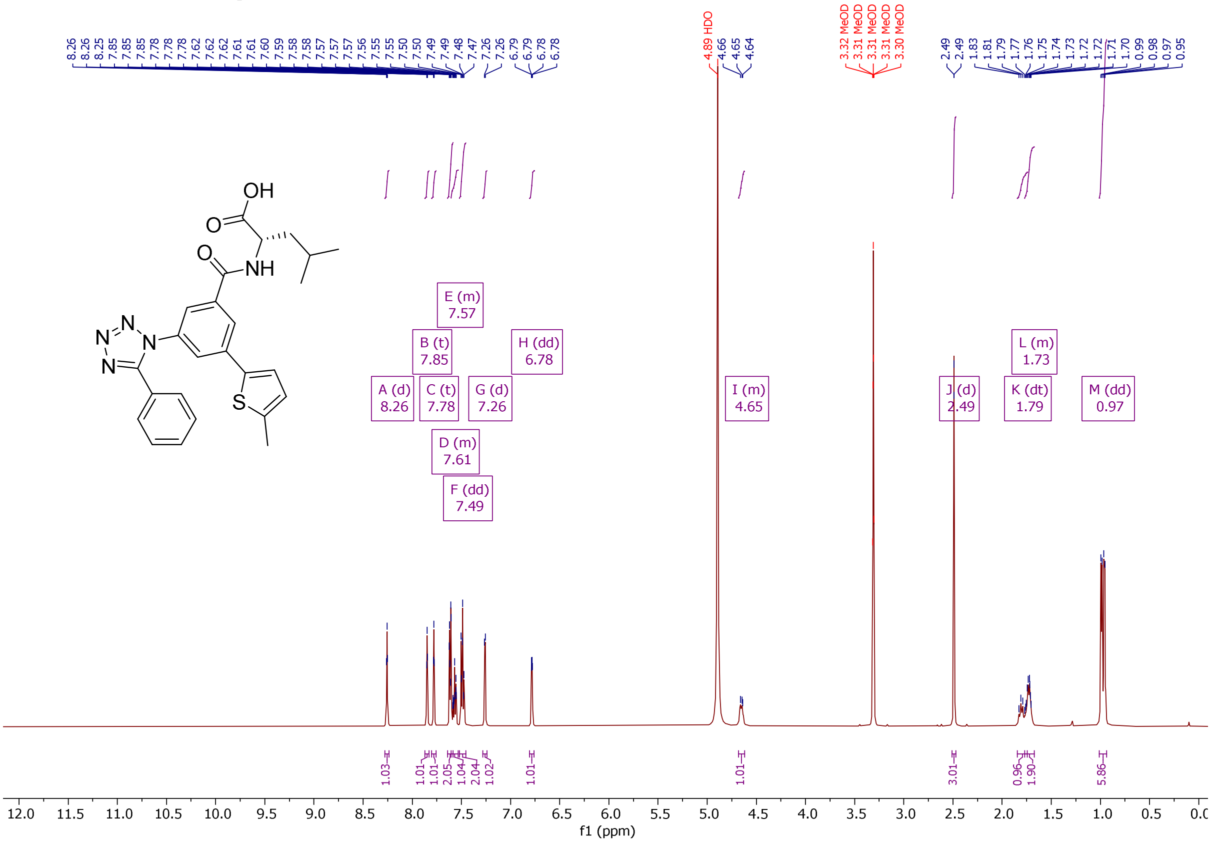

^{13}C NMR of **Compound 29a** (125 MHz, CD_3OD)

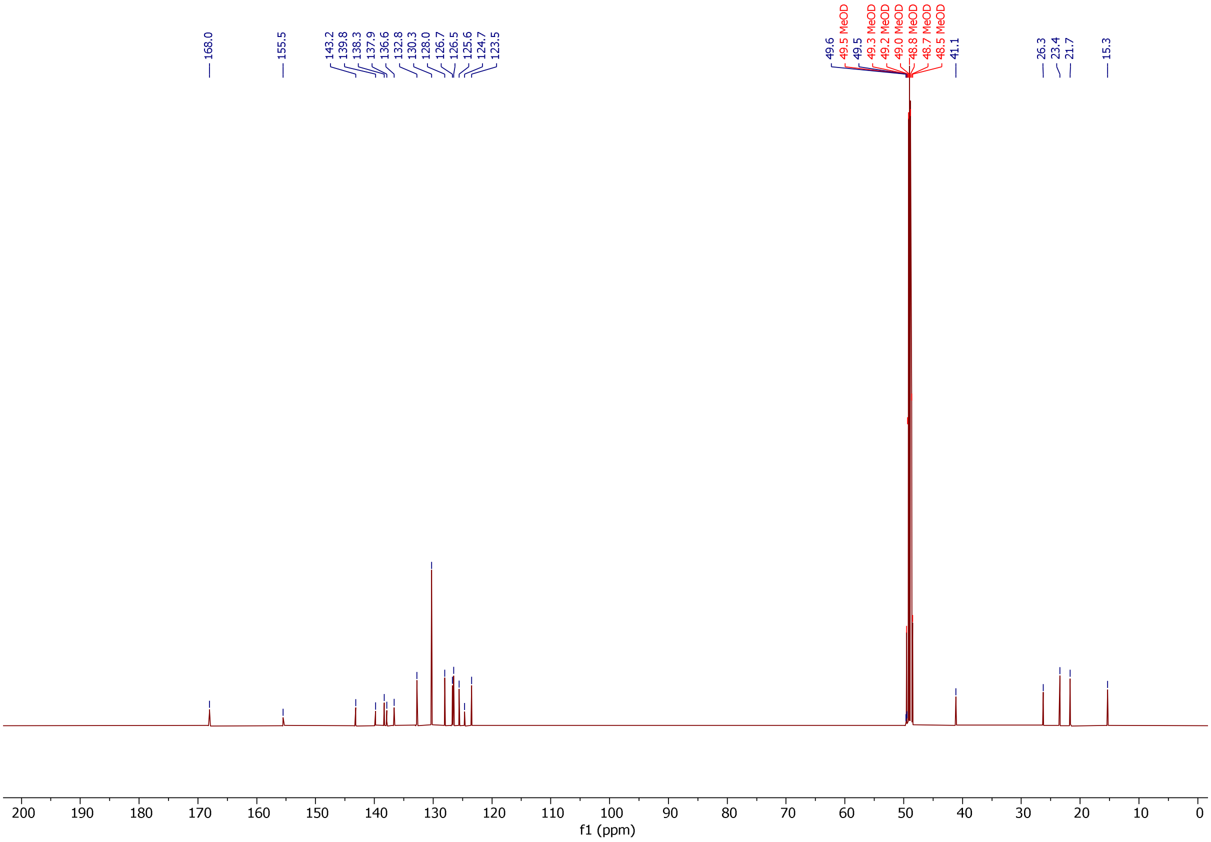

^{1}H NMR of **Compound 30a** (500 MHz, CD_3OD)

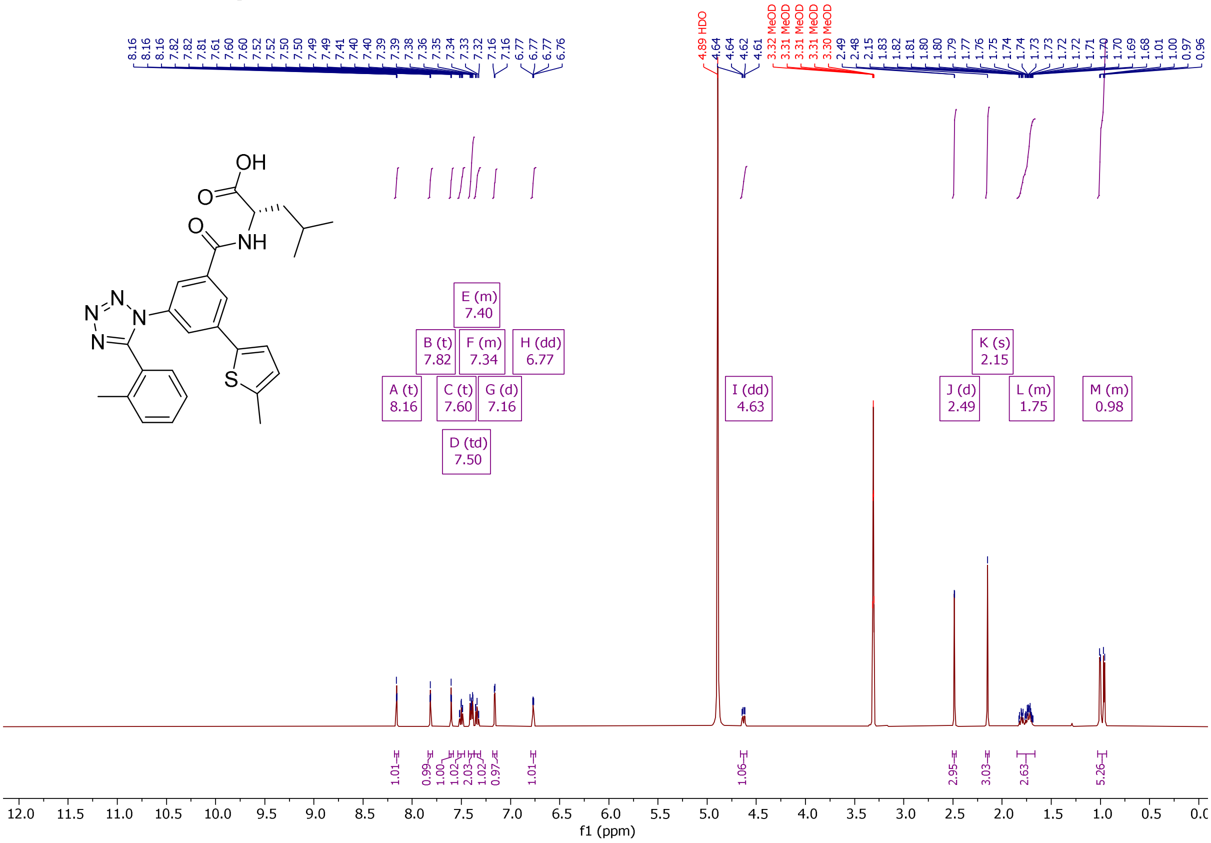

^{13}C NMR of **Compound 30a** (125 MHz, CD_3OD)

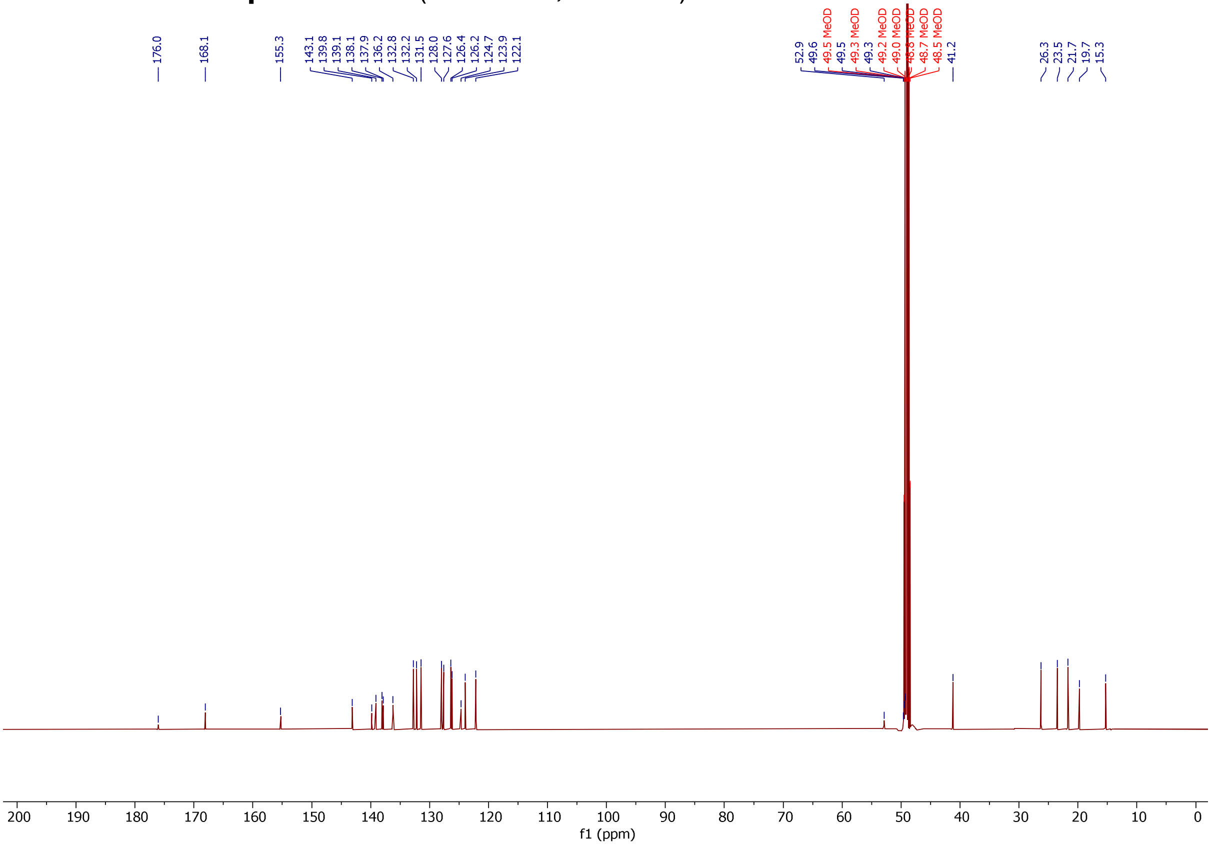

^{1}H NMR of **Compound 31a** (500 MHz, CD_3OD)

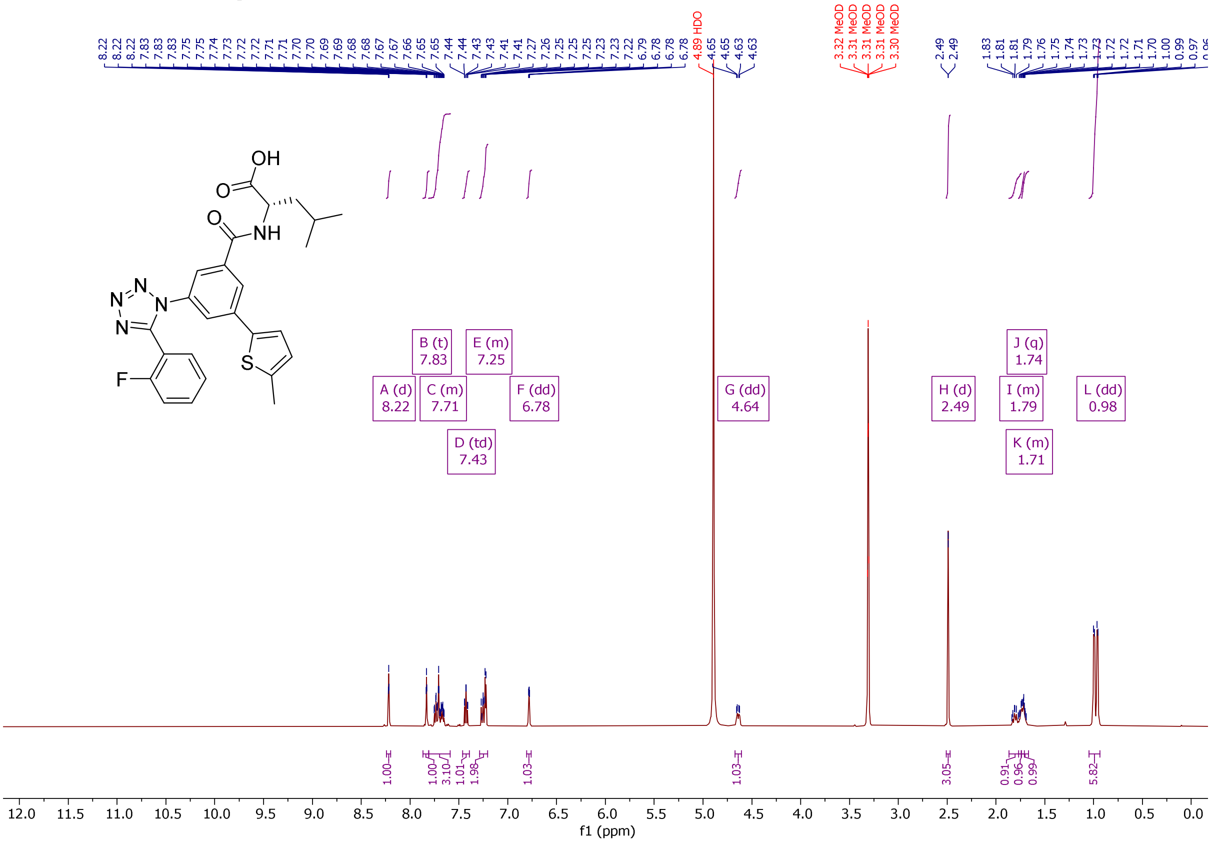

^{13}C NMR of **Compound 31a** (125 MHz, CD_3OD)

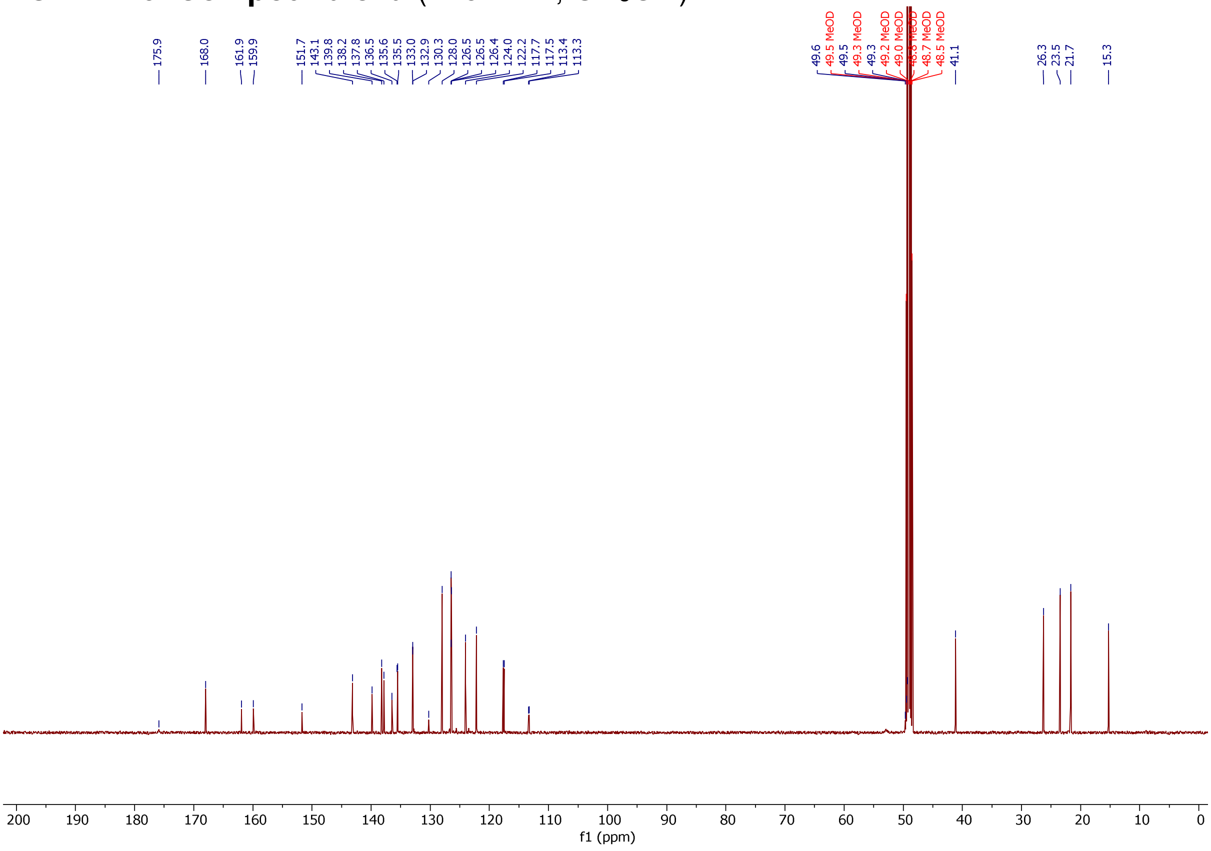

^{1}H NMR of **Compound 32a** (500 MHz, CD_3OD)

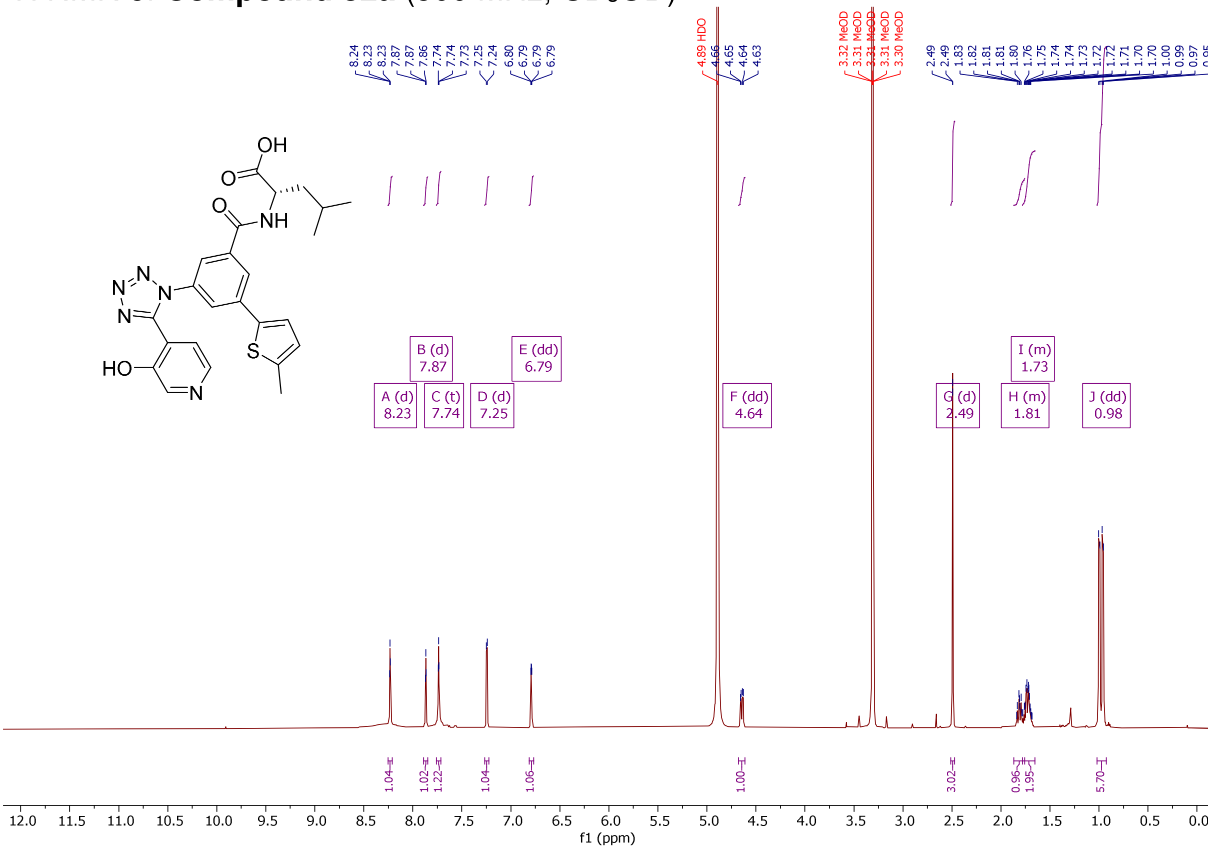

^{13}C NMR of **Compound 32a** (125 MHz, CD_3OD)

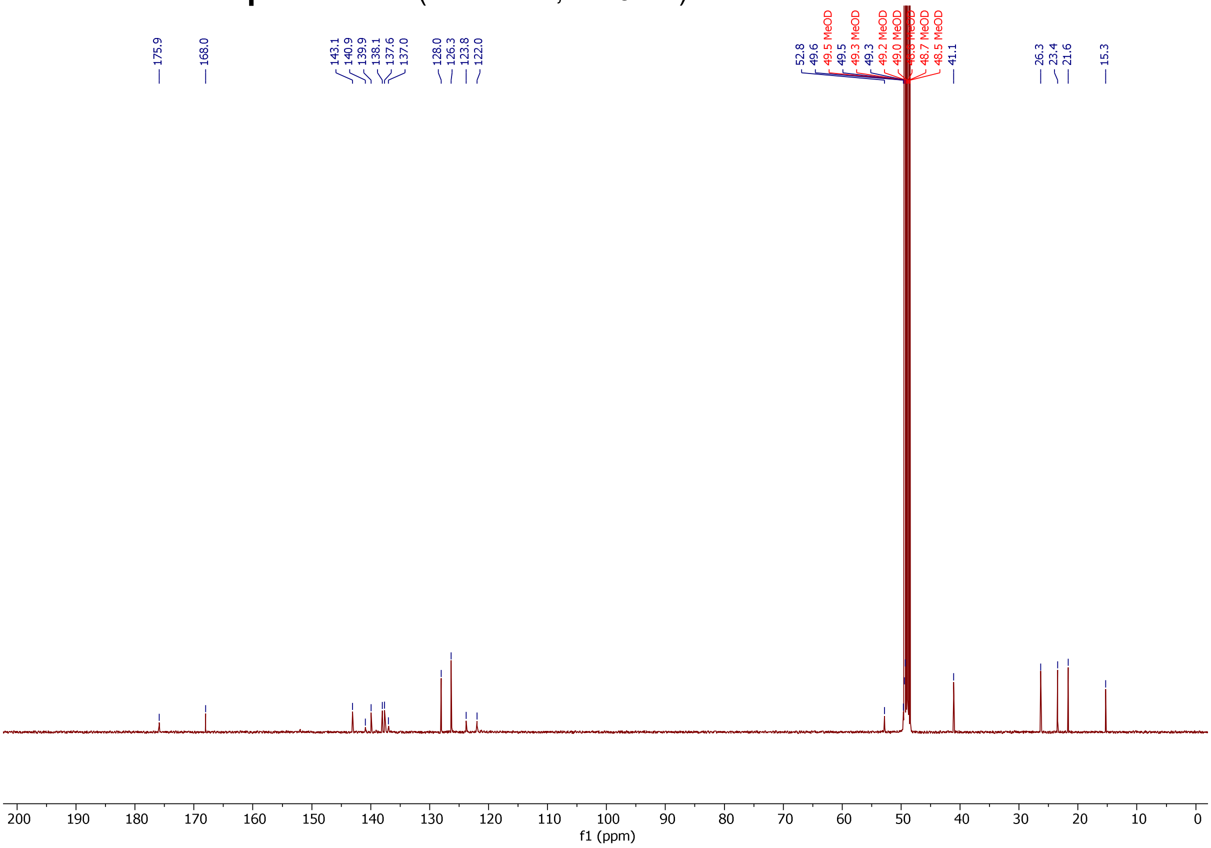

^{1}H NMR of **Compound 33a** (500 MHz, CD_3OD)

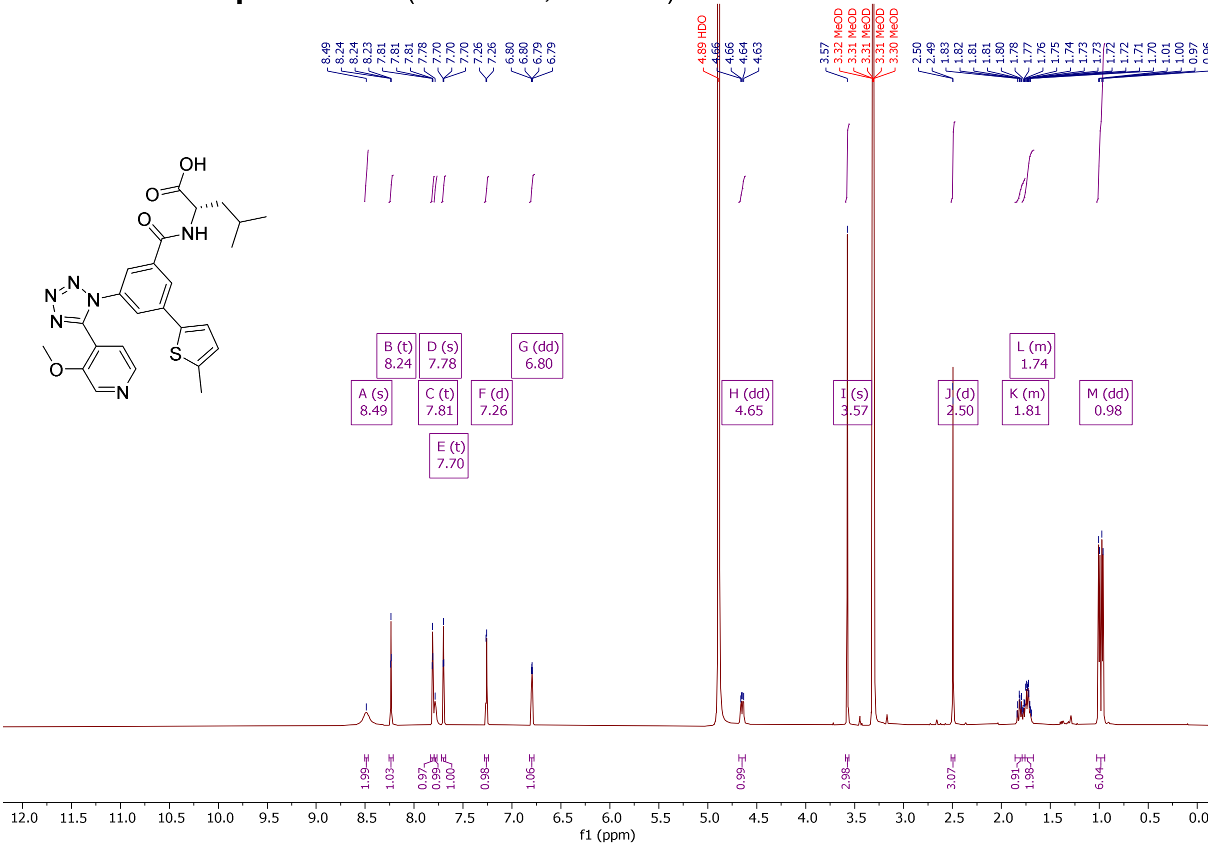

^{13}C NMR of **Compound 33a** (125 MHz, CD_3OD)

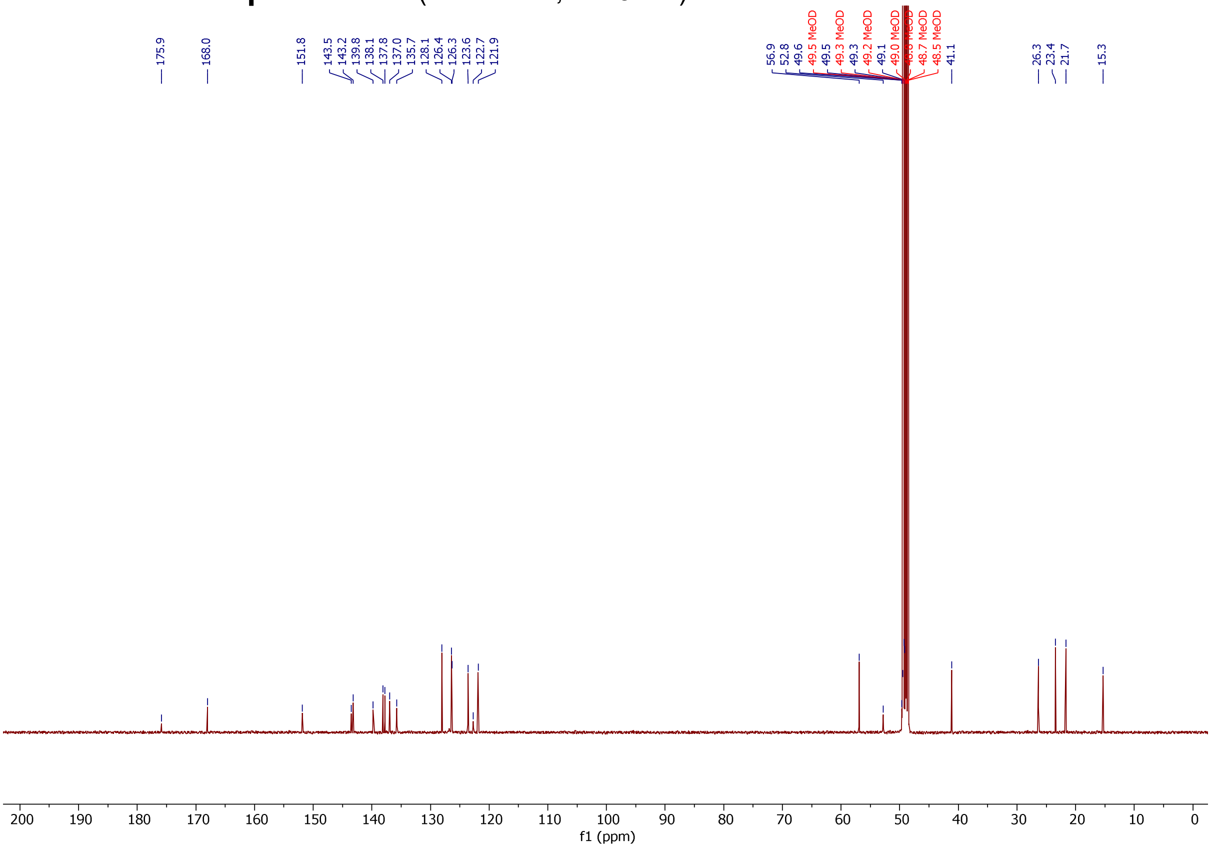

^{1}H NMR of **Compound 34a** (500 MHz, CD_3OD)

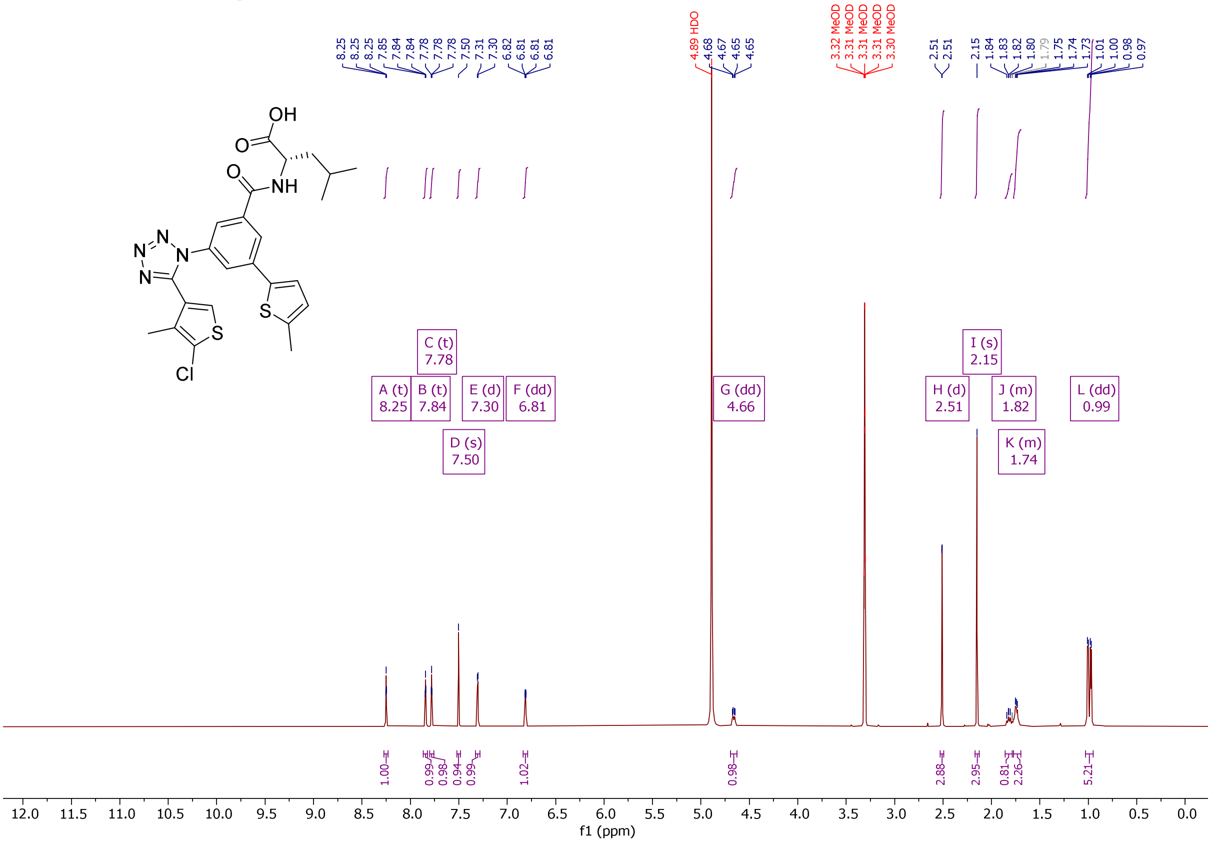

^{13}C NMR of **Compound 34a** (125 MHz, CD_3OD)

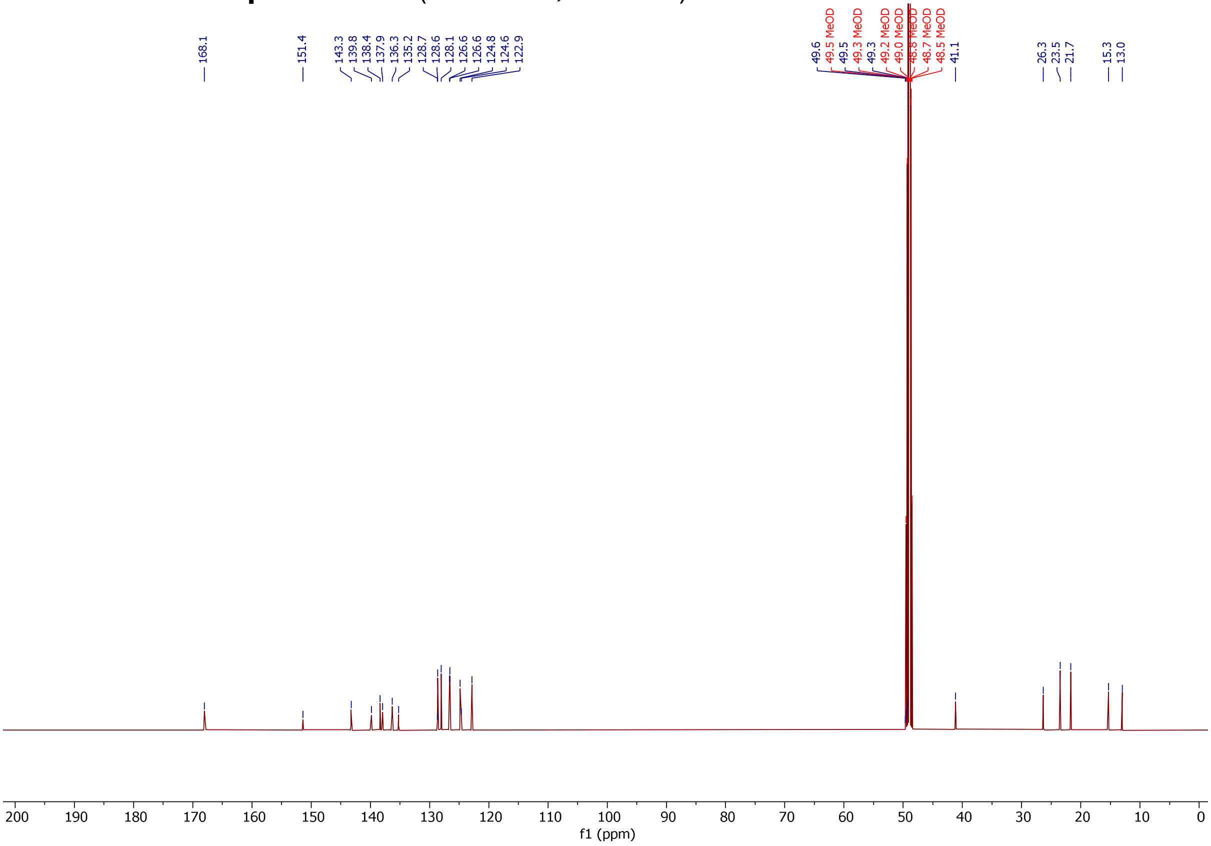

^{1}H NMR of **Compound 35a** (500 MHz, DMSO-d6)

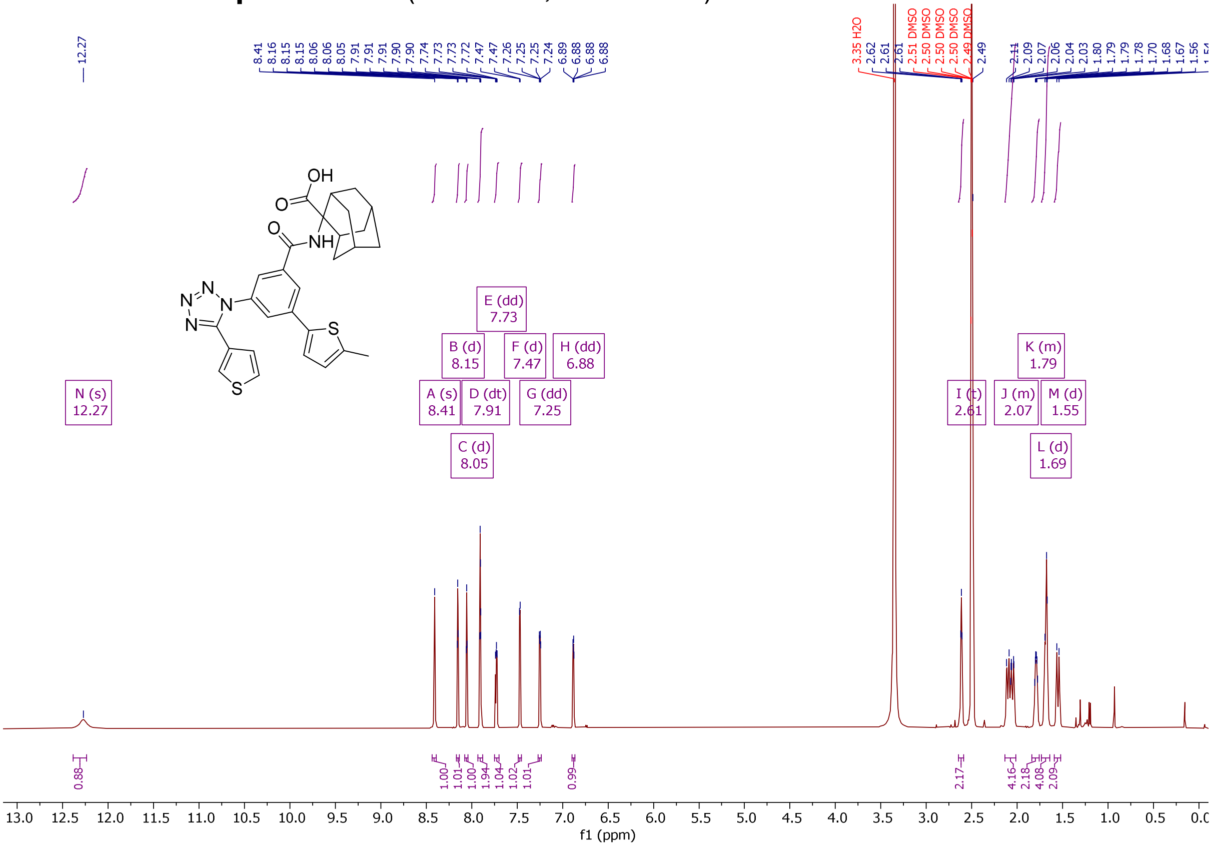

^{13}C NMR of **Compound 35a** (125 MHz, DMSO-d6)

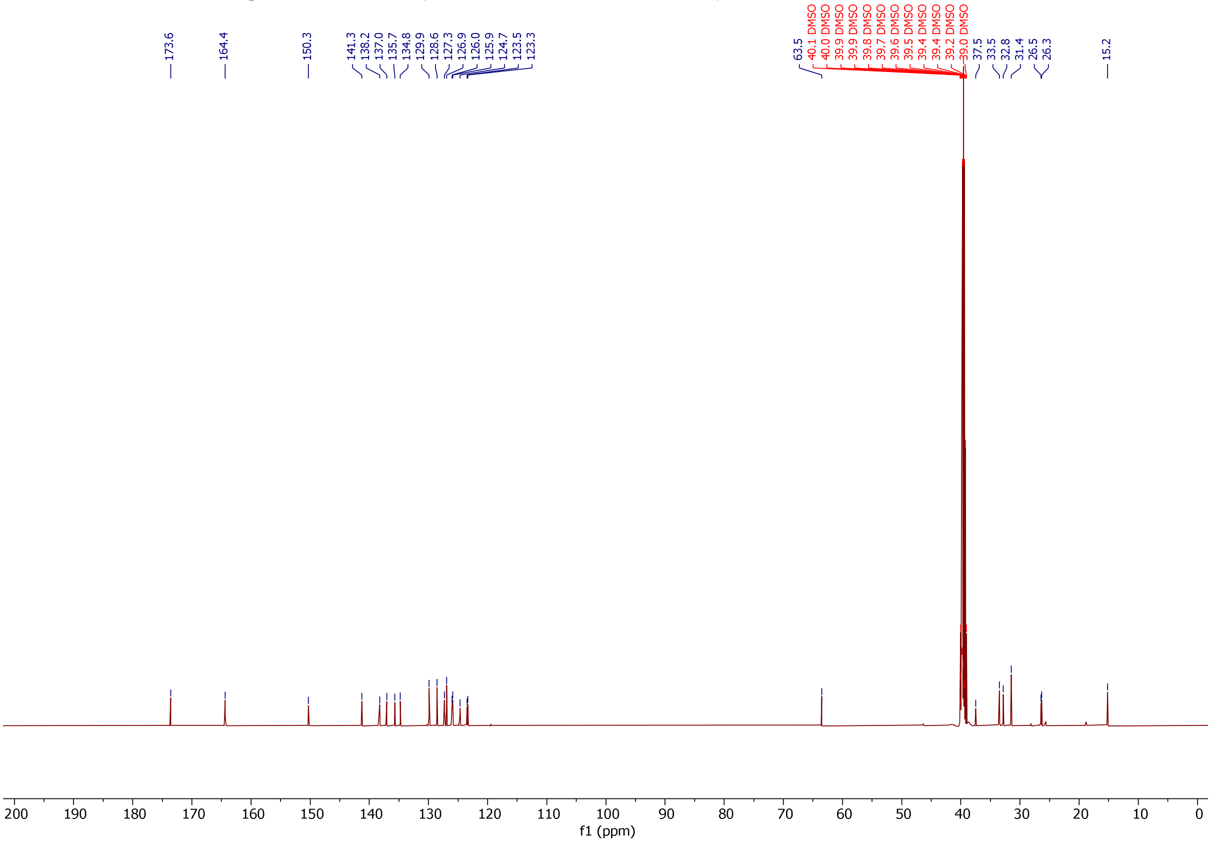

Compound 25 optimization (scaffold b) NMR and LCMS spectra

^{1}H NMR of **Compound 1b** (500 MHz, DMSO-d$_6$)

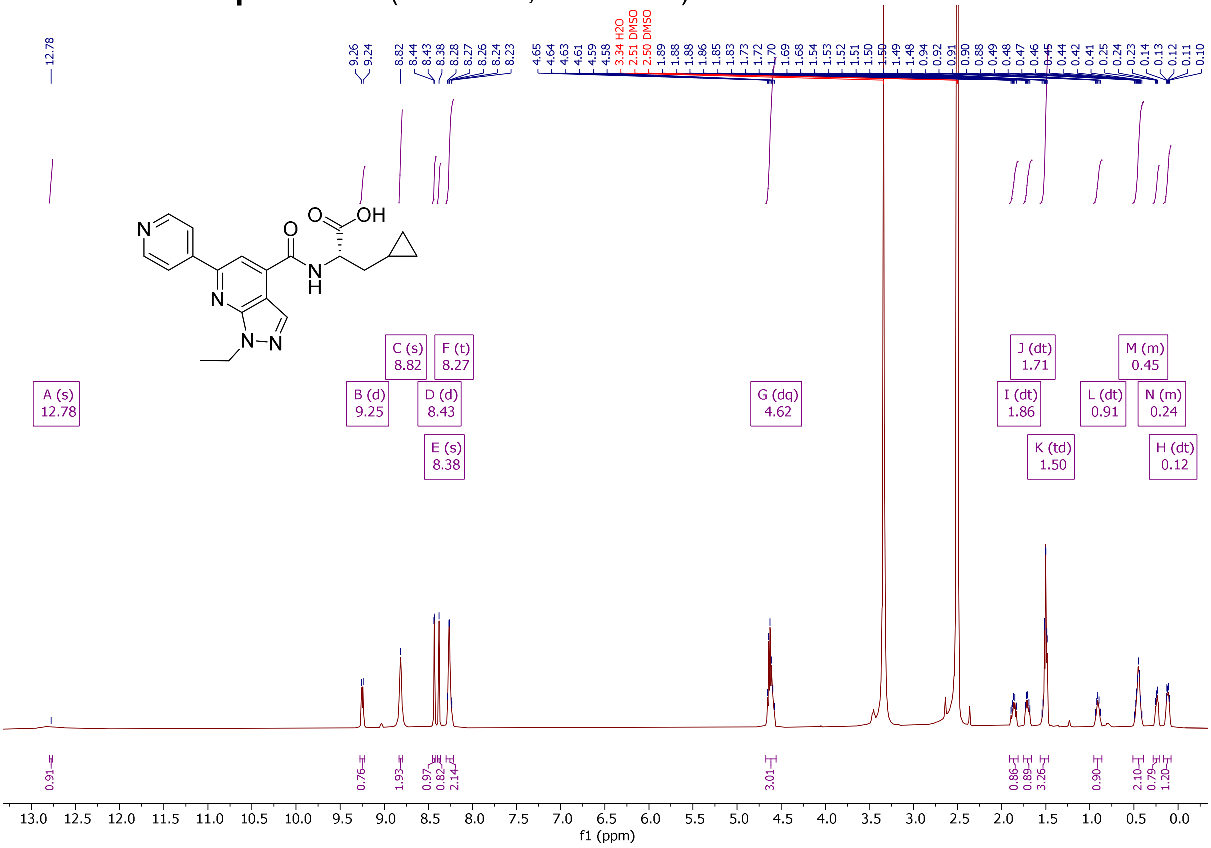

^{13}C NMR of **Compound 1b** (125 MHz, DMSO-d$_6$)

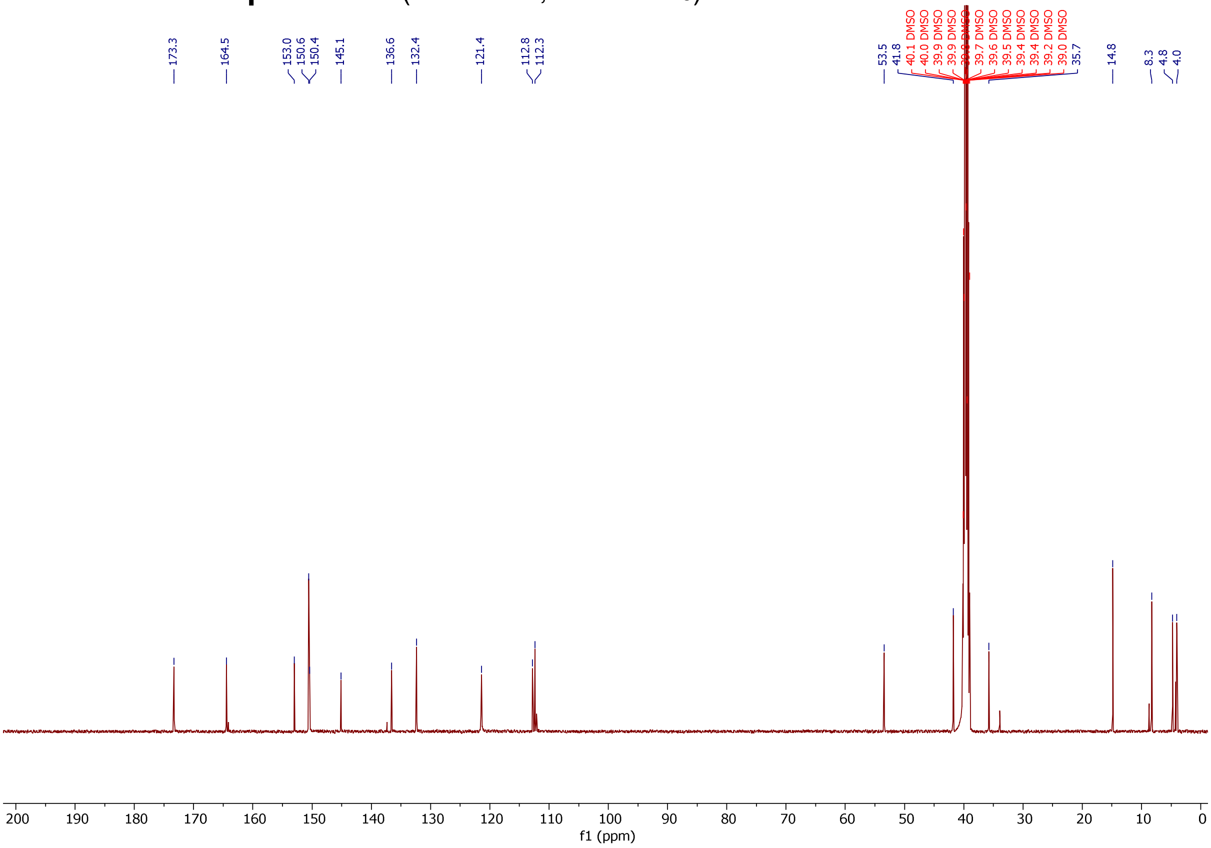

^{1}H NMR of **Compound 2b** (500 MHz, DMSO-d6)

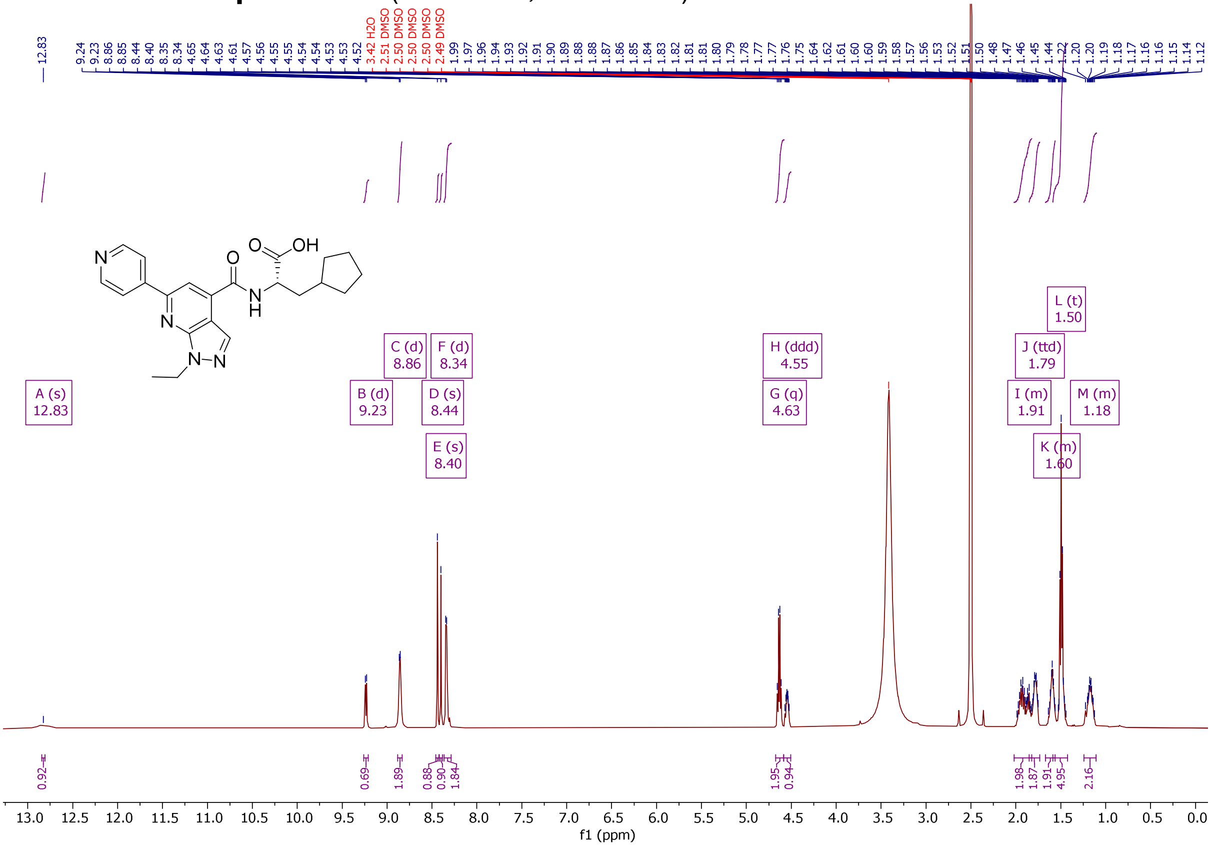

^{13}C NMR of **Compound 2b** (125 MHz, DMSO-d6)

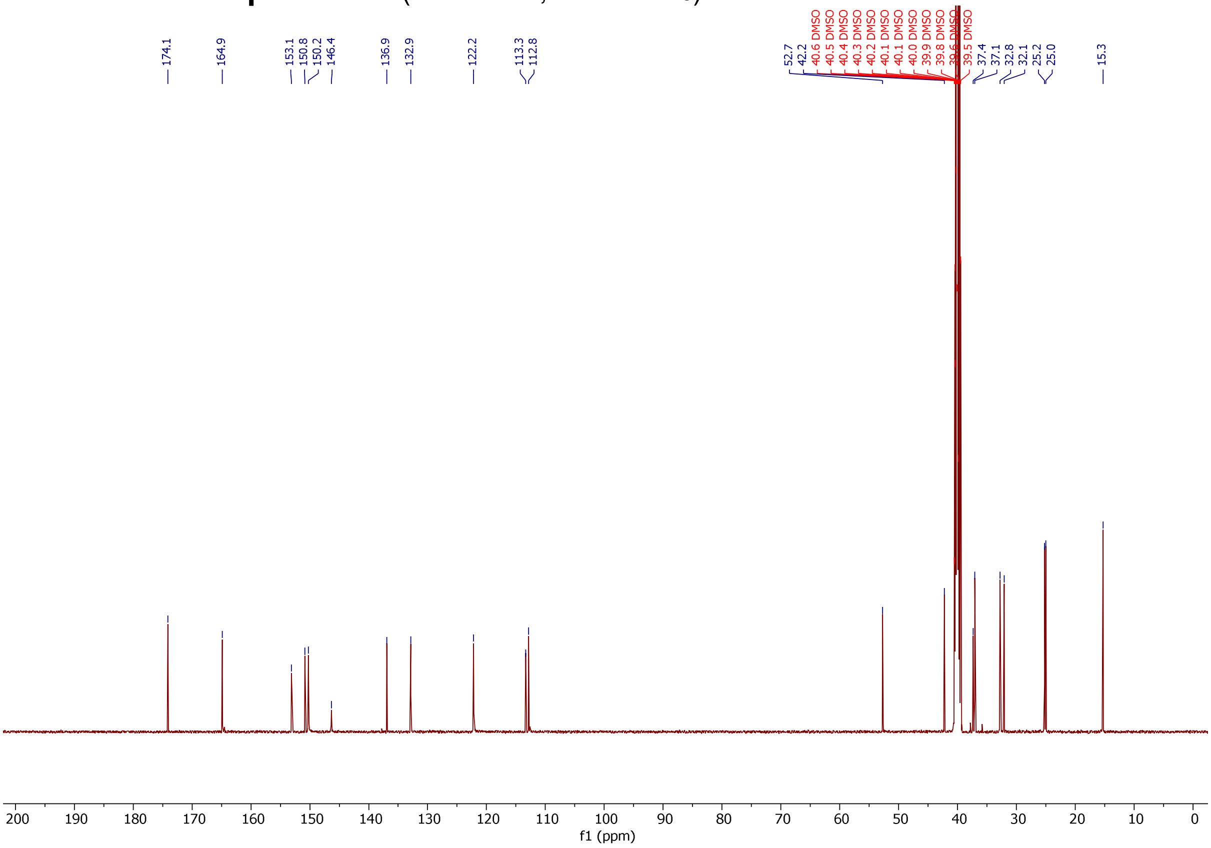

^{1}H NMR of **Compound 3b** (500 MHz, CD_3OD)

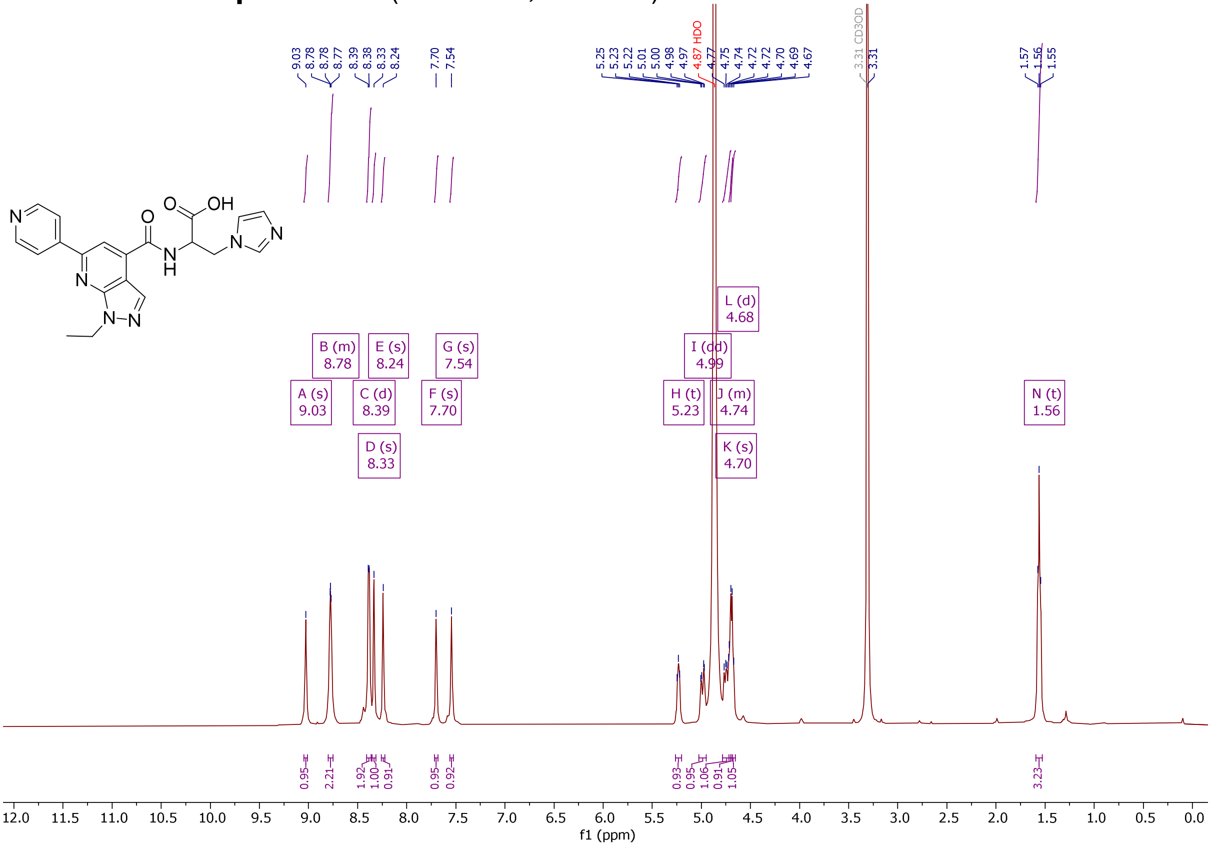

^{13}C NMR of **Compound 3b** (125 MHz, CD_3OD)

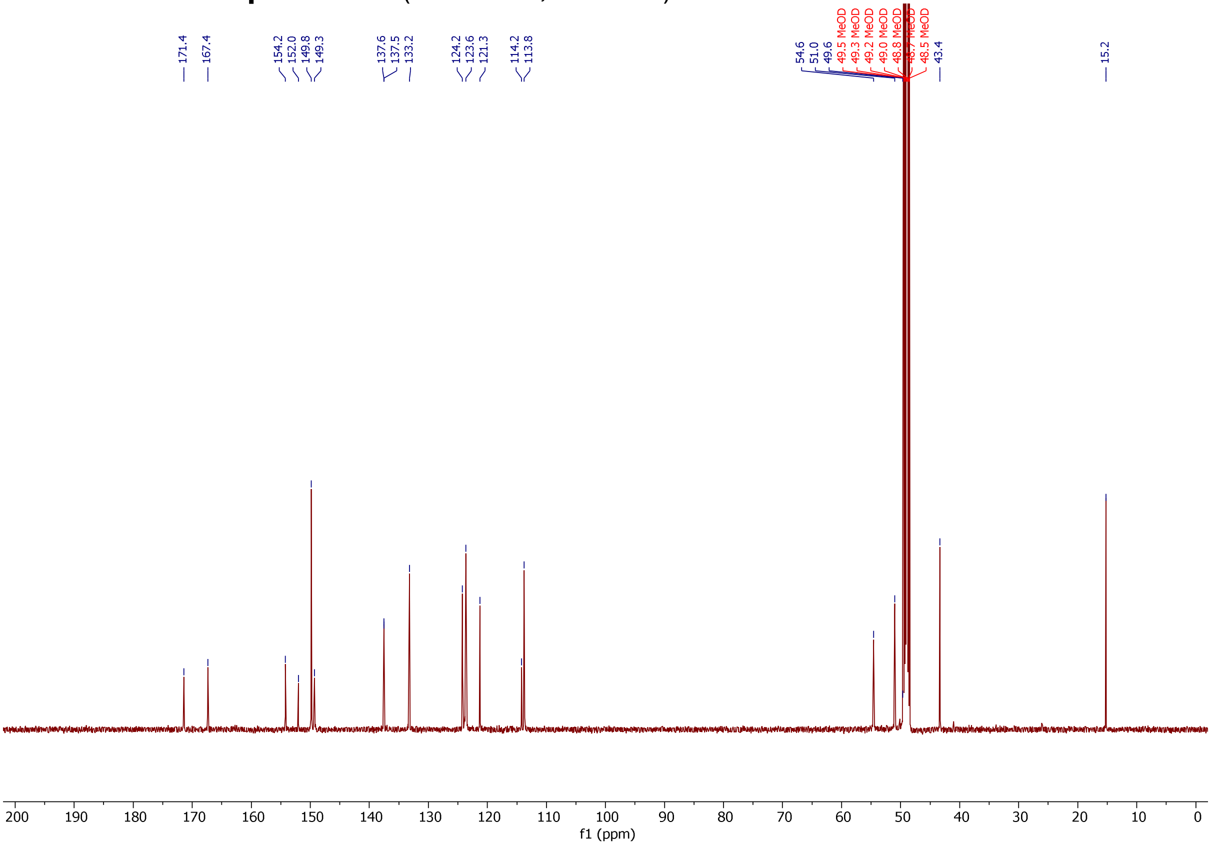

^{1}H NMR of **Compound 4b** (500 MHz, CD_3OD)

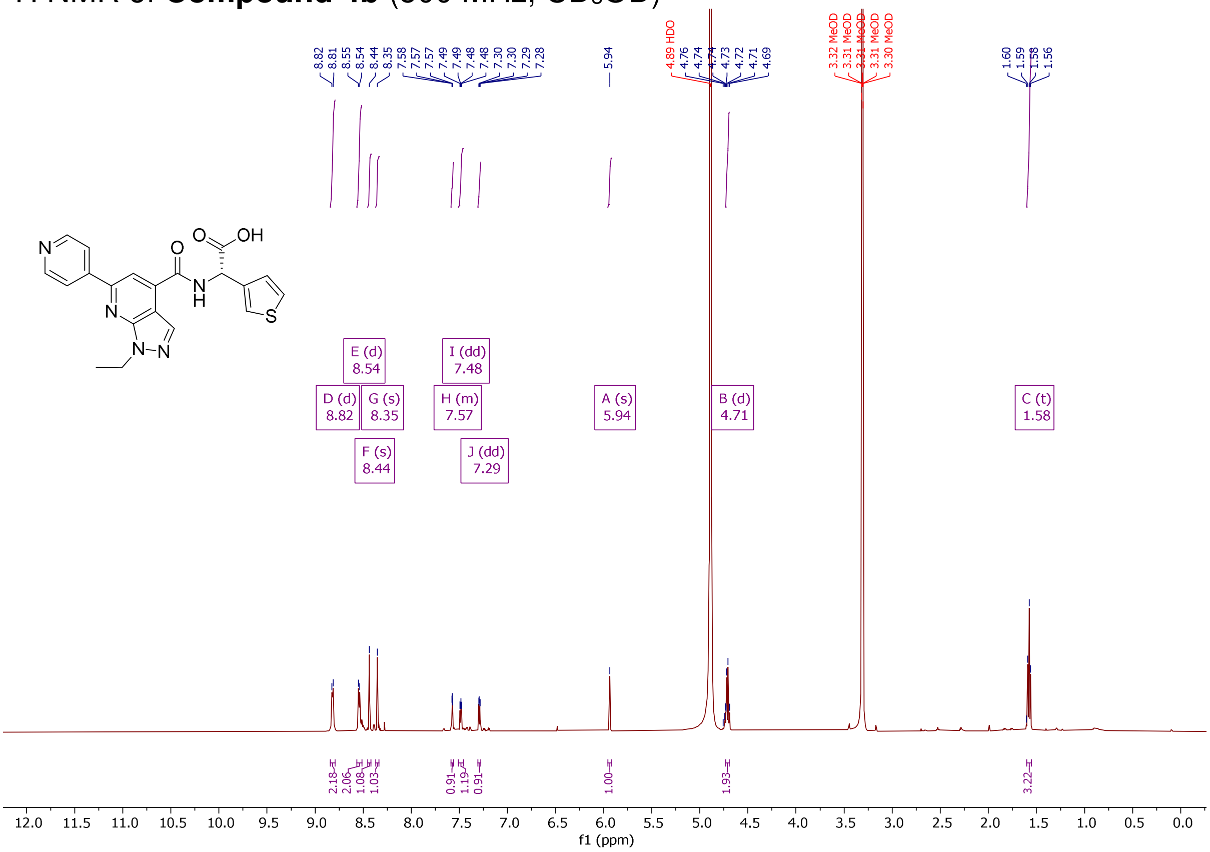

^{13}C NMR of **Compound 4b** (125 MHz, CD_3OD)

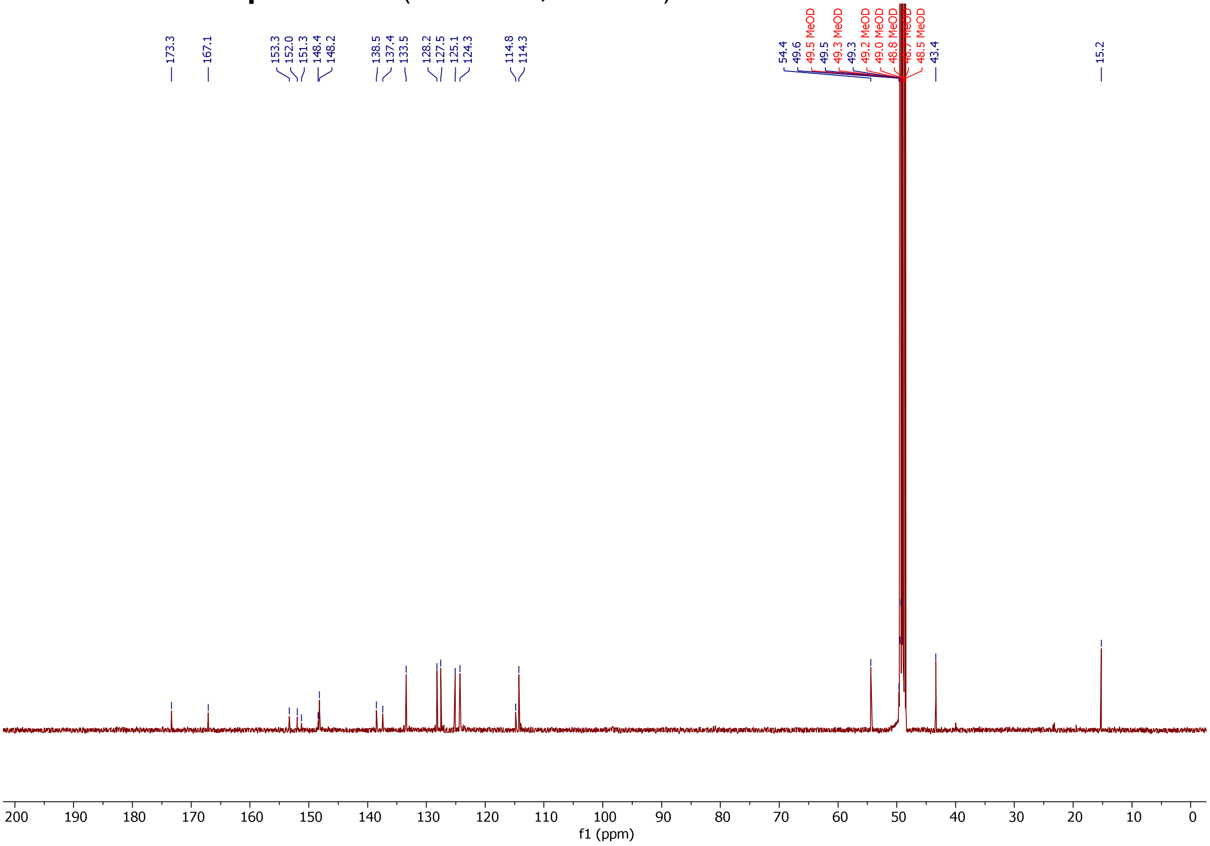

^{1}H NMR of **Compound 5b** (500 MHz, DMSO-d6)

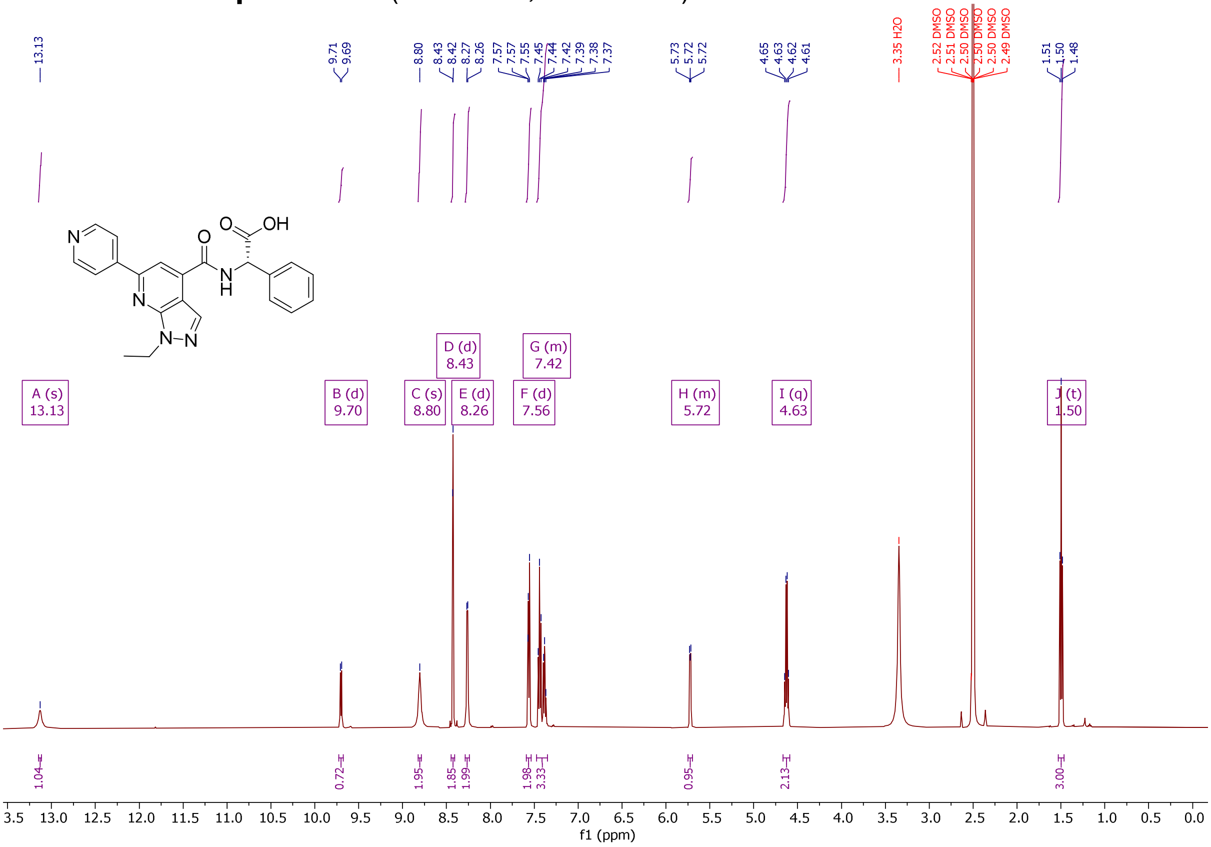

^{13}C NMR of **Compound 5b** (125 MHz, DMSO-d6)

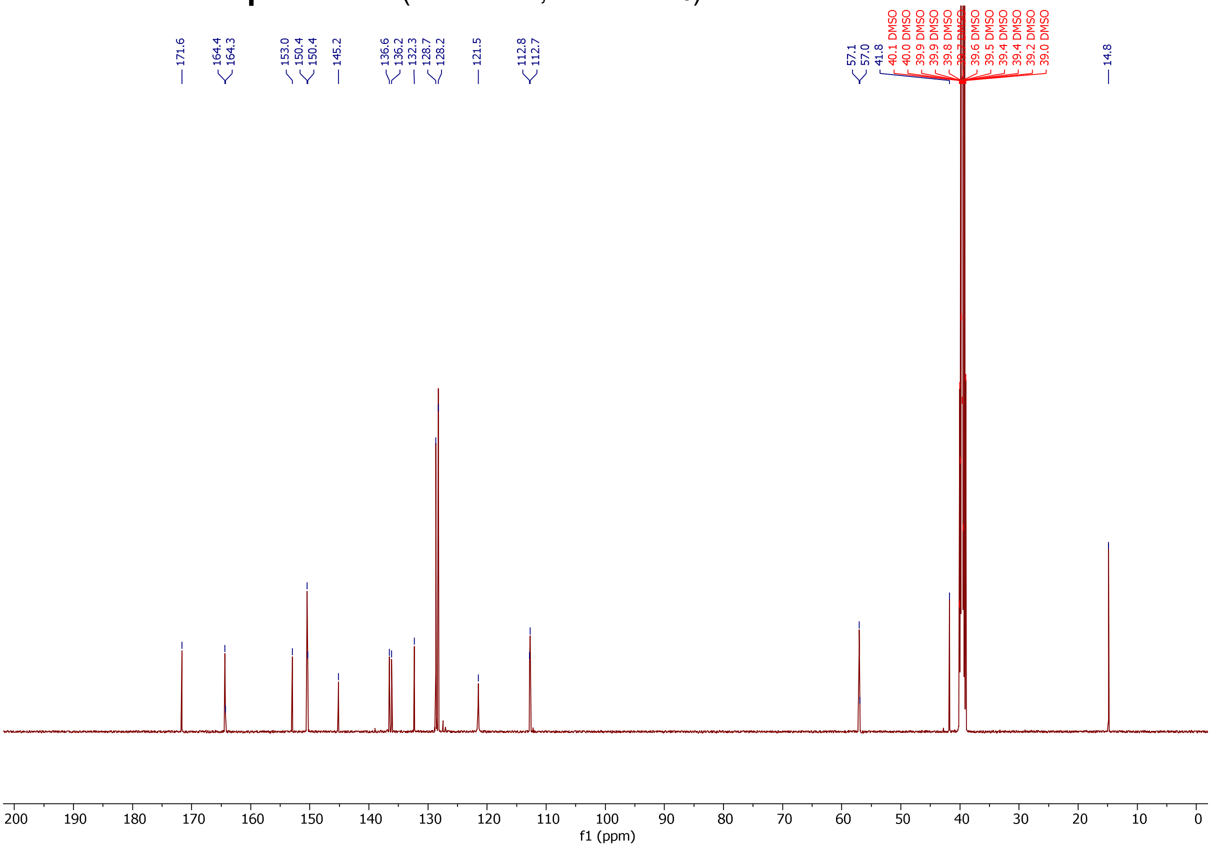

^{1}H NMR of **Compound 6b** (500 MHz, DMSO-d6)

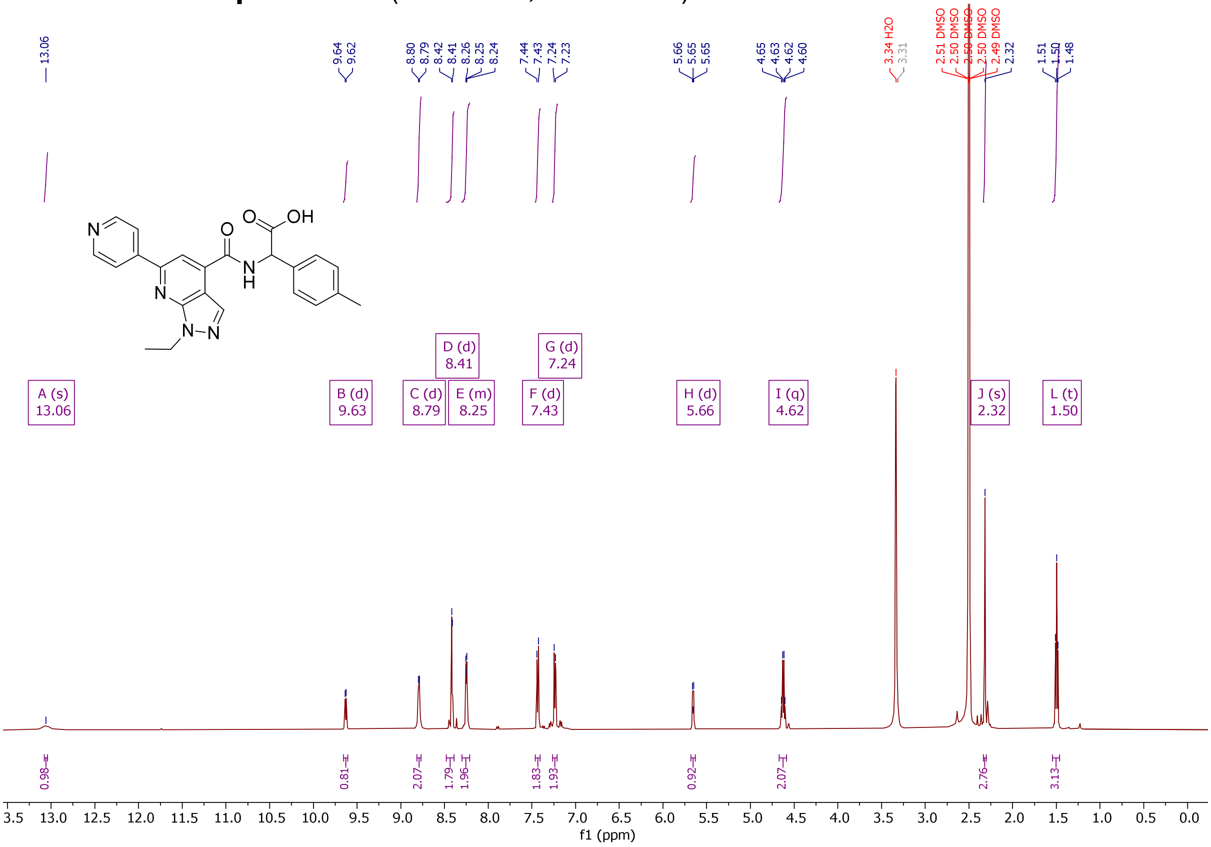

^{13}C NMR of **Compound 6b** (125 MHz, DMSO-d6)

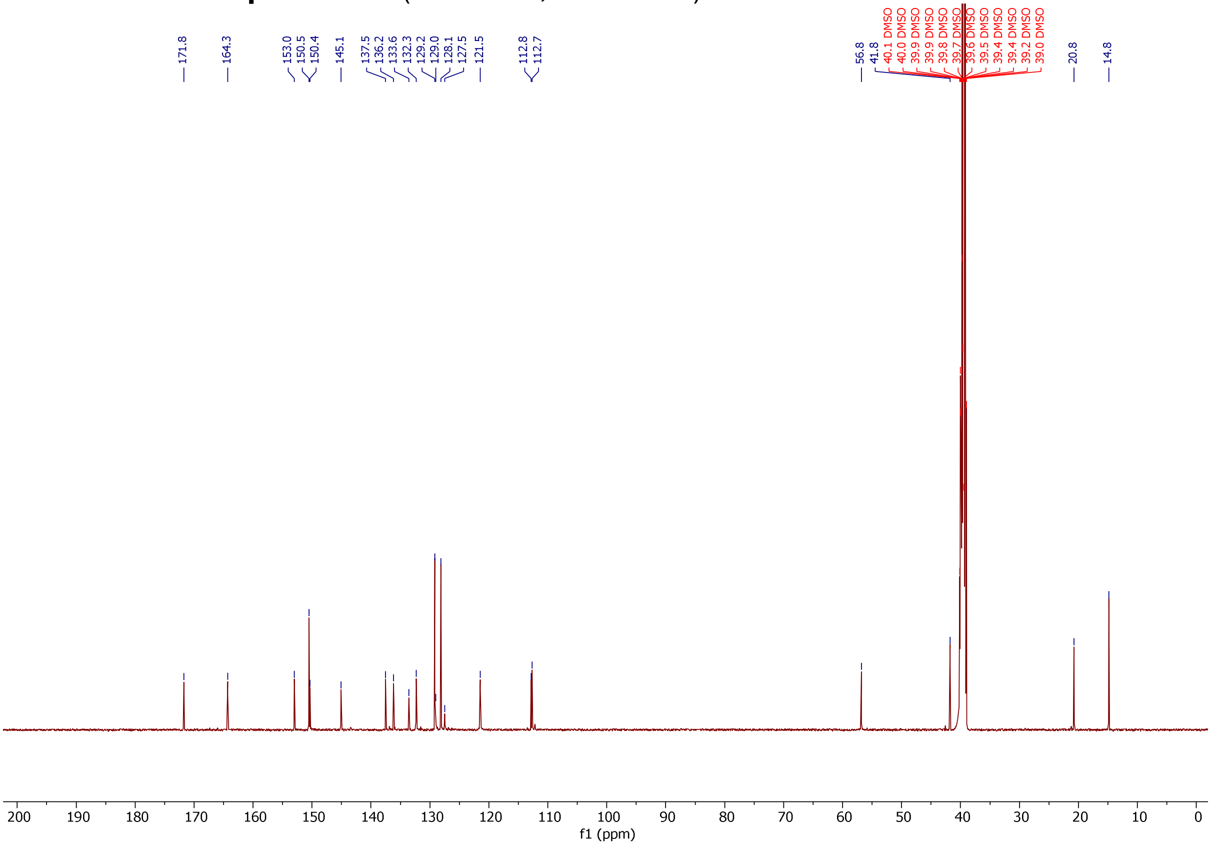

^{1}H NMR of **Compound 7b** (500 MHz, DMSO-d6)

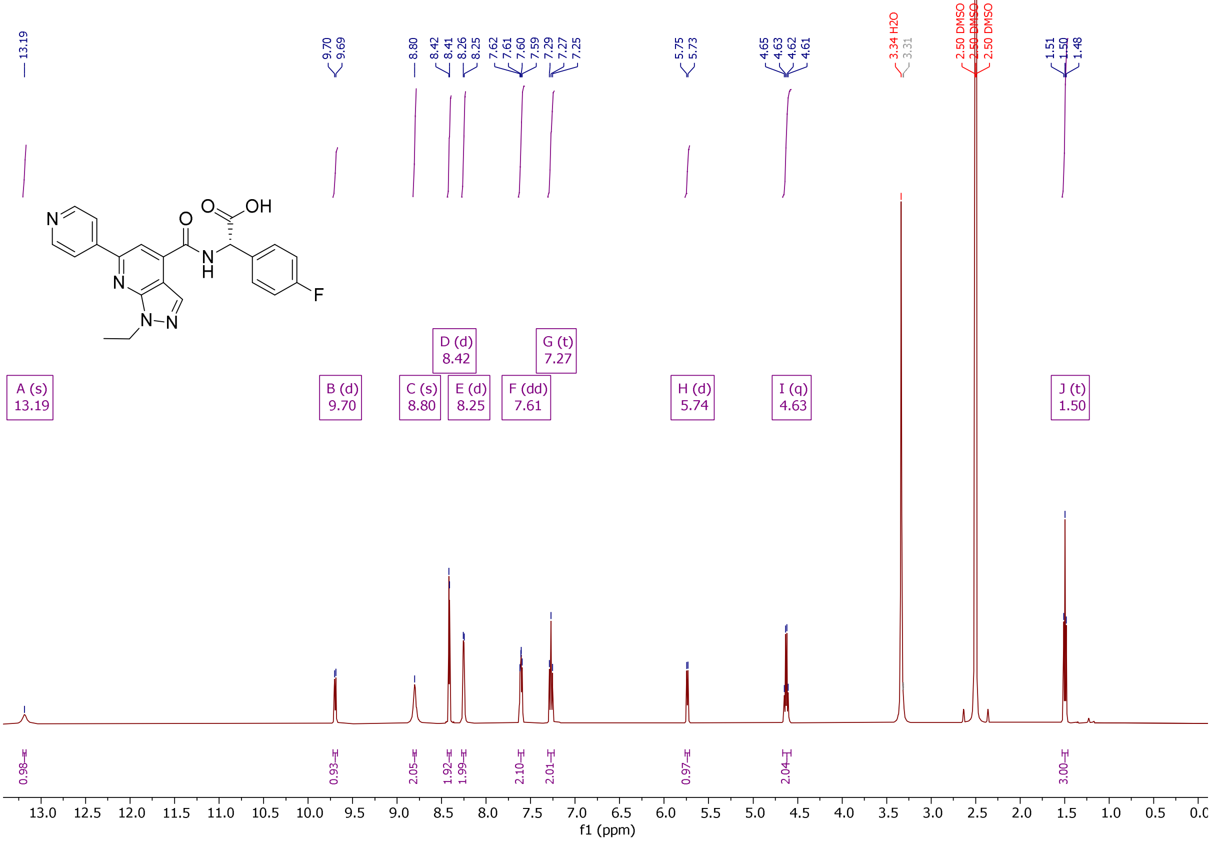

^{13}C NMR of **Compound 7b** (125 MHz, DMSO-d6)

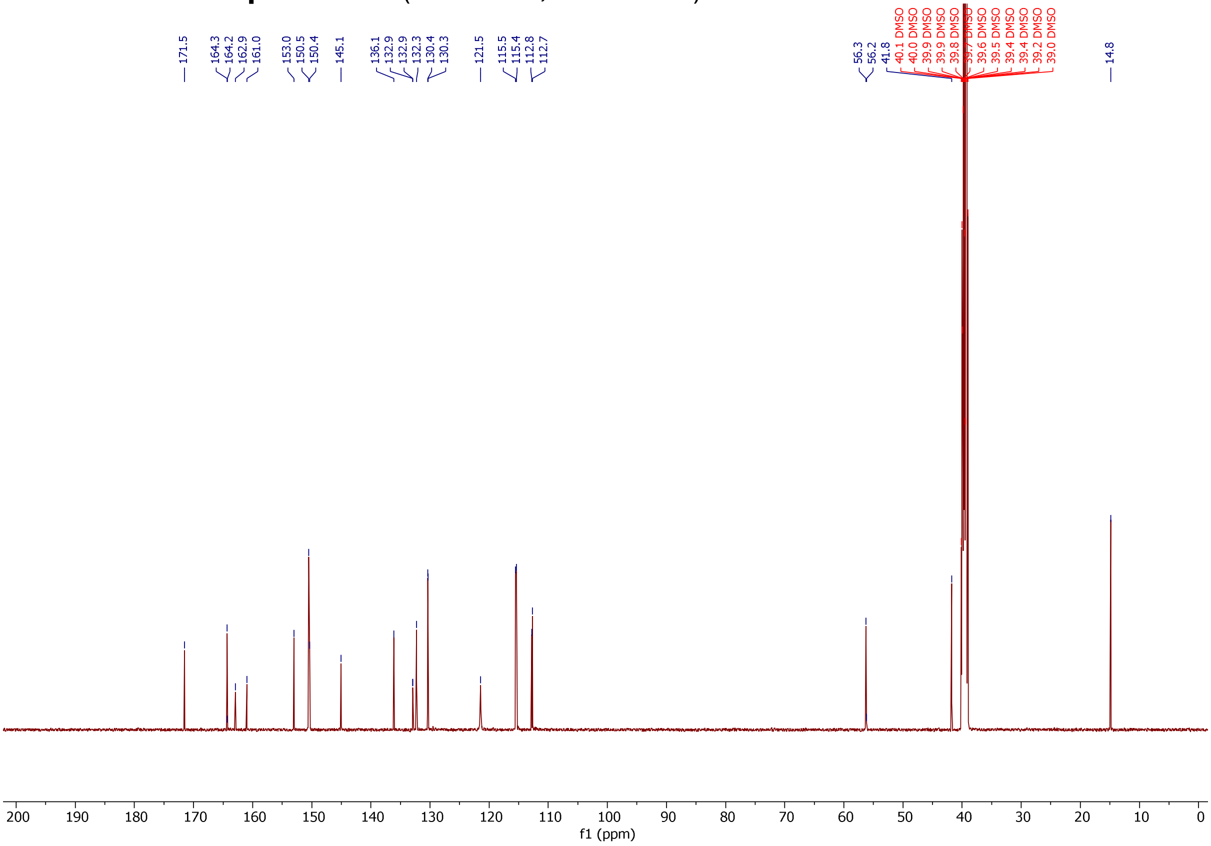

^{1}H NMR of **Compound 8b** (500 MHz, DMSO-d6)

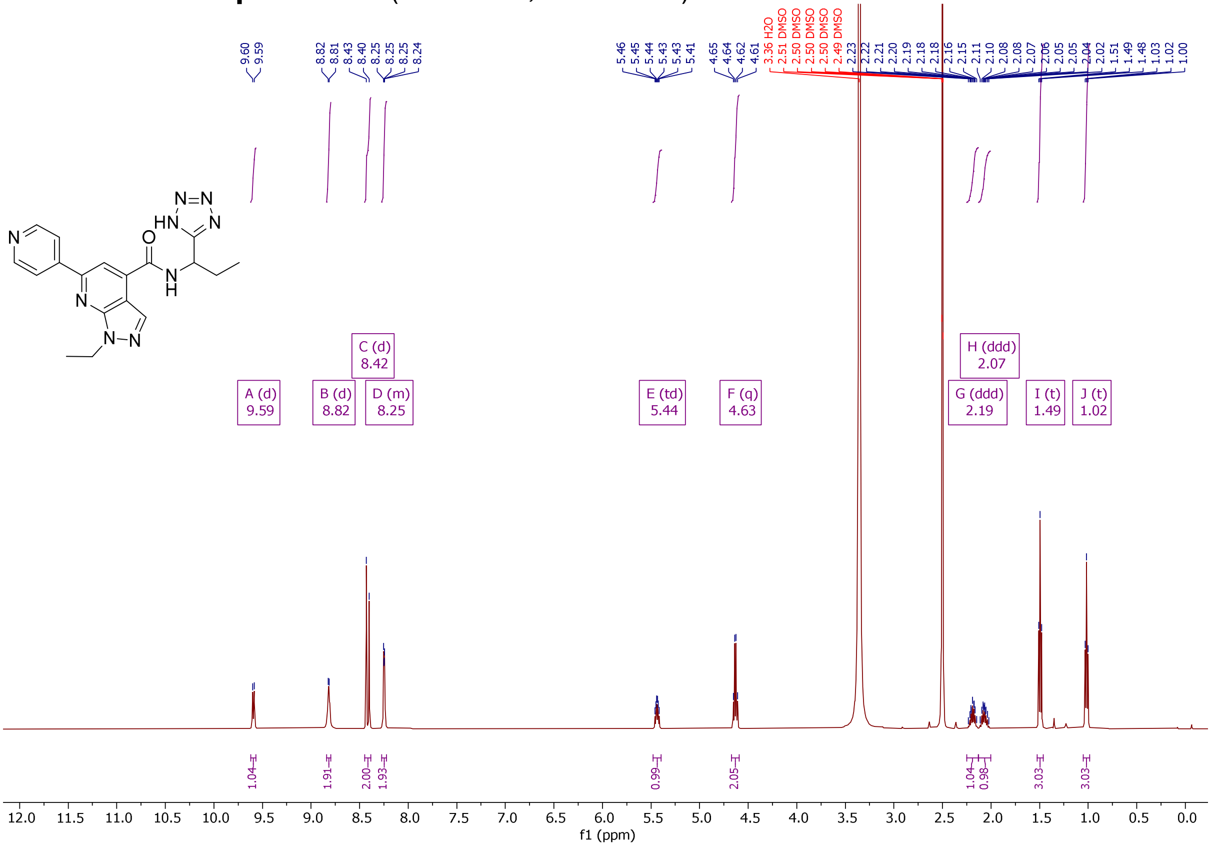

^{13}C NMR of **Compound 8b** (150 MHz, DMSO-d6)

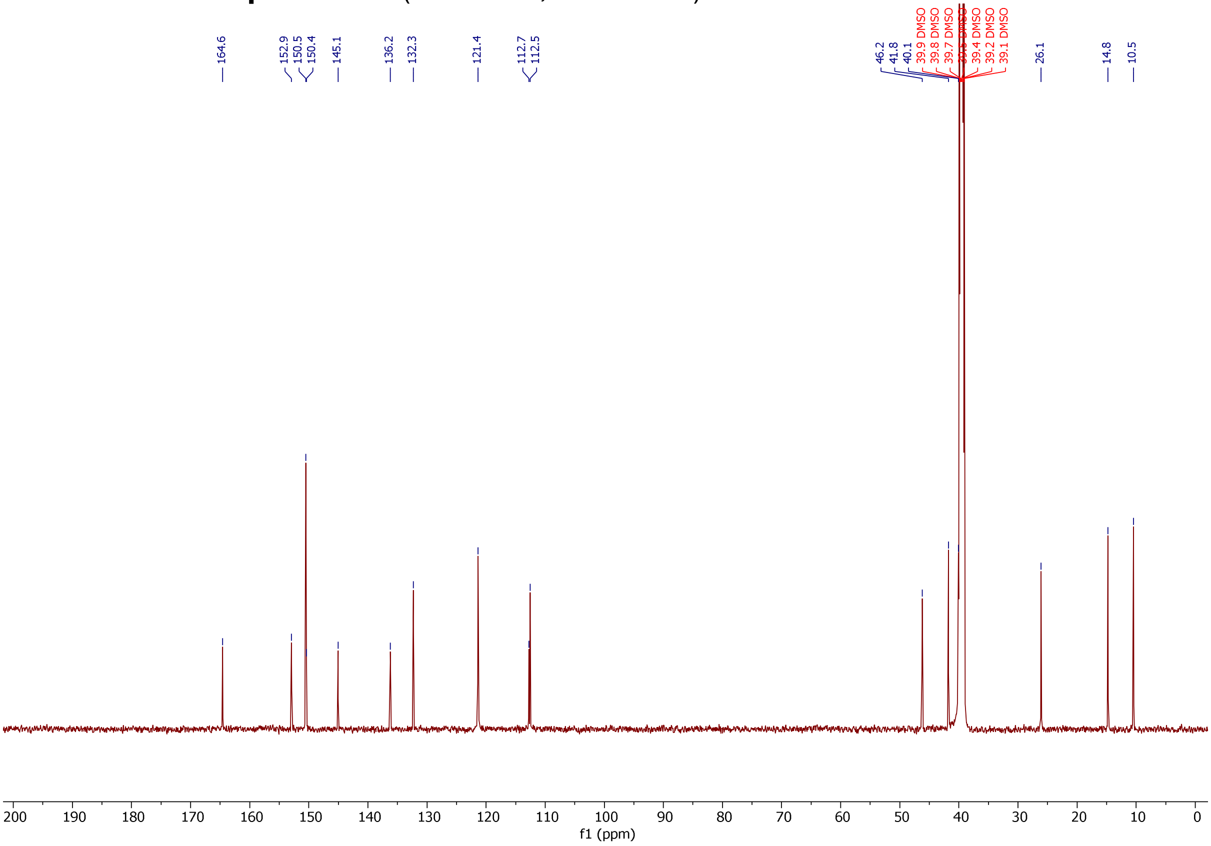

^{1}H NMR of **Compound 9b** (500 MHz, DMSO-d$_6$)

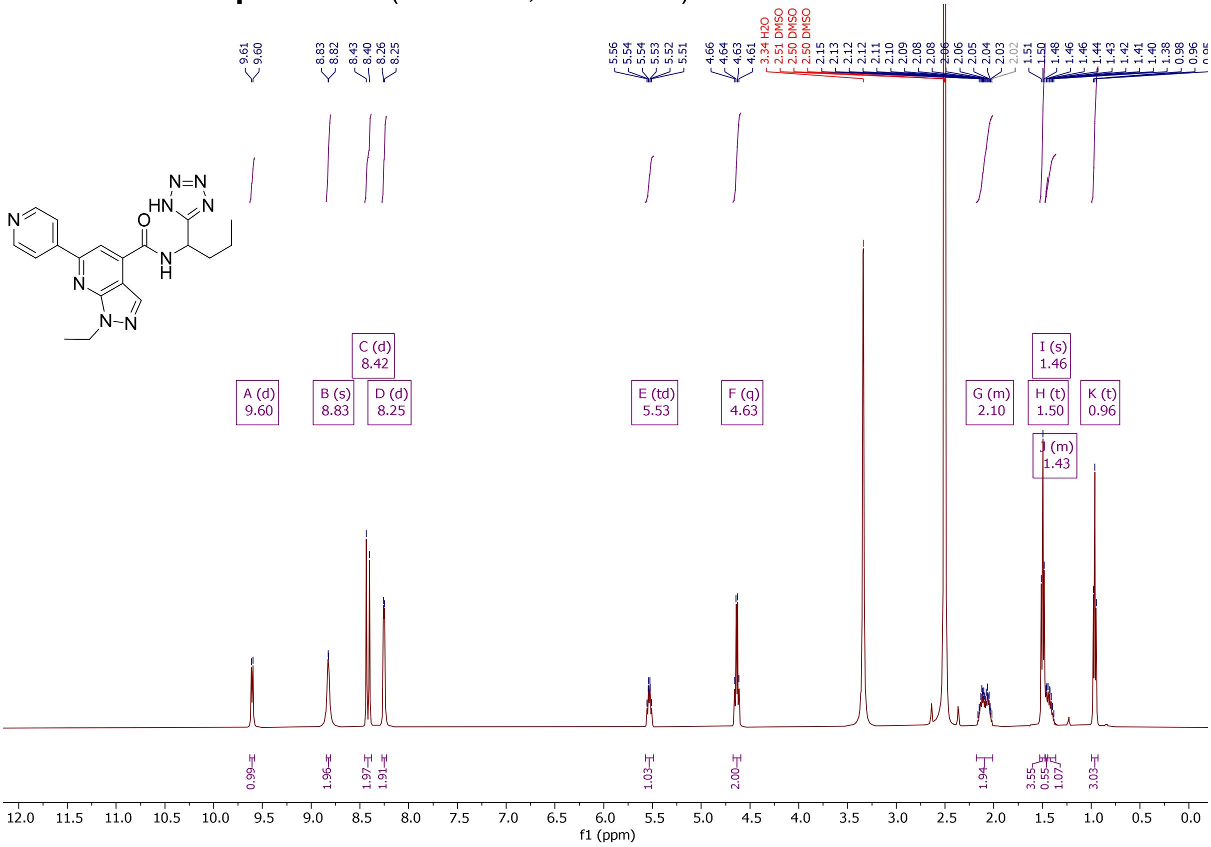

^{13}C NMR of **Compound 9b** (150 MHz, DMSO-d$_6$)

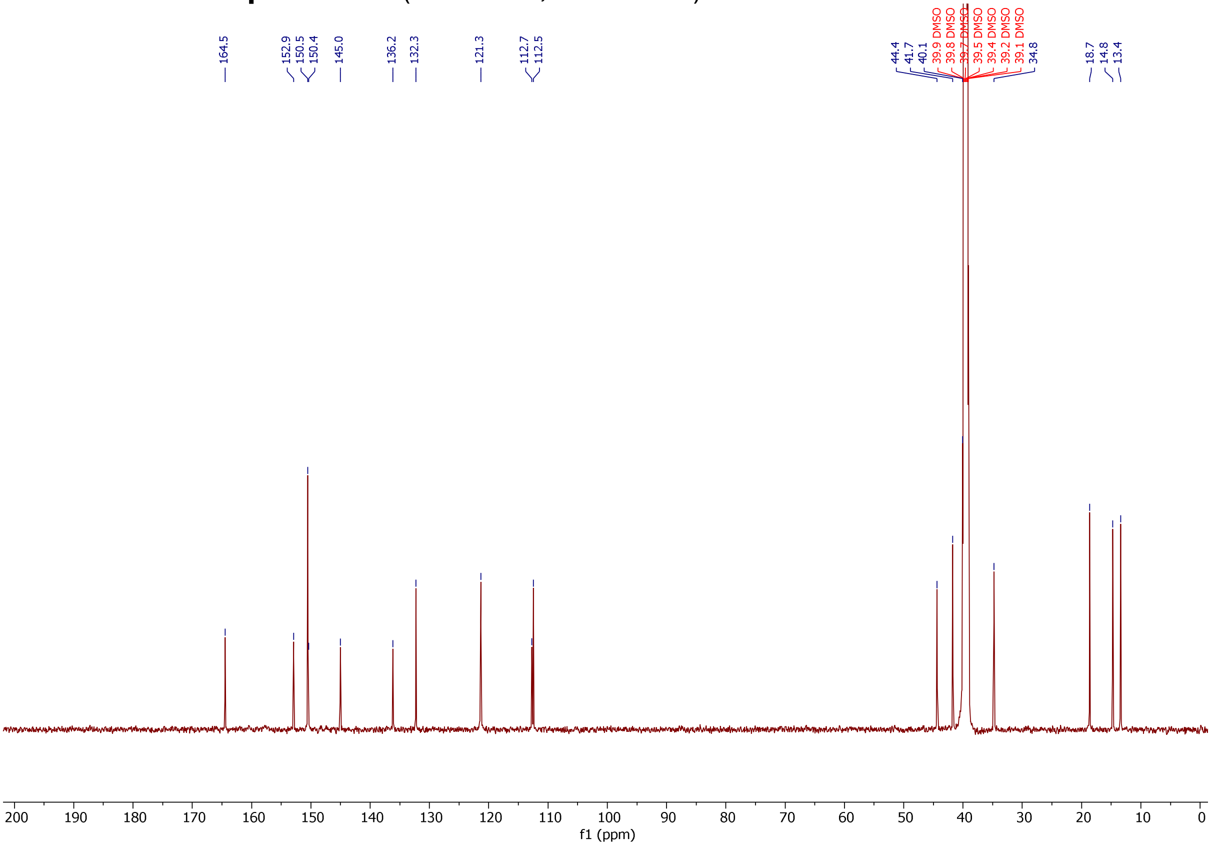

^{1}H NMR of **Compound 10b** (500 MHz, DMSO-d$_6$)

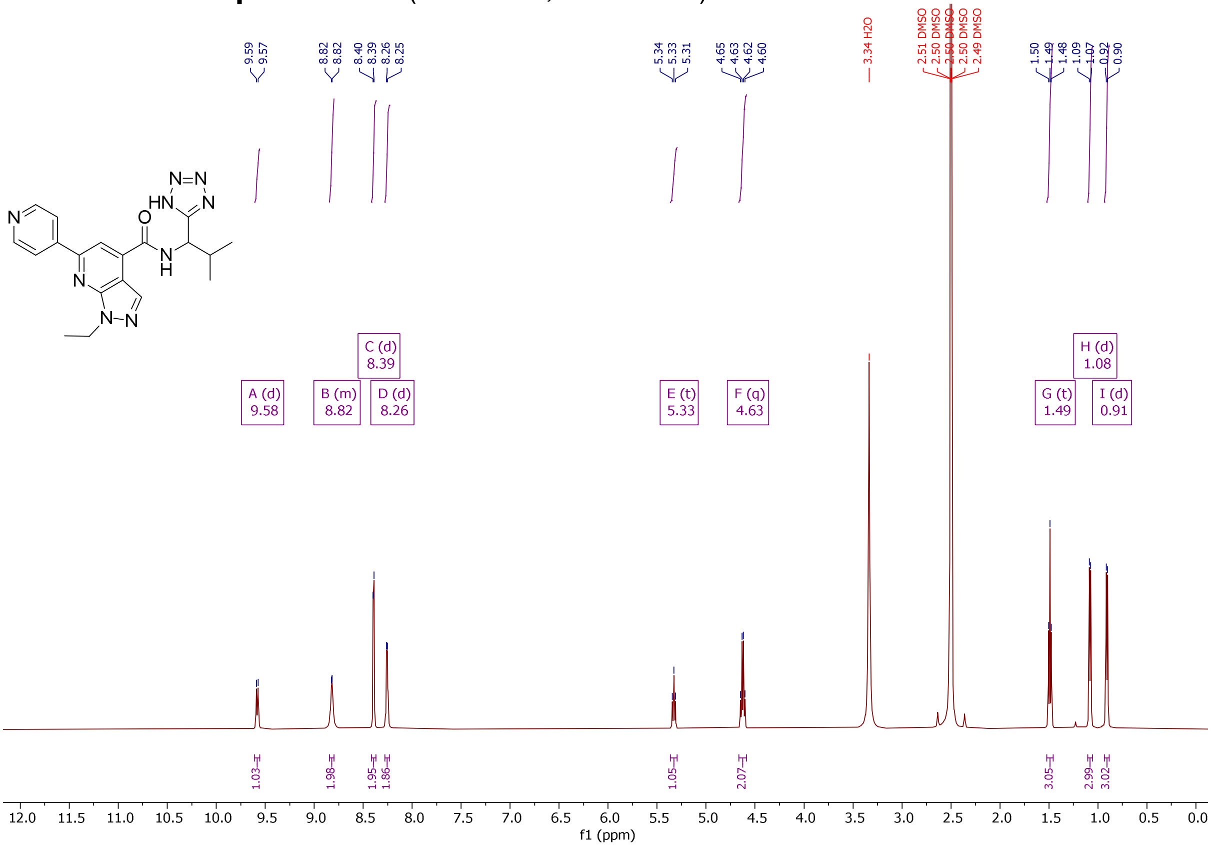

^{13}C NMR of **Compound 10b** (150 MHz, DMSO-d$_6$)

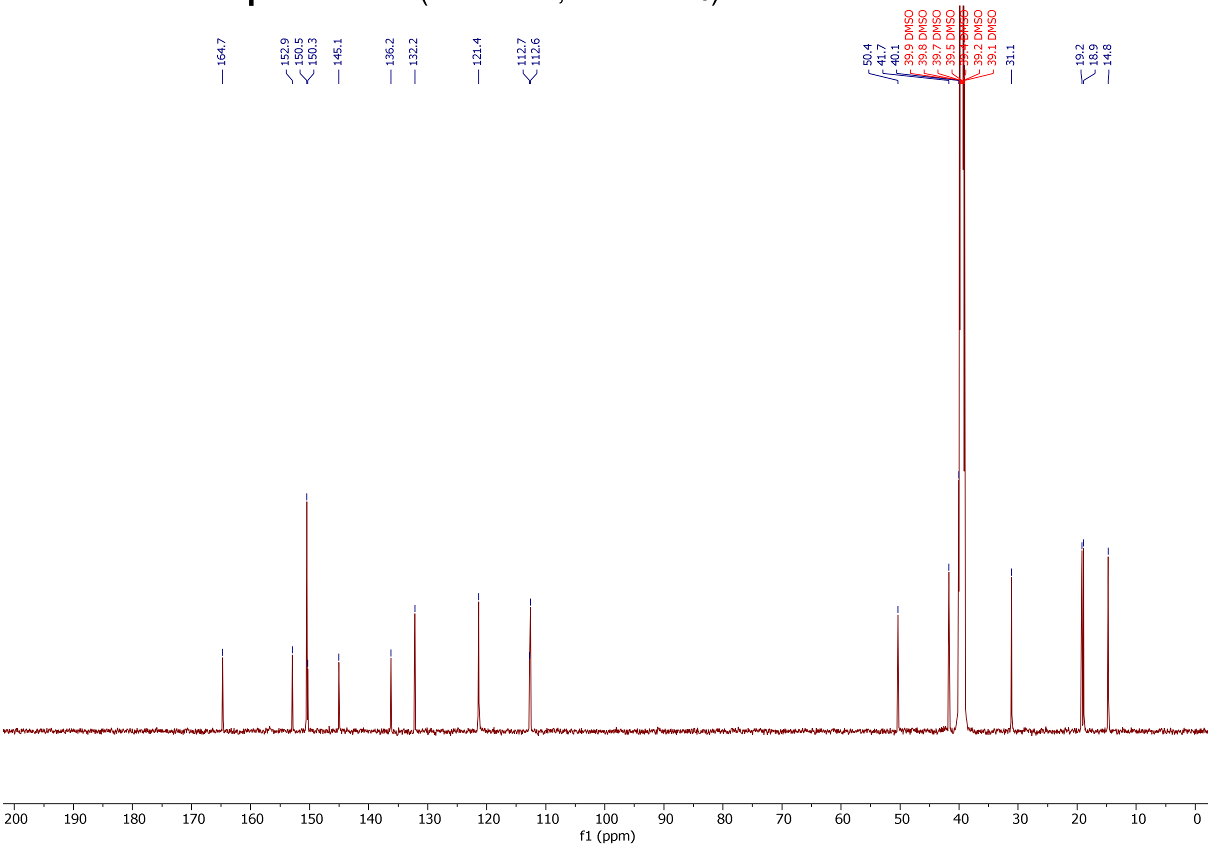

^{1}H NMR of **Compound 11b** (500 MHz, DMSO-d_6)

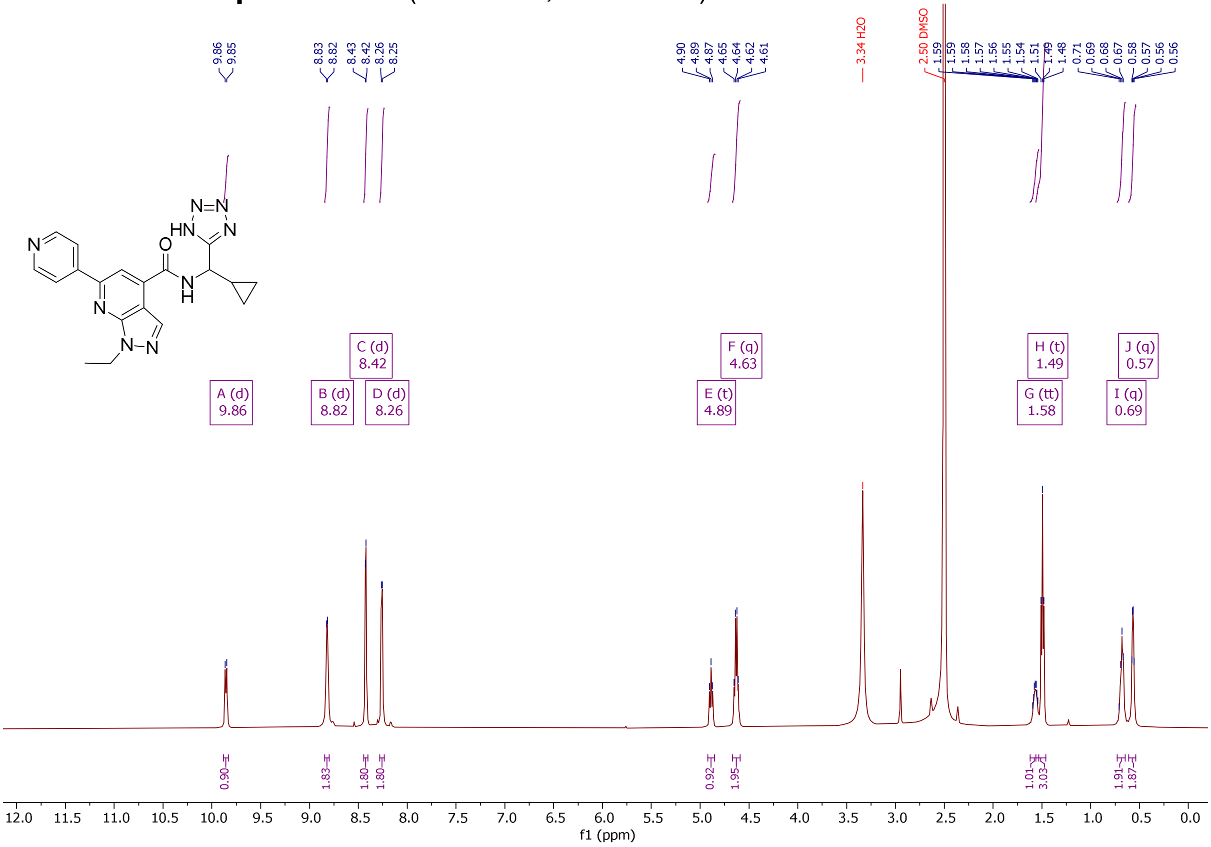

^{13}C NMR of **Compound 11b** (150 MHz, DMSO-d_6)

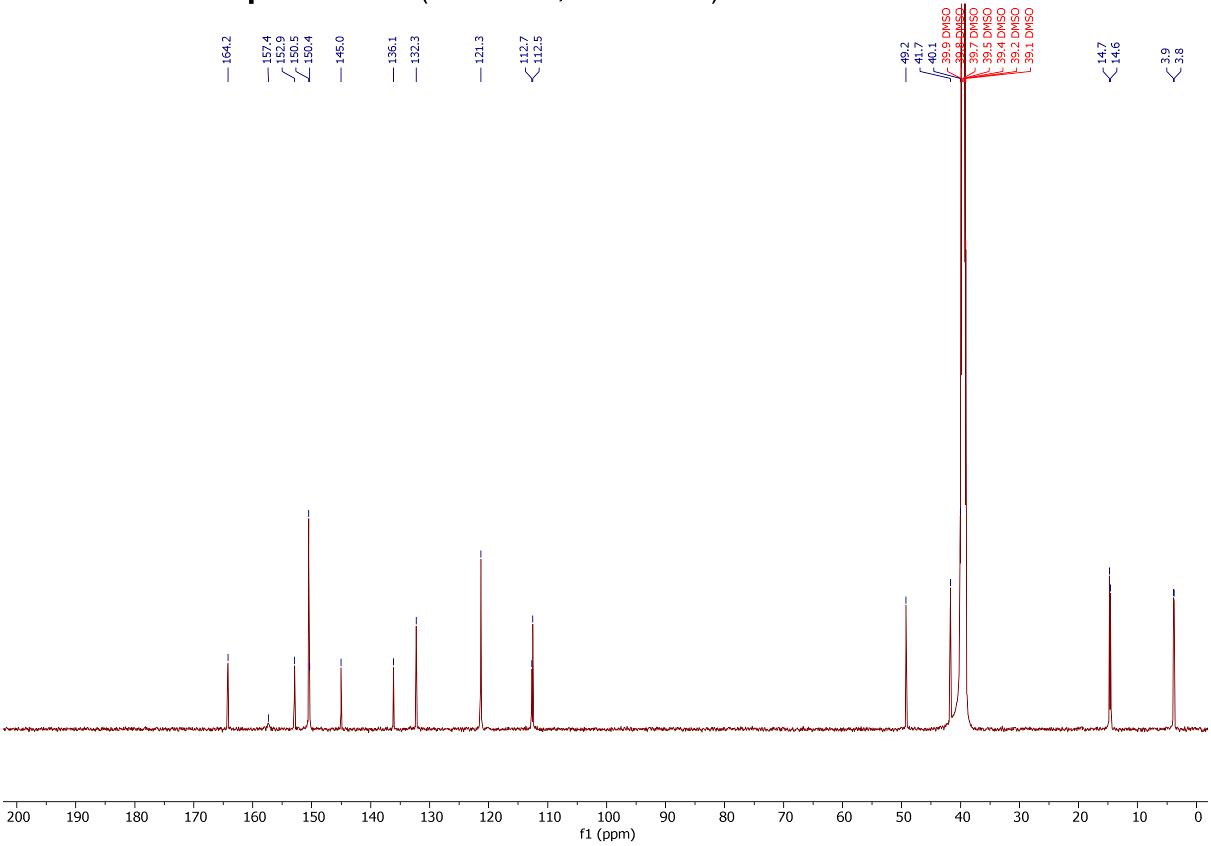

^{1}H NMR of **Compound 12b** (500 MHz, DMSO-d$_6$)

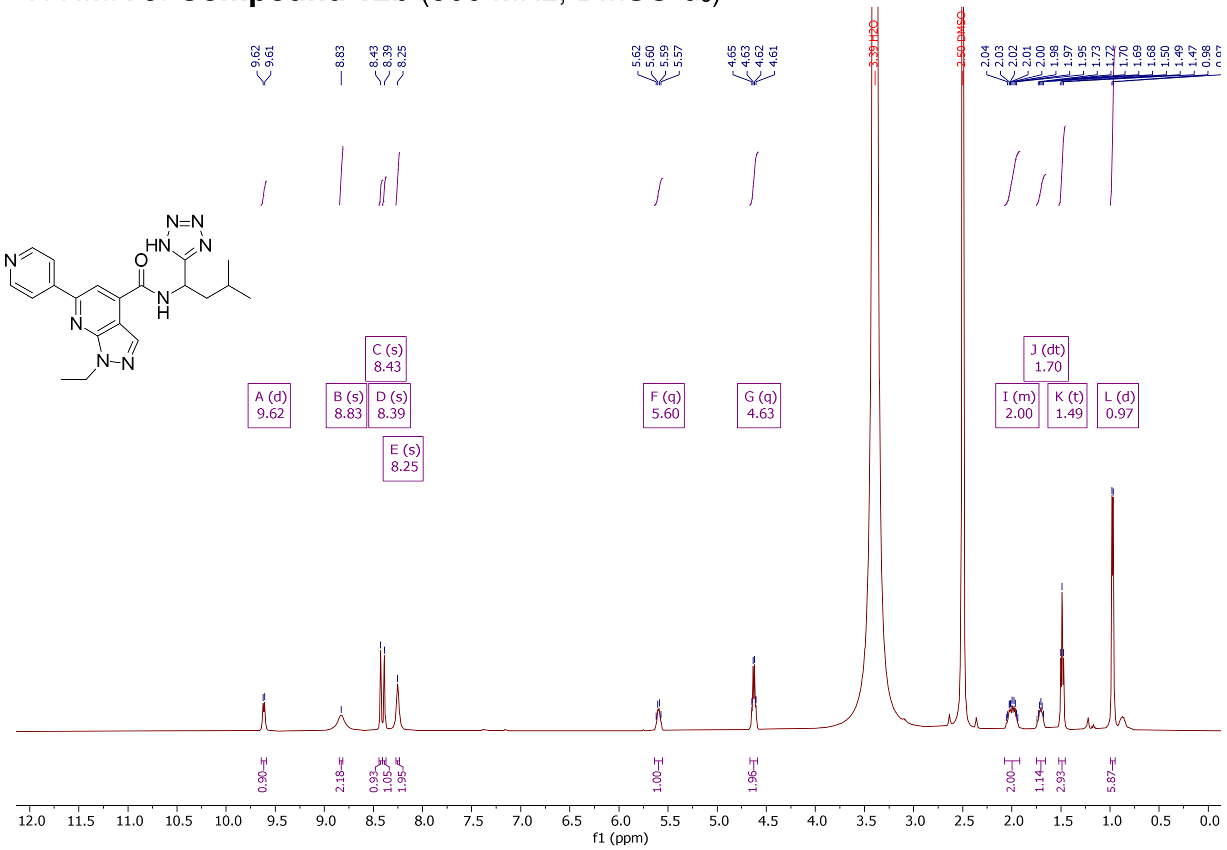

^{13}C NMR of **Compound 12b** (125 MHz, DMSO-d$_6$)

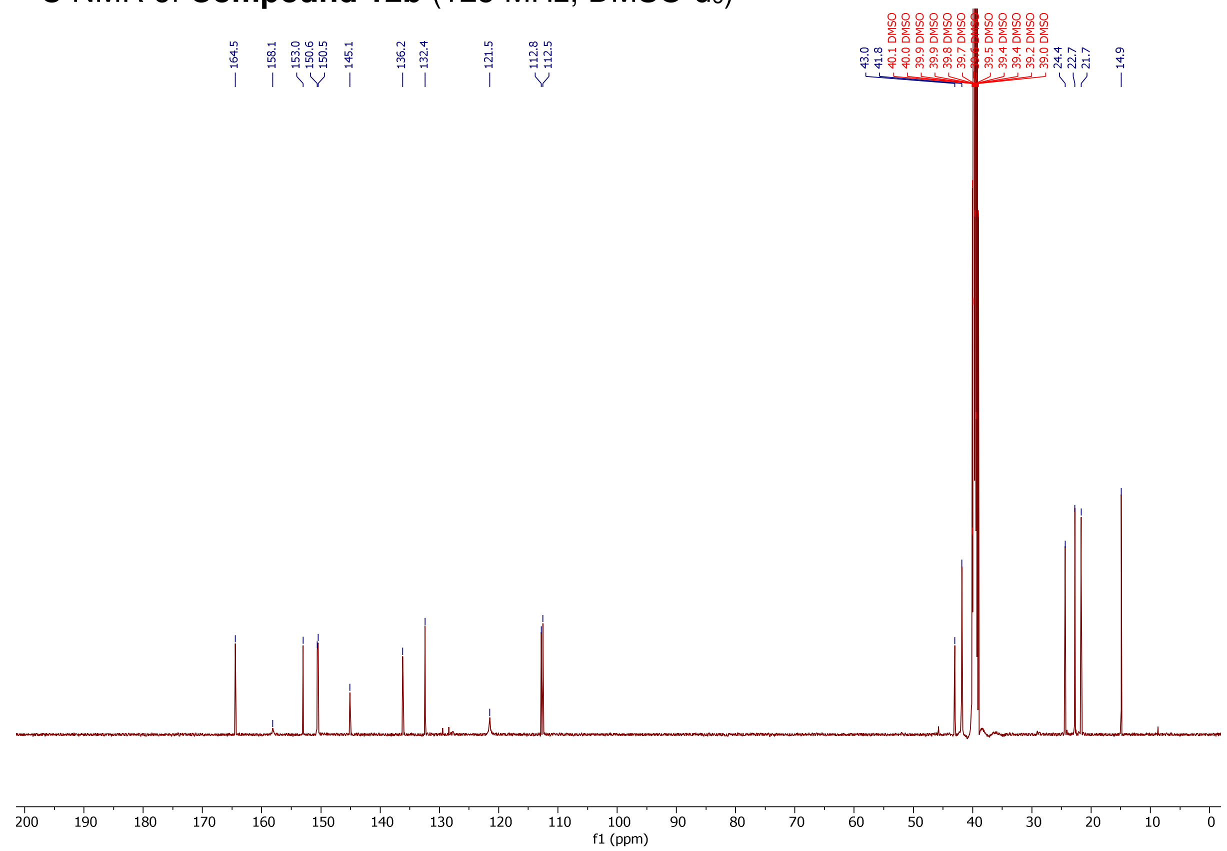

^{1}H NMR of **Compound 13b** (500 MHz, DMSO-d6)

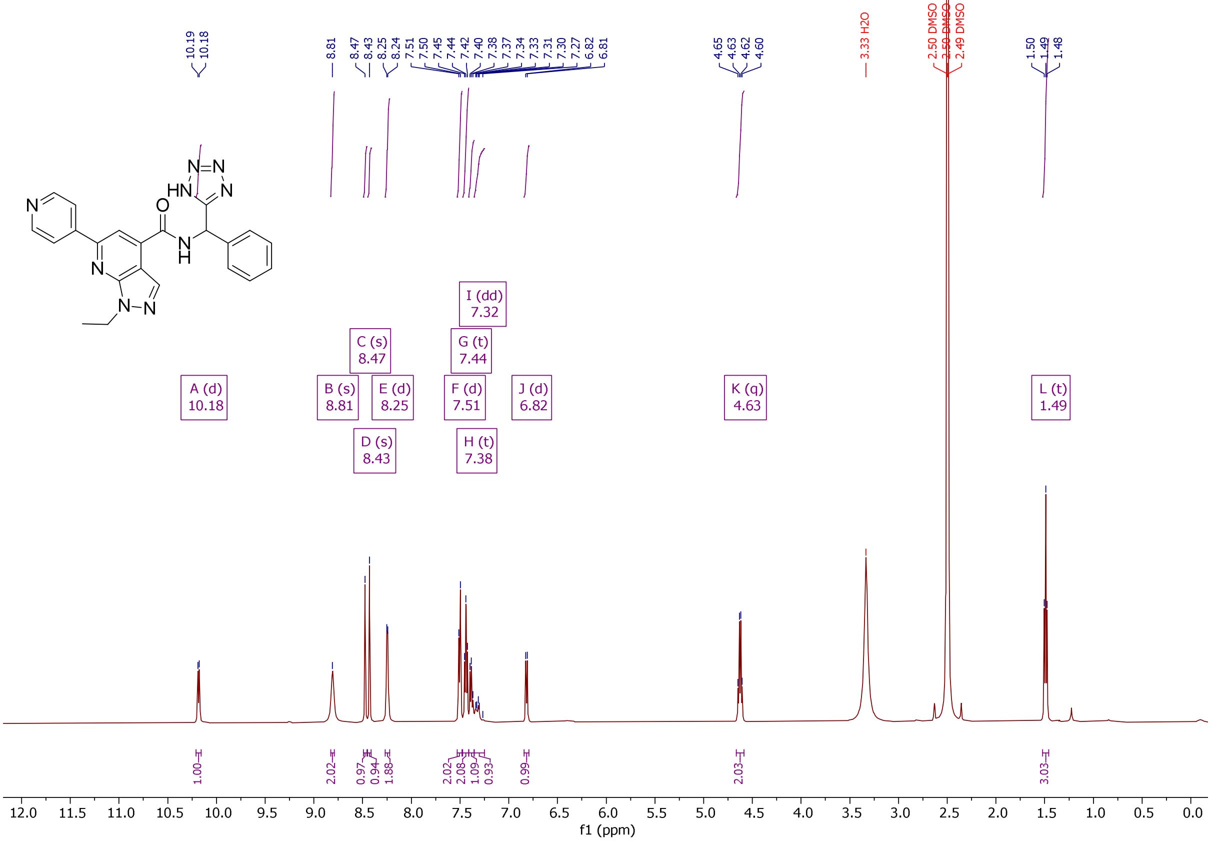

^{13}C NMR of **Compound 13b** (125 MHz, DMSO-d6)

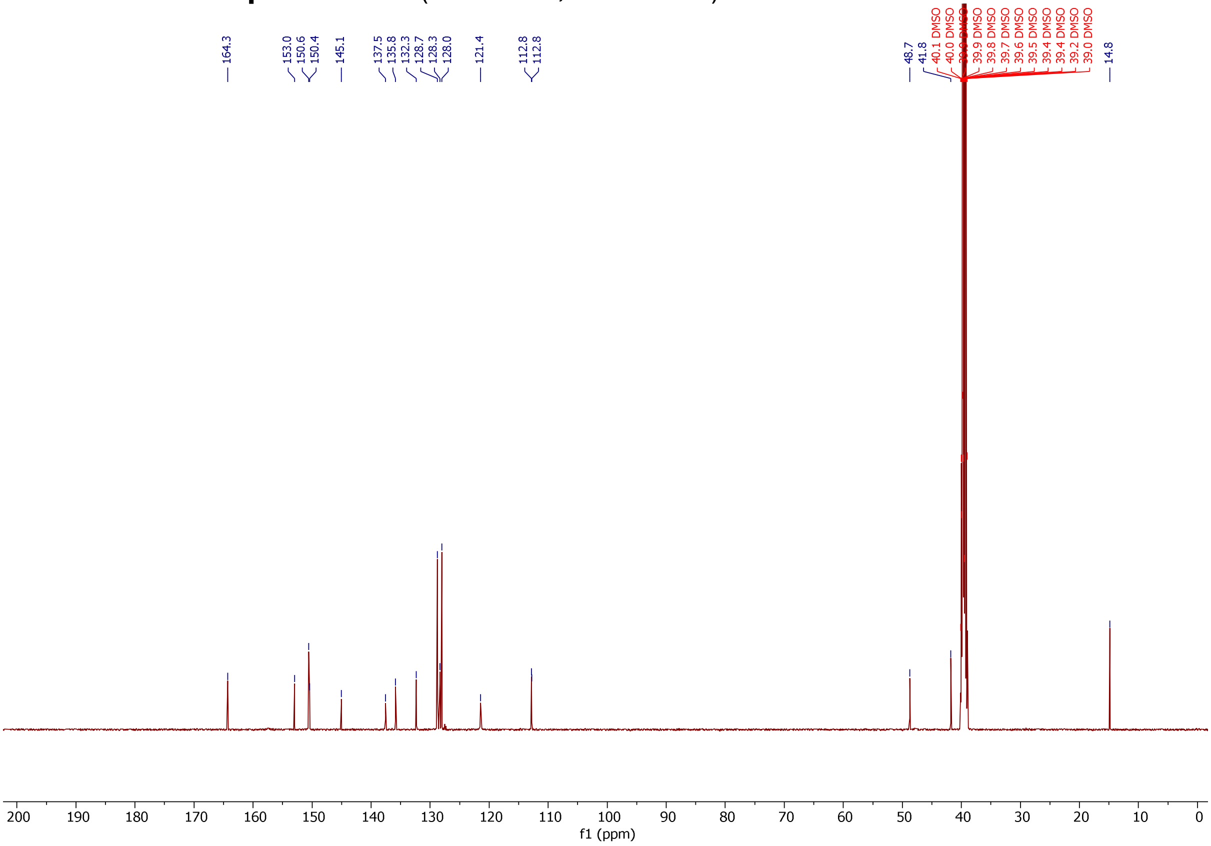

Compound 14b

MaxPeak: 100.00%
Ret_Time: 1.319 min

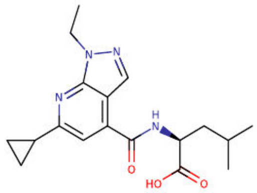

Mol Wt 344.41
Exact Mass 344.21

#	Time	Area%
1	1.319	100.00

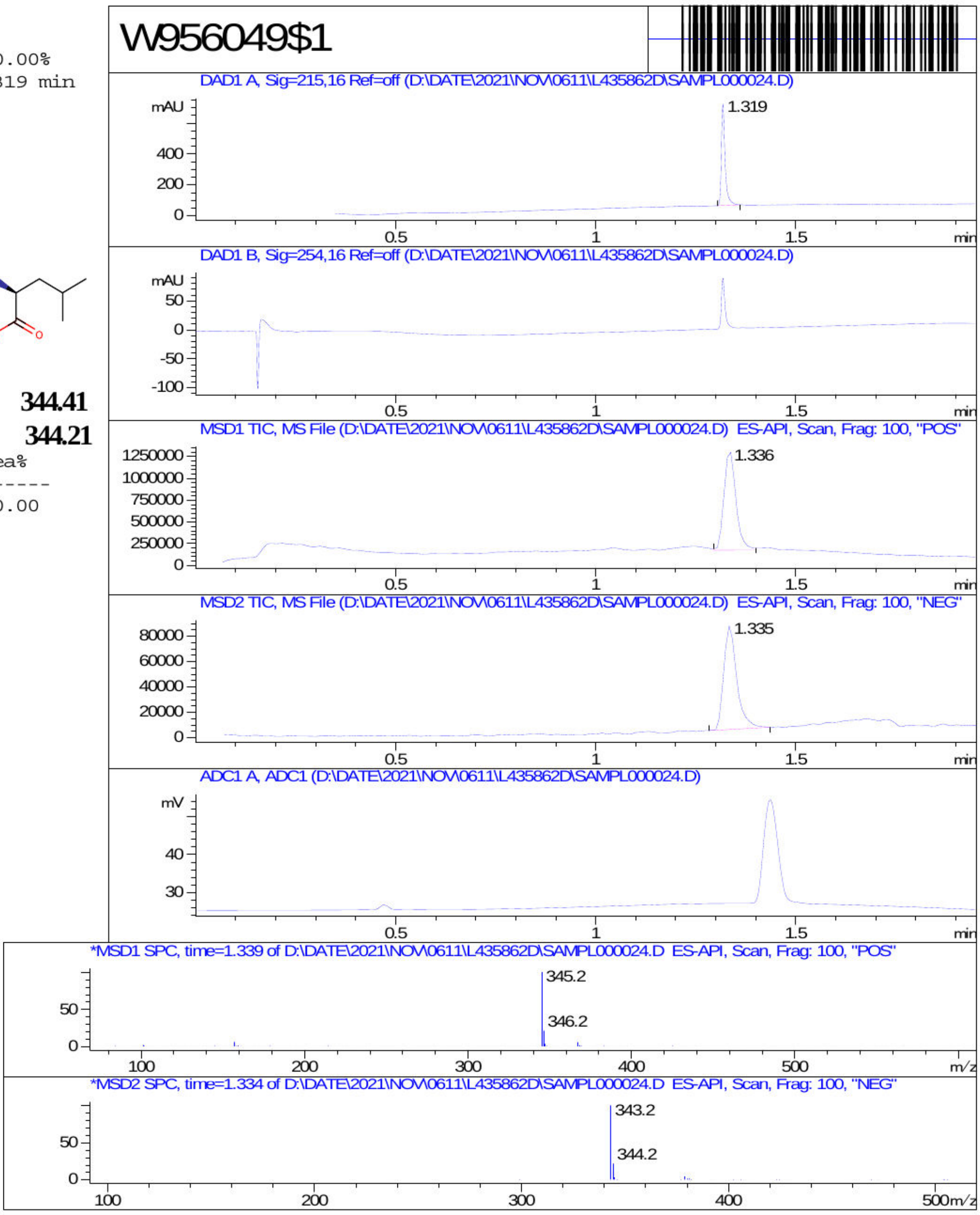

RT 1.336

RT 1.335

Inj.Date 05-Nov-21 A P2-C-06 - 4 - Acq. Method C:\CHEM32\-> ->

Compound 15b

MaxPeak: 94.34%
Ret_Time: 1.391 min

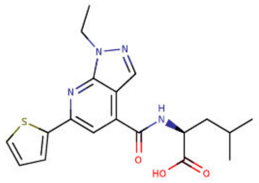

Mol Wt 386.47
Exact Mass 386.16

#	Time	Area%
1	1.391	94.34
2	1.434	3.17
3	1.509	2.49

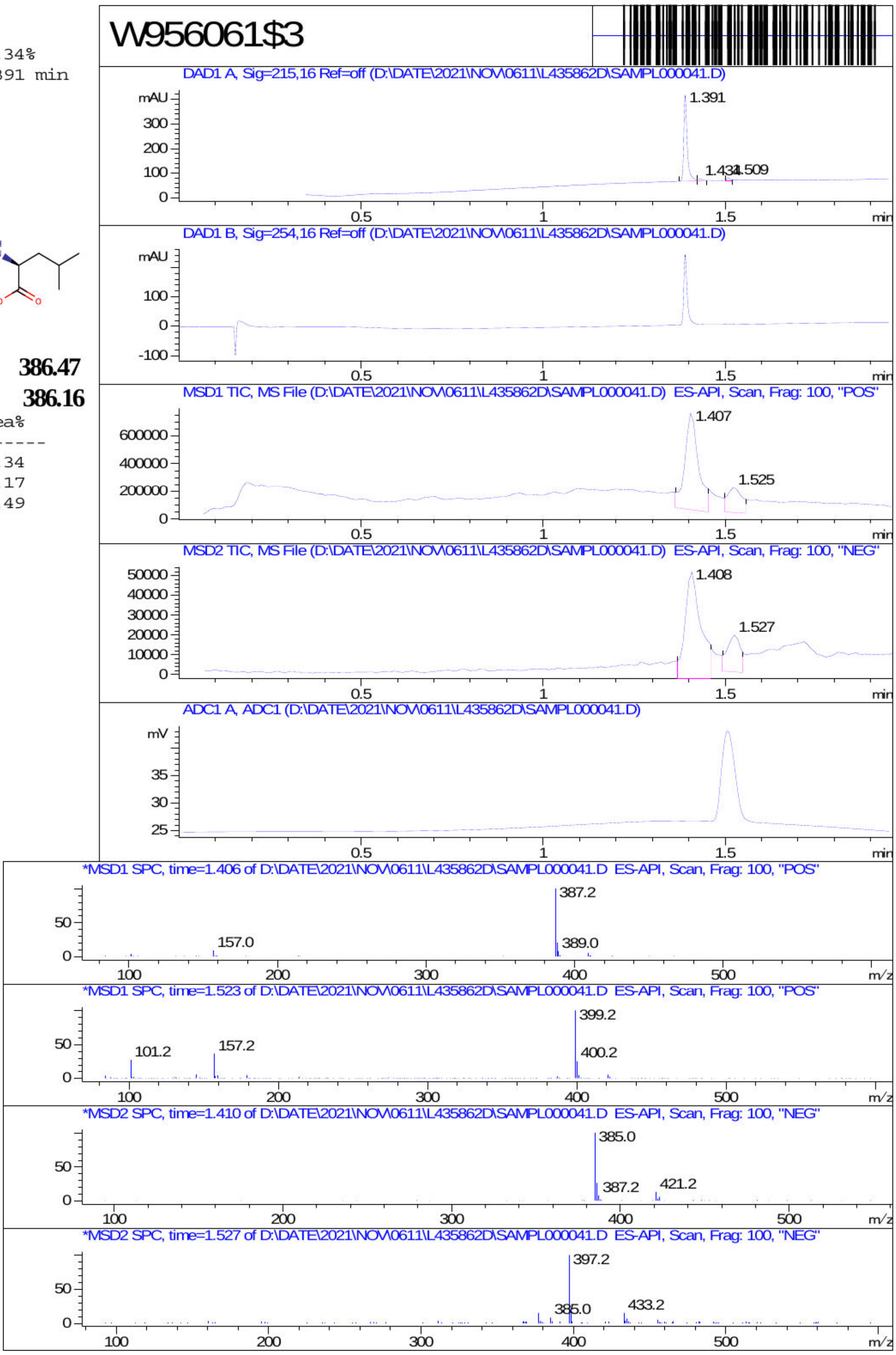

Inj.Date 06-Nov-21 A P2-E-06 - 4 - Acq. Method C:\CHEM32\-> ->

^{1}H NMR of **Compound 16b** (500 MHz, CD_3OD)

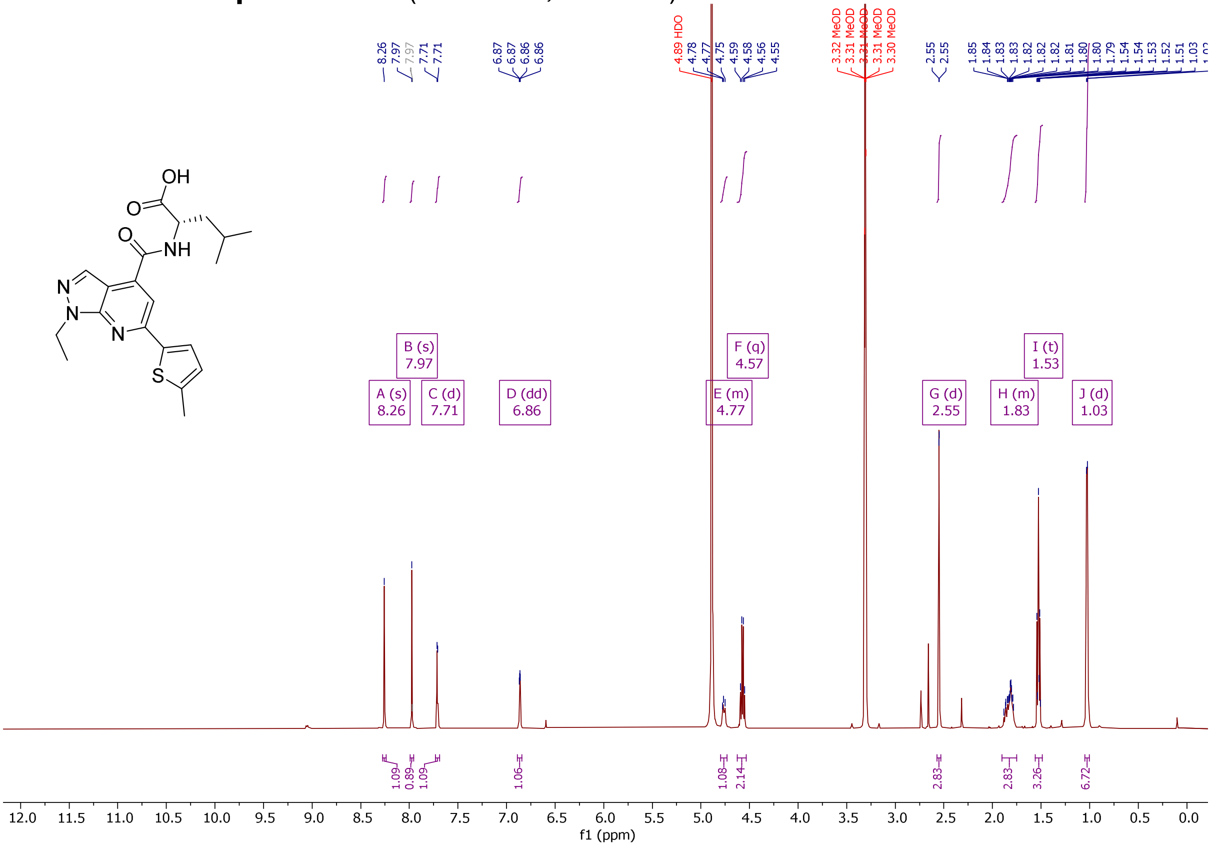

^{13}C NMR of **Compound 16b** (125 MHz, CD_3OD)

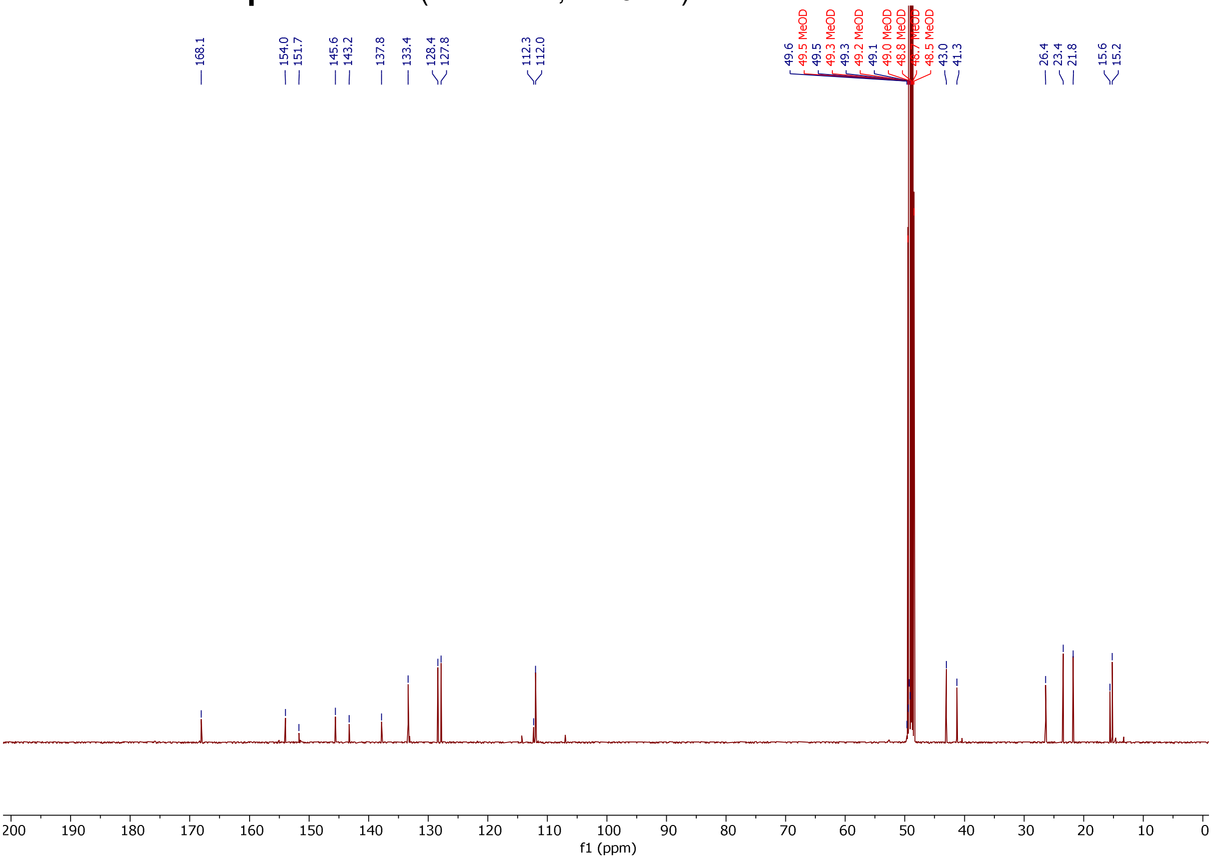

^{1}H NMR of **Compound 17b** (500 MHz, CD_3OD)

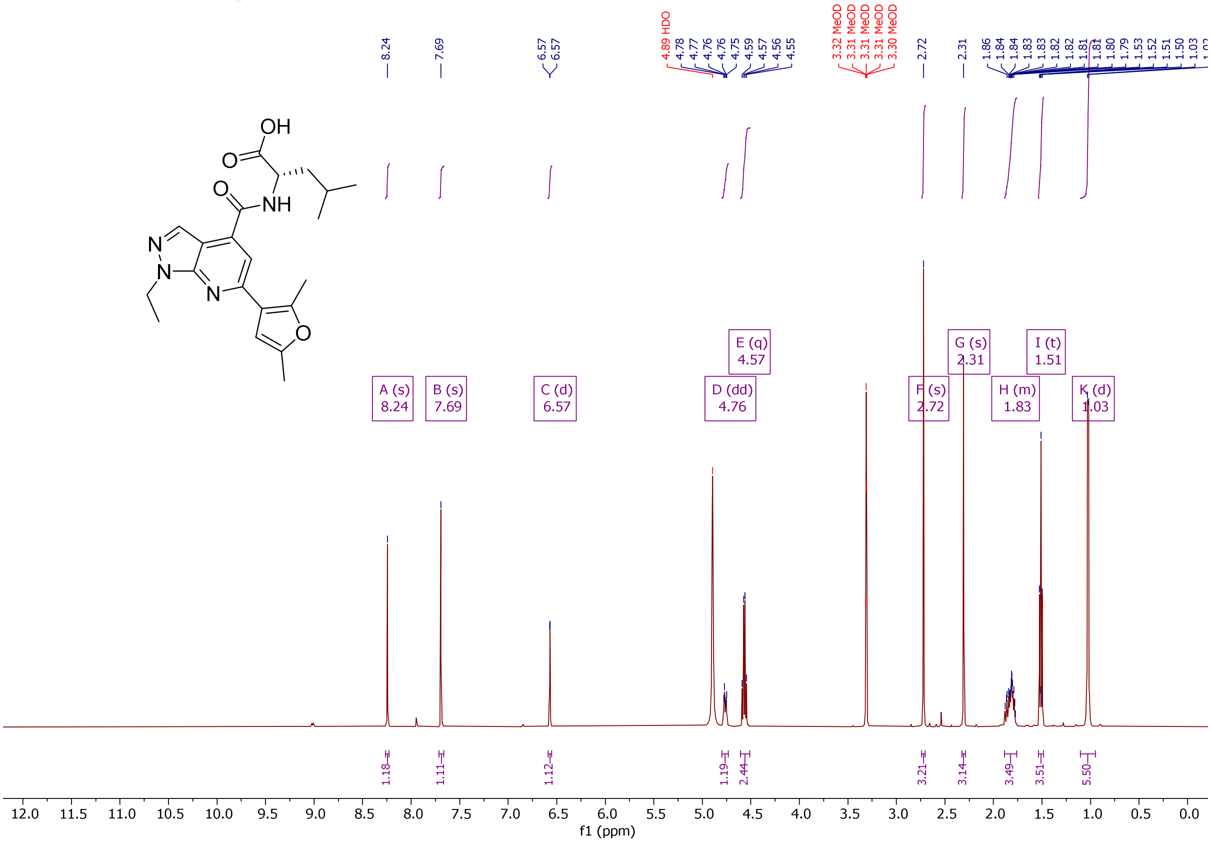

^{13}C NMR of **Compound 17b** (125 MHz, CD_3OD)

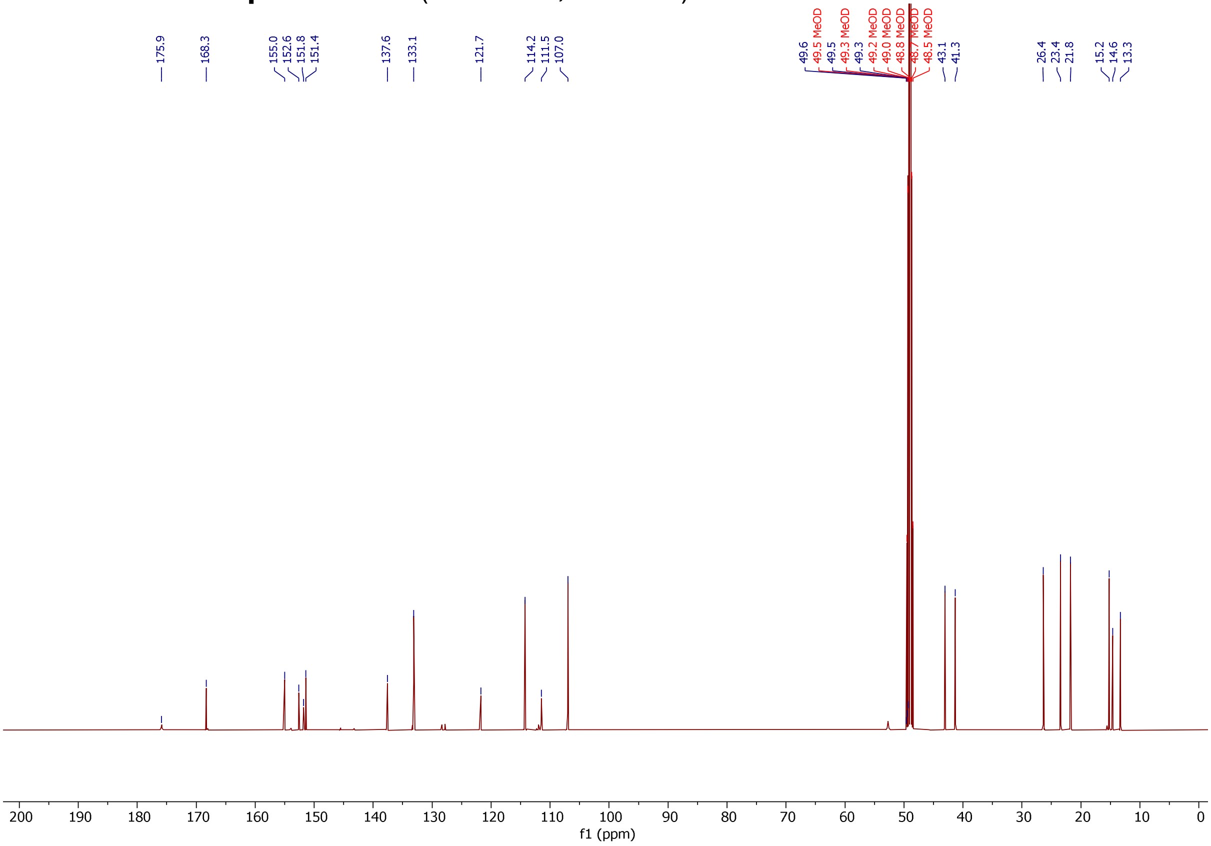

Compound 18b

MaxPeak: 100.00%
Ret_Time: 1.409 min

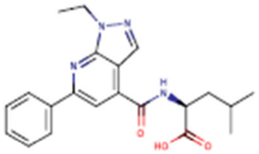

Mol Wt 380.44
Exact Mass 380.21

#	Time	Area%
1	1.409	100.00

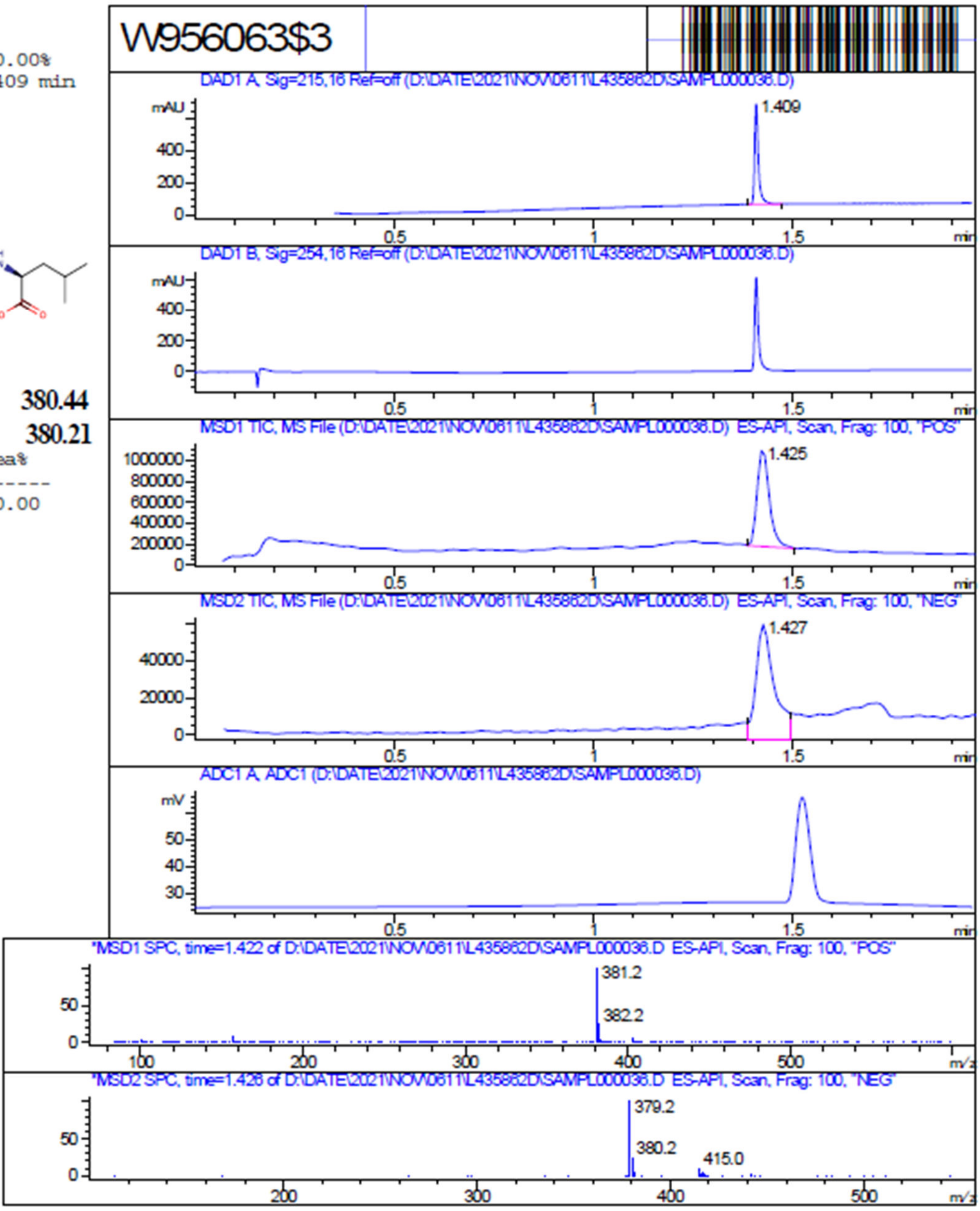

RT 1.425

RT 1.427

Inj.Date 05-Nov-21 A P2-E-01 - 4 - Acq. Method C:\CHEM32\-> ->

^{1}H NMR of **Compound 19b** (600 MHz, CD_3OD)

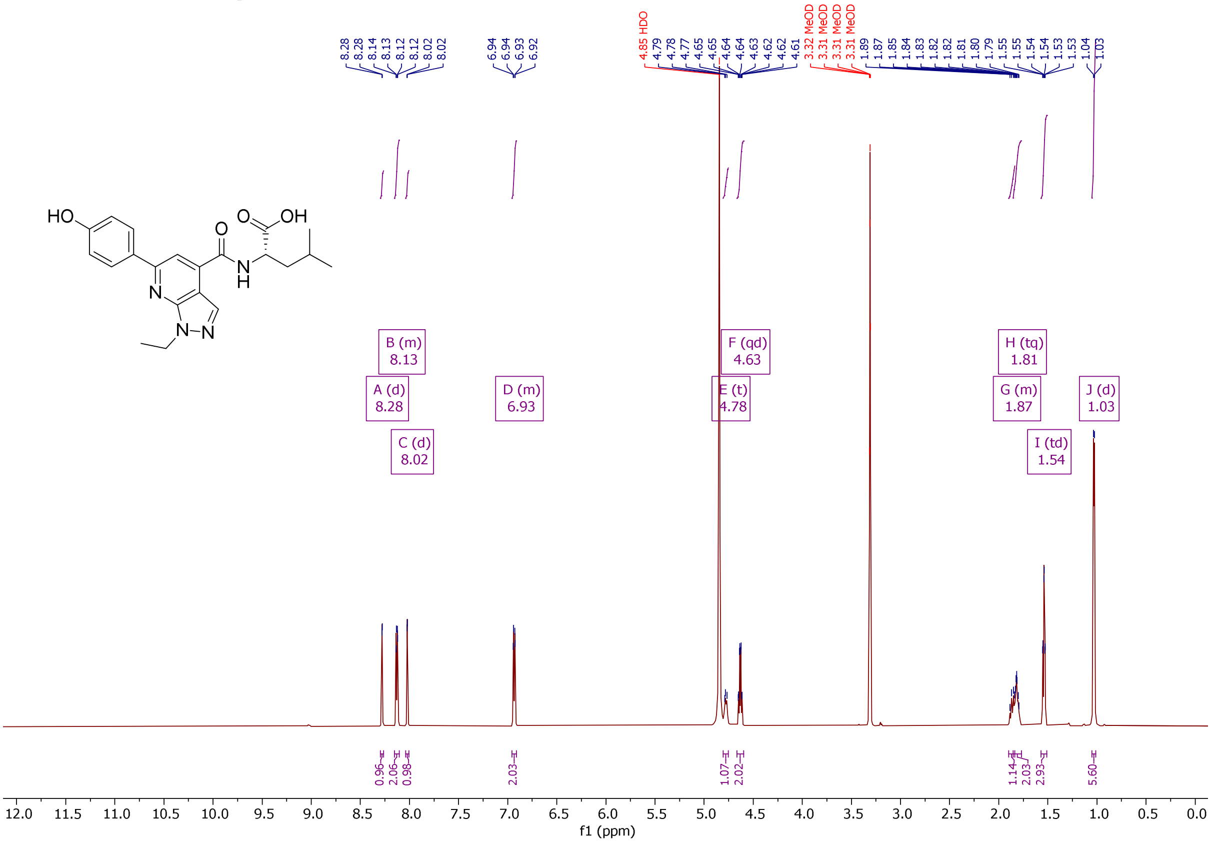

^{13}C NMR of **Compound 19b** (150 MHz, CD_3OD)

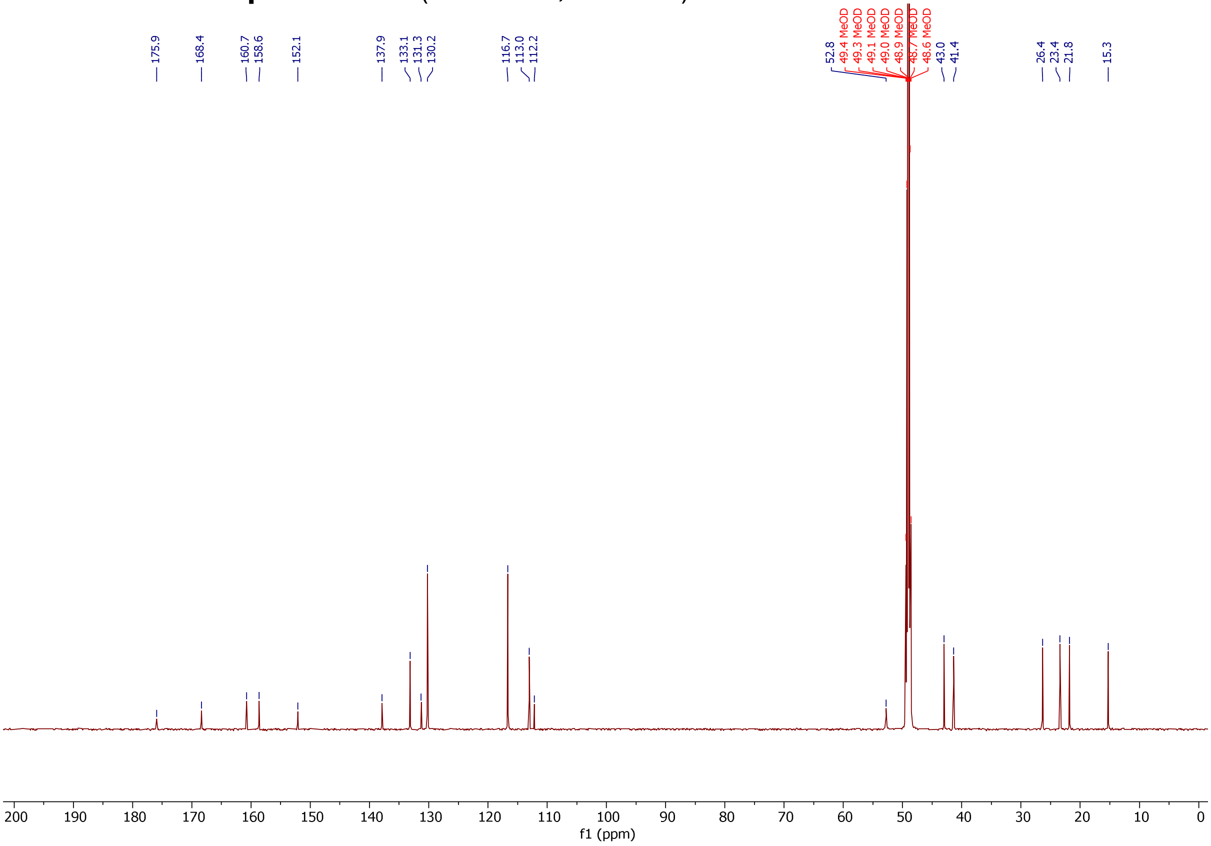

^{1}H NMR of **Compound 20b** (500 MHz, CD_3OD)

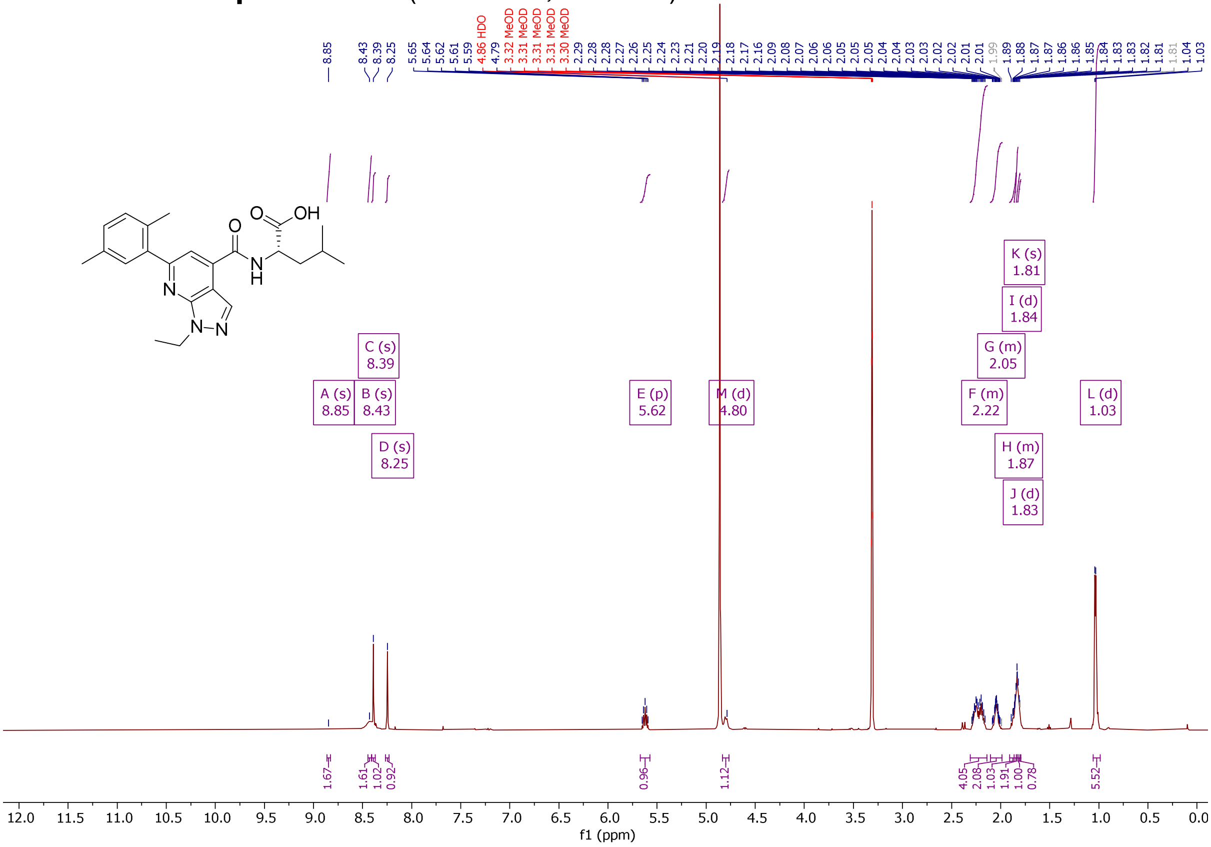

^{13}C NMR of **Compound 20b** (125 MHz, CD_3OD)

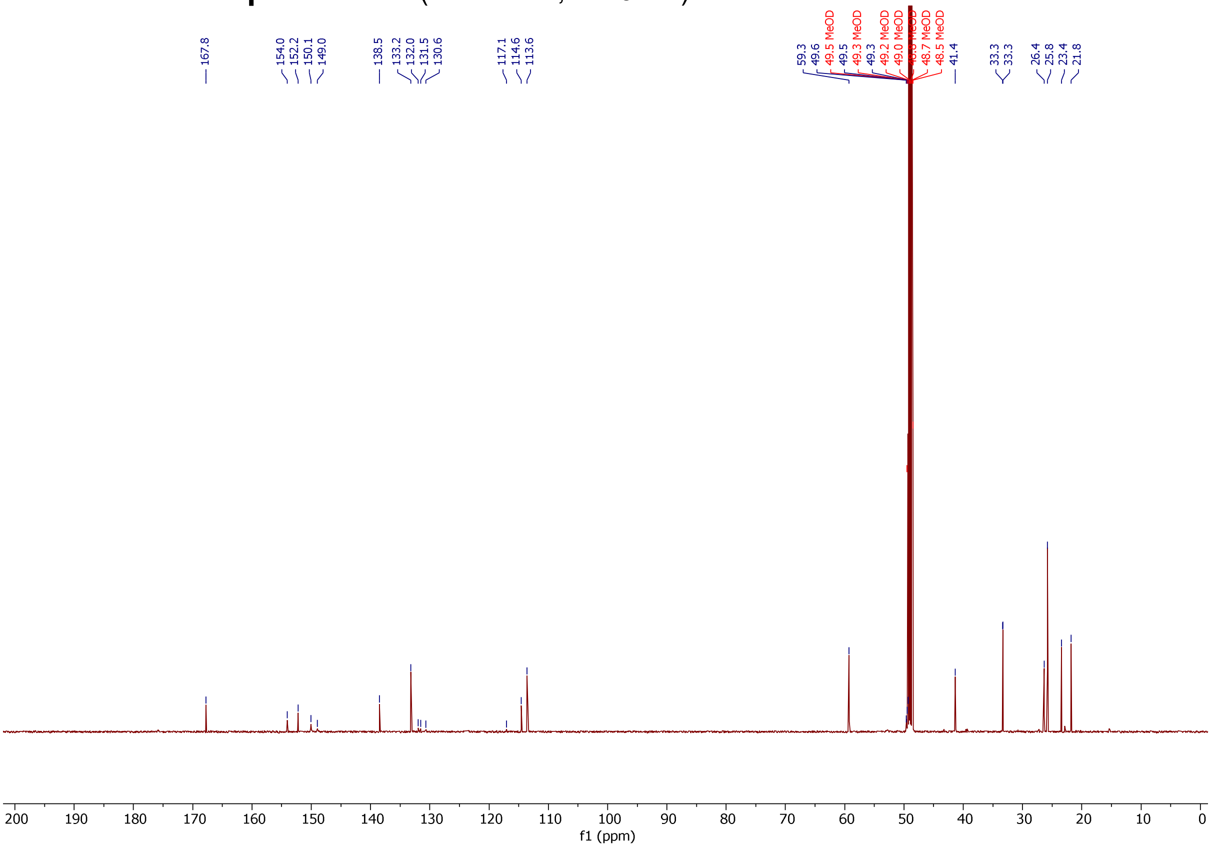

^{1}H NMR of **Compound 21b** (500 MHz, CD3OD)

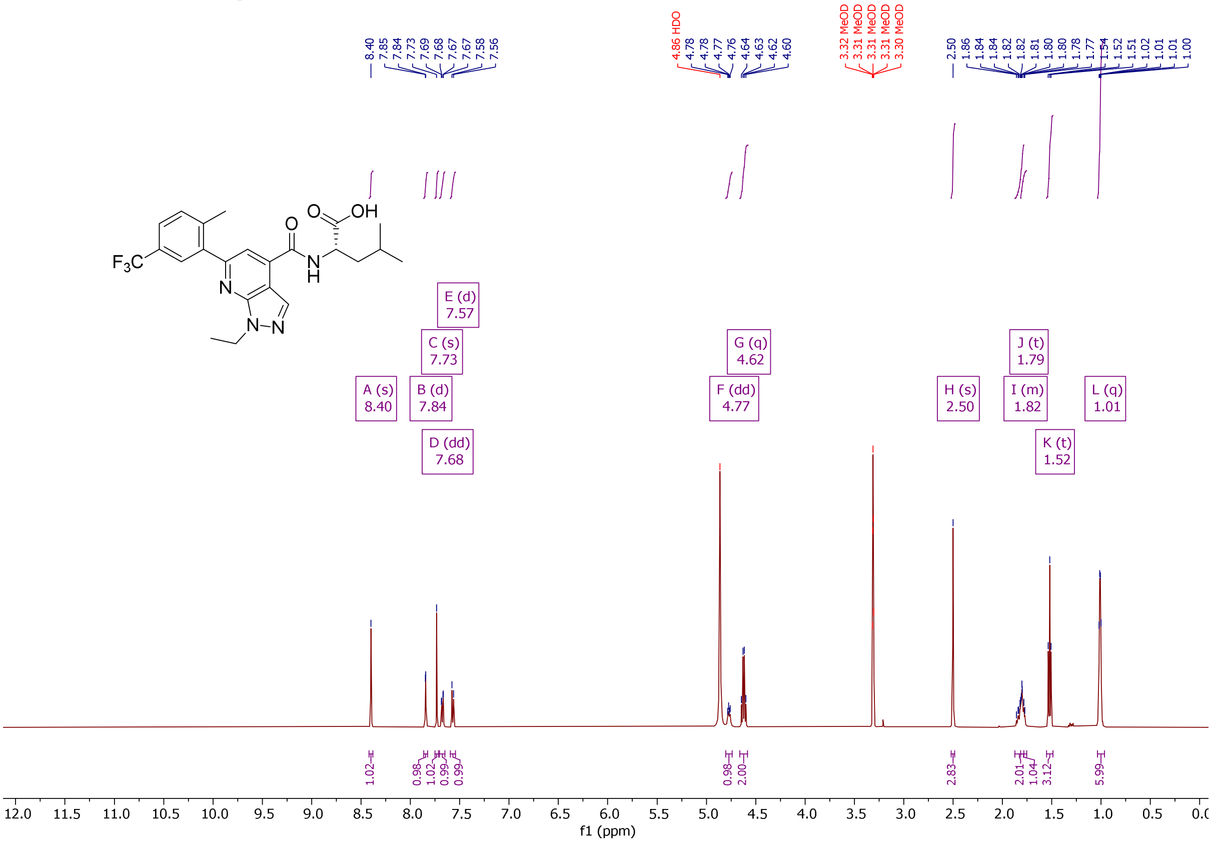

^{13}C NMR of **Compound 21b** (125 MHz, CD3OD)

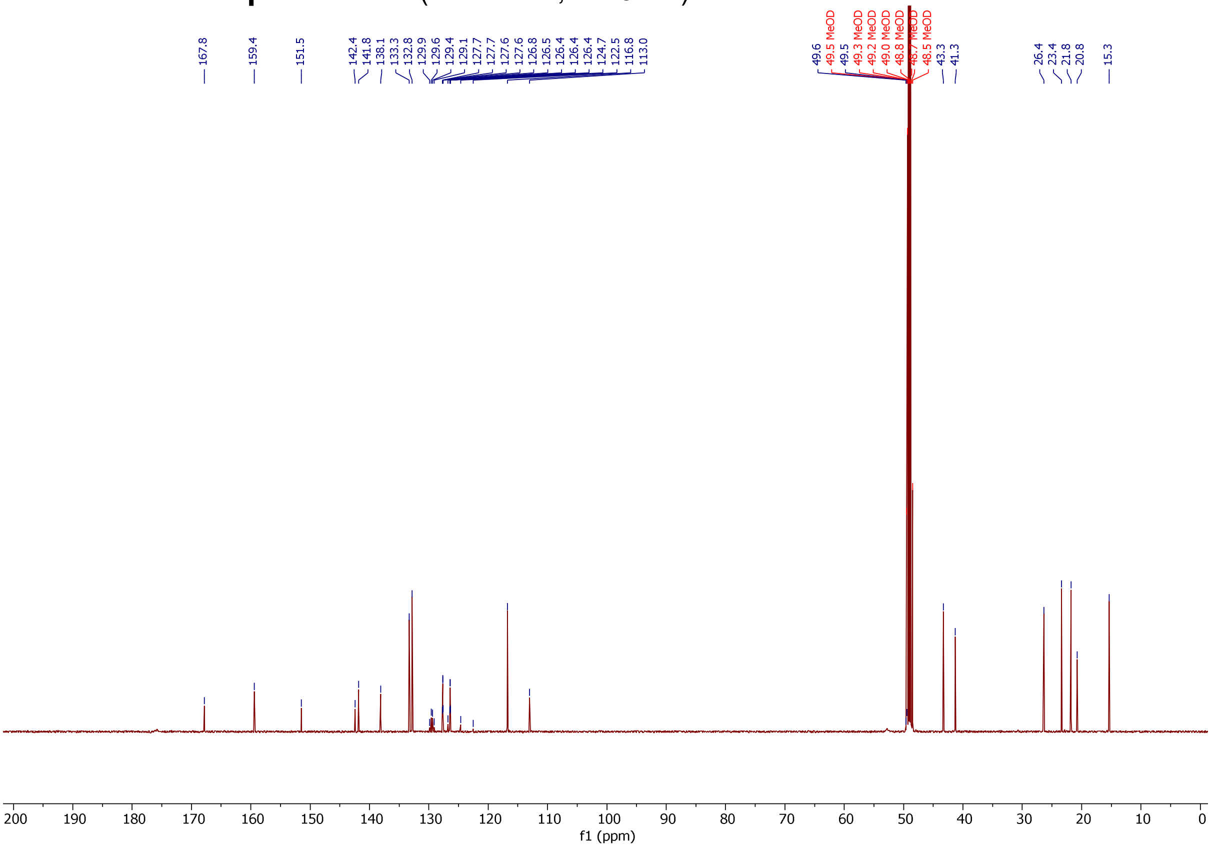

^{1}H NMR of **Compound 22b** (500 MHz, CD_3OD)

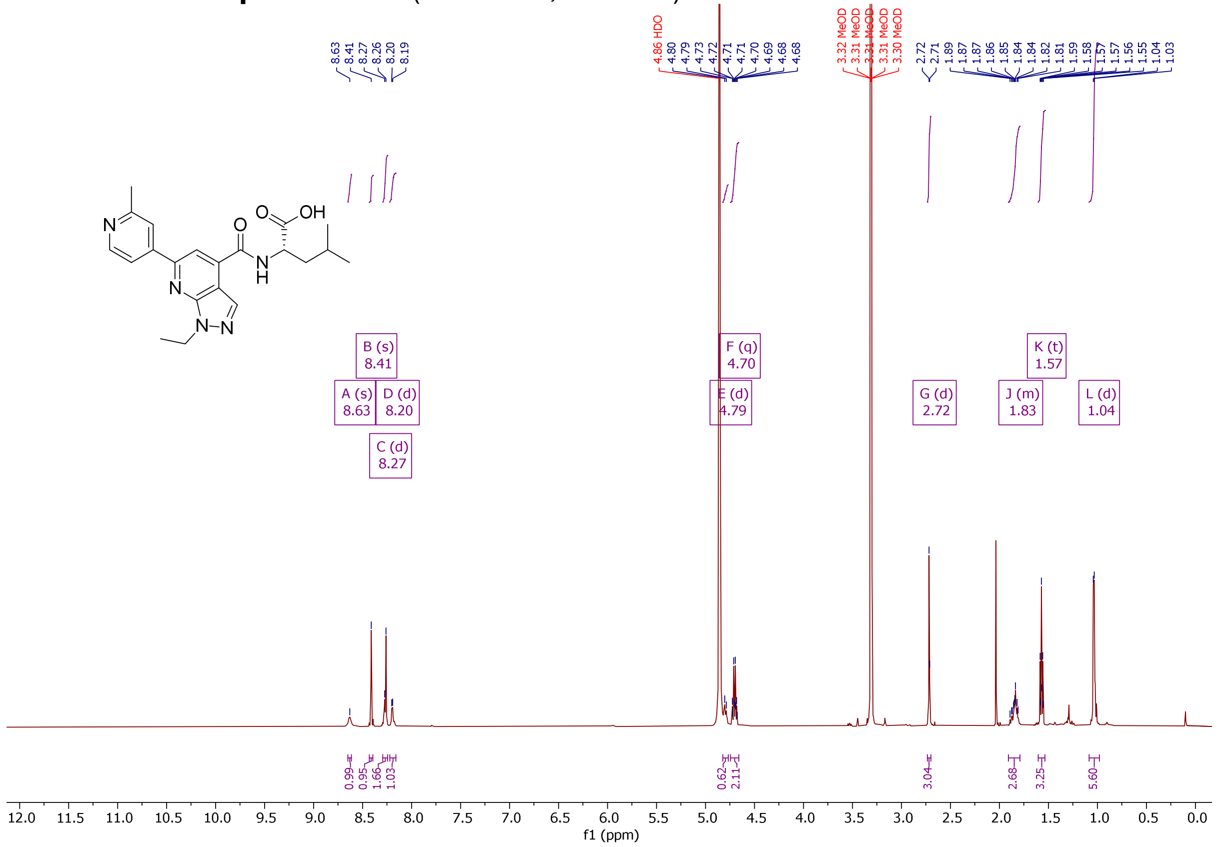

^{13}C NMR of **Compound 22b** (125 MHz, CD_3OD)

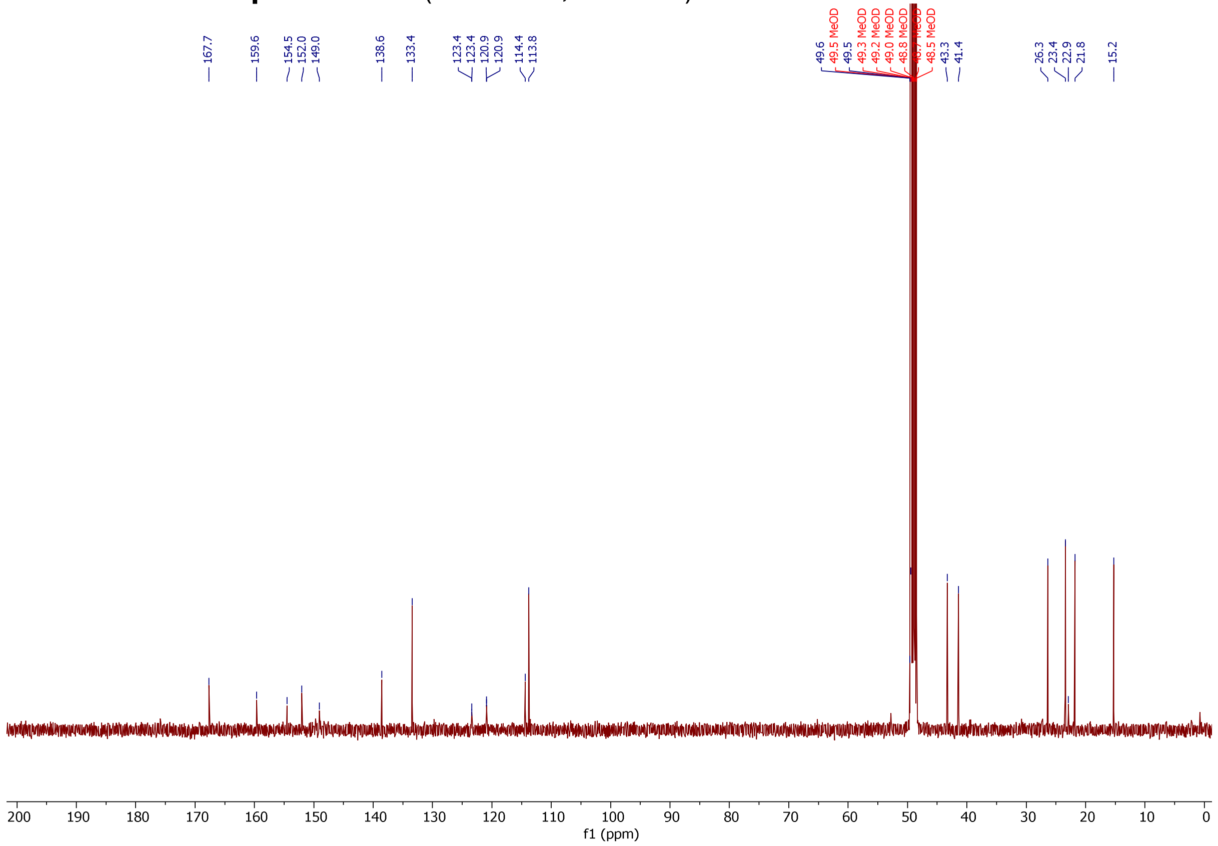

^{1}H NMR of **Compound 23b** (600 MHz, CD_3OD)

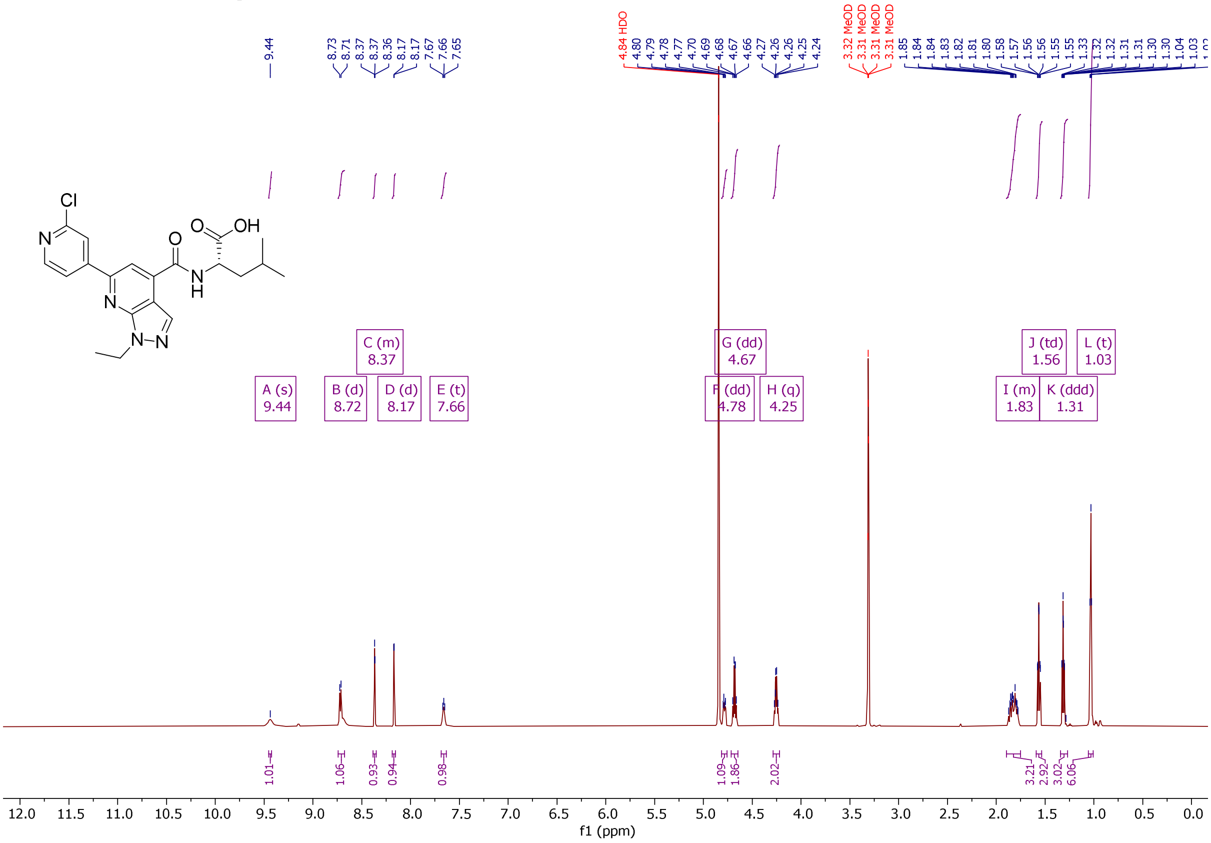

^{13}C NMR of **Compound 23b** (150 MHz, CD_3OD)

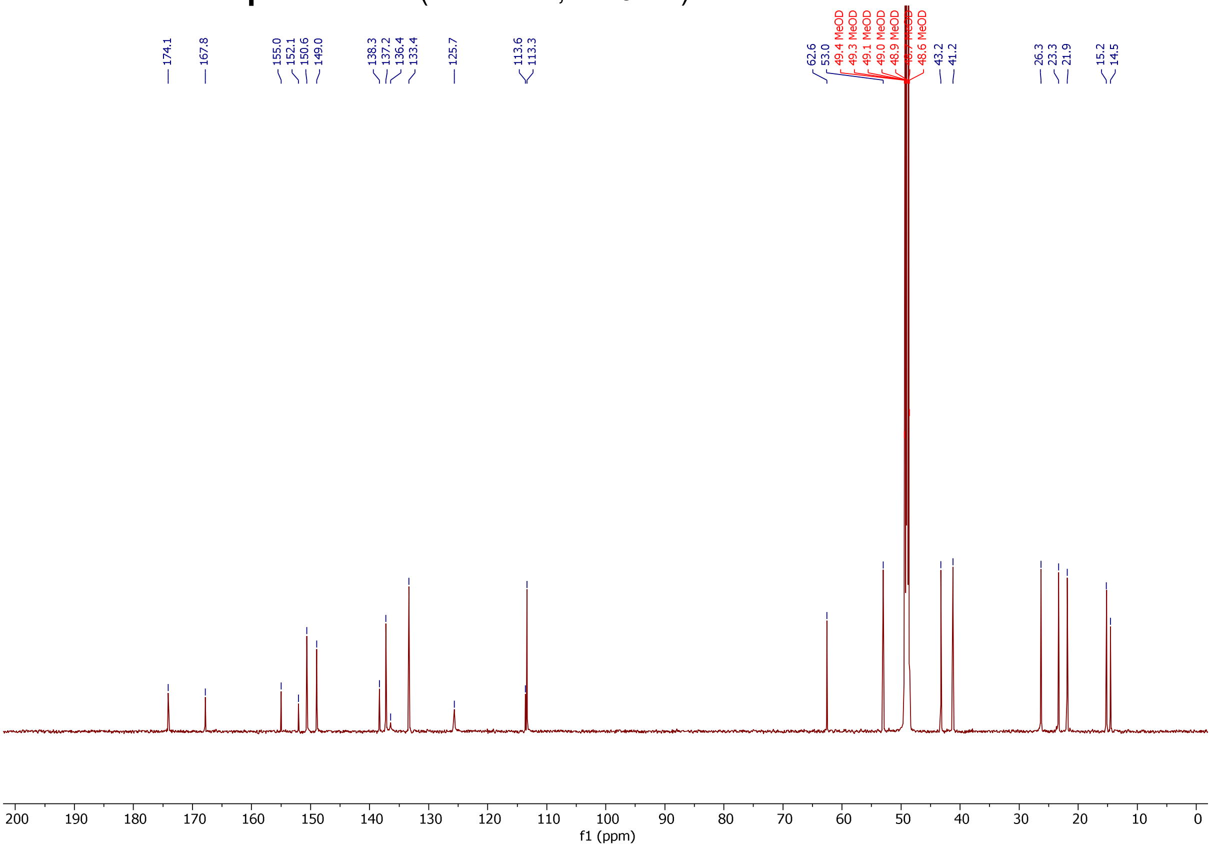

^{1}H NMR of **Compound 24b** (500 MHz, CD_3OD)

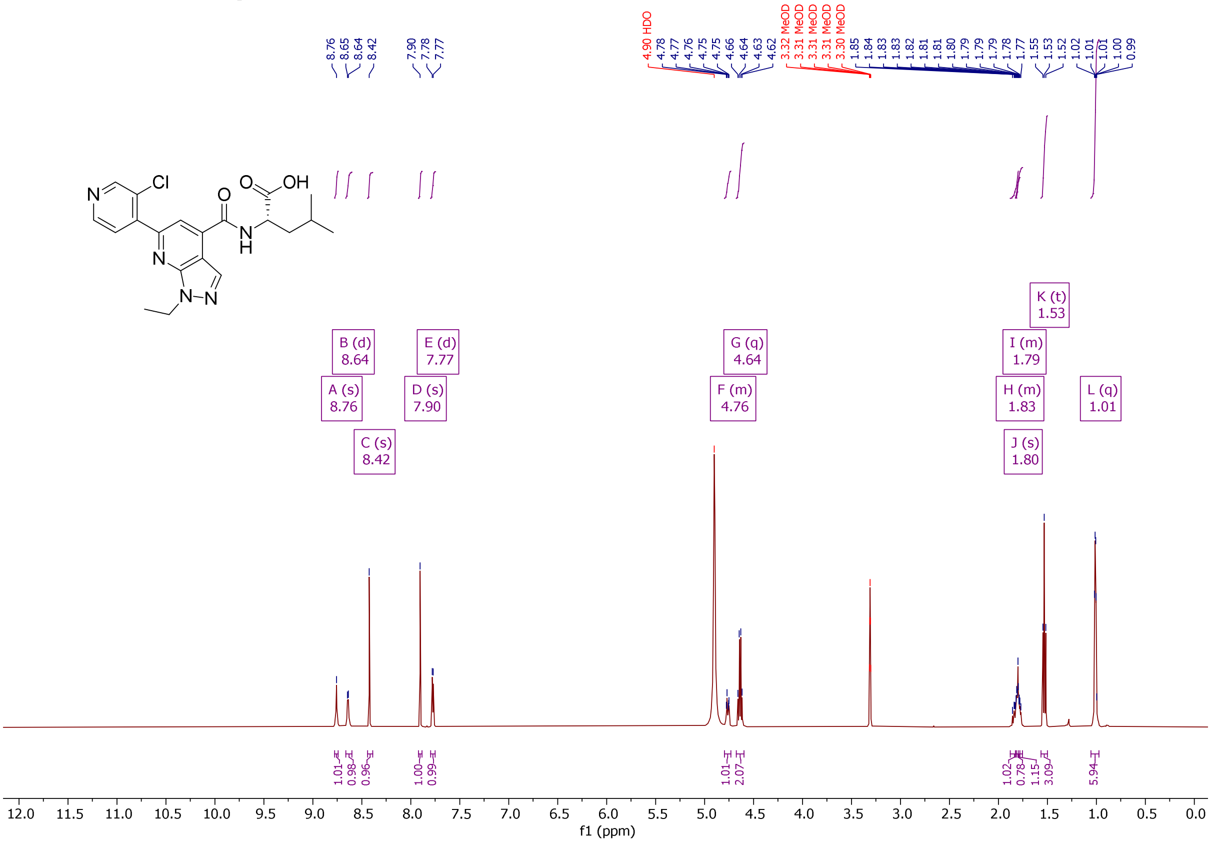

^{13}C NMR of **Compound 24b** (125 MHz, CD_3OD)

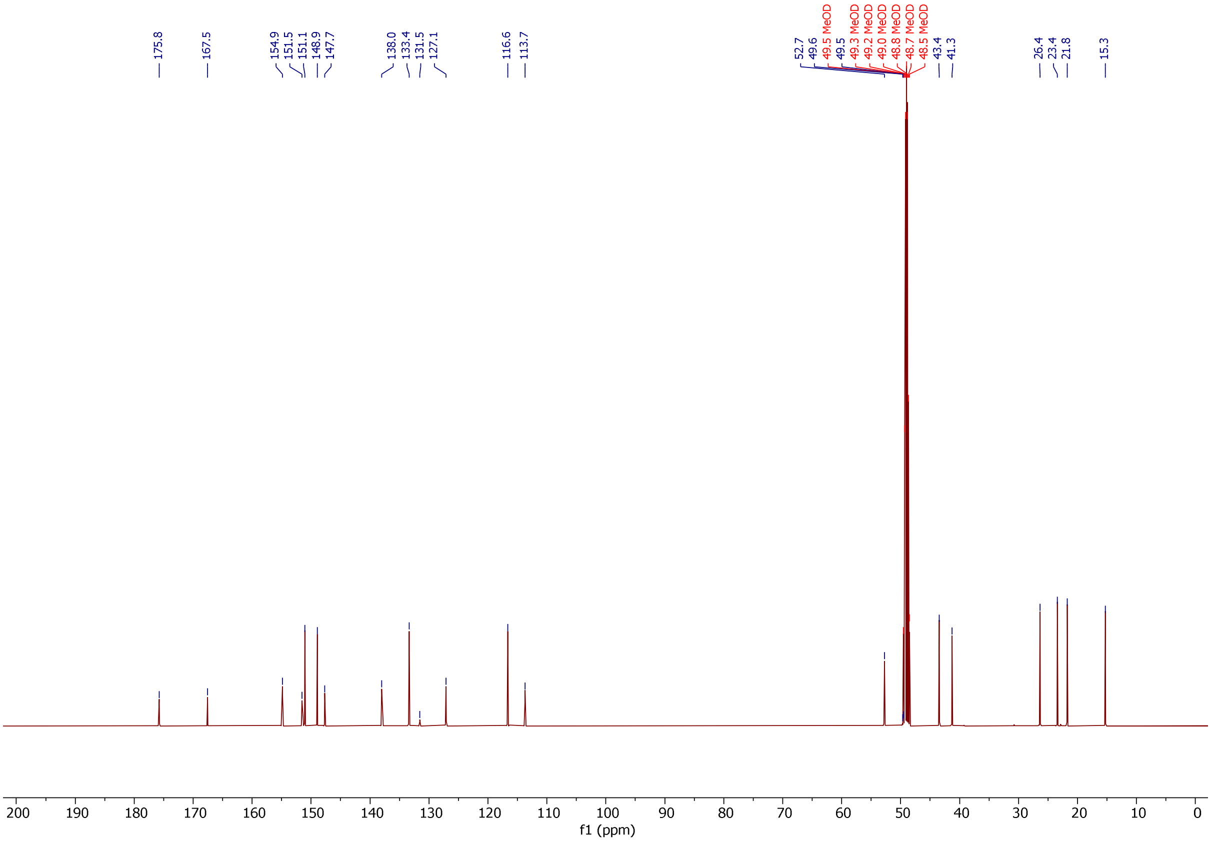

^{1}H NMR of **Compound 25b** (500 MHz, CD_3OD)

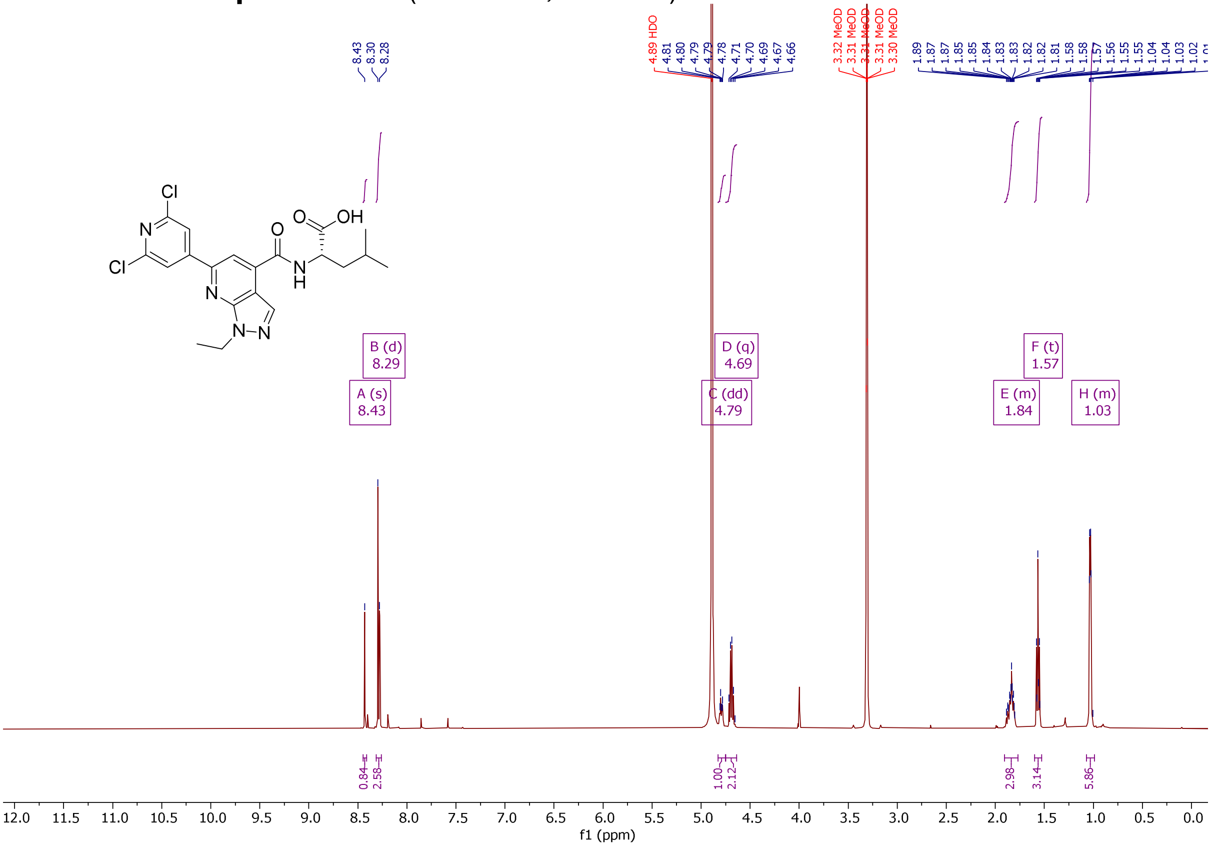

^{13}C NMR of **Compound 25b** (125 MHz, CD_3OD)

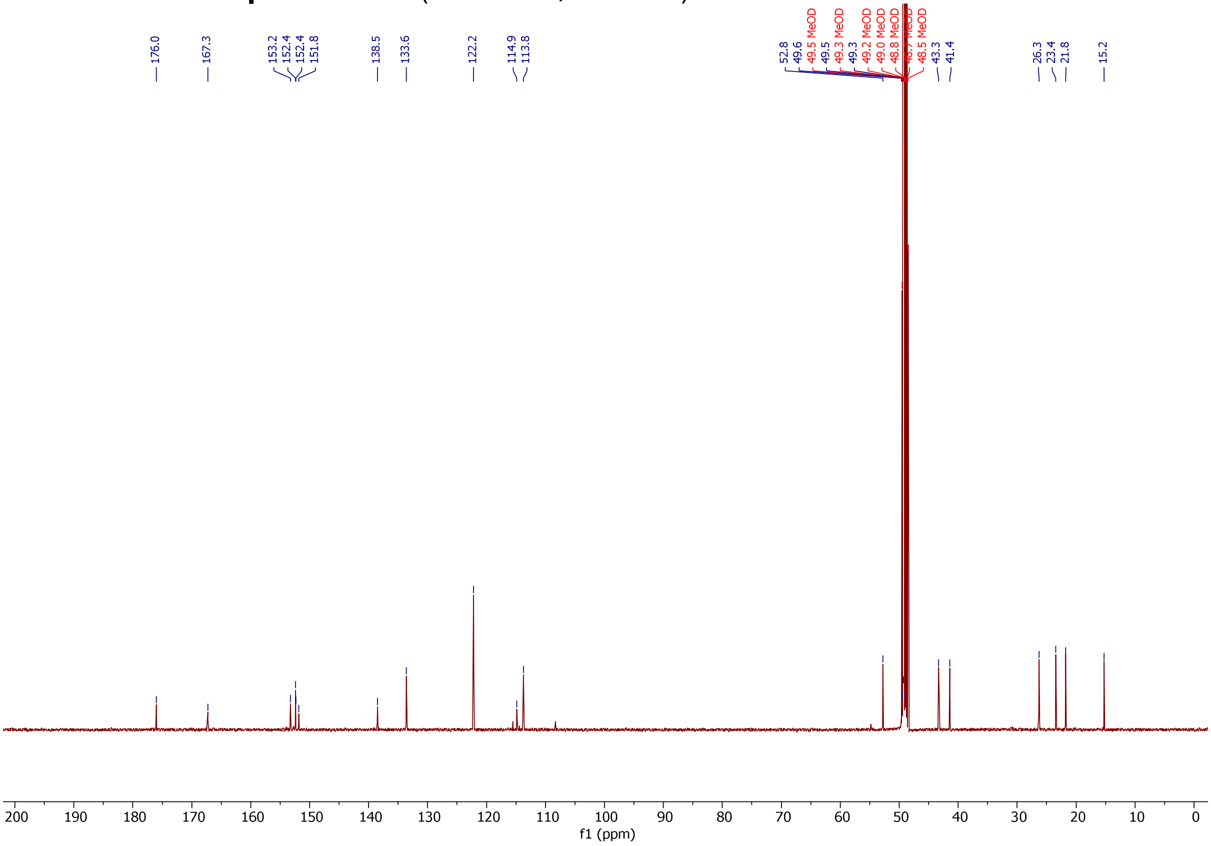

^{1}H NMR of **Compound 26b** (500 MHz, CD_3OD)

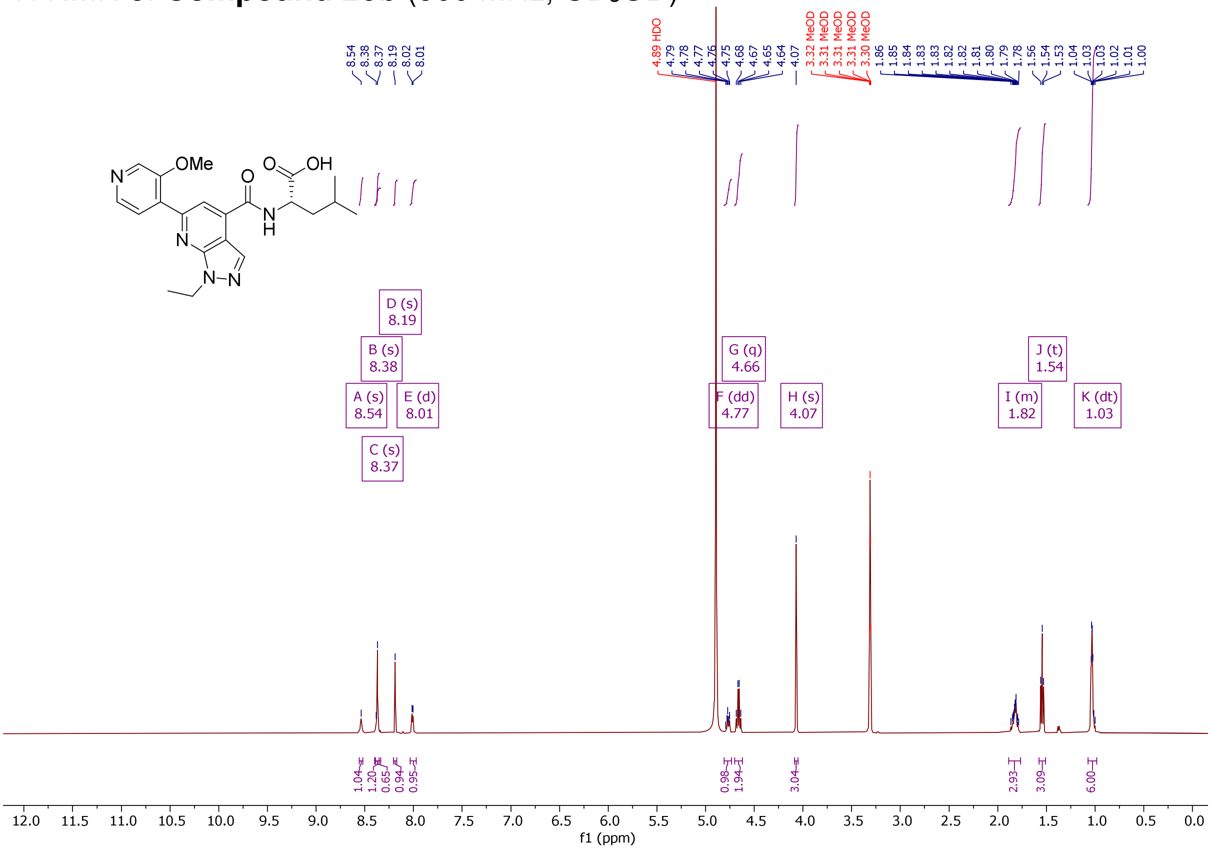

^{13}C NMR of **Compound 26b** (125 MHz, CD_3OD)

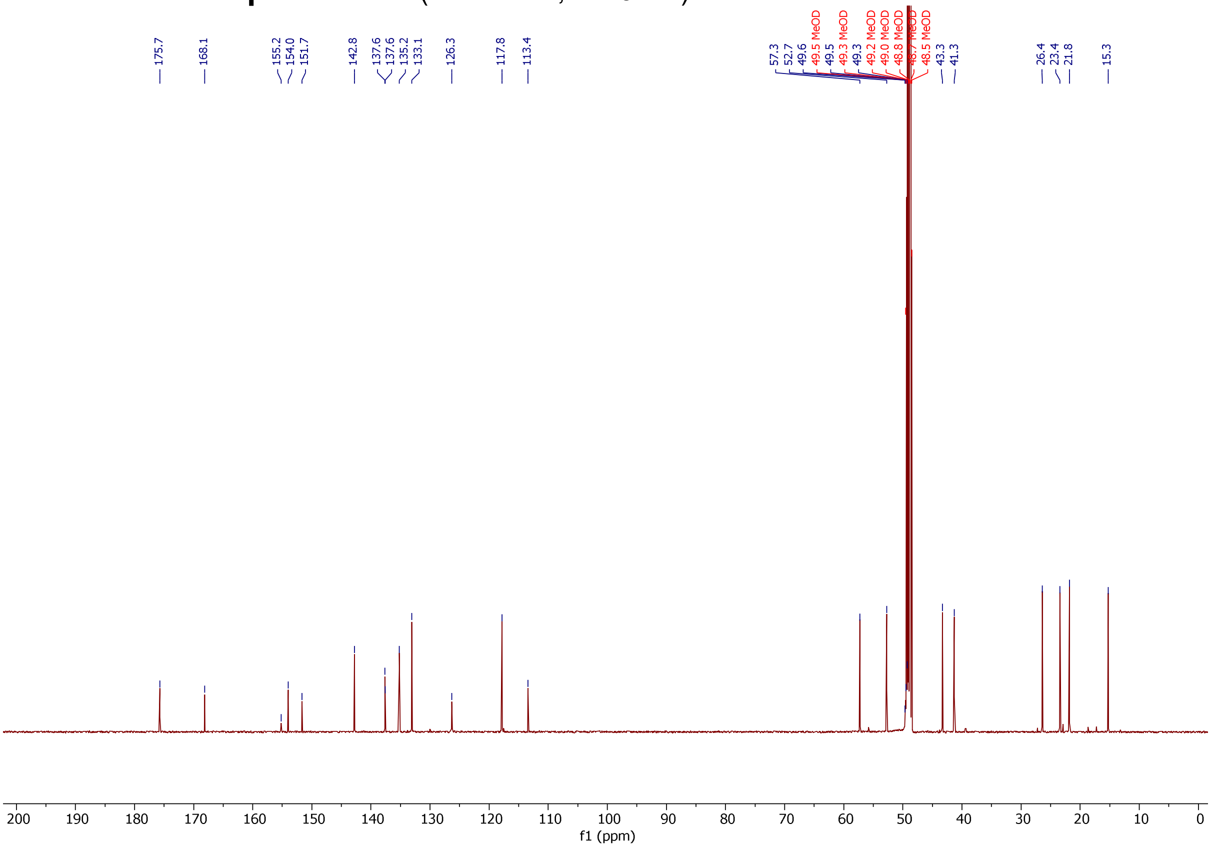

^{1}H NMR of **Compound 27b** (600 MHz, CD_3OD)

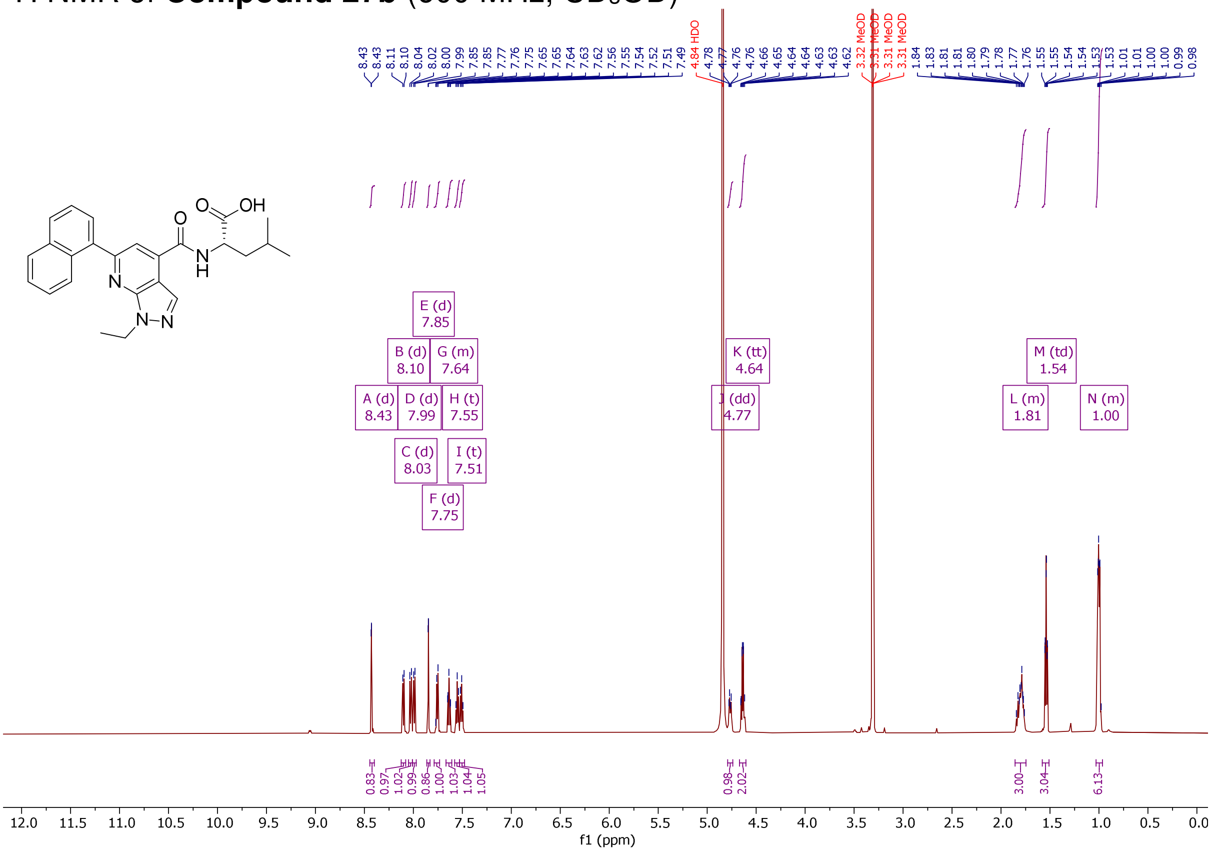

^{13}C NMR of **Compound 27b** (150 MHz, CD_3OD)

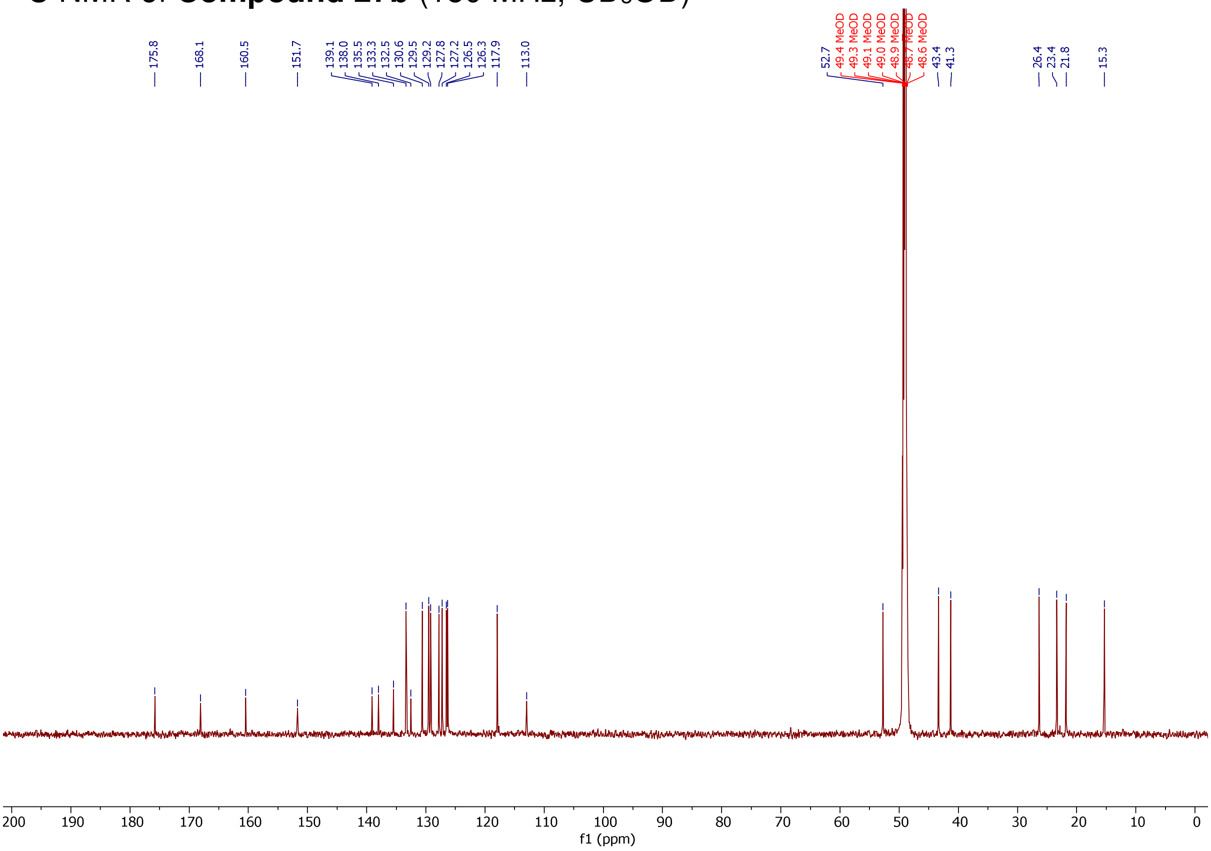

^{1}H NMR of **Compound 28b** (600 MHz, CD_3OD)

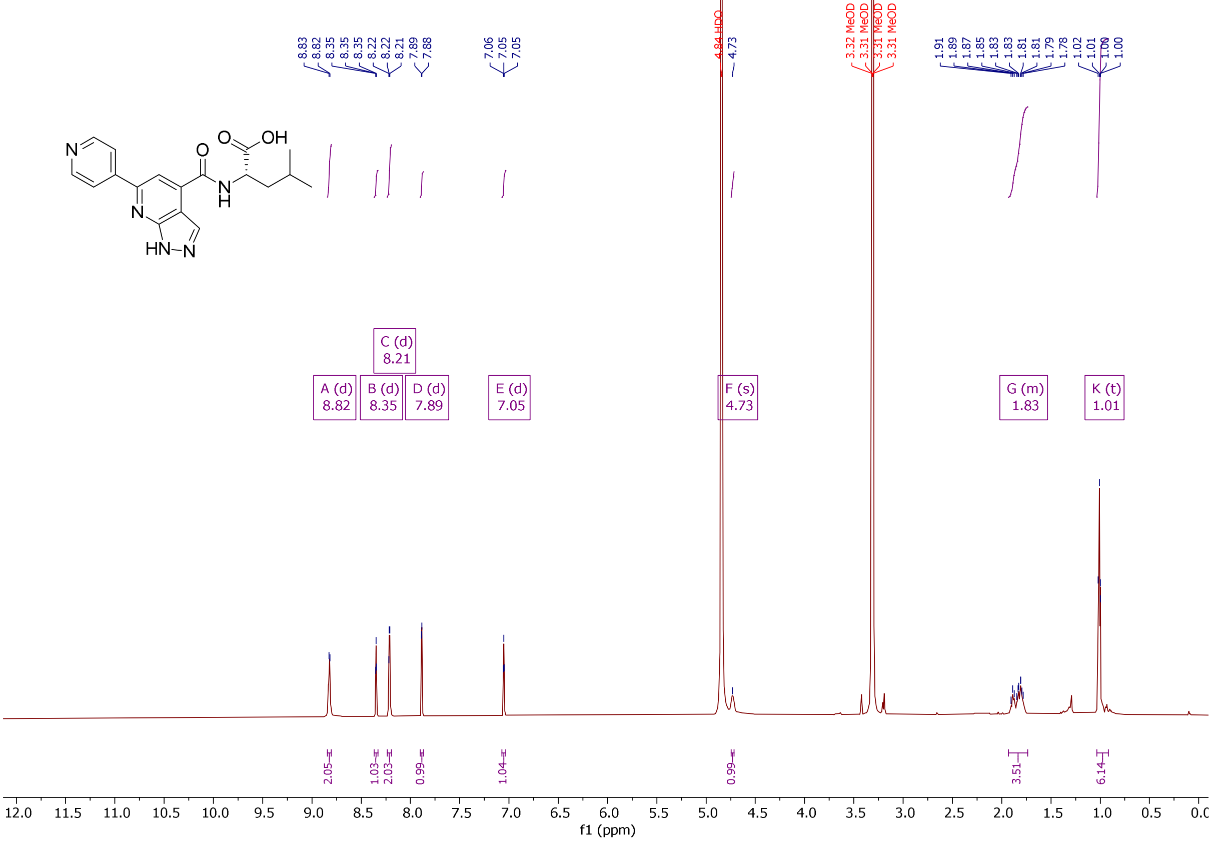

^{13}C NMR of **Compound 28b** (150 MHz, CD_3OD)

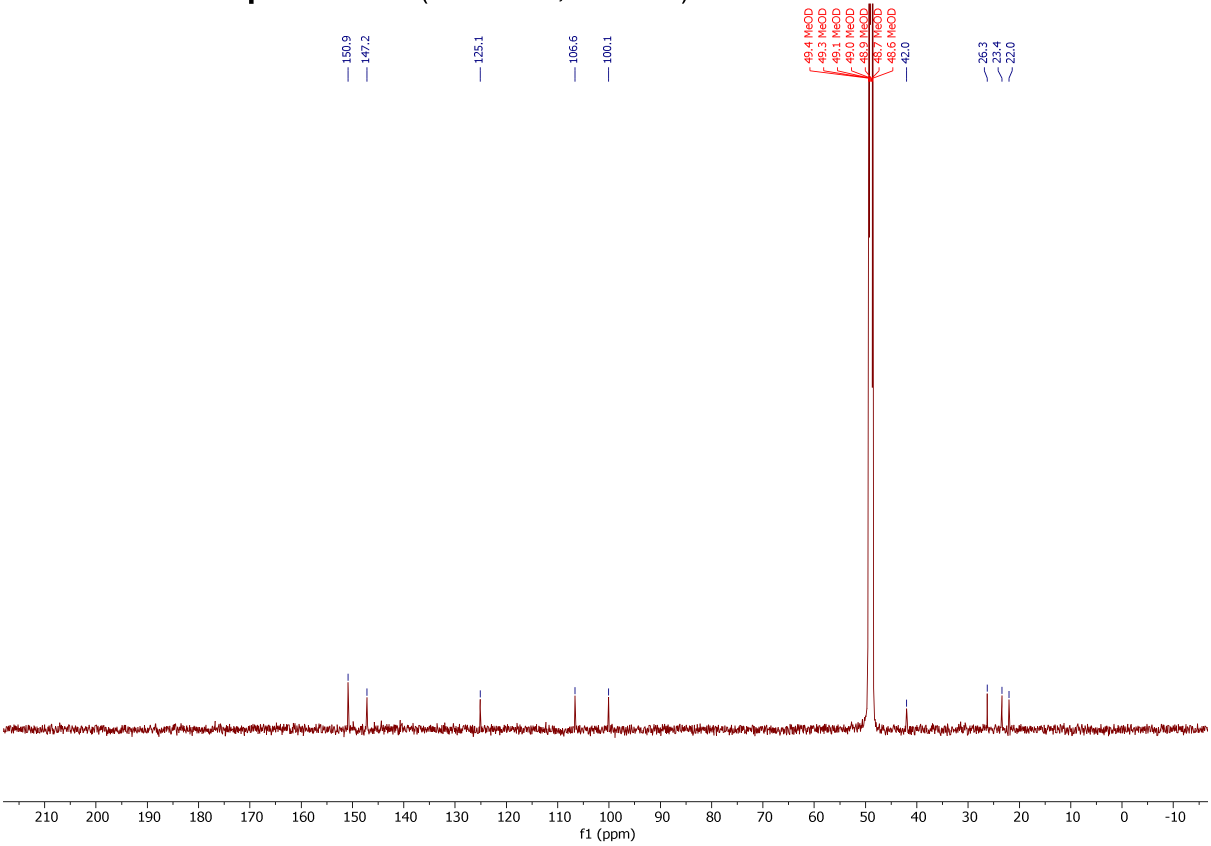

^{1}H NMR of **Compound 29b** (600 MHz, CD_3OD)

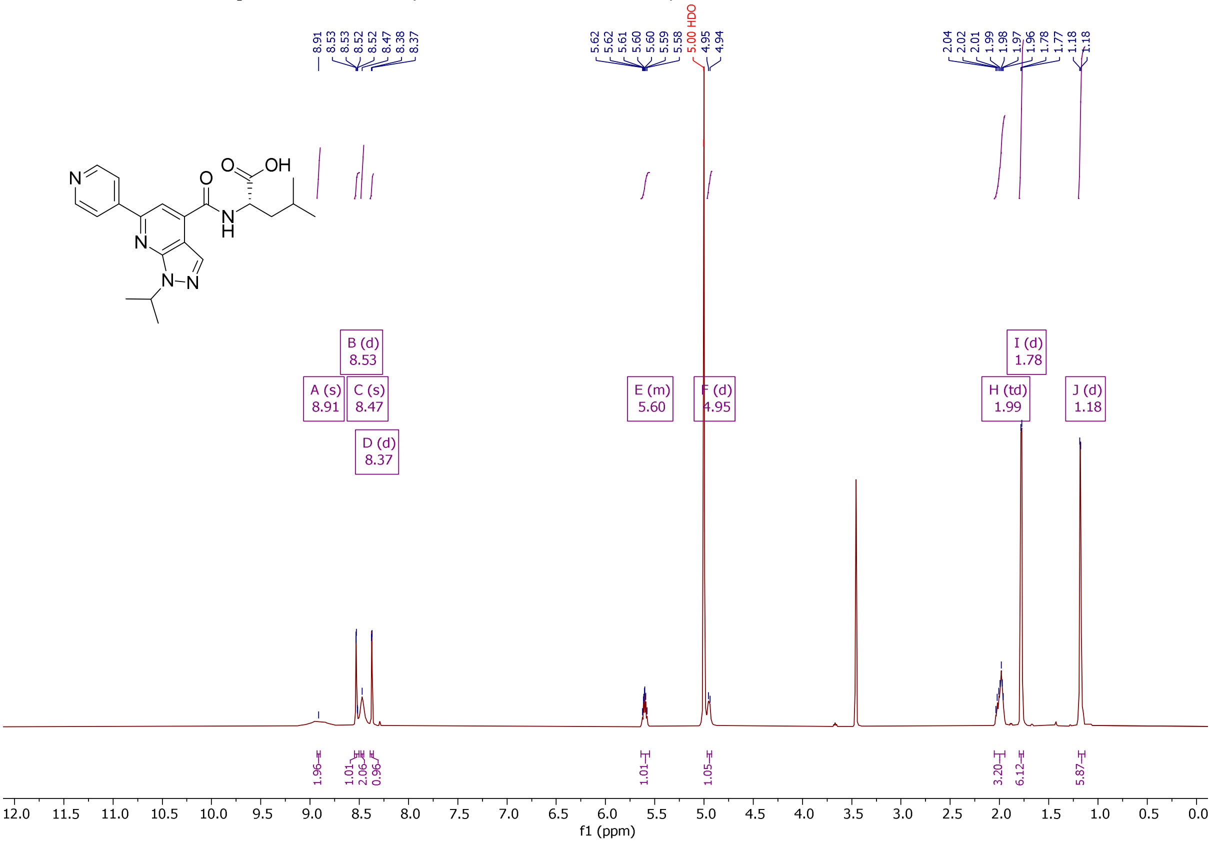

^{13}C NMR of **Compound 29b** (150 MHz, CD_3OD)

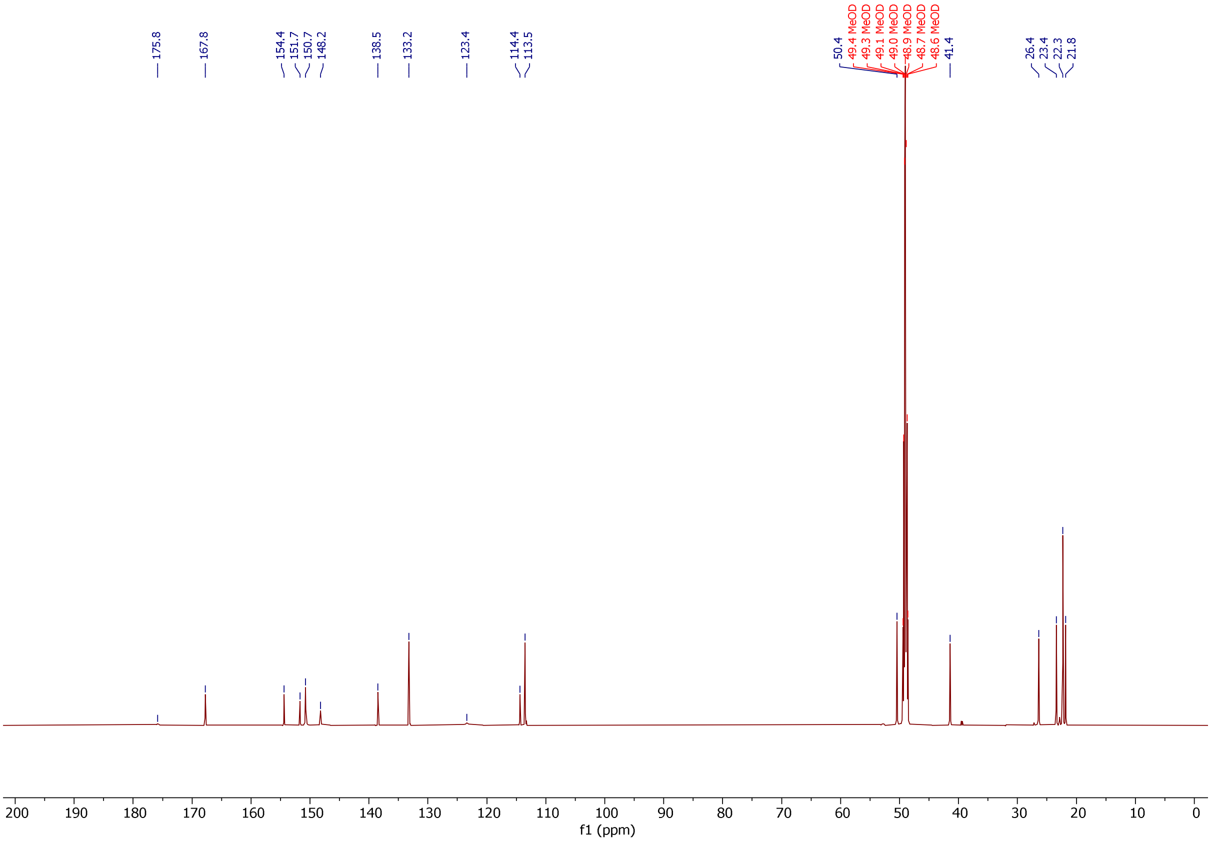

^{1}H NMR of **Compound 30b** (600 MHz, CD3OD)

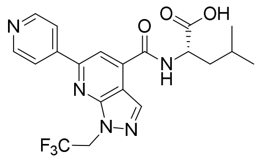

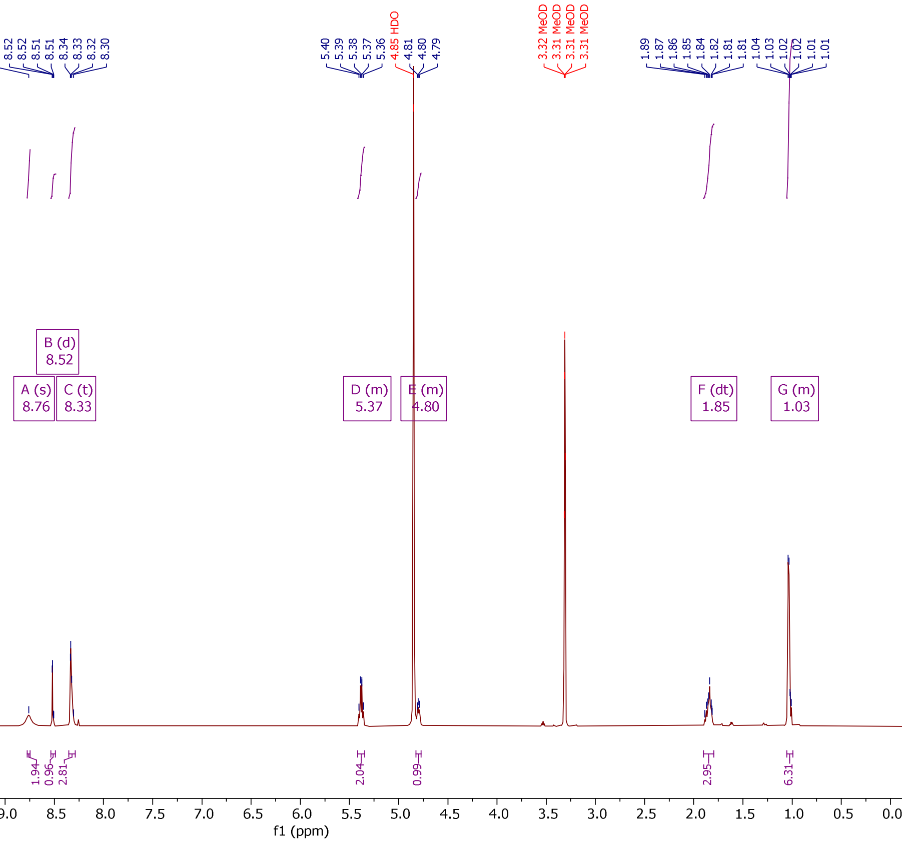

^{13}C NMR of **Compound 30b** (150 MHz, CD3OD)

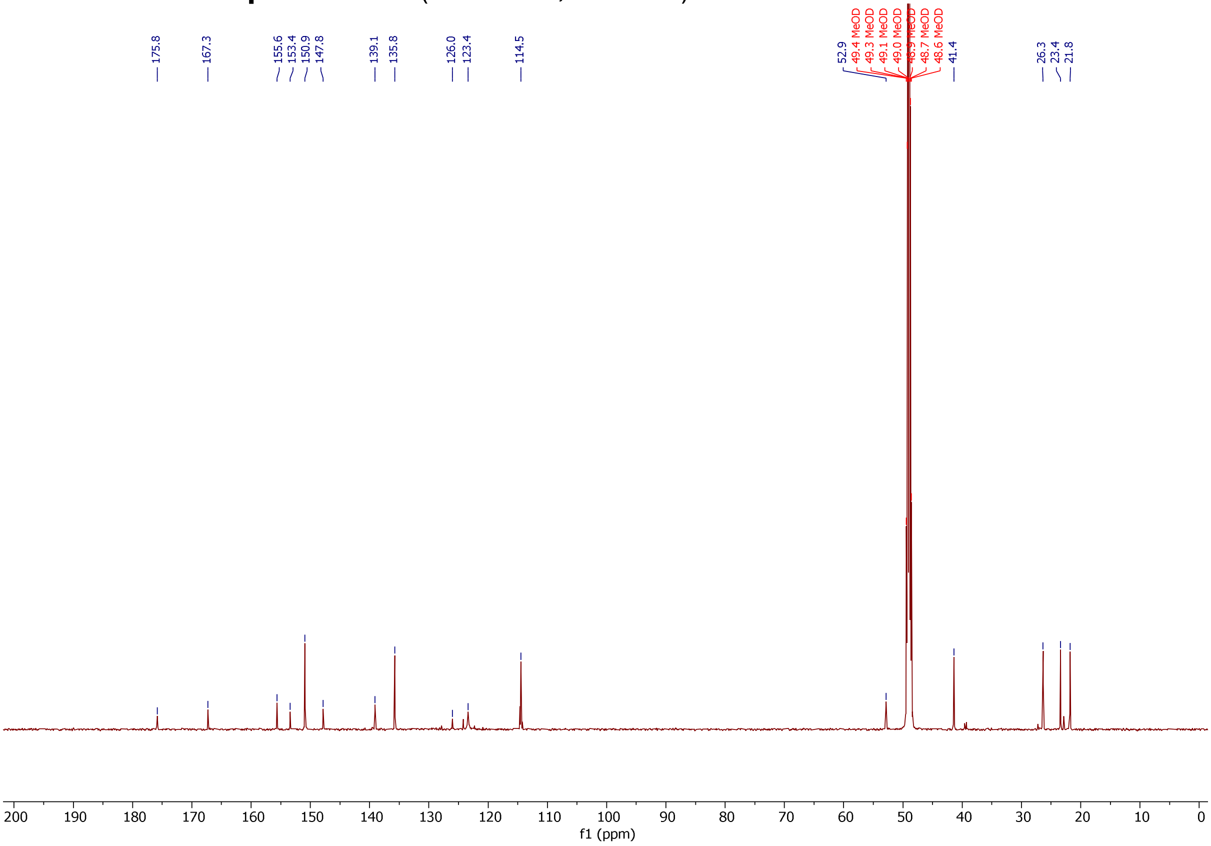

^{1}H NMR of **Compound 31b** (500 MHz, CD_3OD)

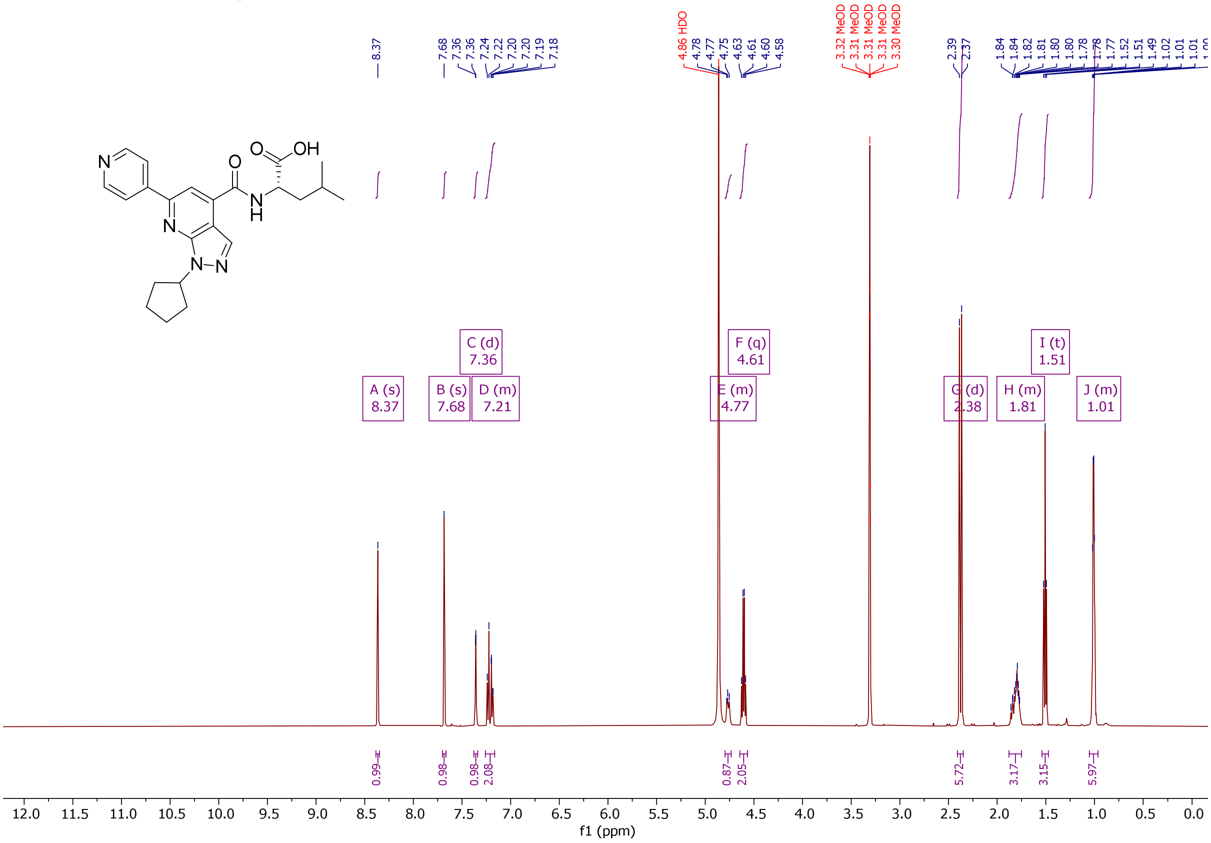

^{13}C NMR of **Compound 31b** (125 MHz, CD_3OD)

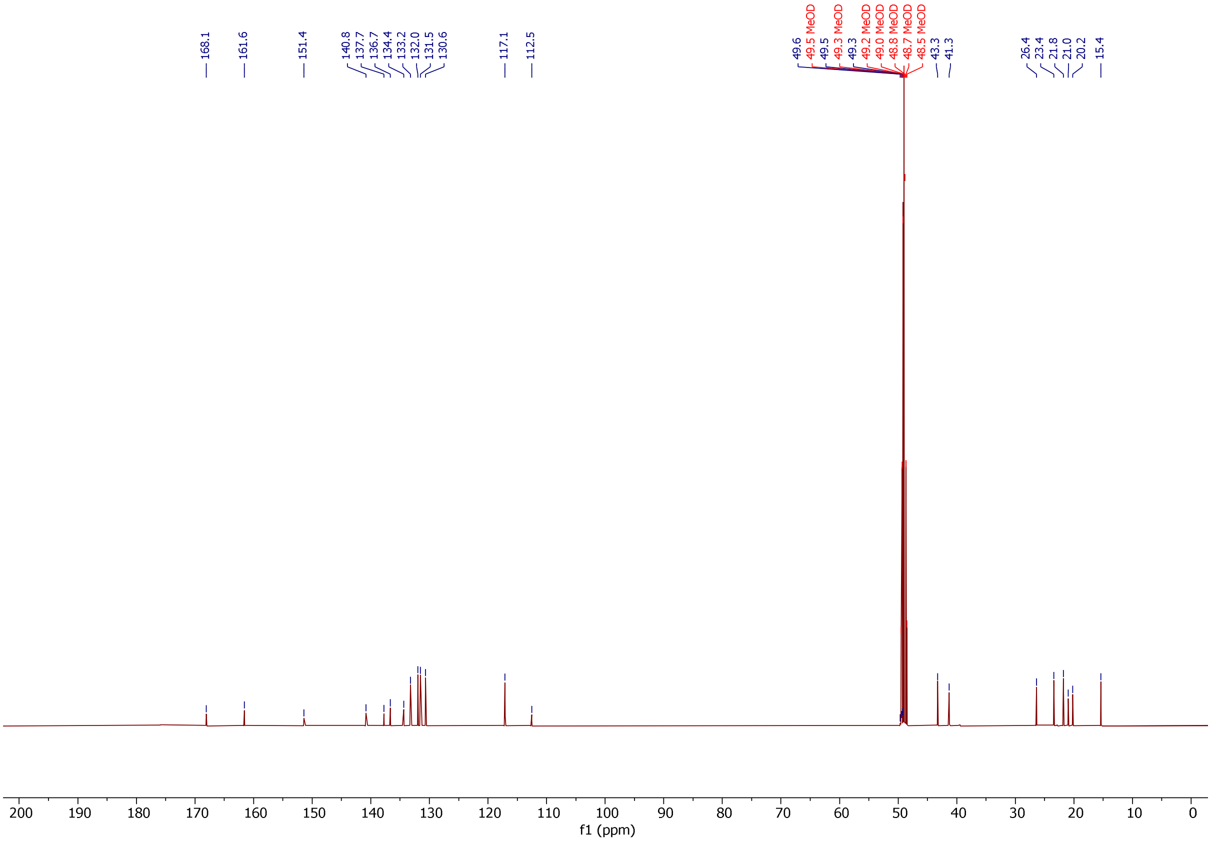

^{1}H NMR of **Compound 32b** (600 MHz, CD_3OD)

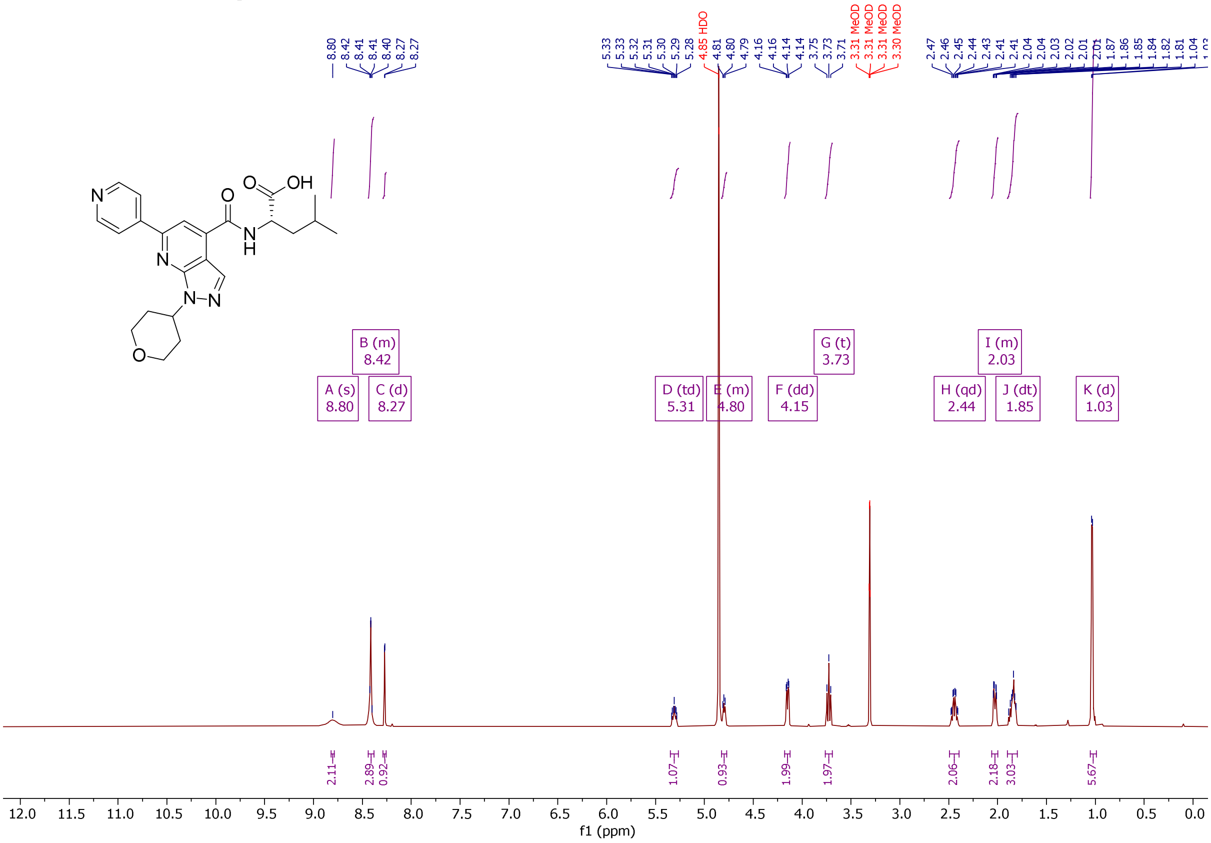

^{13}C NMR of **Compound 32b** (150 MHz, CD_3OD)

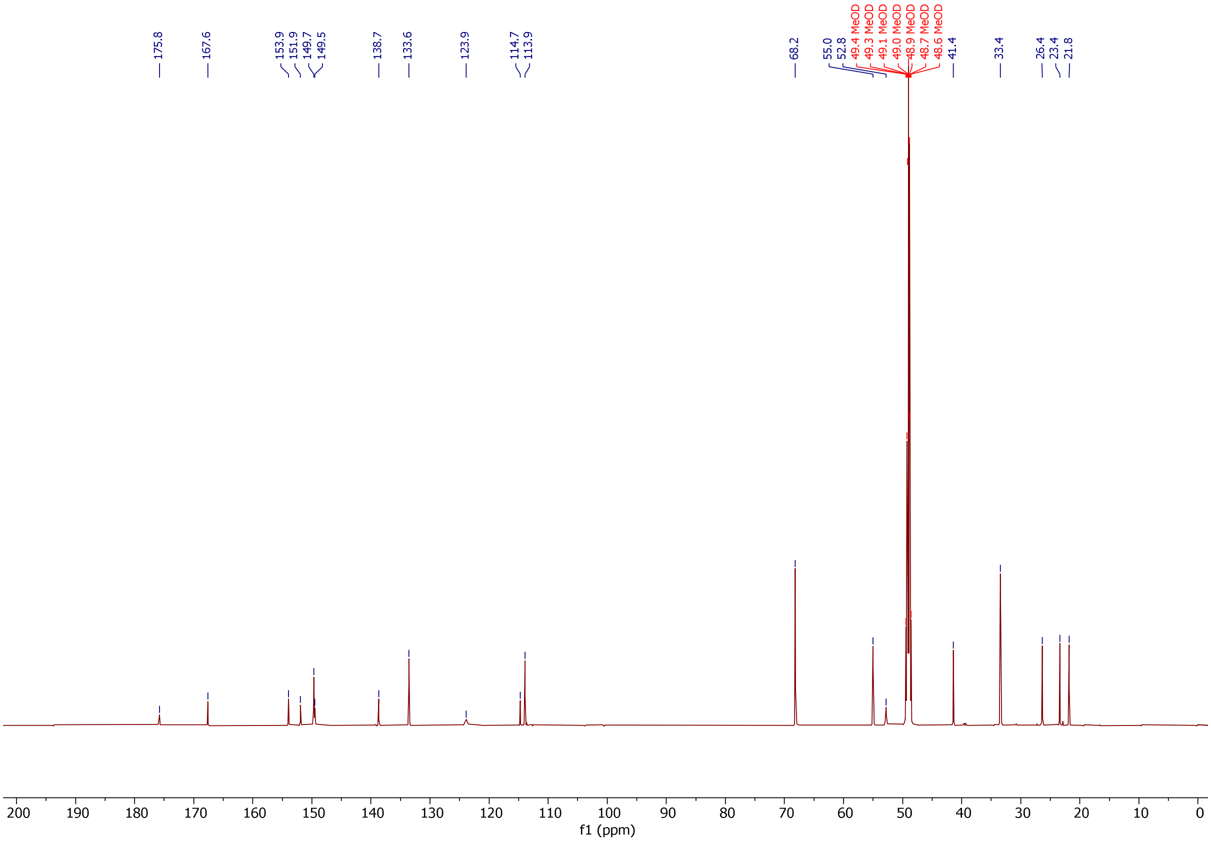

^{1}H NMR of **Compound 33b** (500 MHz, CD_3OD)

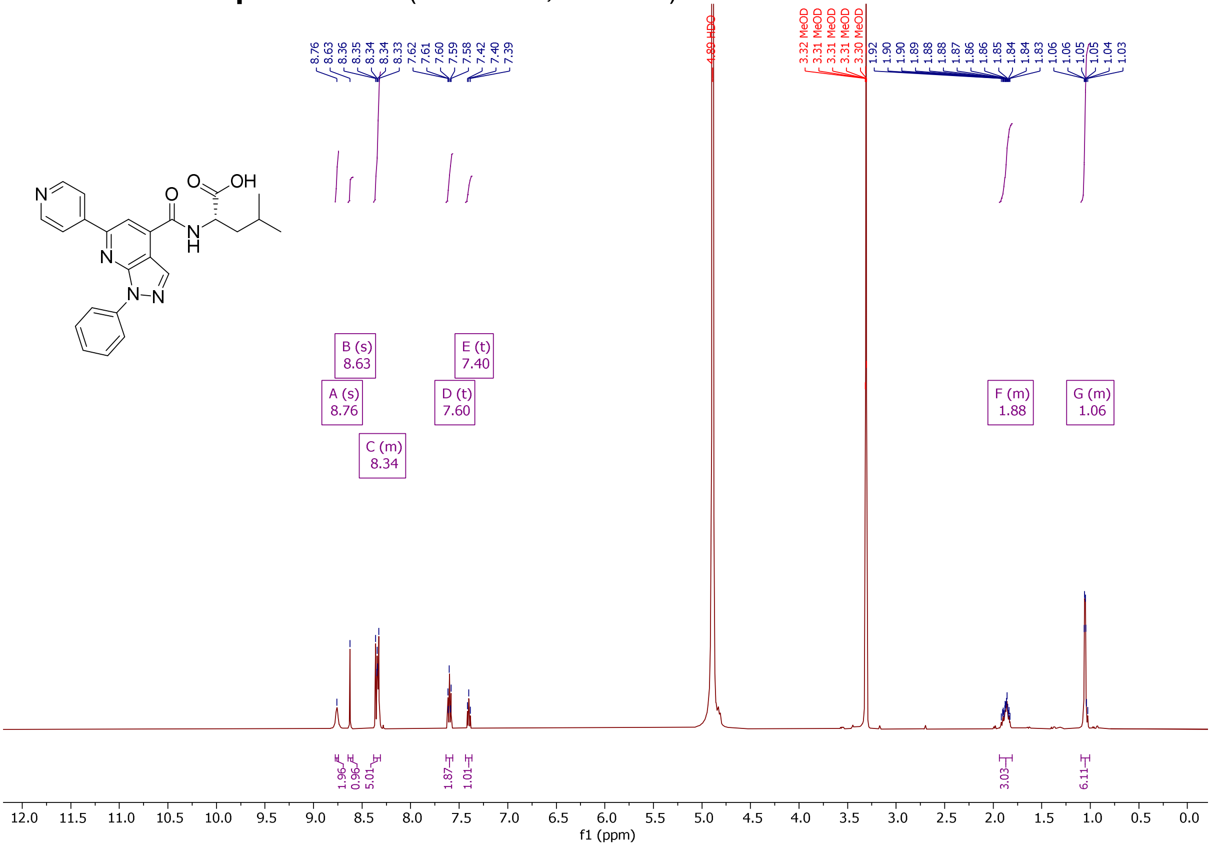

^{13}C NMR of **Compound 33b** (125 MHz, CD_3OD

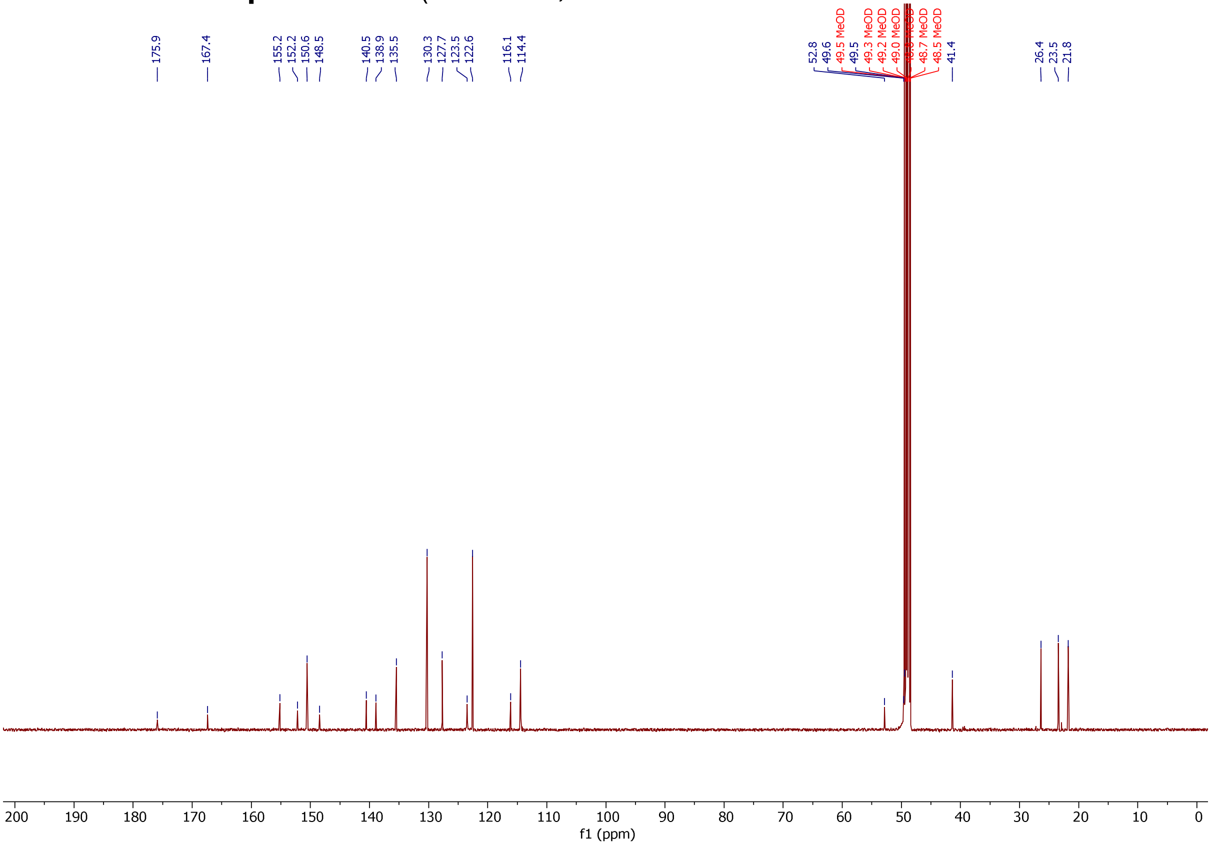

^{1}H NMR of **Compound 34b** (600 MHz, CD_3OD)

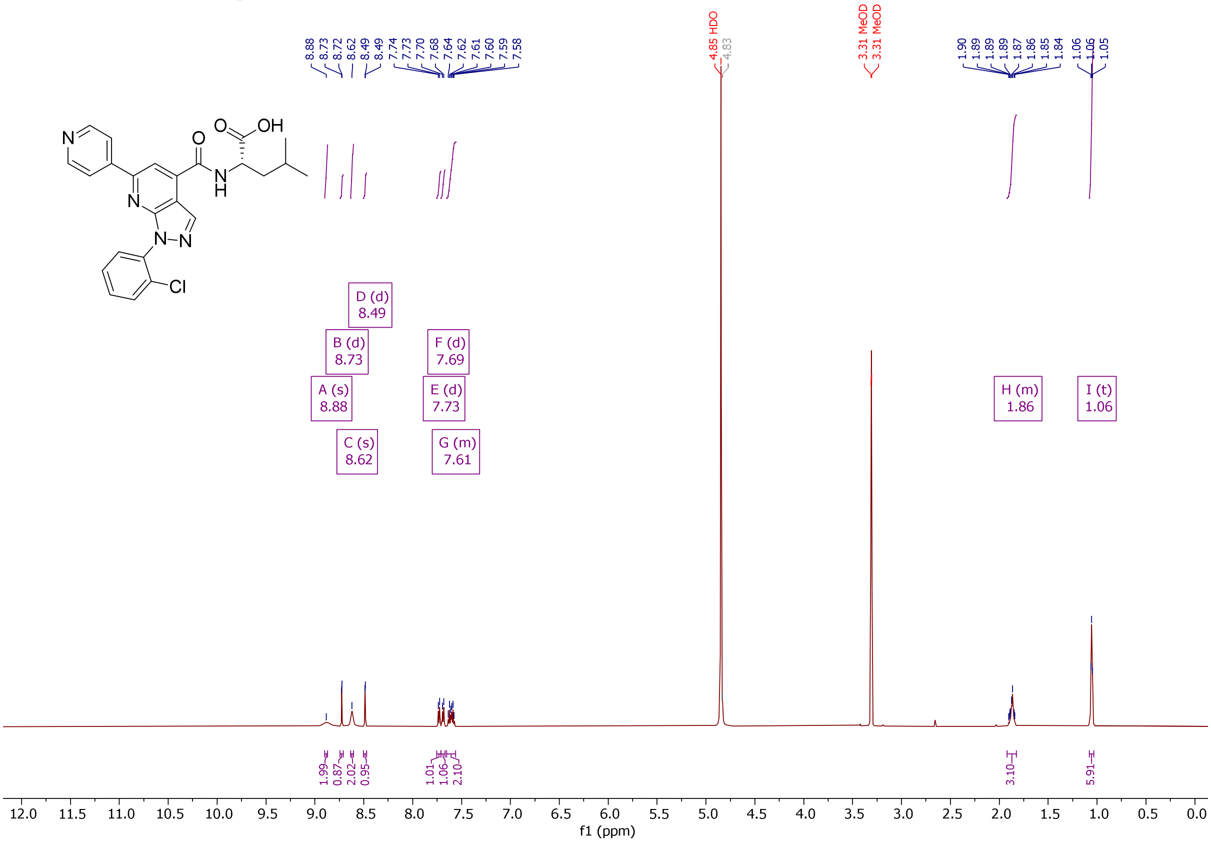

^{13}C NMR of **Compound 34b** (150 MHz, CD_3OD)

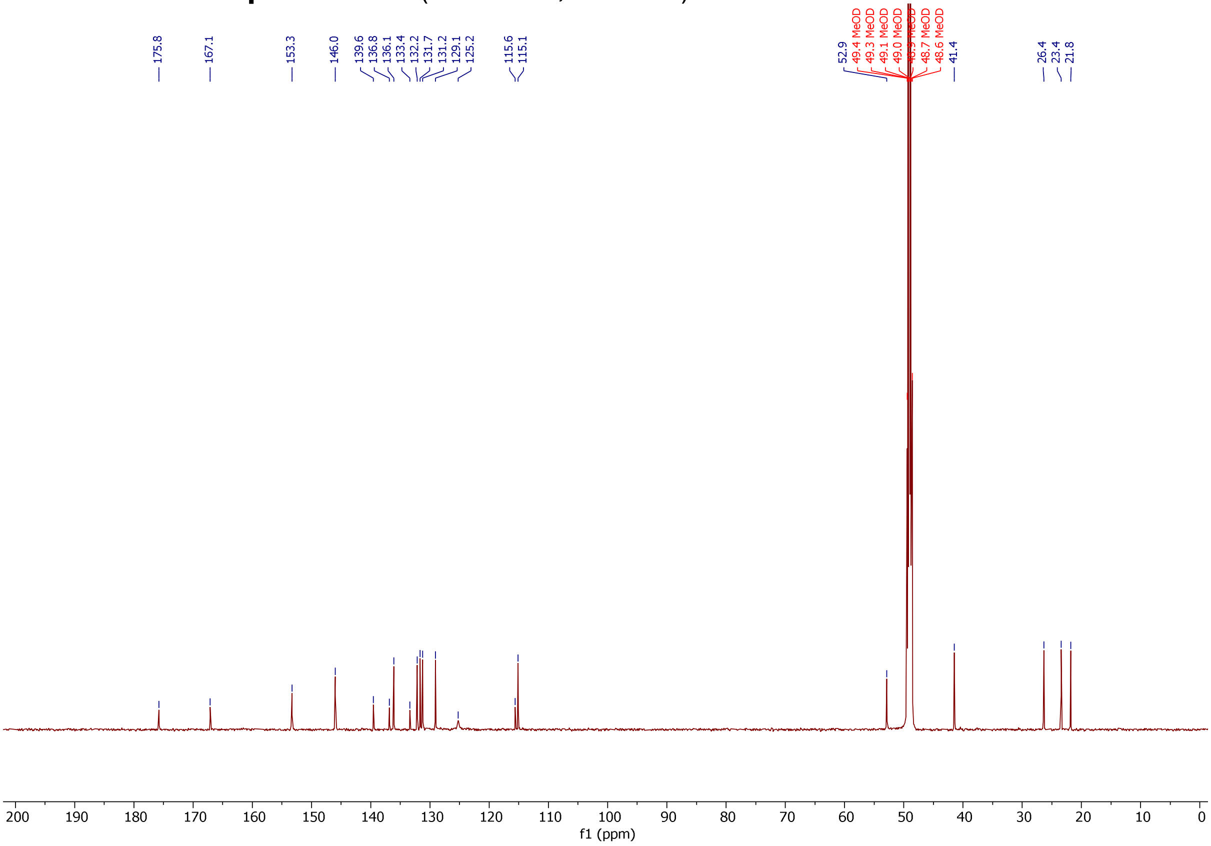

^{1}H NMR of **Compound 35b** (500 MHz, CD_3OD)

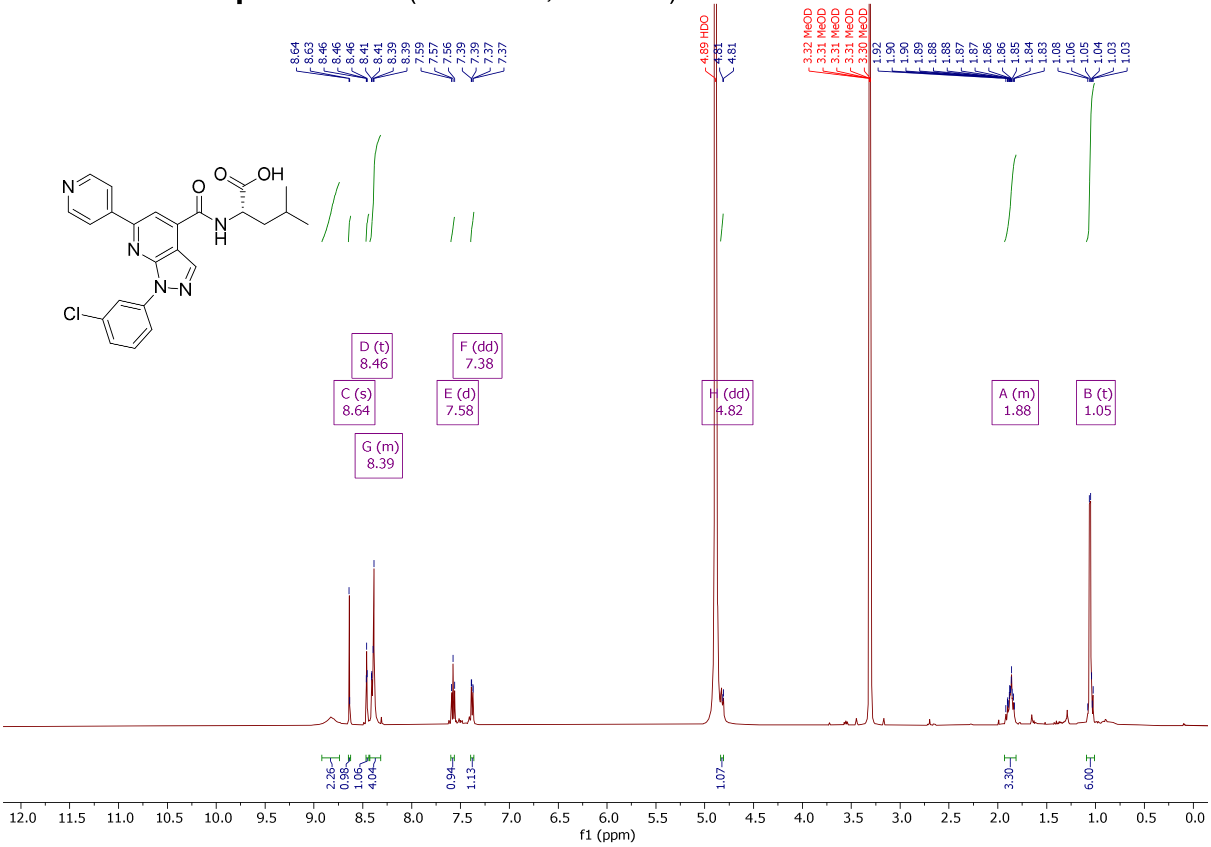

^{13}C NMR of **Compound 35b** (125 MHz, CD_3OD)

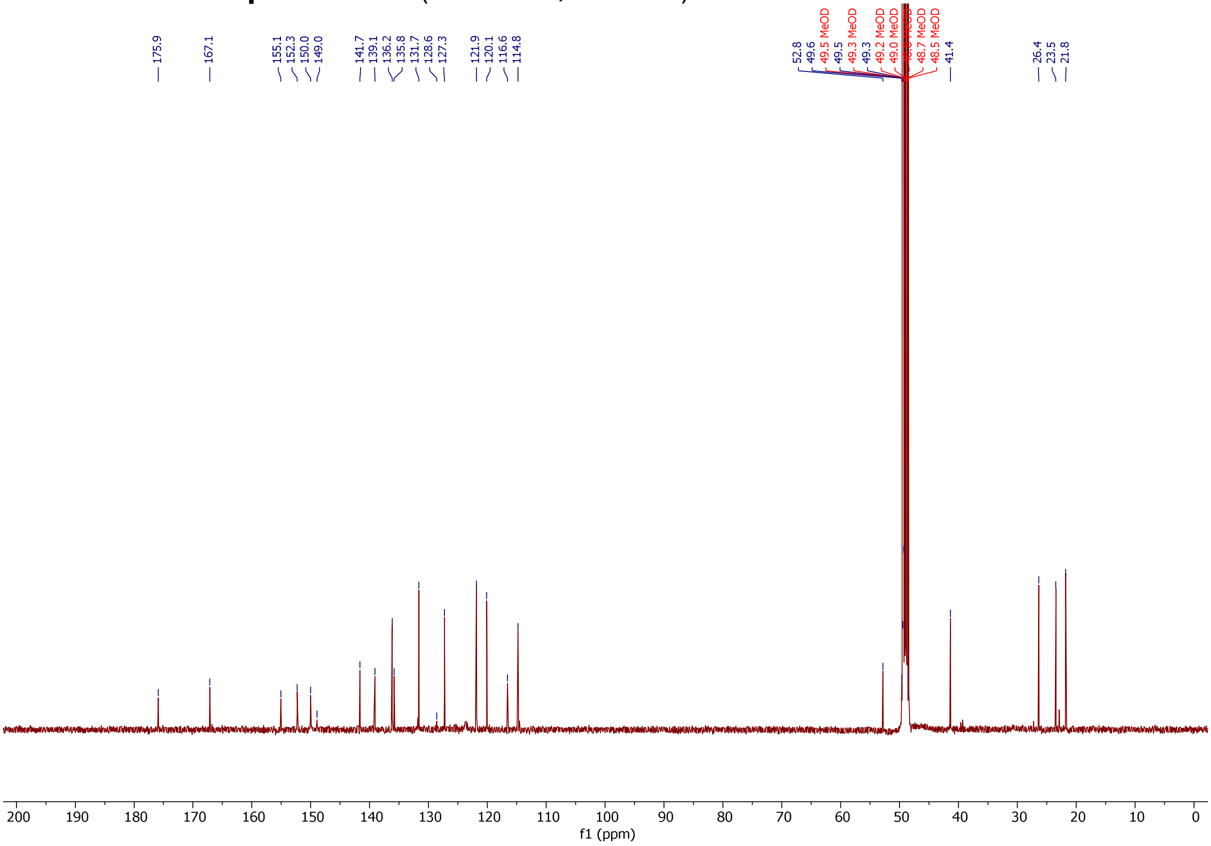

^{1}H NMR of **Compound 36b** (600 MHz, CD_3OD)

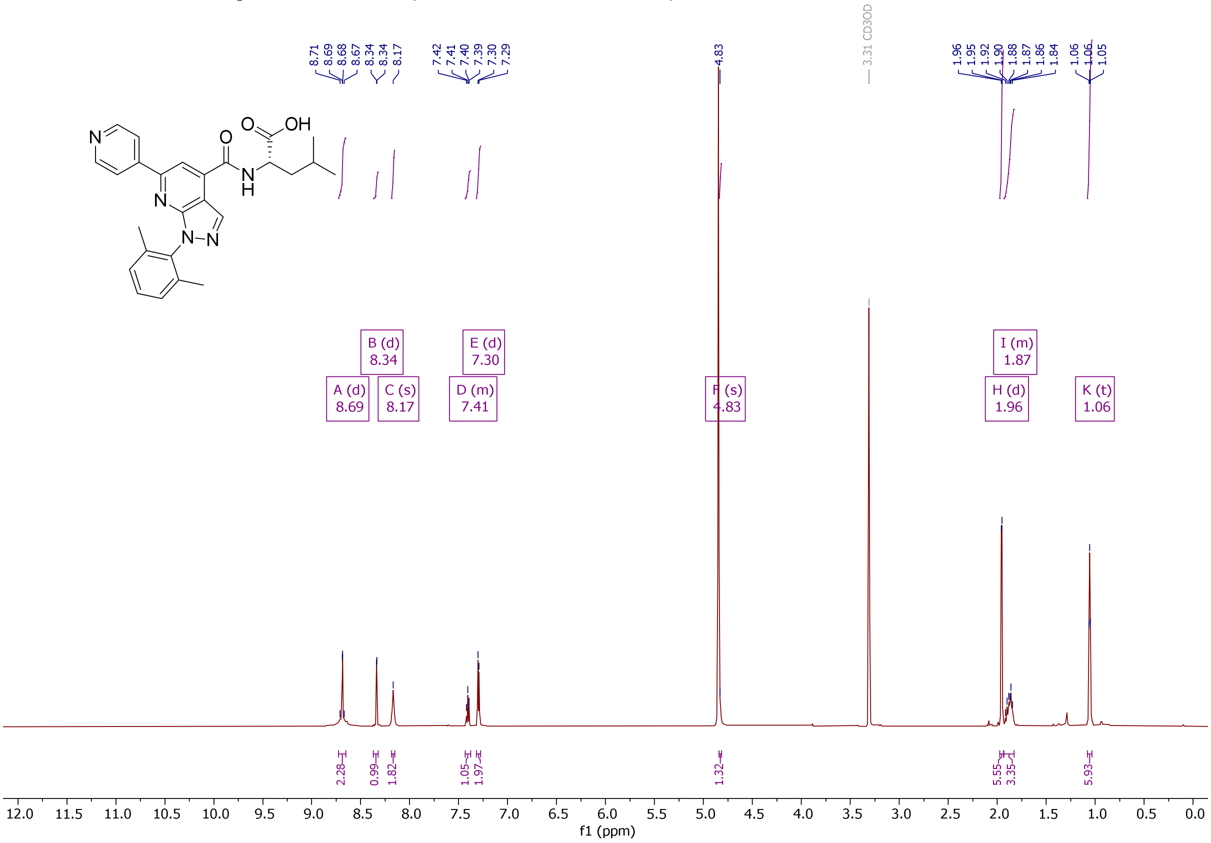

^{13}C NMR of **Compound 36b** (150 MHz, CD_3OD)

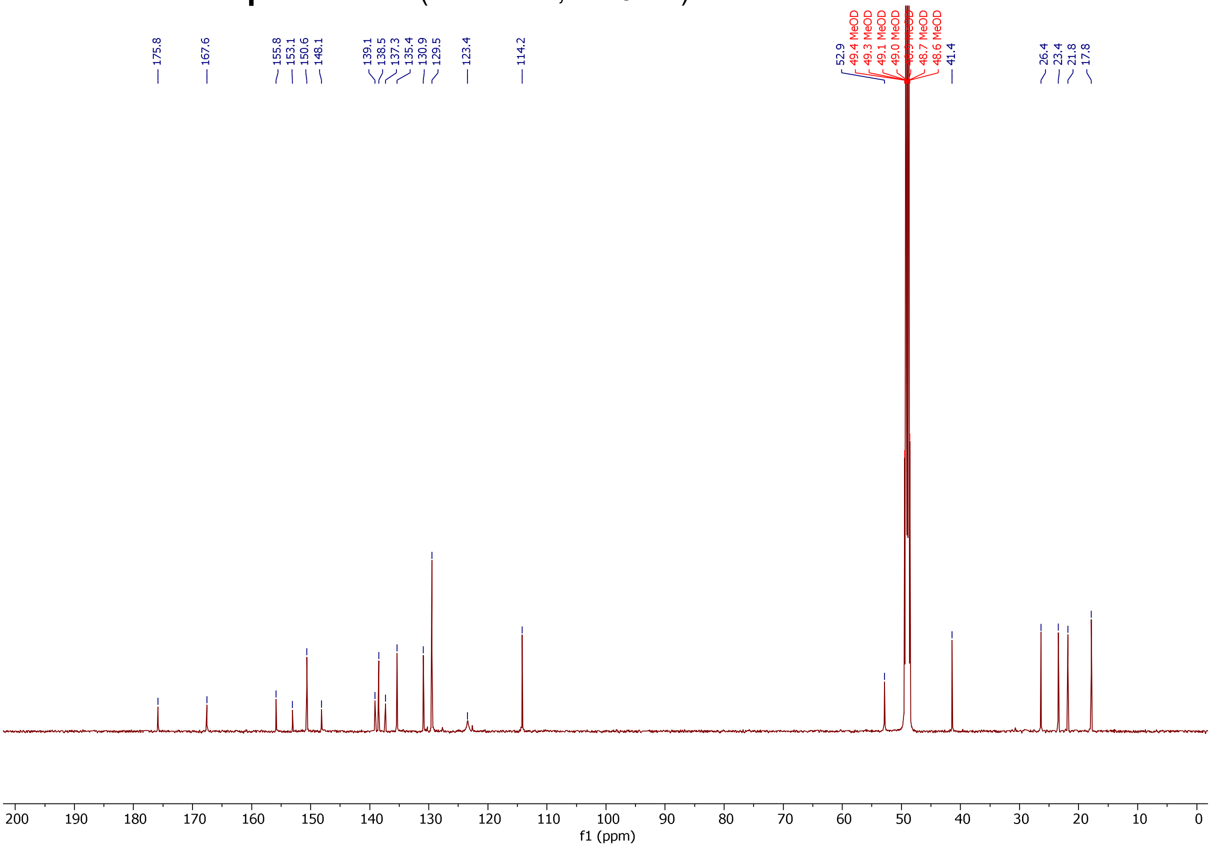

^{1}H NMR of **Compound 37b** (500 MHz, CD3OD)

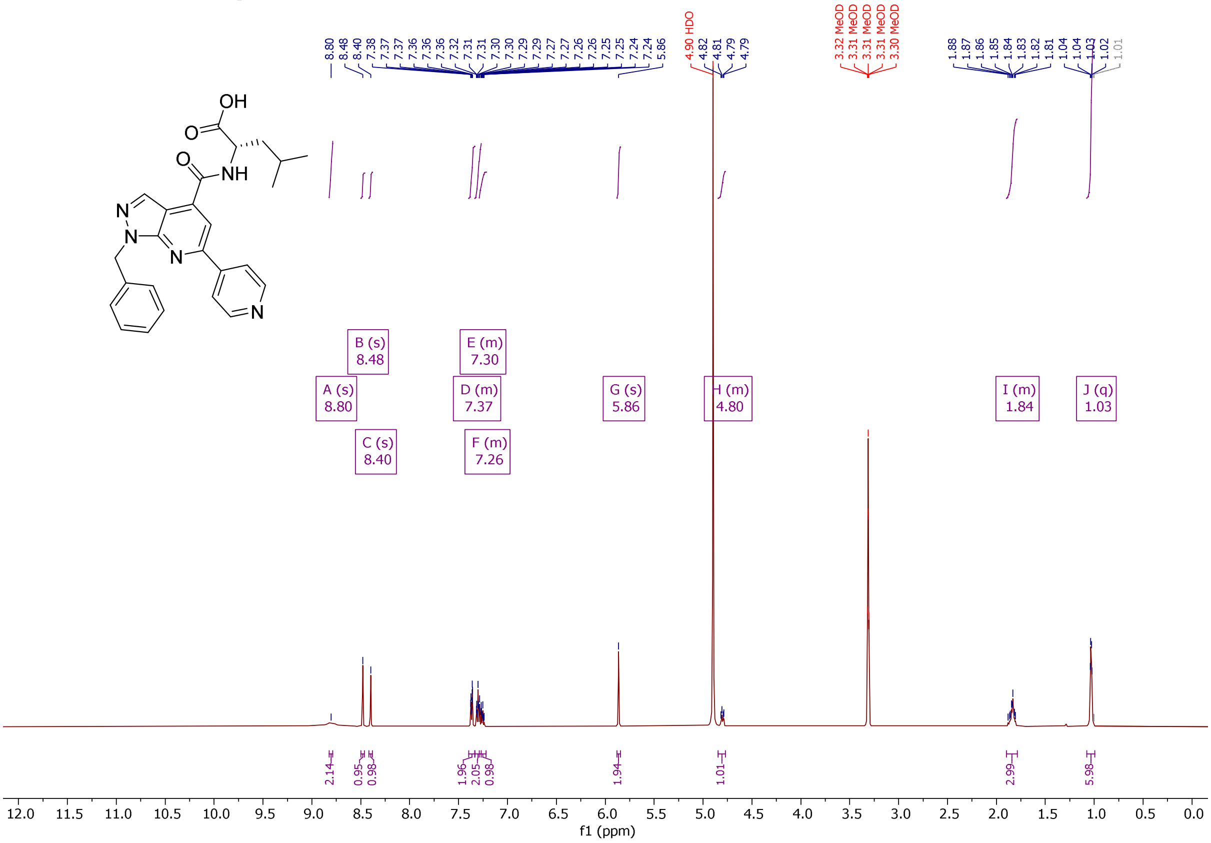

^{13}C NMR of **Compound 37b** (125 MHz, CD3OD)

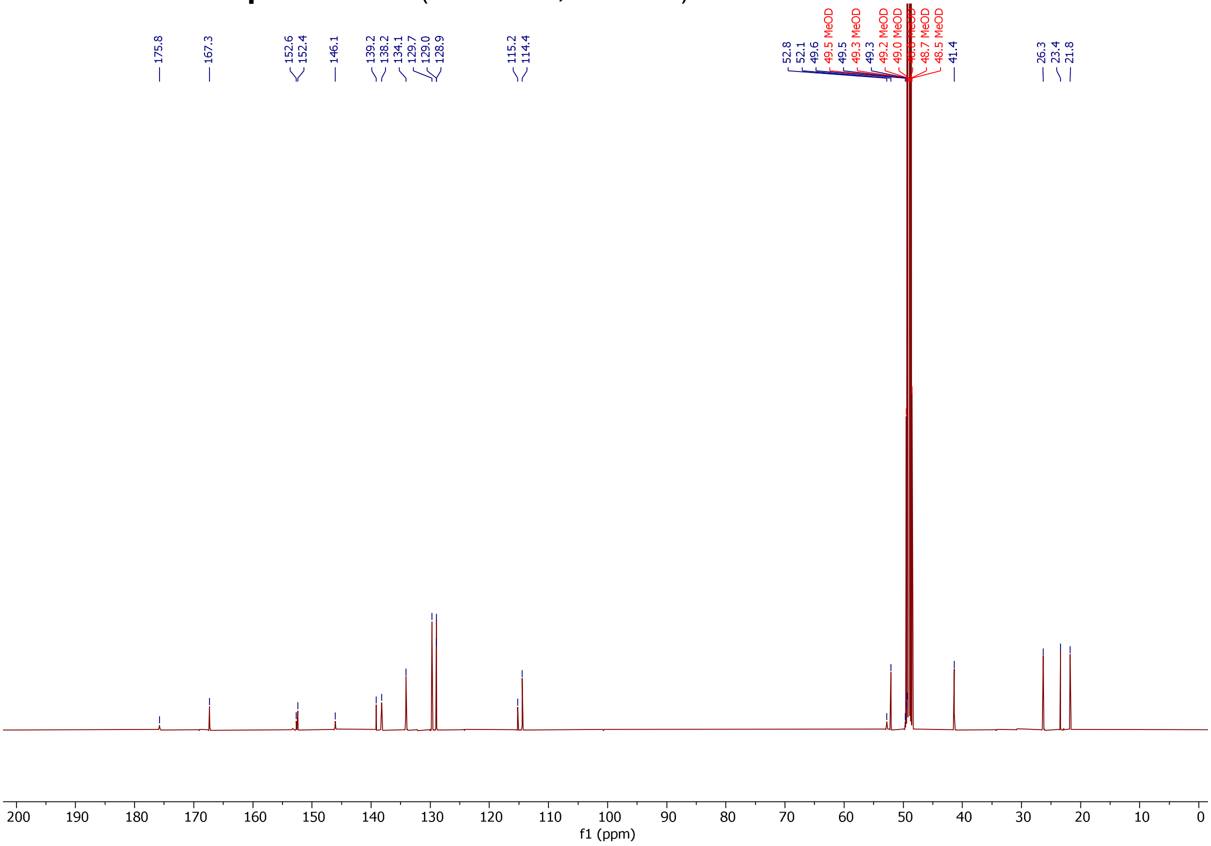

^{1}H NMR of **Compound 38b** (500 MHz, CD_3OD)

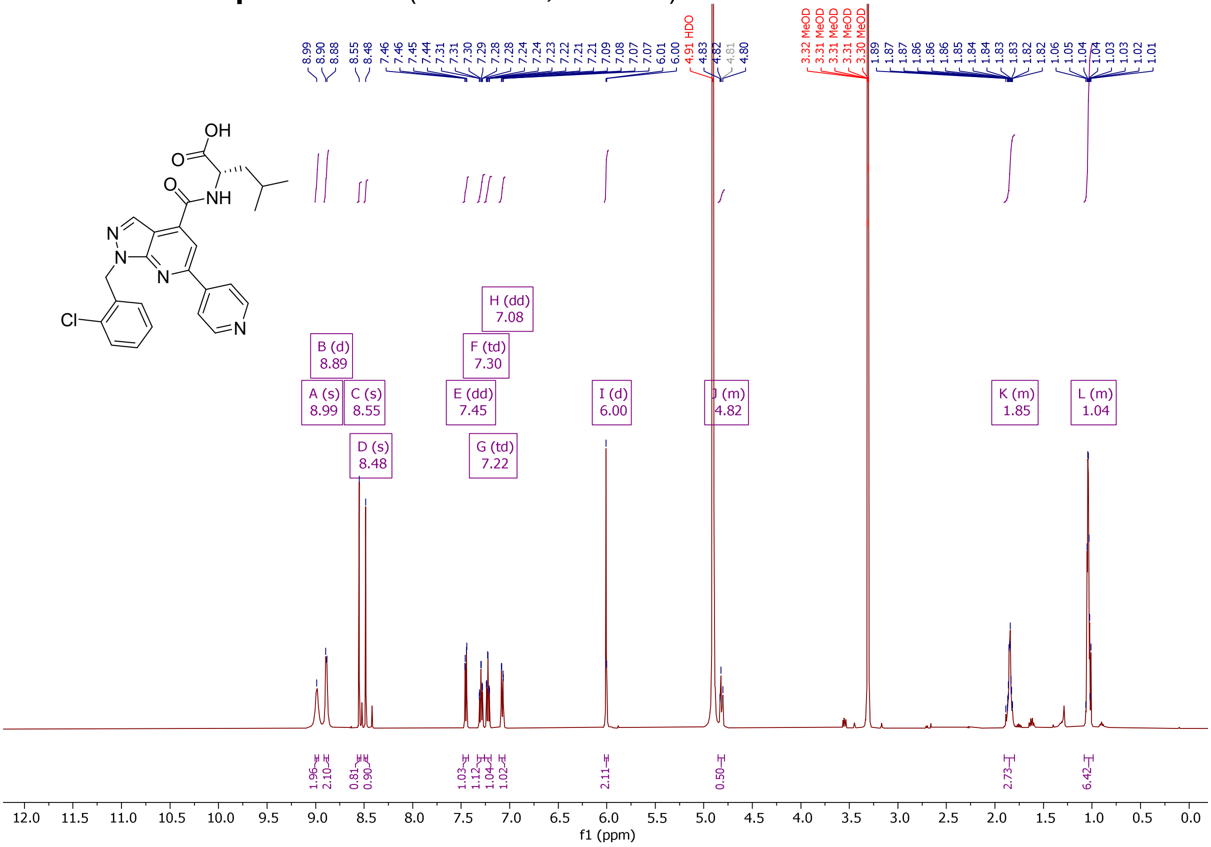

^{13}C NMR of **Compound 38b** (125 MHz, CD_3OD)

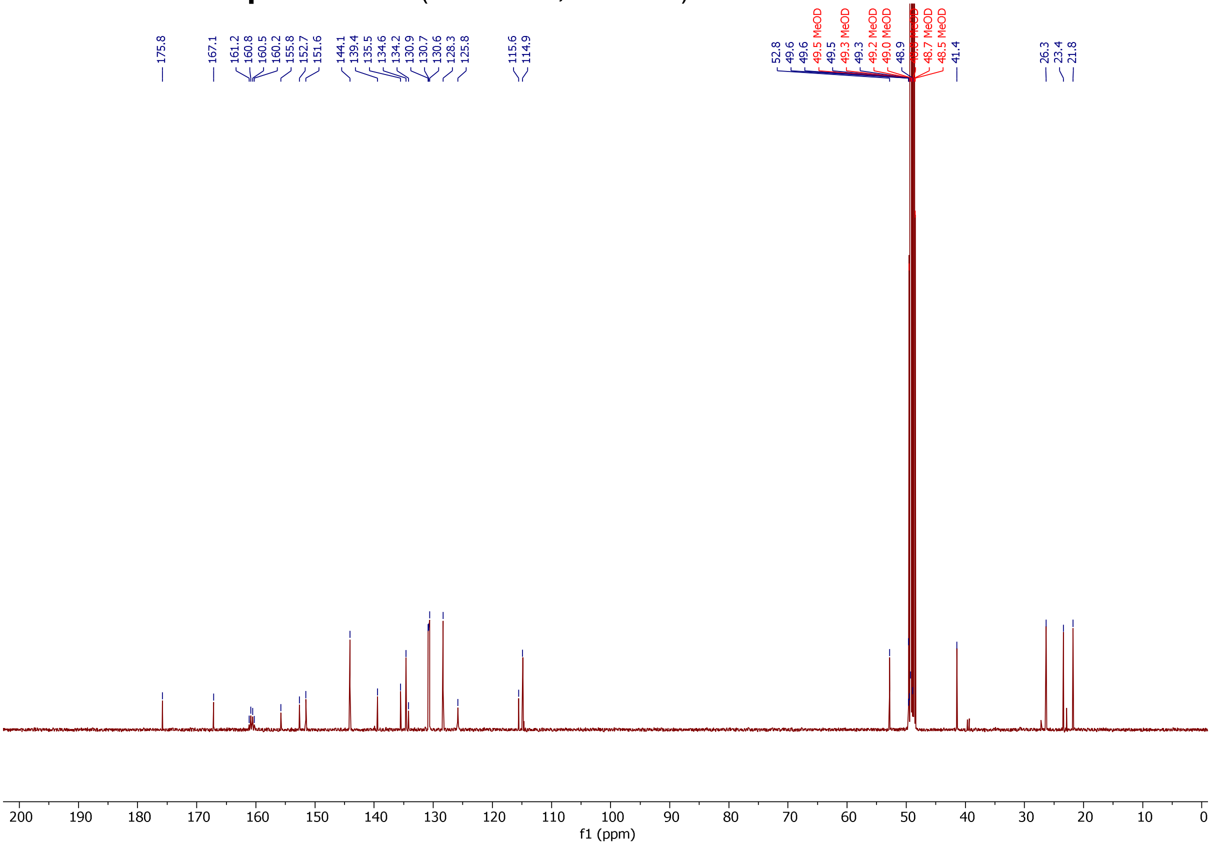

^{1}H NMR of **Compound 39b** (500 MHz, CD_3OD)

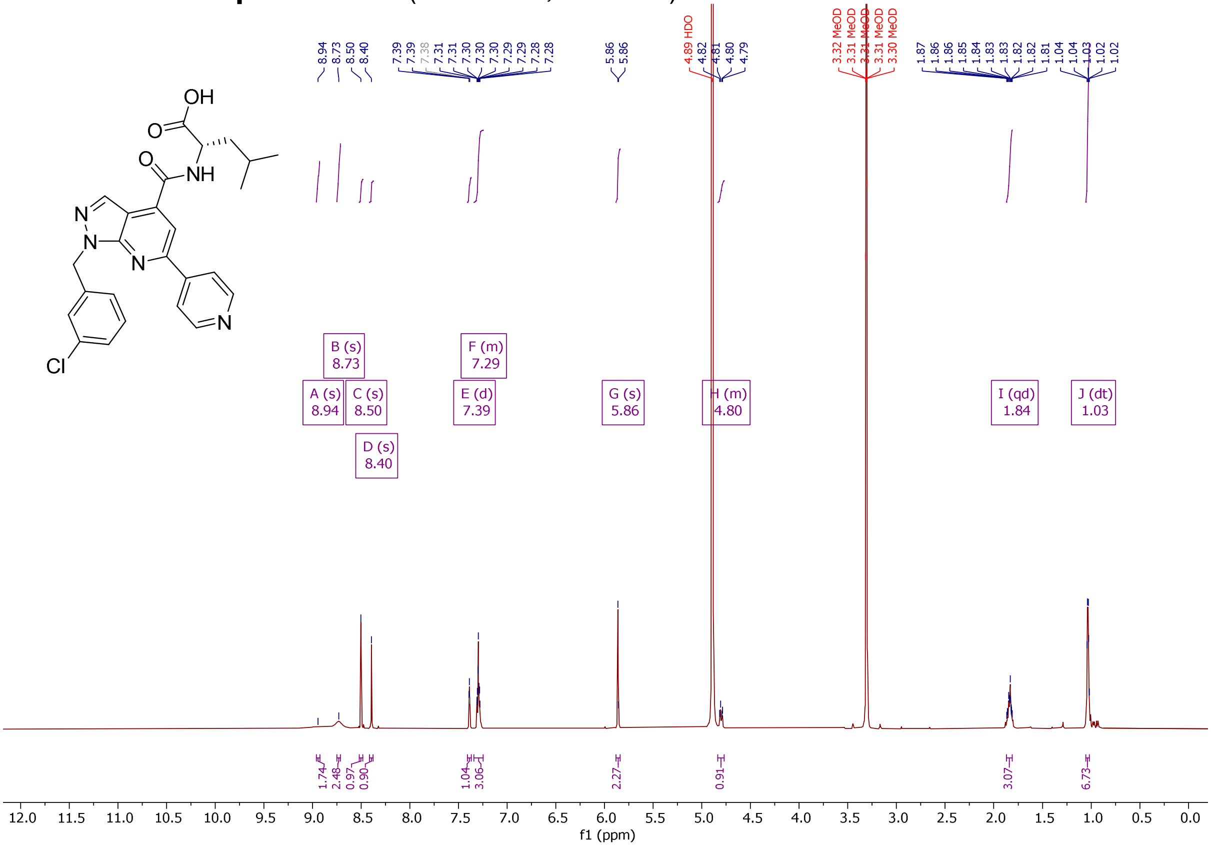

^{13}C NMR of **Compound 39b** (125 MHz, CD_3OD)

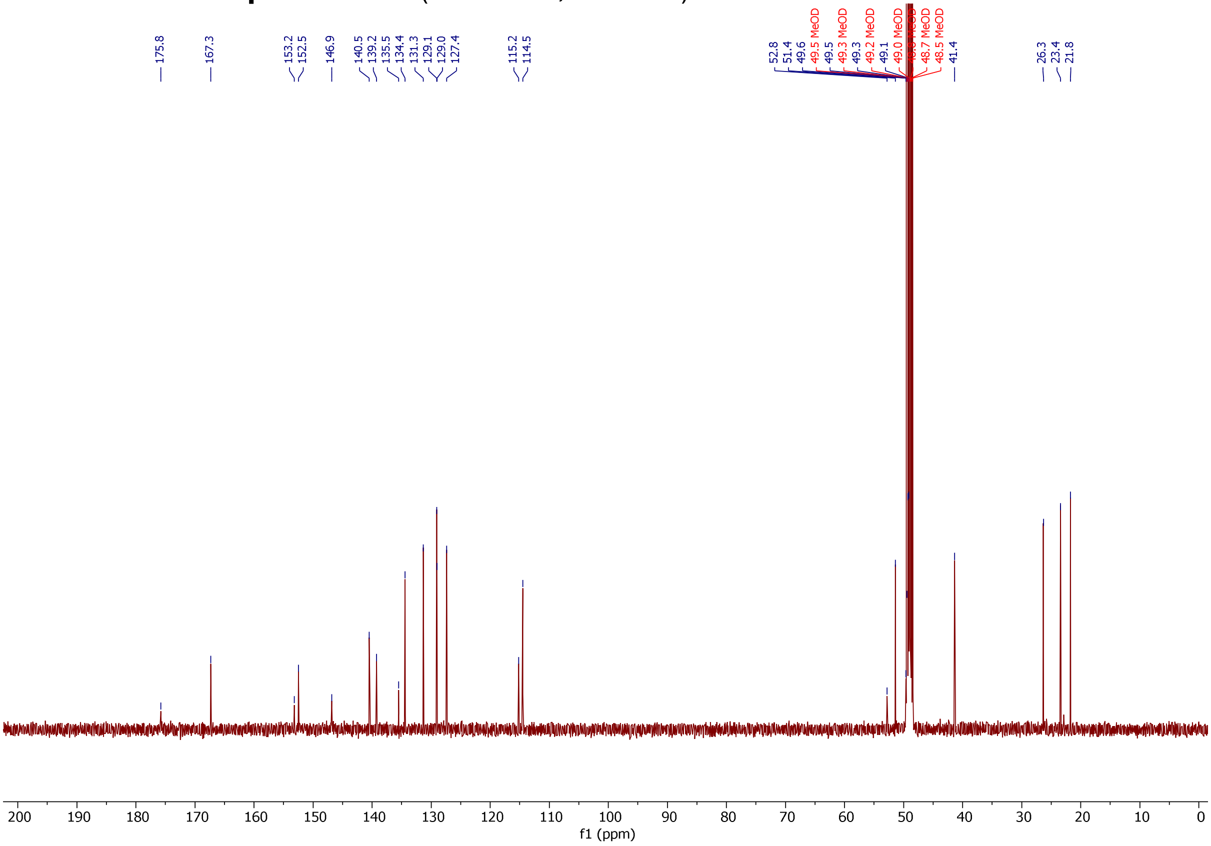

^{1}H NMR of **Compound 40b** (500 MHz, CD_3OD)

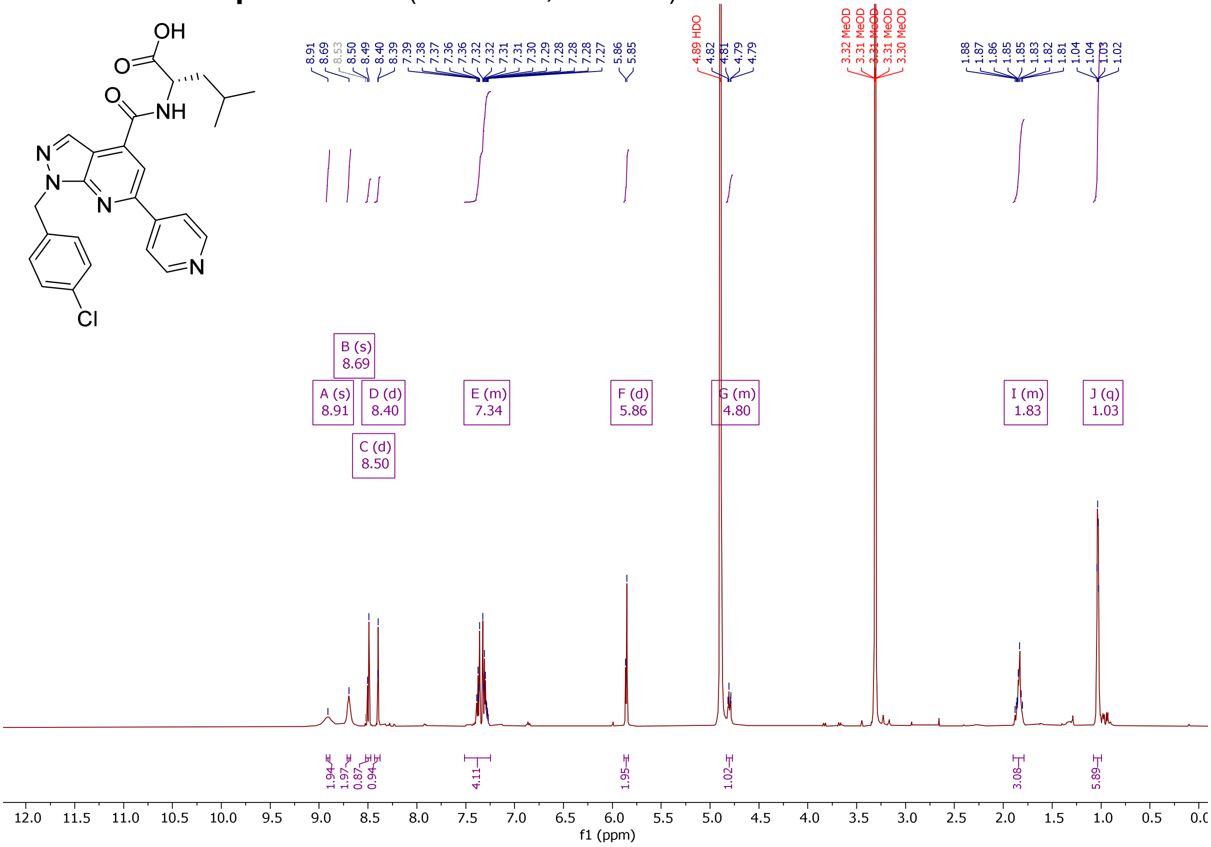

^{13}C NMR of **Compound 40b** (125 MHz, CD_3OD)

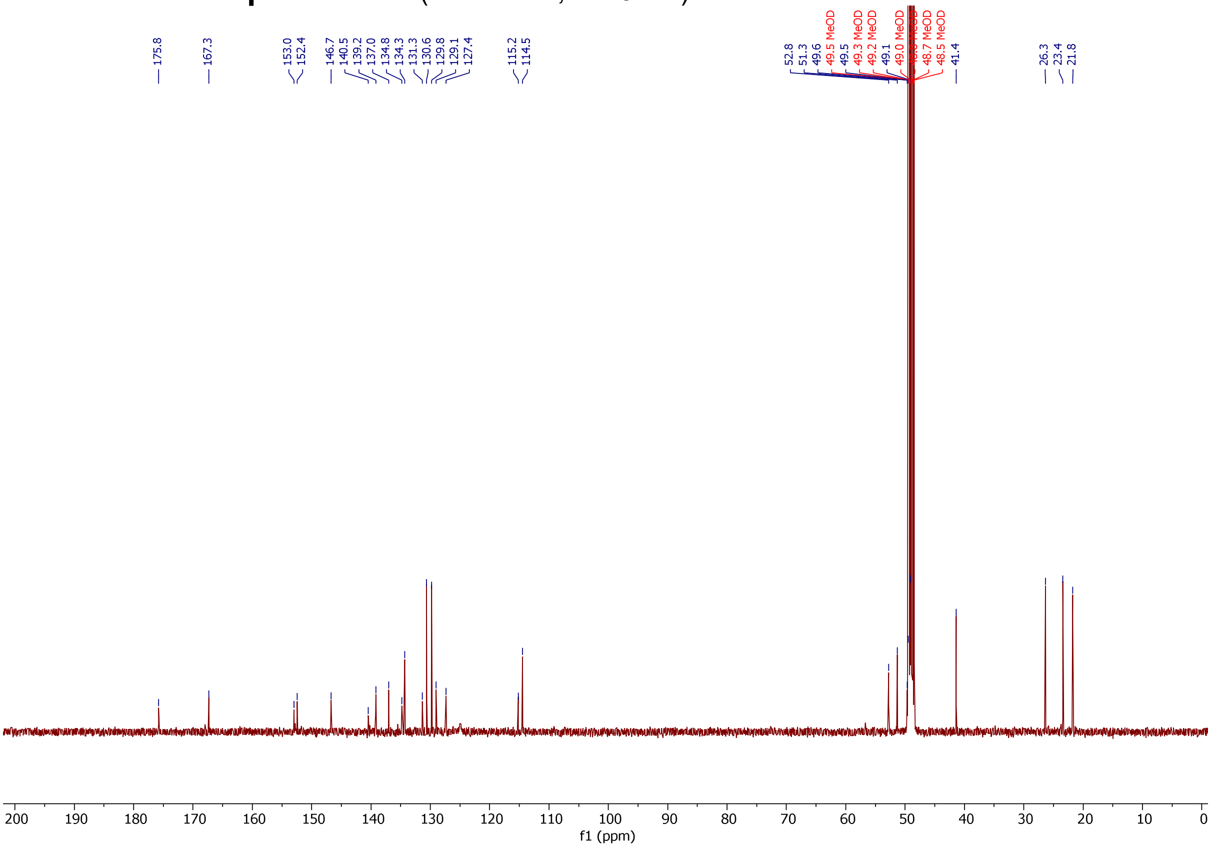

^{1}H NMR of **Compound 41b** (500 MHz, CD_3OD)

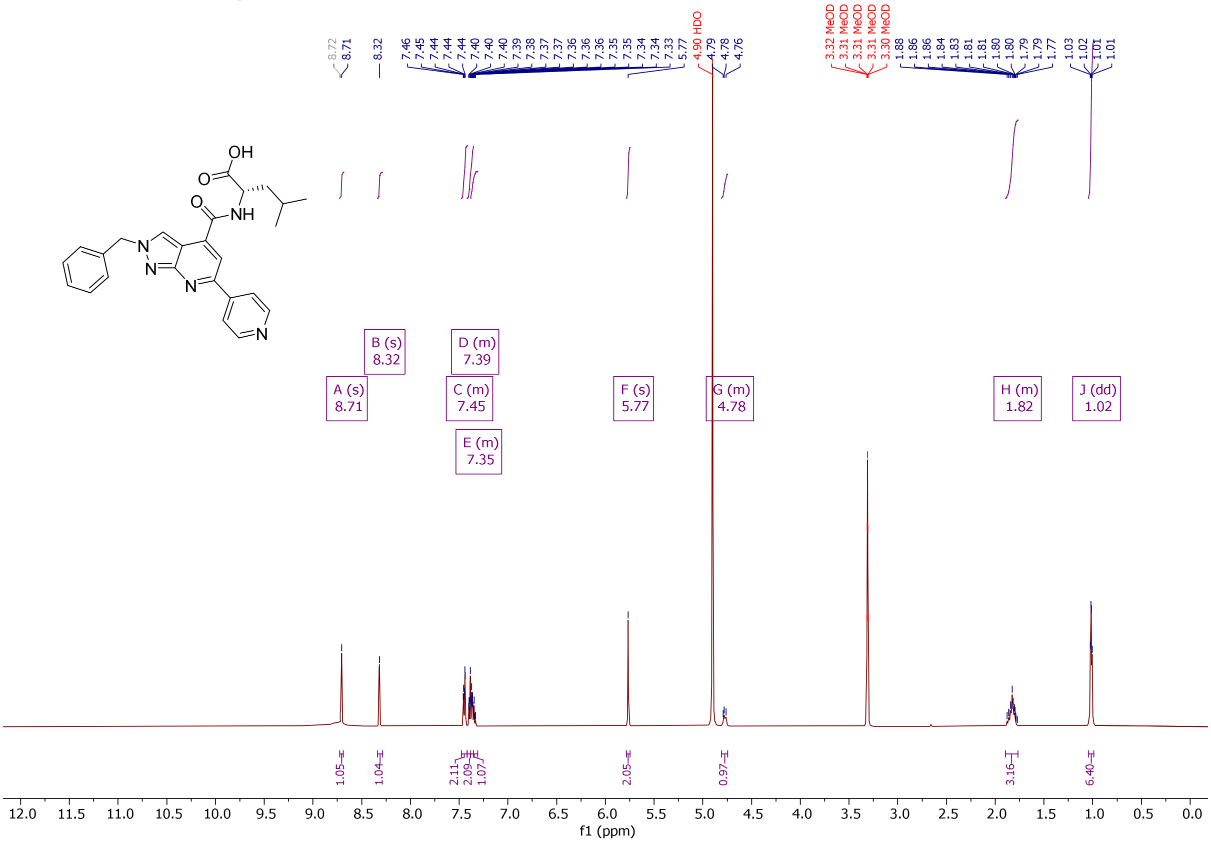

^{13}C NMR of **Compound 41b** (125 MHz, CD_3OD)

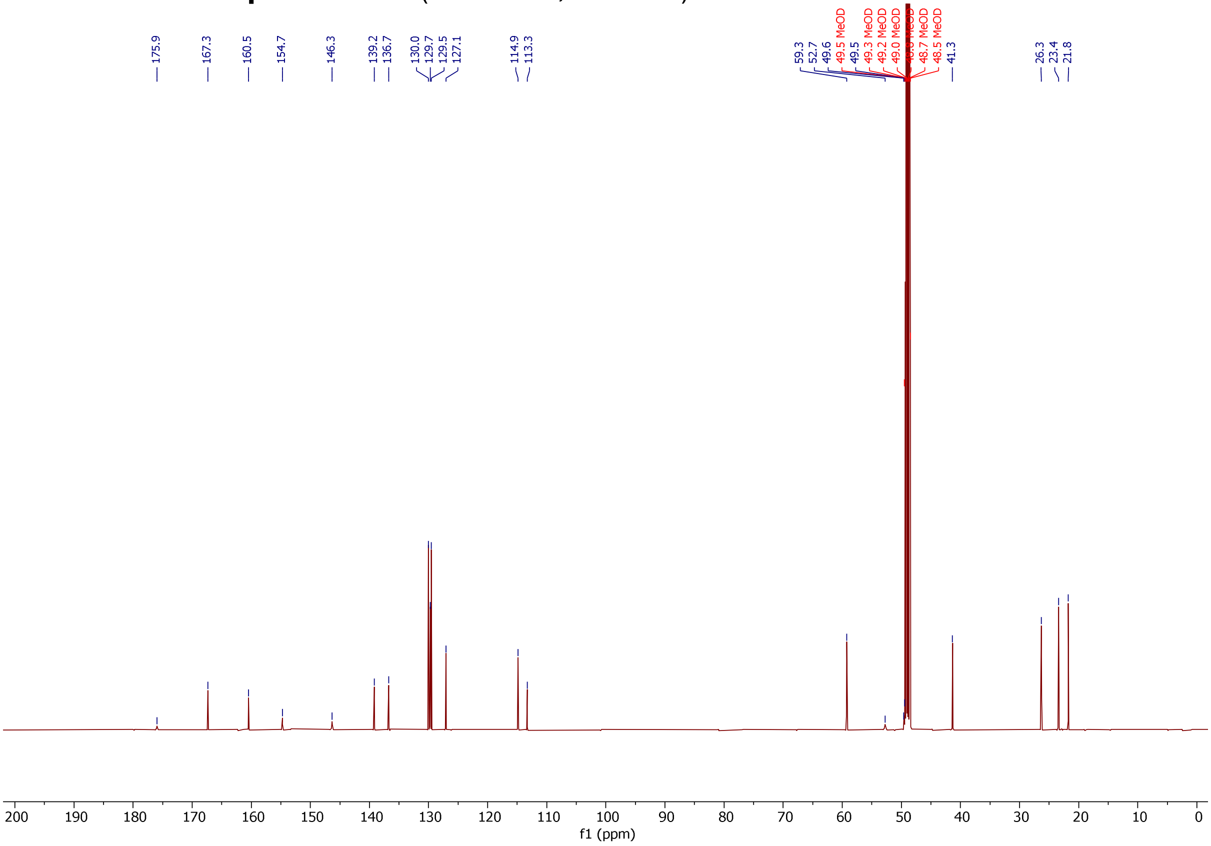

Compound 42b

MaxPeak: 100.00%
Ret_Time: 1.497 min

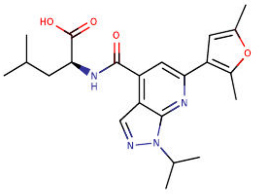

Mol Wt 412.48
Exact Mass 412.24

#	Time	Area%
1	1.497	100.00

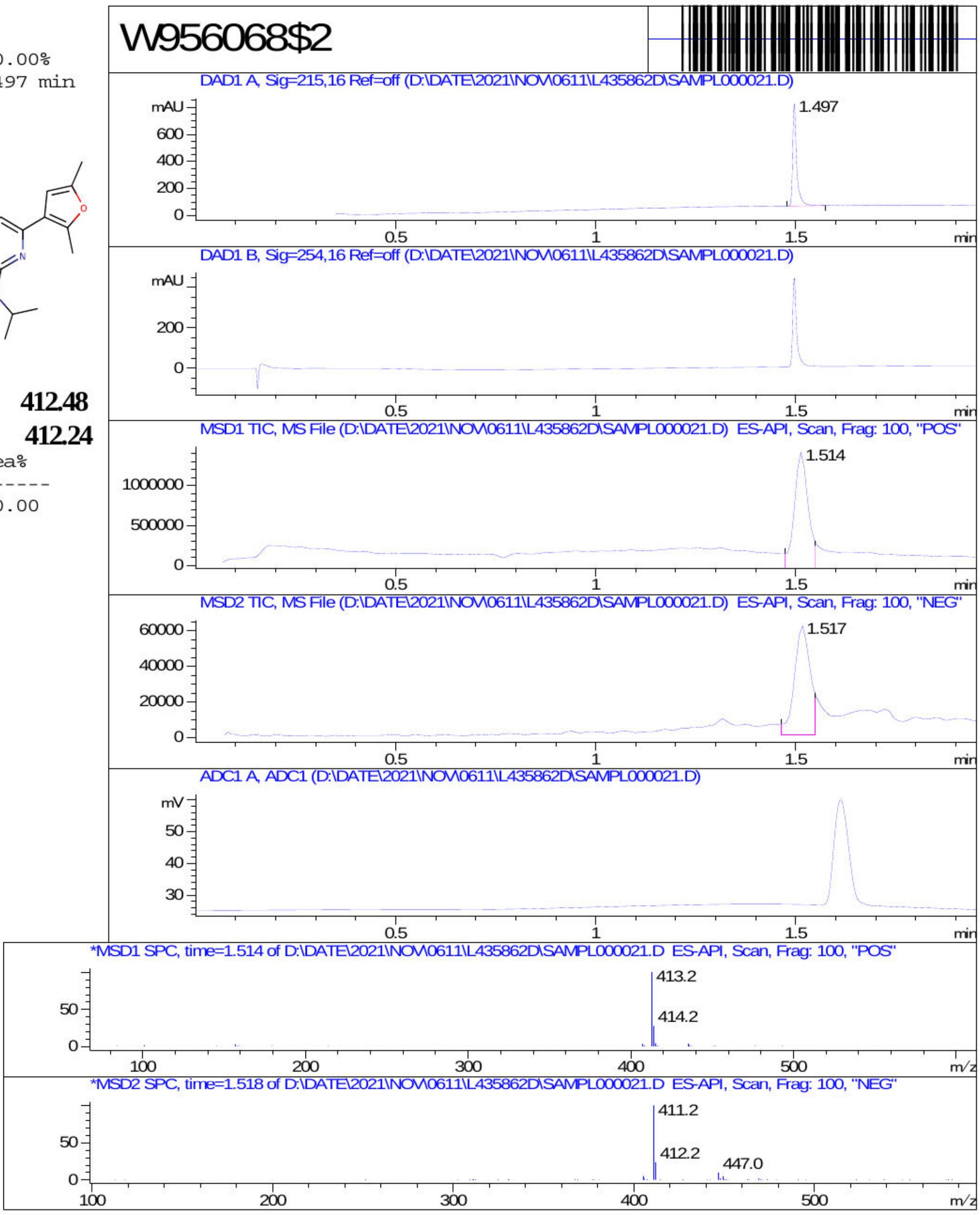

RT 1.514

RT 1.517

Inj.Date 05-Nov-21 A P2-C-03 - 4 - Acq. Method C:\CHEM32\-> ->

Compound 43b

MaxPeak: 100.00%
Ret_Time: 1.072 min

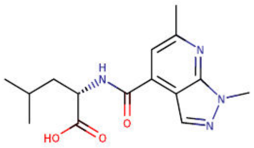

Mol Wt 304.34

Exact Mass 304.17

#	Time	Area%
1	1.072	100.00

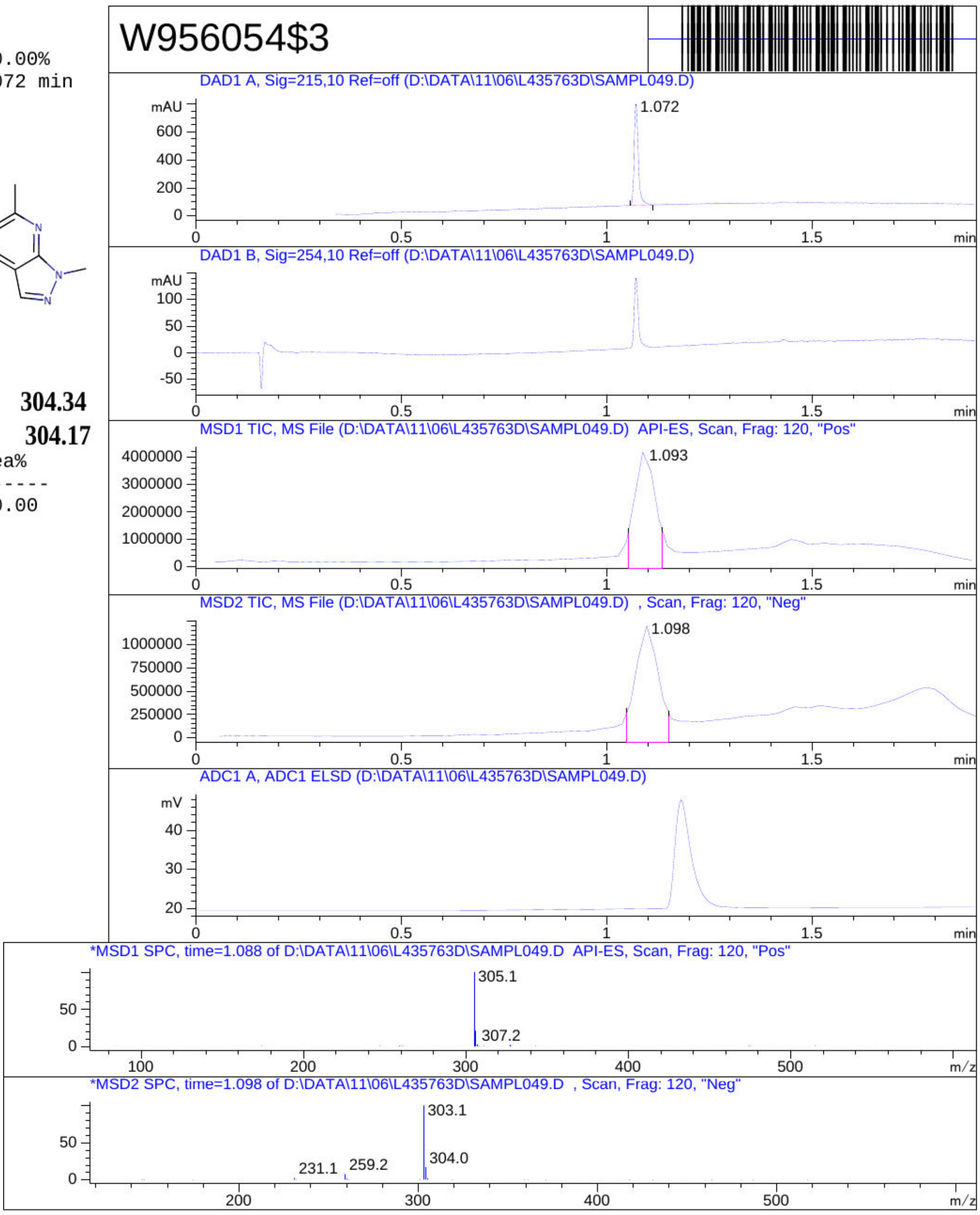

RT 1.093

RT 1.098

Inj.Date 11/6/2021 LB -SL- Acq. Method C:\HPCHEM\-> ->

Compound 44b

MaxPeak: 100.00%
Ret_Time: 1.174 min

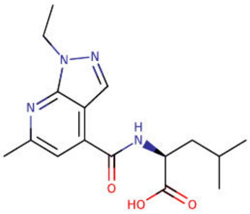

Mol Wt 318.37
Exact Mass 318.19

#	Time	Area%
1	1.174	100.00

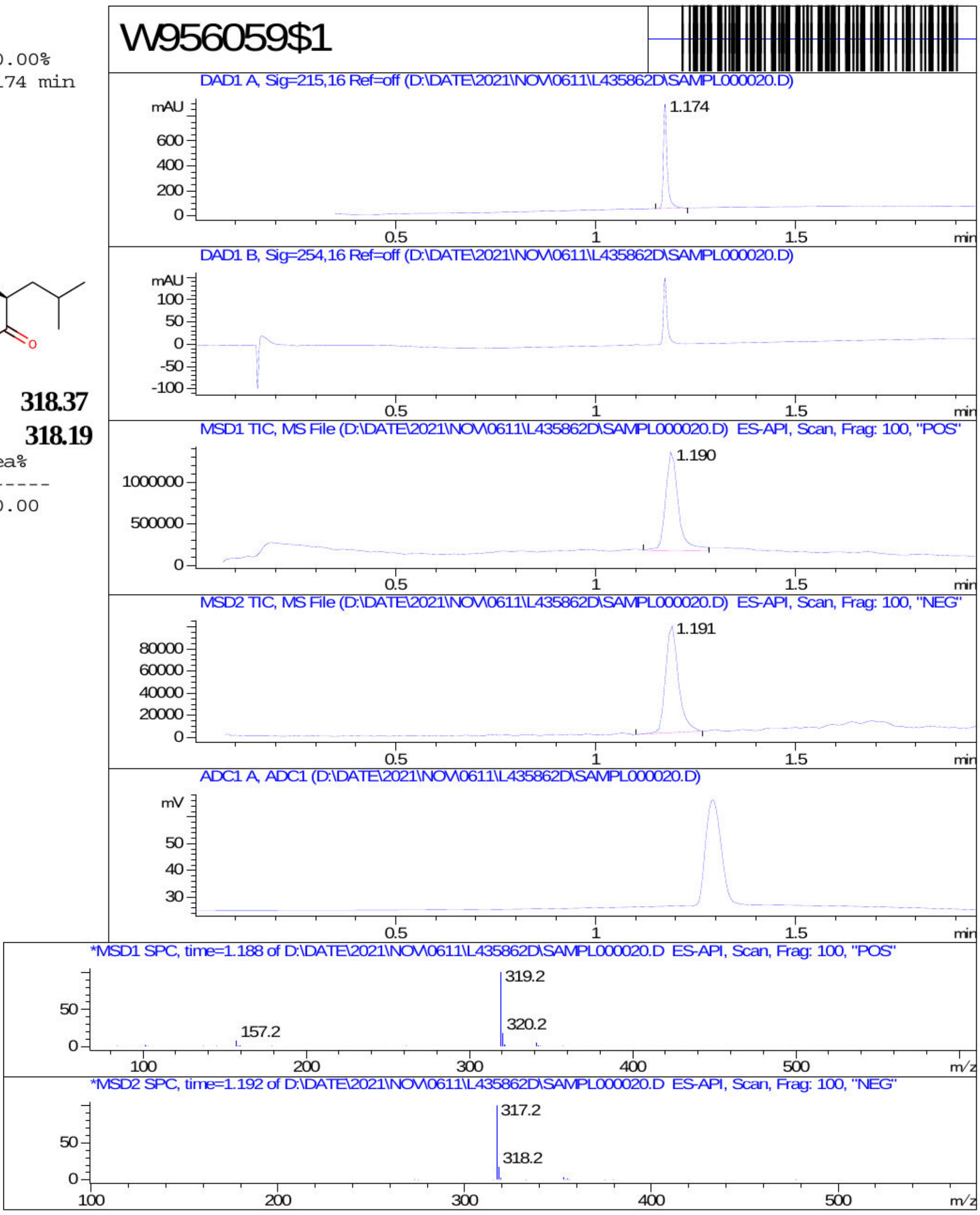

RT 1.190

RT 1.191

Inj.Date 05-Nov-21 A P2-C-02 - 4 - Acq. Method C:\CHEM32\-> ->

Compound 45b

MaxPeak: 100.00%
Ret_Time: 1.472 min

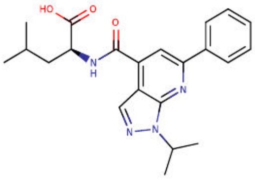

Mol Wt 394.47

Exact Mass 394.23

#	Time	Area%
1	1.472	100.00

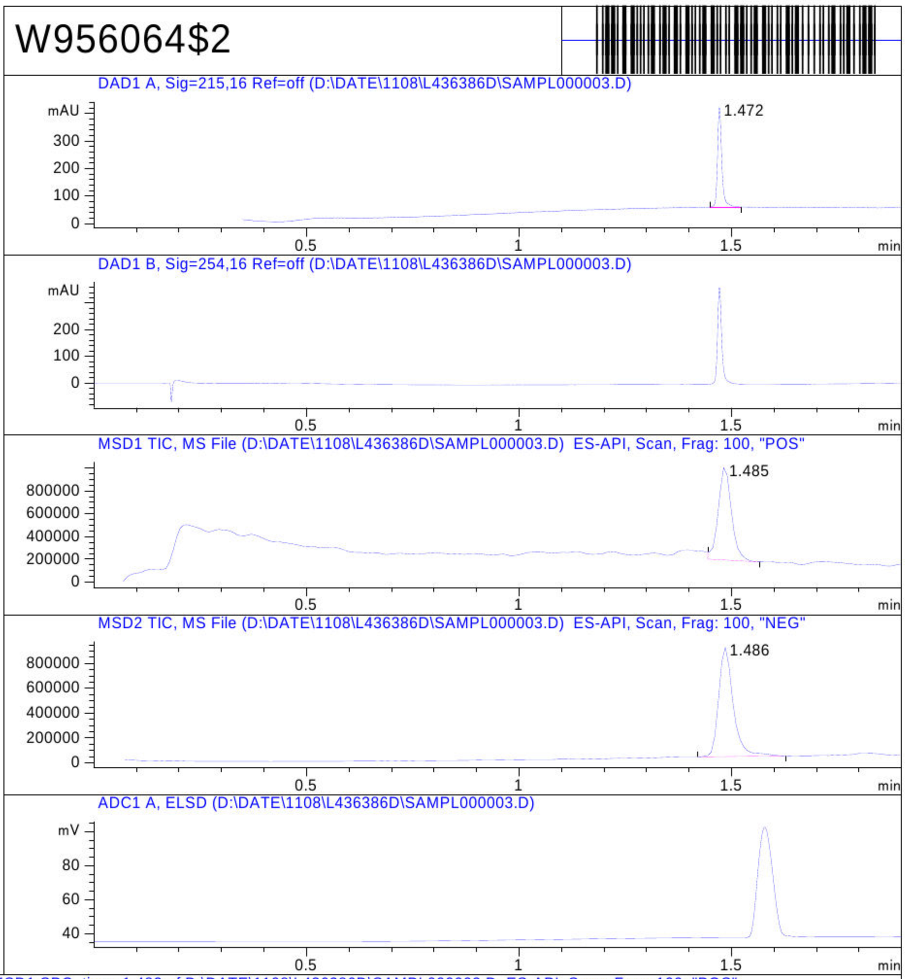

RT 1.485

RT 1.486

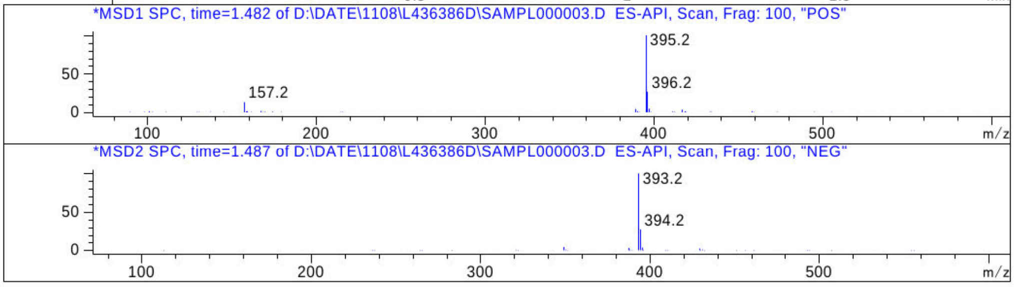

Inj.Date 11/8/2021 E -5- Acq. Method C:\CHEM32\-> ->

Compound 46b

MaxPeak: 100.00%
Ret_Time: 1.378 min

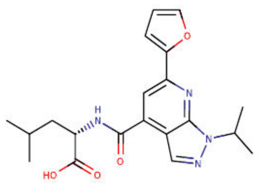

Mol Wt 384.43
Exact Mass 384.2

#	Time	Area%
1	1.378	100.00

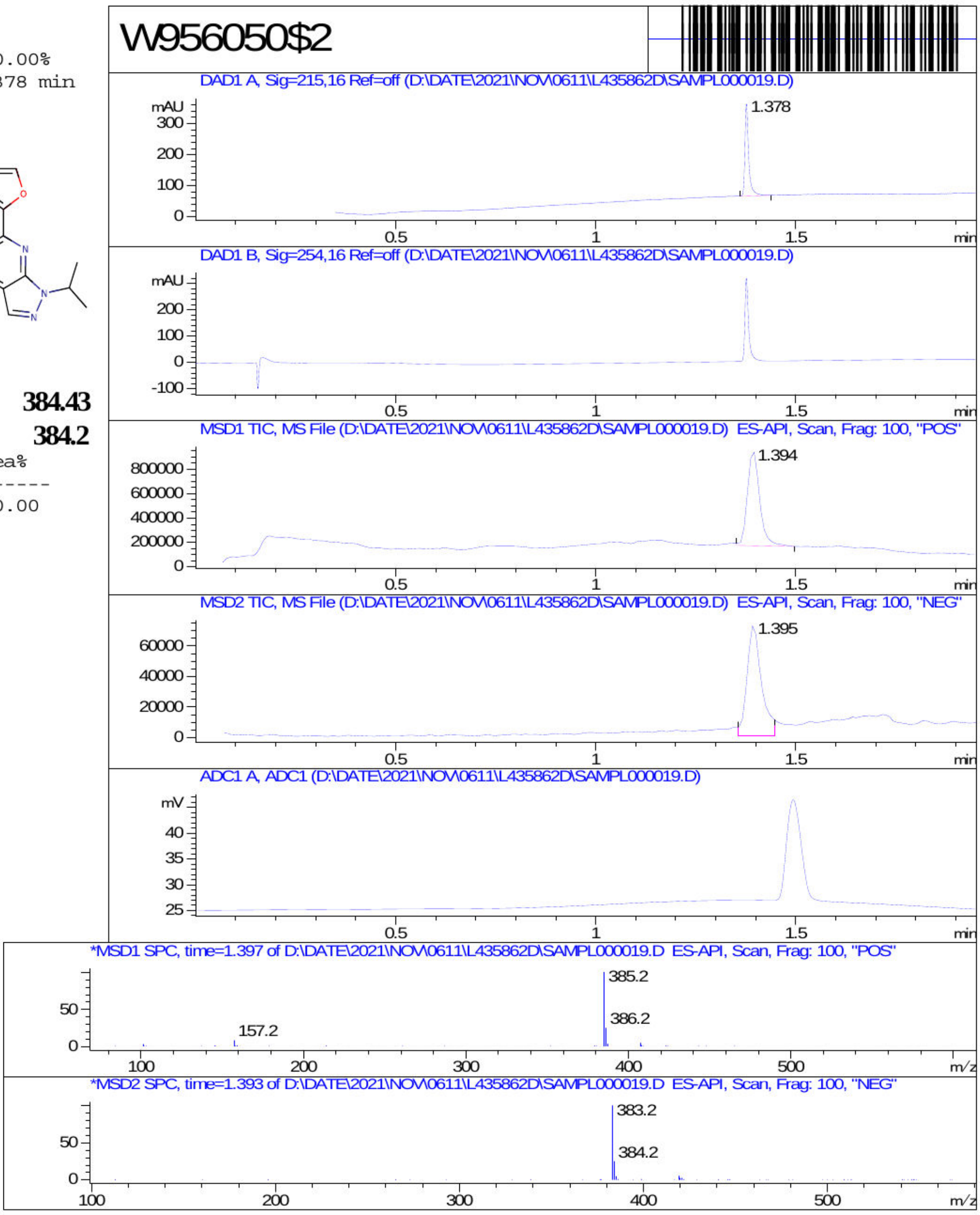

RT 1.394

RT 1.395

Inj.Date 05-Nov-21 A P2-C-01 - 4 - Acq. Method C:\CHEM32\-> ->

Compound 47b

MaxPeak: 100.00%
Ret_Time: 1.497 min

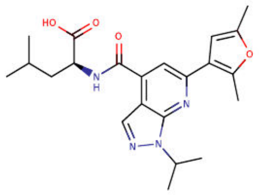

Mol Wt 412.48

Exact Mass 412.24

#	Time	Area%
1	1.497	100.00

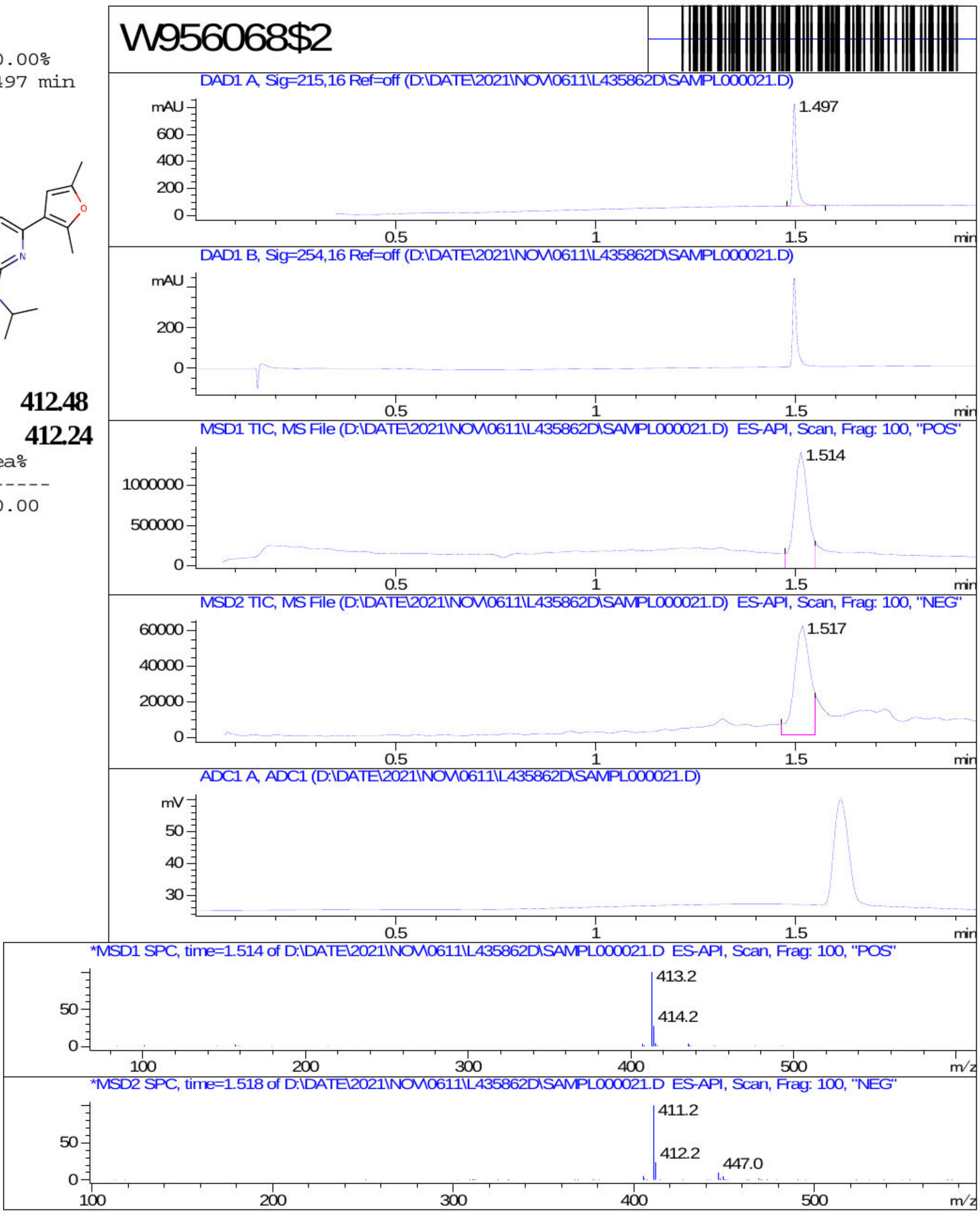

Inj.Date 05-Nov-21 A P2-C-03 - 4 - Acq. Method C:\CHEM32\-> ->

^{1}H NMR of **Compound 48b** (500 MHz, CD_3OD)

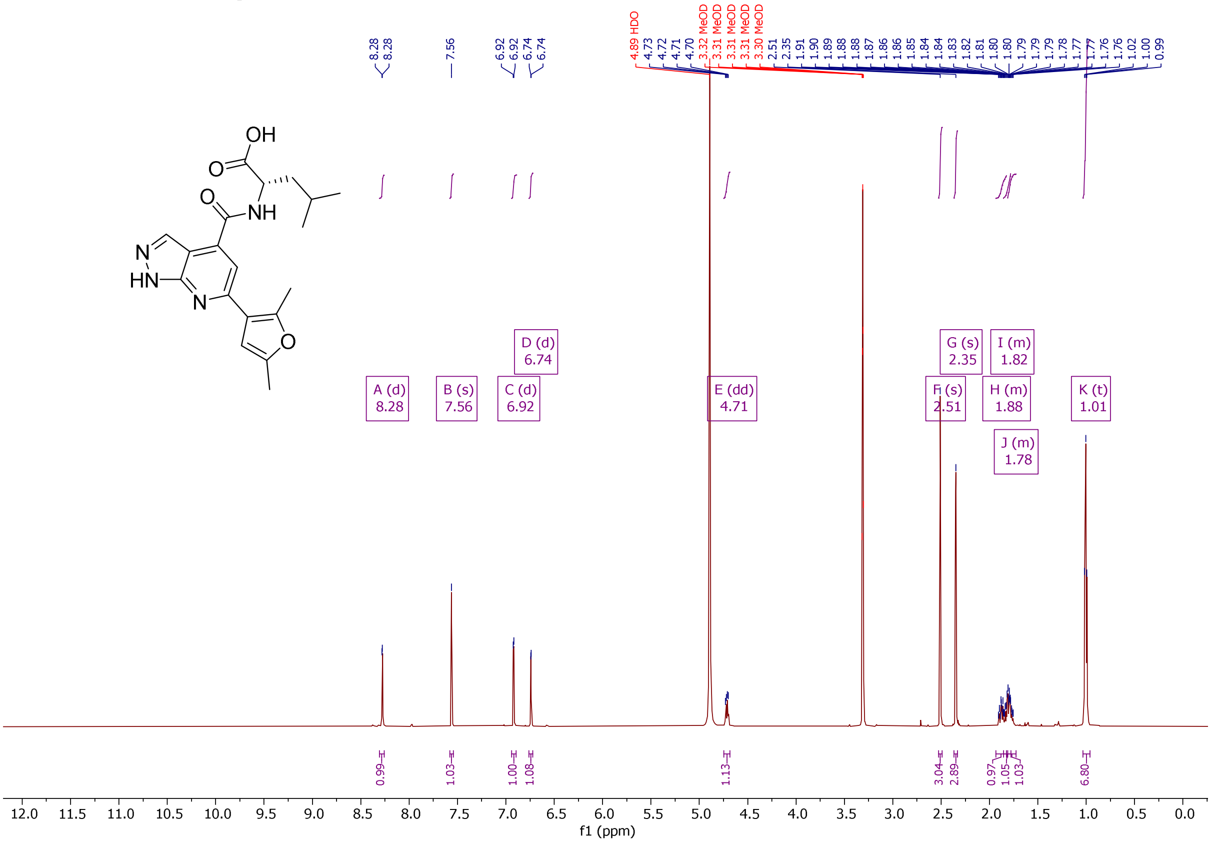

^{13}C NMR of **Compound 48b** (125 MHz, CD_3OD)

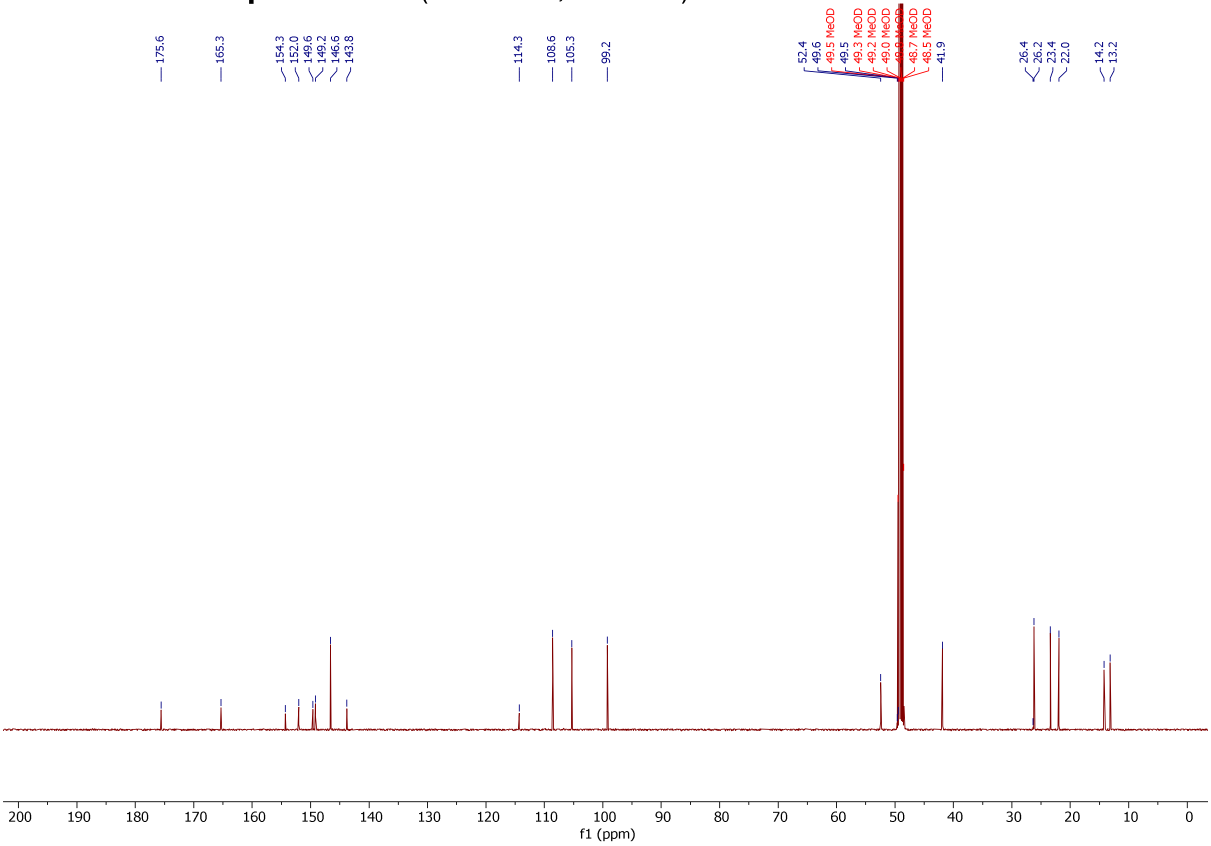

Compound 49b

MaxPeak: 100.00%
Ret_Time: 1.447 min

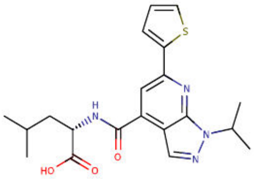

Mol Wt 400.5
Exact Mass 400.18

#	Time	Area%
1	1.447	100.00

RT 1.465

RT 1.466

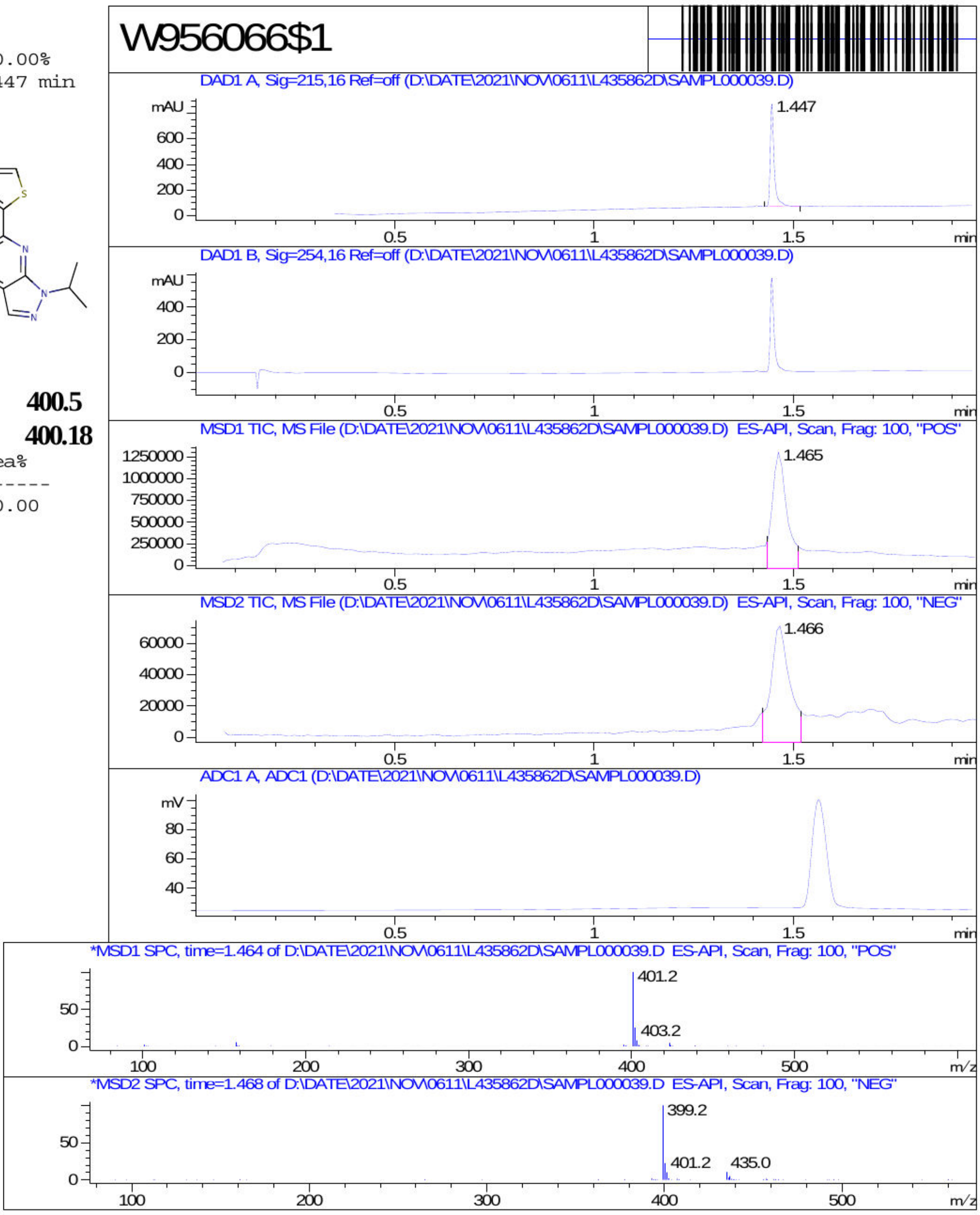

Inj.Date 05-Nov-21 A P2-E-04 - 4 - Acq. Method C:\CHEM32\-> ->

Compound 50b

MaxPeak: 100.00%
Ret_Time: 1.501 min

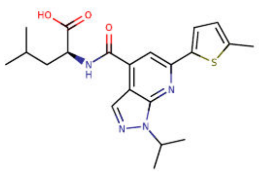

Mol Wt 414.52
Exact Mass 414.2

#	Time	Area%
1	1.501	100.00

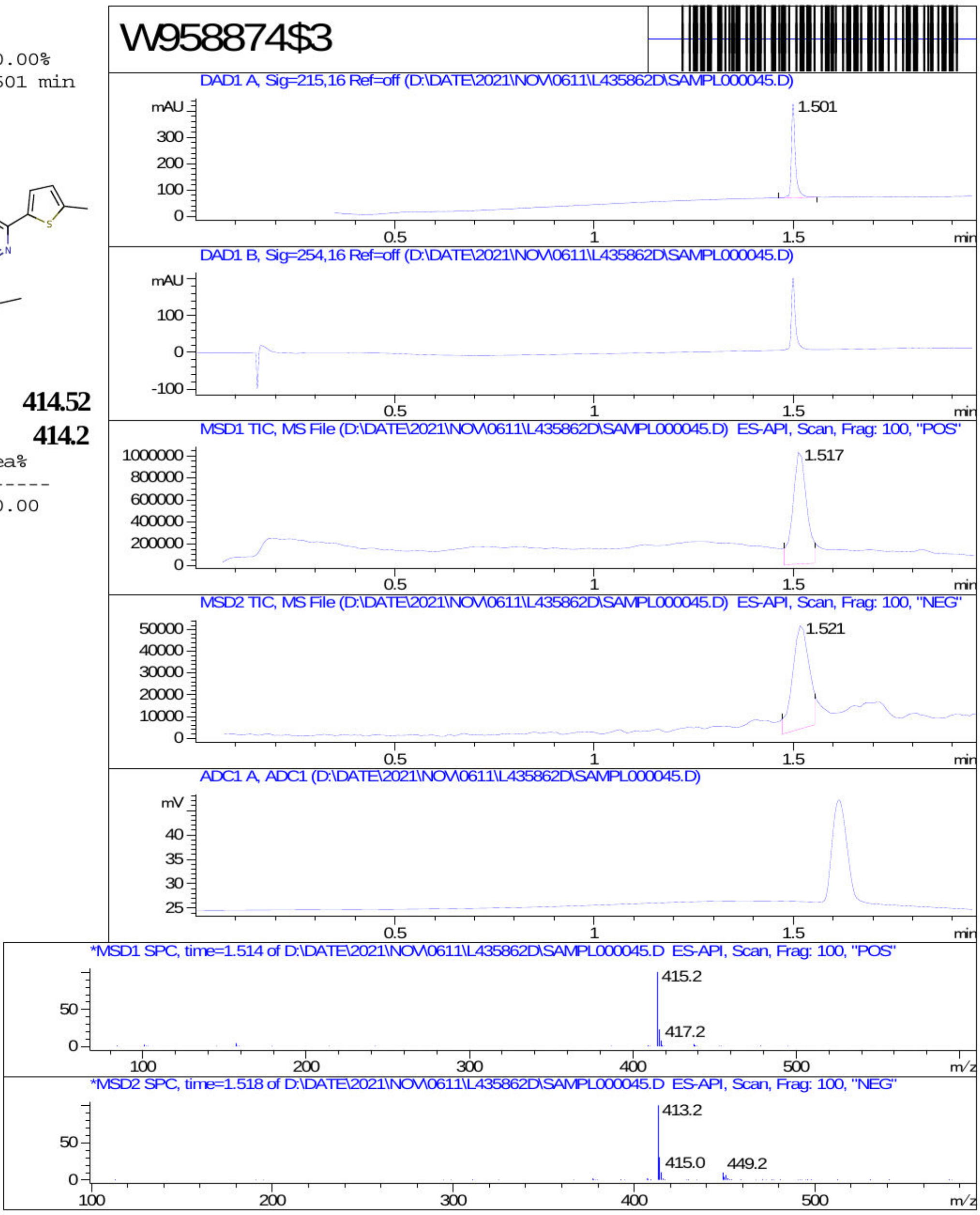

RT 1.517

RT 1.521

Inj.Date 06-Nov-21 A P2-F-01 - 4 - Acq. Method C:\CHEM32\-> ->

Compound 51b

MaxPeak: 100.00%
Ret_Time: 1.473 min

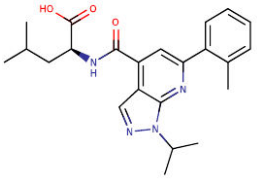

Mol Wt 408.49
Exact Mass 408.25

#	Time	Area%
1	1.473	100.00

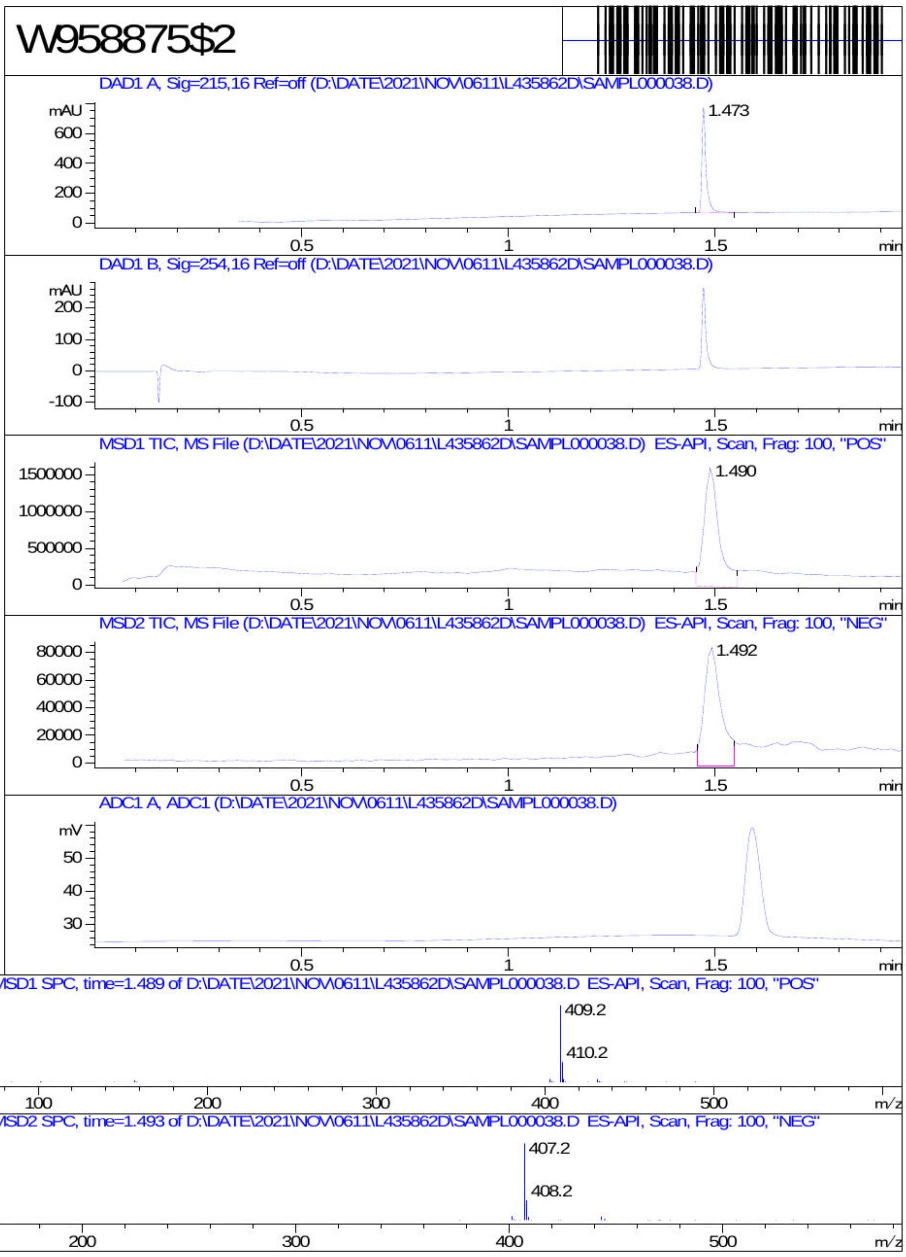

RT 1.490

RT 1.492

Inj.Date 05-Nov-21   A   P2-E-03   - 4 -   Acq. Method C:\CHEM32\->   ->

Compound 52b

MaxPeak: 100.00%
Ret_Time: 1.502 min

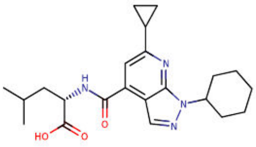

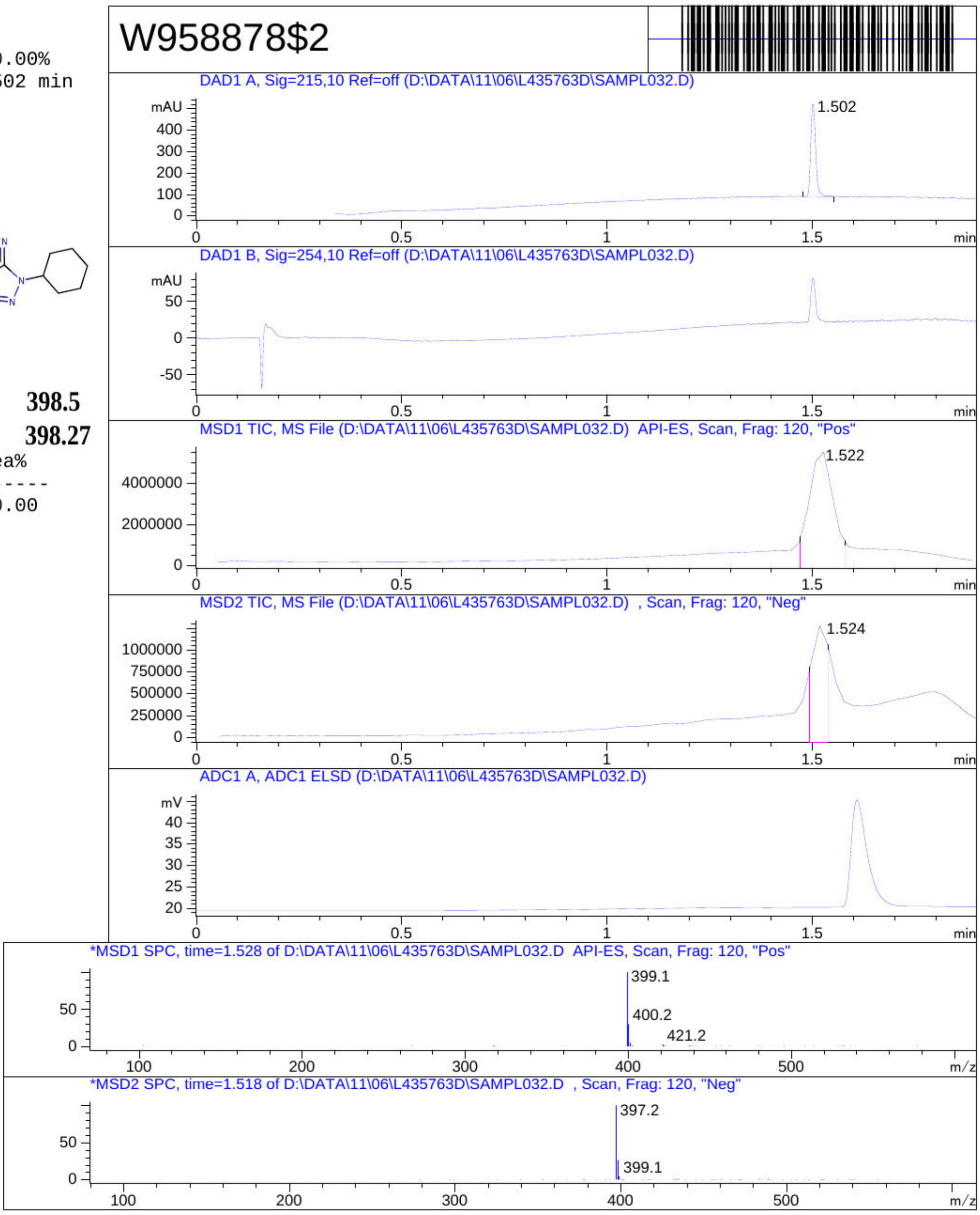

Mol Wt 398.5
Exact Mass 398.27

#	Time	Area%
1	1.502	100.00

RT 1.522

RT 1.524

Inj.Date 11/6/2021 LB -SL- Acq. Method C:\HPCHEM\-> ->

Compound 53b

MaxPeak: 100.00%
Ret_Time: 1.381 min

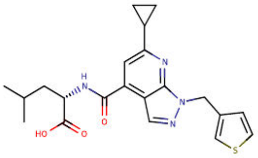

Mol Wt 412.5
Exact Mass 412.18

#	Time	Area%
1	1.381	100.00

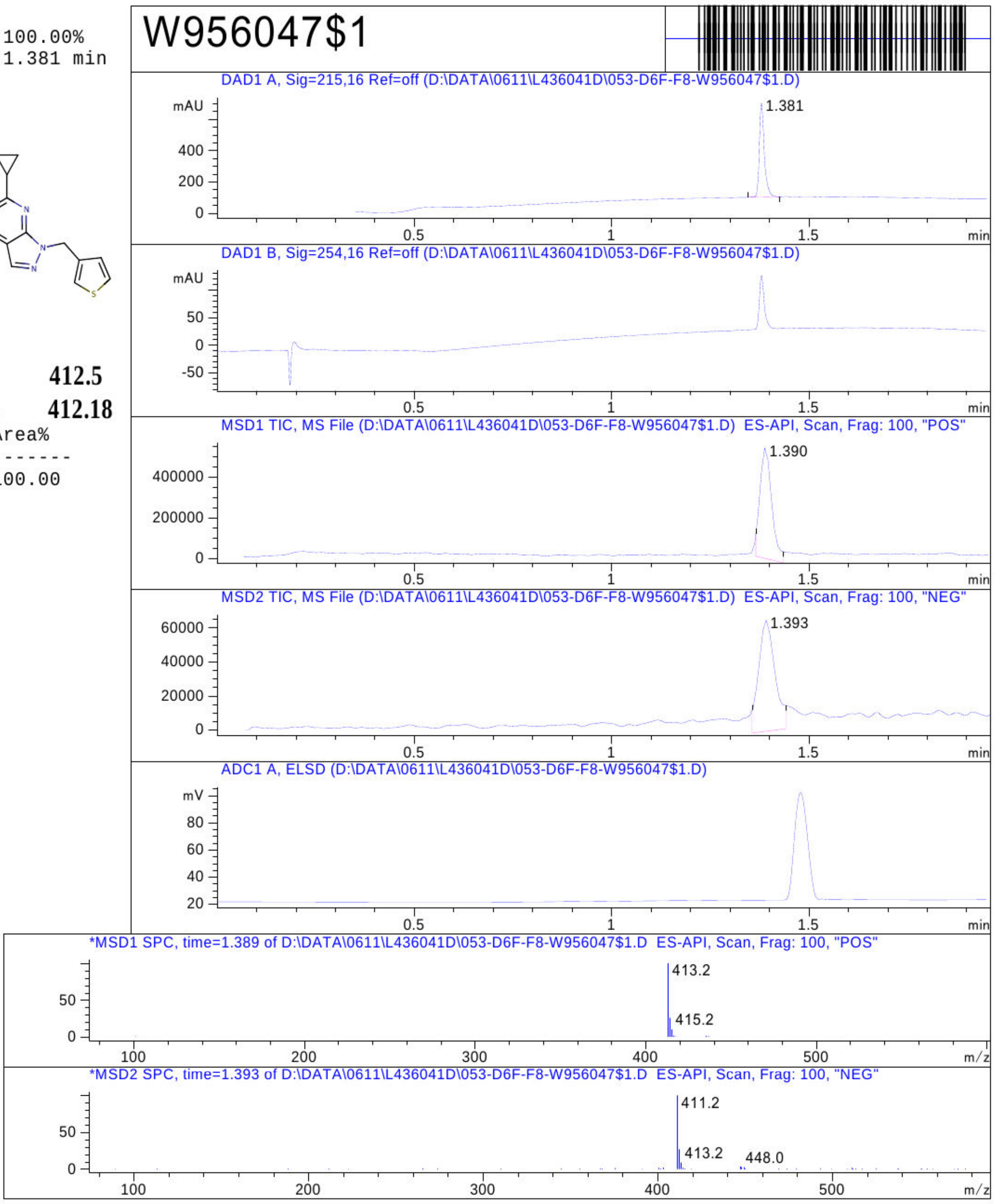

RT 1.390

RT 1.393

Inj.Date 11/5/2021 H -9- Acq. Method C:\Chem32\-> ->

^{1}H NMR of **Compound 54b** (500 MHz, CD_3OD)

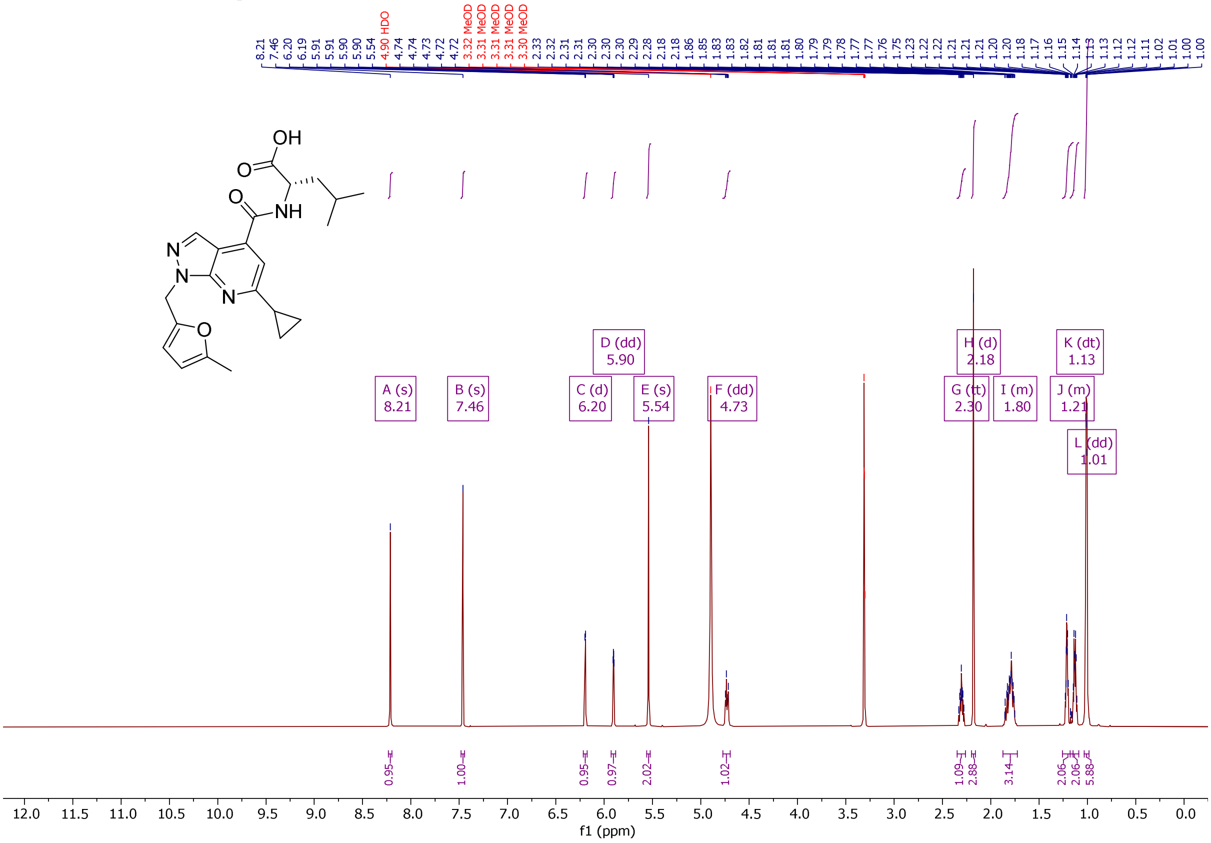

^{13}C NMR of **Compound 54b** (125 MHz, CD_3OD)

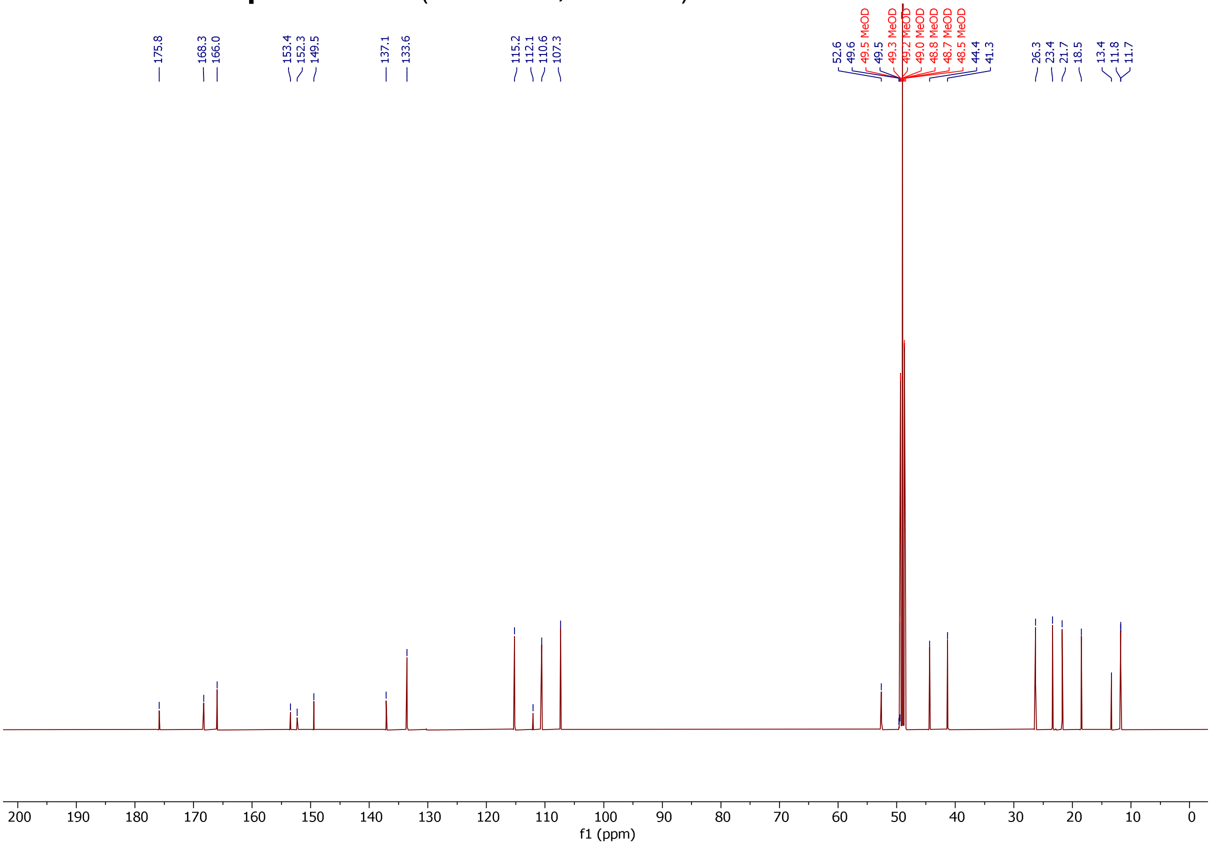

Compound 55b

MaxPeak: 100.00%
Ret_Time: 1.418 min

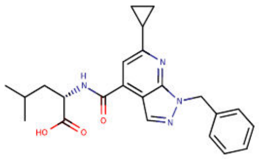

Mol Wt 406.48
Exact Mass 406.23

#	Time	Area%
1	1.418	100.00

W956055$1

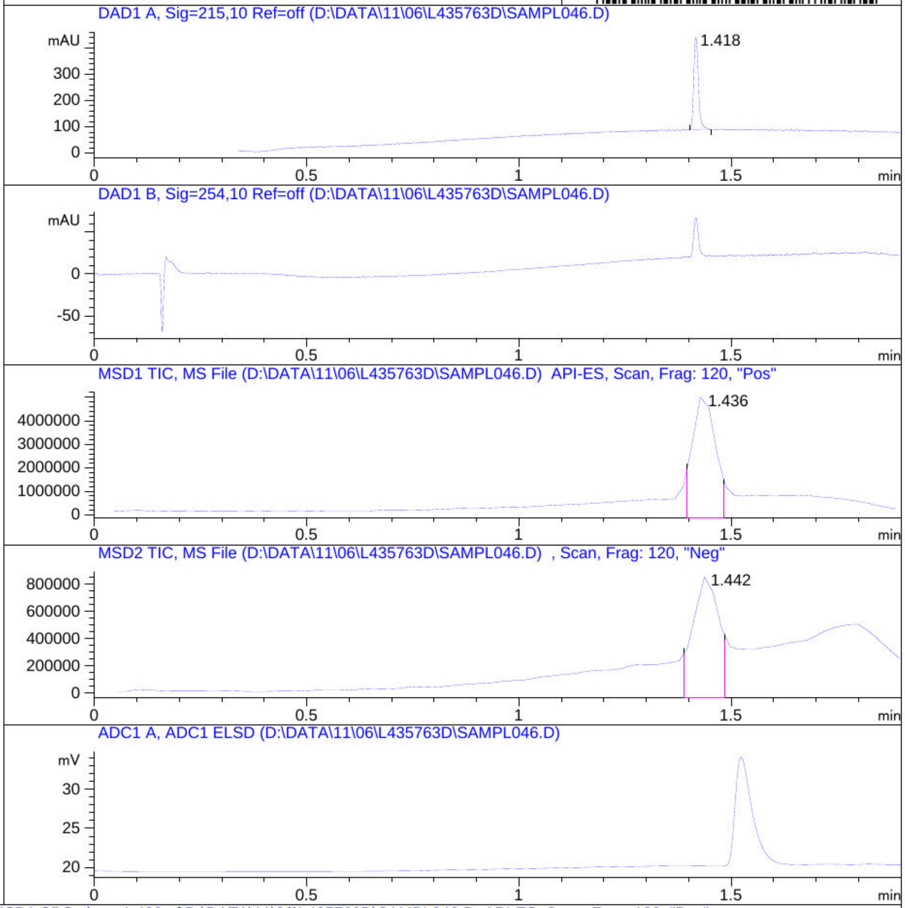

RT 1.436

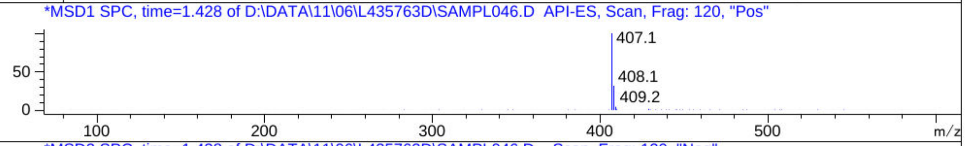

RT 1.442

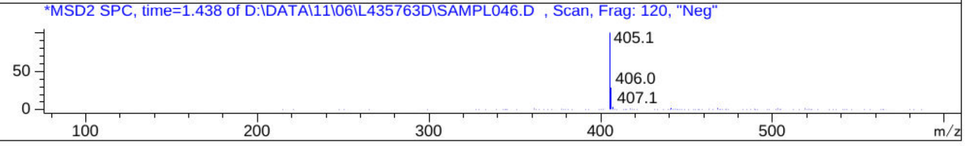

Inj.Date 11/6/2021 LB -SL- Acq. Method C:\HPCHEM\-> ->

^{1}H NMR of **Compound 56b** (500 MHz, CD_3OD)

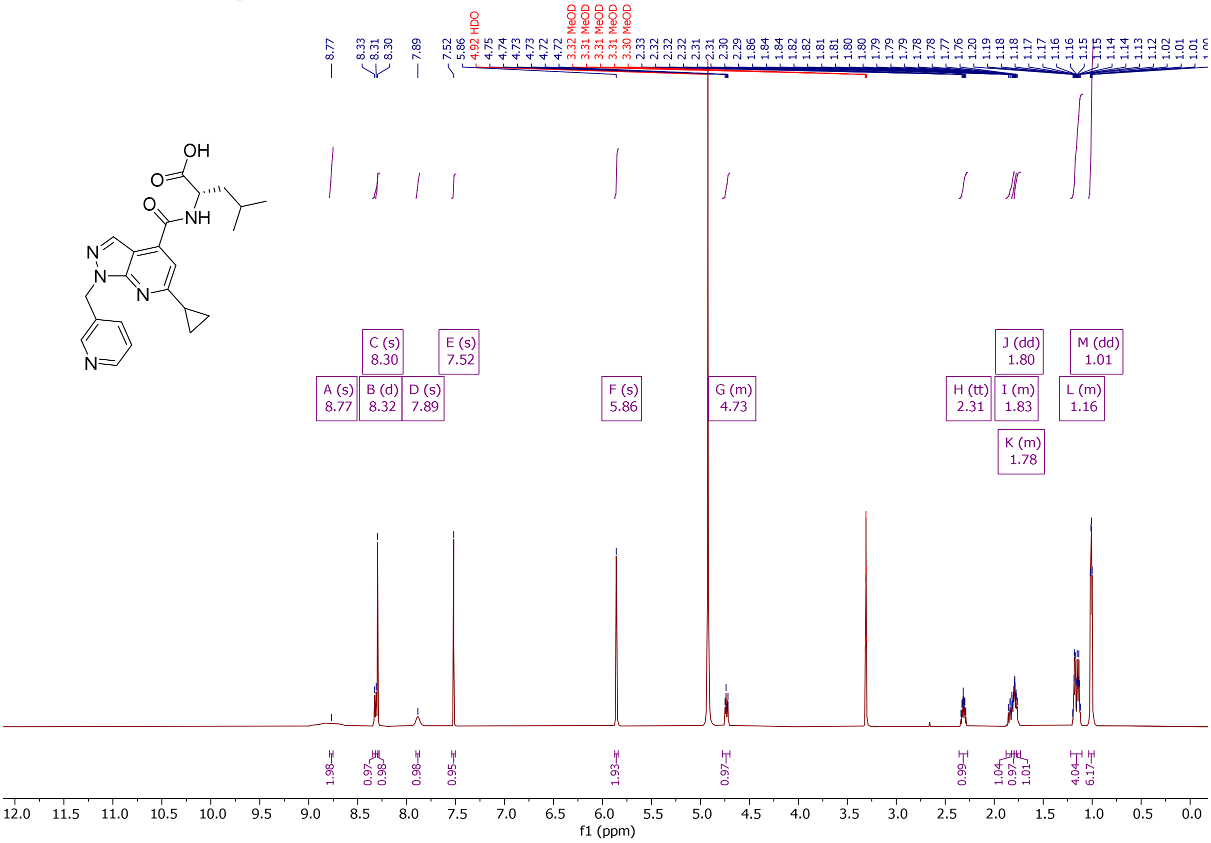

^{13}C NMR of **Compound 56b** (125 MHz, CD_3OD)

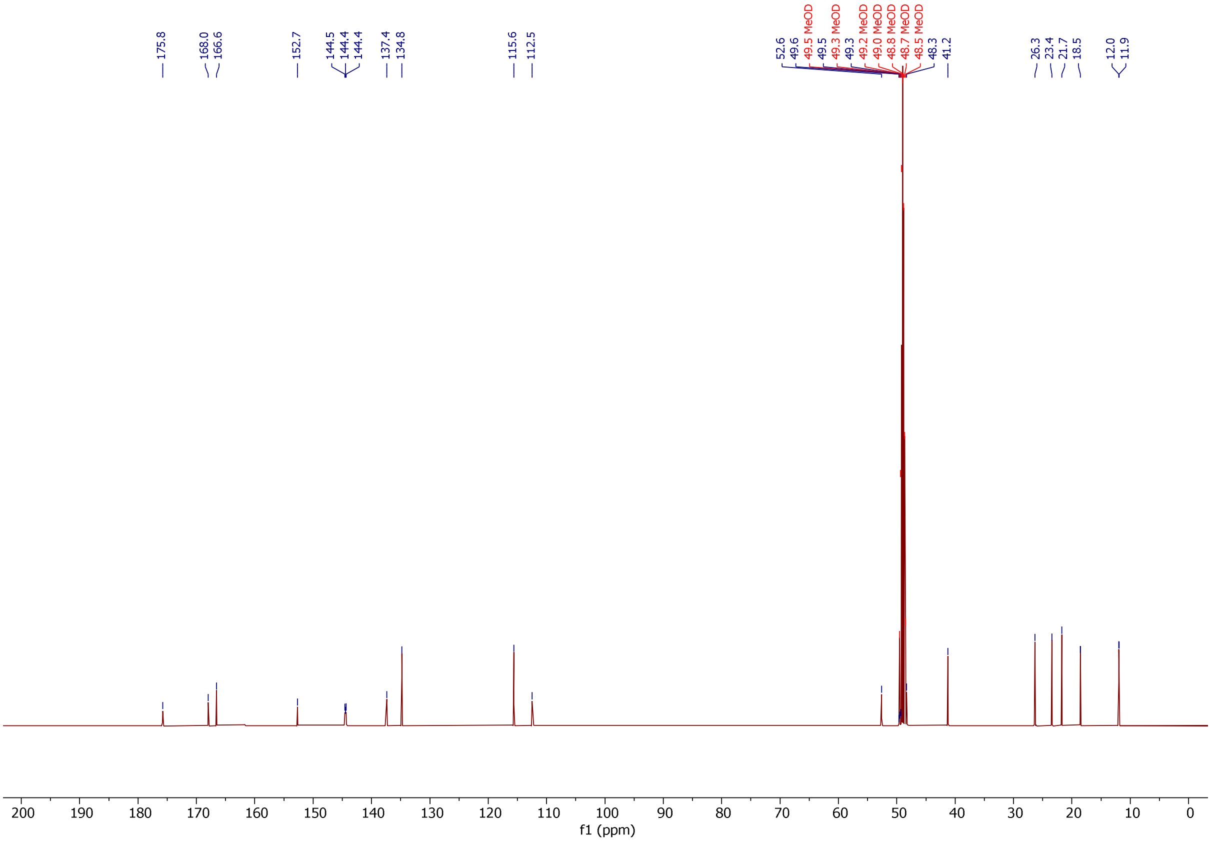

^{1}H NMR of **Compound 57b** (500 MHz, CD_3OD)

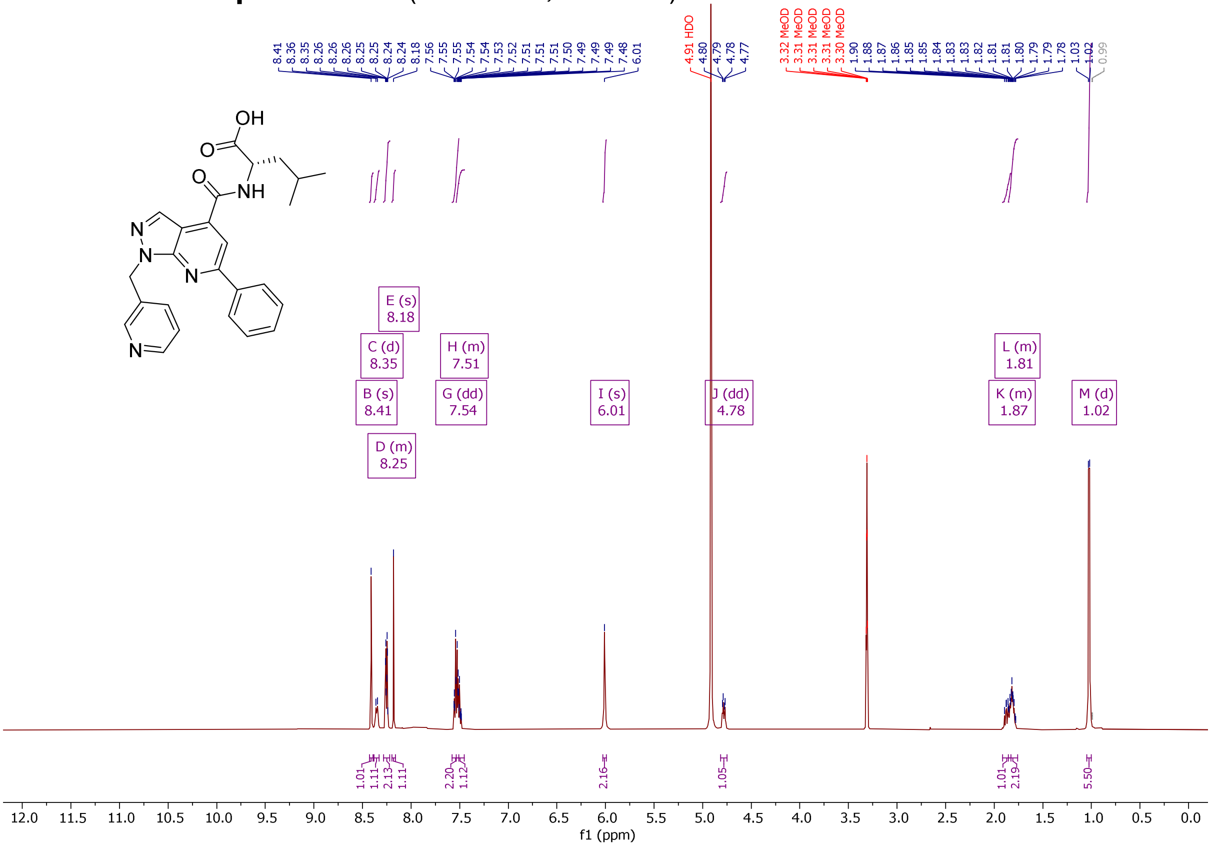

^{13}C NMR of **Compound 57b** (125 MHz, CD_3OD)

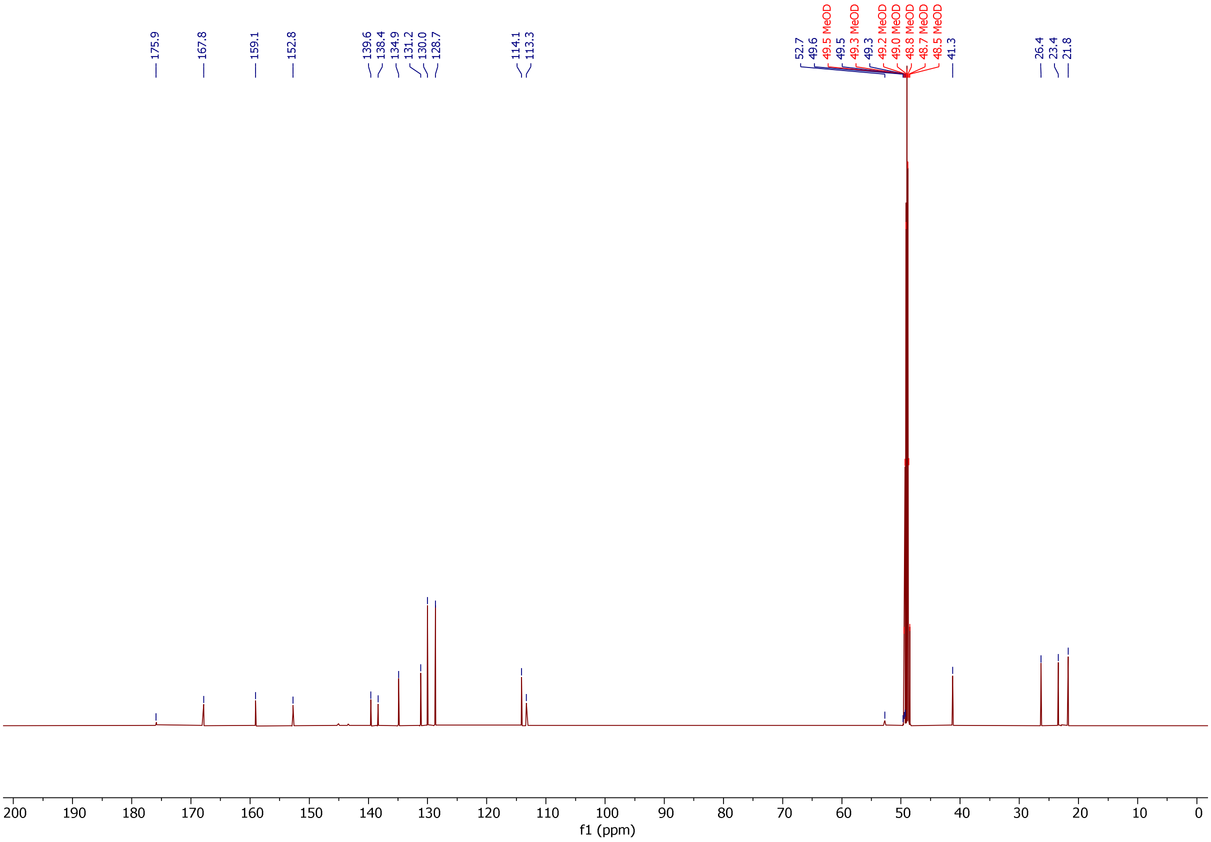

^{1}H NMR of **Compound 58b** (500 MHz, CD_3OD)

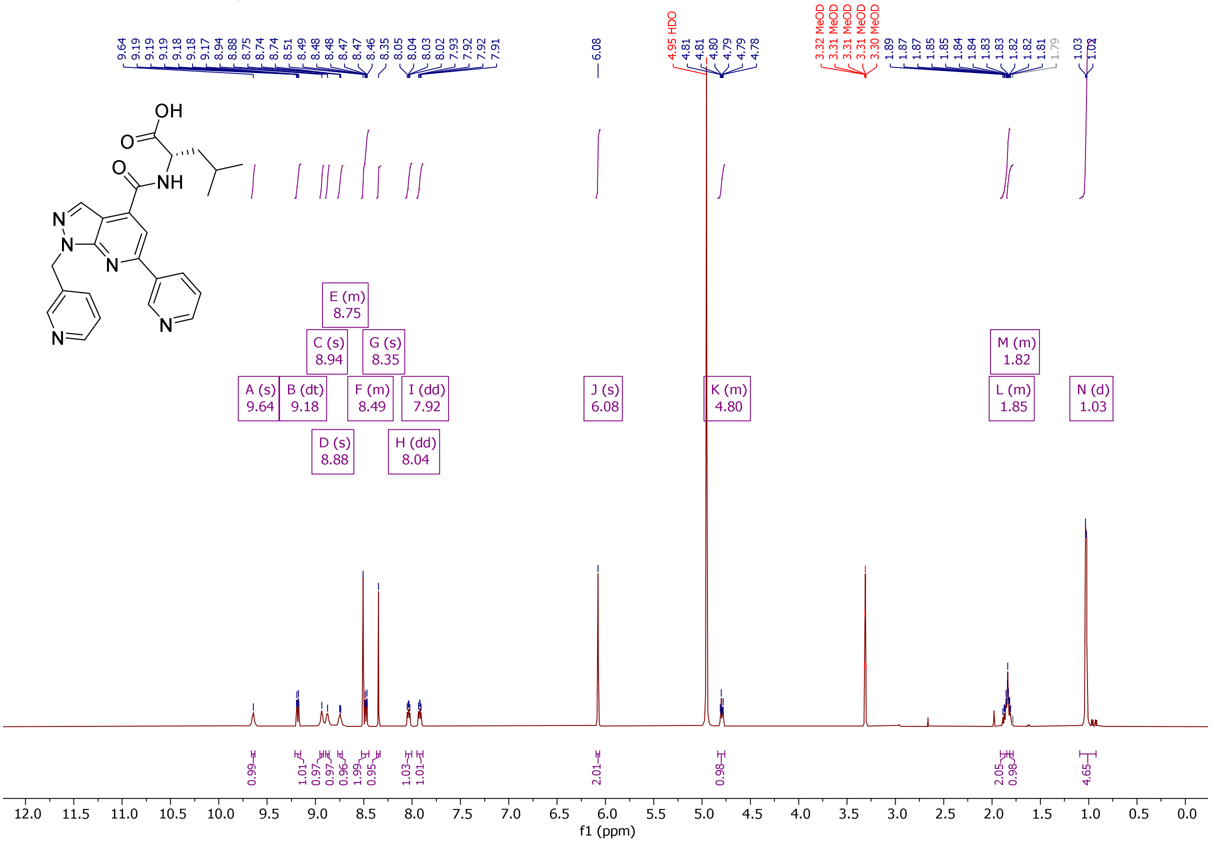

^{13}C NMR of **Compound 58b** (125 MHz, CD_3OD)

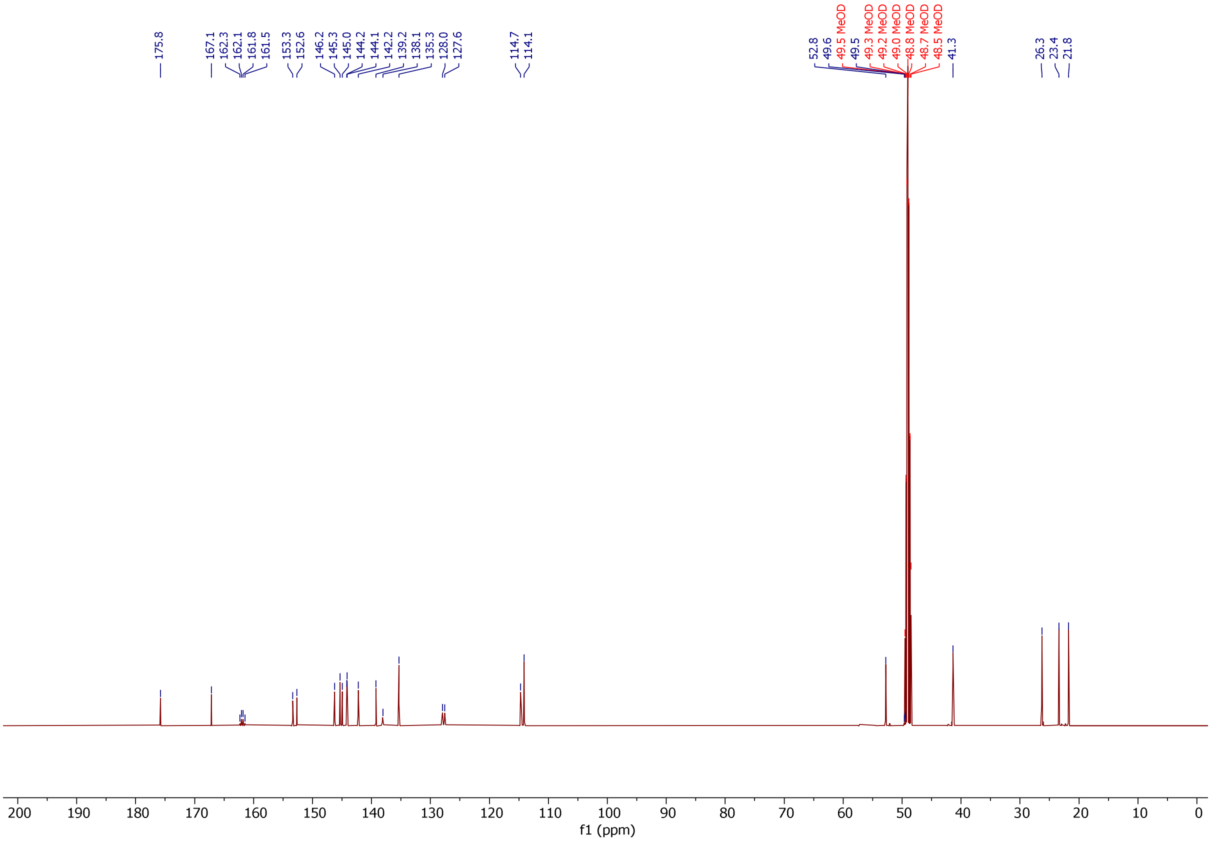

^{1}H NMR of **Compound 59b** (500 MHz, CD_3OD)

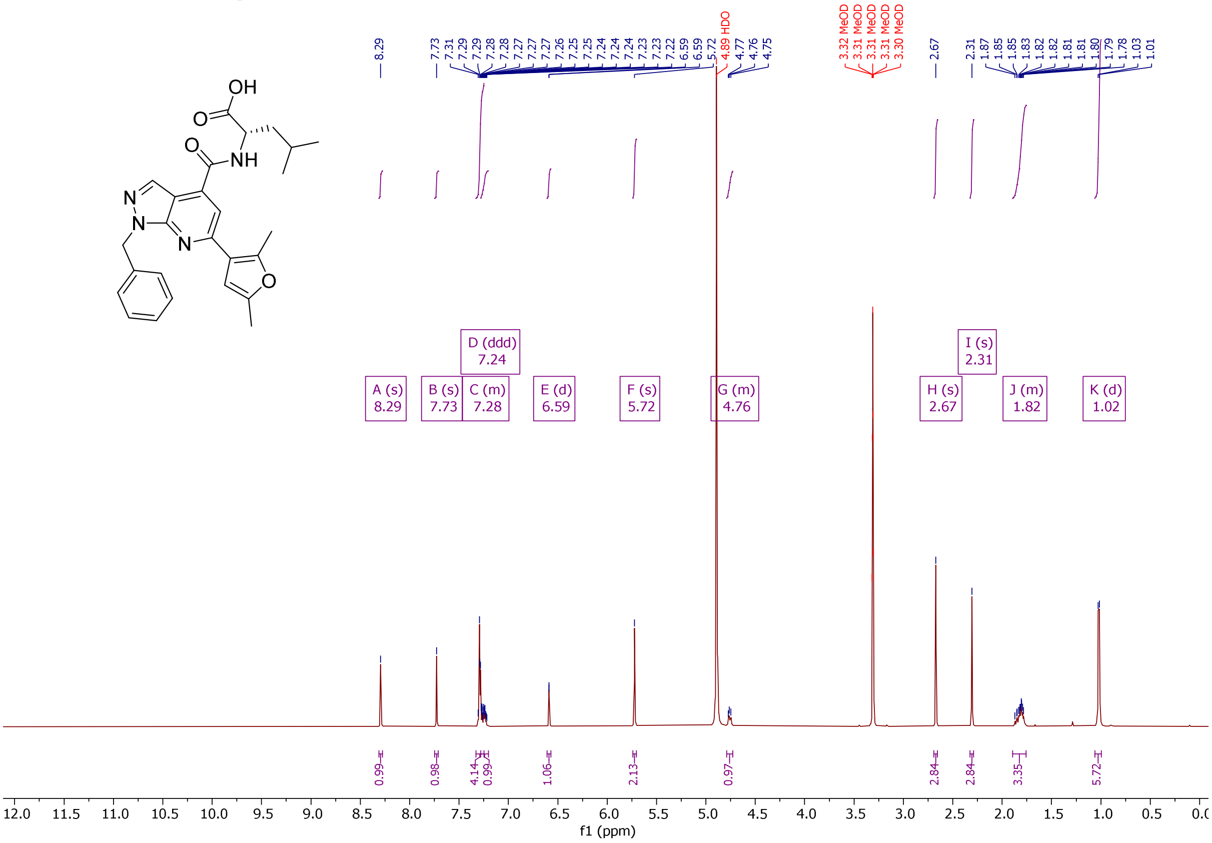

^{13}C NMR of **Compound 59b** (125 MHz, CD_3OD)

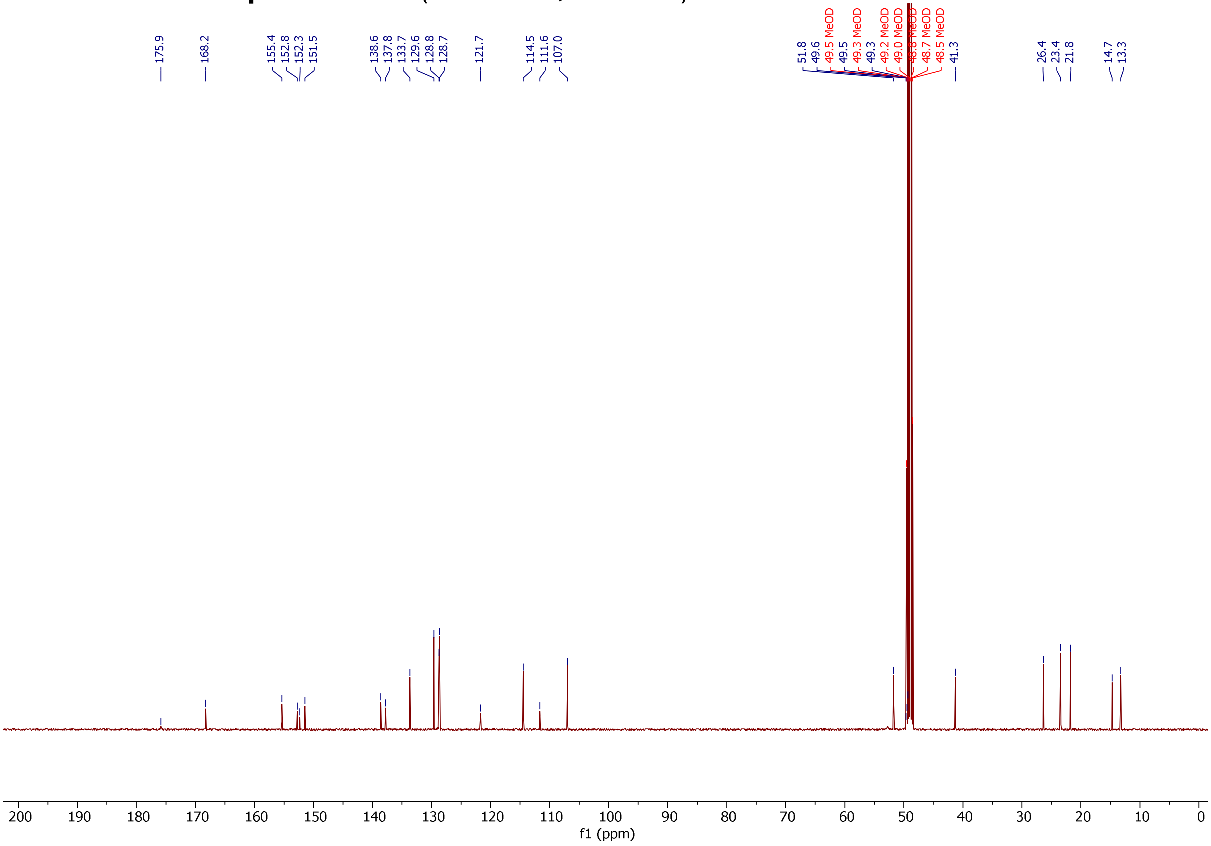

^{1}H NMR of **Compound 60b** (500 MHz, CD_3OD)

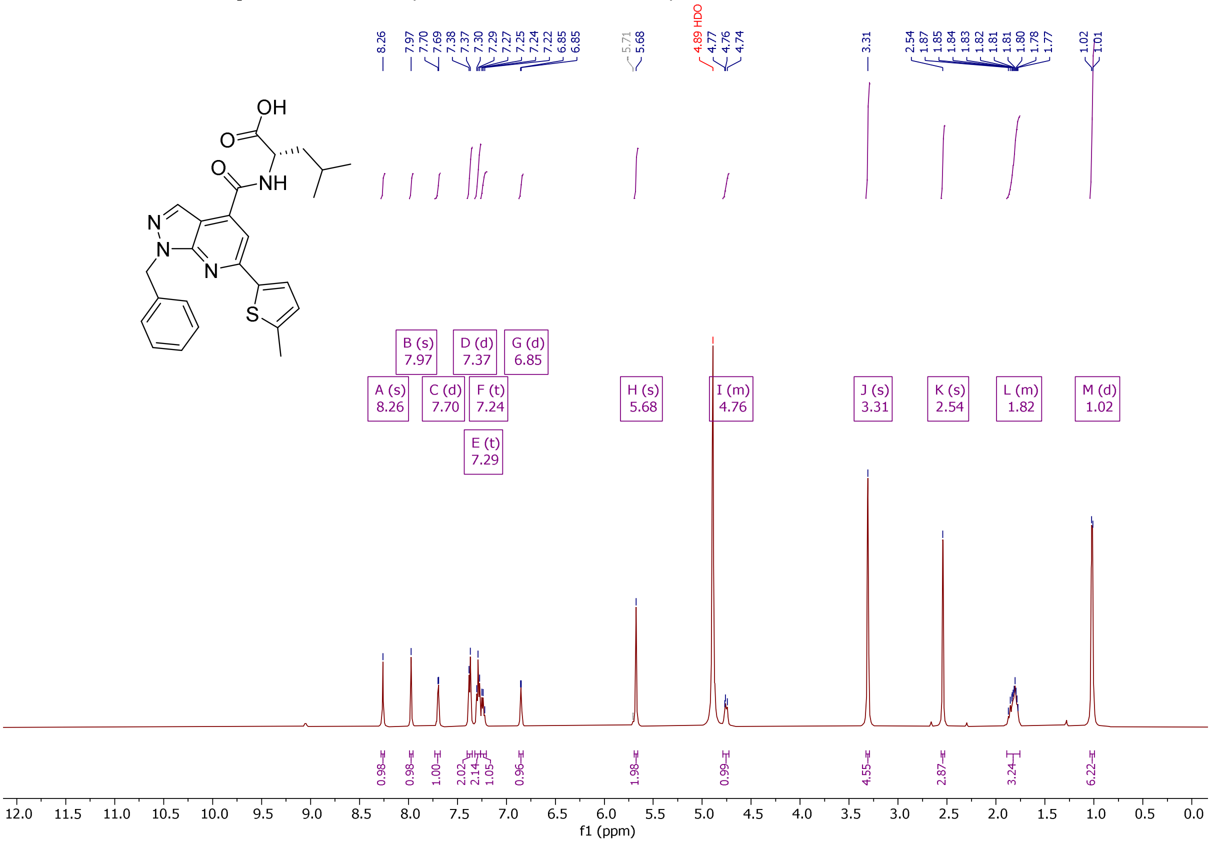

^{13}C NMR of **Compound 60b** (125 MHz, CD_3OD)

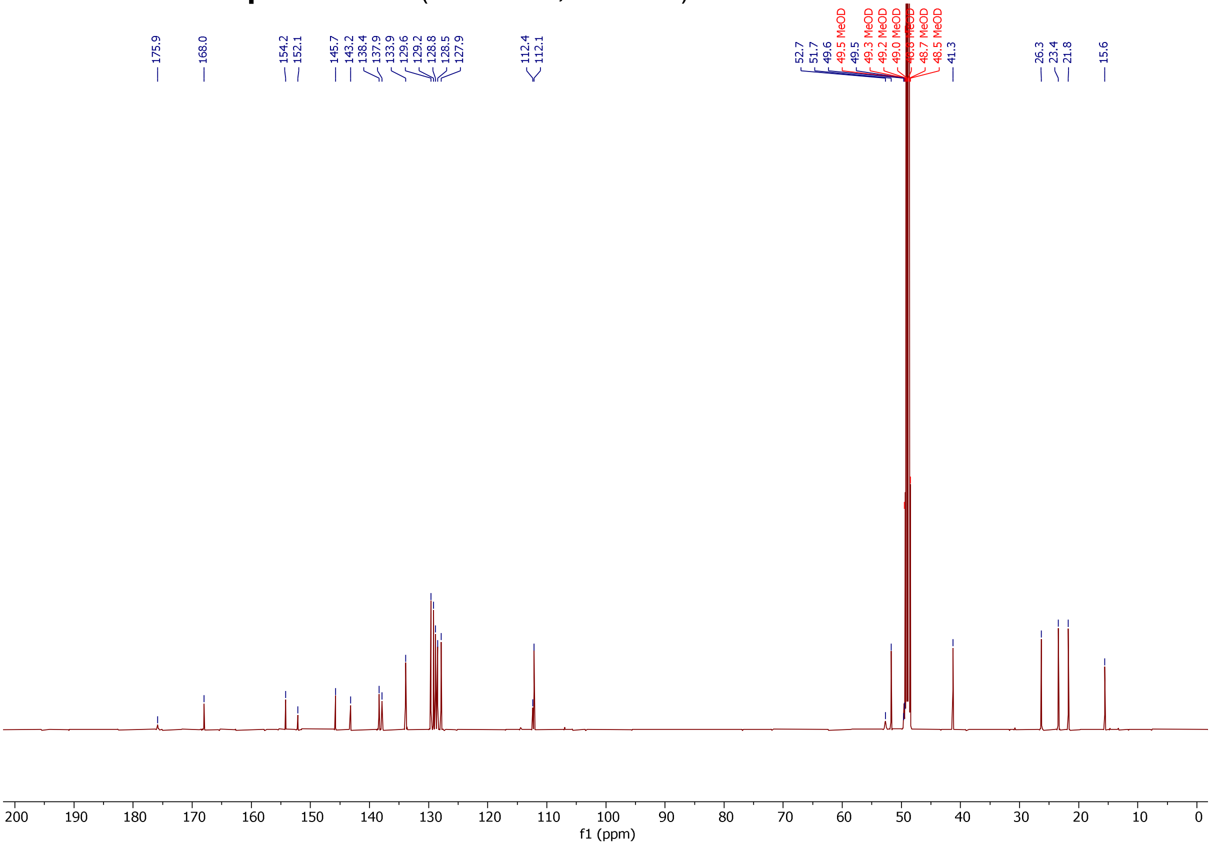

Supplementary references

1. Enamine REAL Space. https://enamine.net/compound-collections/real-compounds.
2. Wang, W.-W. *et al.* Structure-based design of non-hypertrophic apelin receptor modulator. *Cell* **187**, 1460-1475.e20 (2024).
3. Harris, J. A. *et al.* Selective G protein signaling driven by substance P–neurokinin receptor dynamics. *Nat. Chem. Biol.* **18**, 109–115 (2022).
4. You, C. *et al.* Structural basis for motilin and erythromycin recognition by motilin receptor. *Sci. Adv.* **9**, eade9020 (2023).
5. Zhuang, Y. *et al.* Molecular recognition of formylpeptides and diverse agonists by the formylpeptide receptors FPR1 and FPR2. *Nat. Commun.* **13**, 1054 (2022).
6. Zdrazil, B. *et al.* The ChEMBL Database in 2023: a drug discovery platform spanning multiple bioactivity data types and time periods. *Nucleic Acids Res.* **52**, D1180–D1192 (2024).
7. RDKit: Open-source cheminformatics. https://www.rdkit.org.
8. Bender, B. J. *et al.* A practical guide to large-scale docking. *Nat. Protoc.* **16**, 4799–4832 (2021).
9. Cheng, Y. & Prusoff, W. H. Relationship between the inhibition constant (K_I) and the concentration of inhibitor which causes 50 per cent inhibition (I_{50}) of an enzymatic reaction. *Biochem. Pharmacol.* **22**, 3099–3108 (1973).
10. Vonrhein, C. *et al.* Data processing and analysis with the autoPROC toolbox. *Acta Crystallogr. D* **67**, 293–302 (2011).
11. Kabsch, W. XDS. *Acta Crystallogr. D* **66**, 125–132 (2010).
12. Evans, P. R. & Murshudov, G. N. How good are my data and what is the resolution? *Acta Crystallogr. D* **69**, 1204–1214 (2013).

13. Tickle, I.J., Flensburg, C., Keller, P., Paciorek, W., Sharff, A., Vonrhein, C., Bricogne, G. STARANISO. *Cambridge, United Kingdom: Global Phasing Ltd.* (2018).
14. Croll, T. I. ISOLDE: a physically realistic environment for model building into low-resolution electron-density maps. *Acta Crystallogr. D* **74**, 519–530 (2018).
15. Murshudov, G. N., Vagin, A. A. & Dodson, E. J. Refinement of Macromolecular Structures by the Maximum-Likelihood Method. *Acta Crystallogr. D* **53**, 240–255 (1997).
16. Williams, C. J. *et al.* MolProbity: More and better reference data for improved all-atom structure validation. *Protein Sci.* **27**, 293–315 (2018).
17. Liebschner, D. *et al.* Macromolecular structure determination using X-rays, neutrons and electrons: recent developments in Phenix. *Acta Crystallogr. D* **75**, 861–877 (2019).
18. Scott, D. J., Kummer, L., Egloff, P., Bathgate, R. A. D. & Plückthun, A. Improving the apo-state detergent stability of NTS1 with CHESS for pharmacological and structural studies. *Biochim. Biophys. Acta* **1838**, 2817–2824 (2014).
19. Egloff, P. *et al.* Structure of signaling-competent neurotensin receptor 1 obtained by directed evolution in Escherichia coli. *Proc. Natl. Acad. Sci.* **111**, E655–E662 (2014).
20. Egloff, P., Deluigi, M., Heine, P., Balada, S. & Plückthun, A. A cleavable ligand column for the rapid isolation of large quantities of homogeneous and functional neurotensin receptor 1 variants from *E. coli*. *Protein Expr. Purif.* **108**, 106–114 (2015).

21. Ballesteros, J. A. & Weinstein, H. Integrated methods for the construction of three-dimensional models and computational probing of structure-function relations in G protein-coupled receptors. *Methods Neurosci.* **25**, 366–428 (1995).

22. Volochnyuk, D. M. *et al.* Approach to the Library of Fused Pyridine-4-carboxylic Acids by Combes-Type Reaction of Acyl Pyruvates and Electron-Rich Amino Heterocycles. *J. Comb. Chem.* **12**, 510–517 (2010).

23. Xing, Y., Zuo, J., Krogstad, P. & Jung, M. E. Synthesis and Structure–Activity Relationship (SAR) Studies of Novel Pyrazolopyridine Derivatives as Inhibitors of Enterovirus Replication. *J. Med. Chem.* **61**, 1688–1703 (2018).
