# Supplementary material for: Docking of virtual libraries identifies small-molecule agonists of neurotensin receptors with analgesic activity
Source: Nat Commun. 2026 Jul 22;17:6970. doi: 10.1038/s41467-026-74990-1 (PMC13392031; doi:10.1038/s41467-026-74990-1)
Supplement: Supplementary file 3 — Transparent Peer Review file [file 41467_2026_74990_MOESM3_ESM.pdf]

nature portfolio

Peer Review File

Docking of virtual libraries identifies small-molecule agonists of neurotensin receptors with analgesic activity

Corresponding Author: Professor Jens Carlsson

This file contains all reviewer reports in order by version, followed by all author rebuttals in order by version.

Version 0:

Reviewer comments:

Reviewer #1

(Remarks to the Author)
Summary:
This paper describes the identification and optimization of small-molecule agonists for neurotensin receptors (NTSRs), focusing on their potential as non-opioid analgesics. The authors employed a structure-based virtual screening approach, beginning with the design of two chemical libraries: a diverse library of 8.2 million commercially available anionic compounds and a focused library of 6.3 million compounds centered around a C-terminal leucine moiety, a key interaction for NTS1R activation. Computational docking of 14.5 million compounds against the NTS1R orthosteric site led to the selection of 39 candidates for synthesis and experimental evaluation.
The focused library yielded a notably higher hit rate (38%) compared to the diverse library (4%), with seven compounds confirmed as NTS1R activators. Subsequent structure-guided optimization, particularly of an initial hit (compound 24), resulted in submicromolar agonists (e.g., 28a, 30a) exhibiting varied signaling profiles. X-ray crystal structures of these optimized agonists complexed with NTS1R validated the computational binding models and elucidated molecular determinants of receptor activation. Significantly, one optimized ligand, compound 35a, demonstrated nanomolar affinity and selectivity for the human NTS2R subtype and produced antinociceptive effects in a rodent pain model without the hypotensive side effects associated with peptide agonists. The study underscores the utility of tailored virtual libraries for discovering ligands for peptide-binding GPCRs and offers promising lead compounds for further development of non-opioid pain therapeutics.

Major:
-Page 8: The authors should provide additional information about the optimization procedure. Details can be left for the Methods section, but a bit more info in the Results section would be helpful for the reader. For example, how many analogs of 24 and 25 were generated? How was the selection performed? What were the driving criteria for selection? Was the procedure identical to the original library screening selection or were learnings from the initial screening applied to the optimized compounds? Is this a reproducible approach? Could the selection criteria be encapsulated in an algorithm? If not, could machine learning based on the selections be used to infer the next selections?

-Figure 4: It looks like the modeled conformation of the thiophene is perpendicular to the attached phenyl. It is important to highlight issues like this, especially for a broad audience like Nature Communications. Many readers will not have the technical background to assess the models, so the authors would do a great service by elaborating on some of the model deficiencies. Furthermore, the authors could discuss the model assumptions and potential areas for improvement. For example, was the thiophene perpendicular in the original Dock pose? If so, was that due to the initial conformation generation? Could it have been fixed with an unrestrained minimization as opposed to the rigid-body minimization that the authors described? If not, a QM minimization would likely fix this. The reviewer is not proposing that the authors do any additional calculations, but they should highlight the model errors and potential solutions. This will help ground the paper in reality and not be as subjected to overhype.

-Page 33: The authors trained docking parameters based on known active and inactive compounds for NTSR. That point should be made clearly earlier in the manuscript. Reading the results, there is no mention of custom fitting based on existing data. This makes a big difference for people trying to assess the applicability of such a protocol for their target(s) of interest. Furthermore, the authors should explain in more detail which parameters were adjusted during this retrospective

optimization exercise. Was there a systematic exploration of parameters? Which parameters? How many enrichment trials were run before converging on the final parameters?

-Page 33: The authors note “The best scoring member of the cluster was chosen as a representative, and 1000 compounds from each library were visually inspected to select compounds for synthesis and experimental evaluation.” The authors should explain this step in more detail. A reader will be curious how much of the visual inspection impacted the results. What were the key factors? Which kind of people were making the selections (chemists, modelers, biologists, etc)? Furthermore, could the visual inspection decisions be automated? Any thoughts about training an ML model based on the human selections? More details would be greatly appreciated. As it stands, the work is quite unreproducible.

Minor:
-Page 33: The authors noted “500 steps of rigid-body minimization”. What was the purpose of this step? It appears the scores were obtained before this step. Furthermore, why rigid-body minimization?
The name “ANCHOR” does not appear in the results, only in the Methods. It would be nice to mention it before the Methods. Also, a recommendation from the reviewer is to call the library “AANCHOR” because it will be easier to find in searches and more is a fuller representation of the acronym.

-Page 9, last sentence: Adverbs like “remarkably” are superfluous for a scientific publication like this. Let the data speak for itself.

-Figure 4: It would be nice to show the ligand RMSD between the predicted models and x-ray structures for 28a and 30a.

-Page 36 states the size of the Enamine REAL library is 39B compounds, whereas the Enamine website lists a downloadable database of 9.6B compounds. The authors should clarify which collection is referenced. Is it possible that the 39B compounds represent an expanded set of the 9.6B, with tautomers, stereoisomers, etc?

Typos and Grammar:
-“The RDKit” is mentioned several times. In some cases it is followed by the URL and in other cases it is not. Probably only the first instance needs to include the URL.

-Page 55: For PDB codes, the authors write “9QC1 (compound 28a) and 9QD (compound 30a)”. It looks like the PDB code for compounds 30a is missing a character.

-Page 31 and Page 32: The sizes of the libraries, 8226610 and 6345793, should include commas for improved readability (e.g., 8,226,610 and 6,345,793).

Reviewer #2

(Remarks to the Author)
Comments on NCOMMS-25-28689
The authors reported some serendipity results on virtual screening, hit identification and optimization, mechanism of action study, and structural biology results on NTS1R agonists ending up with some NTS2R agonists as potential analgesics. While the study design seemed reasonable and the results seemed reliable, the overall integrity of the research project is missing. Therefore, it is not recommended for publishing.
First, there is no literature summary and comparison regarding both NTS1R and NTS2R agonists regarding their sequence homology analysis, functional properties, current research status, potential as novel analgesics, etc.
Second, while the majority of the studies were focused on NTS1R (e.g. virtual screening, hit identification and optimization, mechanism of action, and structural biology), merely some binding affinity data to support the end point of the discussion on NTS2R.
Third, the chemical entities identified as hits/leads are “me-too” compounds of known ligands SRI-9829 and RTI-3a. The basic structural skeletons are very similar among all of them.
Fourth, the in vivo analgesic study included only one animal model. The administration route is not suitable for future development of the hits.

Reviewer #3

(Remarks to the Author)
I co-reviewed this manuscript with one of the reviewers who provided the listed reports. This is part of the Nature Communications initiative to facilitate training in peer review and to provide appropriate recognition for Early Career Researchers who co-review manuscripts.

Reviewer #4

(Remarks to the Author)
the authors report on identification of novel chemical probes for NTS1 and NTS2 receptors, three of which capable to exert antinociceptive activity in a pain model with the reasons for efficacy not entirely clear. Crystal structures for two selected NTS1 ligands are also disclosed including a proposal for their mechanism of activation. This is a tour de force towards development of novel NTSR tools with interesting biological data and the surprising identification of NTS2R ligands. The manuscript is well written, easy to read, has very few flaws, the figures are very well crafted and highly appealing; yet this nice piece of work requires enhanced semantic precision in several instances and some additional experimentation to make sure that the claims are entirely supported by the experimental data.

Specific comments:

Abstract:
1. "safer pain medications". Safer than what? what is your comparator? Please consider to rephrase or add the less safe type of pain medications you have in mind.

Results:
2. Fig1c: I am not sure whether it is fair to refer to compound 25 as full agonist here. It clearly shows less efficacy as 24 and a bell-shaped concentration effect behavior. To me this looks like a molecule with a lower max effect due to strong desensitization which I think the authors discover later. I also think that the strong desensitization is the reason why the max is not reached. If the authors tested these ligands head to head in arrestin KO cells, efficacy may be the same.
3. sentence related to Fig1d, please re-read careful and check for correctness. To me the second part of the sentence does not fit to the first part.
4. Fig3, biological data related to compound 35a: please complete the Gq activation data in panel d, particularly if the incomplete curve may hide (or not) a bell shape as the reader wants to know and as it is mechanistically relevant.
5."To assess the pharmacological profiles of the newly designed ligands, we evaluated the ability of three compounds (28a, 30a, and 35a; Figure 3a) and reference agonists (RTI-3a and SRI-9829) to activate NTS1R-related signaling (Table 1)". Please rephrase to signaling and arr recruitment as you also measure arrestin recruitment; arr recruitment is no measure for signaling. Please also adapt the heading of table 1. It reads "Table 1. Functional activities of the target compounds for the different signaling pathways activated by hNTS1R in HEK293 cells. " arr recruitment is no signaling pathway, correct the heading to signaling pathways and arr recruitment, arrs can't be treated like signaling molecules as they don't signal.
6. "Moreover, NTS1R is known to tightly interact closely with β-arrestins that mediate receptor desensitization, which are involved in receptor desensitization, play an important role in receptor internalization, and may be involved in G protein-independent signaling
(Figure 3b). " Please check the sentence and remove redundancies, please also delete G protein-independent signaling as there is no literature support for such a statement, just unsupported claims. Replace this term with the known and established arrestin functions: arrestins desensitize and internalize and scaffold signaling processes, they don't signal on their own.
7. Moreover, you measure arr recruitment in the presence of GRK2 which is G protein-dependent, so you are setting up your assay to enhance G protein-dependent phosphorylation and arr recruitment which you notice as enhanced ligand potencies after GRK2 over-expression. Particularly if you refer to arrestin bias of ligands but make the arr recruitment G protein-dependent (GRK2 enrichment), this is both not meaningful and also confusing. Please reconsider this very careful and be more semantically precise. Look at Matthees et al for more information and conceptual help, doi: 10.1038/s42003-024-06490-1.
8."Compared to signaling via the primary coupling partner Gq, the potency of NTS8–13 was 2.5-fold lower for the activation of Gi1 (EC50 = 1.6 nM) and 22-fold lower (EC50 = 14 nM) for the Gs-mediated activation of adenylyl cyclase". Please rephrase carefully as you can't compare EC50 values for one ligand in different assays, EC50 values totally depend on the chosen assay. If you use different methods for cAMP detection, you may also produce differing EC50 values.
9."The effects of compounds 30a and 35a were weaker, although compound 35a proved to be the most potent small molecule under these conditions (EC50 = 2.7 µM). " most potent sounds "dramatic" but the data in Fig3i rather indicate that the ligands are very similar without any significant difference. I would rephrase to not imply a dramatic effect while the figures shows curves that are all on top of each other.
10."Despite the comparable potencies in IP accumulation, the agonists hence showed different abilities to influence the binding of the allosteric modulator." why do you choose the potencies in IP accumulation assays as comparator while the efficacies are clearly distinct indicating that RTI is different because it is just a partial agonist, too partial to have an effect on the intracellular ligand. I think the profile of the ligands in Fig3i makes perfect sense and the term "despite" is not necessary.
11. "As the backbone of residues 333–350 in the compound 28a and 30a complexes aligns much more closely with the NTS8–13 complex, it is possible that this difference is correlated with the RTI-3a complex is responsible for the increased efficacy of compounds 28a and 30a, and that this mirrors the inclination of helix 6 and 7 responsible for access of the C-terminal helix of Gα. " please check and correct the sentence, it is not really clear.
12. why do the newly identified compounds produce analgesia? Because of their particular signaling fingerprints on NTS1 and/or because of their efficacy on NTS2? This is important for developing new painkillers. without this knowledge, it is unclear what to optimize, a particular signaling profile or just action on NTS2 or both.

Discussion.
13. "Second, structure-guided optimization led to the discovery of agonists with signaling signatures distinct from the natural peptide. " please comment and explain the relevance of the distinct signaling signatures. I appreciate the signatures but the relevance of the profile remains unclear. If it is unclear also from the authors point of view, this should be stated. Successful drugs for GPCRs may not need particular signaling fingerprints as most successful drugs were found when nothing was

known about signaling fingerprints.
14. "Crystal structures of the receptor-agonist complexes confirmed the computational models and helped rationalize the signaling profiles. " is this true? did the complexes help rationalize the signaling fingerprints or the ligand efficacies? please clarify.
15. "Finally, low nanomolar NTS2R selective compounds produced analgesia in a rodent model, providing starting points for the development of novel drugs to treat pain. " is this claim supported by the findings? Is analgesia mediated via NTS2 and/or NTS1 or both? The compounds prefer NTS2 over NTS1 but the reason for their in vivo efficacy is not clear based on the presented data. For example, absence of the analgesic effect in a NTS2KO model or maintenance of the analgesic effect in a NTS1KO model needs to be demonstrated. Some genetic evidence is required to claim analgesic action of the compounds in vivo through NTS2. at present the provided experimental evidence is correlative but needs to be causal to make a strong claim. Along the same lines, any future drug discovery effort needs to know what exactly to optimize, NTS2 action over NTS1 or a particular NTS1 signaling profile.
16. "The small-molecule agonists essentially lack interactions mimicking those of Arg8, Arg9, and Pro10, likely preventing the stabilization of high agonist-affinity states in wild-type NTS1R. These observations provide directions for further optimization of small-molecule NTS1R agonists." please comment whether optimized ligands for NTS1 which mimic the interactions of Arg8, Arg9, and Pro10 can still be considered small molecules or will need to be substantially bigger.
17. "Our study shows that it is also possible to design orthosteric agonists with distinct signaling signatures, and compound 35a biased NTS1R signaling toward β-arrestin-2". please rephrase as you have no data showing that you bias NTS1R signaling towards arrestin. arrestins don't signal and the assay you use is a protein-protein interaction assay. Make sure you are semantically precise or, alternatively, use a true arrestin signaling assay for your compounds. Keep in mind that you measure arrestin recruitment in the presence of a G protein-dependent kinase, enhancing G protein-dependence of arrestin recruitment.
18. "This scaffold provides a starting point for development of a portfolio of biased NTS1R agonists, which could be used to identify the optimal profiles for different therapeutic areas. " please explain the rationale for developing biased NTS1R agonists as all three compounds with distinct signaling signatures have the same in vivo effect most likely originating from NTS2 activation. I would suggest to not claim that biased ligands will have any value as there is no evidence for it. The data rather suggest the opposite, ie that the signaling profile is irrelevant for the in vivo efficacy. I also suggest to delete speculations about bias profiles and different therapeutic areas as the idea of bias in general has not really proven to be successful in the clinic.
19. "Compound 35a showed exceptionally high affinity for NTS2R and is the first small molecule of this target to show an analgesic effect in vivo." this is very exciting data; with causal evidence that 35a acts through NTS2 but not NTS1 using the respective KO animals, this will be a strong paper.

Reviewer #5

(Remarks to the Author)
The manuscript by Panel at al. reports the identification and evaluation of novel NTSR ligands from peptide structure-based small molecule libraries using virtual and cell-based screening approaches and show. Three of the identified ligands have µM affinities for NTS1R and nM affinity for the NTS2R, with compound 35a showing highest affinity for both NTSRs. The best ligands came from a focused library of compounds containing the structure of the C-terminal leucine in NTS. Co-crystalization with stabilized active NTS1R is performed and modeling-based structural properties affecting affinity, efficacy and selectivity are examined and discussed. The molecular pharmacological evaluation is quite comprehensive, including multi-pathway signaling analysis at NTS1R, binding affinities at NTS1 and 2Rs, stimulation of NTS1R PAM binding, and off-target binding assessment at some other peptide-binding GPCRs. In vivo, the three top novel compounds are found to produce antinociception in a rat formalin-evoked pain model. The study is generally well done and reports novel information. However, some key questions remain unaddressed and the rational for focusing on NTS1R instead of NTSR2 is unclear. These concerns and several minor concerns are listed below.

1. The identified ligands were found to have ~43-360-fold greater affinity for NTS2R than NTS1R, but all the signaling pathway studies were done only with the NTS1R. It would provide more insight into the utility of these ligands as NTS2R agonist analgesics if signaling pathways at NTS2R assessed with these ligands, at minimum for the major (Gq/11) pathway. Findings from these studies would support or oppose the speculation, based on relative binding affinities, that the antinociceptive effects of these ligands are due to NTS2R activation in vivo.

2. In a related concern, the rationale for focusing on NTS1R in the ligand discovery process was not entirely clear, given that the authors predict the NTS2R would have fewer side effects as a potential analgesic target. The preexisting ligands in Table 2 all appear to have moderate to high affinity for NTS2R. Perhaps this choice was based purely on the availability of structural information on NTS1R, but this is not explicitly stated.

3. The authors mention multiple times that small molecule compounds are more suitable to cross the blood brain barrier and are more likely to be bioavailable than peptides. However, the authors used an intrathecal route of administration in their behavioral assay, which doesn’t account for either of these things. At minimum, a rationale for using the intrathecal route should be provided.

4. A related concern is that physiochemical and ADME properties of the top ligands are not presented. For example, there are computational tools for determining the Lipinski criteria, etc. that could have been applied.

5. Minor points:

a. The connection between beta-arrestin2 and desensitization is mentioned twice in the same sentence on p. 11, paragraph 1. “Moreover NTS1R is known to tightly interact closely with β-arrestins that mediate receptor desensitization, which are involved in receptor desensitization, play an important role…”

b. In the “Analgesic activity of selective high-affinity ligands of NTS2R” section: The sentence referring to the results showing compound affinity for rNTS2R in radioligand binding assays (Supplemental Table S8) may be better discussed in the prior section with the results describing affinity at hNTS2R and selectivity of the compounds.

c. In paragraph 1 on p. 20: the phrase: “…it is possible that this difference is correlated with the RTI-3a complex is responsible…” is awkwardly worded. Perhaps it should read: “…'and' is responsible…”?

d. In the radioligand methods descriptions on p. 43-44: Perkin-Elmer radiochemical division is now called Revvity (at least in the U.S.).

Reviewer #6

(Remarks to the Author)
I co-reviewed this manuscript with one of the reviewers who provided the listed reports. This is part of the Nature Communications initiative to facilitate training in peer review and to provide appropriate recognition for Early Career Researchers who co-review manuscripts.

Reviewer #7

(Remarks to the Author)
The manuscript "Docking of virtual libraries identifies small-molecule agonists of neurotensin receptors with analgesic activity" by Panel, N et al. is a well written manuscript and a well concieved study with great promise to further the field for not only the neurotensin receptors but also other peptide GPCRs. The authors developed a peptide GPCR focused docking library, performed docking studies with this focused library on NTSR1, identified several good affinity compounds and determined the crystal structure of NTSR1 in complex with these compounds.

With this, there are several areas of the manuscript that could be expanded or elaborated upon to improve the manuscript for the reader (below).

1) The rationale for optimization of chemical compounds is not very clear, could more information be added to help the reader understand why modifications were made, ie. docking indicated that there was areas within the binding pocket to expand, or maybe modifications were made to interact with additional key residues such as ...

2) When testing compounds against different signaling pathways, bias calculations should be included. Additionally, the authors elude to interactions with potential key residues for bias, mutagenesis studies along with MD would help to support their claim and further expand research to identify orthosteric binding pocket biased ligands.

3) When discussing the the peptide receptor focused virtual library, it would be great to have a comparison with a known library. Does it yield better results, what is the hit rate, etc..

4) When comparing different ligands with SBI-553, it would be great to have the allosteric parameters along with comparison of curves. The probe effect could be related to the compound bias.

5) Much discussion in the field regarding neurotensin receptors involved in pain response is related to whether the pain signal is commented at the neuron level or at the site of injury. PK studied of the new NTSR2 compound which show antinociceptive properties to indicate if it is peripherally restricted or passes the blood brain barrier would a good along way in helper other researchers.

Version 1:

Reviewer comments:

Reviewer #1

(Remarks to the Author)
The authors have addressed my concerns and the paper is much stronger now.

Reviewer #2

(Remarks to the Author)
The revised manuscript has responded to my critics point-by-point. Though some arguments are not very strong, their effort should be recognized. Therefore, I would like the editor and other reviewers to decide if the revised manuscript is suitable for further consideration.

Reviewer #3

(Remarks to the Author)
I co-reviewed this manuscript with one of the reviewers who provided the listed reports. This is part of the Nature Communications initiative to facilitate training in peer review and to provide appropriate recognition for Early Career Researchers who co-review manuscripts.

Reviewer #4

(Remarks to the Author)
I thank the authors for their detailed responses and the considerable effort invested in revising the manuscript. I am largely satisfied with the revision; however, I believe that, now that it is clear that the analgesic activity is mediated through NTSR2, the abstract should place less emphasis on NTSR1 and on signaling fingerprints that are not relevant to the in vivo effects. The current data suggest that, although the initial goal was to identify NTSR1-targeted compounds, the observed robust in vivo effects resulted instead from activity at NTSR2. This point should be communicated more clearly in the abstract. Even if these findings arose partly through serendipity, the abstract should reflect this outcome with greater transparency and precision.

Reviewer #5

(Remarks to the Author)
The authors responded to all reviews with thoughtful revisions and my concerns have been sufficiently addressed. I congratulate the authors on a significant and interesting manuscript.

Reviewer #6

(Remarks to the Author)
I co-reviewed this manuscript with one of the reviewers who provided the listed reports. This is part of the Nature Communications initiative to facilitate training in peer review and to provide appropriate recognition for Early Career Researchers who co-review manuscripts.

Reviewer #7

(Remarks to the Author)
I want to thank the authors for incorporating not only my comments but the other reviewers' comments into their manuscript "Docking of virtual libraries identifies small-molecule agonists of neurotensin receptors with analgesic activity." The authors' have responded exceptionally well and addressed all concerns to my satisfaction and have improved an already great manuscript providing clarity where needed. Furthermore, It is my pleasure to recommend this manuscript for acceptance in the journal Nature Communications.

Open Access This Peer Review File is licensed under a Creative Commons Attribution 4.0 International License, which permits use, sharing, adaptation, distribution and reproduction in any medium or format, as long as you give appropriate credit to the original author(s) and the source, provide a link to the Creative Commons license, and indicate if changes were made.
In cases where reviewers are anonymous, credit should be given to 'Anonymous Referee' and the source.
The images or other third party material in this Peer Review File are included in the article's Creative Commons license, unless indicated otherwise in a credit line to the material. If material is not included in the article's Creative Commons license and your intended use is not permitted by statutory regulation or exceeds the permitted use, you will need to obtain permission directly from the copyright holder.
To view a copy of this license, visit https://creativecommons.org/licenses/by/4.0/

March 6th, 2026

We want to thank the referees for taking the time to review our manuscript (*"Docking of virtual libraries identifies small-molecule agonists of neurotensin receptors with analgesic activity"*, Manuscript: NCOMMS-25-28689) and the many constructive suggestions. We have carefully considered all comments and performed additional calculations as well as new *in vitro* and *in vivo* experiments, resulting in a substantially stronger manuscript. Please find below point-by-point responses to the referees with their comments in ***Blue***. Relevant changes to the manuscript have been marked in yellow to facilitate the review process.

Reviewer #1

The first reviewer wrote that our study ***"underscores the utility of tailored virtual libraries for discovering ligands for peptide-binding GPCRs and offers promising lead compounds for further development of non-opioid pain therapeutics"***. The reviewer requested a more detailed description of our methodology and additional information regarding the hit optimization step. As outlined below, we have addressed these points along with all minor comments:

Major comments:

1. Reviewer: ***The authors should provide additional information about the optimization procedure. Details can be left for the Methods section, but a bit more info in the Results section would be helpful for the reader. For example, how many analogs of 24 and 25 were generated? How was the selection performed? What were the driving criteria for selection? Was the procedure identical to the original library screening selection or were learnings from the initial screening applied to the optimized compounds? Is this a reproducible approach? Could the selection criteria be encapsulated in an algorithm? If not, could machine learning based on the selections be used to infer the next selections?***

We thank the reviewer for this suggestion and have added additional information regarding the optimization step in the revised manuscript. We included the number of synthesized analogs of compounds **24** and **25**, and provide a more detailed description of the analog selection process. Compared to the original library screening selection, we typically make smaller, more systematic changes during the hit optimization step to elucidate structure-activity relationships. In the first analog selection step, we identified compounds that extended into the TM2-TM7 pocket occupied by **RTI-3a**, *e.g.*, by forming interactions with $H343^{7.32}$ and $Y346^{7.35}$. In a second step, we focused particularly on identifying substituents mimicking the interactions of the Tyr_{11} side chain of $NTS_{8\text{-}13}$ (*e.g.*, interactions with with $H131^{ECL1}$, $T225^{ECL2}$, and $V223^{ECL2}$), which has

been shown to be essential for high-affinity peptide binding (DOI: 10.1021/acs.jmedchem.0c01726). In the revised manuscript, we have added more details regarding the optimization on pages 9-10.

Several of the above steps could be made more efficient by utilizing machine-learning tools. For example, machine-learning methods could have provided alternative synthetic routes to obtain our compounds, additional reactions yielding more diverse substitutions, or been used to predict pharmacokinetic properties. However, because the available bioactivity data in this project, and for the target in general, are relatively sparse and show limited variation, machine-learning-based models of compound potency or efficacy would likely be of limited value in the optimization step. In this case, we would instead consider using higher-level physics-based methods, such as molecular dynamics simulations, to guide compound selection. In the revised manuscript, we have added the reviewer's suggestion to use machine-learning tools in the discussion (pages 28-29).

2. Reviewer: ***It looks like the modeled conformation of the thiophene is perpendicular to the attached phenyl. It is important to highlight issues like this, especially for a broad audience like Nature Communications. Many readers will not have the technical background to assess the models, so the authors would do a great service by elaborating on some of the model deficiencies. Furthermore, the authors could discuss the model assumptions and potential areas for improvement. For example, was the thiophene perpendicular in the original Dock pose? If so, was that due to the initial conformation generation? Could it have been fixed with an unrestrained minimization as opposed to the rigid-body minimization that the authors described? If not, a QM minimization would likely fix this. The reviewer is not proposing that the authors do any additional calculations, but they should highlight the model errors and potential solutions. This will help ground the paper in reality and not be as subjected to overhype.***

We agree with the reviewer that identifying weaknesses of the computational models is important, as this can reveal how to improve predictions. As the reviewer noticed, the torsional angle between the thiophene and phenyl is almost perpendicular in our computational model. Assessment of the conformation of a representative ligand (compound **28a**) from our docking calculations using an online tool (https://torsions.zbh.uni-hamburg.de) clearly showed that this is a strained torsion angle. The strained conformation was generated by the program OMEGA (https://www.eyesopen.com/omega), which is used by the docking program for conformer generation. We agree that minimization using quantum mechanics or higher-accuracy molecular mechanics force fields would have generated a planar structure, but such techniques are computationally expensive. Whereas it is not feasible to use such methods to predict ligand conformations in screens of large libraries, this would actually be feasible to perform in the hit optimization phase (*e.g.*, molecular dynamics simulations). In the revised manuscript, we mention the weaknesses of our docking model and discuss potential solutions (pages 18 and 28-29).

3. Reviewer: ***The authors trained docking parameters based on known active and inactive compounds for NTSR. That point should be made clearly earlier in the manuscript. Reading the results, there is no mention of custom fitting based on existing data. This makes a big difference for people trying to assess the applicability of such a protocol for their target(s) of interest. Furthermore, the authors should explain in more detail which parameters were adjusted during this retrospective optimization exercise. Was there a systematic exploration of parameters? Which parameters? How many enrichment trials were run before converging on the final parameters?***

We thank the reviewer for pointing out that we did not describe the preparation of the molecular docking calculations in sufficient detail. We use the protocols described by Bender *et al.* (DOI: 10.1038/s41596-021-00597-z) to prepare docking parameters, an approach that is common practice among users of the program DOCK. The DOCK binding energy score is calculated from grids describing electrostatic, van der Waals, and ligand desolvation energies. In the calculation of electrostatic and desolvation energy grids, the boundary between low dielectric regions (occupied protein and ligand) and high dielectric regions (occupied by water) of the binding site is determined by a layer of spheres placed on the protein surface. Before performing the prospective docking screen, we optimized the enrichment of known ligands over decoys by evaluating different sphere radii. In the revised manuscript, we describe the optimization of docking parameters in the methods section (pages 6 and 36-37).

4. Reviewer: ***The authors note "The best scoring member of the cluster was chosen as a representative, and 1000 compounds from each library were visually inspected to select compounds for synthesis and experimental evaluation." The authors should explain this step in more detail. A reader will be curious how much of the visual inspection impacted the results. What were the key factors? Which kind of people were making the selections (chemists, modelers, biologists, etc)? Furthermore, could the visual inspection decisions be automated? Any thoughts about training an ML model based on the human selections? More details would be greatly appreciated. As it stands, the work is quite unreproducible.***

We thank the reviewer for these suggestions. As outlined below, the steps for performing the structure-based virtual screen and compound selection follow best practices in the field, as described in previous publications (DOI: 10.1038/s41596-021-00597-z, 10.1124/pharmrev.120.000246). In the revised manuscript, we describe the compound selection step in more detail and provide relevant references to enable others to reproduce our approach for other targets.

Compound selection from prospective docking screens is typically performed in two steps: **(1)** molecular docking of the library and **(2)** visual inspection of top-ranked compounds. In our study, the docking calculations reduced the number of compounds considered for experimental evaluation by over 99.9% (from 6–8 million to only 1,000 molecules). In the second step, visual inspection of predicted complexes is standard practice. For example, the protocol suggested by Bender et al. (DOI: 10.1038/s41596-021-00597-z) recommends inspecting 5,000 compounds – five times more than we considered in our screens. During this step, the team of computational and medicinal chemists performing the virtual screen compensates for limitations of the docking

scoring function. A published survey indicates that the research field applies similar criteria at this stage (DOI: 10.1021/acs.jmedchem.0c02227).

In the selection step, we first focus on identifying compounds to exclude from further consideration. The key factors in compound selection are (See Table 2 in DOI: 10.1038/s41596-021-00597-z):

• **Unsatisfied polar groups of the ligand**
Hydrogen bond donors or acceptors of the ligand are sometimes predicted to interact with nonpolar pockets in the binding site, likely reflecting an underestimation of the ligand desolvation penalty by the scoring function. Such compounds are common and can be readily identified through visual inspection.

• **Ligand strain**
Because ligand strain is not explicitly accounted for in the scoring function, top-ranked compounds can adopt high-energy conformations, which is a key reason for excluding certain compounds from experimental evaluation. Assessing this contribution visually can be challenging, so to improve reproducibility, we used online servers (https://torsions.zbh.uni-hamburg.de/) to evaluate ligand strain during the assessment.

• **Tautomer and ionization state of the ligand**
Compounds with multiple possible tautomeric or ionization states are carefully analyzed using several prediction programs to identify the most probable form. Top-ranked compounds docked in a tautomeric or ionization state considered unlikely are deprioritized during selection.

• **Binding site desolvation**
The scoring function does not account for desolvation of binding site residues, so nonpolar ligand groups are sometimes positioned near polar residues. This is a common reason for excluding compounds during visual inspection.

After applying the above exclusion criteria, we evaluate the overall shape complementarity of the complex. Compounds that form key non-polar and polar interactions (*e.g.*, protein-ligand hydrogen bonds observed in experimental structures) are prioritized for selection. In a final step, we inspect the compounds that fulfil all of these criteria and select a subset that forms diverse interactions with the binding site.

As we have published a detailed description of all the above steps in *Nature Protocols* (DOI: 10.1038/s41596-021-00597-z) and *Pharmacological Reviews* (GPCR specific protocol, DOI: 10.1124/pharmrev.120.000246), we have added a brief summary of the selection criteria in the results section (page 8). The reviewer's suggestion to further automate compound selection using machine learning is very interesting. A recent paper (DOI: 10.1021/acs.jcim.5c00850) suggests that such an approach can increase the efficiency of this labor-intensive step and also increase screening hit rates. We cite this work in the discussion section of the manuscript (pages 28-29).

Minor comments:

1. Reviewer: ***The authors noted "500 steps of rigid-body minimization". What was the purpose of this step? It appears the scores were obtained before this step. Furthermore, why rigid-body minimization?***

We thank the reviewer for pointing out that this methodological detail was not clear. In the docking program used in this study (DOCK3.7), hundreds of compound conformations are pre-calculated. These conformations are then docked as an ensemble into the binding site using an efficient sampling algorithm (DOI: 10.1371/journal.pone.0075992). To further optimize the best scoring conformation, a rigid-body minimization in the binding site is then performed. As the conformations are pre-calculated, no minimization of the ligand can be performed at this stage. The final score after the rigid-body minimization step is used in the ranking of the chemical library. We have added a more detailed description of the sampling and scoring in the methods section (pages 37-38).

2. Reviewer: ***The name "ANCHOR" does not appear in the results, only in the Methods. It would be nice to mention it before the Methods. Also, a recommendation from the reviewer is to call the library "AANCHOR" because it will be easier to find in searches and more is a fuller representation of the acronym.***

We thank the reviewer for this excellent suggestion. The name of our library has been changed to AANCHOR, and we the naming was introduced in the results section of the manuscript (page 27).

3. Reviewer: ***Adverbs like "remarkably" are superfluous for a scientific publication like this. Let the data speak for itself.***

We agree with the reviewer and have modified the manuscript text (pages 10 and 19).

4. Reviewer: ***It would be nice to show the ligand RMSD between the predicted models and x-ray structures for 28a and 30a.***

As suggested by the reviewer, we have added ligand RMSD values on page 18 of the revised manuscript.

5. Reviewer: ***Page 36 states the size of the Enamine REAL library is 39B compounds, whereas the Enamine website lists a downloadable database of 9.6B compounds. The authors should clarify which collection is referenced. Is it possible that the 39B compounds represent an expanded set of the 9.6B, with tautomers, stereoisomers, etc?***

Enamine provides several compound collections (https://enamine.net/compound-collections/real-compounds). In this study, we have used the *"REAL space"* (https://enamine.net/compound-collections/real-compounds/real-space-navigator), which contained 39 billion compounds at the time of the study (currently: 76.9 billion). The reviewer is likely referring to the *"REAL database"* (https://enamine.net/compound-collections/real-compounds/real-database), a library

that contains 10.1 billion compounds today. All of these collections contain unique compounds, *i.e.* multiple tautomers for the same molecule are not part of the database. We have included a reference to the REAL Space website in the revised manuscript.

6. Reviewer: ***"The RDKit" is mentioned several times. In some cases it is followed by the URL and in other cases it is not. Probably only the first instance needs to include the URL.***

We thank the reviewer for noting this inconsistency and have moved the URL to the references.

7. Reviewer: ***Page 55: For PDB codes, the authors write "9QC1 (compound 28a) and 9QD (compound 30a)". It looks like the PDB code for compounds 30a is missing a character.***

We thank the reviewer for noticing this error and we have corrected the PDB accession code to 9QD4 (page 66).

8. Reviewer: ***Page 31 and Page 32: The sizes of the libraries, 8226610 and 6345793, should include commas for improved readability (e.g., 8,226,610 and 6,345,793).***

We have added the suggested commas in the revised manuscript.

Reviewer #2

The second reviewer had several suggestions on how to improve our study. In the revised manuscript, we provide additional information regarding the study design, demonstrate the novelty of our agonist scaffold, and have performed additional *in vivo* and *in vitro* characterization of our compounds. We respond to the reviewer's questions below:

Major comments:

1. Reviewer: ***First, there is no literature summary and comparison regarding both NTS1R and NTS2R agonists regarding their sequence homology analysis, functional properties, current research status, potential as novel analgesics, etc.***

We thank the reviewer for this suggestion and have added a summary of sequence homology and relevance for development of novel analgesics in the revised manuscript (pages 3-4).

2. Reviewer: ***Second, while the majority of the studies were focused on NTS1R (e.g. virtual screening, hit identification and optimization, mechanism of action, and structural biology), merely some binding affinity data to support the end point of the discussion on NTS2R.***

The virtual screen, hit optimization, and structural biology efforts were focused on the NTS_1R receptor because structure determination of NTS_2R has proven very challenging. Currently, there are no available structures of NTS_2R. However, as the binding sites of the two receptors are highly similar, we anticipated that compounds

designed based on the NTS_1R structure would be relevant for both subtypes. This is further supported by that **RTI-3a**, which is bound in the structure used to design the ligands, binds to both subtypes. We have added these clarifications to the introduction and results of the revised manuscript (pages 3-4 and 5). To complement the binding assays for NTS_2R, we have also added new *in vitro* and *in vivo* experiments demonstrating that the key compounds (**28a**, **30a**, and **35a**) activate NTS_2R (pages 21-22), and that their analgesic effects are mediated by this subtype (pages 23-24).

3. Reviewer: ***Third, the chemical entities identified as hits/leads are "me-too" compounds of known ligands SRI-9829 and RTI-3a. The basic structural skeletons are very similar among all of them.***

In the virtual screening research field, the similarity of designed compounds to known ligands is typically quantified using Tanimoto similarity and chemical fingerprints. Recently published protocols (DOI: 10.1038/s41596-021-00597-z) for virtual screening use a Tanimoto similarity coefficient (T_c) of 0.35 as a threshold for novelty (ECPF4-based fingerprints). Accordingly, we quantified the chemical similarity of compounds **28a**, **30a**, and **35a** from our study and the ligands mentioned by the reviewer. The maximal T_c values were 0.29, 0.31, and 0.16 for compounds **28a**, **30a**, and **35a**, respectively. Based on the best practices of the field, these compounds would hence be considered structurally distinct from these reference ligands. We have added this result to page 10 of the revised manuscript and Supplementary Table 5. As the reviewer noted, the feature that **SRI-9829** and **RTI-3a** share with the designed agonists is the C-terminal leucine. This was part of the design principle of the focused chemical library, an approach that substantially increased the virtual screening hit rates. However, we agree that this choice could reduce the novelty of the hits from this library. This strategy is therefore most suitable for challenging targets expected to yield low virtual screening hit rates, including many peptide- and protein-binding GPCRs. We have added these conclusions to the discussion of the revised manuscript (page 28).

4. Reviewer: ***Fourth, the in vivo analgesic study included only one animal model. The administration route is not suitable for future development of the hits.***

As requested by the reviewer, we assessed the analgesic efficacy of compound **28a** in a second pain model, the acute tail-flick assay, in addition to the tonic formalin pain test. As shown in the new Figures **6e** and **6f**, compound **28a** produced robust analgesic effects, evidenced by a significant increase in tail-flick latency. These results have been added on pages 23-24 of the revised manuscript. We also agree with the reviewer that intrathecal delivery is not the most practical or widely applicable route for pain management. However, we respectfully wish to point out that several analgesic agents, including certain opioids, α_2 adrenergic agonists, and ziconotide (a macrocyclic peptide that inhibits calcium channels involved in pain signaling) are administered intrathecally in clinical practice for some refractory pain conditions, such as severe cancer-related pain. We have added in the discussion that further optimization of the pharmacokinetic properties of the compounds will be beneficial (page 31).

Reviewer #3

Reviewer #3 co-reviewed this manuscript with one of the reviewers who provided the listed reports.

Reviewer #4

The fourth reviewer was also enthusiastic about our work and described our manuscript as ***"a tour de force towards development of novel NTSR tools"***, ***"nice piece of work"***, and ***"well written"***. The reviewer only had minor comments to improve the manuscript, which we address below:

Minor comments:

1. Reviewer: ***"safer pain medications". Safer than what? what is your comparator? Please consider to rephrase or add the less safe type of pain medications you have in mind.***

We thank the reviewer for highlighting that the motivation for developing novel pain medications was unclear. While opioids are widely used for pain management, they are associated with numerous side effects, including constipation, tolerance, and respiratory depression. Moreover, misuse of these medications can lead to addiction and overdose. Neurotensin receptor agonists produce analgesic effects independent of the opioid system and are therefore a promising alternative target for the development of novel drugs to treat both acute and chronic pain. In the revised manuscript, we have added drawbacks of opioid analgesics in the introduction (page 4), modified the abstract (page 2), and made the comparison to opioids clearer in the discussion (page 32).

2. Reviewer: ***Fig1c: I am not sure whether it is fair to refer to compound 25 as full agonist here. It clearly shows less efficacy as 24 and a bell-shaped concentration effect behavior. To me this looks like a molecule with a lower max effect due to strong desensitization which I think the authors discover later. I also think that the strong desensitization is the reason why the max is not reached. If the authors tested these ligands head to head in arrestin KO cells, efficacy may be the same.***

We agree with the reviewer that the efficacy of compound **25** rather reflects partial agonism. We did not perform experiments in arrestin KO cells, as compounds **24** and **25** were initial screening hits and small differences in efficacy were not considered relevant at this stage of ligand optimization. In the revised manuscript, we now refer to these ligands as *"high efficacy"* agonists (page 8).

3. Reviewer: ***Sentence related to Fig1d, please re-read careful and check for correctness. To me the second part of the sentence does not fit to the first part.***

We thank the reviewer for pointing out that this description was not clear. We have rewritten this section in the revised manuscript (pages 8-9).

4. Reviewer: ***Fig3, biological data related to compound 35a: please complete the Gq activation data in panel d, particularly if the incomplete curve may hide (or not) a bell shape as the reader wants to know and as it is mechanistically relevant.***

Following the reviewer's suggestion, we have performed additional experiments and found no signs of a bell-shaped concentration-response profile. In the revised manuscript, we have updated Figure 3d, its legend, the respective entry for compound **35a** in Table 1, and the text on page 12.

5. Reviewer: ***"To assess the pharmacological profiles of the newly designed ligands, we evaluated the ability of three compounds (28a, 30a, and 35a; Figure 3a) and reference agonists (RTI-3a and SRI-9829) to activate NTS1R-related signaling (Table 1)". Please rephrase to signaling and arr recruitment as you also measure arrestin recruitment; arr recruitment is no measure for signaling. Please also adapt the heading of table 1. It reads "Table 1. Functional activities of the target compounds for the different signaling pathways activated by hNTS1R in HEK293 cells. " arr recruitment is no signaling pathway, correct the heading to signaling pathways and arr recruitment, arrs can't be treated like signaling molecules as they don't signal.***

We agree with the reviewer and modified the text on page 12 and in the Table 1 header.

6. Reviewer: ***"Moreover, NTS1R is known to tightly interact closely with β-arrestins that mediate receptor desensitization, which are involved in receptor desensitization, play an important role in receptor internalization, and may be involved in G protein-independent signalling (Figure 3b)." Please check the sentence and remove redundancies, please also delete G protein-independent signaling as there is no literature support for such a statement, just unsupported claims. Replace this term with the known and established arrestin functions: arrestins desensitize and internalize and scaffold signaling processes, they don't signal on their own.***

Following this reviewer's suggestion, we have modified the sentence on page 12: *"Moreover, NTS_1R is known to tightly interact with β-arrestins that mediate receptor desensitization, play an important role in receptor internalization and scaffold signaling processes (Figure 3b)."*

7. Reviewer: ***Moreover, you measure arr recruitment in the presence of GRK2 which is G protein-dependent, so you are setting up your assay to enhance G protein-dependent phosphorylation and arr recruitment which you notice as enhanced ligand potencies after GRK2 over-expression. Particularly if you refer to arrestin bias of ligands but make the arr recruitment G protein-dependent (GRK2 enrichment), this is both not meaningful and also confusing. Please reconsider this very careful and be more semantically precise. Look at Matthees et al for more information and conceptual help, doi: 10.1038/s42003-024-06490-1.***

We thank the reviewer for drawing our attention to this aspect. In fact, NTS_1R is phosphorylated by at least GRK5 and GRK2 (DOI: 10.1021/acs.biochem.5b00285).

As already stated in the manuscript, we have measured β-arrestin-2 recruitment with (Figure 3h) and without additional expression of GRK2 (Figure S4d). We observed high efficacy under both conditions for ligands **28a**, **30a**, and **35a** (E_{max} of 89-93% with GRK2, and 98-100% without GRK2 co-expression), although potencies were slightly higher with GRK2 co-expression. For the peptide agonist NTS_{8-13}, potency was slightly higher without GRK2 co-expression, suggesting that the $G_{\beta\gamma}$-dependent GRK2 may have some beneficial effect for the small molecule agonists, but is not required for peptide-agonist induced arrestin recruitment. In the revised manuscript, we have modified the passage on page 14 to point out the $G_{\beta\gamma}$-dependence of GRK2-mediated phosphorylation. Furthermore, we modified the conclusion of the functional activity section to put less emphasis on the relatively small differences in potency (pages 14-15).

8. Reviewer: ***"Compared to signaling via the primary coupling partner Gq, the potency of NTS8–13 was 2.5-fold lower for the activation of Gi1 (EC50 = 1.6 nM) and 22-fold lower (EC50 = 14 nM) for the Gs-mediated activation of adenylyl cyclase". Please rephrase carefully as you can't compare EC50 values for one ligand in different assays, EC50 values totally depend on the chosen assay. If you use different methods for cAMP detection, you may also produce differing EC50 values.***

The reviewer is right, EC_{50} values inherently depend on the used assay system. It was not our intention to comment on the preferences of NTS_{8-13}, but to provide a reference for the different potencies observed with our newly designed ligands. Since this information is already summarized in Table 1, we removed the sentence in question.

9. Reviewer: ***"The effects of compounds 30a and 35a were weaker, although compound 35a proved to be the most potent small molecule under these conditions (EC50 = 2.7 µM)." most potent sounds "dramatic" but the data in Fig3i rather indicate that the ligands are very similar without any significant difference. I would rephrase to not imply a dramatic effect while the figures shows curves that are all on top of each other.***

We thank the reviewer for this comment and reworded the section (pages 14-15).

10. Reviewer: ***"Despite the comparable potencies in IP accumulation, the agonists hence showed different abilities to influence the binding of the allosteric modulator." Why do you choose the potencies in IP accumulation assays as comparator while the efficacies are clearly distinct indicating that RTI is different because it is just a partial agonist, too partial to have an effect on the intracellular ligand. I think the profile of the ligands in Fig3i makes perfect sense and the term "despite" is not necessary.***

We agree with the reviewer and removed the sentence in question.

11. Reviewer: ***"As the backbone of residues 333–350 in the compound 28a and 30a complexes aligns much more closely with the NTS8–13 complex, it is possible that this difference is correlated with the RTI-3a complex is responsible for the increased efficacy of compounds 28a and 30a, and that this mirrors the inclination of helix 6 and 7 responsible for access of the C-terminal helix of Gα." please check and correct the sentence, it is not really clear.***

We thank the reviewer for pointing out that this sentence was not clear. We have corrected the text in the revised manuscript (pages 20-21).

12. Reviewer: ***Why do the newly identified compounds produce analgesia? Because of their particular signaling fingerprints on NTS1 and/or because of their efficacy on NTS2? This is important for developing new painkillers. without this knowledge, it is unclear what to optimize, a particular signaling profile or just action on NTS2 or both.***

We thank the reviewer for this important point. We fully agree that, based on the current dataset, we cannot formally conclude whether the analgesic effects of the newly identified small molecules arise exclusively from NTS_2R activation, from NTS_1R signaling, or from a combined contribution of both receptors, despite their higher binding affinity for NTS_2R relative to NTS_1R. Indeed, neurotensin produces robust, opioid-independent analgesia through activation of both NTS_1 and NTS_2 receptors. As detailed below (see Point #15), we therefore employed a pharmacological approach using non-selective and NTS_2R-selective antagonists to discriminate the respective contributions of the NTS_1 and NTS_2 receptor subtypes. These new *in vivo* data demonstrate that NTS_2R plays a major role in mediating the analgesic effects of the newly identified compounds. These results have been added on pages 23-24.

13. Reviewer: ***"Second, structure-guided optimization led to the discovery of agonists with signaling signatures distinct from the natural peptide." Please comment and explain the relevance of the distinct signaling signatures. I appreciate the signatures but the relevance of the profile remains unclear. If it is unclear also from the authors point of view, this should be stated. Successful drugs for GPCRs may not need particular signaling fingerprints as most successful drugs were found when nothing was known about signaling fingerprints.***

Following this reviewer's suggestion, we have added a sentence to the discussion to address the knowledge gap between the desired pharmacological outcome and specific signaling profiles (page 32): *"However, clear connections between specific signaling signatures and the desired pharmacological outcomes in vivo remain to be established."*

14. Reviewer: ***"Crystal structures of the receptor-agonist complexes confirmed the computational models and helped rationalize the signaling profiles." Is this true? did the complexes help rationalize the signaling fingerprints or the ligand efficacies? please clarify.***

We thank the reviewer for this comment. In the revised manuscript, we replaced *"signalling profiles"* with *"structure-activity relationships"*. This is a better description of the role of the structures in the study and refers to comparisons of compounds **28a**, **30a**, and **RTI-3a**. The structures provide insights into the relative affinities and efficacies of these compounds, which is described on pages 19-21.

15. Reviewer: ***"Finally, low nanomolar NTS2R selective compounds produced analgesia in a rodent model, providing starting points for the development of novel drugs to treat pain." is this claim supported by the findings? Is analgesia mediated via NTS2 and/or NTS1 or both? The compounds prefer NTS2 over NTS1 but the reason for their in vivo efficacy is not clear based on the presented data. For example, absence of the analgesic effect in a NTS2KO model or maintenance of the analgesic effect in a NTS1KO model needs to be demonstrated. Some genetic evidence is required to claim analgesic action of the compounds in vivo through NTS2. at present the provided experimental evidence is correlative but needs to be causal to make a strong claim. Along the same lines, any future drug discovery effort needs to know what exactly to optimize, NTS2 action over NTS1 or a particular NTS1 signaling profile.***

We fully agree with the reviewer that our current data do not allow us to conclude whether the small molecules, despite displaying higher affinity for NTS_2R than NTS_1R, produce their analgesic effects through NTS_1R, NTS_2R or a combination of both receptors. To further evaluate the contributions of the two receptor subtypes, we employed a pharmacology strategy using two well-characterized antagonists: First, we used **SR142948A**, a non-selective neurotensin receptor antagonist with comparable high affinity for both NTS_1R and NTS_2R (DOI: 10.1016/s0014-2999(97)01510-0, 10.1016/S0022-3565(24)36482-1), to confirm that the analgesic effects of these compounds are mediated through NTS receptors. Second, we used **NTRC844**, a potent and selective NTS_2R antagonist that has previously been shown to abolish the analgesic actions of linear and macrocyclic NTS_2R agonists (PMID: 27359371, 34229249). As illustrated in the Figure R1 below, the analgesic effect of compound **28a**, assessed in both an acute thermal nociception assay (tail-flick) and a tonic inflammatory pain model (formalin test), was fully prevented by **SR142948A** and, importantly, also by the selective NTS_2R antagonist **NTRC844**. These results strongly support the involvement of NTS_2R in mediating the analgesic actions of **28a**. This is consistent with the expression of peripheral NTS_2Rs on sensory neurons and spinal dorsal horn (DOI: 10.1097/j.pain.0000000000002561, 10.1523/JNEUROSCI.0810-05.2005). We have revised the manuscript to incorporate new *in vivo* data demonstrating that the analgesic effects are mediated by NTS_2R. These additions include the addition of new figures (Figure 6e-f), along with corresponding updates to the results (pages 23-24) and methods (pages 58-61) sections.

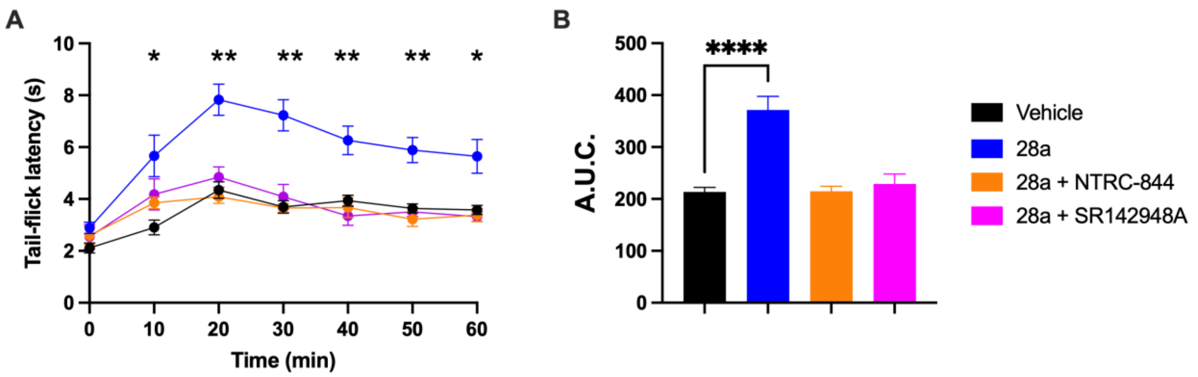

Figure R1. Analgesic effect of compound 28a. (**A-B**) Antinociceptive effects of **28a** in the tail-flick thermal nociception assay, administered alone or in combination with the non-selective neurotensin receptor antagonist **SR142948A** (5,000 nmol/kg) or the selective NTS_2R antagonist **NTRC844** (5,000 nmol/kg). Data are expressed as mean ± SEM (N=5-7/group). Time-course data in the tail-flick assay were analyzed using two-way ANOVA followed by Dunnett's multiple comparisons test. AUC data were analyzed using one-way ANOVA with Dunnett's correction for multiple comparisons. * p<0.05, ** p<0.01 and **** p<0.0001 versus vehicle.

16. Reviewer: ***"The small-molecule agonists essentially lack interactions mimicking those of Arg8, Arg9, and Pro10, likely preventing the stabilization of high agonist-affinity states in wild-type NTS1R. These observations provide directions for further optimization of small-molecule NTS1R agonists." Please comment whether optimized ligands for NTS1 which mimic the interactions of Arg8, Arg9, and Pro10 can still be considered small molecules or will need to be substantially bigger.***

We agree with the reviewer that further optimization of the compounds to form interactions within the pockets occupied by Arg_8–Arg_9–Pro_{10} would lead to increased molecular weight. These observations illustrate challenges inherent in the development of small-molecule agonists of peptide-binding GPCRs, and this important point has been included in the revised discussion (page 31).

17. Reviewer: ***"Our study shows that it is also possible to design orthosteric agonists with distinct signaling signatures, and compound 35a biased NTS1R signaling toward β-arrestin-2." Please rephrase as you have no data showing that you bias NTS1R signaling towards arrestin. arrestins don't signal and the assay you use is a protein-protein interaction assay. Make sure you are semantically precise or, alternatively, use a true arrestin signaling assay for your compounds. Keep in mind that you measure arrestin recruitment in the presence of a G protein-dependent kinase, enhancing G protein-dependence of arrestin recruitment.***

Following the suggestion of the reviewer, we do not mention bias towards arrestin for our compounds in the revised discussion.

18. Reviewer: ***"This scaffold provides a starting point for development of a portfolio of biased NTS1R agonists, which could be used to identify the optimal profiles for different therapeutic areas." please explain the rationale for developing biased NTS1R agonists as all three compounds with distinct signaling signatures have the same in vivo effect most likely originating from NTS2 activation. I would suggest to not claim that biased ligands will have any value as there is no evidence for it. The data rather suggest the opposite, ie that the signaling profile is irrelevant for the in vivo efficacy. I also suggest to delete speculations about bias profiles and different therapeutic areas as the idea of bias in general has not really proven to be successful in the clinic.***

Following the reviewer's suggestion, we have deleted the sentence about the connection between "bias profiles" and different "therapeutic areas". As discussed in Point #13, we instead note that clear connections between specific signaling signatures and the desired pharmacological outcomes *in vivo* remain to be established (pages 31-32).

19. Reviewer: ***"Compound 35a showed exceptionally high affinity for NTS2R and is the first small molecule of this target to show an analgesic effect in vivo." This is very exciting data; with causal evidence that 35a acts through NTS2 but not NTS1 using the respective KO animals, this will be a strong paper.***

We thank the reviewer for this encouraging comment and fully agree that causal validation using NTS_1R- and NTS_2R-deficient mice would provide the most definitive demonstration of receptor-specific mechanisms. Unfortunately, NTS_1R and NTS_2R knockout mouse lines were not available for the *in vivo* assays, preventing a direct genetic assessment of receptor-specific contributions in the present study. To address this limitation (See response to Point #15), we therefore implemented a pharmacological strategy using well-characterized non-selective and NTS_2R-selective antagonists to probe receptor involvement *in vivo*. As detailed in the revised manuscript, the analgesic effects of these small-molecule agonists were fully abolished by the selective NTS_2R antagonist, strongly supporting NTS_2R as the primary mediator of their antinociceptive action. While we cannot formally exclude a contribution of NTS_1R, these data provide compelling functional evidence that NTS_2R activation is required for their analgesic effects. These results have been included on pages 23-24 of the revised manuscript.

Reviewer #5

The fifth reviewer appreciated our work: ***"The study is generally well done and reports novel information"***. We respond to the reviewer's questions below:

Major comments:

1. Reviewer: ***The identified ligands were found to have ~43-360-fold greater affinity for NTS2R than NTS1R, but all the signaling pathway studies were done only with the NTS1R. It would provide more insight into the utility of these ligands as NTS2R agonist analgesics if signaling pathways at NTS2R assessed with these ligands, at minimum for the major (Gq/11) pathway. Findings from these studies would support or oppose the speculation, based on relative binding affinities, that the antinociceptive effects of these ligands are due to NTS2R activation in vivo.***

As suggested by the reviewer, we have done additional functional assays with digested murine heart tissue showing that **28a**, **30a**, and **35a** are able to induce accumulation of IP_1 in experiments with cells from wild-type animals but not NTS_2R knockout-mice. These results have been included as a new Supplementary Figure S8 and on pages 21-22 of the revised manuscript.

2. Reviewer: ***In a related concern, the rationale for focusing on NTS1R in the ligand discovery process was not entirely clear, given that the authors predict the NTS2R would have fewer side effects as a potential analgesic target. The preexisting ligands in Table 2 all appear to have moderate to high affinity for NTS2R. Perhaps this choice was based purely on the availability of structural information on NTS1R, but this is not explicitly stated.***

We thank the reviewer for noting that the rationale for selecting NTS_1R as the target for the virtual screen was not clear. As the reviewer correctly points out, no high-resolution structures of NTS_2R are currently available. Because a large number of NTS_1R structures have been solved, we focused on this subtype in the docking screen. Importantly, the residues anchoring the peptide agonist in the orthosteric site are 75% conserved between NTS_1R and NTS_2R, suggesting that compounds designed from the NTS_1R structure could be relevant for both subtypes. In the revised manuscript, we have included these clarifications in the introduction (pages 3-4) and results sections (page 5).

3. Reviewer: ***The authors mention multiple times that small molecule compounds are more suitable to cross the blood brain barrier and are more likely to be bioavailable than peptides. However, the authors used an intrathecal route of administration in their behavioral assay, which doesn't account for either of these things. At minimum, a rationale for using the intrathecal route should be provided.***

We agree that intrathecal (i.t.) administration does not allow direct assessment of blood–brain barrier penetration or systemic bioavailability. The i.t. route was therefore deliberately chosen to enable a direct evaluation of central analgesic efficacy and mechanism of action, independent of pharmacokinetic and BBB-related variables. This approach is well established in pain research and allows robust target engagement at

spinal and supraspinal sites while minimizing peripheral confounding factors. Our additional pharmacokinetic analyses indicate that these compounds do not cross the blood–brain barrier following systemic administration, which further supports the rationale for using the i.t. route in the present study. In the revised manuscript, we have added the results of the pharmacokinetic study (page 25). As we now know that our compounds do not cross the blood–brain barrier, we do not mention this advantage of small molecules in the discussion of the revised manuscript and that further optimization the pharmacokinetic properties of our scaffold is necessary (page 31).

4. Reviewer: ***A related concern is that physiochemical and ADME properties of the top ligands are not presented. For example, there are computational tools for determining the Lipinski criteria, etc. that could have been applied.***

We thank the reviewer for this suggestion. In the revised manuscript, we present calculated physicochemical properties relevant to Lipinski's rule of five. Encouragingly, two of the three key compounds (**28a** and **30a**) fulfil all Lipinski's criteria for drug-likeness, and we have added these results to the revised manuscript (page 10, Supplementary Table S5).

Minor comments:

5. Reviewer: ***The connection between beta-arrestin2 and desensitization is mentioned twice in the same sentence on p. 11, paragraph 1. "Moreover NTS1R is known to tightly interact closely with β-arrestins that mediate receptor desensitization, which are involved in receptor desensitization, play an important role…"***

We thank the reviewer for identifying this error and corrected the sentence (page 12).

6. Reviewer: ***In the "Analgesic activity of selective high-affinity ligands of NTS2R" section: The sentence referring to the results showing compound affinity for rNTS2R in radioligand binding assays (Supplemental Table S8) may be better discussed in the prior section with the results describing affinity at hNTS2R and selectivity of the compounds.***

As suggested by the reviewer, we have moved the rNTS$_2$R binding date to page 21.

7. Reviewer: ***In paragraph 1 on p. 20: the phrase: "…it is possible that this difference is correlated with the RTI-3a complex is responsible…" is awkwardly worded. Perhaps it should read: "…'and' is responsible…"?***

We thank the reviewer for pointing out that this sentence was not clear. We have updated this section in the revised manuscript (pages 20-21).

8. Reviewer: ***In the radioligand methods descriptions on p. 43-44: Perkin-Elmer radiochemical division is now called Revvity (at least in the U.S.).***

We thank the reviewer for identifying this error, which we have corrected in the revised manuscript (page 49).

Reviewer #6

Reviewer #6 co-reviewed this manuscript with one of the reviewers who provided the listed reports.

Reviewer #7

The seventh reviewer was also enthusiastic about our work and described our manuscript as ***"well concieved"*** with ***"great promise to further the field for not only the neurotensin receptors but also other peptide GPCRs"***. The reviewer had comments to improve the manuscript, which we address below:

Major comments:

1. Reviewer: ***The rationale for optimization of chemical compounds is not very clear, could more information be added to help the reader understand why modifications were made, ie. docking indicated that there was areas within the binding pocket to expand, or maybe modifications were made to interact with additional key residues such as ...***

We thank the reviewer for pointing out that the rationale for ligand optimization was not clear. In a first step, we first explored the possibility of replacing the 5-methylthiophen-2-yl group at position 3. The structure of **RTI-3a** bound to NTS_1R indicated that larger substituents could be accommodated by the TM2-TM7 pocket. Based on docking calculations, we identified compounds that maintained the key interactions with $Y145^{3.29}$ and $R322^{6.54}$, and extended into the TM2-TM7 pocket, *e.g.*, by forming van der Waals interactions with $H343^{7.32}$ and $Y346^{7.35}$. In a second step, we focused on identifying compounds extending into the pockets occupied by Tyr_{11} in the NTS_1R-peptide complex, as the Tyr_{11} side chain has been shown to be essential for high-affinity binding in mutagenesis studies. We selected compounds predicted to interact with residues in the extracellular loops, *e.g.*, $H131^{ECL1}$, $V223^{ECL2}$, and $T225^{ECL2}$, and. In the revised manuscript, we have introduced these additional details regarding our optimization strategy (pages 9-10).

2. Reviewer: ***When testing compounds against different signaling pathways, bias calculations should be included. Additionally, the authors elude to interactions with potential key residues for bias, mutagenesis studies along with MD would help to support their claim and further expand research to identify orthosteric binding pocket biased ligands.***

We agree that it would be highly interesting to identify individual amino acids and or receptor/ligand contacts that are responsible for biased signaling at NTS_1R. However, mutation of key residues alone or in combination with MD are unlikely to provide such insights as **(a)** evidence suggests multiple residues, not single contacts govern the signaling profile of a receptor; **(b)** the intracellular coupling partner is relevant for the signal transduction mechanism, which would ideally require structure determination of multiple active state complexes to provide precise answers (DOI: 10.1016/j.tips.2025.10.008). As mentioned by Reviewer #4, there is not yet clear connection between distinct NTS_1R signaling profiles for and desired antinociceptive *in vivo* activity, and we therefore decided not to include the aforementioned investigations or bias calculations in our manuscript. In the revised manuscript, we

generally less emphasize on biased signaling, and focus more on the differences in intrinsic activity and selectivity, which are more likely to be responsible for the observed *in vivo* effects of our compounds.

3. Reviewer: ***When discussing the peptide receptor focused virtual library, it would be great to have a comparison with a known library. Does it yield better results, what is the hit rate, etc..***

We thank the reviewer for this interesting question. In the revised manuscript, we have added a comparison to the MLSMR library, which contains a diverse set of compounds has been used in hundreds of high-throughput screens. In one of these campaigns (DOI: 10.1021/ml400176n), a screen of 331,861 compounds was performed with the goal to identify NTS_1R agonists. Of these, 184 compounds showed activity (>40% of NTS at 20 μM), corresponding to a hit rate of 0.06%. For comparison, our virtual screens of the diverse and focused library resulted in hit rates of 4% and 38%, illustrating the efficiency of virtual screening and the use of focused libraries. We have added this analysis in the discussion of the revised manuscript (page 28).

4. Reviewer: ***When comparing different ligands with SBI-553, it would be great to have the allosteric parameters along with comparison of curves. The probe effect could be related to the compound bias.***

We thank the reviewer for this suggestion. In fact, we did not compare the ligands with **SBI-553**, but we performed ligand binding assays with the fluorescent allosteric probe **PJ125** (derived from SBI-553) and titrated our newly identified orthosteric agonists. We analyzed the data using a standard non-linear regression model implemented in GraphPad Prism 10.1, yielding estimates for EC_{50} and E_{max}, describing the potency (related to affinity) and efficacy (related to cooperativity) for the enhancement of binding of the allosteric probe. We have considered analyzing these data with the "allosteric modulator titration model" to deduce α as a quantitative parameter for the cooperativity between our orthosteric agonists and **PJ125**. However, the allosteric ligand binding model is designed for the inverse assay setup (labeled orthosteric probe and titration of the modulator), and requires significant specific binding of the probe as well as its affinity (K_D) in the absence of the non-labeled compounds to accurately fit the data. As we have previously shown (DOI: 10.1021/acsptsci.4c00086), **PJ125** shows negligible specific binding in the absence of an orthosteric agonist, and hence it is not possible to deduce its K_D value under these conditions. Without these parameters, a reliable quantification with the "allosteric modulator titration model" is not possible, which is why we refrained from including this analysis in our manuscript.

5. Reviewer: ***Much discussion in the field regarding neurotensin receptors involved in pain response is related to whether the pain signal is commented at the neuron level or at the site of injury. PK studied of the new NTSR2 compound which show antinociceptive properties to indicate if it is peripherally restricted or passes the blood brain barrier would a good along way in helper other researchers.***

As suggested by the reviewer, we characterized the pharmacokinetic profile of compound **28a** in rats. Following intravenous administration of 5 mg/kg, total plasma concentrations declined from 98 µM at 5 minutes to 0.1 µM at 6 hours, and were below

the lower limit of quantification (LLOQ) at 24 hours. The estimated plasma half-life, terminal volume of distribution, and clearance of compound **28a** was 2.8 hours, 1.4 L/kg, and 90 mL/h, respectively. Cerebrospinal fluid (CSF) and brain tissue were collected between 2 and 24 hours post-administration. Concentrations of compound **28a** were below LLOQ in all brain samples (< 4 nM) and in the majority of CSF samples (< 2 nM)**.** These results indicate that compound **28a** is largely peripherally restricted and does not cross the blood-brain barrier to a significant extent. We have added these new results to the revised manuscript (page 25). Considering that our compounds are the first small molecule agonists of NTS_2R to shown analgesic effect *in vivo*, our scaffold represents a promising starting point for further optimization of blood-brain barrier penetration and other pharmacokinetic properties. We have added the need for further optimization in the discussion of the revised manuscript (page 31).

May 12th, 2026

We want to thank the referees for taking the time to review our manuscript (*"Docking of virtual libraries identifies small-molecule agonists of neurotensin receptors with analgesic activity"*, Manuscript: NCOMMS-25-28689A) and the many constructive suggestions. Only one of the seven reviewers had additional suggestions on how to improve the revised manuscript. Please find below responses to the referee with the comments in ***Blue***.

Reviewer #4

The reviewer was ***"largely satisfied with the revision"***. The reviewer requested that we place more emphasis on NTS_2R in the abstract, and we have addressed this point in the revised manuscript.

1. Reviewer: ***I believe that, now that it is clear that the analgesic activity is mediated through NTSR2, the abstract should place less emphasis on NTSR1 and on signaling fingerprints that are not relevant to the in vivo effects. The current data suggest that, although the initial goal was to identify NTSR1-targeted compounds, the observed robust in vivo effects resulted instead from activity at NTSR2. This point should be communicated more clearly in the abstract. Even if these findings arose partly through serendipity, the abstract should reflect this outcome with greater transparency and precision.***

As suggested by the reviewer, we have modified the abstract and added that the antinociceptive activity is consistent with signalling via the NTS_2R.
